# Identification of *Nicotiana benthamiana* microRNAs and their targets using high throughput sequencing and degradome analysis

**DOI:** 10.1186/s12864-015-2209-6

**Published:** 2015-12-01

**Authors:** Ivett Baksa, Tibor Nagy, Endre Barta, Zoltán Havelda, Éva Várallyay, Dániel Silhavy, József Burgyán, György Szittya

**Affiliations:** Institute of Plant Biotechnology, National Agricultural Research and Innovation Centre, Agricultural Biotechnology Institute, Szent-Györgyi Albert ut 4, H-2100 Gödöllő, Hungary

**Keywords:** *Nicotiana benthamiana*, microRNA, Non-coding RNAs, Small RNA, Degradome, High throughput sequencing

## Abstract

**Background:**

*Nicotiana benthamiana* is a widely used model plant species for research on plant-pathogen interactions as well as other areas of plant science. It can be easily transformed or agroinfiltrated, therefore it is commonly used in studies requiring protein localization, interaction, or plant-based systems for protein expression and purification. To discover and characterize the miRNAs and their cleaved target mRNAs in *N. benthamiana*, we sequenced small RNA transcriptomes and degradomes of two *N. benthamiana* accessions and validated them by Northern blots.

**Results:**

We used a comprehensive molecular approach to detect and to experimentally validate *N. benthamiana* miRNAs and their target mRNAs from various tissues. We identified 40 conserved miRNA families and 18 novel microRNA candidates and validated their target mRNAs with a genomic scale approach. The accumulation of thirteen novel miRNAs was confirmed by Northern blot analysis. The conserved and novel miRNA targets were found to be involved in various biological processes including transcription, RNA binding, DNA modification, signal transduction, stress response and metabolic process. Among the novel miRNA targets we found the mRNA of REPRESSOR OF SILENCING (ROS1). Regulation of ROS1 by a miRNA provides a new regulatory layer to reinforce transcriptional gene silencing by a post-transcriptional repression of ROS1 activity.

**Conclusions:**

The identified conserved and novel miRNAs along with their target mRNAs also provides a tissue specific atlas of known and new miRNA expression and their cleaved target mRNAs of *N. benthamiana*. Thus this study will serve as a valuable resource to the plant research community that will be beneficial well into the future.

**Electronic supplementary material:**

The online version of this article (doi:10.1186/s12864-015-2209-6) contains supplementary material, which is available to authorized users.

## Background

*Nicotiana benthamiana* is a widely used model plant species for research on plant-pathogen interactions as well as other areas of plant science. It is particularly popular among plant virologist as it is susceptible to a large number of different plant viruses. It is also widely used in studies requiring protein localization, interaction, or plant-based systems for protein expression and purification. In addition, its susceptibility to pathogens has been used to transiently express proteins, using either engineered viruses or syringe-infiltration of *Agrobacterium tumefaciens* [1, 2]*.* The syringe-infiltration method proved to be extremely powerful tool to identify and characterize many viral silencing suppressor proteins [3], pathogens effector proteins [4] and also components of the plant nonsense-mediated mRNA decay system [5].

*N. benthamiana* is an herbaceous plant endemic to Australia [2], belonging to the Suaveolentes section of the *Nicotiana* genus of Solanaceae family. *N. benthamiana* is an allotetraploid species with 19 chromosome pairs, resulting from the hybridization of two unknown progenitors. The estimated genome size of *N. benthamiana* is 3 GB [2]. The draft genome sequence of the *N. benthamiana* Nb-1 accession has been published recently [1]*.* The origins of the *N. benthamiana* accessions used for research are generally not known. However it has been suggested that only a single accession of *N. benthamiana*, or a collection of closely related accessions, are being used by the plant research community today, since they are very similar in both susceptibility to pathogens and also in phenotype [2]. The genomes of economically important close relatives of *N. benthamiana*, such as potato (*Solanum tuberosum*), tomato (*Solanum lycopersicum*) and pepper (*Capsicum annuum*), genomes have been also sequenced recently [6–9]. These species has either smaller or very similar genome size to *N. benthamiana*. The Potato *(Solanum tuberosum)* genome size is 844 Mb [6], the tomato *(Solanum lycopersicum)* genome size is approximately 900 Mb [8] and both species has twelve chromosomes. Other economically important members of the *Solanaceae* family is the pepper and the eggplant. The widely cultivated *C. annuum* accession *Zunla-1* genome size is estimated to be 3.26 Gb [9], while the genome size of the eggplant is approximately 833.1 Mb [10].

Gene expression is regulated at several layers to ensure optimal spatial and temporal accumulation of proteins. RNA silencing is an evolutionarily conserved, important gene expression regulator of various cellular processes in eukaryotes, whereby 21-24 nt small RNAs (sRNAs) direct silencing of endogenous genes or pathogens. Plant sRNAs have two major categories based on their biogenesis: small interfering RNA (siRNA) processed from perfectly double stranded RNA; and microRNA (miRNA) derived from single stranded RNA transcripts that form imperfectly double-stranded stem loop precursor structures [11]. *MIR* genes encode the precursor transcripts which are cleaved into miRNAs by Dicer-like 1 (DCL-1), and subsequently loaded into an **R**NA **I**nduced **S**ilencing **C**omplex (RISC), containing an ARGONAUTE (AGO) protein. miRNA guided RISCs are able to inhibit expression of genes with at least partial homology to the miRNA by either transcript cleavage or translational arrest [12]. miRNAs have important roles in different cellular processes including developmental/ physiological processes and abiotic/biotic stress [13, 14]. miRNAs are classified into families, whereby miRNAs with the same or very similar sequences are grouped into the same family. It has been shown that only a small number of miRNA families are present across phylogenetically distant species [15–17]. The majority of plant miRNAs are evolutionarily young and are only present in one to few species and it is likely that *MIR* loci in plant genomes are in a constant dynamic evolutionary state [15, 16].

Although the draft genome of *N. benthamiana* is available and transcriptome-wide expression studies have been reported [1, 18–20], the miRNA profile of *N. benthamiana* has not been analysed. In this study, we used a comprehensive molecular and bioinformatic approach to detect and experimentally validate miRNAs and their target mRNAs from five different tissues of two *N. benthamiana* lines (the *N. benthamiana* plant, which is used in our laboratory and the sequenced Nb-1 accession). In our study, we used Illumina sequencing to identify small RNAs, their cleaved target mRNAs followed by Northern blot validation of the miRNAs. Furthermore, we examined cleaved miRNA targets from five different tissues of two aforementioned *N. benthamiana* accessions. We identified 40 conserved and 18 novel microRNAs and validated their target mRNAs in *N. benthamiana* with a genomic scale method. The targets were found to be involved in various biological processes including transcription, RNA binding, DNA modification, signal transduction, stress response and metabolic processes. We anticipate that this study will serve as a valuable resource of *N. benthamiana* miRNAs and their validated target mRNAs to the whole plant research community.

## Results and discussion

### High-throughput sequencing of *N. benthamiana* small RNAs

**Fig. 1:**
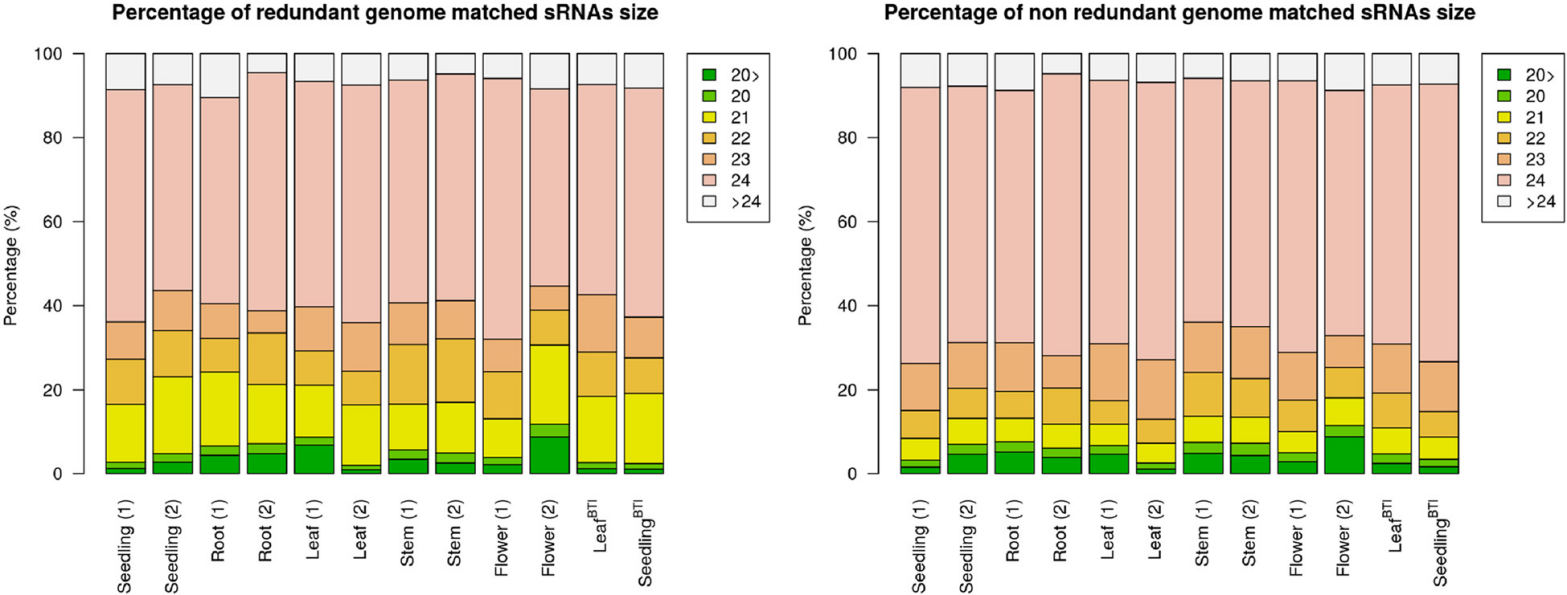
The size distribution of sequenced small RNA reads. The size class distribution of redundant and non redundant small RNA sequences in the twelve tissue samples. The percentage of the different size classes (y-axis) and the different tissues, which includes seedling, root, leaf, stem and flower with biological replicates and BTI seedling and leaf cDNA libraries (x-axis) represented in *N. benthamiana*

To identify and comprehensively describe the expression of endogenous sRNAs in *N. benthamiana*, sRNAs containing 5′-phosphate and 3′-OH (likely to be a products of Dicer-like proteins) were cloned from roots, stems, leaves, flowers and small seedlings (containing both root tissue and aerial parts) of *N. benthamiana* plants. The libraries were then sequenced by high-throughput Illumina sequencing. By using biological replicates of five tissues, ten cDNA libraries were made from the *N. benthamiana* line that is used in our laboratory. To compare the sRNA profile of our *N. benthamiana* line and the line, which has been sequenced (Nb-1 accession), two additional sRNA libraries were prepared and sequenced from the leaves and small seedlings of Nb-1 accession. These twelve cDNA libraries yielded approximately 90 million reads. Sequences flanked by the 5′ and 3′ Solexa adaptors, with a minimum and maximum length of 16 and 28 nt, respectively, were compared with the draft *N. benthamiana* genome sequences. More than 15 million non-redundant reads were perfectly matched to at least one locus, and were analysed further (Table 1). The size distribution of sequence reads showed that the majority of sRNAs in our libraries belonged to the 24 nt size class and it was followed by the 21–22 nt size classes (Fig. 1). Our sequence data are in agreement with previous observations which show that in plants, this 24 nt size class is the overall most abundant class of sRNAs [15].

**Table 1:** High-throughput sequencing statistics of *N. benthamiana* sRNAs

		Redundant		Non-redundant				Redundant		Non-redundant	
		Reads	Matching *N. benthamiana* genome	Reads	Matching *N. benthamiana* genome			Reads	Matching *N. benthamiana* genome	Reads	Matching *N. benthamiana* genome
Seedling 1	Raw reads	4 353 615				Stem 1	Raw reads	2 673 839			
	Adaptor removed	3 192 563		1 106 502			Adaptor removed	2 468 386		858 314	
	Filter by sequence properties	3 187 558		1 102 305			Filter by sequence properties	2 464 474		854 744	
	t/rRNA removed	3 034 009	2 791 655	1 085 271	942 175		t/rRNA removed	2 404 489	2 205 954	846 701	746 712
	Match known miRNAs	262 998	259 394	1 531	792		Match known miRNAs	121 361	119 819	1 376	860
Seedling 2	Raw reads	13 565 620				Stem 2	Raw reads	13 390 095			
	Adaptor removed	9 931 841		1 048 202			Adaptor removed	12 682 160		3 280 430	
	Filter by sequence properties	9 916 497		1 036 381			Filter by sequence properties	12 662 117		3 263 087	
	t/rRNA removed	9 461 135	8 656 790	1 016 371	841 123		t/rRNA removed	12 195 396	11 213 510	3 246 517	2 773 011
	Match known miRNAs	1 132 848	1 117 758	2 423	1 030		Match known miRNAs	664 082	654 984	3 455	1 969
Root 1	Raw reads	4 416 396				Flower 1	Raw reads	17 715 434			
	Adaptor removed	3 567 326		673 485			Adaptor removed	15 107 462		4 920 011	
	Filter by sequence properties	3 561 838		669 035			Filter by sequence properties	15 084 084		4 899 263	
	t/rRNA removed	3 250 883	2 926 826	652 495	553 372		t/rRNA removed	14 374 989	13 270 386	4 872 774	4 186 250
	Match known miRNAs	368 908	363 793	1557	827		Match known miRNAs	517 965	510 930	3 461	2 128
Root 2	Raw reads	6 227 488				Flower 2	Raw reads	8 784 135			
	Adaptor removed	5 472 237		2 020 365			Adaptor removed	8 156 033		2 71814	
	Filter by sequence properties	5 463 791		2 013 081			Filter by sequence properties	8 143 554		262 570	
	t/rRNA removed	5 041 719	4 673 608	1 992 006	1 746 022		t/rRNA removed	7 295 596	6 508 891	253 399	185 299
	Match known miRNAs	308 179	304 276	2 402	1 474		Match known miRNAs	817 620	807 317	1 526	547
Leaf 1	Raw reads	4 481 051				Leaf ^BTI^	Raw reads	2 646 118			
	Adaptor removed	3 752 733		1 675 147			Adaptor removed	2 373 816		445 460	
	Filter by sequence properties	3 746 943		1 670 236			Filter by sequence properties	2 370 062		442 372	
	t/rRNA removed	3 567 987	3 252 343	1 644 909	1 440 195		t/rRNA removed	2 277 189	2 092 901	433 297	375 749
	Match known miRNAs	258 610	255 179	2 368	1 502		Match known miRNAs	234 022	231 082	1 025	489
Leaf 2	Raw reads	3 801 050				Seedling ^BTI^	Raw reads	7 874 296			
	Adaptor removed	2 925 945		1 149 340			Adaptor removed	7 174 326		2 105 046	
	Filter by sequence properties	2 921 408		1 145 456			Filter by sequence properties	7 163 048		2 095 876	
	t/rRNA removed	2 834 354	2 596 026	1 131 710	982 432		t/rRNA removed	6 906 776	6 088 762	2 070 845	287 724
	Match known miRNAs	274 028	270 476	1 434	737		Match known miRNAs	786 266	423 581	2205	113

### Identification of known and conserved miRNAs in *N. benthaniana*

**Fig. 2:**
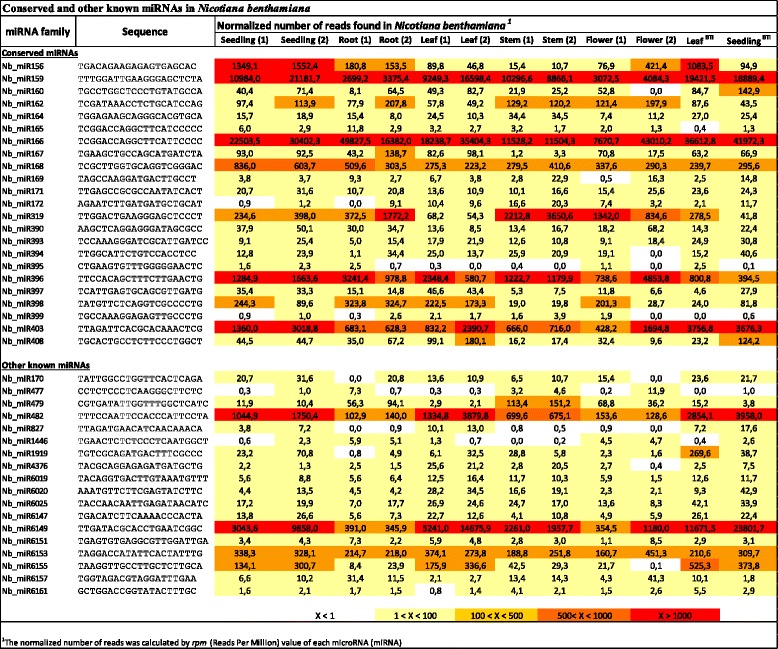
Conserved and other known miRNAs in *N. benthamiana*. Normalized read numbers of conserved and other known miRNAs across the tissues included in this study. Expression profiles are expressed in reads per million (RPM) genome matching reads. Heat map colours represents absolute normalized levels of miRNA expression ranging from less than 1 RPM (white) to more than 1000 RPM (red) as indicated in the colour key. We have identified 40 known or conserved miRNA families in the twelve sRNA libraries generated. All of the deeply conserved miRNA families (miR156/157, miR159/319, miR160, miR165/166, miR171, miR408, miR390/391 and miR395) were present in our data sets. The expression levels of different miRNA families were different and we also observed clear tissue-specific expressional changes within some miRNA families as it was expected

miRNAs are a well-studied class of regulatory sRNAs. They are present in all plant species studied so far and have important functions in plant development and stress responses. Conserved families of miRNAs were found in many plant species and analysis of data from high-throughput sequencing approaches identified eight deeply conserved miRNA families that are present in all embryophytes [16]. First we looked for known miRNAs by comparing our libraries to known miRNAs from other plant species and we identified 40 known or conserved miRNA families in the twelve sRNA libraries generated (Fig. 2, Additional file 1: Table S1). As expected, all of the deeply conserved miRNA families (miR156/157, miR159/319, miR160, miR165/166, miR171, miR408, miR390/391 and miR395) were present in our data sets (Fig. 2). We analysed the number of reads for conserved miRNAs and miR156, 159, 166, 168, 319, 396 and 403 were represented most frequently in the libraries (Fig. 2). As expected miRNA families expressed at very different levels and some miRNA families showed clear tissue-specific expression changes (Fig. 2). For example, the expression level of miR159 and miR166 families were high, while the expression of miR395 and miR399 families were similarly low in all tissues (Fig. 2). However, for miR395 and miR399 low expression is not surprising because their expression level is generally low under normal growth conditions as they are induced by sulphur or phosphate deficiency respectively [21, 22]. A recent comprehensive atlas of miRNAs from 31 representative species across vascular plants identified 82 known miRNA families that were present across several terrestrial plants. These families formed eight groups depending on their distribution across lineages and species. miRNAs belonging to group 1 were ubiquitous and generally highly expressed across all terrestrial species (miR156, miR166, miR167, miR168 and miR172) while group 2 miRNA families were represented in all taxonomic lineages but could be absent or present at very low abundance levels in some species (miR158, miR159, miR160, miR162, miR164, miR169, miR171, miR319, miR390, miR393, miR394, miR396, miR397, miR529, miR535 and miR4414) [15]. Most of the group 1 and group 2 miRNA families were present in our data sets except the miR529, miR535 and miR4414 families (Fig. 2). Similar to our result, these three miRNA families were absent or their abundance was very low (1–10 reads per million (RPM) or in some cases 11–100 RPM) in sRNA libraries prepared from different Solanaceae species such as petunia, pepper, potato, tomato and tobacco [15]. Furthermore, the remaining six miRNA groups were distributed across species with diverse lineage enrichment and one of them (group 8, containing miR1919, miR4376, miR5300, miR53001, miR6022, miR6023, miR6024, miR6025, miR6026, miR6027 and miR6149) was shown to be predominant in Solanaceae species [15]. As expected, in our libraries we identified several miRNA families that belong to group 8 such as miR1919, miR4376, miR6025 and miR6149. Among the group 8 miRNA families the miR6149 abundance level was the highest in our libraries similar to the tobacco libraries reported by Chavez Montes et al. [15]. Some of the group 8 miRNA families were missing from our libraries (miR5300, miR5301, miR6022, miR6023, miR6024, miR6026 and miR6027), however those miRNA families also showed low abundance (0–100 RPM) in the reported tobacco libraries (such as miR5300, miR5301, miR6023 and miR6027) [15].

We also identified several miRNA families which were identified previously as tobacco (*N. tabacum*) specific miRNAs such as miR6019, miR6020, miR6147, miR6151, miR6153, miR6155, miR6157 and miR6161 [23, 24]. These miRNA families generally showed low abundance across all tissues in our libraries with the exception of miR6153, which was expressed at medium levels in all samples (160–450 RPM across all tissue) and miR6155, which was relatively abundant in leaf and seedling samples (175–525 RPM) (Fig. 2).

**Fig. 3:**
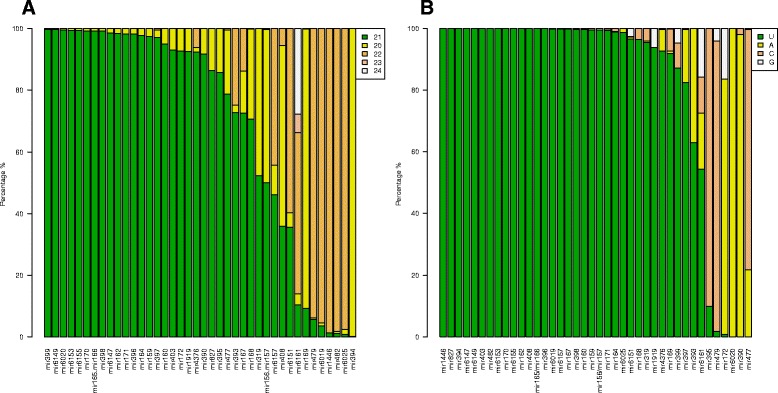
Size distribution and starting nucleotide of the conserved and other known miRNAs. The relative abundance of different size categories (**a**), from 20 to 24 nucleotides is shown for the conserved and other known miRNAs presented in Fig. 2. The relative abundance of the 5′-nucleotide (**b**) is shown for the conserved and other known miRNAs presented in Fig. 2. In 31 miRNA families, 21 nt long miRNAs were the most significant size class. In 32 of the 40 conserved miRNA families, the majority of sequences started with uracyl at their 5′-end, although a portion of these have a different nucleotide composition at position 1 to a variable degree. Three miRNA families exhibited a preference for an adenine at position 1

We checked the size distribution and starting nucleotide of the conserved and known miRNA families in our libraries. In 30 miRNA families, 21 nt long miRNAs were the most significant size class (Fig. 3a). In several cases they were accompanied by additional size classes, mostly 20 nucleotide long sequences or in some cases 22 nucleotide sequences were present in significant proportions (miR4376, miR393, miR167 and miR6157). Three miRNA families exhibited a strong preference for 20 nucleotide sequences and in seven miRNA families the 22 nucleotide long sequences were the most abundant size class (Fig. 3a). Our observation that the miR394 has a predominant 20 nucleotide size in our libraries is consistent with previous result and it might suggest a functional consequence since its size and sequence is conserved in terrestrial plant species [15]. It was shown in several cases that size specificity has a functional consequence, like the 22 nucleotide preference of phase initiating miRNAs associated with the generation of tasiRNAs [25, 26]. We found seven miRNA families with the 22 nucleotide sequence dominance and two of them (miR6019 and miR482) belongs to the described phase initiating miRNAs [23, 27, 28]. It is likely they are also involved in tasiRNA generation in *N. benthamiana*.

Sorting of sRNAs into AGO complexes is influenced by their 5′ terminal nucleotide. Hence, we checked the 5′-nucleotide distribution of the conserved and known miRNA families in our libraries, since it is an important feature that is correlating to their biogenesis and function [29]. In 34 of the 40 conserved miRNA families, the majority of sequences started with uracyl at their 5′-end, although a portion of these have a different nucleotide composition at position 1 to a variable degree (Fig. 3b). Three miRNA families exhibited a preference for an adenine at position 1, although one of them has a different nucleotide composition at position 1 to a variable degree (miR172). In three miRNA families the most frequently found first nucleotide at their 5′-end was cytosine (miR395, miR479, miR477). miRNAs mostly are incorporated into the AGO1 effector complex to give sequence specificity to either slice or translationally repress their target mRNAs [12]. AGO1 harbours miRNAs that favour a 5′ terminal uridine [30]. In line with this observation, most of the conserved and known miRNA families in our libraries started with uracyl at their 5′-end. We also found several miRNAs that start with either adenine or cytosine (miR172, miR390 and miR395). It was reported in many different plant species that miRNAs belonging to these miRNA families are starting with the same nucleotide at the 5′-position [15].

**Fig. 4:**
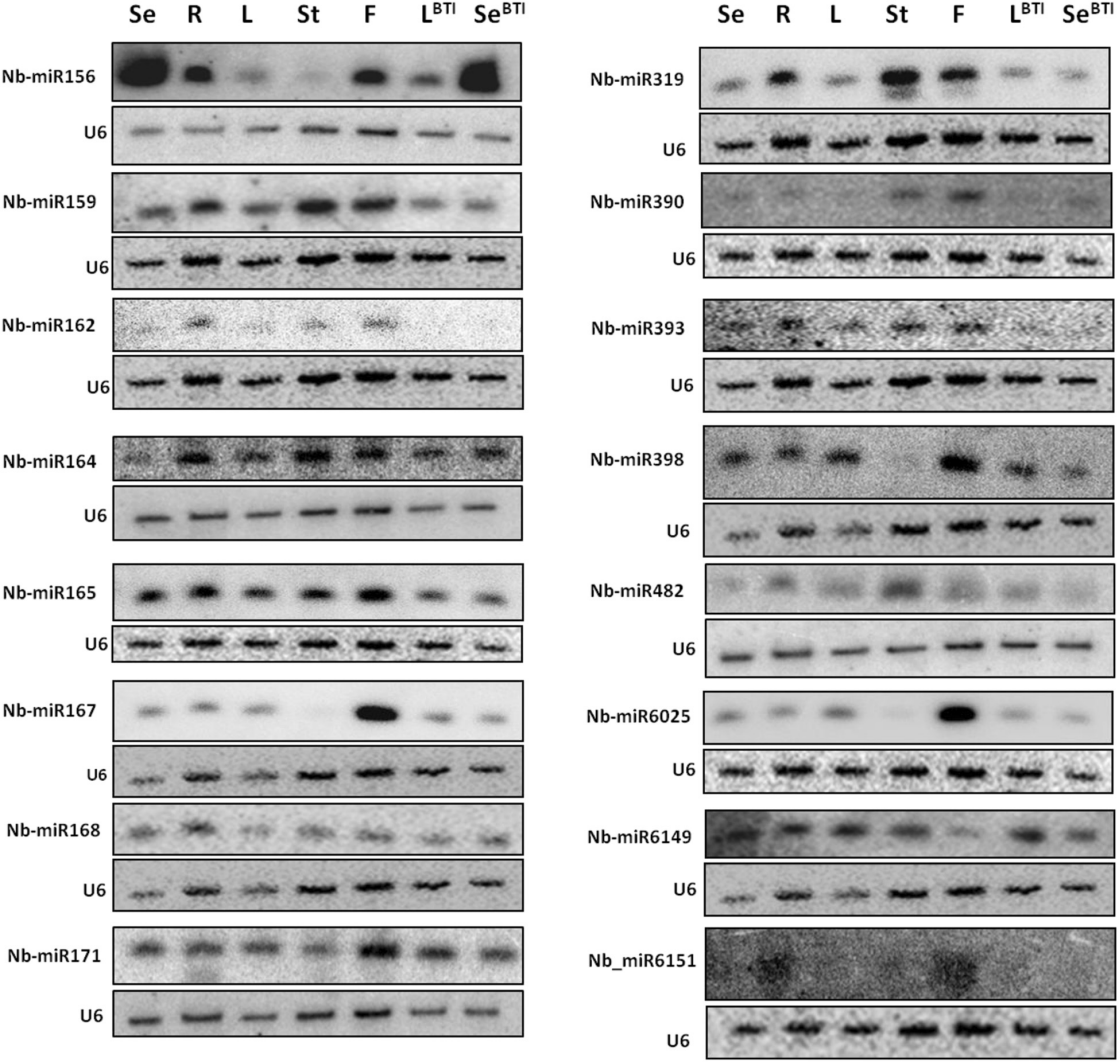
Expression analysis of selected conserved and other known miRNAs in different *N. benthamiana* tissues. Total RNA was extracted from different tissues including, seedling (Se), root (R), leaf (L), stem (St), flower (F) from *N. benthamiana* plants used in our laboratory and from plants from Boyce Thompson Institute (leaf^BTI^ - L^BTI^, seedling^BTI^ - Se^BTI^). The RNA was separated on PAGE and transferred to nylon membranes for Northern blot analysis of the miRNAs. Oligonucleotide probes were used to detect specific miRNAs, and an U6-specific probe was used to detect U6 RNA as a loading control for each membrane

Many plant miRNAs show tissue-specific and developmental-stage-specific expression patterns and they have important roles during development and stress adaptation [13]. To validate the tissue-specific expression patterns of selected conserved and known miRNAs we carried out Northern blot analysis using seedling (Se), root (R), leaf (L), stem (St) and flower (F) samples isolated from our *N. benthamiana* line; and leaf (L^BTI^) and seedling (Se^BTI^) samples from *N. benthamiana* Nb-1 accession that were obtained from the Boyce Thompson Institute for Plant Research. Northern analysis usually was in broad agreement with the sequencing data as in the cases of miR156, miR159, miR162, miR164, miR165, miR168, miR390 and miR393 (Fig. 4). In certain cases we observed a discrepancy between the Northern blot and sequencing data, as in the cases of miR167, miR171, miR482, miR6025 and miR6149 (Fig. 4; Fig. 2). However, these differences are not unexpected as it has been reported that Northern analysis are not always in full agreement with the sequencing data, presumably due to the inherent biases in next-generation sequencing technologies [31–36]. Theoretically, the oligoprobes used for Northern analysis might have detected heterogeneous miRNAs. However, we expect that Northern analysis should reveal more accurate *in vivo* expression patterns. We also performed RT-qPCR analysis to validate our Northern results by checking the expression pattern of miR167 and miR482 in *N. benthamiana* tissues. We found that the results of Northern analysis and RT-qPCR were identical (Additional file 2 Figure S1.). Furthermore, comparing the Northern analysis data of our *N. benthamiana* line to the Nb-1 accession to detect tissue specific expression pattern of miRNAs, we were not able to confirm the differences occasionally observed in the miRNA read numbers at certain miRNA families (in seedlings: miR156, miR168, miR319, miR396, miR482 and miR6149; in leaf: miR156, miR319, miR398, miR403 and miR1919) (Fig. 4). Our miRNA expression data therefore might indicate that these *N. benthamiana* lines are closely related based on their very similar miRNA expression patterns.

### Targets of conserved and known miRNAs

To generate a miRNA cleaved target library (degradome) from *N. benthamiana* we applied a high-throughput experimental approach that can identify mRNAs targeted by sRNAs [37]. The poly-A fraction of total RNA extracted from seedling (Se), root (R), leaf (L), stem (St), flower (F) of *N. benthamiana* plants; and leaf (L^BTI^) and seedling (Se^BTI^) of the Nb-1 accession were analysed for the identification of target transcripts of conserved, other known, and new miRNAs.

We obtained a total number of 47 million short sequencing reads representing the 5′ ends of uncapped, poly-adenylated RNAs. After initial processing, equal numbers of 20- and 21-nt sequence reads were obtained, and 48 % of the short sequencing reads could be mapped to the *N. bentamiana* transcriptome.

In plants, miRNA mediated mRNA cleavage is highly specific and miRNAs have been shown to bind with near perfect complementarity to their mRNA targets, which generally leads to the slicing of the mRNA between positions 10 and 11 of the AGO1 bound miRNA. As a consequence, the cleaved mRNA targets should have distinct peaks in the degradome sequence tags at the predicted cleavage site relative to the other regions of the transcript [38, 39]. In our analysis we have applied the PAREsnip pipeline [40] to identify cleaved targets for both known (conserved and non-conserved) and new miRNAs in *N. benthamiana*. Abundance of the sequenced tags was plotted on each transcript and the results are shown on Additional file 3: Figure S2. The cleaved target transcripts have been categorized into five classes (categories 0, 1, 2, 3 and 4) as it was defined previously in CleaveLand (version2) [41]. In our target list we kept high-confidence miRNA-target gene interactions (categories 0, 1, 2) and only category 3 targets as low–confidence miRNA-target pairs. Category 4 targets are defined as only one raw read at the expected cleavage position, however it might also be the result of the degradation of the target RNA, therefore they were omitted from our target list. This approach identified a total of 55 target mRNAs, for conserved and other known miRNAs (Table 2, Additional file 4: Table S2).

**Table 2:** Targets of conserved and other known miRNAs in *Nicotiana benthamiana* ^a^

miRNA family	miRNA sequence	Gene ID	Clevage position	Category	Normalized abundance^a^	Annotation
Conserved miRNAs and their targets						
Nb_miR156	TGACAGAAGAGAGTGAGCAC	comp77119_c0_seq6 2667_2942	1787	0	2,40	Squamosa promoter-binding-like protein 6
Nb_miR156	TGACAGAAGAGAGTGAGCAC	comp69399_c0_seq4 393_944	650	2	1,28	Squamosa promoter-binding-like protein 16
Nb_miR159	TTTGGATTGAAGGGAGCTCTA	comp71582_c0_seq2 1_1230	673	0	40,63	No annotation^b^
Nb_miR159	TTTGGATTGAAGGGAGCTCTA	comp69815_c0_seq4 1_894	601	0	0,39	Transcription factor GAMYB
Nb_miR159	TTTGGATTGAAGGGAGCTCTA	comp73942_c0_seq1 927_2375	1839	0	2,14	Transcription factor GAMYB
Nb_miR160	TGCCTGGCTCCCTGTATGCCA	comp78286_c0_seq3 693_2783	2065	0	307,20	Auxin response factor 18
Nb_miR160	TGCCTGGCTCCCTGTATGCCA	comp79462_c0_seq1 2477_3379	2658	0	6,41	Auxin response factor 16
Nb_miR164	TGGAGAAGCAGGGCACGTGCA	comp72325_c0_seq2 310_1305	994	0	3,43	Protein CUP-SHAPED COTYLEDON 2
Nb_miR165/166	TCGGACCAGGCTTCATTCCCC	comp80060_c0_seq5 1298_3817	1868	0	51,20	Homeobox-leucine zipper protein REVOLUTA
Nb_miR165/166	TCGGACCAGGCTTCATTCCTC	comp79517_c0_seq6 1043_3556	1601	0	246,61	Homeobox-leucine zipper protein ATHB-15
Nb_miR165/166	TCGGACCAGGCTTCATTCCTC	comp79347_c3_seq8 336_611	546	1	1,60	No annotation^b^
Nb_miR167	AGATCATGTGGTAGCTTCACC	comp79742_c0_seq4 124_2421	1700	2	5,90	5-methyltetrahydropteroyltriglutamate--homocysteine methyltransferase^b^
Nb_miR167	AGATCATGTGGTAGCTTCACC	comp74516_c0_seq2 292_2619	1160	2	3,54	Subtilisin-like protease^b^
Nb_miR168	TCGCTTGGTGCAGGTCGGGACC	comp79927_c1_seq7 1_243	876		1,60	Protein argonaute 1A
Nb_miR169	TAGCCAAGGATGACTTGCCT	comp72459_c1_seq2 1230_1673	1724	2	0,19	Nuclear transcription factor Y subunit A-5/CCAAT-binding transcription factor
Nb_miR169	TAGCCAAGGATGACTTGCCT	comp72338_c0_seq4 1046_1396	1439	2	0,29	Nuclear transcription factor Y subunit A-5/CCAAT-binding transcription factor
Nb_miR169	TAGCCAAGGATGACTTGCCT	comp73705_c1_seq8 1254_1739	1836	1	3,15	Nuclear transcription factor Y subunit A-8/CCAAT-binding transcription factor
Nb_miR169	TAGCCAAGGATGACTTGCCT	comp67524_c0_seq9 544_1167	1246	0	1,96	Nuclear transcription factor Y subunit A-10/CCAAT-binding transcription factor
Nb_miR171	TTGAGCCGCGCCAATATCACG	comp76988_c0_seq2 277_1911	896	0	26,05	Scarecrow-like protein 15
Nb_miR171	TTGAGTCGCGCCAATATCACT	comp80119_c0_seq4 672_2573	1552	0	34,34	Scarecrow-like protein 6
Nb_miR171	TTGAGTCGCGCCAATATCACT	comp75200_c0_seq2 459_2717	1696	0	109,89	Scarecrow-like protein 6
Nb_miR172	AGAATCTTGATGATGCTGCAG	comp78340_c2_seq4 552_1409	2200	0	4,42	Floral homeotic protein APETALA 2
Nb_miR319	TTGGACTGAAGGGAGCTCCCT	comp68096_c0_seq1 79_1191	1046	0	1,02	Transcription factor TCP4
Nb_miR319	TTGGACTGAAGGGAGCTCCCT	comp73714_c2_seq12 2093_2317	2006		0,98	Transcription factor TCP2
Nb_miR393	TCCAAAGGGATCGCATTGATCC	comp80206_c0_seq2 468_4724	4235	2	0,29	No annotation^b^
Nb_miR394	TTGGCATTCTGTCCACCTCC	comp60101_c0_seq1 5351_5605	5291	2	2,16	Tripeptidyl-peptidase 2^b^
Nb_miR395	CTGAAGTGTTTGGGGGAACTC	comp75863_c0_seq1 200_1609	553	2	0,53	ATP sulfurylase 1, chloroplastic
Nb_miR395	CTGAAGTGTTTGGGGGAACTC	comp29318_c1_seq1 108_620	254	0	1,07	Sulfate transporter 2.1
Nb_miR396	TTCCACATCTTTCTTGAACTG	comp71133_c0_seq1 1089_1355	935	2	2,10	Protein THYLAKOID FORMATION1, chloroplastic^b^
Nb_miR396	TTCCACAGCTTTCTTGAACTG	comp77639_c0_seq4 942_2765	1723	0	4,42	Growth-regulating factor 6
Nb_miR396	TTCCACAGCTTTCTTGAACTG	comp67678_c0_seq1 205_573	279	0	12,19	Thioredoxin H-type 1^b^
Nb_miR396	TTCCACAGCTTTCTTGAACTG	comp50987_c1_seq1 2_379	144	1	0,48	40S ribosomal protein S15^b^
Nb_miR396	TTACACAGCTTTCTTGAACTG	comp69468_c0_seq1 204_1724	1425	2	0,58	Protein IQ-DOMAIN 14^b^
Nb_miR396	TTCCACAGCTTTCTTGAACTG	comp77795_c0_seq2 2_1780	1546		0,58	Pentatricopeptide repeat-containing protein At5g04810, chloroplastic^b^
Nb_miR396c	TTCCACAGCTTTCTTGAACTG	comp77334_c1_seq28 1342_2196	1370	0	3,74	Growth-regulating factor 5
Nb_miR396	TTCCACAGCTTTCTTAAACTG	comp79406_c0_seq1 393_3506	1932	2	0,29	No annotation^b^
Nb_miR396	TTCCACAGCTTTCTTGAACTG	comp76253_c0_seq1 643_1557	767	0	0,19	Growth-regulating factor 9
Nb_miR396	TTCCACAGCTTTCTTGGACTG	comp69128_c0_seq2 114_806	521	2	0,19	DNA-directed RNA polymerase V subunit 5A-l ike^b^
Nb_miR397	TCATTGAGTGCAGCGTTGATG	comp73318_c2_seq1 87_1109	883	2	4,42	Glyceraldehyde-3-phosphate dehydrogenase, cytosolic^b^
Nb_miR397	TCATTGAGTGCAGCGTTGATG	comp75344_c1_seq2 127_1839	810	2	0,24	Laccase-7
Nb_miR397	TCATTGAGTGCAGCGTTGATG	comp77652_c0_seq1 1261_3177	176	2	0,29	Probable serine/threonine-protein kinase abkC^b^
Nb_miR398	TATGTTCTCAGGTCGCCCCTG	comp100607_c0_seq1 102_557	78	0	9,46	Umecyanin
Nb_miR408	TGCACTGCCTCTTCCCTGGCT	comp85708_c0_seq1 121_624	630	0	15,30	Uclacyanin-2
Nb_miR408	TGCACTGCCTCTTCCCTGGCT	comp79061_c1_seq3 184_1473	2346	0	12,38	Copper-transporting ATPase PAA2, chloroplastic
Other known miRNAs and their targets						
Nb_miR482	TTCCAATTCCACCCATTCCTA	comp75828_c0_seq3 249_1742	1609	2	1,07	No annotation^b^
Nb_miR827	TTAGATGAACATCAACAAACT	comp75426_c0_seq1 2_226	1982	2	1,36	UPF0496 protein 4
Nb_miR4376	TACGCAGGAGAGATGATACTG	comp79500_c0_seq4 305_3562	284	2	0,98	Calcium-transporting ATPase 8, plasma membrane-type
Nb_miR6020	AAATGTTCTTCGAGTATCTTC	comp70698_c0_seq1 752_1555	996	1	0,68	GPN-loop GTPase 3 homolog^b^
Nb_miR6020	AAATGTTCTTCGAGTATCTTC	comp74553_c0_seq5 265_702	596	0	1,47	No annotation^b^
Nb_miR6020	AAATGTTCTTCGAGTATCTTC	comp75715_c0_seq5 271_2055	1544	2	0,27	Probable methylenetetrahydrofolate reductase^b^
Nb_miR6149	TTGATACGCACCTGAATCGGG	comp80883_c0_seq1 2_310	14	2	0,27	Tubulin alpha chain^b^
Nb_miR6149	TTGATACGCACCTGAATCGGG	comp75221_c0_seq2 455_1678	1646	2	1,77	Serine/threonine-protein kinase HT1^b^
Nb_miR6151	TGAGTGTGAGGCGTTGGATTGA	comp84169_c0_seq1 105_1127	334	2	2,10	Probable carboxylesterase 8^b^
Nb_miR6157	TGGTAGACGTAGGATTTGAAA	comp66224_c0_seq2 136_780	259		0,68	Ras-related protein RABA2b^b^
Nb miR6161	TGCTGGACCGACATACTTTGT	comp74264_c0_seq1 147_1052	343	2	0,48	60S ribosomal protein L5^b^

^a^ The abundance for the degradome fragment (tag) indicating cleavage at that position/total number of fragments x 1,000,000

^b^ New target in N. benthamiana for conserved and other known miRNAs

Altogether we found 44 targets for conserved miRNAs, from which 23 were classed as category 0, six as category 1 and fifteen as category 2. For other known miRNAs we have found 11 targets, from which one was classed as category 0, two as category 1 and eight as category 2.

We identified targets for 18 conserved miRNA families out of 23. Some of the conserved miRNAs without any identified targets (miR390 and miR399) were expressed at a low level in all of the sRNA libraries (Fig. 2), which may explain the lack of detection of cleaved targets. However, both miR162 and miR403 were expressed at a considerable level in at least some of the tissues (Fig. 2) without mRNA targets identified in the degradome libraries. As an alternative explanation these miRNAs might be engaged in translational repression, however in different plant species cleaved target mRNAs were identified as their target which makes this explanation less plausible [13]. We have confirmed 29 conserved targets of conserved miRNAs already identified in different plants [13]. Many of the identified conserved targets are members of different families of transcription factors, such as squamosal promoter-binding (SBP), MYB, ARF, NAC, HD-ZIPIII, CCAAT-binding, AP2 and TCP. In addition to these targets we also identified 15 new targets in *N. benthamiana* for conserved miRNAs (Table 2). We found one target of the non-conserved miR4376 which was already identified in tomato [42]. We also identified nine new targets of seven non-conserved miRNAs (Table 2). Most of the non-conserved miRNA families for which no target had been identified were expressed at a very low level in all tissues (miR170, miR477, miR1446, miR6019, miR6025, miR6147), except miR479, miR1919, miR6153 and miR6155 (expressed at moderate level at least in one sample) (Table 2, Fig. 2).

### *N. benthamiana* specific miRNAs

**Fig. 5:**
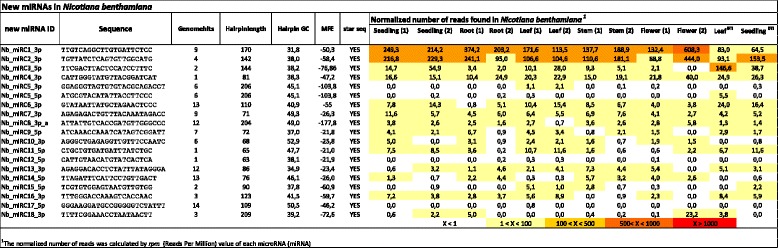
Novel miRNAs in *N. benthamiana*. Normalized read numbers of novel miRNAs across the tissues included in this study. Expression profiles are expressed in reads per million genome matching reads. Heat map colours represents absolute normalized levels of miRNA expression ranging from less than 1 RPM (white) to more than 1000 RPM (red) as indicated in the colour key

We used strict criteria to identify new miRNAs. Briefly, the sRNA reads were mapped to the *N. benthamiana* genome and secondary structures were predicted for each locus. Based on the hairpin prediction, the presence of miRNA* strands in the sRNA libraries and validated target mRNA in the degradome libraries we found 18 new miRNA candidates (Fig. 5, Additional file 5: Figure S3.). Nb_miRC1_3p was the most abundant *Nicotiana* specific miRNA in our sRNA libraries and it expressed in all of the tissues. The sequence of Nb_miRC1_3p was also reported previously from a large scale sRNA dataset as endogenous small RNA of *Nicotiana attenuata* [43], however it was not further investigated which small RNA class it belongs to. The second most abundant miRNA was the Nb_miRC2_3p and it was also present in all of the small RNA libraries. For the remaining *N. benthamiana* specific miRNAs the relative number of reads in all tissues was low (Fig. 5). However, this result is in line with the observation shown previously, that miRNA abundance decreases as the conservation of the sequence decreases [15].

**Fig. 6:**
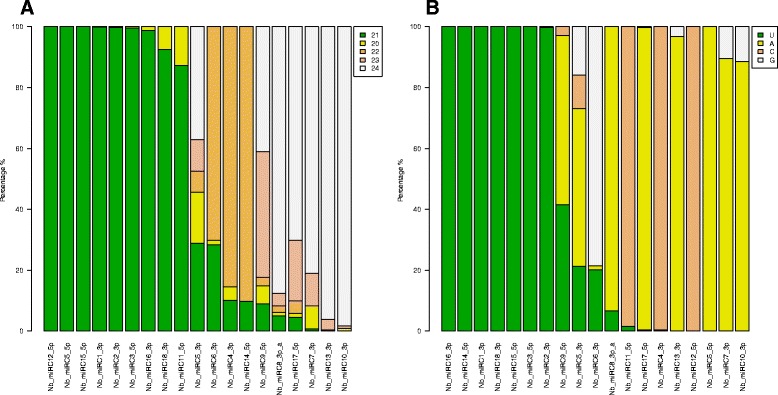
Size distribution and starting nucleotide of the novel *N. benthamiana* specific miRNAs. The relative abundance of different size categories (**a**), from 20 to 24 nucleotides is shown for the novel miRNAs presented in Fig. 5. The relative abundance of the 5′-nucleotide (**b**) is shown for the novel miRNAs presented in Fig. 5

As a next step, we checked the size distribution and starting nucleotide of the new *N. benthamiana* specific miRNAs in our libraries. The 21 nt long miRNAs were the most significant size class and half of the new miRNAs belong to this group (Fig. 6a). In several cases they were accompanied by additional size class, 20 nucleotide long sequences were present in significant proportions (Nb_miRC18_3p and Nb_miRC11_5p). Three miRNAs exhibited a strong preference for 22 nucleotide sequences (Nb_miRC6_3p, Nb_miRC4_3p and Nb_miRC14_5p) and in five miRNA families the 24 nucleotide long sequences were the most abundant size class (Nb_miRC8_3pa, Nb_miRC17_5p, Nb_miRC7_3p, Nb_miRC13_3p and Nb_miRC10_3p). We found that two miRNA families showed the coexistence of 20–24 nucleotide variants with the 24 nucleotides being the most abundant variants (Nb_miRC5_3p and Nb_miRC9_5p) (Fig. 6a).

Next we checked the 5′-nucleotide distribution of the new miRNA families in our libraries. Seven started with uracil at their 5′-end (Nb_miRC16_3p, Nb_miRC14_5p, Nb_miRC1_3p, Nb_miRC18_3p, Nb_miRC15_5p, Nb_miRC3_5p and Nb_miRC2_3p) (Fig. 6b). Eight miRNA families exhibited a preference for an adenine at position 1, although six of these have a different nucleotide composition at position 1 to a variable degree (NB_miRC9_5p, NB_miRC5_3p, NB_miRC8_3p_a, NB_miRC13_3p, NB_miRC7_3p and NB_miRC10_3p). In three miRNA families the most frequently found first nucleotide at their 5′-end was cytosine (Nb_miRC11_5p, Nb_miRC4_3p and Nb_miRC12_5p). One miRNA family exhibited a preference for a guanine at position 1, although a significant fraction has uracil at the 5′-position (Nb_miRC6_3p) (Fig. 6b). Although the majority of plant miRNAs have a uracil at position 1, it is not unusual that some miRNA families contain adenine, cytosine and even guanine in significant proportions at the 5′-position [15].

Young miRNAs are often weakly expressed, processed imprecisely or lack targets [16]. Our data fits well with these previous observations since most of the *N. benthamiana* specific miRNAs showed low relative abundance in our libraries (below 25 RPM; except Nb_miRC1_3p, Nb_miRC2_3p and Nb_miRC3_5p) and in several cases they were accompanied by additional size classes for various degrees. Furthermore, some of the new miRNAs identified in our sRNA libraries are produced from long hairpin precursors that may also indicate the young evolutionary origin (Nb_miRC2_3p, Nb_miRC3_5p, Nb_miRC5_3p, Nb_miRC8_3pa and Nb_miRC18_3p) (Additional file 5: Figure S3). This situation is well observable on the Nb_miRC8 precursor where two miRNA duplexes were both identified in the sRNA libraries and detected by Northern blots (Additional file 6: Figure S4). However, we did not identify miRNAs without target mRNAs in our data set, since we used the presence of cleaved target mRNA as a criterion to select new miRNAs.

**Fig. 7:**
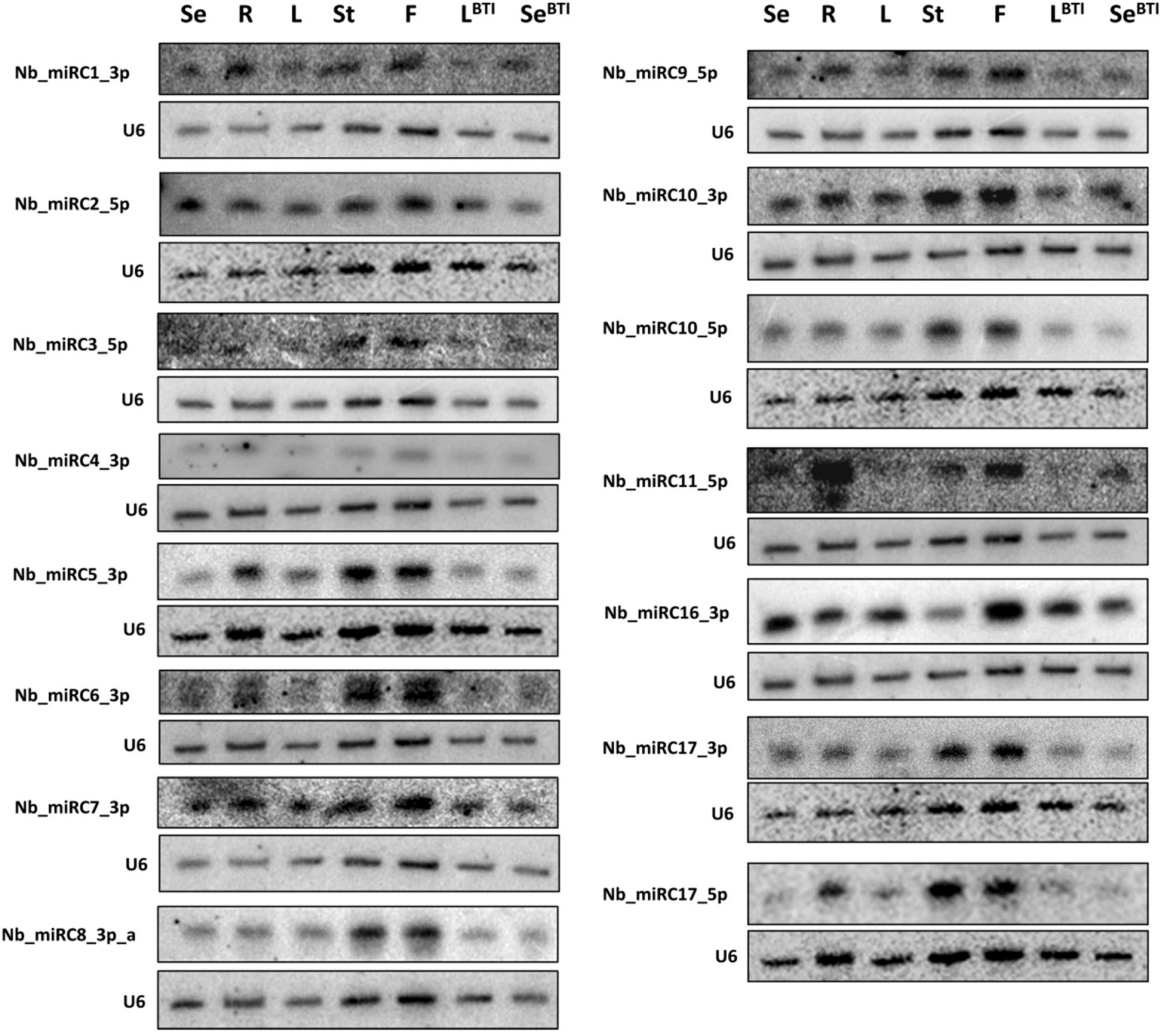
Expression patterns of novel miRNAs found in *N. benthamiana*. Total RNA was extracted from different tissues including, seedling (Se), root (R), leaf (L), stem (St), flower (F) from *N. benthamiana* plants used in our laboratory and from plants from Boyce Thompson Institute (leaf^BTI^ - L^BTI^, seedling^BTI^ - Se^BTI^). The RNA was separated on PAGE and transferred to nylon membranes for Northern blot analysis of the novel miRNAs. Oligonucleotide probes were used to detect specific miRNAs, and an U6-specific probe was used to detect U6 RNA as a loading control for each membrane

We used Northern blot analysis to check the expression level of *N. benthamiana* specific miRNAs in five tissues (seedling, root, leaf, stem and flower). The miRNAs that gave signal on the Northern blot, in most cases were detectable in all of the tissues tested (Fig. 7). Some of the new miRNAs were abundant in specific tissues (Nb_miRC10_3p in stem and flower; Nb_miRC16_3p in seedling and flower), and many of them showed differential expression in the tissues analysed (Nb_miRC1_3p, Nb_miRC2_5p, Nb_miRC10_3p, Nb_miRC11_5p and Nb_miRC16_3p) (Fig. 7). The relative number of reads in all tissues was very low for most of the miRNAs (Nb_miRC1_3p, Nb_miRC2_3p and Nb_miRC3_5p was an exception) and this was also reflected in the Northern blot analysis, since we did not obtain signal for six miRNAs (Nb_miRC5_5p, Nb_miRC12_5p, Nb_miRC13_3p, Nb_miRC14_5p, Nb_miRC15_5p and Nb_miRC18_3p). The expression profiles obtained by Northern blot analysis were different from the sequencing data for Nb_miRC3_5p, Nb_miRC6_3p, Nb_miRC8_3pa, Nb_miRC11_5p and Nb_miRC17_5p. However, for the other new miRNAs (Nb_miRC1_3p, Nb_miRC7_3p, Nb_miRC9_5p and Nb_miRC10_3p), sequencing data and expression profiles obtained by Northern blot analysis showed good correlation (Fig. 5 and Fig. 7). Although the relative read numbers for most of the new miRNAs were low, we were able to confirm the presence of 13 *N. benthamiana* specific miRNAs by Northern blot.

### Targets of *N. benthamiana* specific miRNAs

We identified 32 targets for new miRNAs using our degradome libraries. Interestingly, in the case of Nb_miRC5 we found target for both the mature and the star strand of the new miRNA (Table 3; Additional file 7: Figure S5; Additional file 8: Table S3). Based on the degradome data, among the identified target mRNAs of the specific, new *N. benthamiana* miRNAs, three were classed as category 0, two as category 1, twenty-two as category 2 and five as category 3. Comparing the target list of the known (conserved and non-conserved) and new miRNAs the most obvious difference between them is that the known miRNA targets mostly belong to category 0 and 1 (Tables 2 and 3). Based on their molecular function both groups regulate various genes that are included in transcriptional regulation or enzymatic activity (Additional file 9: Figure S6.).

**Table 3:** Targets of novel miRNAs found in *Nicotiana benthamiana*

new miRNA ID	Gene ID	Cleavage position	Category	Alignment score	Normalized abundance^a^	Annotation
Nb_miRC1_3p	comp79937_c1_seq1 312_1058	862	3	3,5	0,2	Elongation factor 1-alpha 4
Nb_miRC1_3p	comp78325_c0_seq3 335_2797	1128	1	1,0	0,2	Protein ROS1
Nb_miRC2_3p	comp75577_c0_seq17 1347_1982	405	2	4,0	1,2	Down syndrome critical region protein 3 homolog
Nb_miRC3_5p	comp79768_c0_seq4 287_3412	1868	2	3,5	1,1	Elongation factor Ts
Nb_miRC4_3p	comp76033_c0_seq2 367_1701	917	2	4,0	1,9	CBL-interacting serine/threonine-protein kinase 5
Nb_miRC5_3p	comp73317_c0_seq28 1673_2236	2967	0	3,5	2,1	Peroxisome biogenesis factor 10
Nb_miRC5_3p	comp69461_c0_seq1 371_982	1380	2	0,5	0,2	Protein CREG1
Nb_miRC5_3p	comp76292_c1_seq18 500_808	1632	2	1,5	0,2	Probable WRKY transcription factor 53
Nb_miRC5_3p	comp72235_c0_seq2 239_1444	1720	2	2,5	0,3	Cytochrome b561 and DOMON domain-containing protein At5g47530-like
Nb_miRC5_5p	comp70843_c0_seq1 178_1023	528	2	4,0	1,4	Protein TIC 21, chloroplastic
Nb_miRC6_3p	comp75362_c2_seq1 7645_8472	2399	2	3,5	0,2	G-type lectin S-receptor-like serine/threonine-protein kinase At1g11330
Nb_miRC7_3p	comp73495_c0_seq6 312_1019	1288	2	3,5	1,4	Proteasome subunit alpha type-2-A
Nb_miRC7_3p	comp56753_c0_seq1 215_457	517	0	2,0	5,5	WAT1-related protein At5g40240-like
Nb_miRC8_3p_a	comp71383_c0_seq6 99_857	1317	3	2,5	0,6	Aquaporin TIP1-3
Nb_miRC8_3p_a	comp75266_c2_seq4 3193_3486	3454	2	1,0	0,7	Probable isoaspartyl peptidase/L-asparaginase 3
Nb_miRC9_5p	comp71496_c0_seq1 489_1463	462	2	4,0	0,2	Serine/threonine-protein phosphatase 2A regulatory subunit B” subunit alpha
Nb_miRC9_5p	comp64986_c0_seq1 125_487	1232	2	2,5	0,2	60S ribosomal protein L34
Nb_miRC10_3p	comp77607_c3_seq4 3_1151	2317	2	4,0	0,2	Suberization-associated anionic peroxidase
Nb_miRC10_3p	comp80078_c3_seq4 193_2097	2131	2	1,0	1,1	Formate--tetrahydrofolate ligase
Nb_miRC10_3p	comp72702_c0_seq4 152_2431	2575	3	2,0	1,0	Beta-amyrin synthase
Nb_miRC11_5p	comp82078_c0_seq1 3_1277	938	2	3,5	2,7	Threonine deaminase
Nb_miRC11_5p	comp78978_c0_seq3 374_2785	2557	2	3,0	0,4	Transcription factor RF2a/ Probable transcription factor PosF21
Nb_miRC11_5p	comp89465_c0_seq1 218_1237	532	3	4,0	0,2	Ervatamin-B-like
Nb_miRC12_5p	comp79295_c0_seq1 283_3321	3114	2	3,5	2,7	Probably inactive leucine-rich repeat receptor-like protein kinase At3g28040
Nb_miRC12_5p	comp56752_c0_seq1	568	1	3,5	0,7	ZRT/IRT-like protein 1
Nb_miRC13_3p	comp77835_c0_seq3 61_621	797	2	2,5	0,3	Disease resistance response protein 206
Nb_miRC14_5p	comp74263_c0_seq1 230_2029	966	2	4,0	3,8	Chaperonin 60 subunit beta 2, chloroplastic
Nb_miRC15_5p	comp78088_c1_seq1 1536_2366	3251	3	4,0	0,7	Peroxisome biogenesis protein 19-1
Nb_miRC15_5p	comp85070_c0_seq1 3_2648	2151	2	2,5	1,0	(E,E)-geranyllinalool synthase
Nb_miRC16_3p	comp88815_c0_seq1 2_1909	1845	2	2,5	0,9	Polyphenol oxidase, chloroplastic
Nb_miRC17_5p	comp63365_c1_seq3 3_242	29	0	1,0	1,3	Heterotrimeric GTP binding protein alpha subunit
Nb_miRC18_3p	comp67145_c0_seq1 106_930	361	2	3,5	2,0	50S ribosomal protein L3, chloroplastic

^a^ The abundance for the degradome fragment (tag) indicating cleavage at that position/total number of fragments x 1,000,000

The new miRNAs target different genes with a wide variety of predicted functions. One of the three identified category 0 targets of the new miRNAs is the mRNA of the heterotrimeric GTP binding protein (G proteins) alpha subunit (targeted by Nb_miR17_5p). G proteins are highly conserved in eukaryotes. Plant G proteins play an important regulatory role in multiple physiological processes. They are involved in signal transduction from hormone receptors, including the plant hormones gibberellin and abscisic acid that regulate gene expression; secretion, defence, stomata movements, channel regulation, sugar sensing and cell death [44]. The heterotrimeric G protein complex is minimally composed of three subunits: alpha, beta and gamma. Plants have only a small number of genes encoding the G proteins subunits. *Arabidopsis* has only one canonical G protein alpha subunit, one beta subunit and three gamma subunit [45]. Plant alpha subunits are highly conserved between species, it binds and hydrolyse GTP and relay signals by interacting with downstream effector proteins [46]. Interestingly, during convergent evolution human G protein alpha-13 (GNA13) expression is also regulated by miRNA (miR-31) in breast cancer cells [47].

Another category 0 target is the mRNA of Peroxisome Biogenesis Factor 10 (PEX10). It is cleaved by Nb_miRC5_3p and has been demonstrated to be a component of the peroxisomal matrix protein import machinery. Peroxisomes are eukaryotic organelles and they are involved in a wide range of processes. Plant peroxisomes are important in primary and secondary metabolism, development, and responses to abiotic and biotic stresses. Previous studies identified 22 PEX genes in the *Arabidopsis* genome and demonstrated that they play a pivotal role in the import of peroxisomal matrix proteins [48]. PEX 10 perform multiple functions, including the biogenesis of ER-derived protein and oil bodies [49], the maintenance of ER morphology, the formation of cuticular wax [50], peroxisome and chloroplast connections [51].

One of the category 1 targets is REPRESSOR OF SILENCING (ROS1) which is cleaved by Nb_miRC1_3p. Interestingly, the sequence of Nb_miRC1_3p can also be found in *Nicotiana attenuata* [43] and its target site on ROS1 is conserved in other *Nicotiana* species as well. ROS1 encodes a nuclear DNA glycosylase domain protein that catalyse the release of 5-methylcytosine (5-meC) from DNA via a base excision repair reaction [52]. DNA cytosine methylation is an important epigenetic mark conserved in many eukaryotes. It has important roles in transposon silencing, genome organization, genomic imprinting and regulation of gene expression [53]. During continuous adaptation to variable external and internal environments DNA methylation patterns are dynamically controlled by methylation and demethylation reactions. ROS1 plays an important role in active DNA demethylation and helps to maintain gene expression [54, 55]. The targeting of ROS1 by a miRNA provides a new regulatory layer to reinforce transcriptional gene silencing by a post-transcriptional repression of its activity. The targeting of a DNA glycosylase (DEMETER-LIKE protein3) by a miRNA (miR402) was first predicted in *Arabidopsis* [56] and later it was shown that the expression of DEMETER-LIKE protein3 mRNA was down-regulated in miR402-overexpressing transgenic plants [57].

The other category 1 target is a zinc-regulated transporter (ZRT), iron-regulated transporter (IRT)-like protein 1 (ZRT/IRT-like protein 1) and is cleaved by NB-miRC12_5p. ZRT/IRT-like protein family has been demonstrated to be involved in metal uptake and transport. ZRT/IRT-like proteins generally contribute to metal ion homeostasis by transporting cations into the cytoplasm [58]. It has been reported in several plant species that various miRNAs are vital for maintaining nutrient homeostasis by regulating the expression of transporters that are involved in nutrient uptake and mobilization [59].

Other targets that are worthy to note are the WAT1-related protein At5g40240-like gene targeted by Nb_miRC7_3p; the CBL-interacting serine/threonine-protein kinase gene targeted by Nb_miRC4_3p, proteasome subunit alpha type-2-A gene targeted by Nb_miRC7_3p, suberization-associated anionic peroxidase targeted by Nb_miRC10_3p and beta-amyrin synthase targeted by Nb_miRC10_3p.

## Conclusions

In this study we have confirmed the expression of known and new miRNAs in various tissues of *N. benthamiana* using high-throughput sequencing and Northern blot analysis. As a result, this work represents a tissue specific atlas of known and new miRNA expression of *N. benthamiana*. In addition, we have experimentally identified many targets of both known and new miRNAs using a high-throughput method for the global identification of miRNAs targets. This study provides a valuable resource of miRNAs and their validated target mRNAs to the plant research community that will be beneficial well into the future.

## Methods

### Plant materials

Wild-type *Nicotiana benthamiana* plants and *Nicotiana benthamiana* plants from Boyce Thompson Institute were grown in soil or, for seedling and root samples, on half-strength Murashige and Skoog plates under standard growth conditions at 24 °C with 16 h of illumination per day. Total RNA was extracted from 14 day old seedlings and root tissues grown on plates. Furthermore total RNA was also extracted from leaves, stems and flowers without sepals collected from plants grown in soil. Total RNA was isolated by using the phenol/chloroform extraction method [60]. The quality and the quantity of the isolated total RNAs were checked using a nanodrop spectrophotometer and the integrity of RNAs was checked via non-denaturing agarose gel electrophoresis.

### RNA sequencing (RNA-Seq) library preparation for Solexa sequencing

cDNA library for RNA-Seq was generated from 1 μg total RNA using TruSeq RNA Sample Preparation Kit (Illumina, San Diego, CA, USA) according to the manufacturer’s protocol. Briefly, poly-A tailed RNAs were purified by oligodT conjugated magnetic beads and fragmented on 94 °C for 8 min, then 1st strand cDNA was transcribed using random primers and SuperScript II reverse transcriptase (Lifetechnologies, Carslbad, CA, USA). Following this step second strand cDNA were synthesized, then double stranded cDNA molecules were end repaired and 3′ ends adenylated then Illumina index adapters were ligated. After adapter ligation step enrichment PCR was performed to amplify the adapter-ligated cDNA fragments. Fragment size distribution and molarity of libraries were checked on Agilent BioAnalyzer DNA1000 chip (Agilent Technologies, Santa Clara, CA, USA). 10pM of denatured libraries were used for cluster generation on cBot instrument, and then sequencing run was performed on Illumina HiSeq 2000 instrument (100 bp paired-end) (Illumina, San Diego, CA, USA).

### Small RNA sequencing libraries for Solexa sequencing

The small RNA cDNA libraries were constructed using the Small RNA Sample Prep Kit (Illumina, CA, US), according to the manufacturer’s instructions. Briefly, 1 μg of total RNA from wild-type *Nicotiana benthamiana* samples, including, seedling, root, leaf, stem and flower were ligated to Solexa adaptors and then converted to cDNA by RT-PCR. The resulting cDNAs were then amplified by PCR, gel-purified and submitted for Illumina/Solexa sequencing. High-throughput sequencing was performed using HiScan SQ platform (50 bp single-end).

### Degradome cDNA libraries for Solexa sequencing

The degradome cDNA library was prepared using a total RNA from wild-type *Nicotiana benthamiana* samples, including, seedling, root, leaf, stem and flower following the procedures previously described by German et al. [39]. Conversely to the small RNA libraries, the degradome cDNA library was sequenced with a custom-made sequencing primer (5′-CCACCGACAGGTTCAGAGTTCTACAGT-3′) [36] on HiScan SQ platform (50 bp single-end).

### Northern blot analysis

A total amount of 5 μg of RNA from each *Nicotiana benthamiana* tissues (seedling, root, leaf, stem, flower, leaf BTI, seedling BTI) were individually separated on a 8 M UREA/12 % polyacrylamid 1 x TBE gel. After gel run we transferred the RNAs from dPAGE onto the neutral nylon membrane, Hybond NX (Amersham/GE Healthcare) by semi-dry electroblotter. The transferred molecules were cross-linked by the enhanced sensitivity 1-ethyl-3-(3-dimethylaminopropyl) carbodiimide (EDC) chemical cross-linking method at 60 °C for 90 min as described previously by Pall et al. [61]. The membranes were prehybridized at 38 °C for 1 h in ULTRAhyb® Ultrasensitive Hybridization Buffer (Ambion/thermo Scientific). DNA oligo probes were end labelled by the forward reaction using T4 polynucleotide kinase (Thermo Scientific) and [γ^32^P] ATP for 1 h at 37 °C. Hybridization was performed at 40 °C overnight. Each membrane was stripped than rehybridized with different probes (The sequences of the used probes are available in Additional file 10: Table S4.). The membranes were exposed to a phosphorimager.

### Real-time PCR quantification of miRNAs

We also used real-time looped RT-qPCR procedure for detection and quantification of miRNAs. Stem-loop RT primers and forward qPCR primers were designed according to Chen et al*.* [62]. (The sequence data is aviable in Additional file 10: Table S4). As internal reference gene, nucleolar small RNA U6 was used [63] . We followed the protocol by Varkonyi-Gasic et al*.* [64] with optimalisation to our samples. For DNase treatment of the RNA samples 3 units of DNase I (*Thermo Scientific*) were used to 250 ng total RNA from the different tissues represented in this study. After 30 min incubation at 37 °C, a phenol/chloroform extraction was performed to inactivate the enzyme and purificate the template. For the elution 20 μl MQ water was used. In the next step, a multiplexed cDNA synthesis of U6 and miRNA was performed. Stem-loop pulsed reverse transcription protocol was performed in a 20 μl reaction volume with each sample for RT and NO RT reactions (0.5 μl (10 mM) dNTP’s, 1–1 μl (1 μM) U6 reference and miRNA specific primer and MQ water was mixed and heated to 65 °C for 5 min and incubate on ice for 2 min. Then 4 units of RNaseOUT (*Invitrogen*), 50 units of RevertAid H- Reverse transcriptase, 4 μl 5x RT Buffer (*Thermo Scientific*) and 1 μl RNA template from the previous DNase treatment the was added to the reaction tubes.). The pulsed RT was preformed in thermo cycler: 30 min at 16 °C incubation, followed by pulsed RT of 60 cycles at 30 °C for 30 s, 42 °C for 30 s and 50 °C for 1 s, then at 85 °C for 5 min to inactivate the enzymes. To test the primer specificity and cDNA quality end-point PCR was performed with Pfu polymerase (*Thermo Scientific*) by 40 cycles following the Várkonyi-Galic et al. protocol. To perform real-time PCR the following components were used: 5 μl 2x FastStart Essential DNA Green Master Mix (*Roche*), 0,5 μl (10 μM) forward primer, 0,5 μl (10 μM) Universal reverse primer, 3 μl water and 1 μl RT product. PCR tubes were incubated at 95 °C for 10 min, followed by a 2 Step amplicifation by 40 cycles of 95 °C for 15 s, 60 °C for 60 s. For melting curve analysis, the samples were denaturated at 95 °C for 10 s, 65 °C for 60 s, 97 °C 1 s, then cool to 37 °C in a Roche LightCycles 96 instrument. To analyse results the LightCycler software was used.

### Small RNA processing

Raw sequence reads quality check was performed using FastQC (version 0.11.3). For adaptor trimming cutadapt (version 1.2.1) was used with the following parameters: minimal sequence length after adaptor removal was 16, maximal sequence length was 28 [65]. We used UEA Small RNA Workbench (version 2.5.0) for all miRNA processing tasks [66]. MiRProf calculated the expression of known miRNAs. This program uses Mirbase (version 20). We allowed 2 mismatches during the analysis. We grouped together mismatches, variants and miRNAs from different organisms and worked only with families. MirCat identified candidant microRNAs. We used the default plant specific parameters. PAREsnip was used to process degradome sequences for finding microRNA targets [40] (Additional file 11). During this analysis, we discarded results from category 4, the maximal mismatch number was 4, mismatches at position 10–11 were not allowed and more than two mismatches at adjacent positions also not allowed. Every other parameter was the default. We have created a non-redundant scaffold set from the available *N. benthamiana* genome sequencing projects using Blast. We used this set as genome in all programs where genome sequence was a mandatory requirement. Results from these programs were analysed further with custom made Perl and Python scripts. For statistical analysis and visualization R was used [67].

### Transcript processing and annotation

To generate the corresponding mRNA transcriptome we have sequenced Illumina paired end TruSeq libraries from the same *N. benthamiana* leaf, stem, germ, root, flower and seed samples as at the small RNAs. We pooled together 315 million 2x100 bp reads and used Trinity *de novo* assembly program (r2013-02-16) with default parameters [68].

To find biological function for de-novo transcripts, Trinotate was used (version: 20130225) according to the manual [68]. All related data were pushed into an SQLight database for further processing.

To perform the Gene Ontology (GO) analysis of miRNA targets the agriGO web-based tool and database version 1.2 was used [69]. The analysis setting was the following: Singular Enrichment Analysis (SEA) with the *Arabidopsis thaliana* TAIR9 reference gene model. The graphical results showing the molecular functions.

### Data access

The RNA-seq, sRNA and degradome data discussed in this publication have been deposited in National Center for Biotechnology Informations (NCBI) BioProject database under Accession number: PRJNA288746.

## Additional files

Additional file 1: Table S1. MFEI values of conserved and other known miRNAs in *Nicotiana benthamiana.* (XLSX 18 kb) Additional file 2: Figure S1. Quantification of miRNAs by RT-qPCR. Validation of (A) Nb-miRNA167 and (B) Nb-miR482 expression by RT-qPCR. Northern blot validation of conserved miRNAs (Fig. 4.) compared to RT-qPCR quantification. In both experiments the reference gene was U6. (PDF 134 kb) Additional file 3: Figure S2. Target plots (t-plots) of conserved and other known miRNA targets confirmed by degradome sequencing. In the head of the pictures there is a unique identifier of the mRNA followed by the annotation of the transcript. The solid lines and dot in miRNA: mRNA alignments indicate matched RNA base pairs and GU mismatch, respectively. The relative abundances are plotted against the nucleotide position within the transcript. (PDF 377 kb) Additional file 4: Table S2. Targets of conserved and other known miRNAs in samples. (XLSX 44 kb) Additional file 5: Figure S3. Secondary structures of knew *N. benthamiana* specific miRNAs found in our liraries. The 5′ end of the RNA is marked by a circle. Candidant miRNAs are highlighted with different colours. Image was generated by RNAfold/RNAplot on The University of East Anglia sRNA toolkit (plant version). (PDF 219 kb) Additional file 6: Figure S4. Nb_miRC8 precursor. A) Secondary structure for Nb_miRC8. The 5′ end of the RNA is marked by a circle. The candidant miRNAs labelled with different colours. B) Northen blot expressions for the two Nb_miRC8 candidants (Nb_miRC8_3p_a, Nb_miRC8_3p_b). An U6-specific probe was used to detect U6 RNA as a loading control for each membrane. (PDF 398 kb) Additional file 7: Figure S5. Target plots (t-plots) of *N. benthamiana* specific knew miRNA targets confirmed by degradome sequencing. In the head of the pictures there is a unique identifier of the mRNA followed by the annotation of the transcript. The solid lines and dot in miRNA: mRNA alignments indicate matched RNA base pairs and GU mismatch, respectively. The relative abundances are plotted against the nucleotide position within the transcript. (PDF 364 kb) Additional file 8: Table S3. Targets of novel Nicotiana benthamiana specific miRNAs in samples. (XLSX 25 kb) Additional file 9: Figure S6. Gene Ontology (GO) analysis of miRNA target mRNAs. Functional annotation of known and *N. benthamiana* specific miRNA target mRNAs based on their molecular functions. (PDF 140 kb) Additional file 10: Table S4. Oligonucleotide sequences used for probes in Northern blot analysis and RT-PCR validation. (XLSX 11 kb) Additional file 11:
**This file lists the complete mRNA sequences of miRNA targets found in our samples in fasta formats.** (PDF 416 kb)
