# Supplementary figures and images for: Identification of Nicotiana benthamiana microRNAs and their targets using high throughput sequencing and degradome analysis
Source: BMC Genomics. 2015 Dec 1;16:1025. doi: 10.1186/s12864-015-2209-6 (PMC4667520; doi:10.1186/s12864-015-2209-6)

Additional Figure 1.

**A**

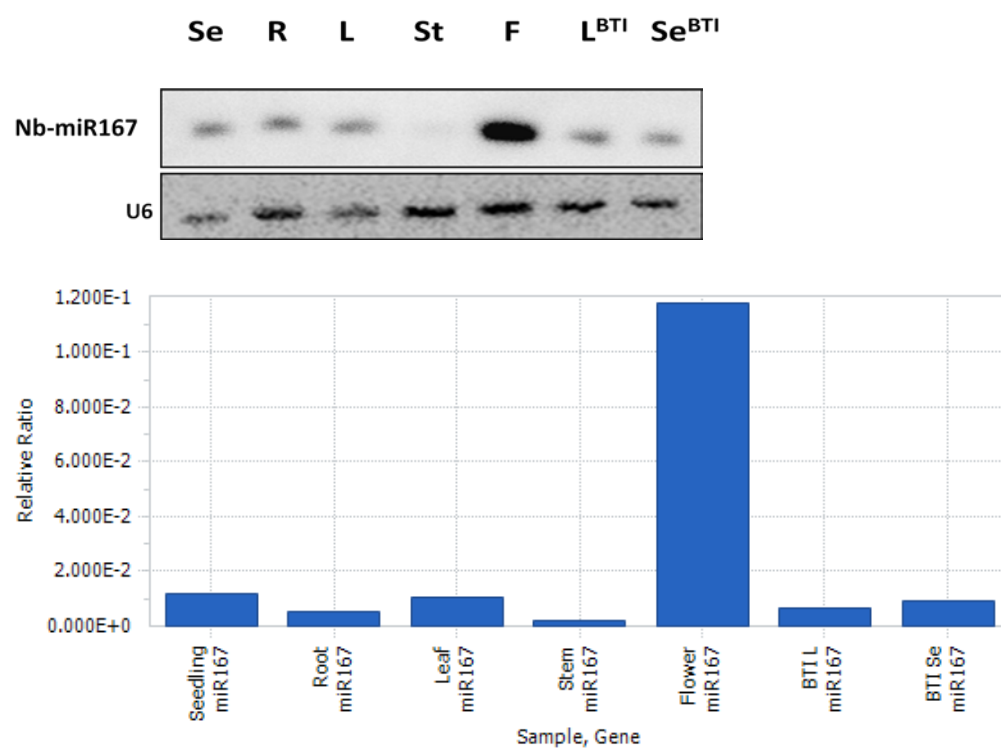

**B**

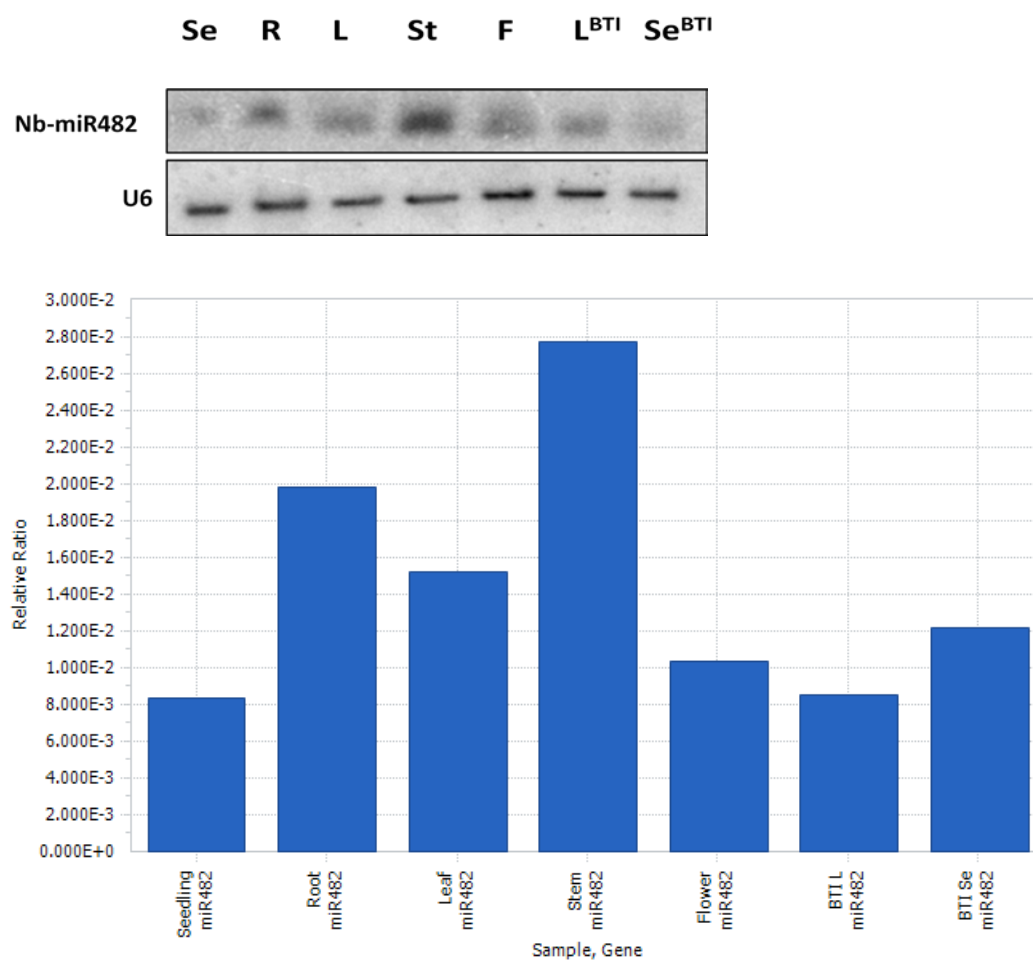

Supplement: Additional file 2: Figure S1. — Quantification of miRNAs by RT-qPCR. Validation of (A) Nb-miRNA167 and (B) Nb-miR482 expression by RT-qPCR. Northern blot validation of conserved miRNAs (Fig. 4.) compared to RT-qPCR quantification. In both experiments the reference gene was U6. (PDF 134 kb) [file 12864_2015_2209_MOESM2_ESM.pdf]

Additional Figure 4.

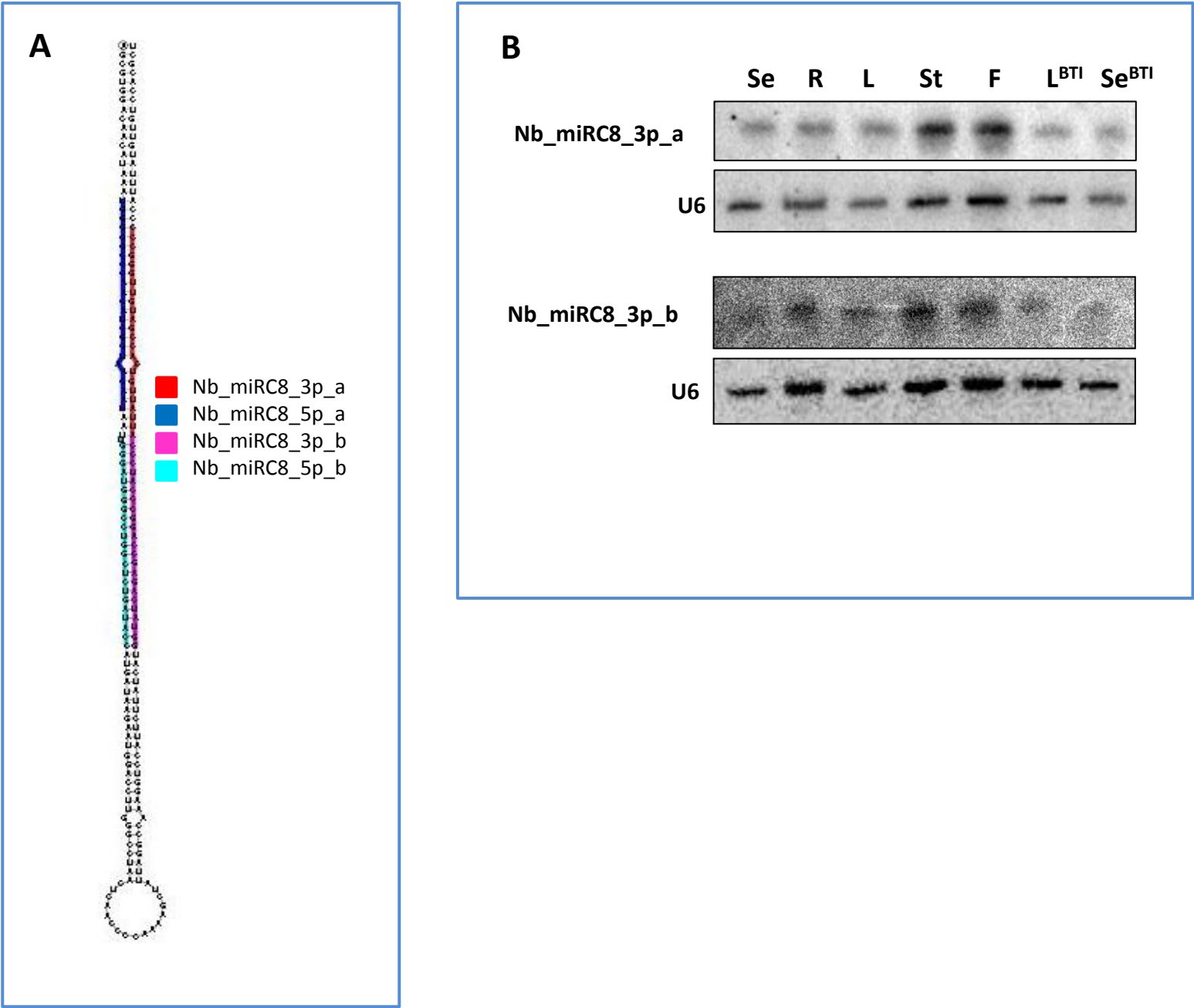

Supplement: Additional file 6: Figure S4. — Nb_miRC8 precursor. A) Secondary structure for Nb_miRC8. The 5′ end of the RNA is marked by a circle. The candidant miRNAs labelled with different colours. B) Northen blot expressions for the two Nb_miRC8 candidants (Nb_miRC8_3p_a, Nb_miRC8_3p_b). An U6-specific probe was used to detect U6 RNA as a loading control for each membrane. (PDF 398 kb) [file 12864_2015_2209_MOESM6_ESM.pdf]

Additional Figure 6.

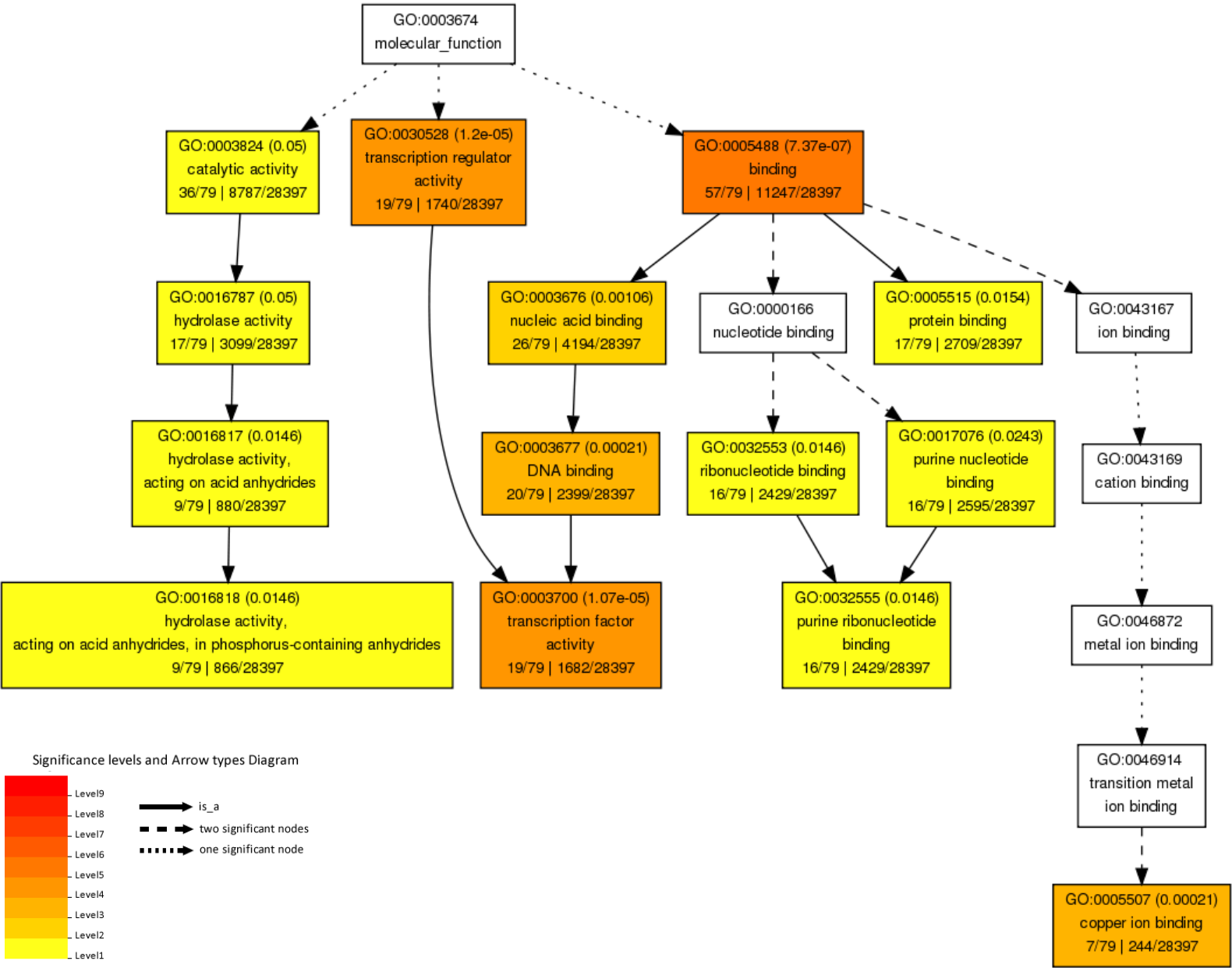

Supplement: Additional file 9: Figure S6. — Gene Ontology (GO) analysis of miRNA target mRNAs. Functional annotation of known and N. benthamiana specific miRNA target mRNAs based on their molecular functions. (PDF 140 kb) [file 12864_2015_2209_MOESM9_ESM.pdf]
