# Supplementary material for: Identification of Nicotiana benthamiana microRNAs and their targets using high throughput sequencing and degradome analysis
Source: BMC Genomics. 2015 Dec 1;16:1025. doi: 10.1186/s12864-015-2209-6 (PMC4667520; doi:10.1186/s12864-015-2209-6)

Additional Figure 2.

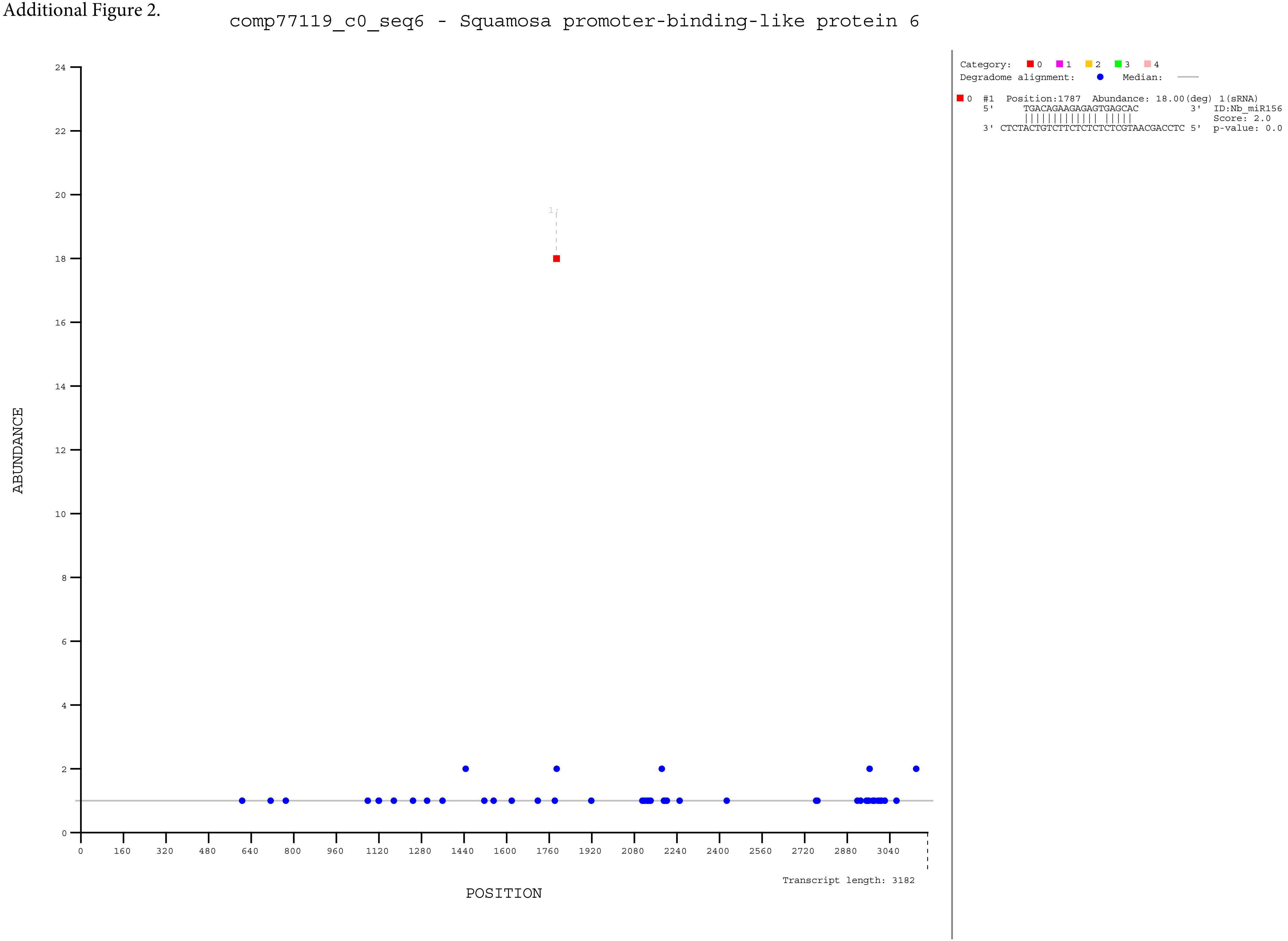

comp69399_c0_seq4 - Squamosa promoter-binding-like protein 16

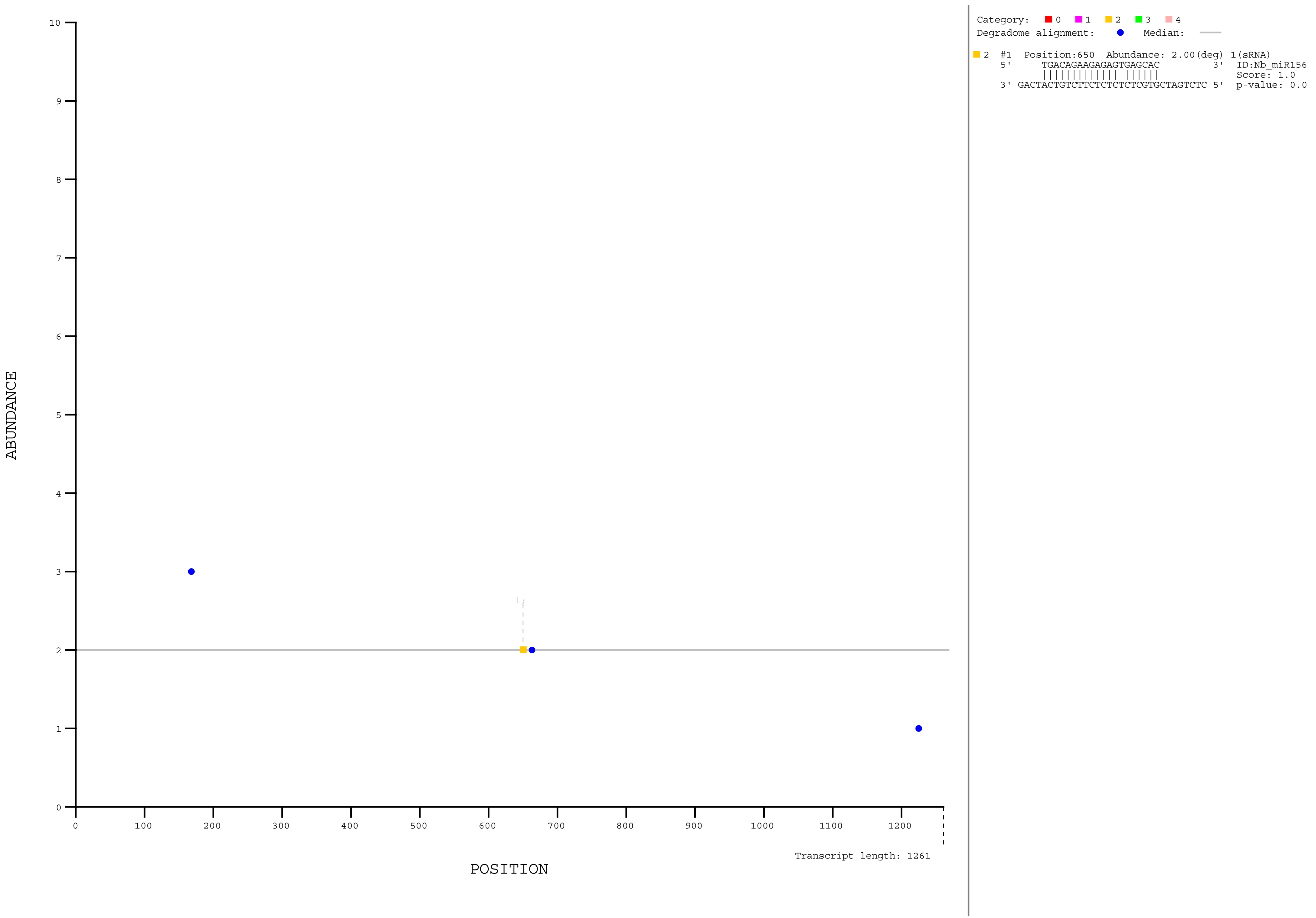

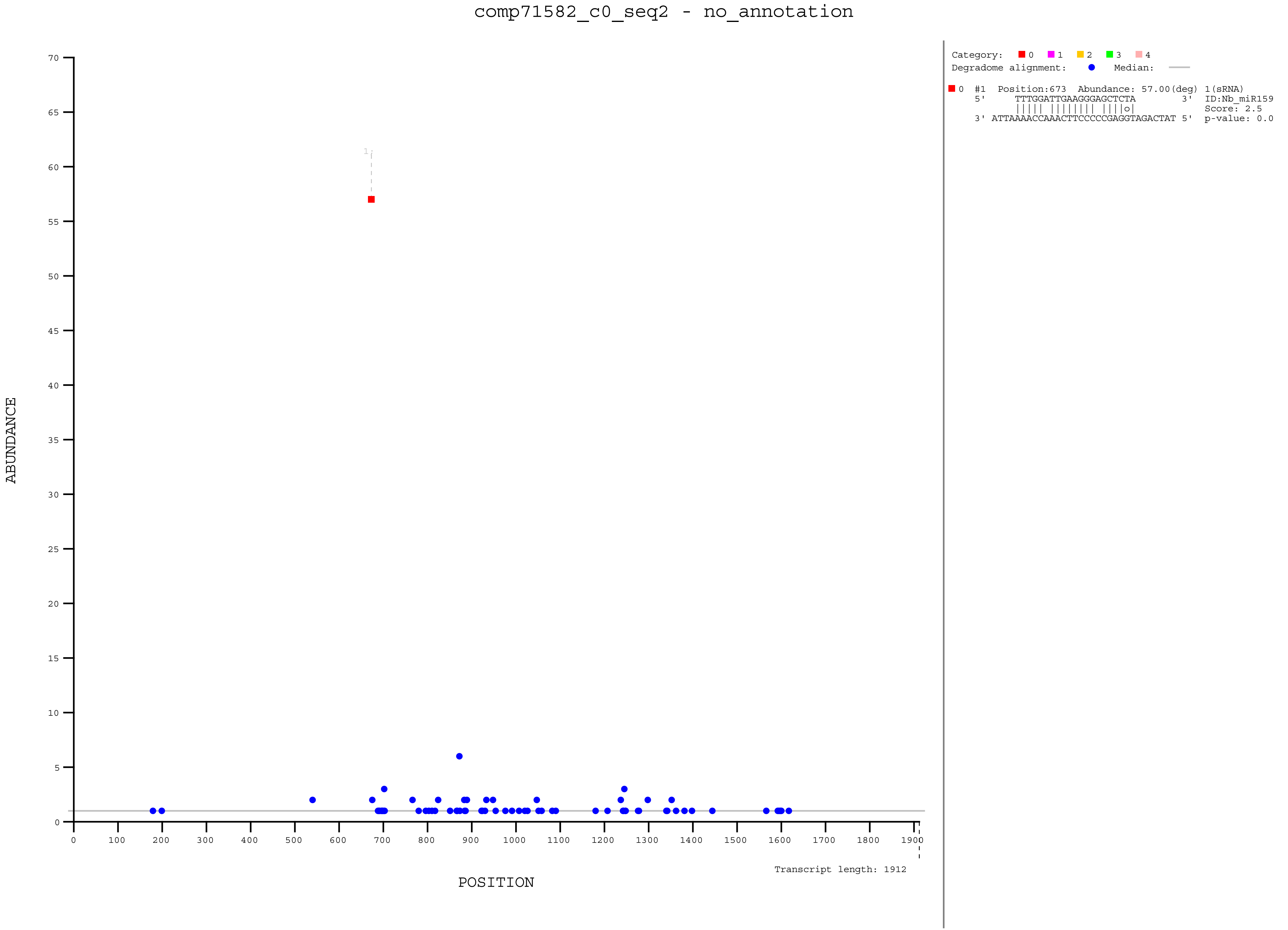
comp71582_c0_seq2 - no_annotation
Category: 0 1 2 3 4
Degradome alignment:
Median:
0 #1 Position:673 Abundance: 57.00(deg) 1(sRNA)
5' TTTGGATTGAAGGGAGCTCTA 3' ID:Nb_miR159
||||| |||||||| ||||o| Score: 2.5
3' ATTAAAACCAAACTTCCCCCGAGGTAGACTAT 5' p-value: 0.0
ABUNDANCE
POSITION
Transcript length: 1912

comp69815_c0_seq4 - Transcription factor GAMYB

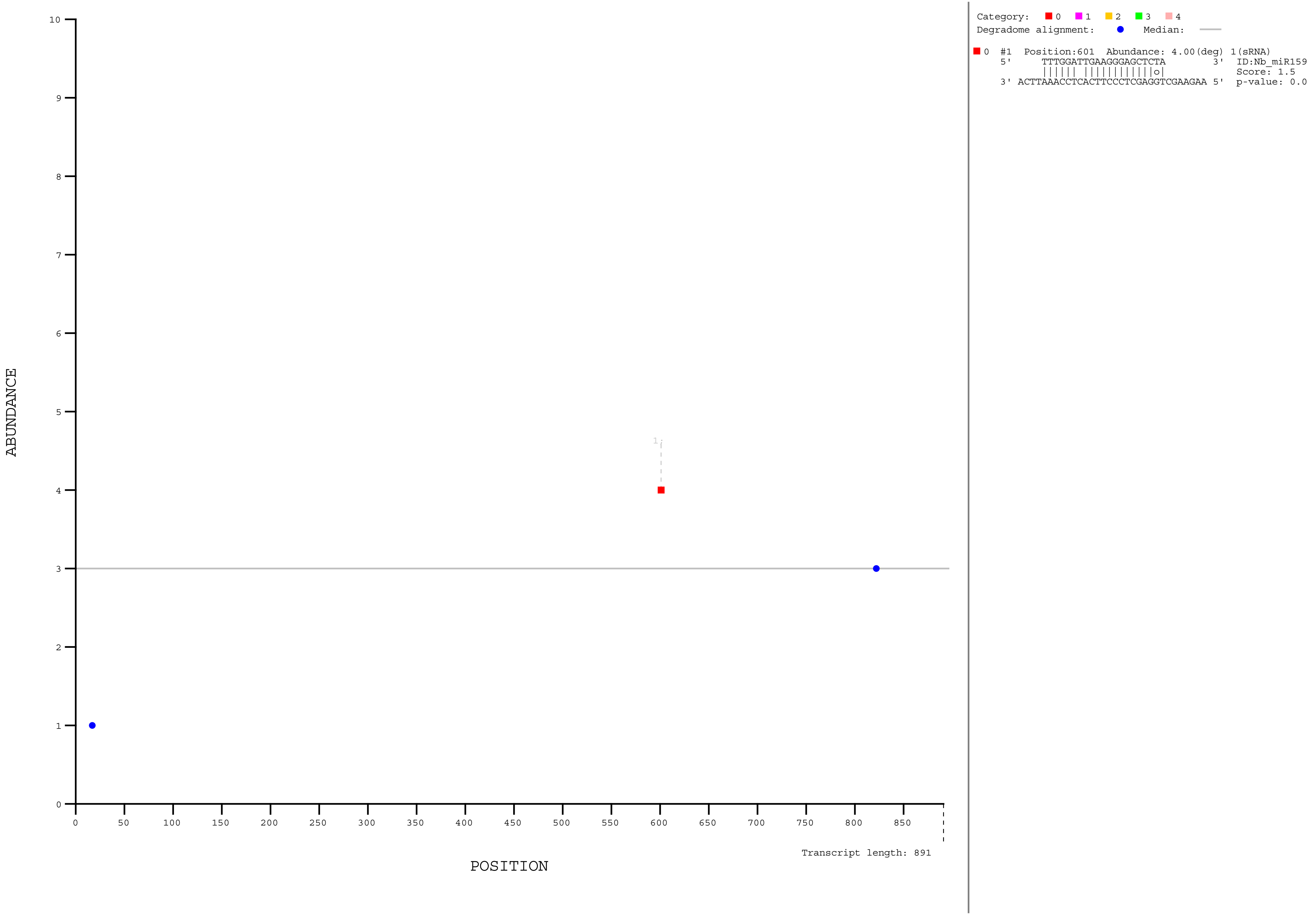

comp73942_c0_seq1 - Transcription factor GAMYB

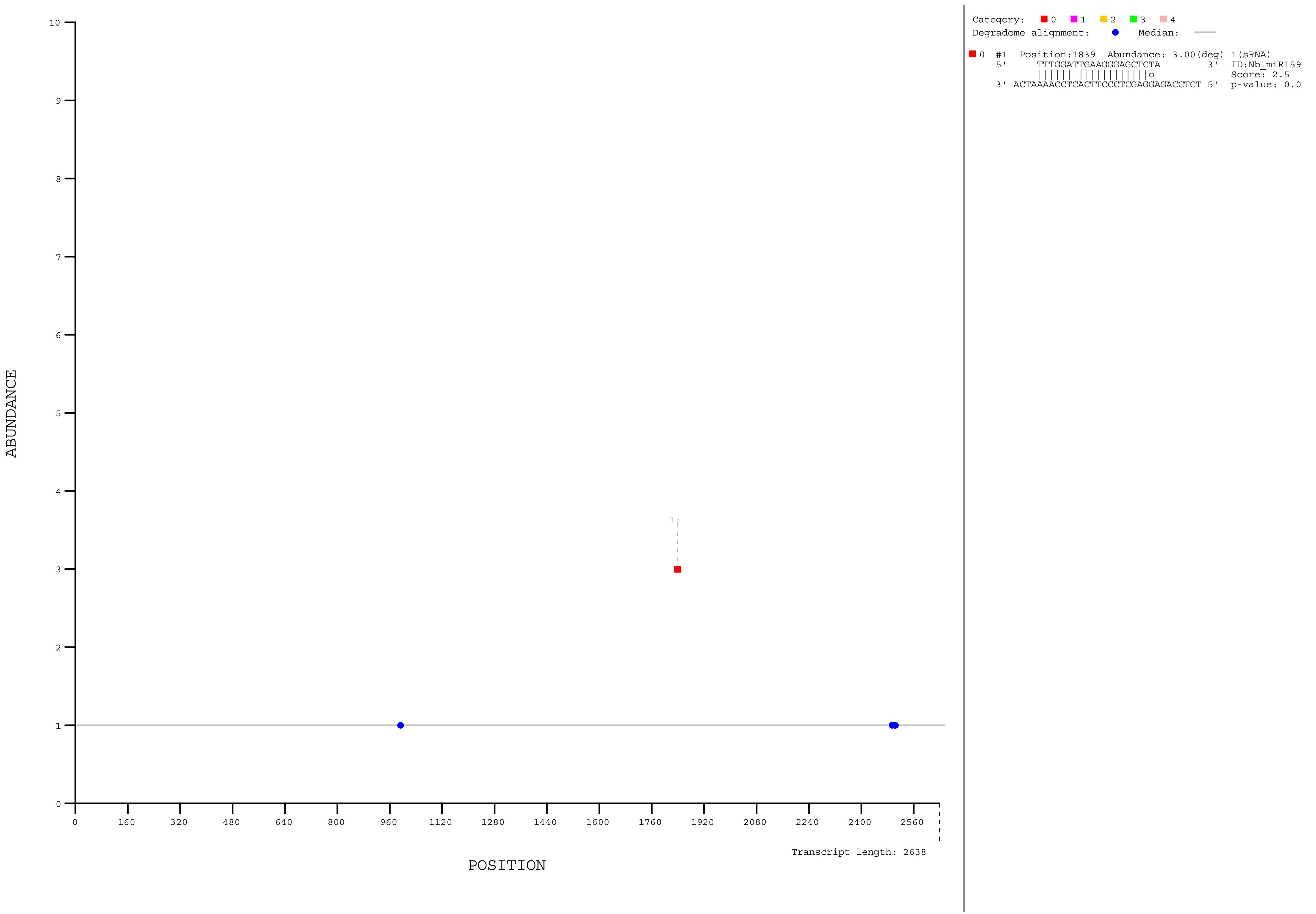

comp78286_c0_seq3 - Auxin response factor 18

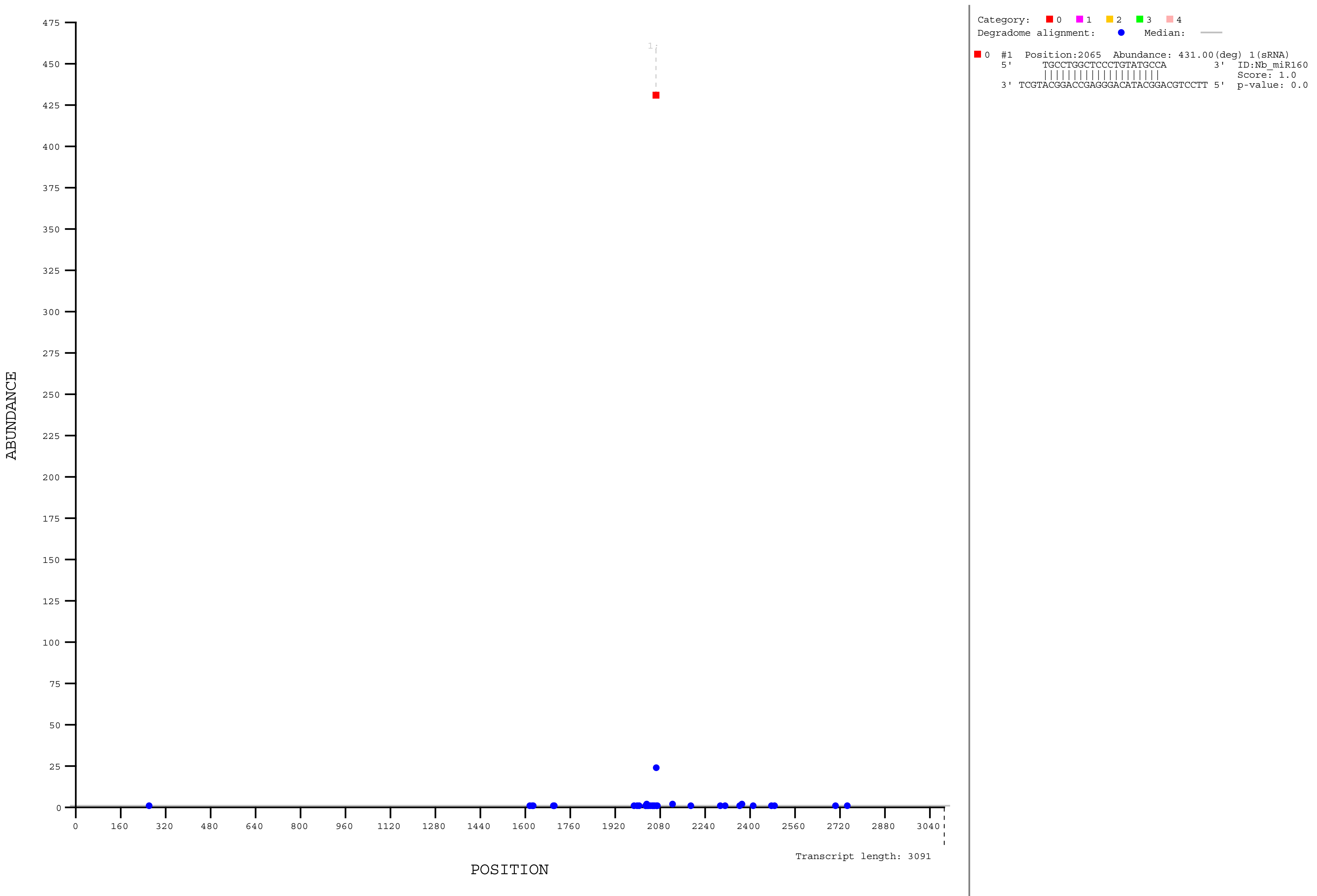


```
5'     TGCCTGGCTCCCTGTATGCCA        3'  ID:Nb_miR160
        ||||||||||||||||||||            Score: 1.0
3' TCGTACGGACCGAGGGACATACGGACGTCCTT 5'  p-value: 0.0
```

comp79462_c0_seq1 - Auxin response factor 16

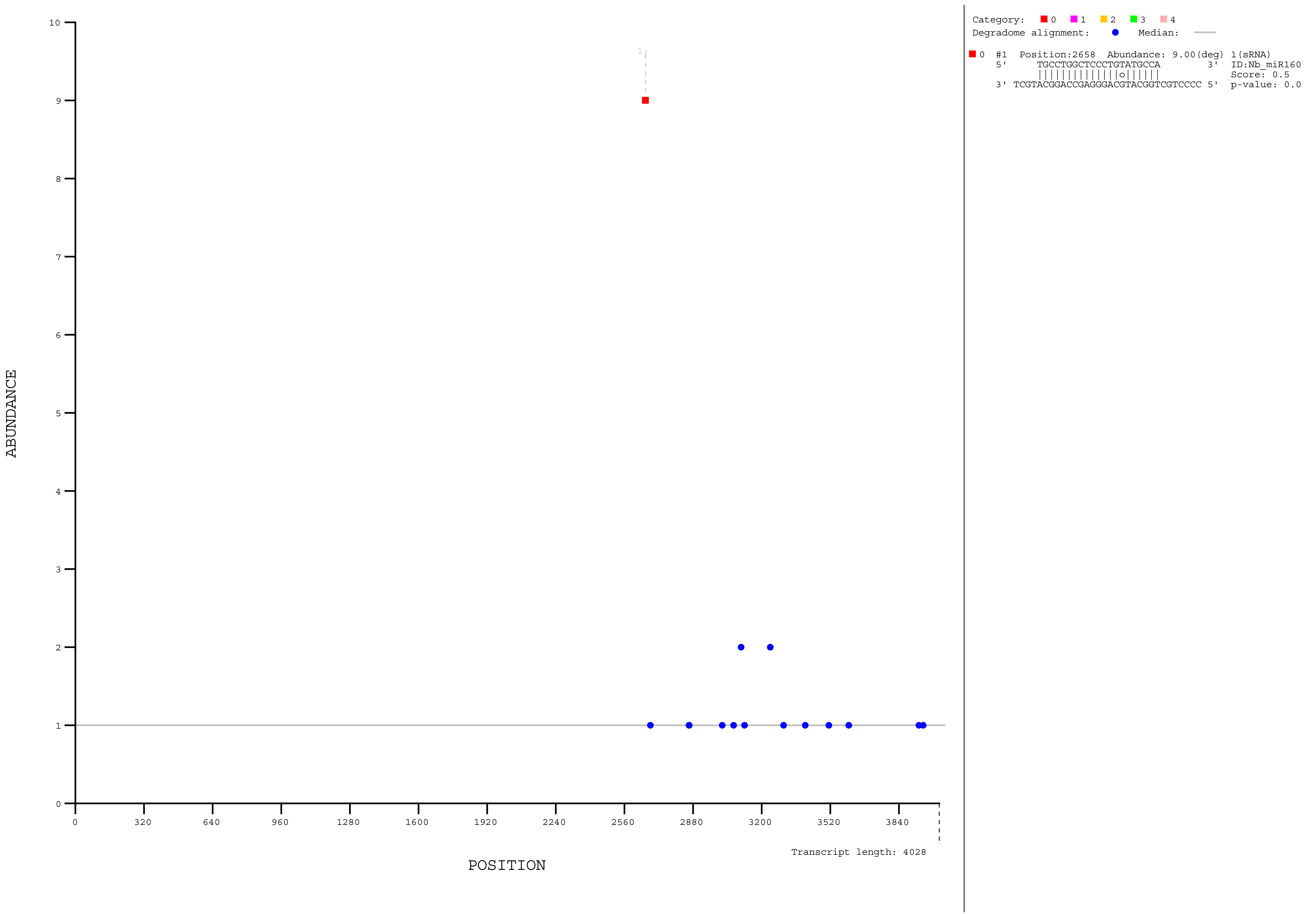

comp72325_c0_seq2 - Protein CUP-SHAPED COTYLEDON 2

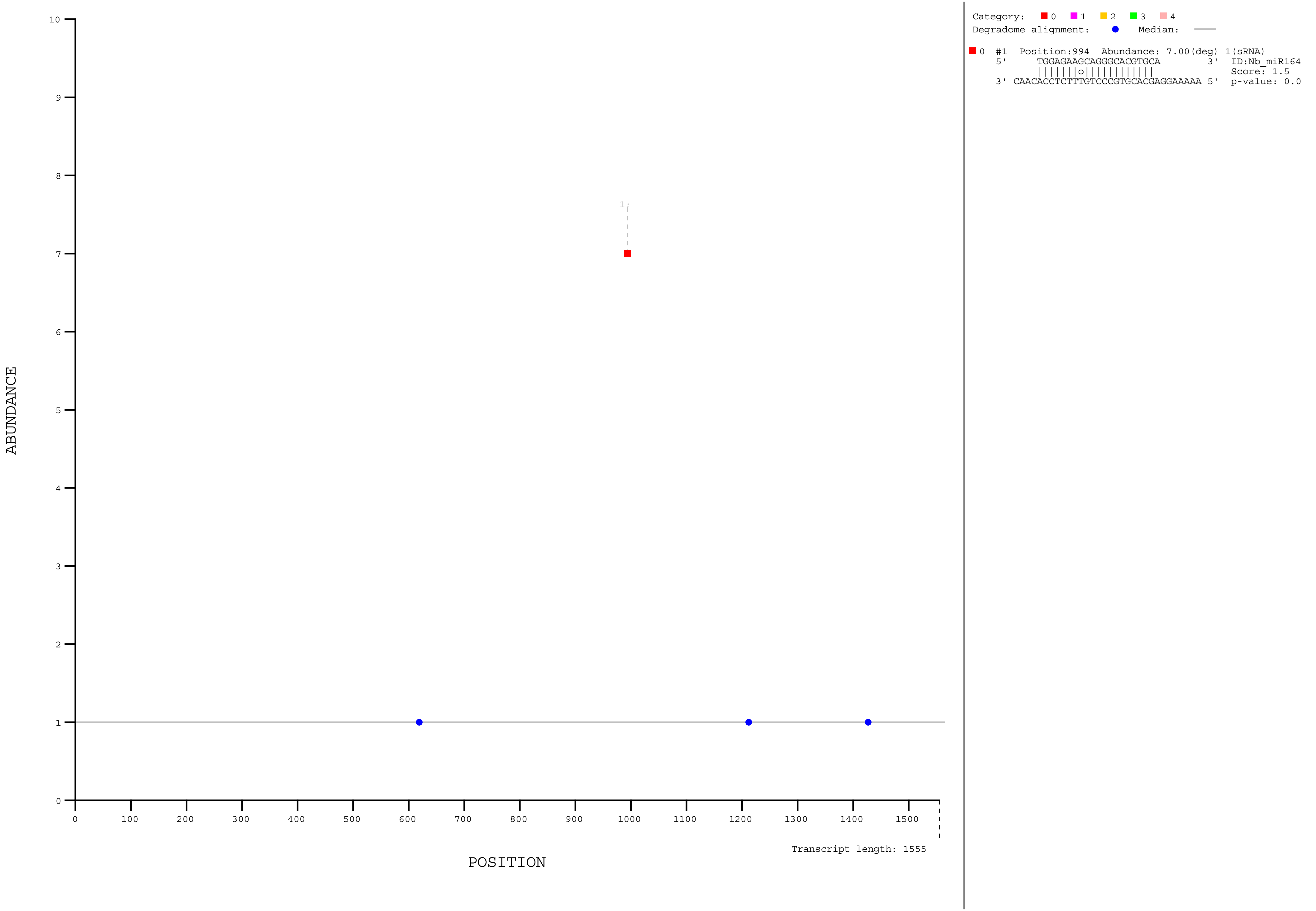

comp80060_c0_seq5 - Homeobox-leucine zipper protein REVOLUTA

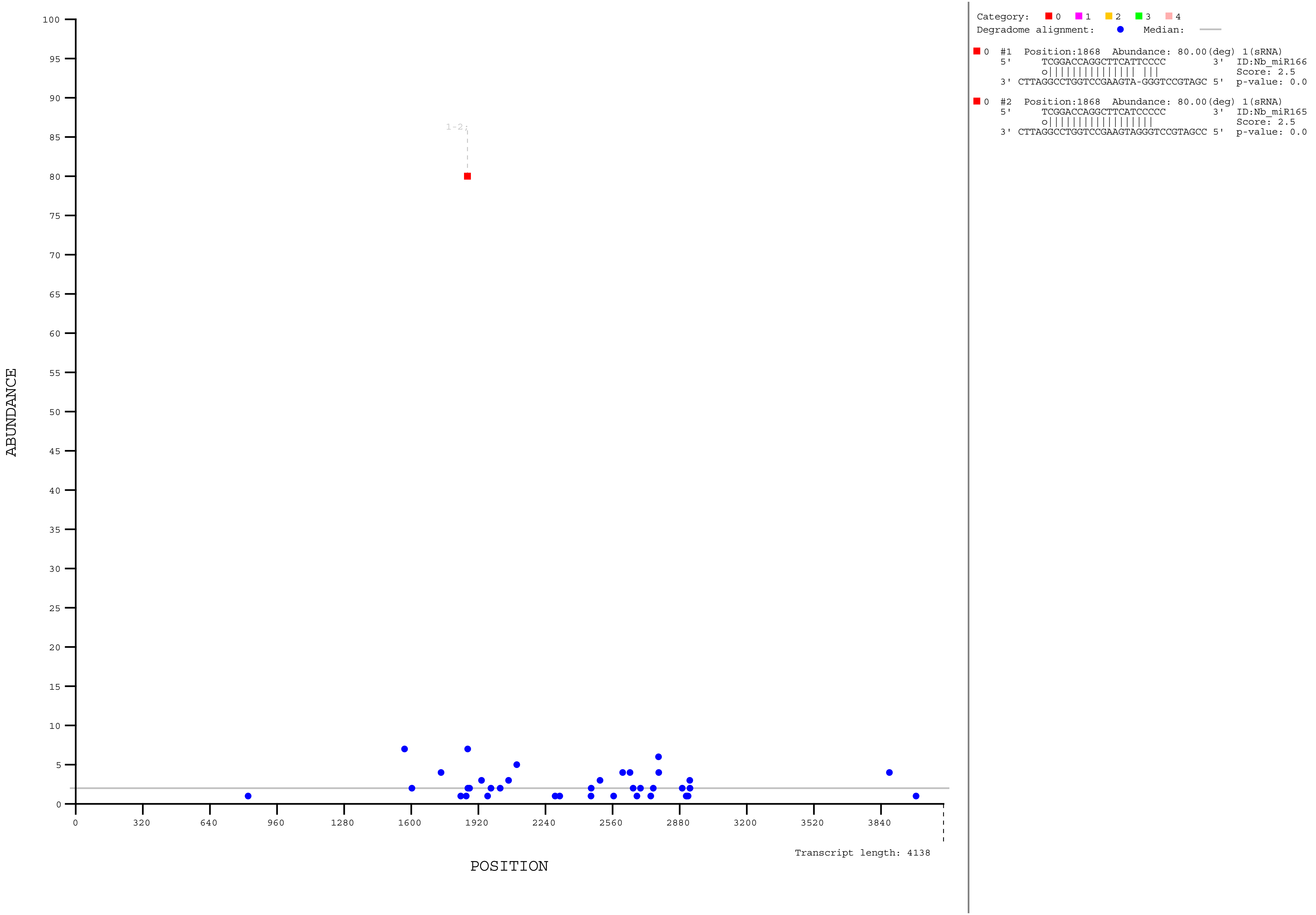

comp79517_c0_seq6 - Homeobox-leucine zipper protein ATHB-15

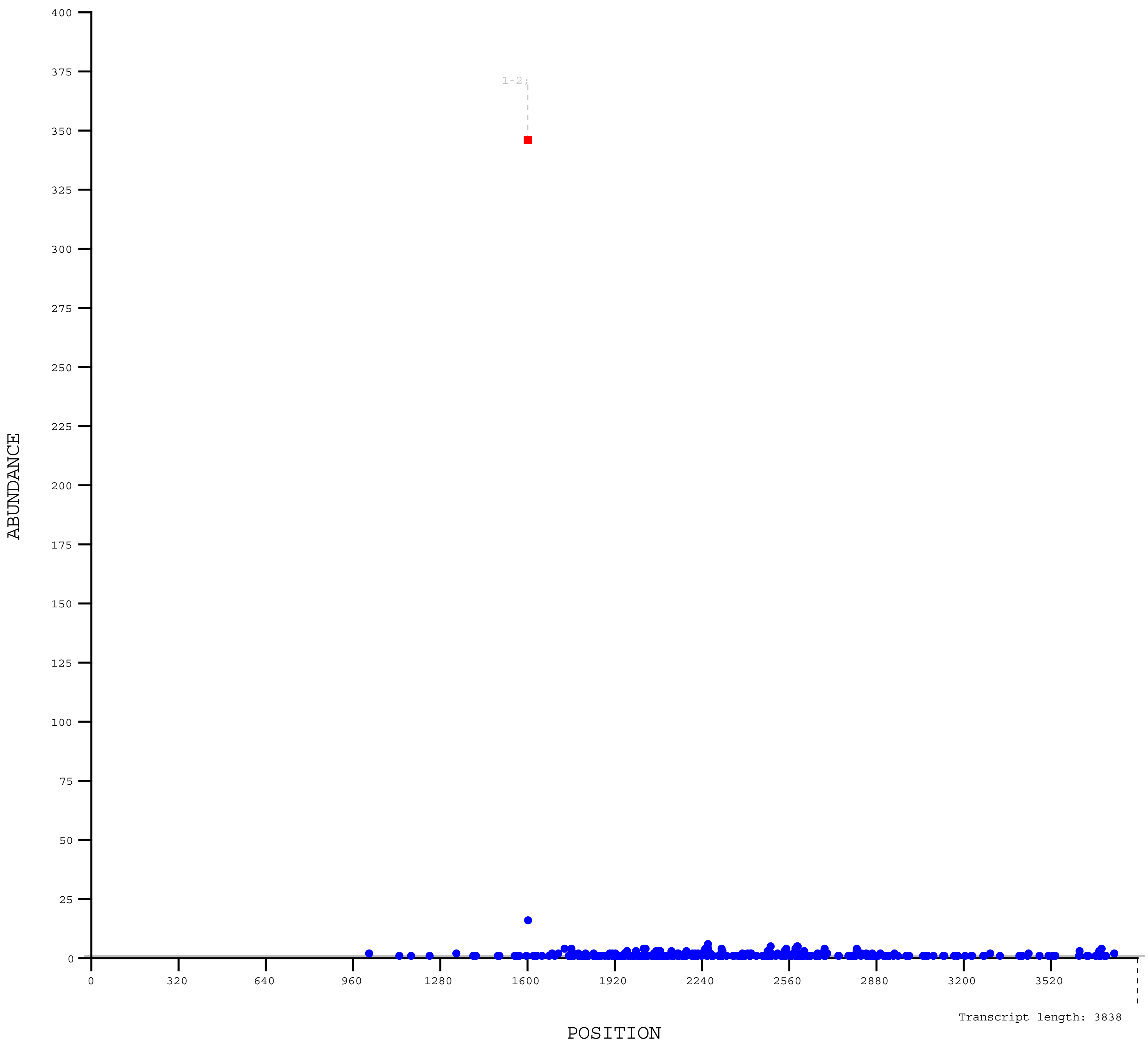


```
Category:  0  1  2  3  4
Degradome alignment:        Median:

0  #1  Position:1601  Abundance: 346.00(deg) 1(sRNA)
   5'     TCGGACCAGGCTTCATTCCCC        3'  ID:Nb_miR166
          o||||||||||||||||||              Score: 2.5
   3' CTTAGGCCTGGTCCGAAGTAAGGTCCGTAAAC 5'  p-value: 0.0

0  #2  Position:1601  Abundance: 346.00(deg) 1(sRNA)
   5'     TCGGACCAGGCTTCATCCCCC        3'  ID:Nb_miR165
          o||||||||||||||| ||              Score: 3.5
   3' CTTAGGCCTGGTCCGAAGTAAGGTCCGTAAAC 5'  p-value: 0.0
```

comp79347_c3_seq8 - no_annotation

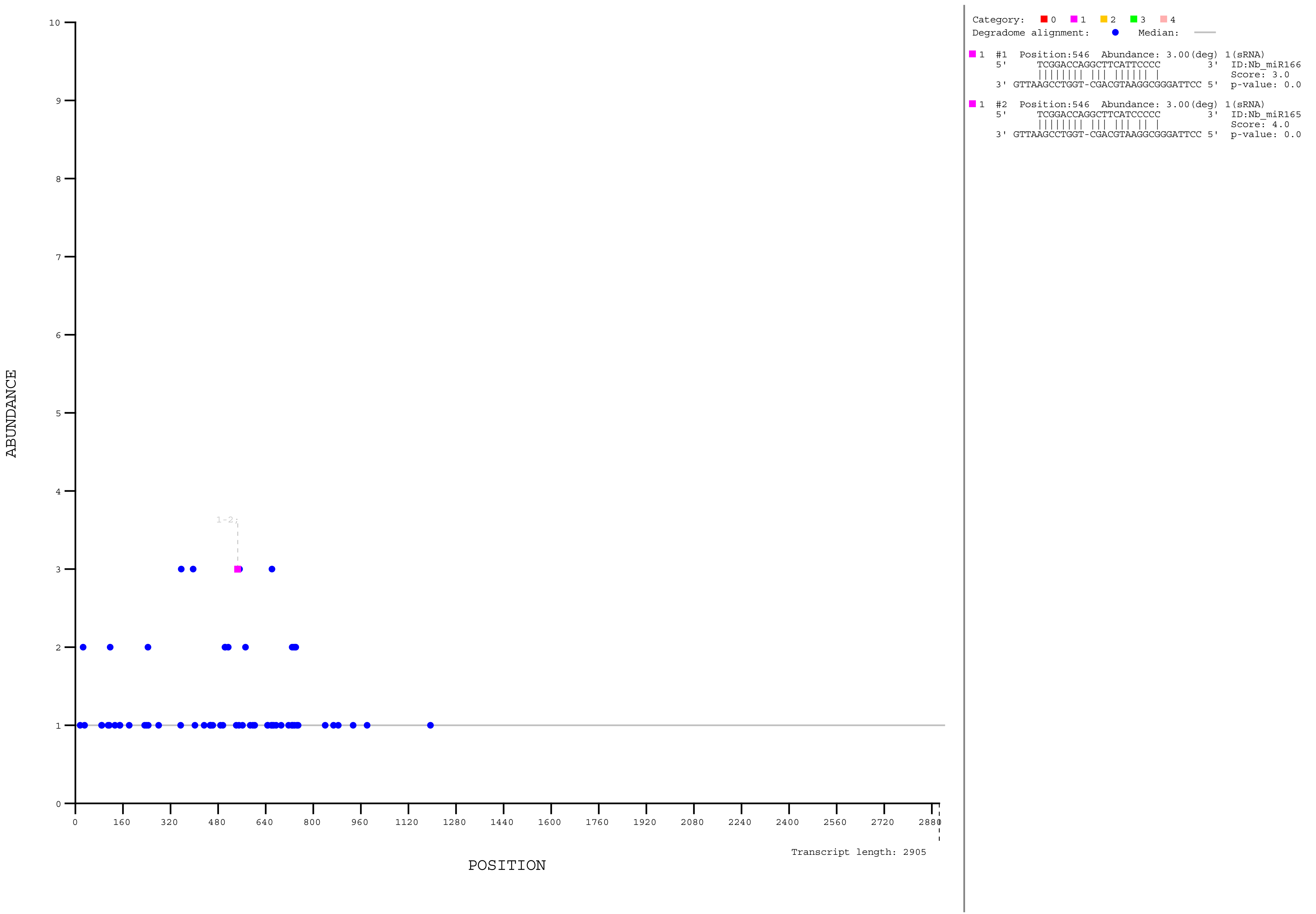

comp79742_c0_seq4 - 5-methyltetrahydropteroyltriglutamate--homocysteine methyltransferase

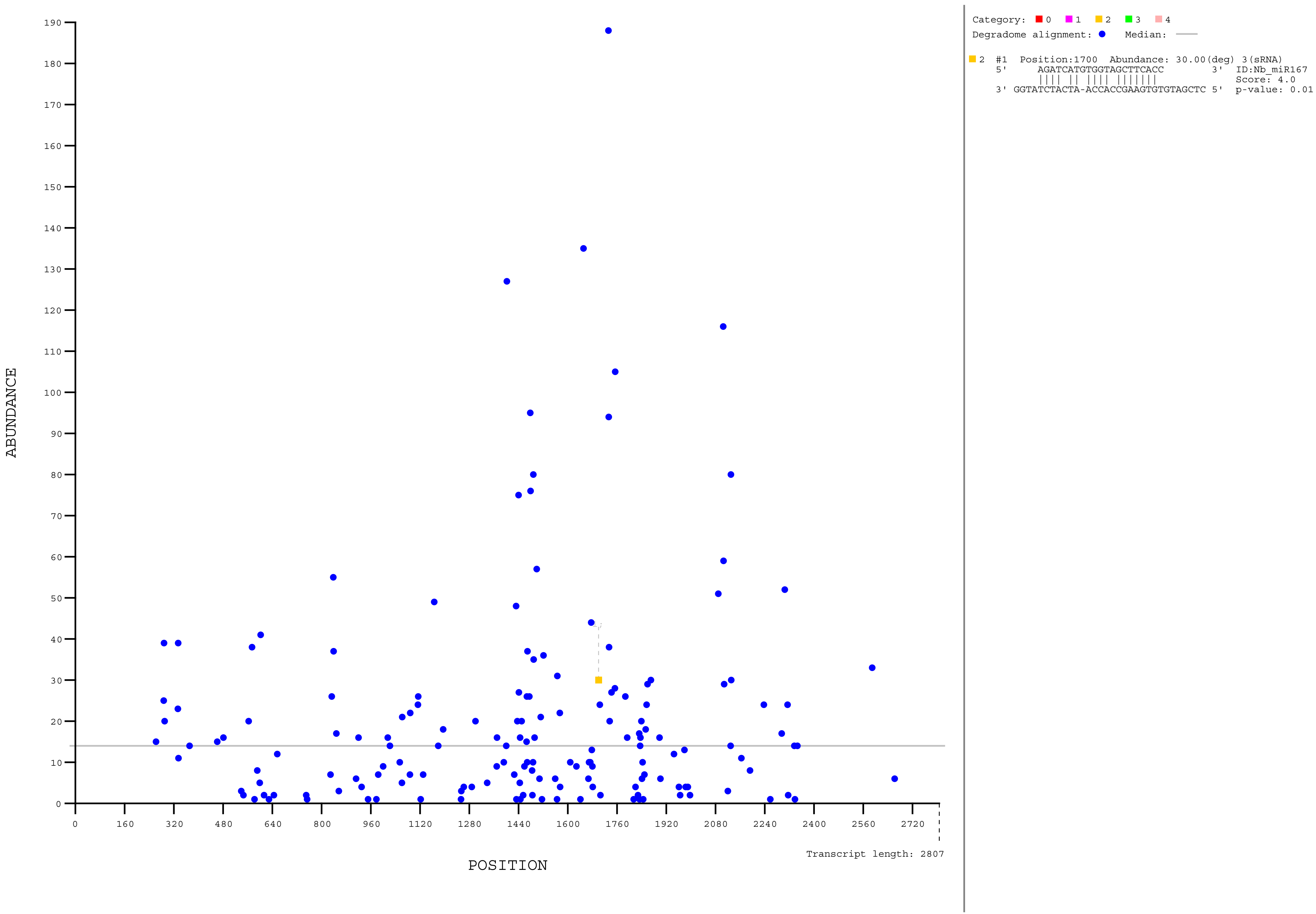

comp74516_c0_seq2 - Subtilisin-like protease

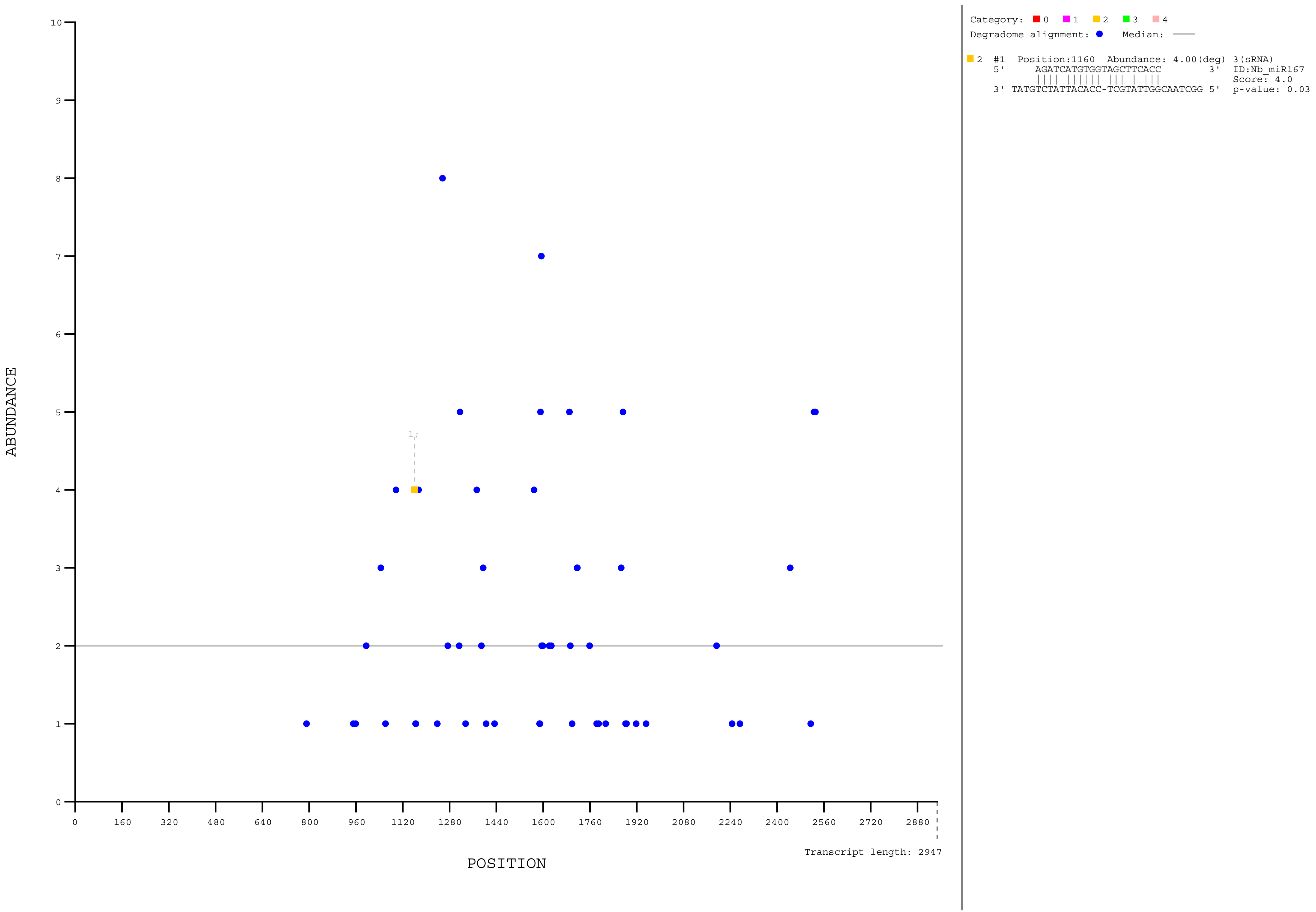

comp79927_c1_seq7 - Protein argonaute 1A

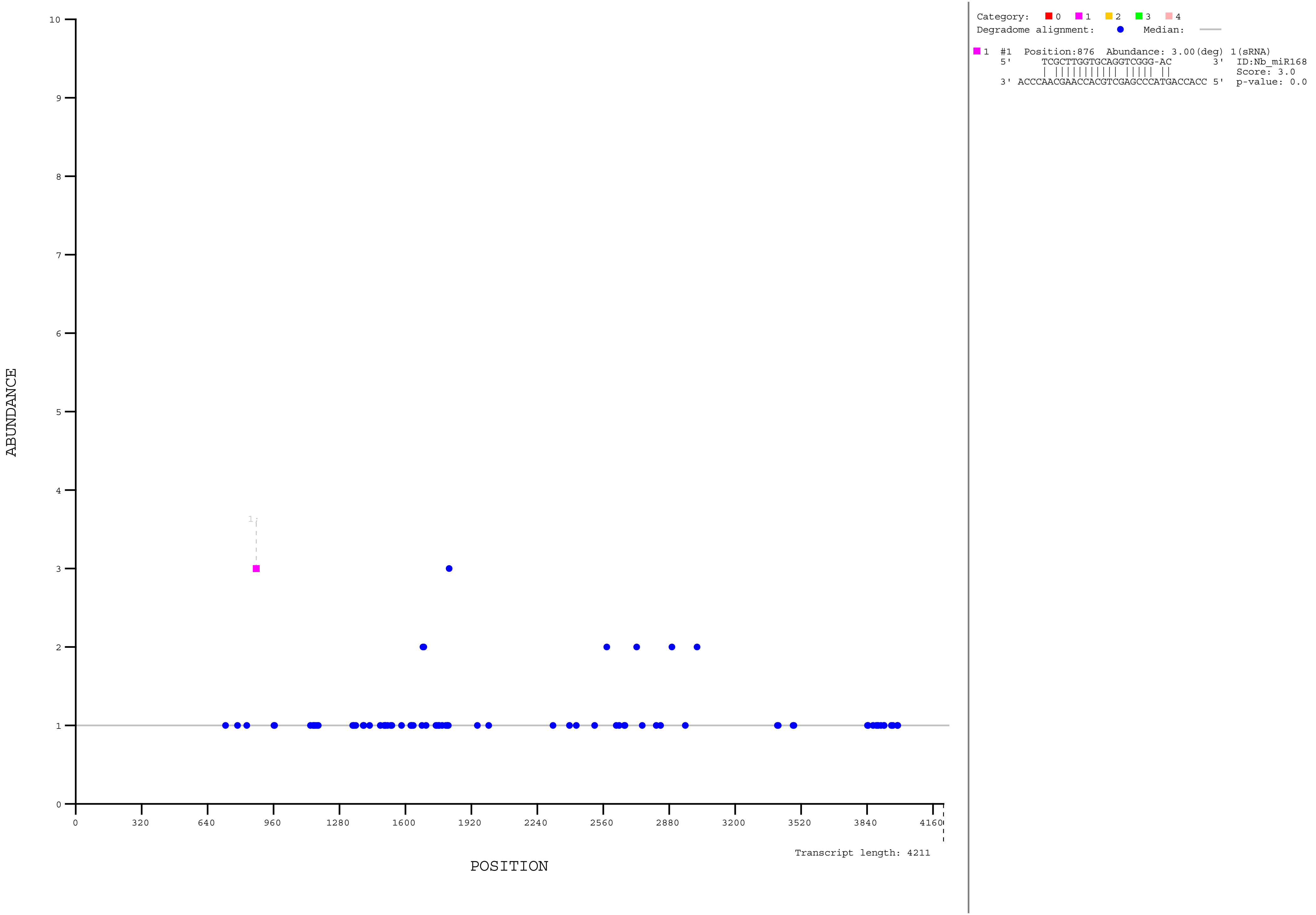

comp72459_c1_seq2 - Nuclear transcription factor Y subunit A-5

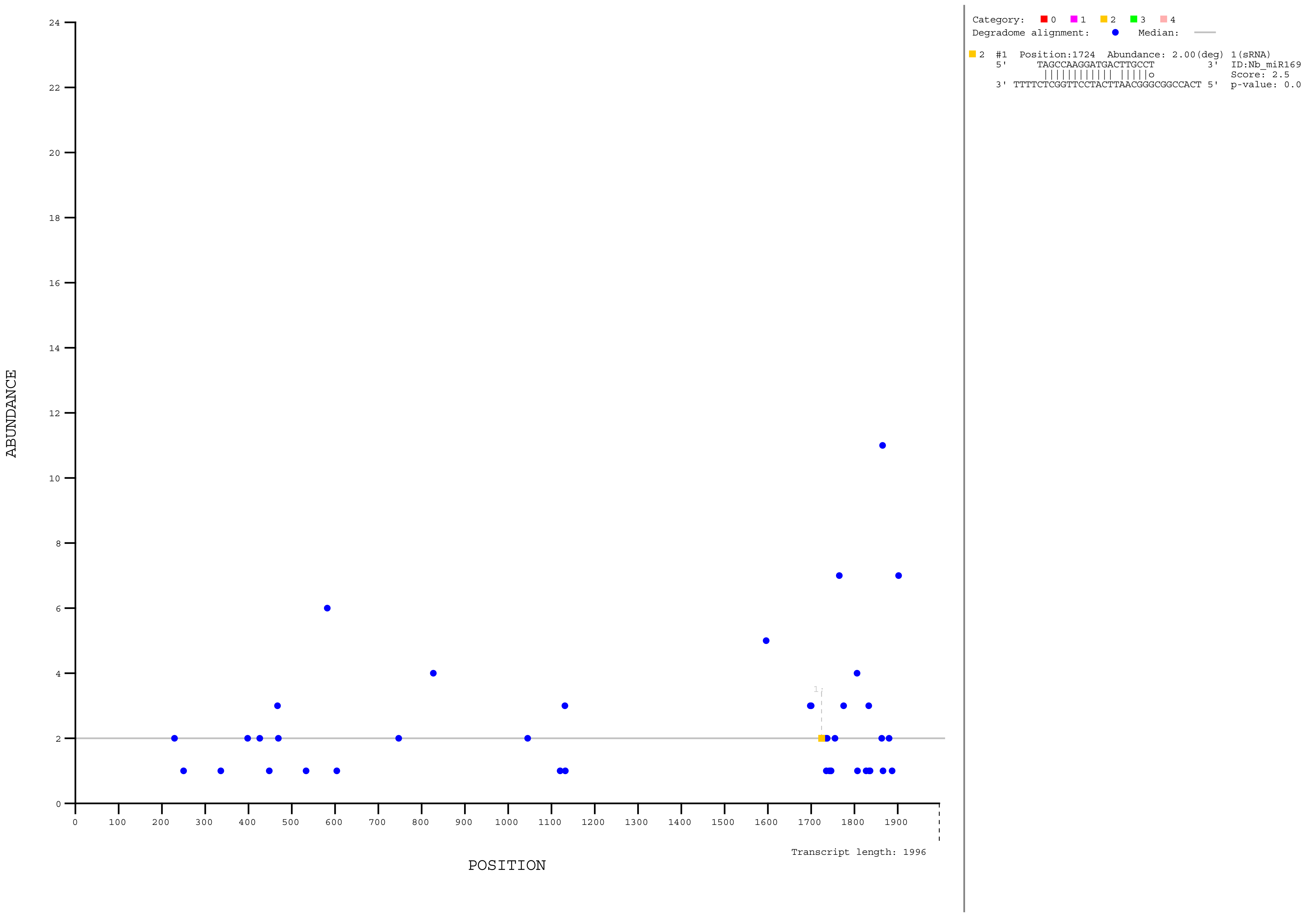

comp72338_c0_seq4 - Nuclear transcription factor Y subunit A-5

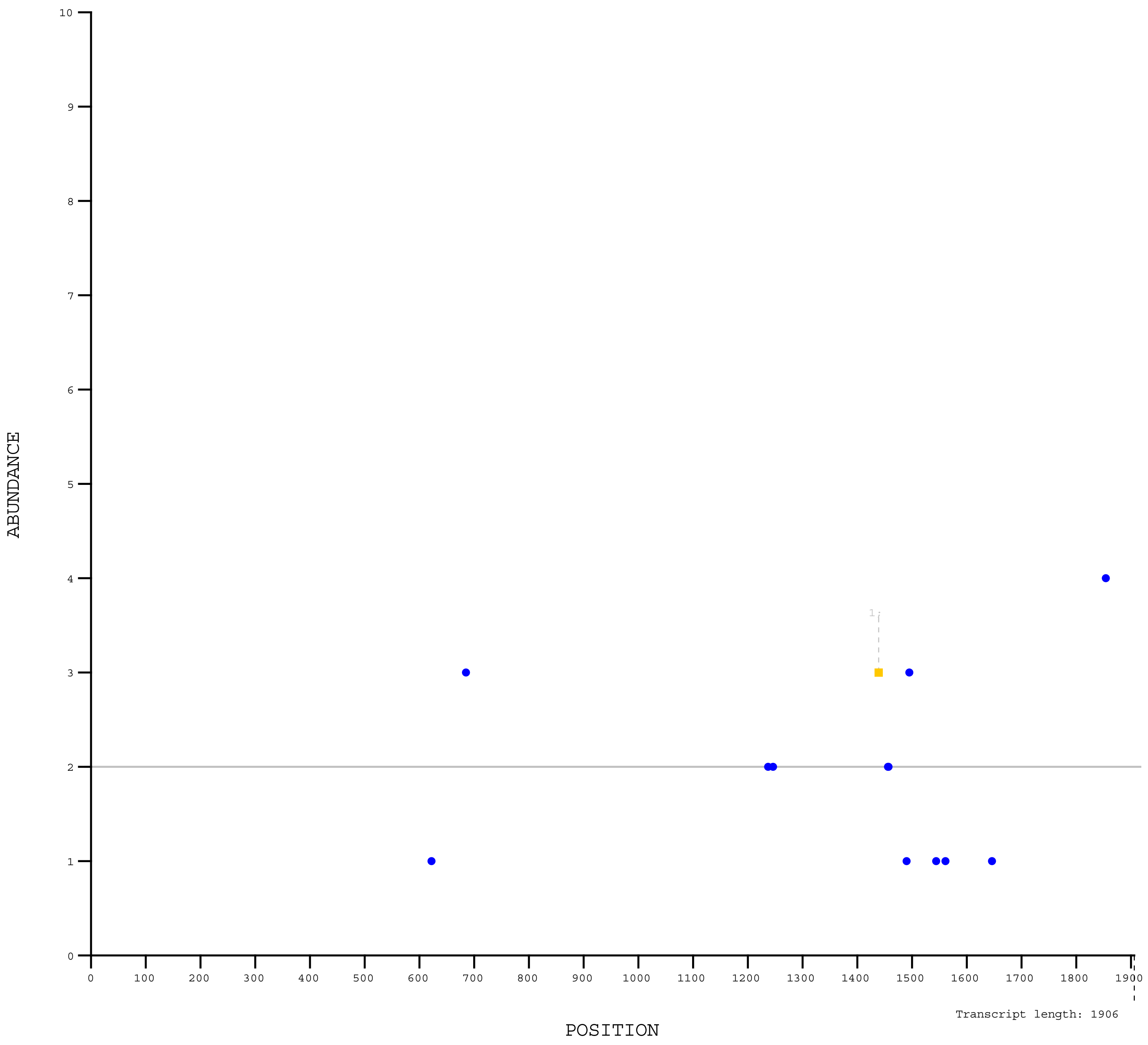

Category: 0 1 2 3 4
Degradome alignment: ● Median:

2 #1 Position:1439 Abundance: 3.00(deg) 1(sRNA)

```
5'     TAGCCAAGGATGACTTGCCT         3'  ID:Nb_miR169
        ||||||||||| ||||||              Score: 2.0
3' CTATTTCGGTTCCTACTAAACGGACGGCCACT 5'  p-value: 0.0
```

comp73705_c1_seq8 - Nuclear transcription factor Y subunit A-8

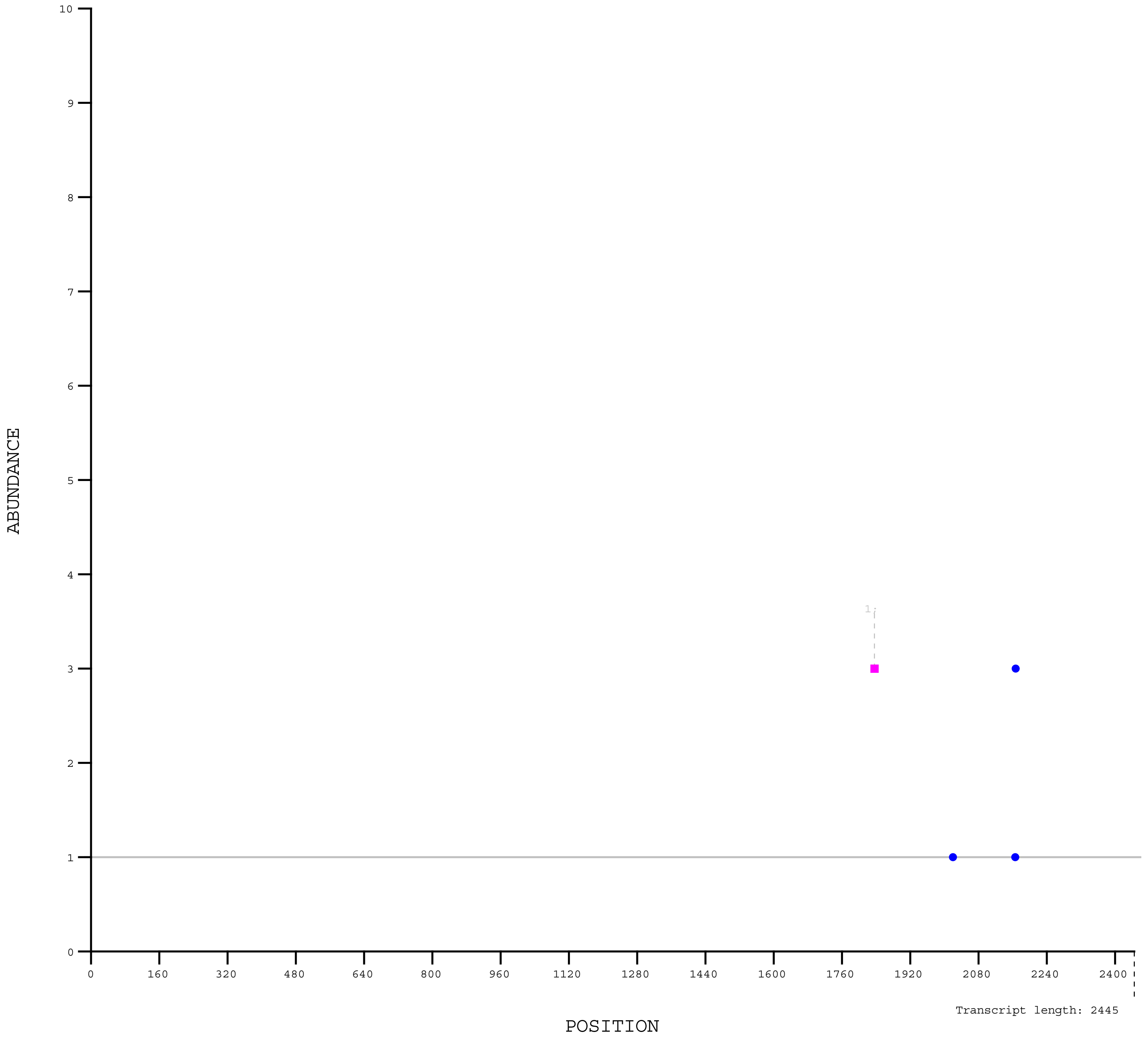

comp67524_c0_seq9 - Nuclear transcription factor Y subunit A-10

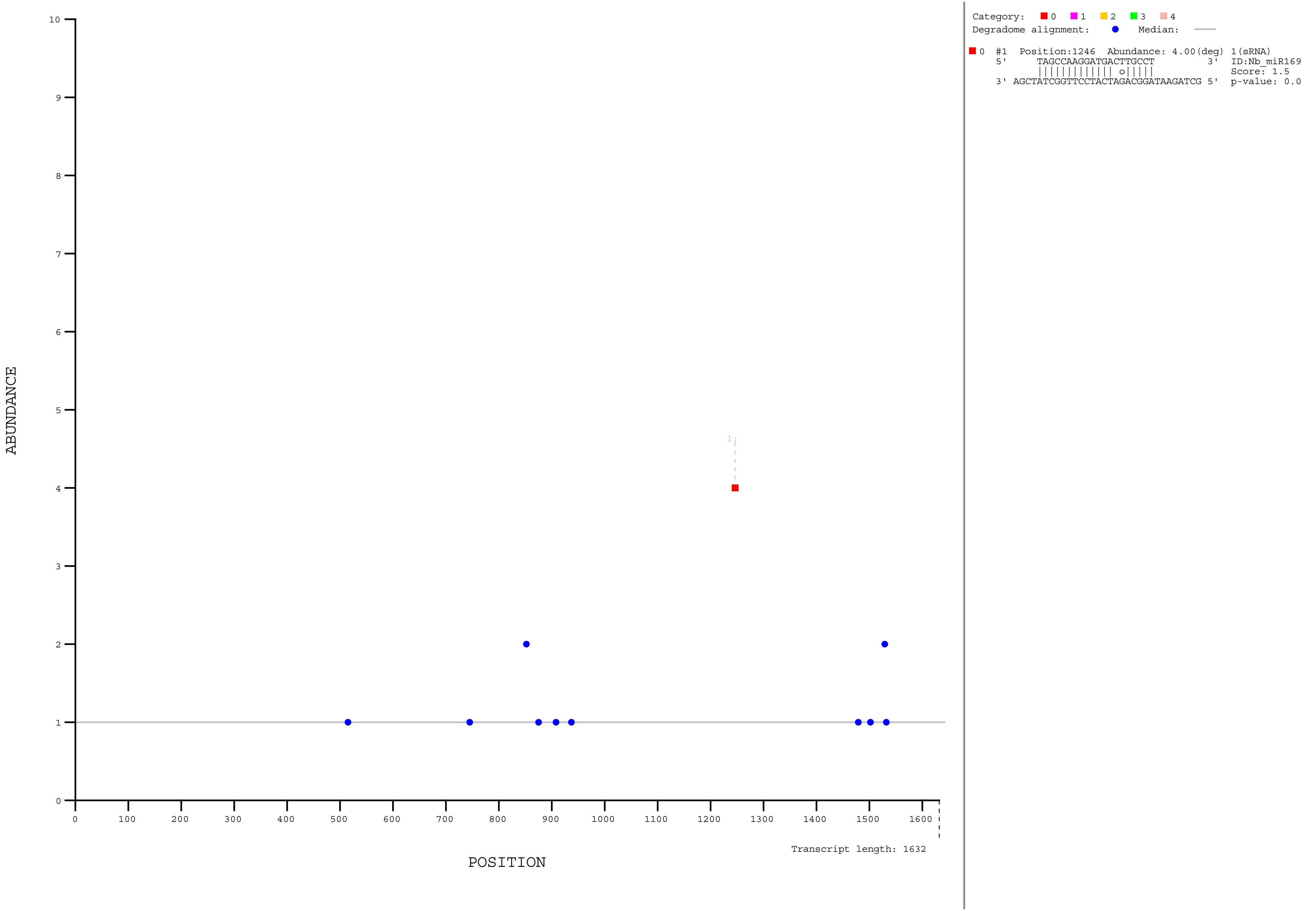

comp76988_c0_seq2 - Scarecrow-like protein 15

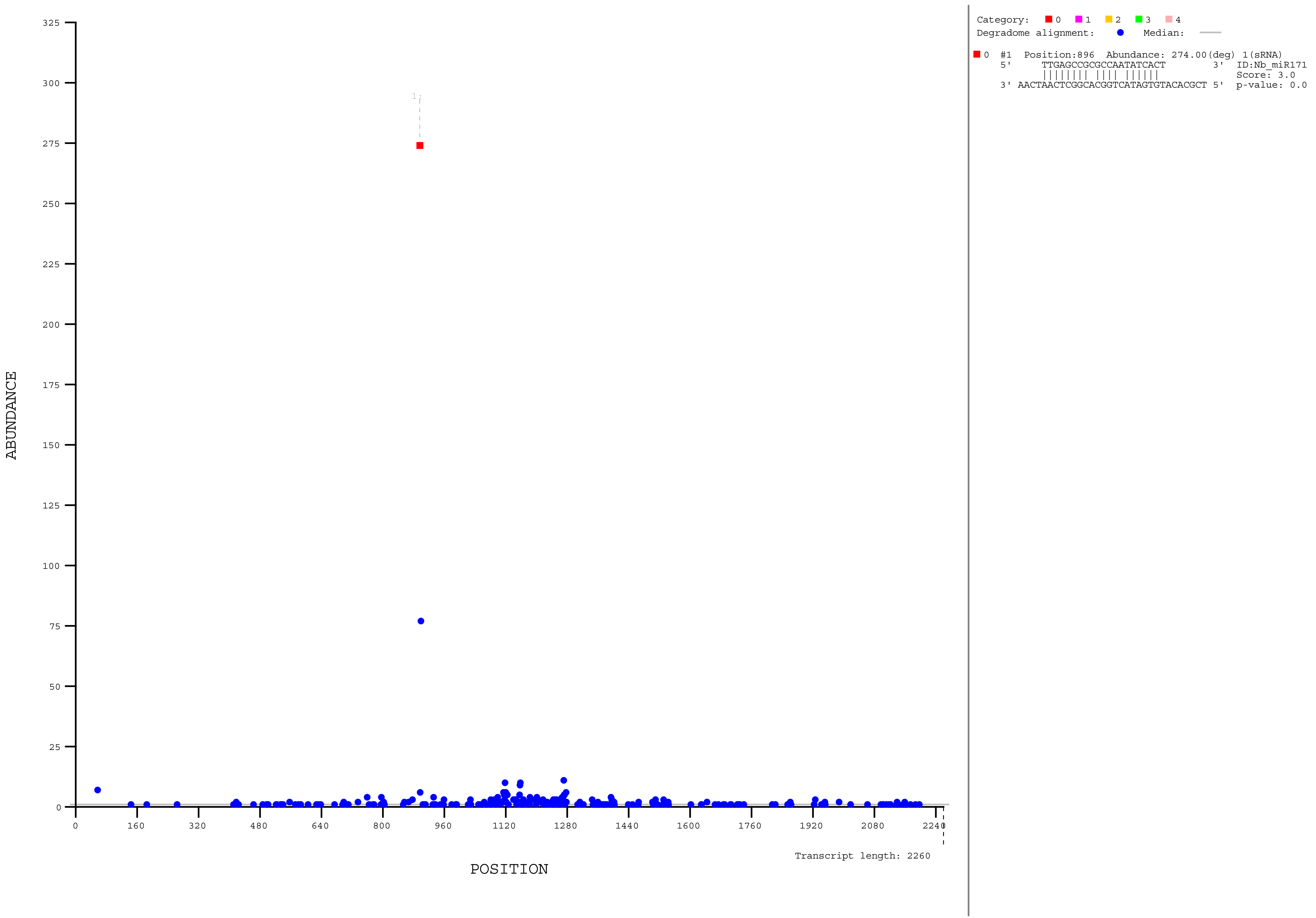

comp80119_c0_seq4 - Scarecrow-like protein 6

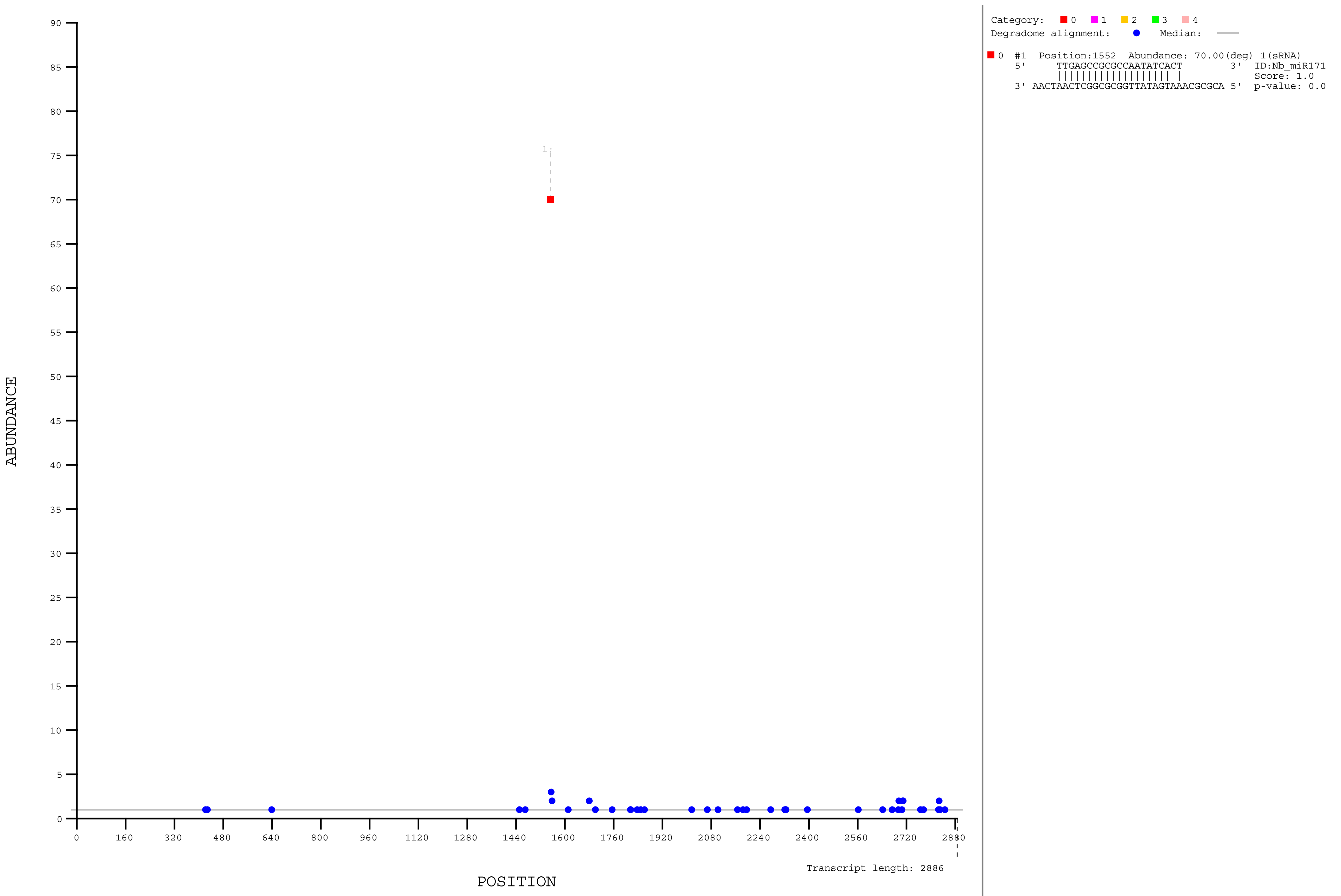

comp75200_c0_seq2 - Scarecrow-like protein 6

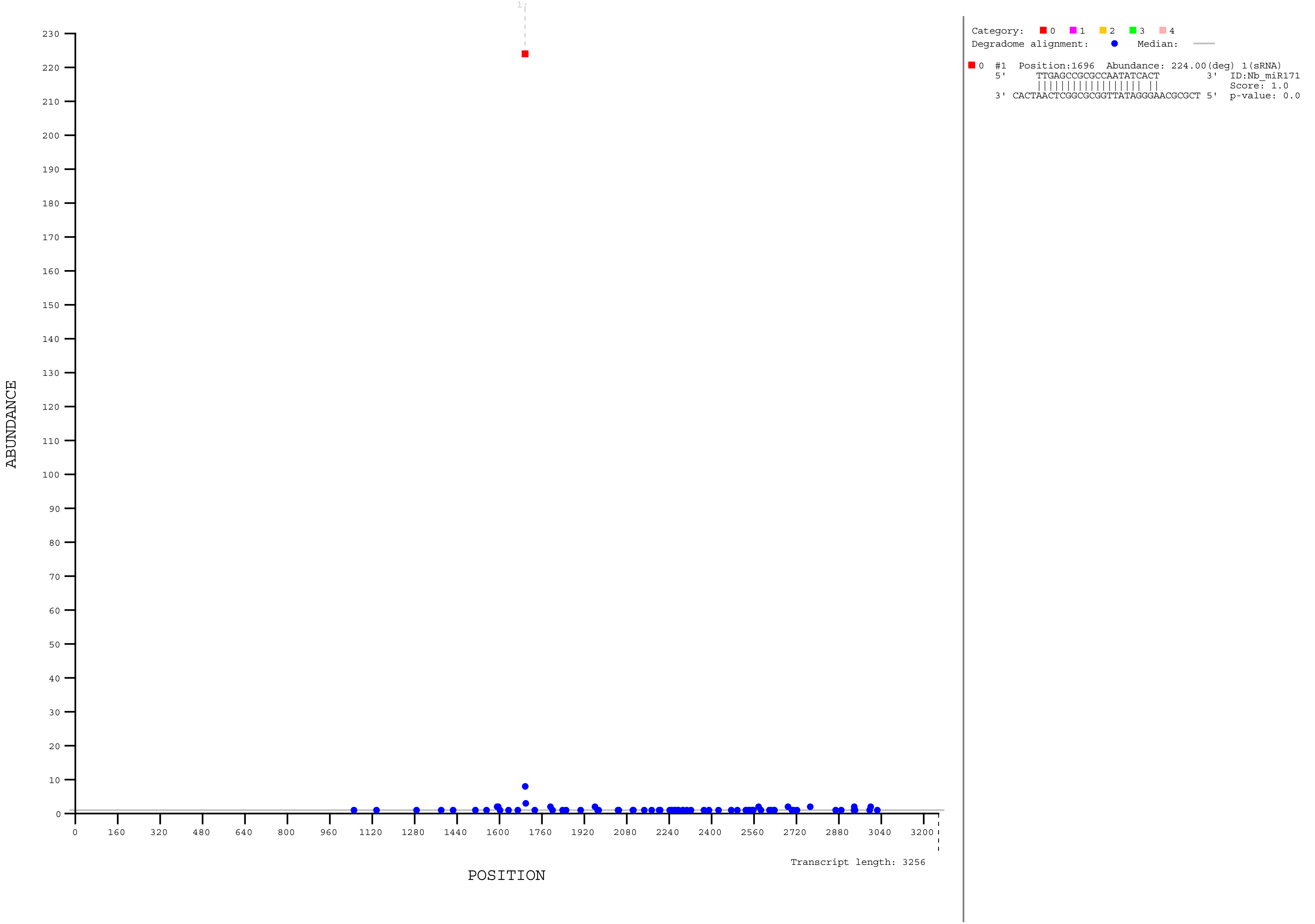

comp78340_c2_seq4 - Floral homeotic protein APETALA 2

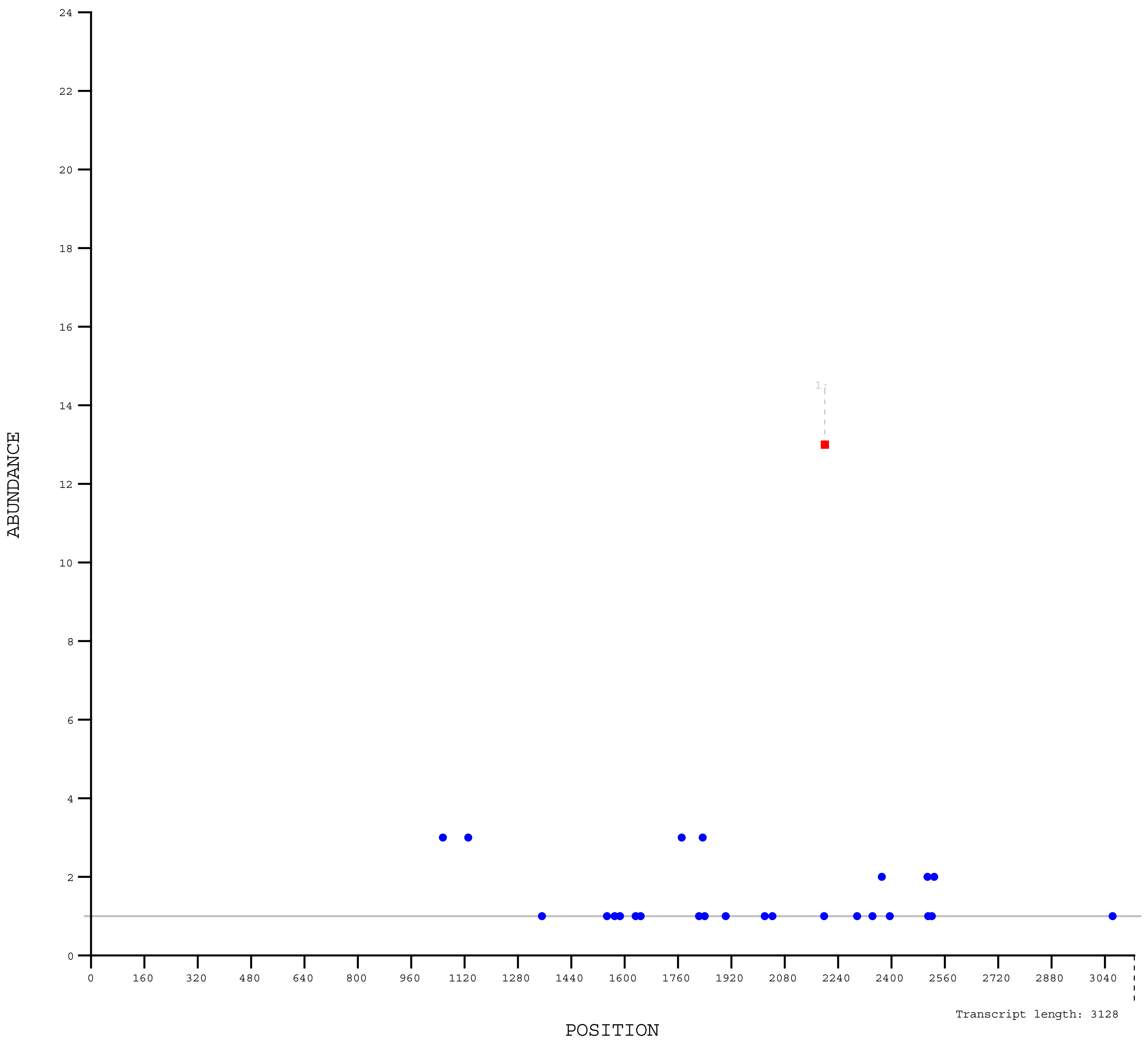

Category: 0 1 2 3 4
Degradome alignment: Median:

0 #1 Position:2200 Abundance: 13.00(deg) 1(sRNA)
5' AGAATCTTGATGATGCTGCAT 3' ID:Nb_miR172
|||||o||||||||||||| Score: 2.5
3' ACCCCCTTAGGACTACTACGACGTCGCCGACG 5' p-value: 0.0

comp68096_c0_seq1 - Transcription factor TCP4

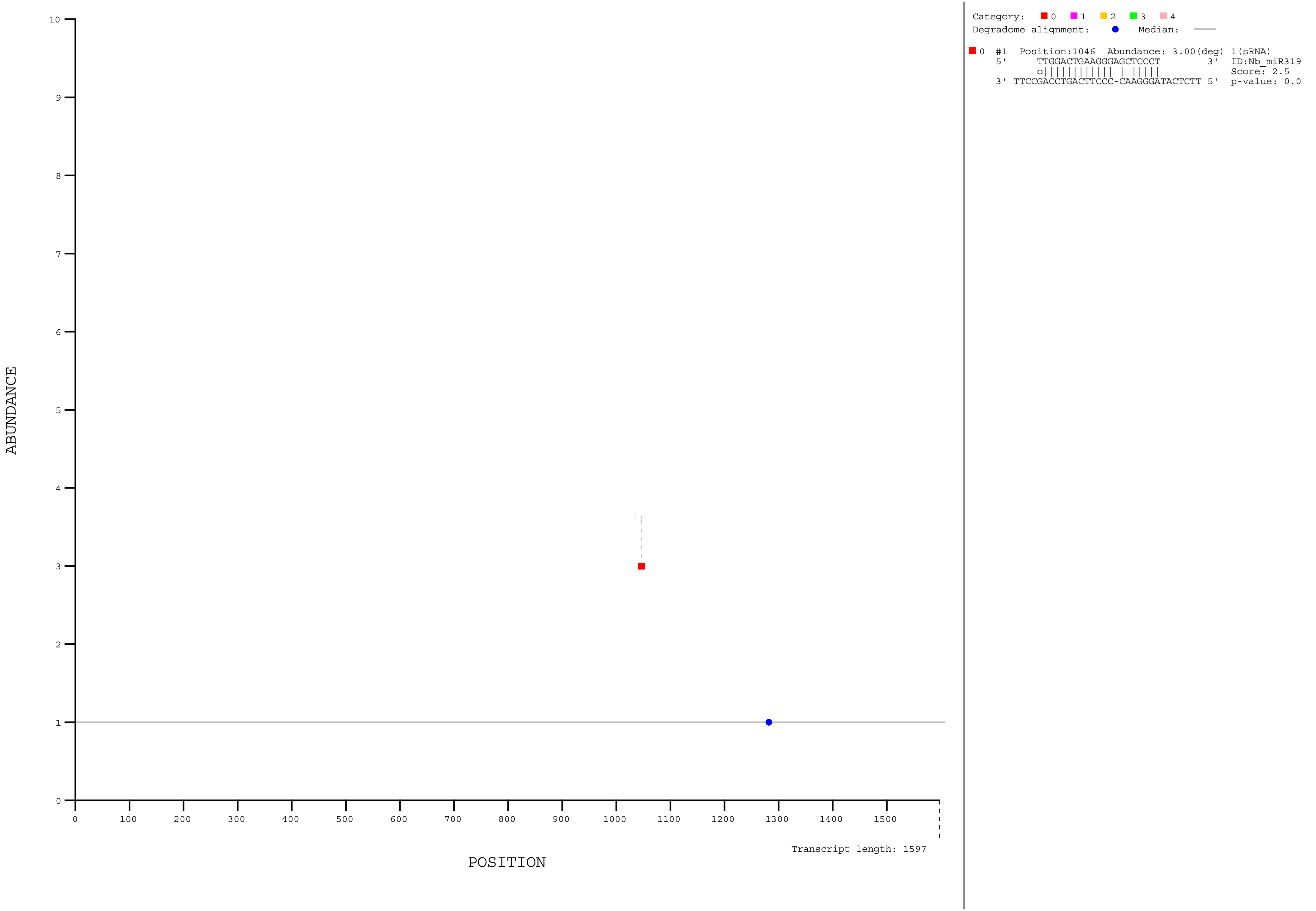

comp73714_c2_seq12 - Transcription factor TCP2

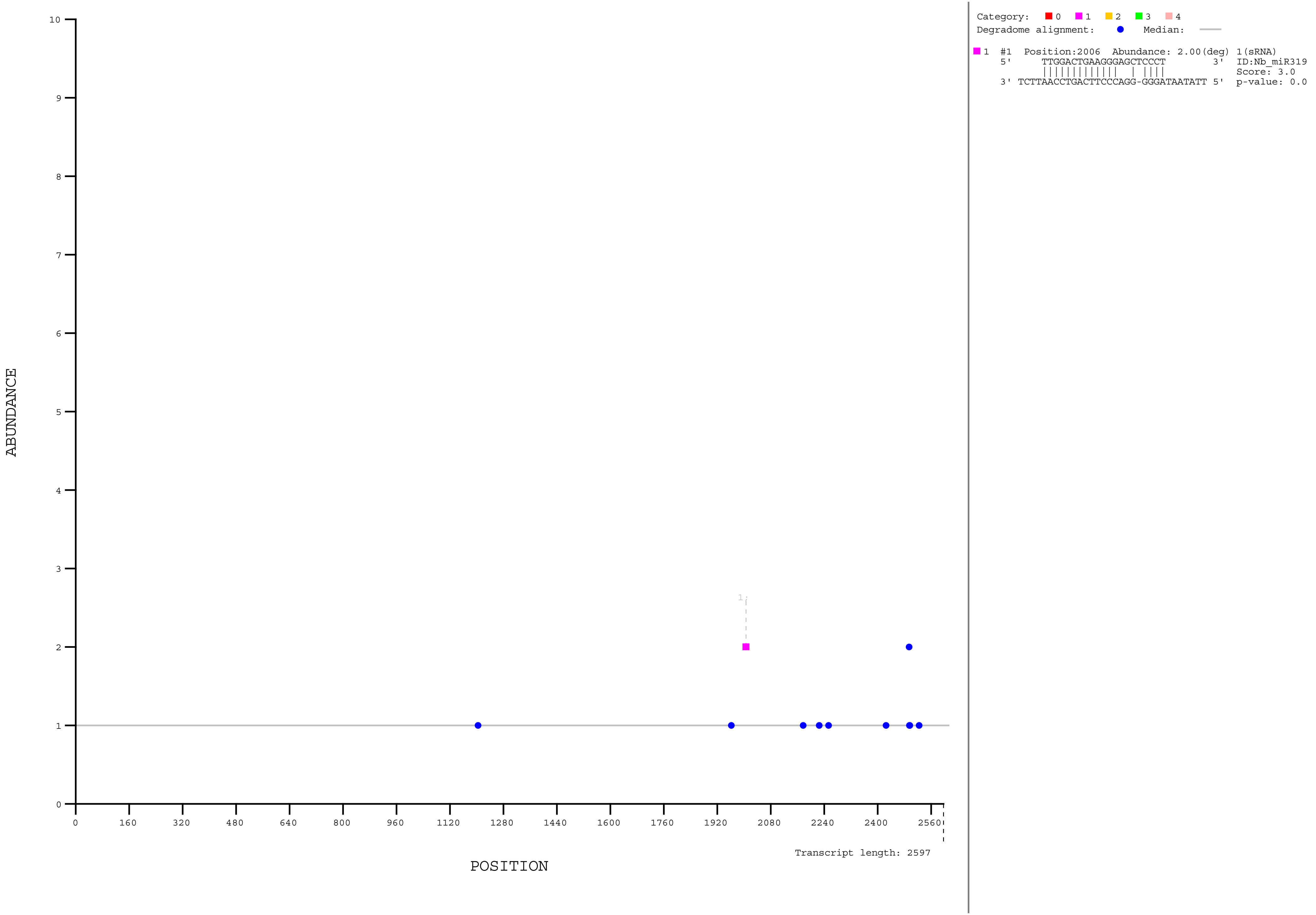

comp80206_c0_seq2 - no annotation

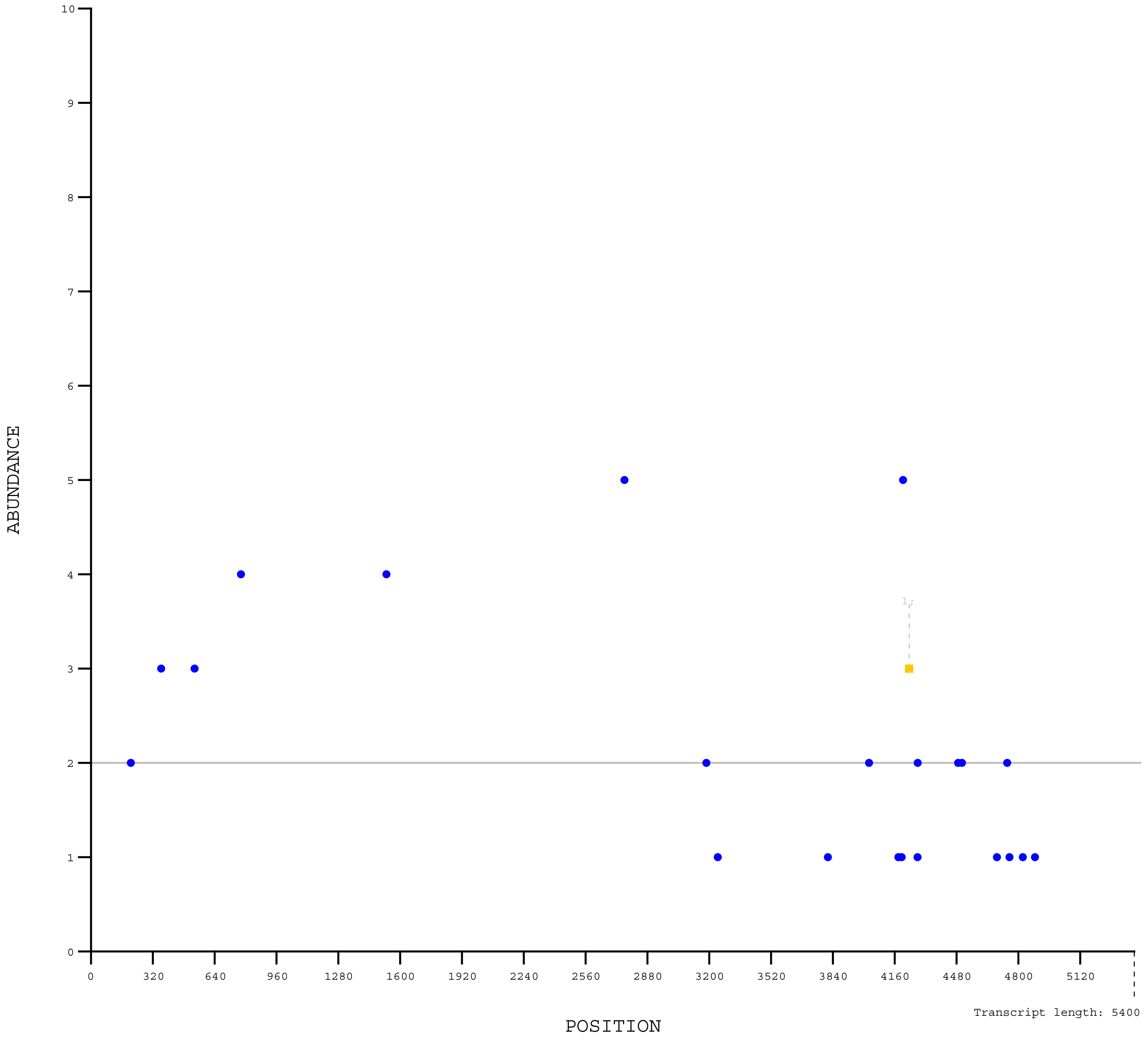

Category: 0 1 2 3 4
Degradome alignment: Median:

2 #1 Position:4235 Abundance: 3.00(deg) 1(sRNA)

```
5'     TCCAAAGGGATCGCATTGATCC       3'  ID:Nb_miR393
          |||| |||||| |||oo||||         Score: 4.0
3' CAGACGGTTGCCCTAG-GTAGTTAGGCGGTAG 5'  p-value: 0.05
```

comp60101_c0_seq1 - Tripeptidyl-peptidase 2

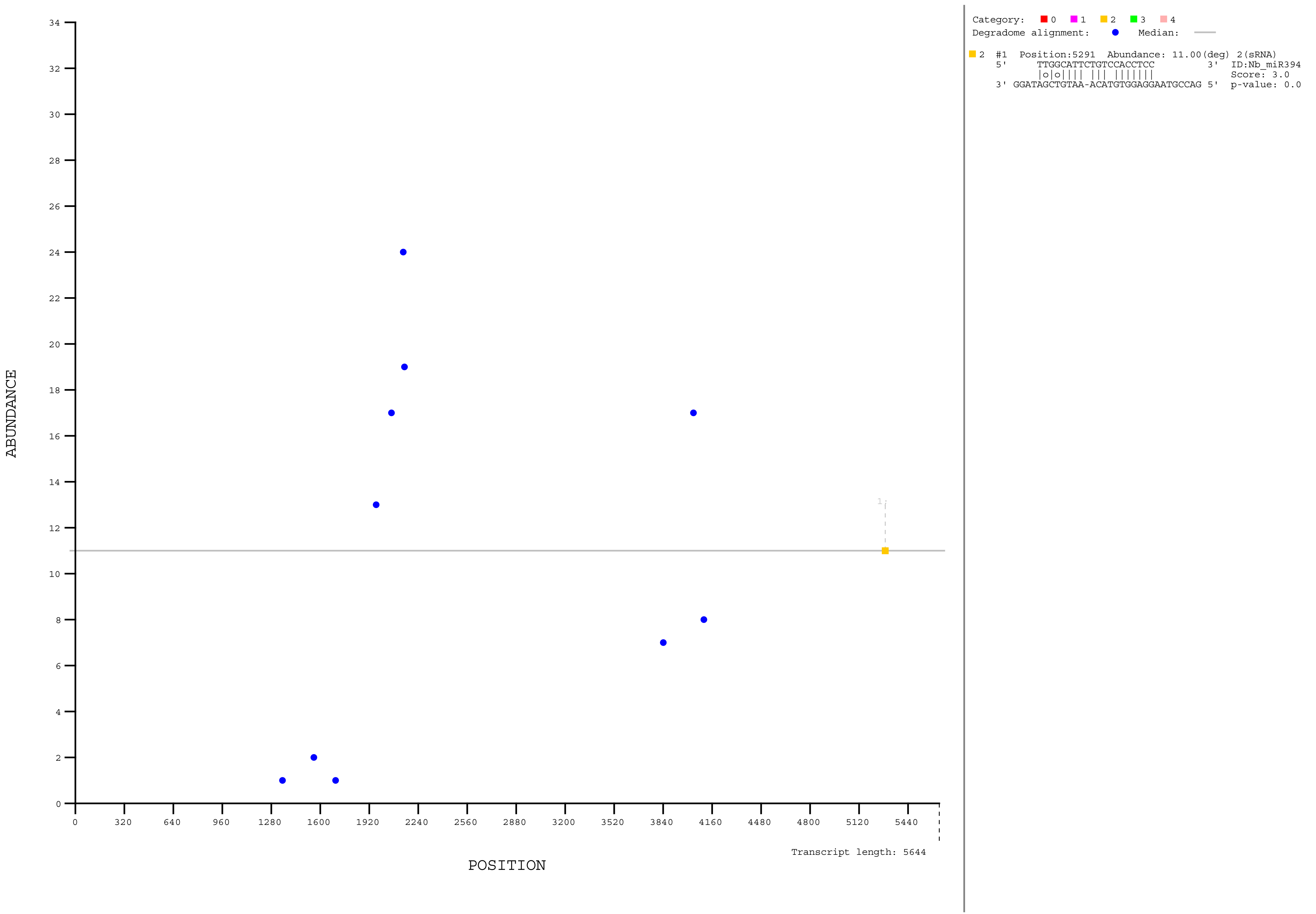

comp75863_c0_seq1 - ATP sulfurylase 1, chloroplastic

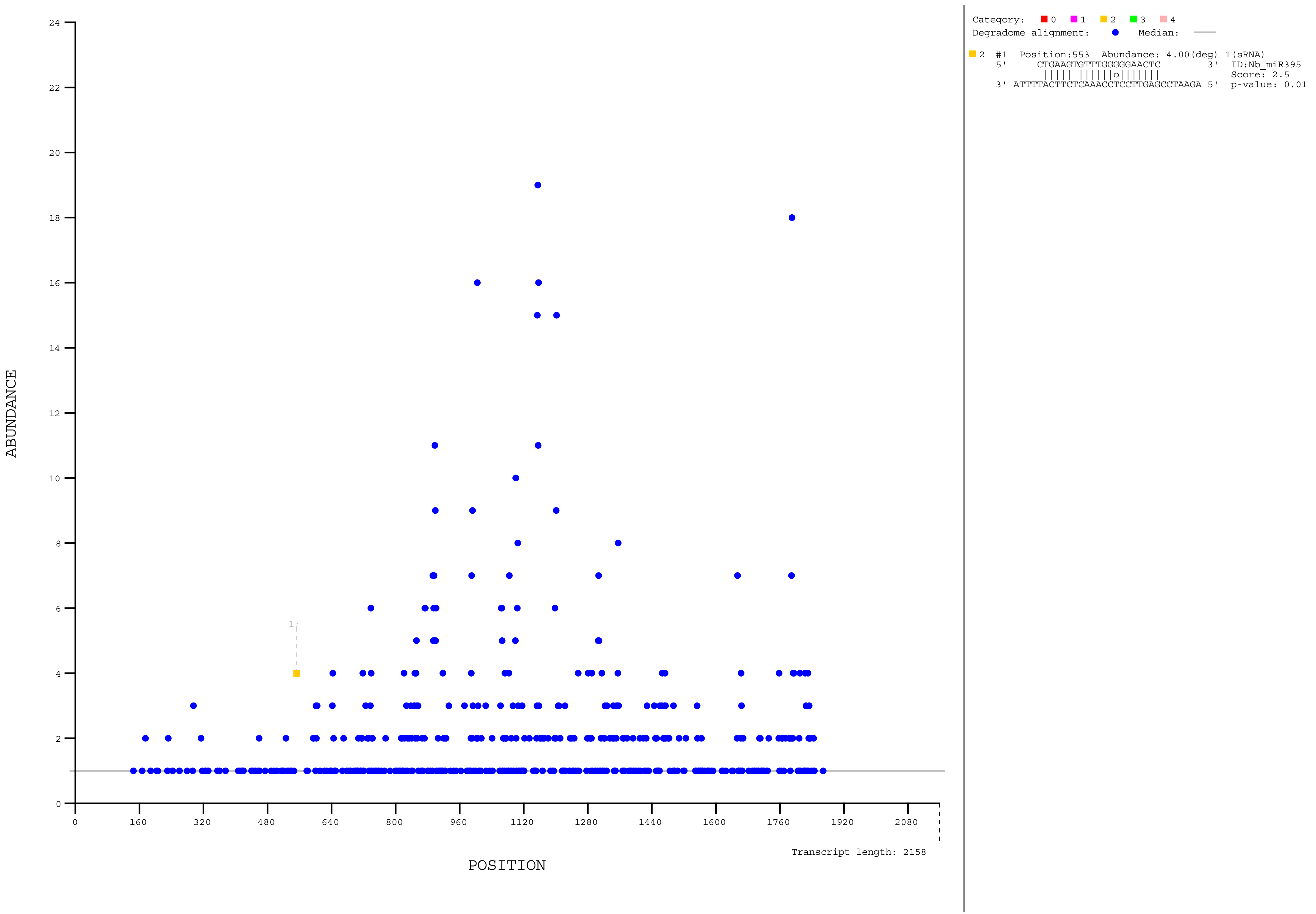

comp29318_c1_seq1 - Sulfate transporter 2.1

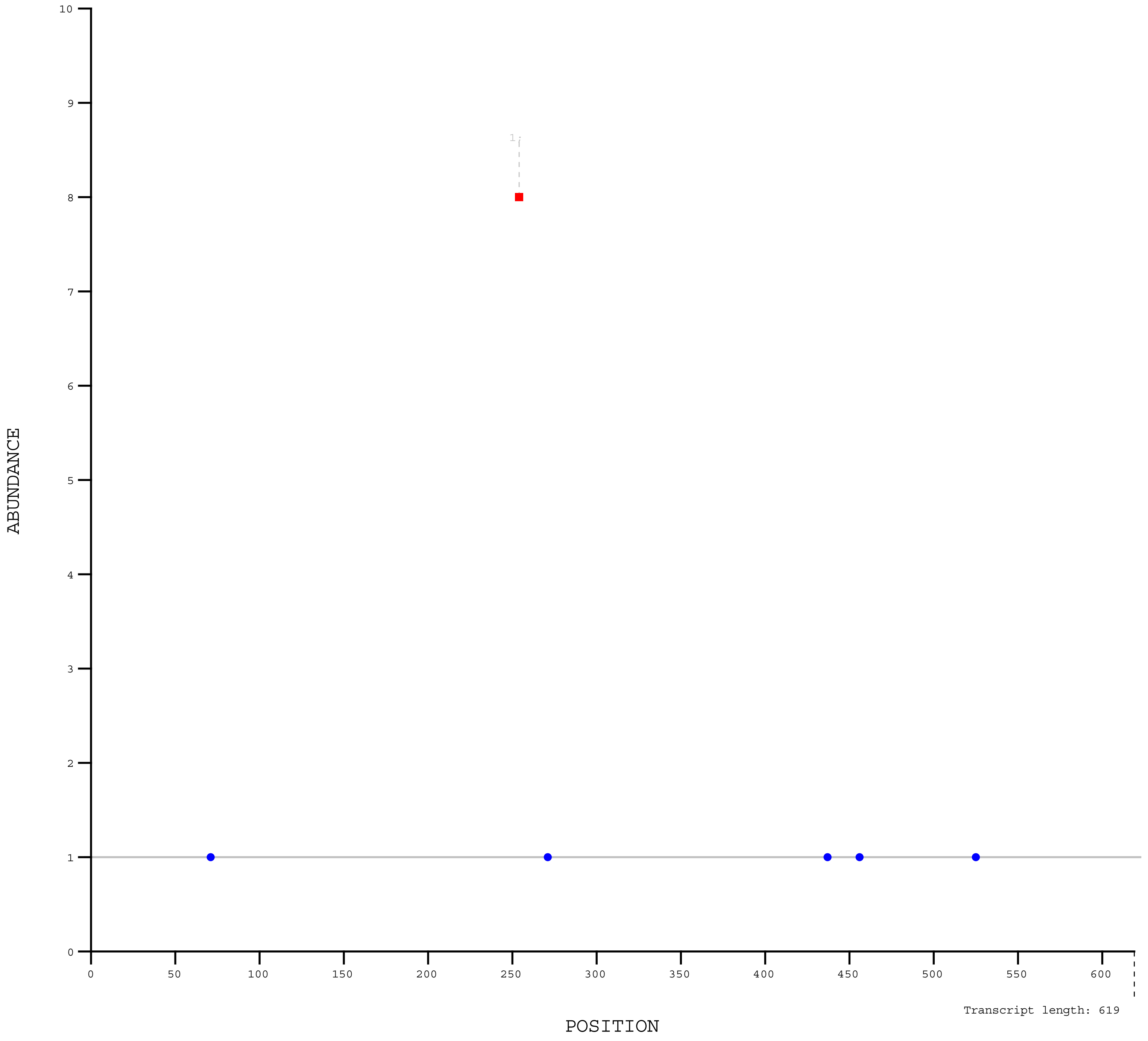


```
Category:  ■ 0  ■ 1  ■ 2  ■ 3  ■ 4
Degradome alignment:  ●   Median:  ——

■ 0  #1  Position:254  Abundance: 8.00(deg) 1(sRNA)
     5'      CTGAAGTGTTTGGGGGAACTC         3'  ID:Nb_miR395
              |||||||||||||o|||||              Score: 2.5
     3' GATGAACTTCACAAACCCTCTTGACTACTGTT 5'  p-value: 0.0
```

comp71133_c0_seq1 - Protein THYLAKOID FORMATION1, chloroplastic

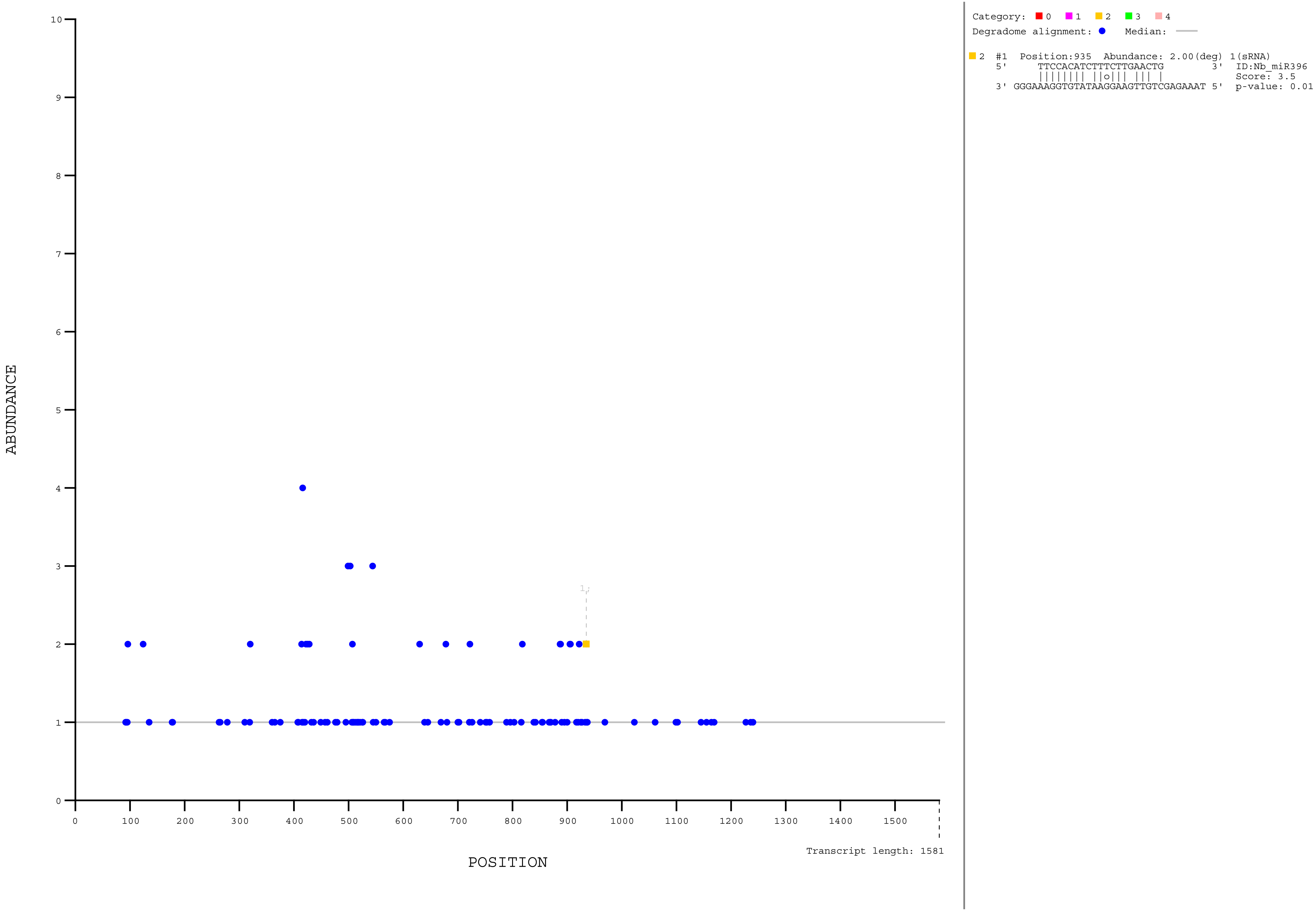

comp77639_c0_seq4 - Growth-regulating factor 6

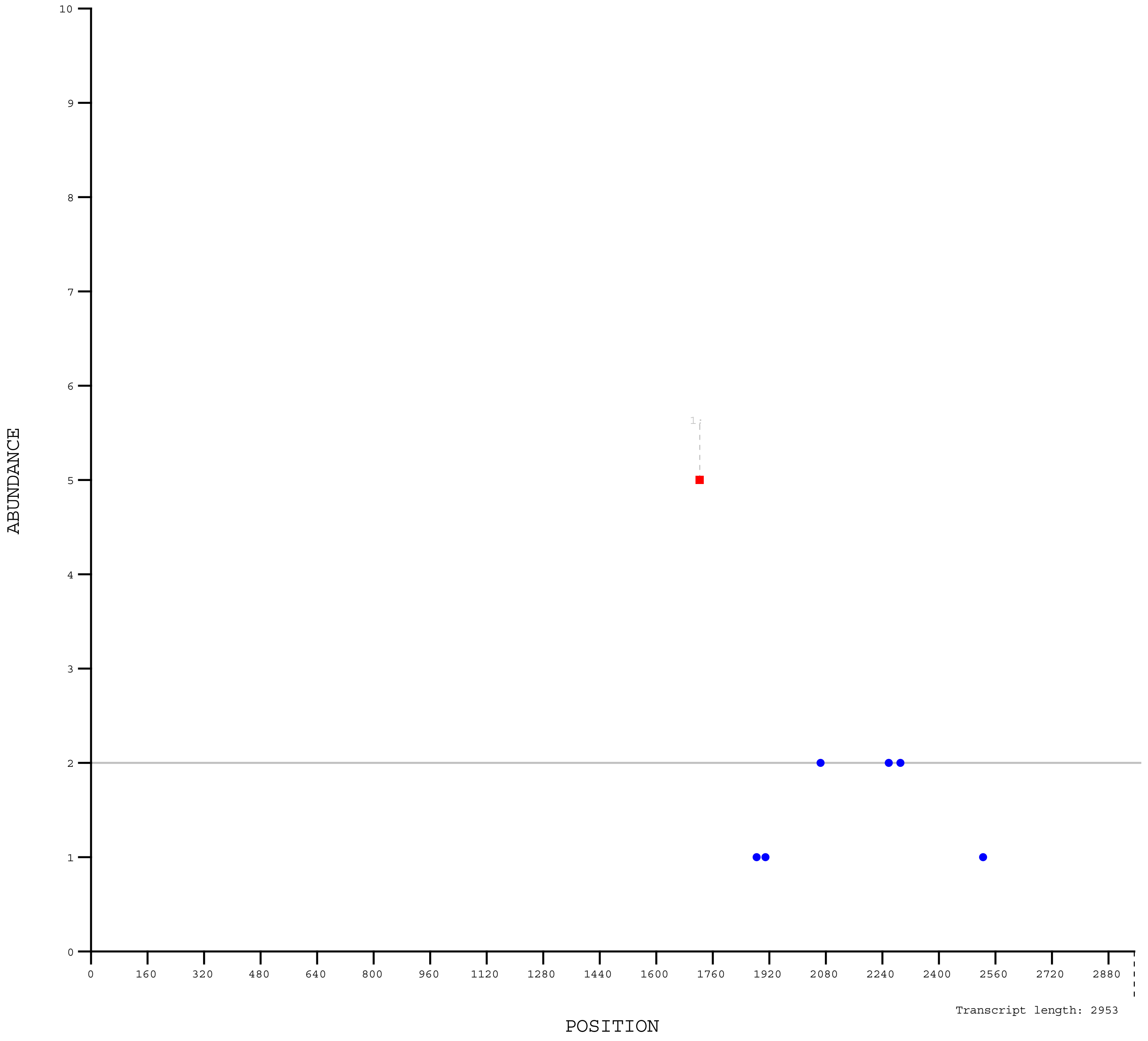


```
Category:  0  1  2  3  4
Degradome alignment:      Median:

0  #1  Position:1723  Abundance: 5.00(deg) 1(sRNA)
   5'    TTCCACA-GCTTTCTTGAACTG        3'  ID:Nb_miR396
         ||||||| ||||||||||||| o           Score: 2.5
   3' AGGAAGGTGTCCGAAAGAACTTGCTACCGCCG 5'  p-value: 0.0
```

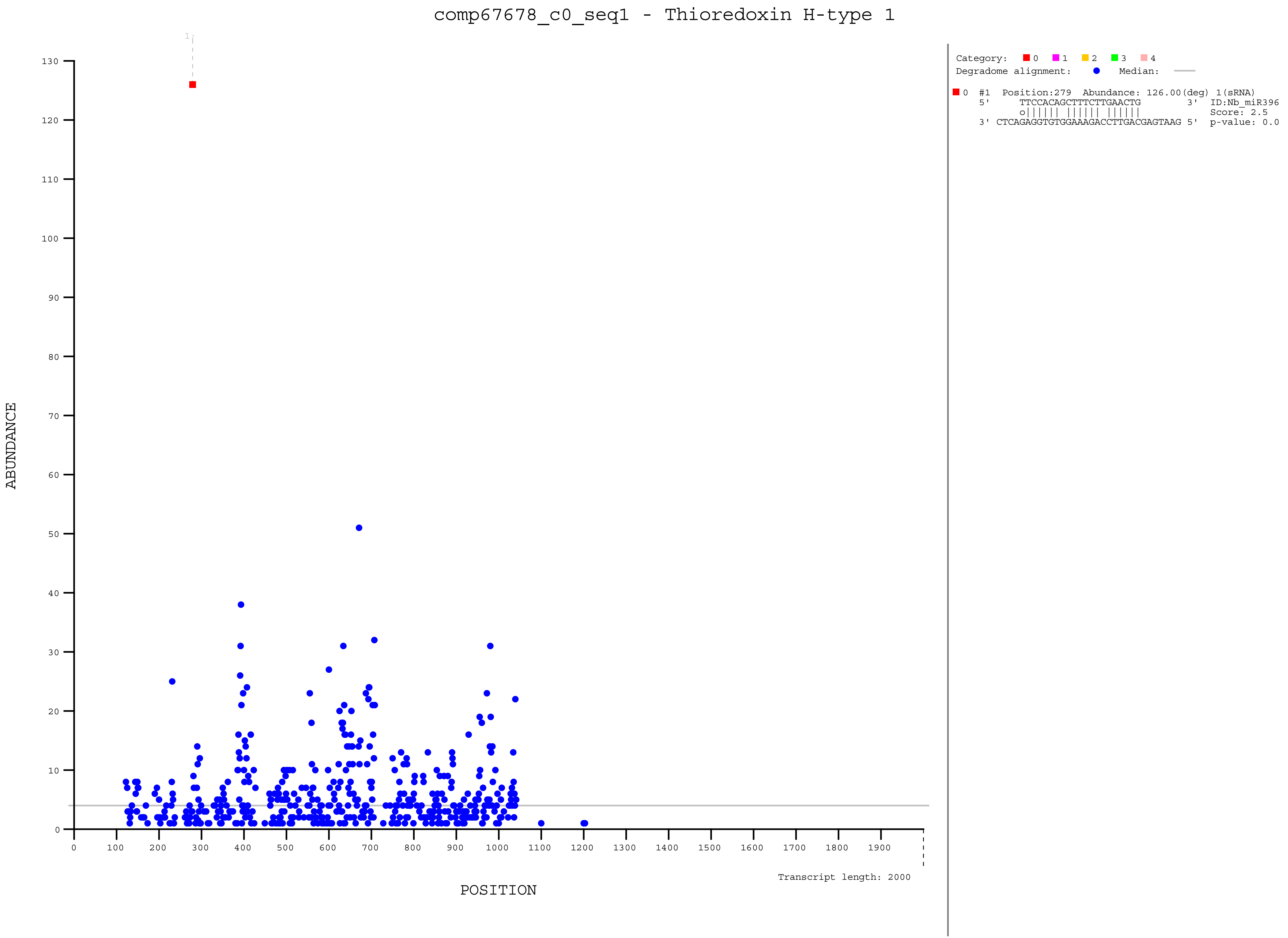
comp67678_c0_seq1 - Thioredoxin H-type 1
ABUNDANCE
130
120
110
100
90
80
70
60
50
40
30
20
10
0
1
0 100 200 300 400 500 600 700 800 900 1000 1100 1200 1300 1400 1500 1600 1700 1800 1900
POSITION
Transcript length: 2000
Category: 0 1 2 3 4
Degradome alignment:
Median:
0 #1 Position:279 Abundance: 126.00(deg) 1(sRNA)
5' TTCCACAGCTTTCTTGAACTG 3' ID:Nb_miR396
o|||||| |||||| ||||||| Score: 2.5
3' CTCAGAGGTGTGGAAAGACCTTGACGAGTAAG 5' p-value: 0.0

comp50987_c1_seq1 - 40S ribosomal protein S15

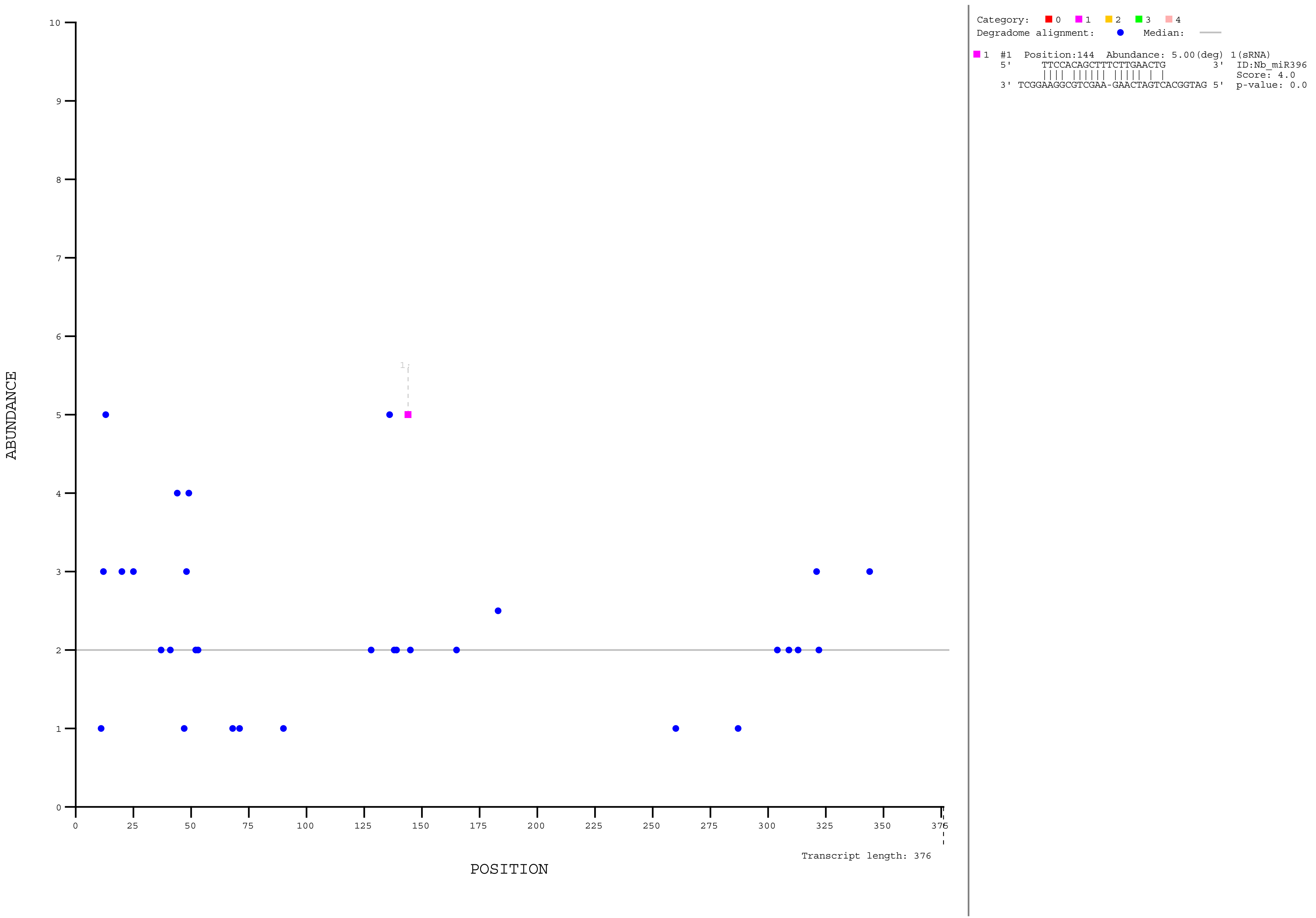

comp69468_c0_seq1 - Protein IQ-DOMAIN 14

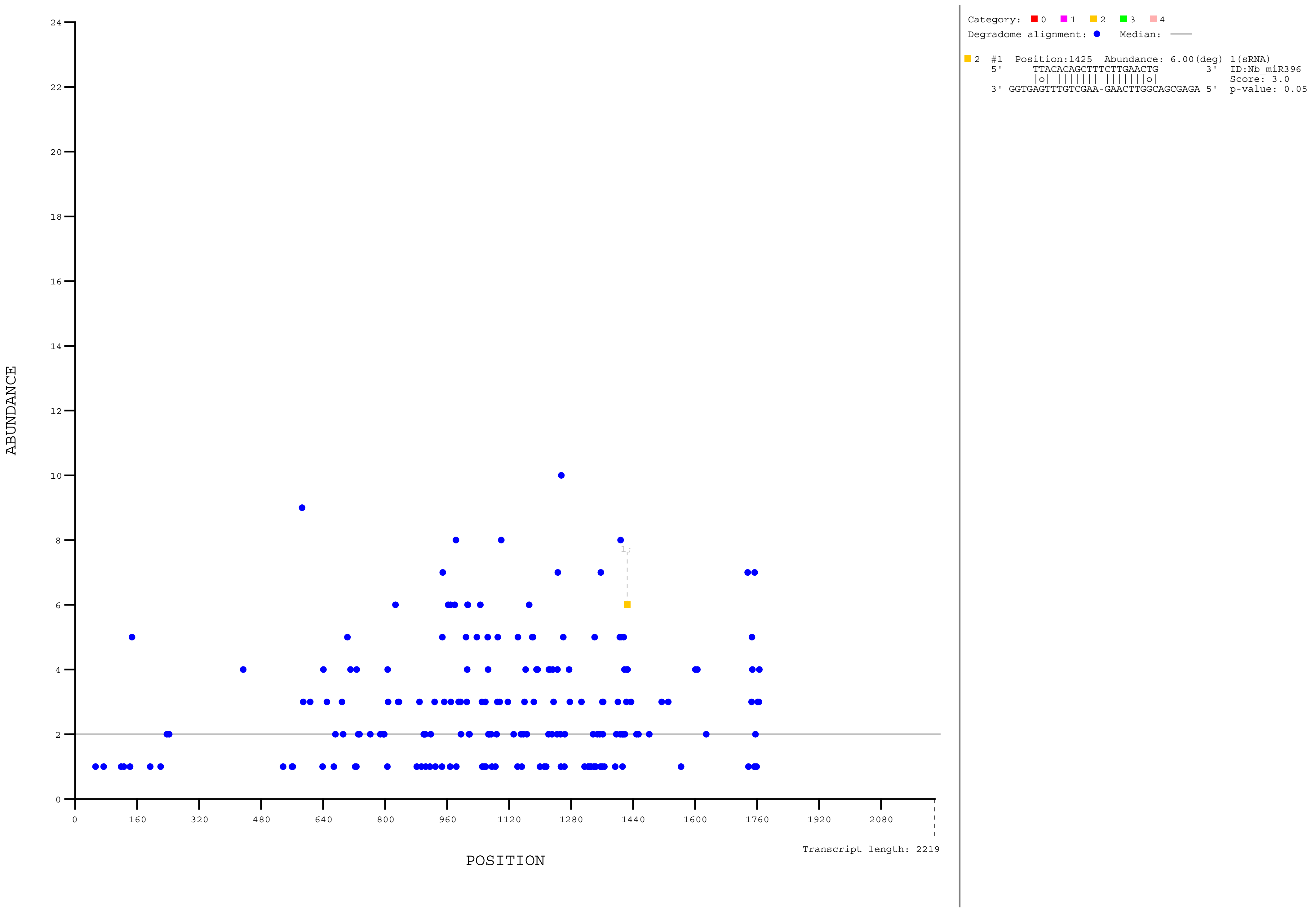

comp77795_c0_seq2 - Pentatricopeptide repeat-containing protein At5g04810, chloroplastic

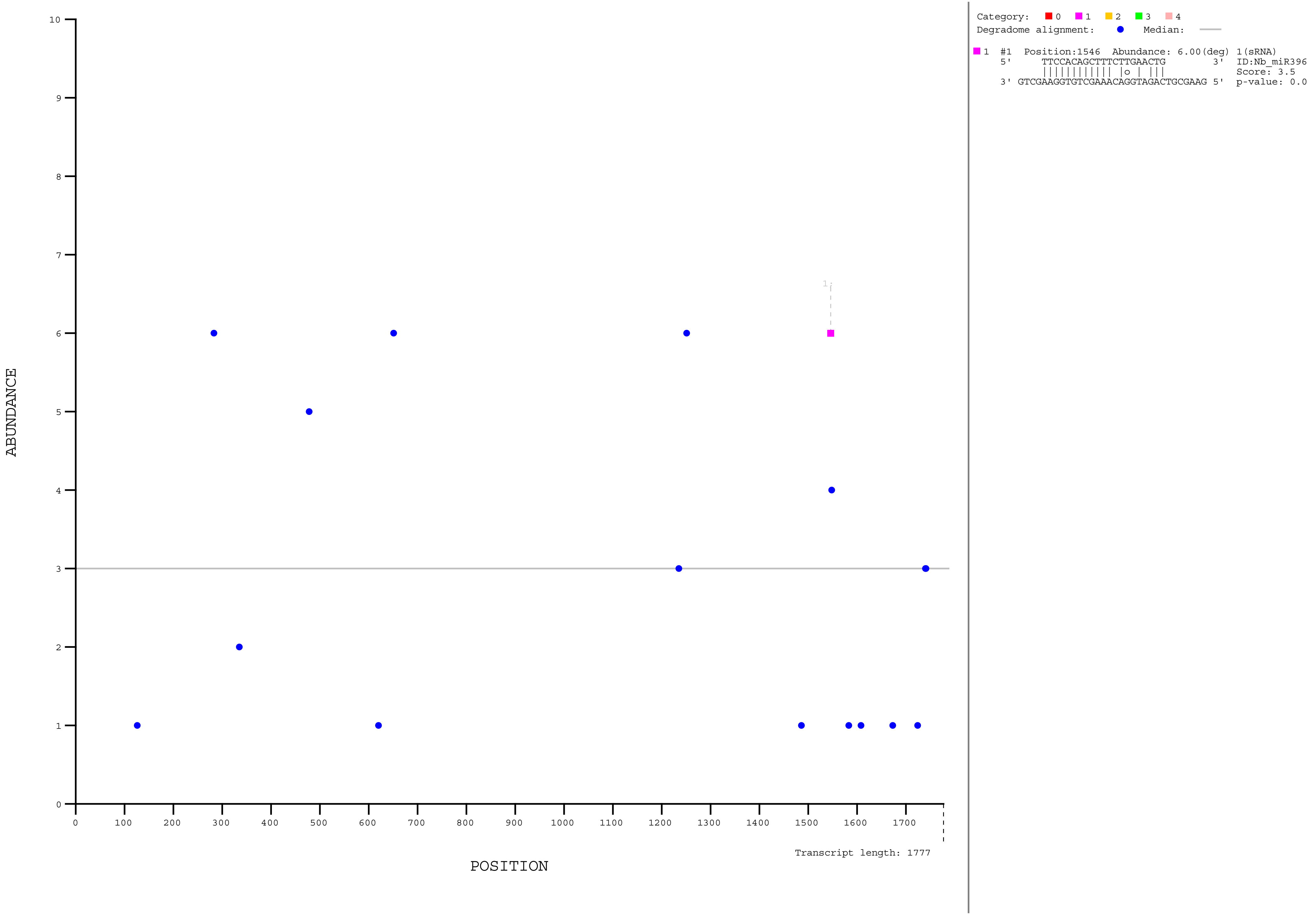

comp77334_c1_seq28 - Growth-regulating factor 5

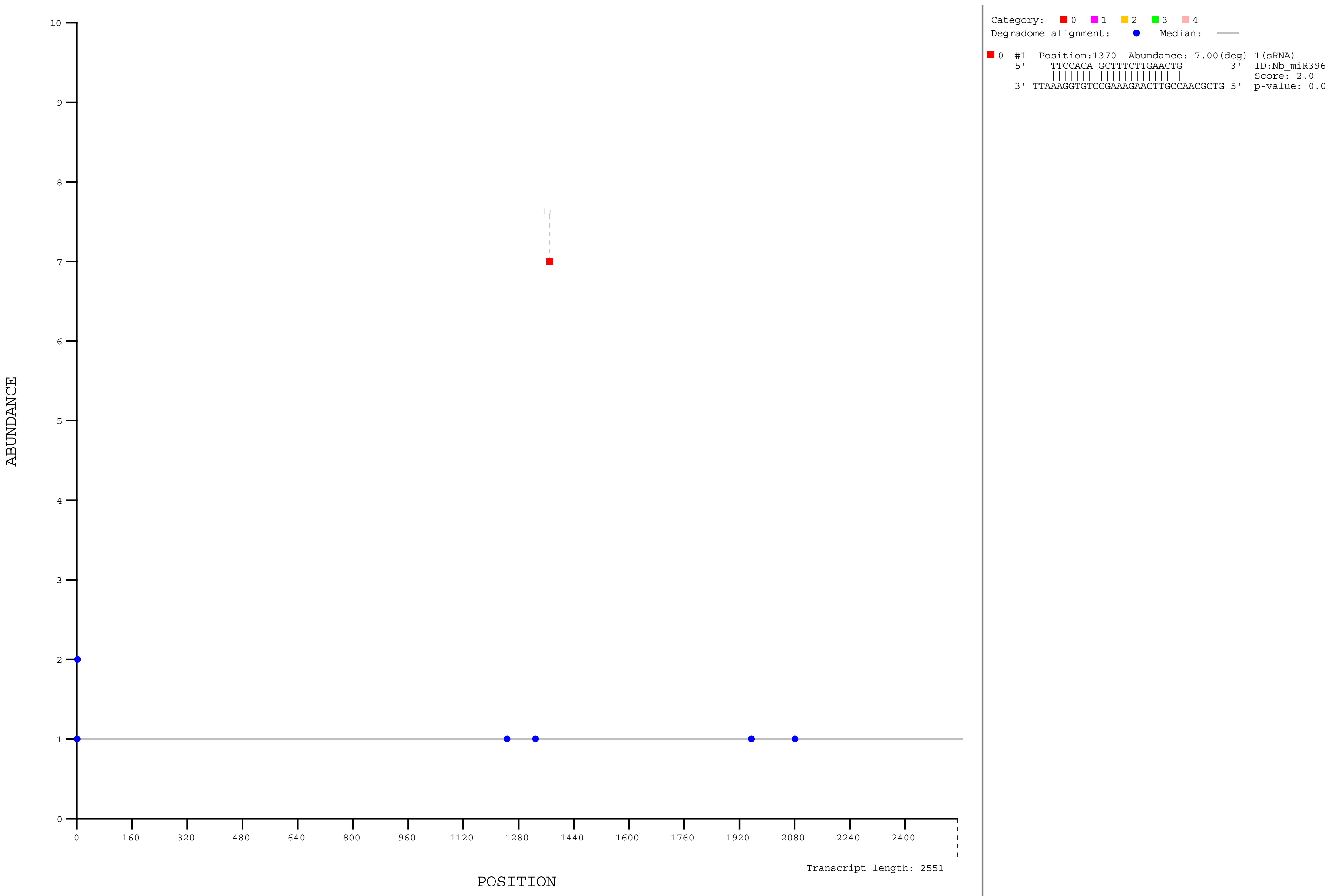

comp79406_c0_seq1 - no_annotation

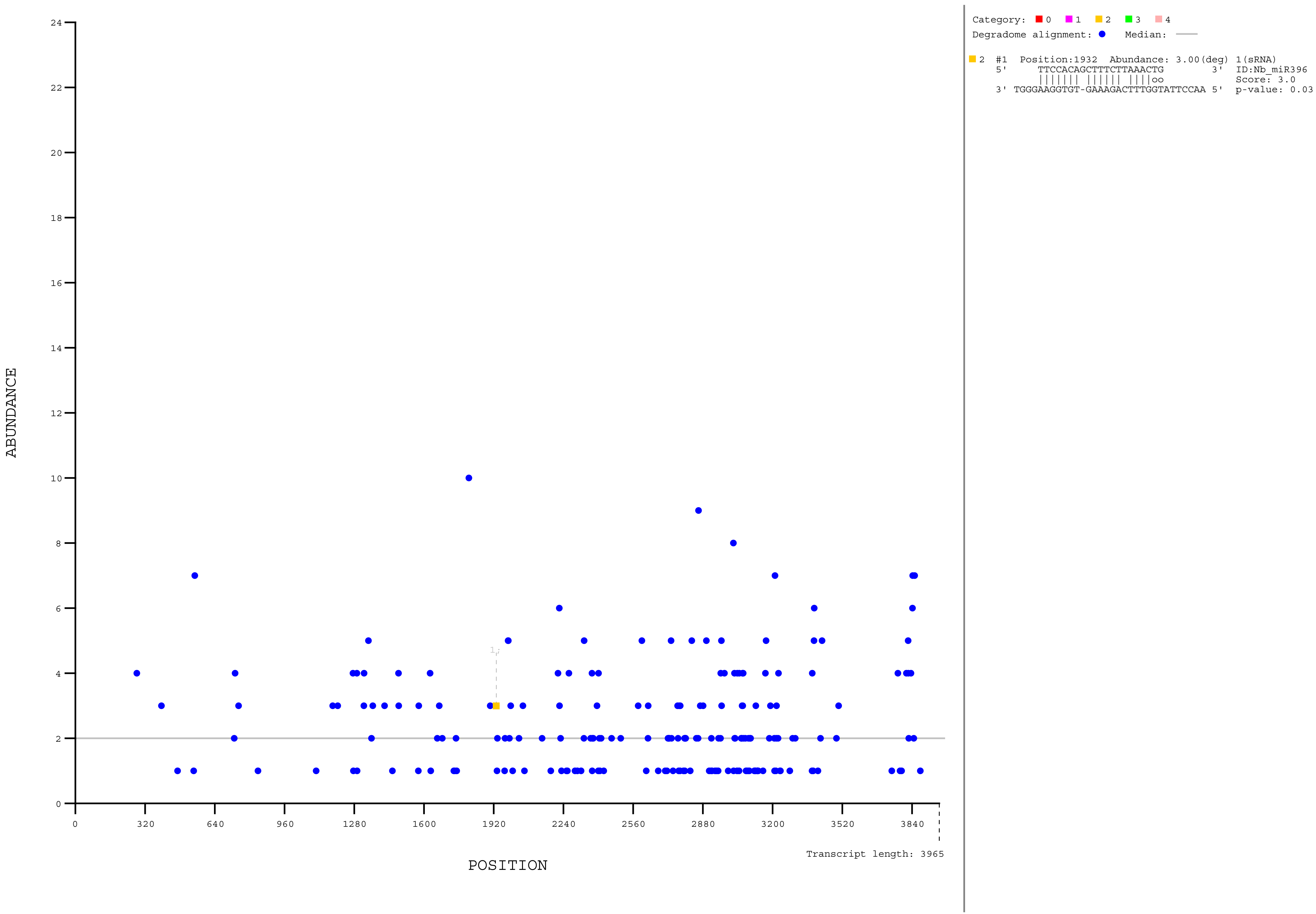

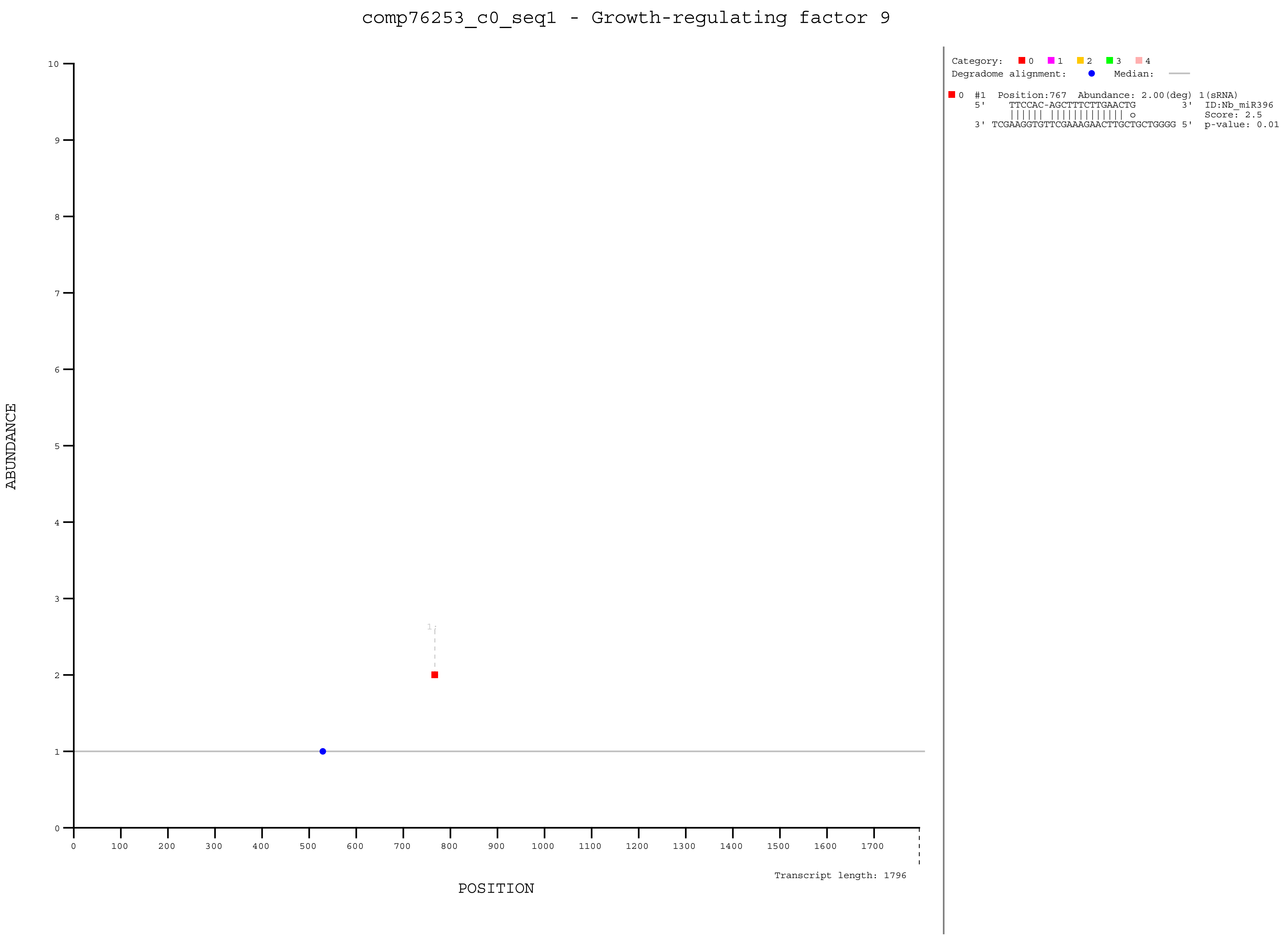
comp76253_c0_seq1 - Growth-regulating factor 9
ABUNDANCE
POSITION
Transcript length: 1796
Category: 0 1 2 3 4
Degradome alignment:
Median:
0 #1 Position:767 Abundance: 2.00(deg) 1(sRNA)
5' TTCCAC-AGCTTTCTTGAACTG 3' ID:Nb_miR396
Score: 2.5
3' TCGAAGGTGTTCGAAAGAACTTGCTGCTGGGG 5' p-value: 0.01

comp69128_c0_seq2 - DNA-directed RNA polymerase V subunit 5A-like

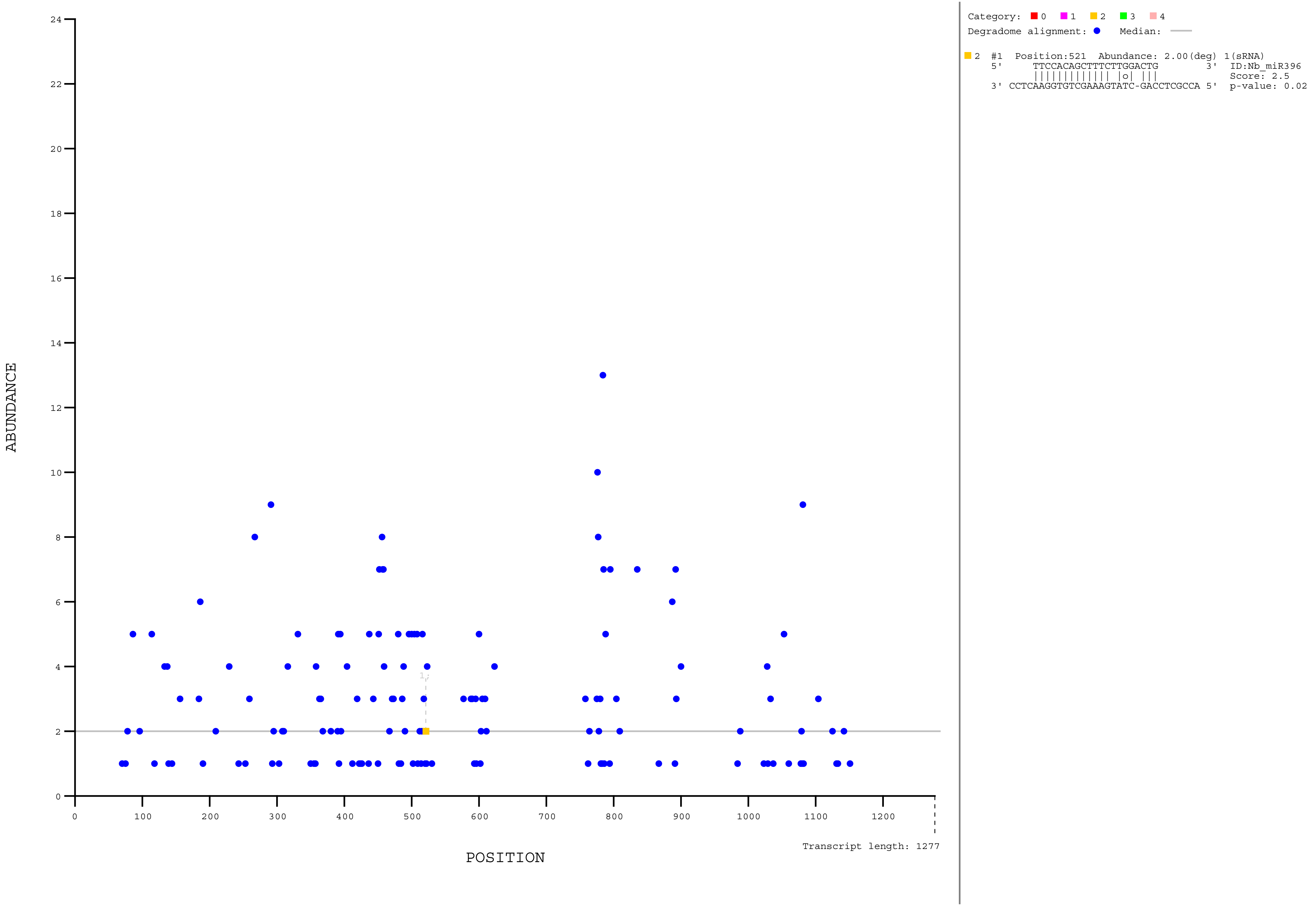

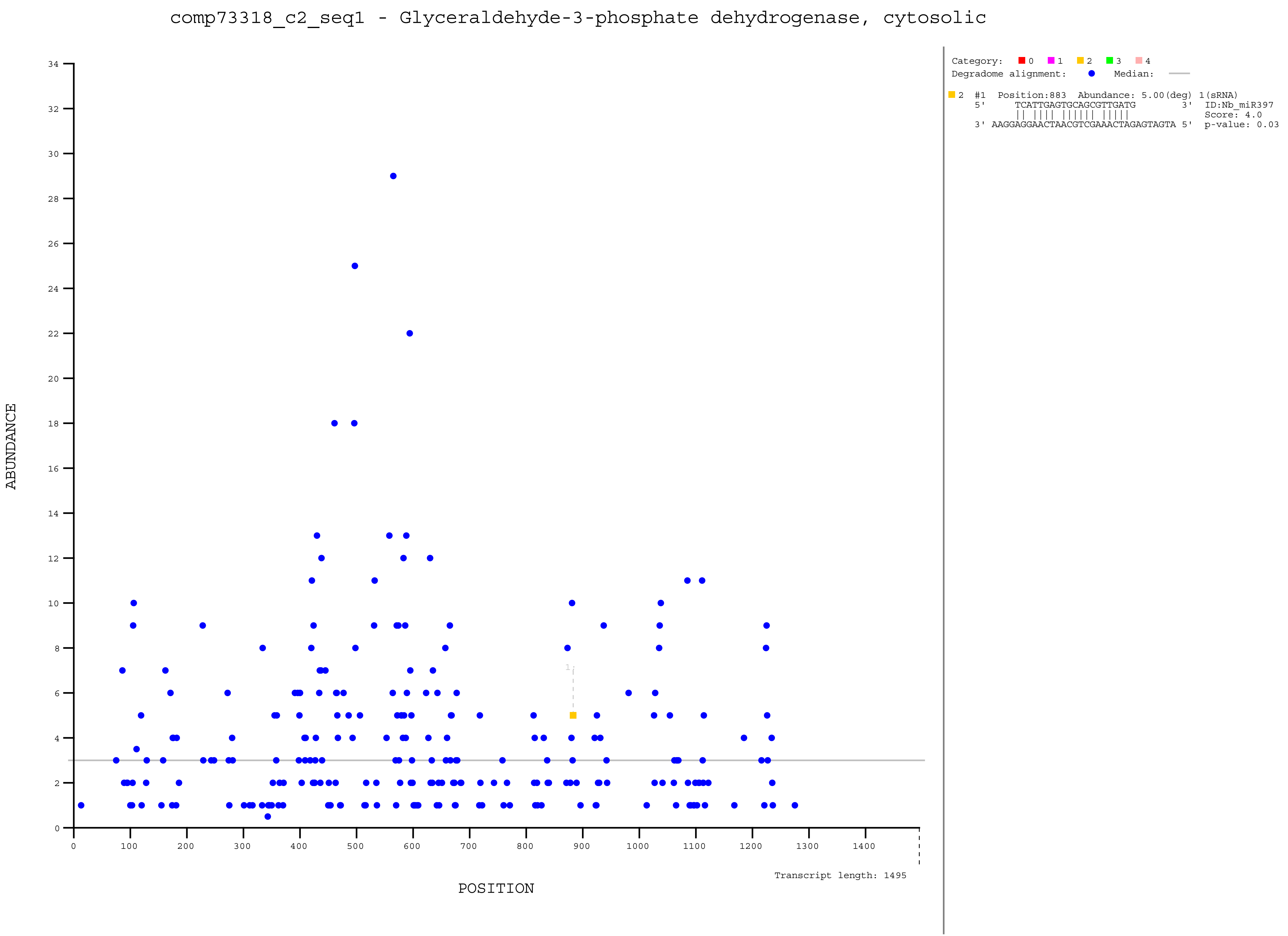
comp73318_c2_seq1 - Glyceraldehyde-3-phosphate dehydrogenase, cytosolic
ABUNDANCE
POSITION
Transcript length: 1495
Category: 0 1 2 3 4
Degradome alignment:
Median:
2 #1 Position:883 Abundance: 5.00(deg) 1(sRNA)
5' TCATTGAGTGCAGCGTTGATG 3' ID:Nb_miR397
Score: 4.0
3' AAGGAGGAACTAACGTCGAAACTAGAGTAGTA 5' p-value: 0.03

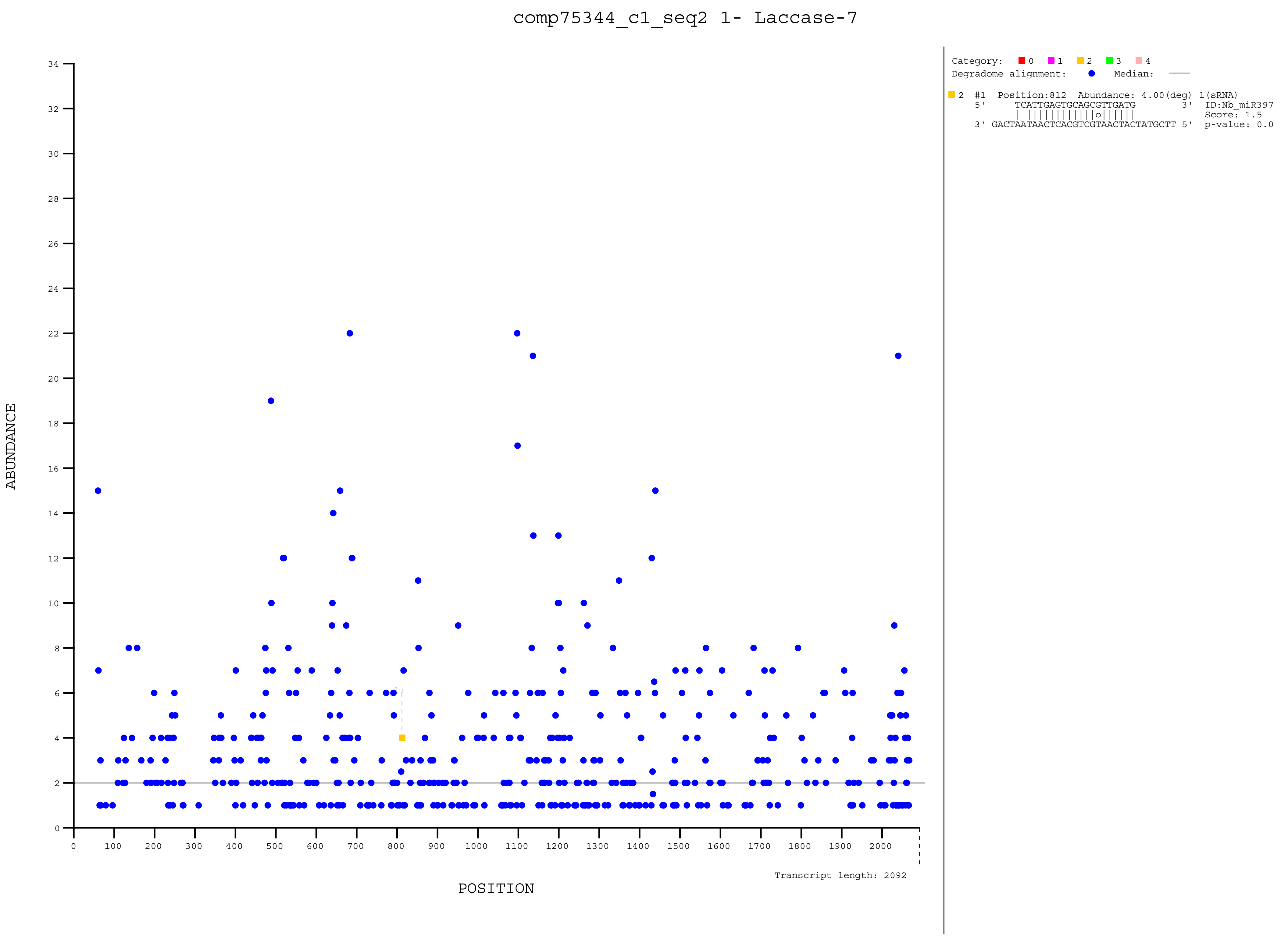
comp75344_c1_seq2 1- Laccase-7
ABUNDANCE
POSITION
Transcript length: 2092
Category: 0 1 2 3 4
Degradome alignment:
Median:
2 #1 Position:812 Abundance: 4.00(deg) 1(sRNA)
5' TCATTGAGTGCAGCGTTGATG 3' ID:Nb_miR397
| |||||||||||||o|||||| Score: 1.5
3' GACTAATAACTCACGTCGTAACTACTATGCTT 5' p-value: 0.0

comp77652_c0_seq1 - Probable serine/threonine-protein kinase abkC

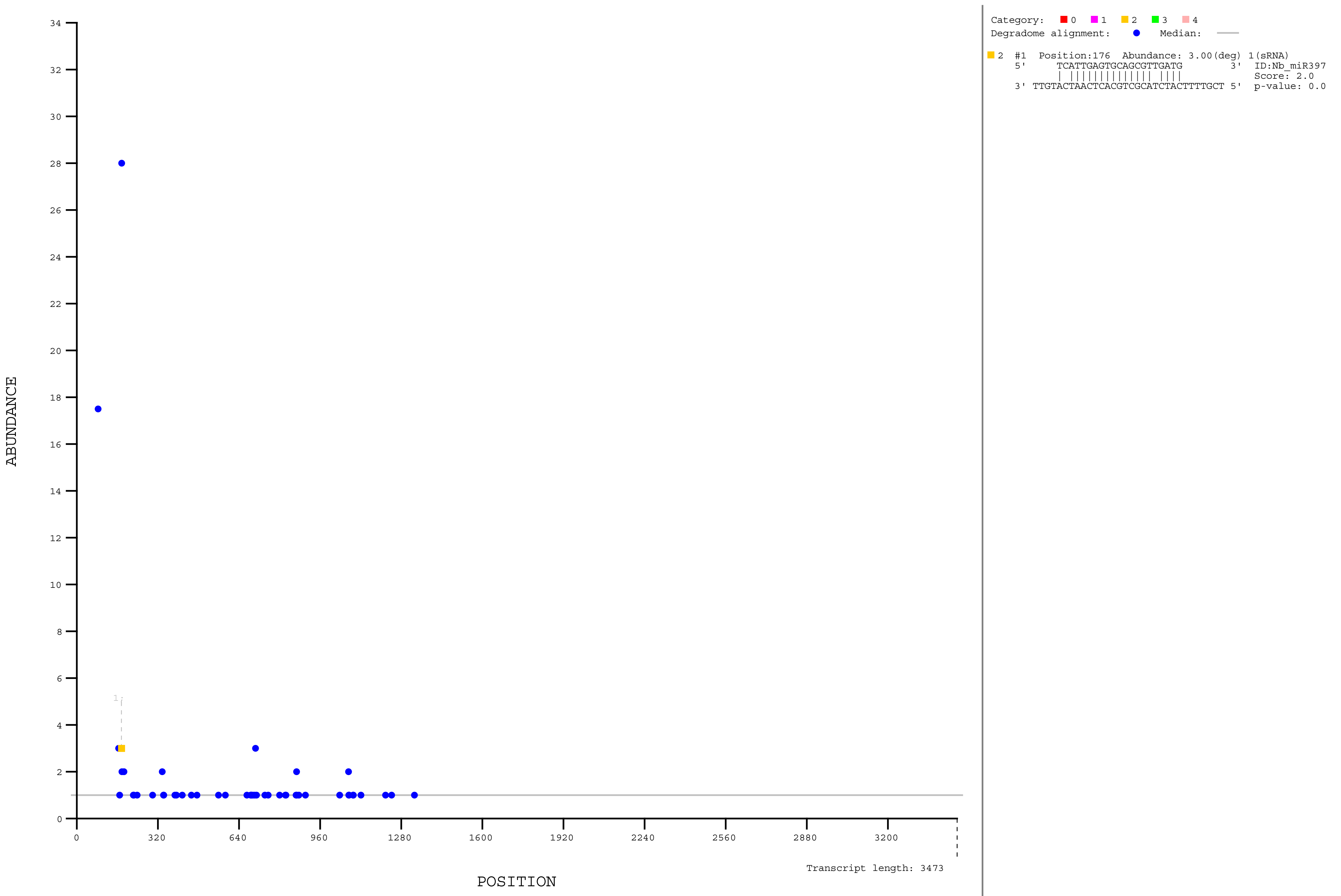

comp100607_c0_seq1 - Umecyanin

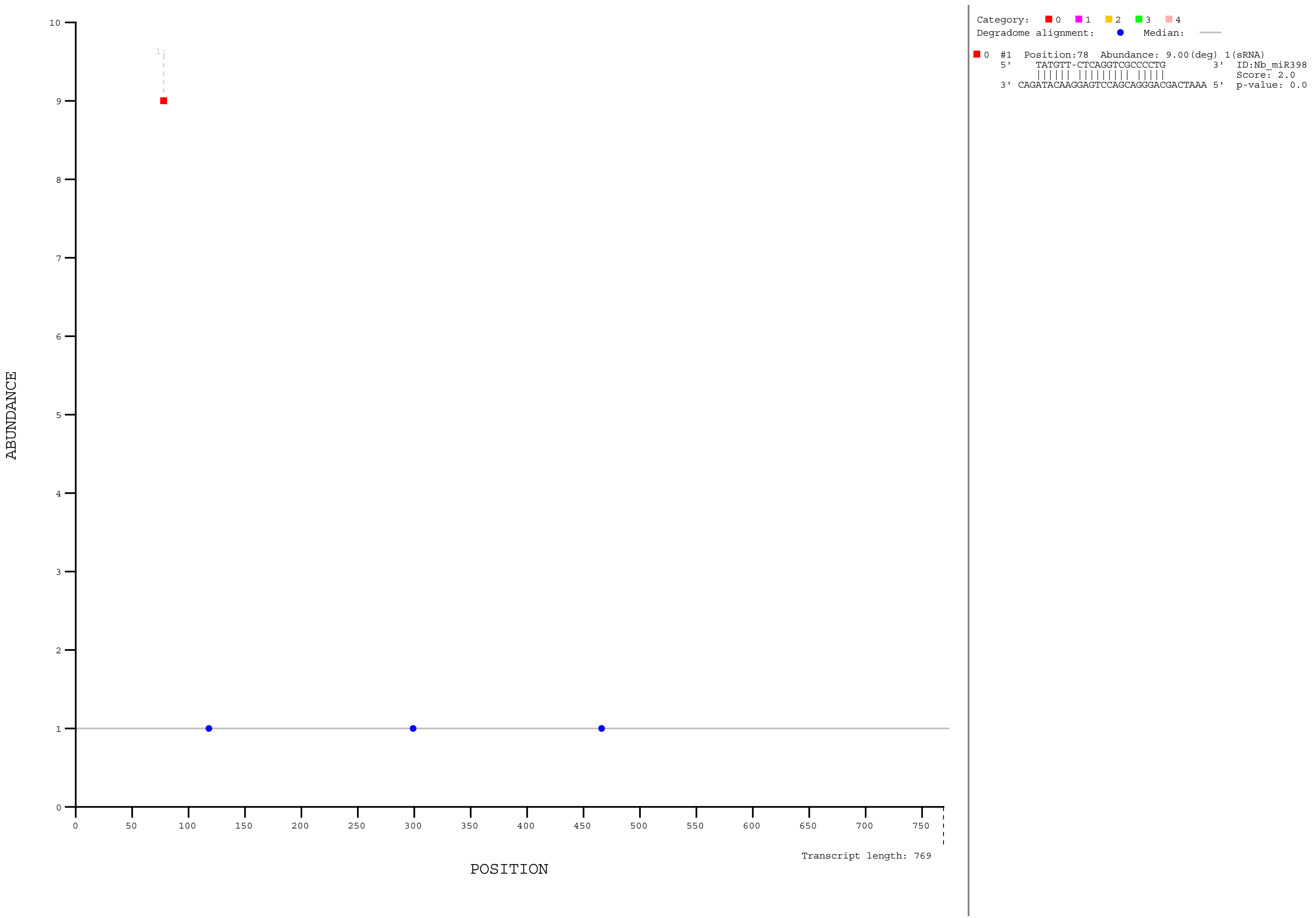

comp85708_c0_seq1 - Uclacyanin-2

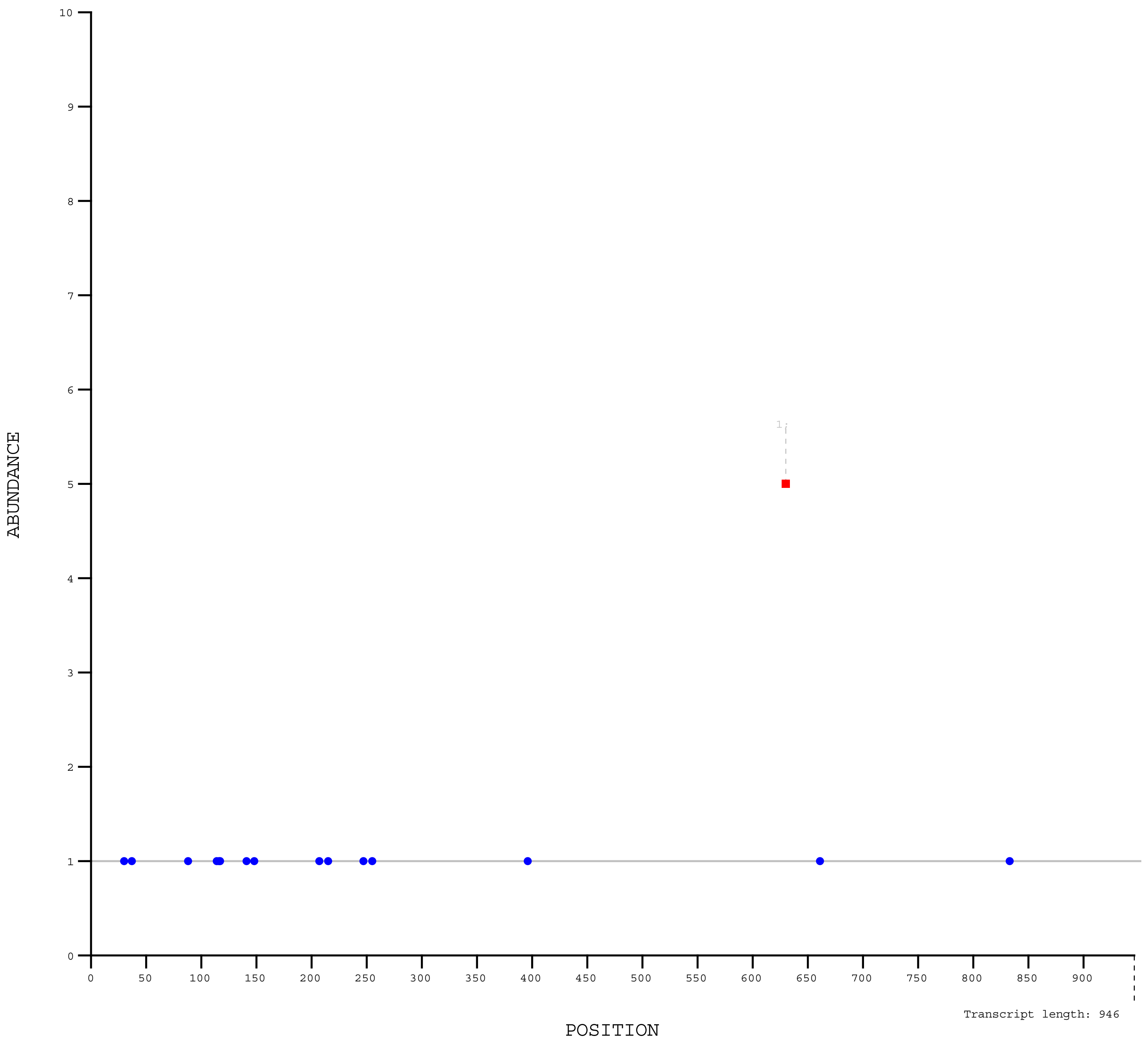


```
Category: ■0 ■1 ■2 ■3 ■4
Degradome alignment: ●   Median: —

■ 0  #1  Position:630  Abundance: 5.00(deg) 1(sRNA)
     5'      TGCACTGCCTCTTCCCTGGCT         3'  ID:Nb_miR408
             ||||||||||||||||o| o              Score: 2.0
     3' TATAACGTGACGGAGAAGGGATCTGGTACTTA 5'  p-value: 0.0
```

comp79061_c1_seq3 -Copper-transporting ATPase PAA2, chloroplastic

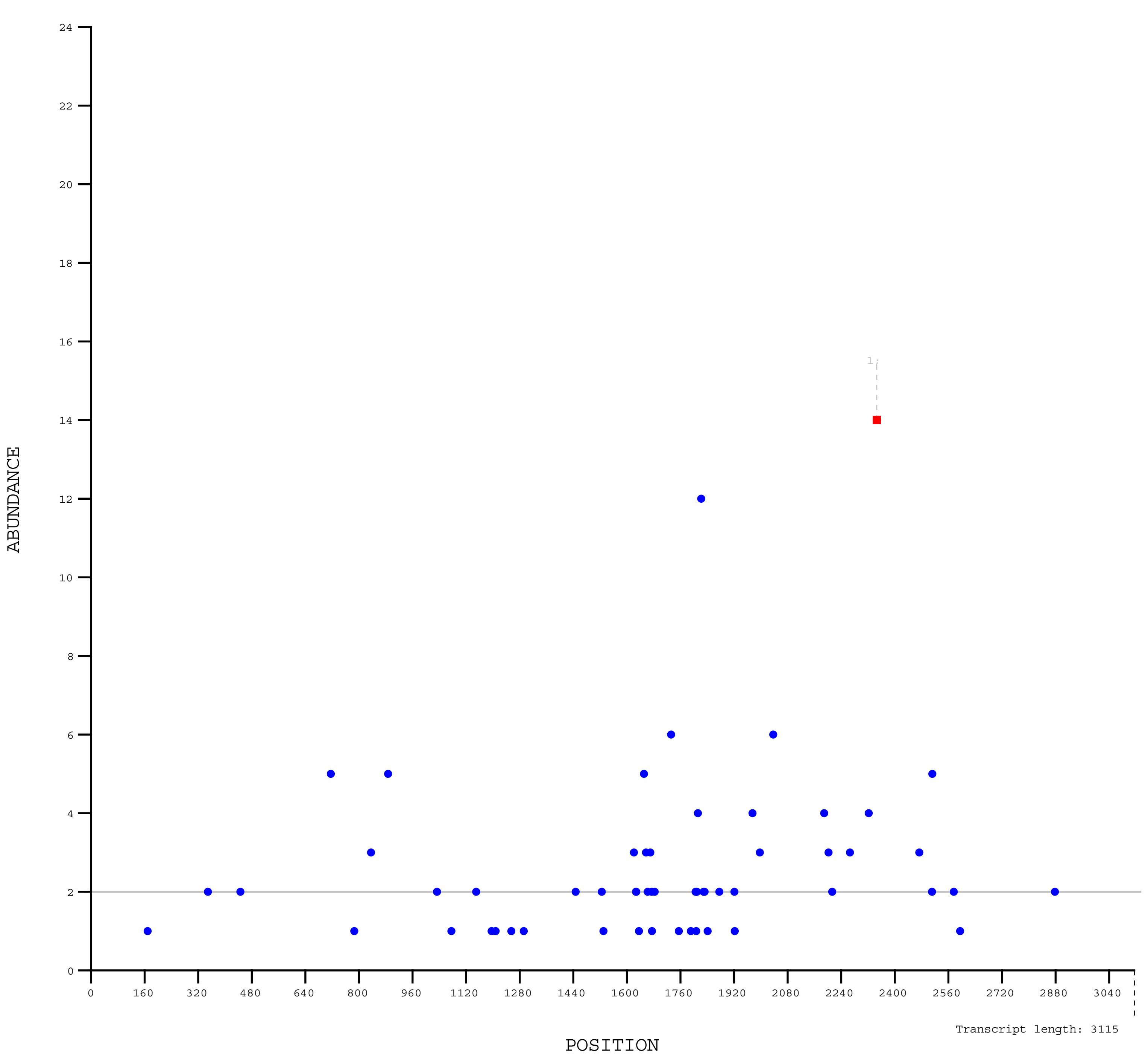

Category: 0 1 2 3 4
Degradome alignment: Median:

```
0  #1  Position:2346  Abundance: 14.00(deg) 1(sRNA)
   5'    TGC-ACTGCCTCTTCCCTGGCT        3'  ID:Nb_miR408
          ||| ||||||||||||||| ||           Score: 2.0
   3' TCAACGTTGACGGAGAAGGGACAGAGGGCTAT 5'  p-value: 0.0
```

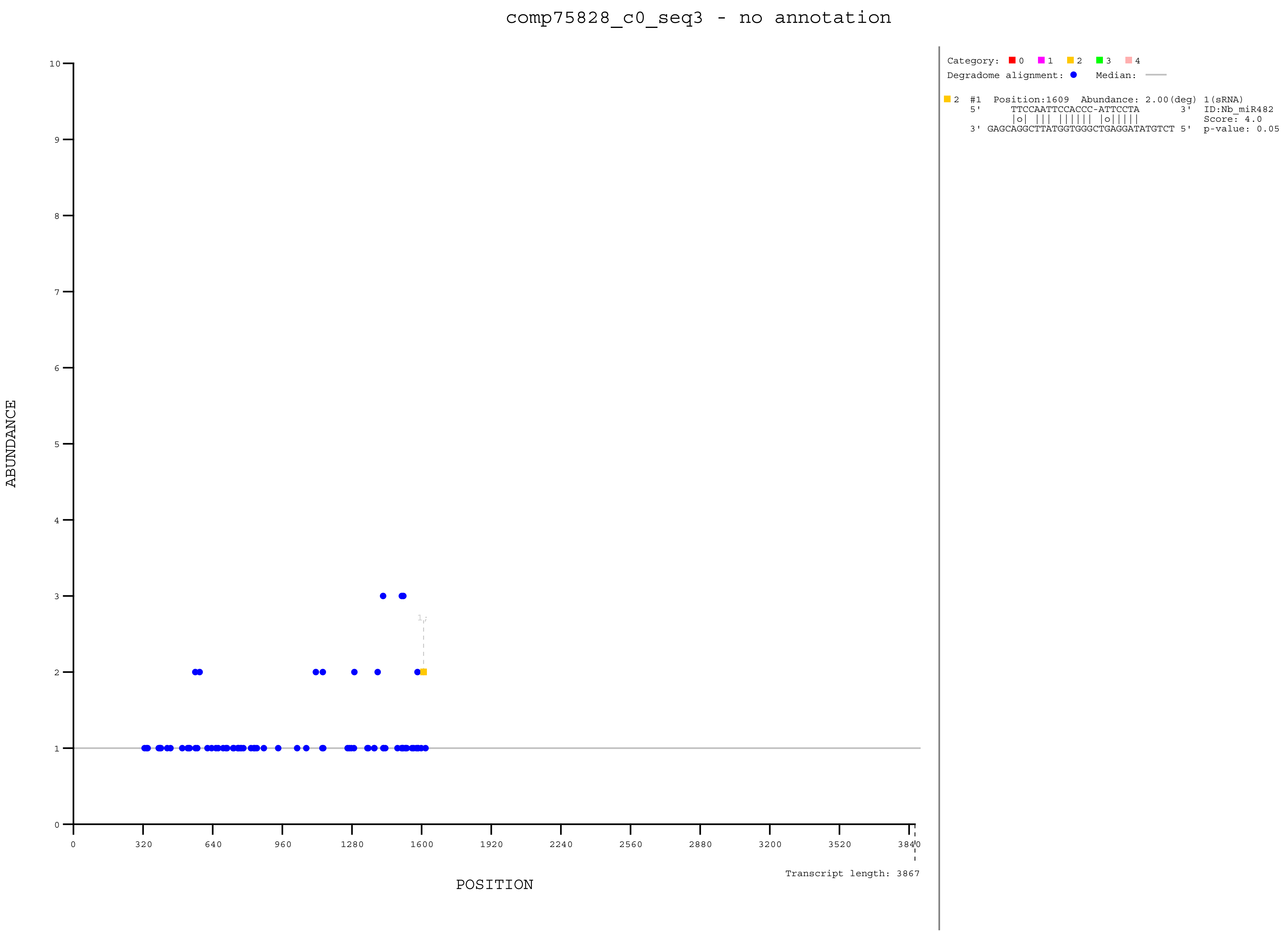
comp75828_c0_seq3 - no annotation
ABUNDANCE
10
9
8
7
6
5
4
3
2
1
0
0
320
640
960
1280
1600
1920
2240
2560
2880
3200
3520
3840
1
Transcript length: 3867
POSITION
Category: 0 1 2 3 4
Degradome alignment: Median:
2 #1 Position:1609 Abundance: 2.00(deg) 1(sRNA)
5' TTCCAATTCCACCC-ATTCCTA 3' ID:Nb_miR482
|o| ||| |||||| |o||||| Score: 4.0
3' GAGCAGGCTTATGGTGGGCTGAGGATATGTCT 5' p-value: 0.05

comp75426_c0_seq1 - UPF0496 protein 4

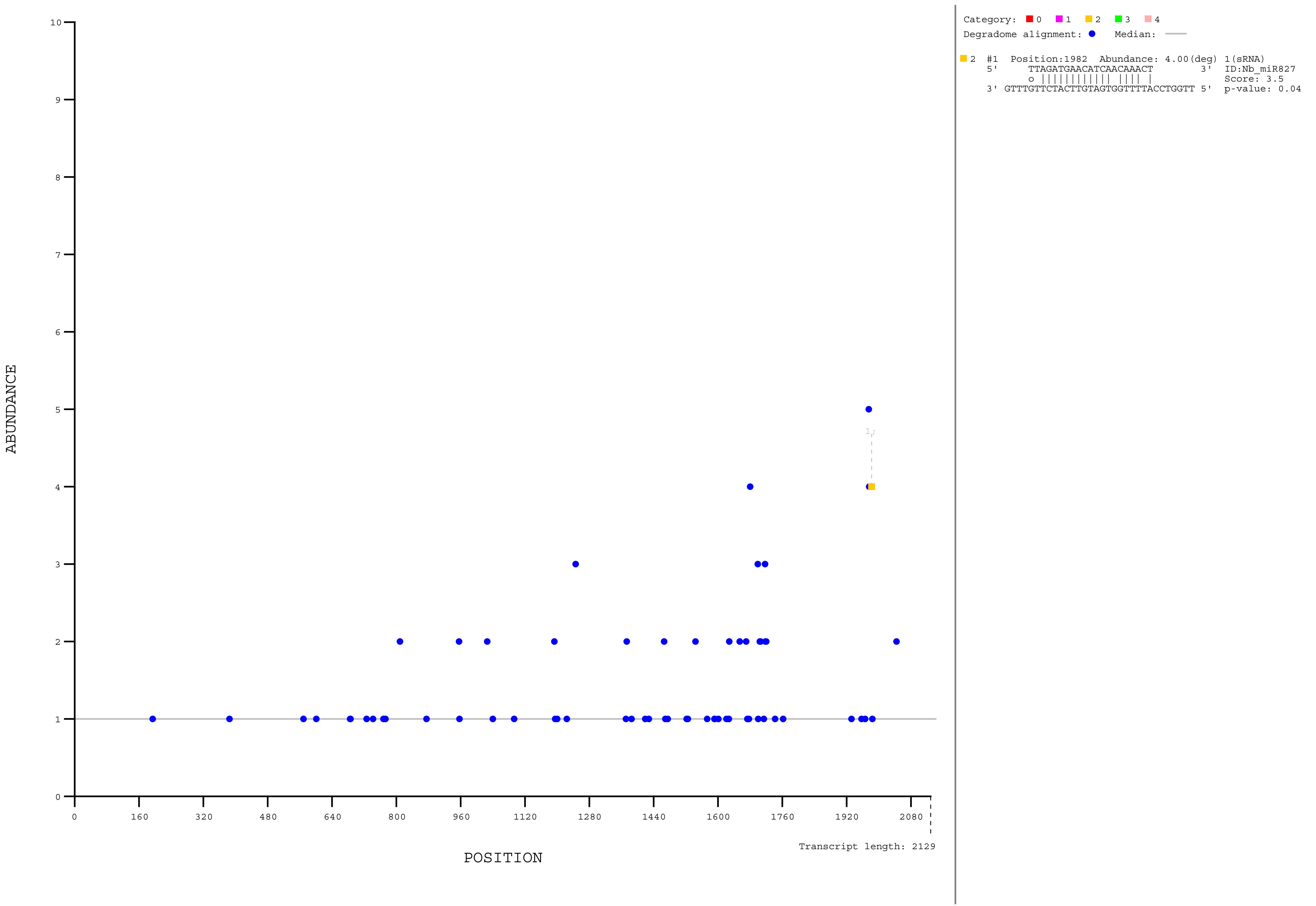

comp79500_c0_seq4 - Calcium-transporting ATPase 8, plasma membrane-type

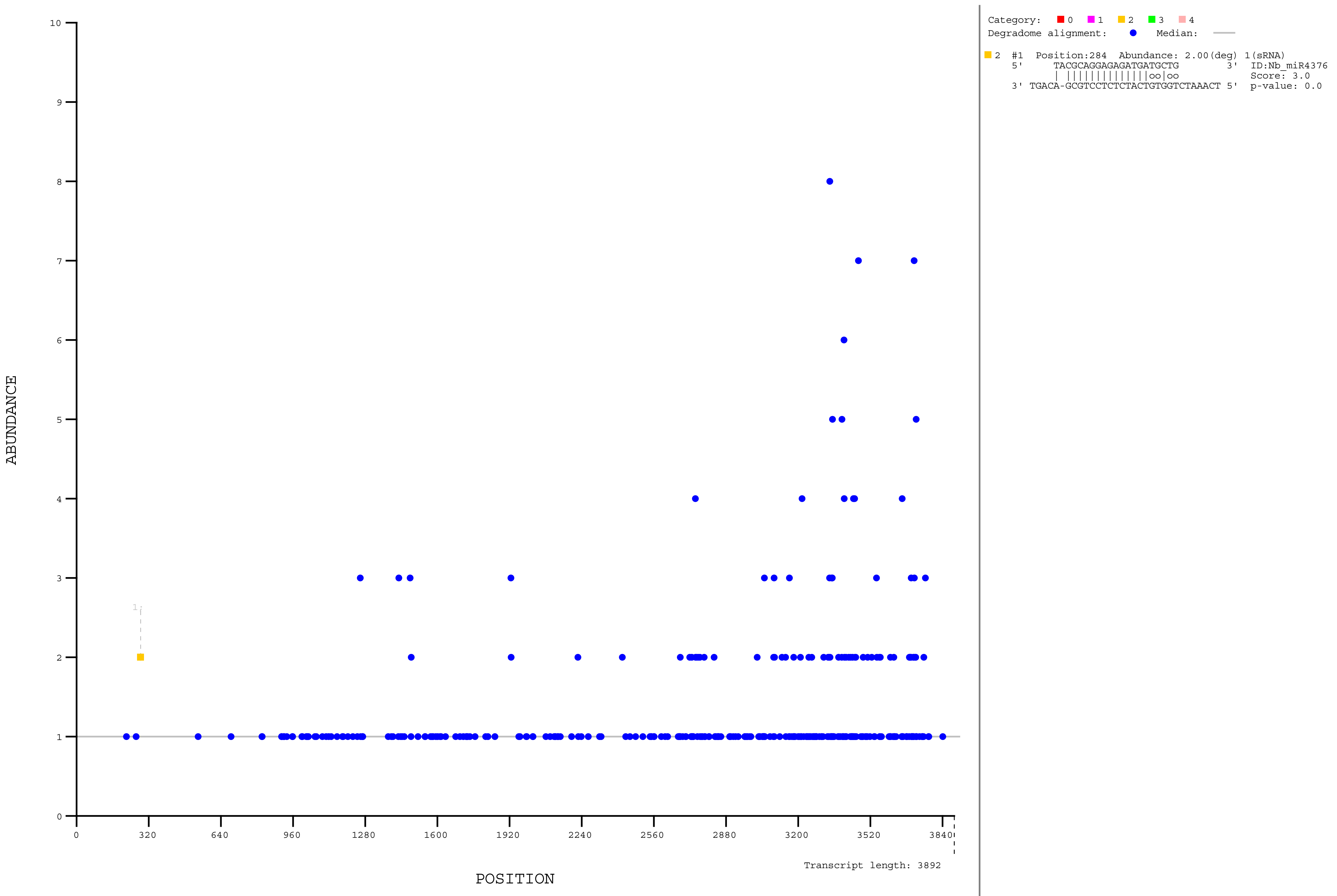

comp70698_c0_seq1 - GPN-loop GTPase 3 homolog

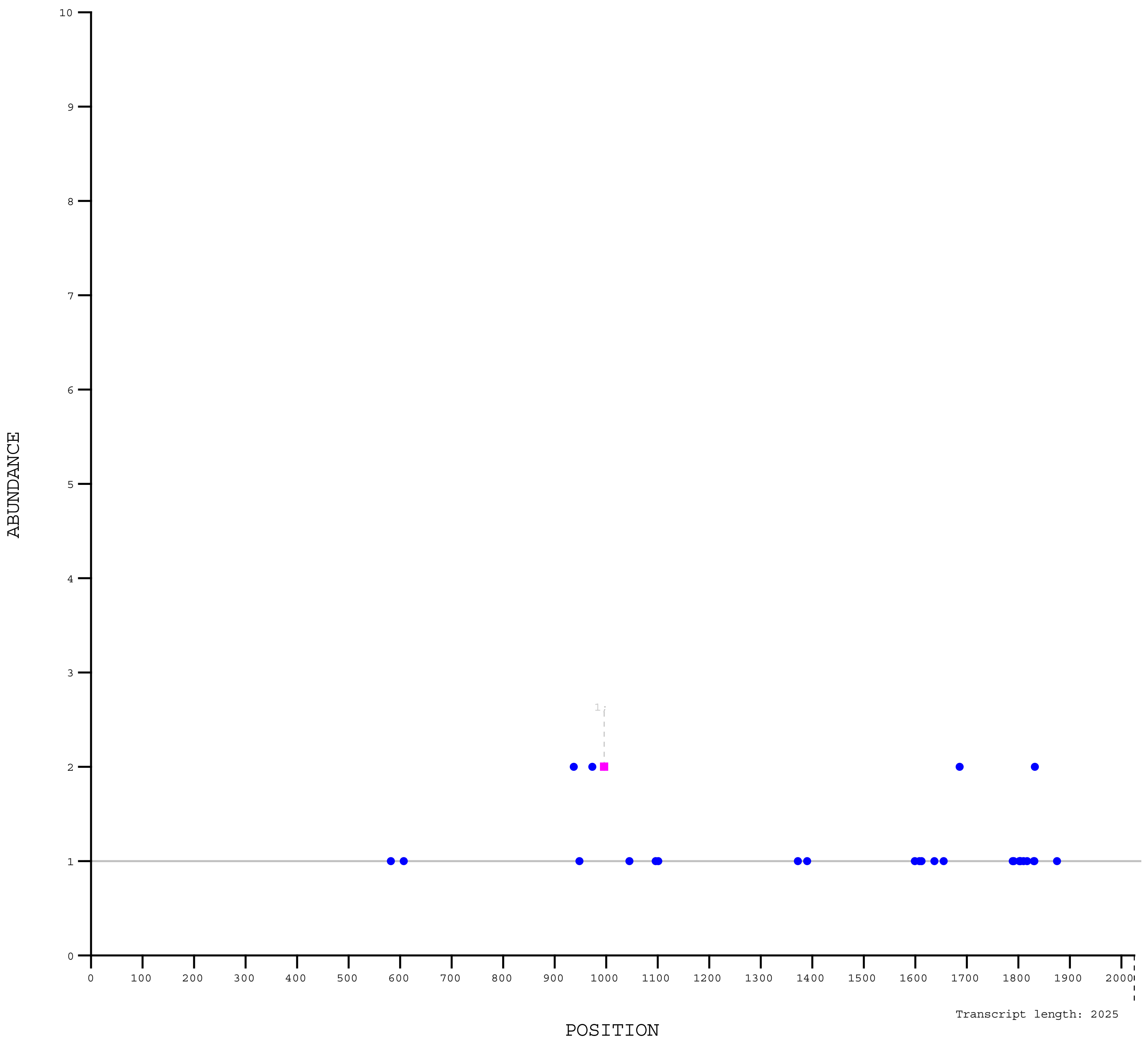


```
Category:  0  1  2  3  4
Degradome alignment:      Median:

1  #1  Position:996  Abundance: 2.00(deg) 1(sRNA)
   5'     AAATGTTCTTCGAGTATCTTC        3'  ID:Nb_miR6020
          |||| ||||||o|| || |||            Score: 3.5
   3' TAGGTTTAAAAGAAGTTC-TACAAGGTACGTT 5'  p-value: 0.01
```

comp74553_c0_seq5 - no_annotation

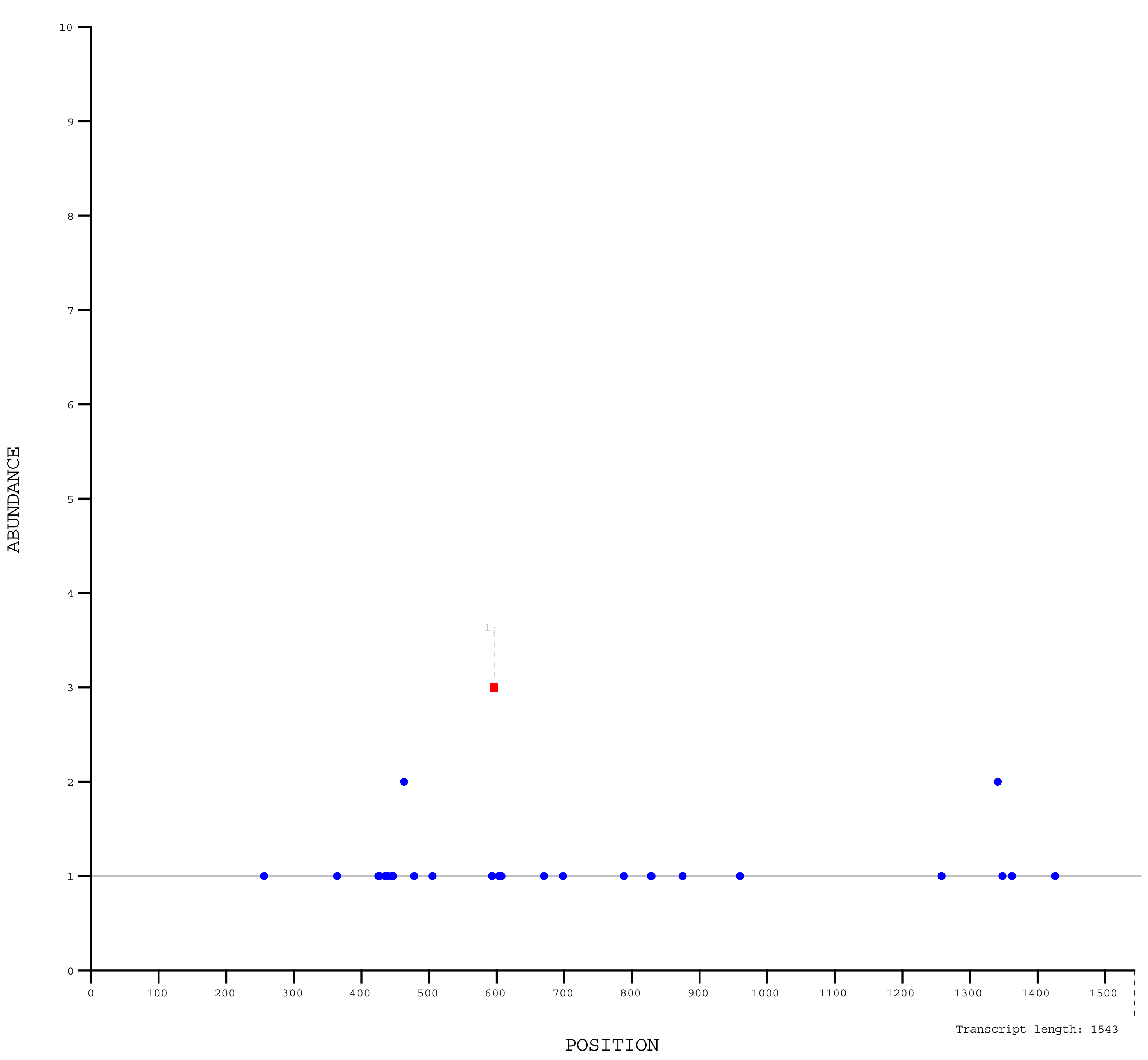

Category: 0 1 2 3 4
Degradome alignment: Median:

```
0  #1  Position:596  Abundance: 3.00(deg) 1(sRNA)
   5'     AAATGTTCTTCGAGTATCTTC        3'  ID:Nb_miR6020
           | || ||||||o||| ||o||           Score: 4.0
   3' TGGTTCTAGAAGAAGTTCAAAGGAGAGTTTCA 5'  p-value: 0.01
```

comp75715_c0_seq5 - Probable methylenetetrahydrofolate reductase

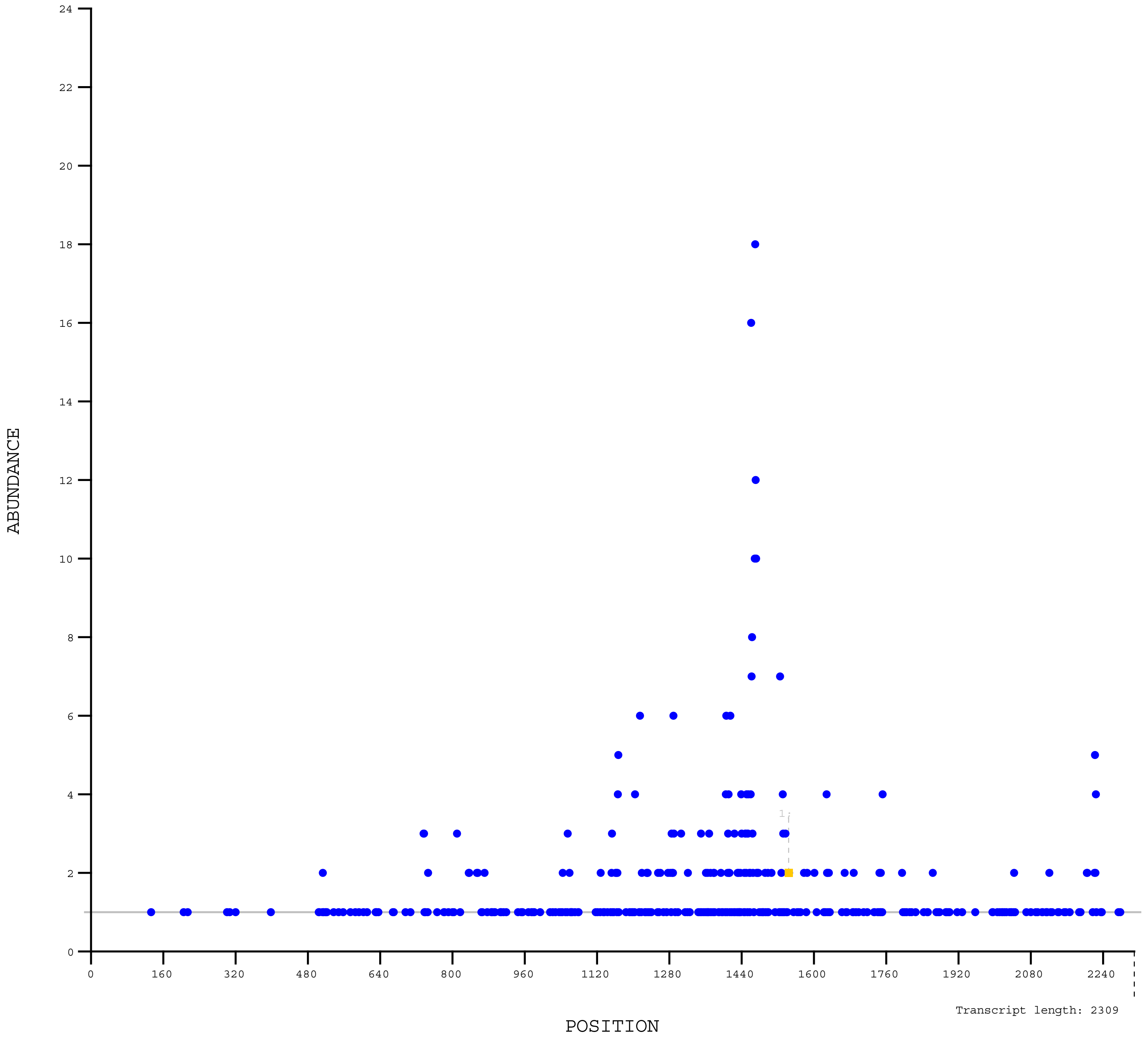


```
Category:  0  1  2  3  4
Degradome alignment:      Median:

2  #1  Position:1544  Abundance: 2.00(deg) 1(sRNA)
   5'     AAATGTTCTTCGAGTATCTTC        3'  ID:Nb_miR6020
          ||||||||| ||||  ||||||           Score: 3.0
   3' TGGGTTTACAAGTAGCTACTAGAAGCAGAGAC 5'  p-value: 0.02
```

comp80883_c0_seq1 - Tubulin alpha chain

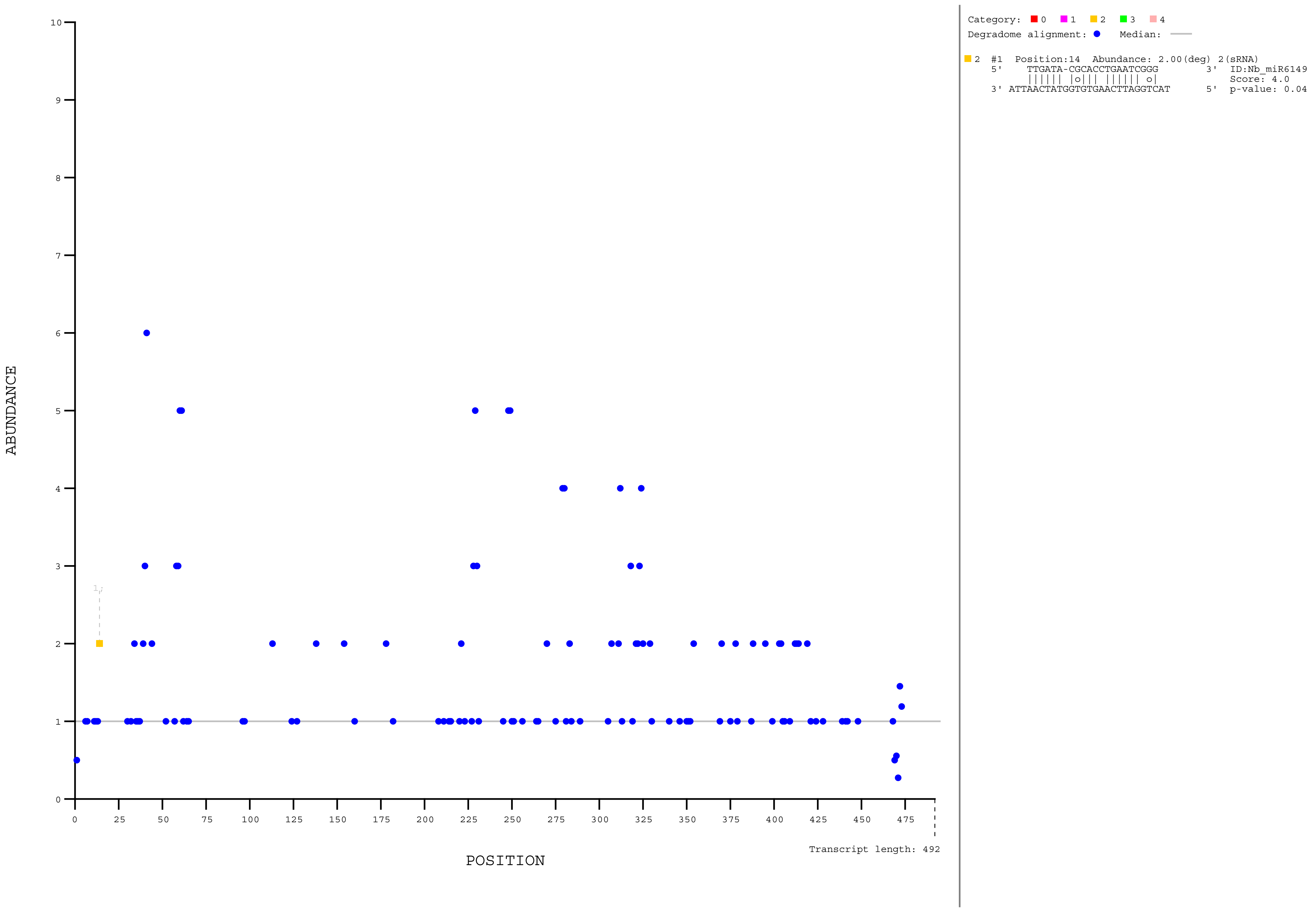

comp75221_c0_seq2 - Serine/threonine-protein kinase HT1

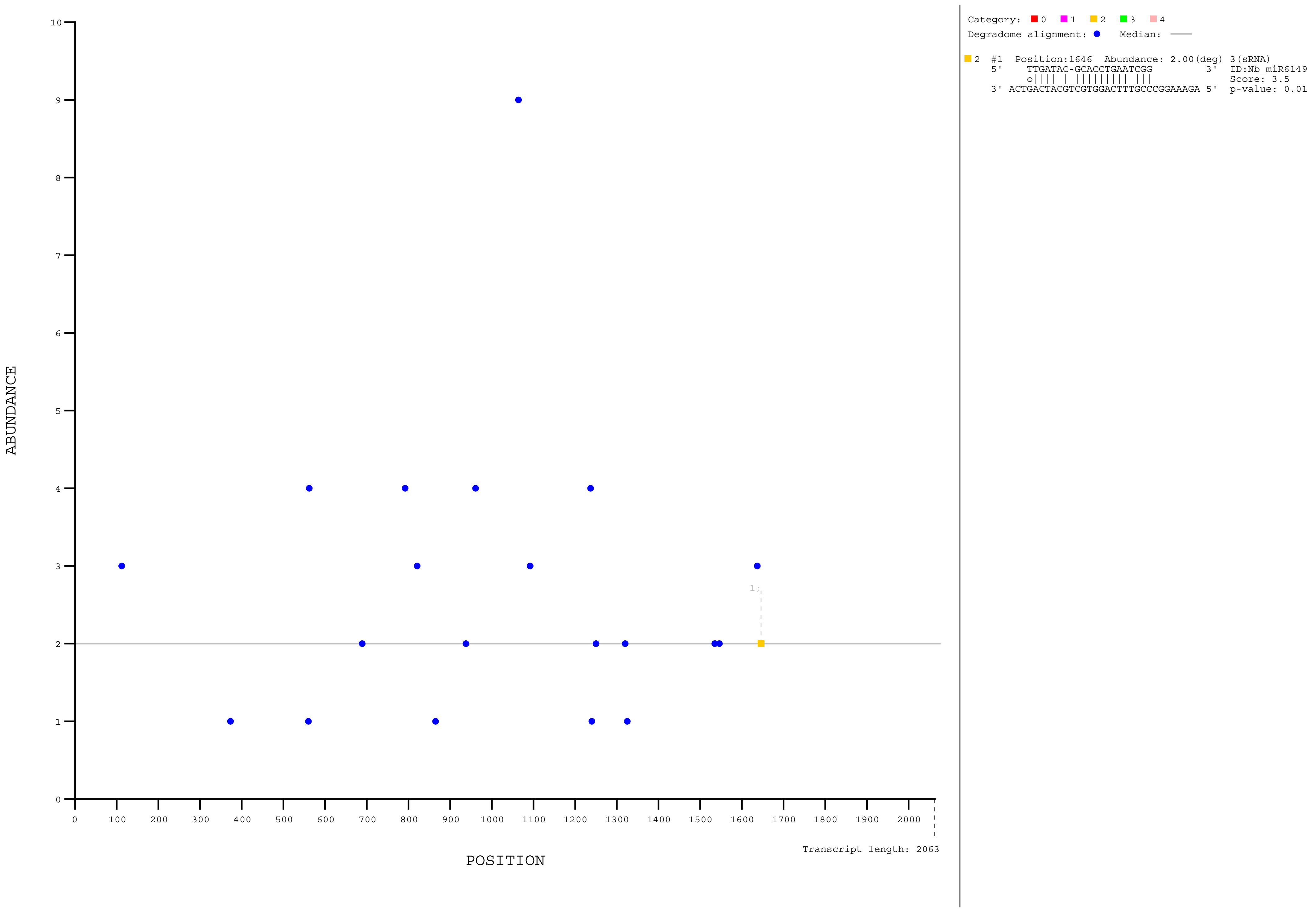

comp84169_c0_seq1 - Probable carboxylesterase 8

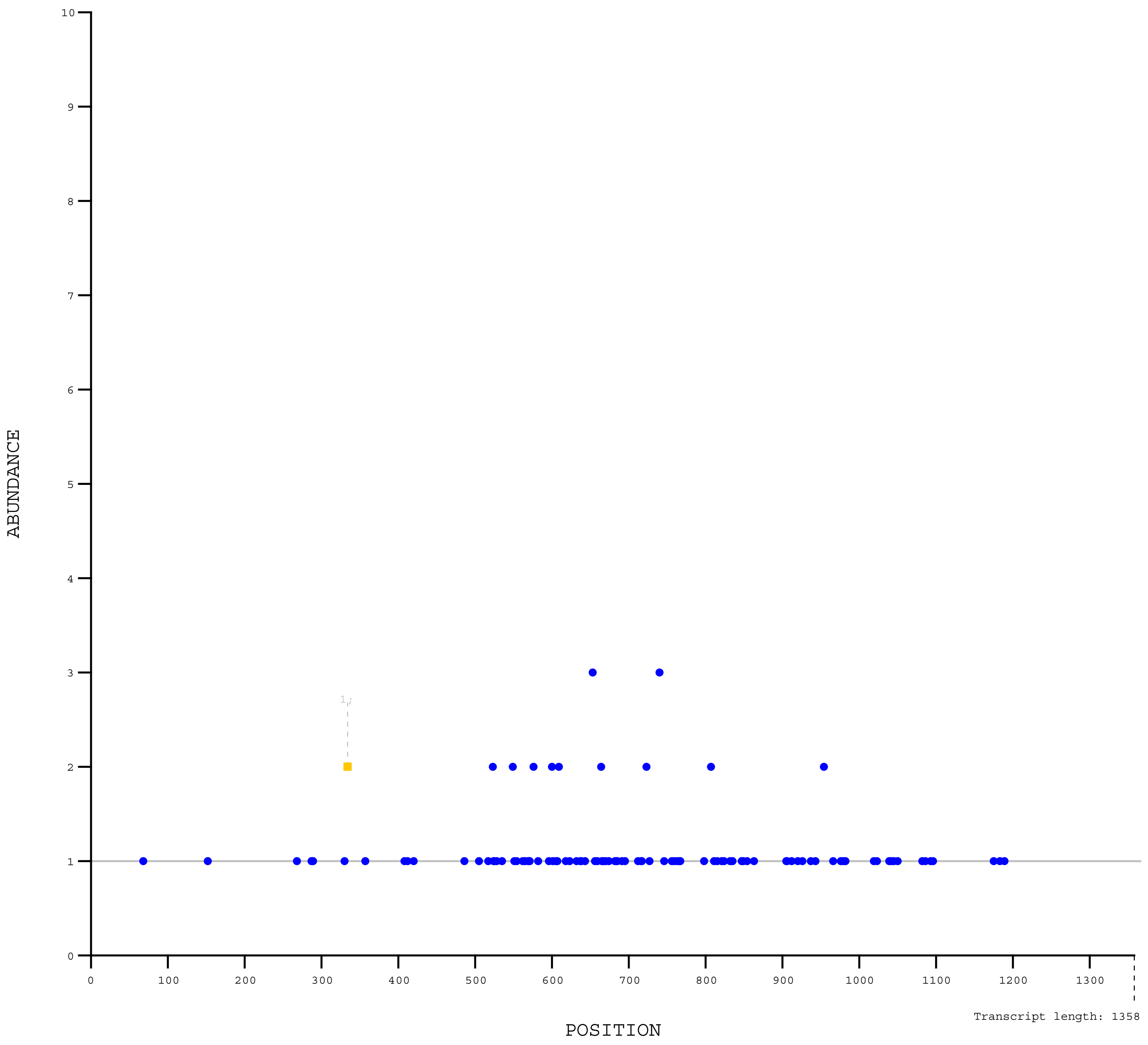

Category: ■ 0 ■ 1 ■ 2 ■ 3 ■ 4
Degradome alignment: ● Median: —

```
■ 2  #1  Position:334  Abundance: 2.00(deg) 3(sRNA)
     5'      TGAGTGTGAGGCGTTGGATTGA       3'  ID:Nb_miR6151
             | ||||| ||||| ||||||o|           Score: 3.5
     3' ATTAAATCACAATCCGCTACCTAATTGCCCGG 5'  p-value: 0.0
```

comp66224_c0_seq2 - Ras-related protein RABA2b

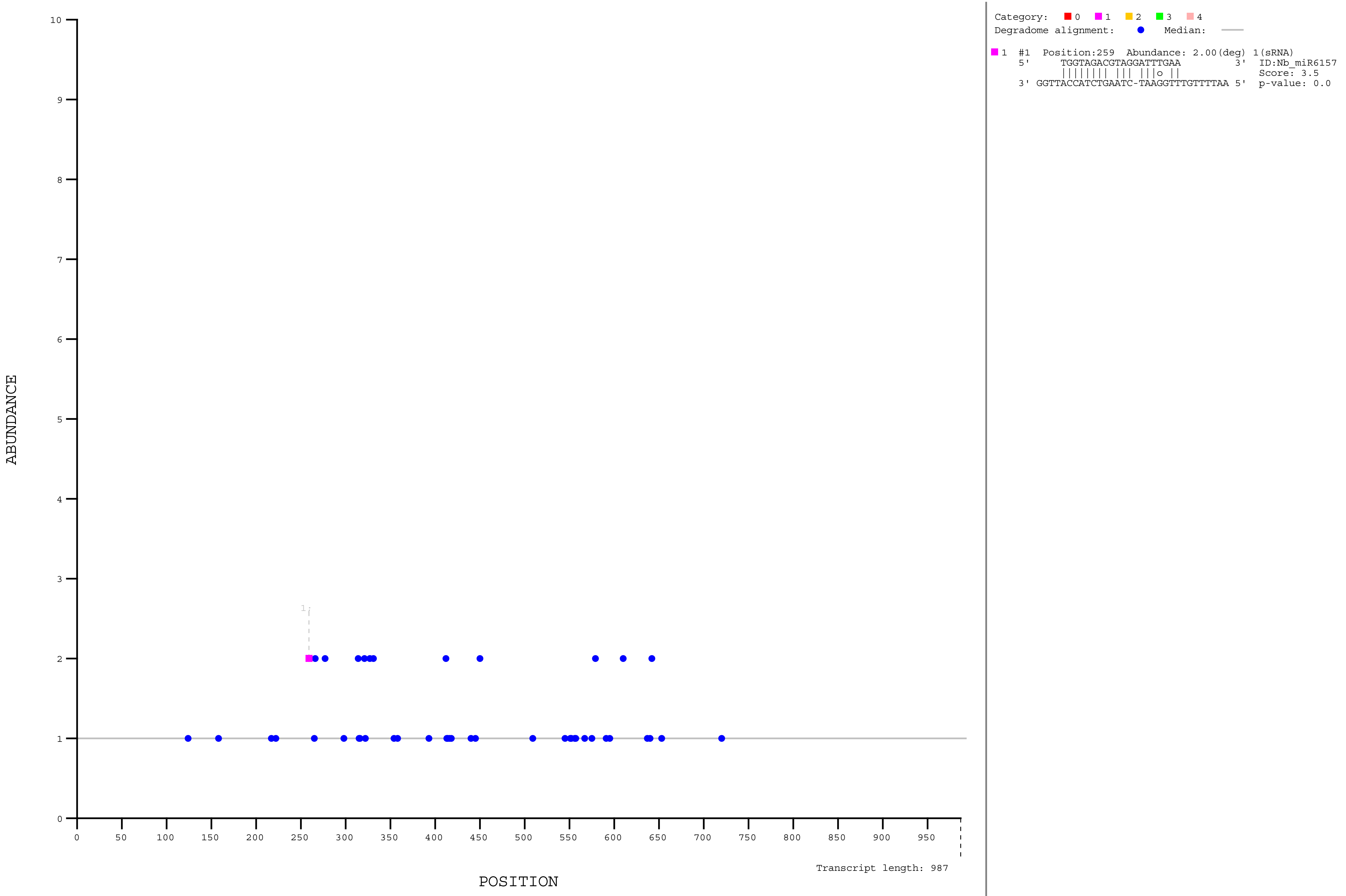

comp74264_c0_seq1 - 60S ribosomal protein L5

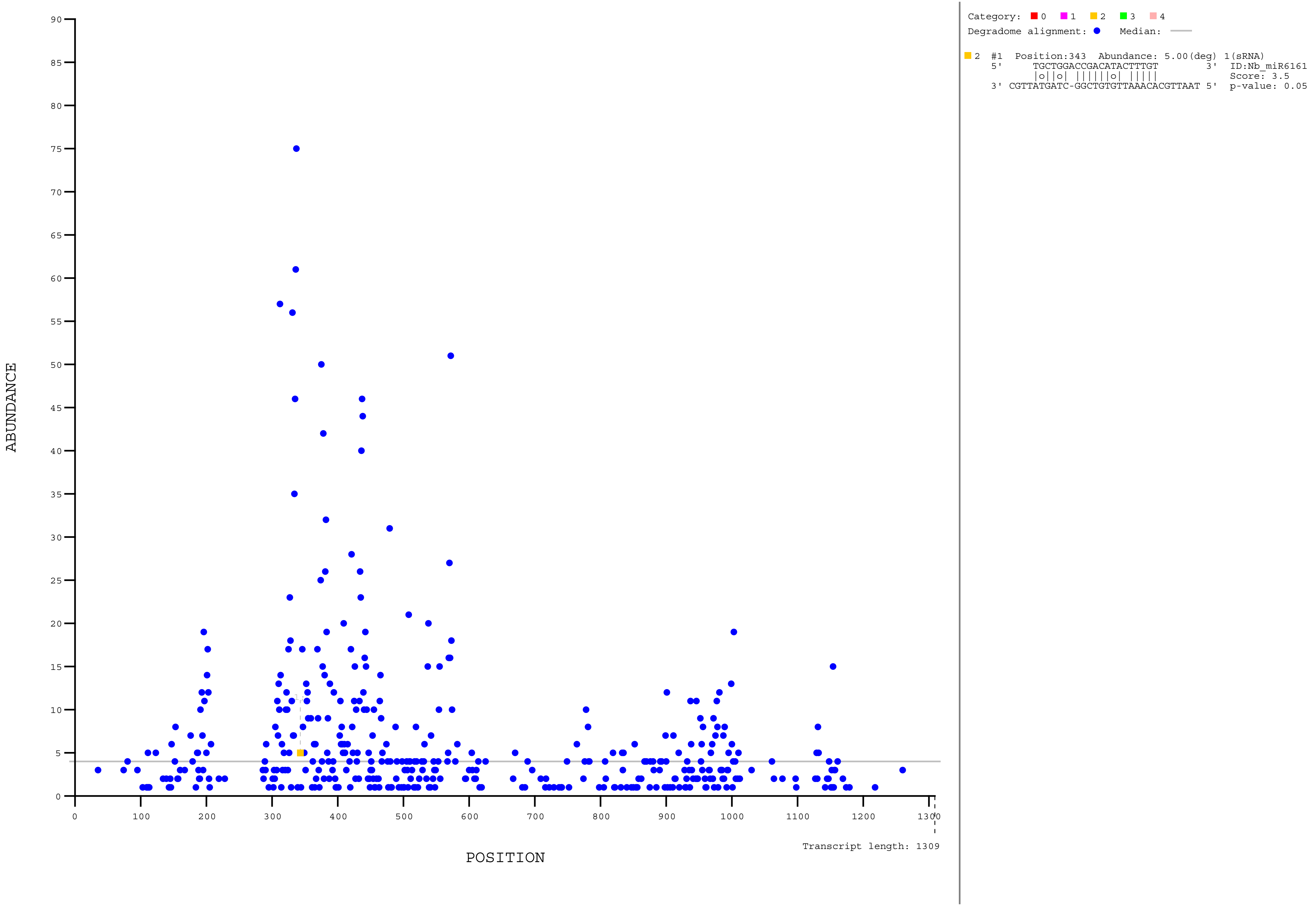

Supplement: Additional file 3: Figure S2. — Target plots (t-plots) of conserved and other known miRNA targets confirmed by degradome sequencing. In the head of the pictures there is a unique identifier of the mRNA followed by the annotation of the transcript. The solid lines and dot in miRNA: mRNA alignments indicate matched RNA base pairs and GU mismatch, respectively. The relative abundances are plotted against the nucleotide position within the transcript. (PDF 377 kb) [file 12864_2015_2209_MOESM3_ESM.pdf]
