# Supplementary material for: Identification of Nicotiana benthamiana microRNAs and their targets using high throughput sequencing and degradome analysis
Source: BMC Genomics. 2015 Dec 1;16:1025. doi: 10.1186/s12864-015-2209-6 (PMC4667520; doi:10.1186/s12864-015-2209-6)
Supplement: Additional file 5: Figure S3. — Secondary structures of knew N. benthamiana specific miRNAs found in our liraries. The 5′ end of the RNA is marked by a circle. Candidant miRNAs are highlighted with different colours. Image was generated by RNAfold/RNAplot on The University of East Anglia sRNA toolkit (plant version). (PDF 219 kb) [file 12864_2015_2209_MOESM5_ESM.pdf]

Additional Figure 3.

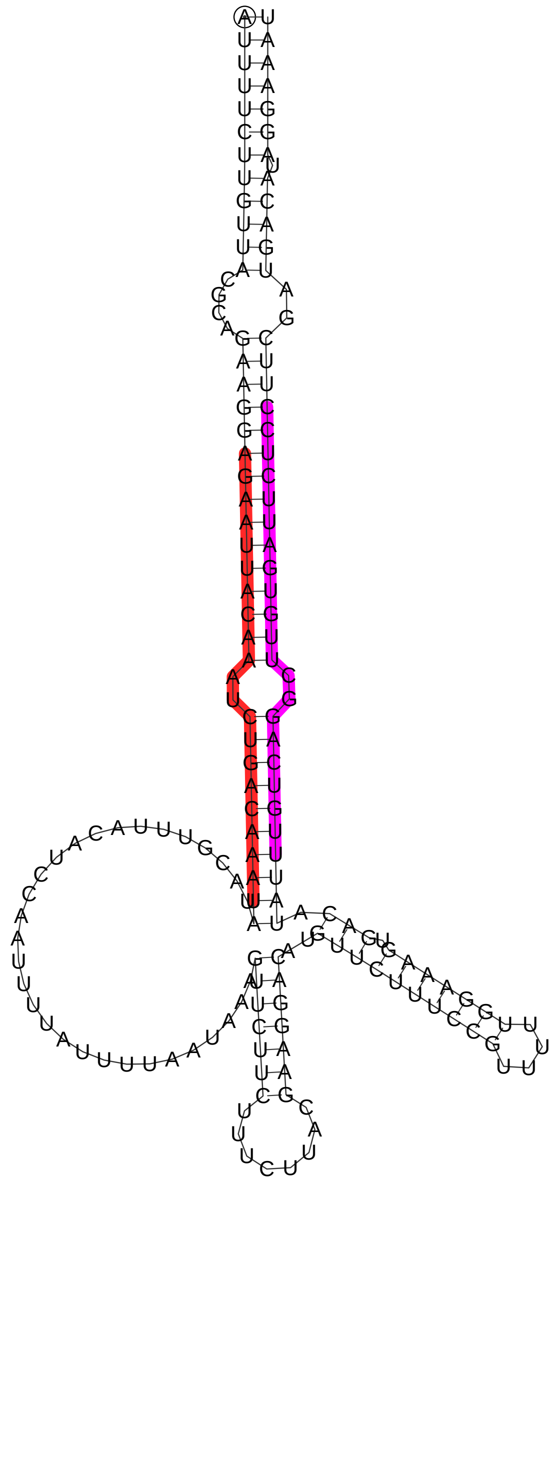

Secondary structure for ’Nb_miRC1’

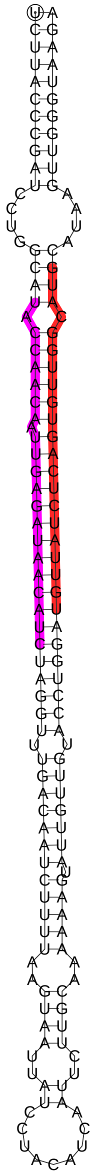

Secondary structure for ’Nb_miRC2’

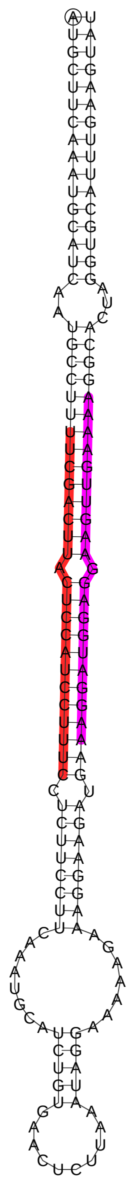

Secondary structure for ’Nb_miRC3’

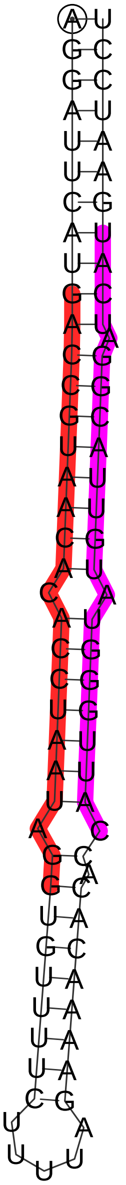

Secondary structure for ’Nb_miRC4’

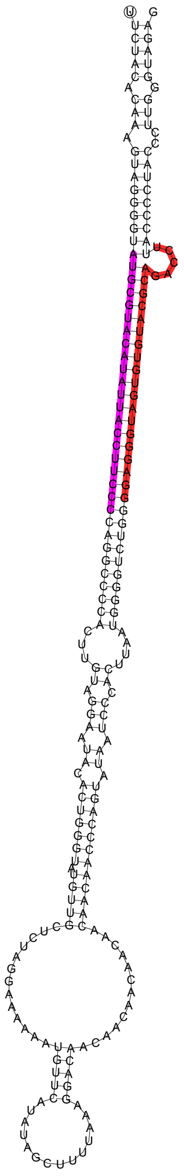

Secondary structure for 'Nb_miRC5'

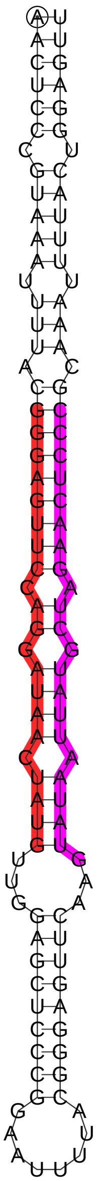

Secondary structure for 'Nb_miRC6'

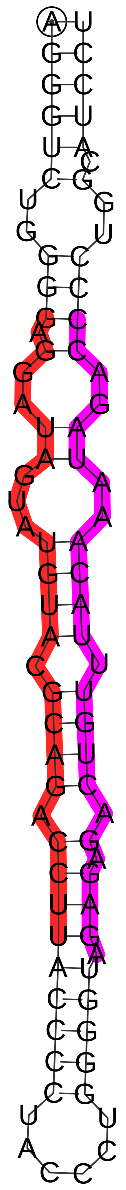

Secondary structure for 'Nb_miRC7'

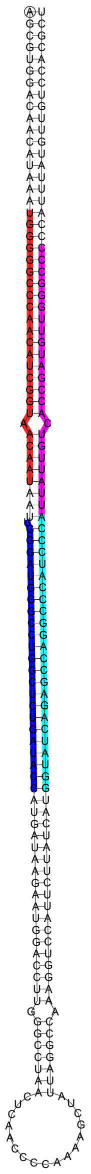

Secondary structure for ’Nb_miRC8’

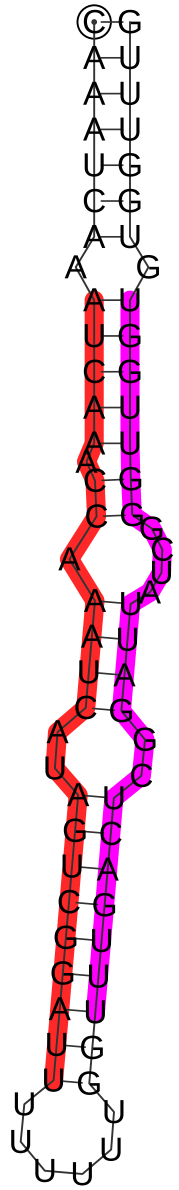

Secondary structure for ’Nb_miRC9’

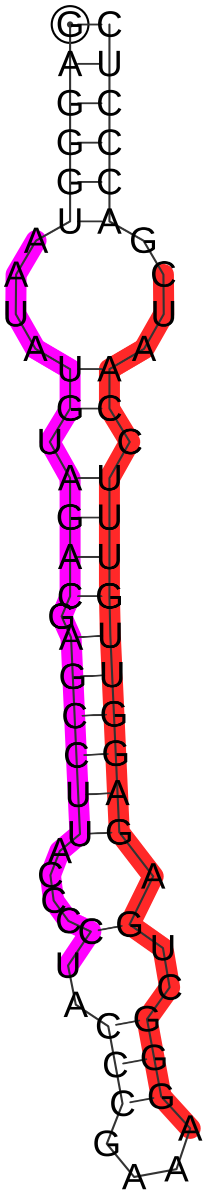

Secondary structure for ’Nb_miRC10’

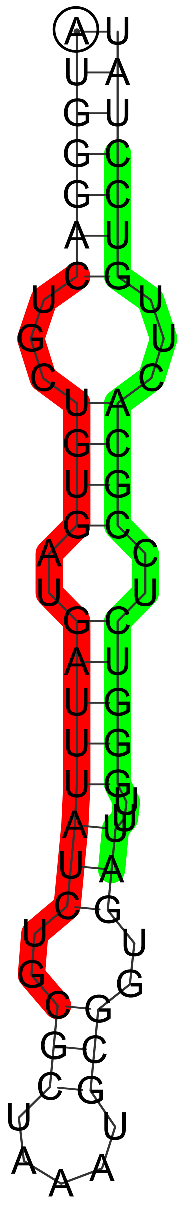

Secondary structure for 'Nb_miRC11

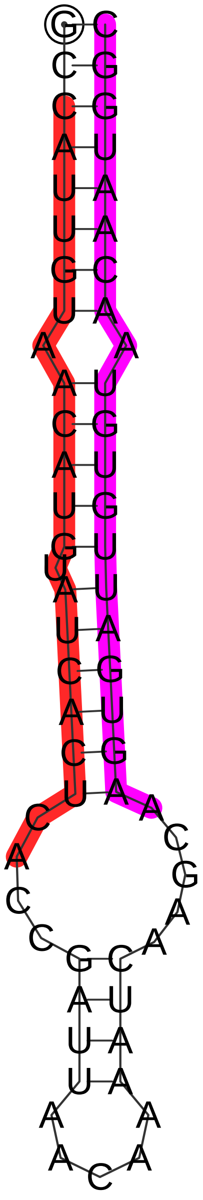

Secondary structure for ’Nb_miRC12’

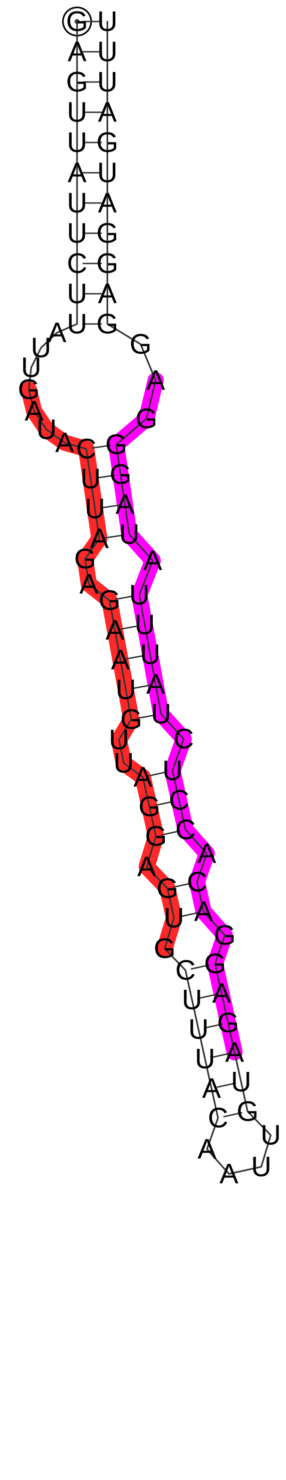

Secondary structure for 'Nb_miRC13'

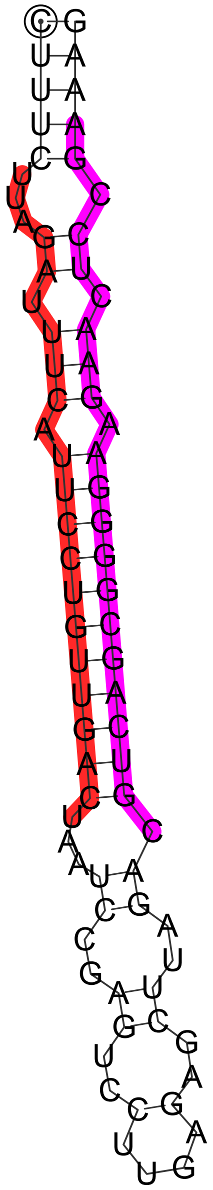

Secondary structure for ’Nb_miRC14’

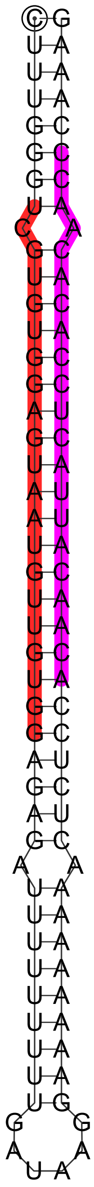

Secondary structure for 'Nb_miRC15'

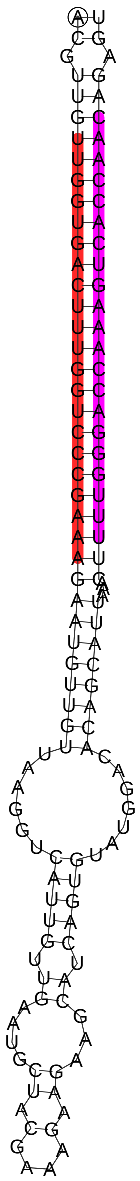

Secondary structure for ’Nb_miRC16’

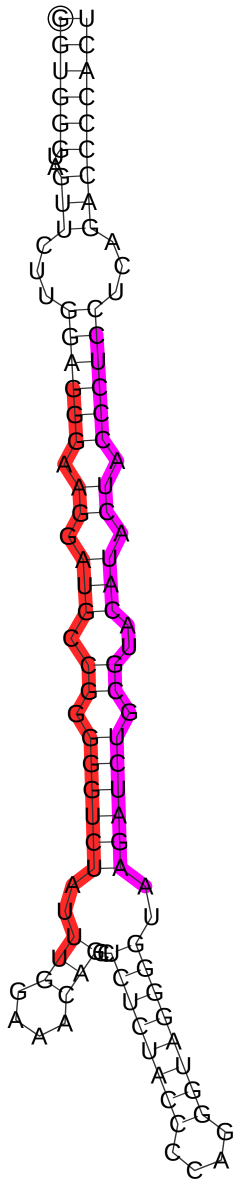

Secondary structure for 'Nb_miRC17'

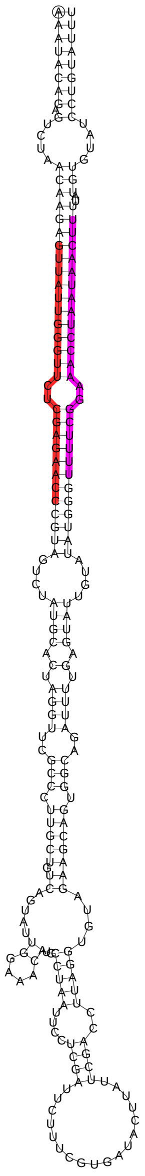

Secondary structure for 'Nb_miRC18'
