# Supplementary material for: Identification of Nicotiana benthamiana microRNAs and their targets using high throughput sequencing and degradome analysis
Source: BMC Genomics. 2015 Dec 1;16:1025. doi: 10.1186/s12864-015-2209-6 (PMC4667520; doi:10.1186/s12864-015-2209-6)
Supplement: Additional file 7: Figure S5. — Target plots (t-plots) of N. benthamiana specific knew miRNA targets confirmed by degradome sequencing. In the head of the pictures there is a unique identifier of the mRNA followed by the annotation of the transcript. The solid lines and dot in miRNA: mRNA alignments indicate matched RNA base pairs and GU mismatch, respectively. The relative abundances are plotted against the nucleotide position within the transcript. (PDF 364 kb) [file 12864_2015_2209_MOESM7_ESM.pdf]

Additional Figure 5.

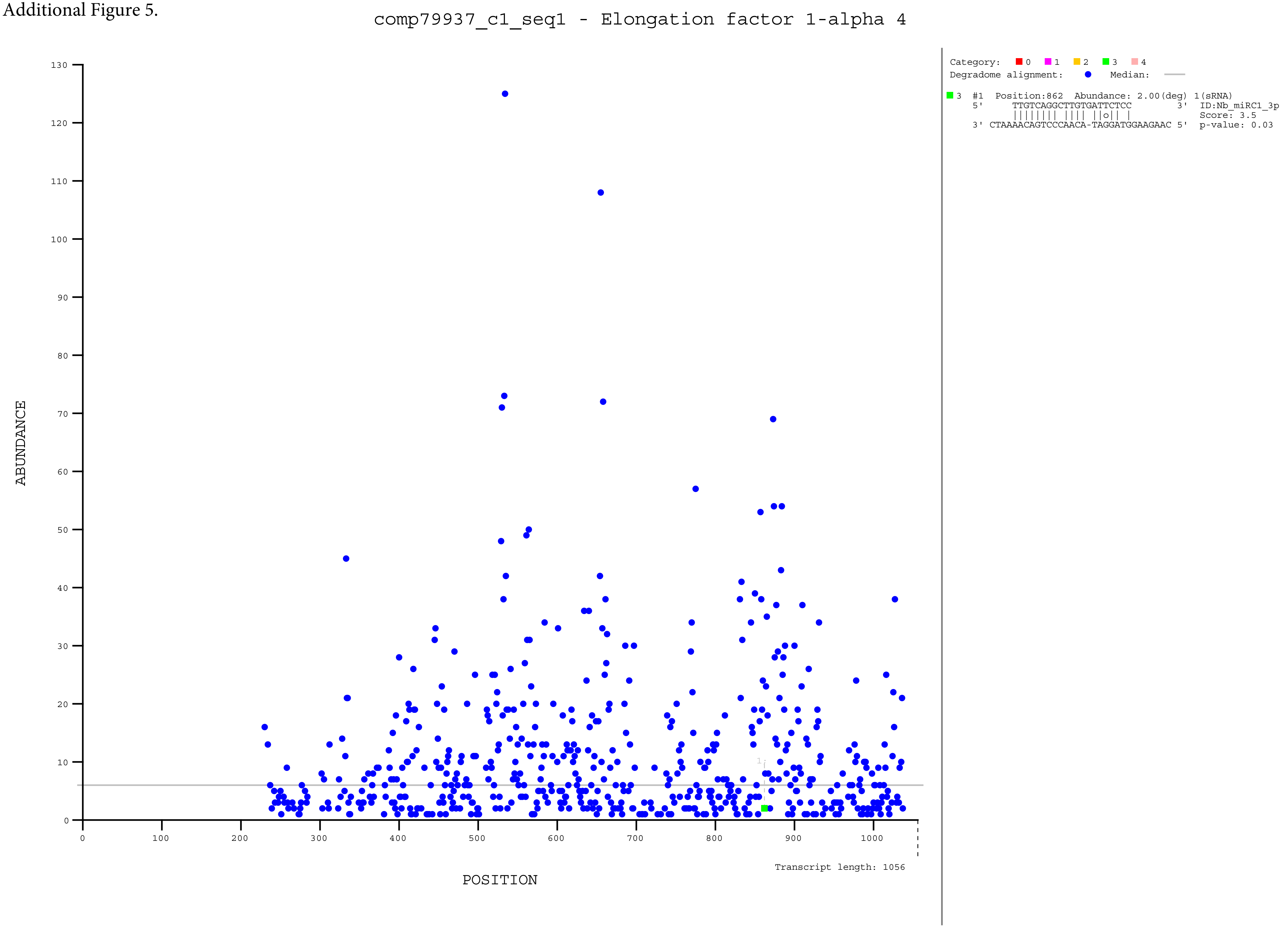

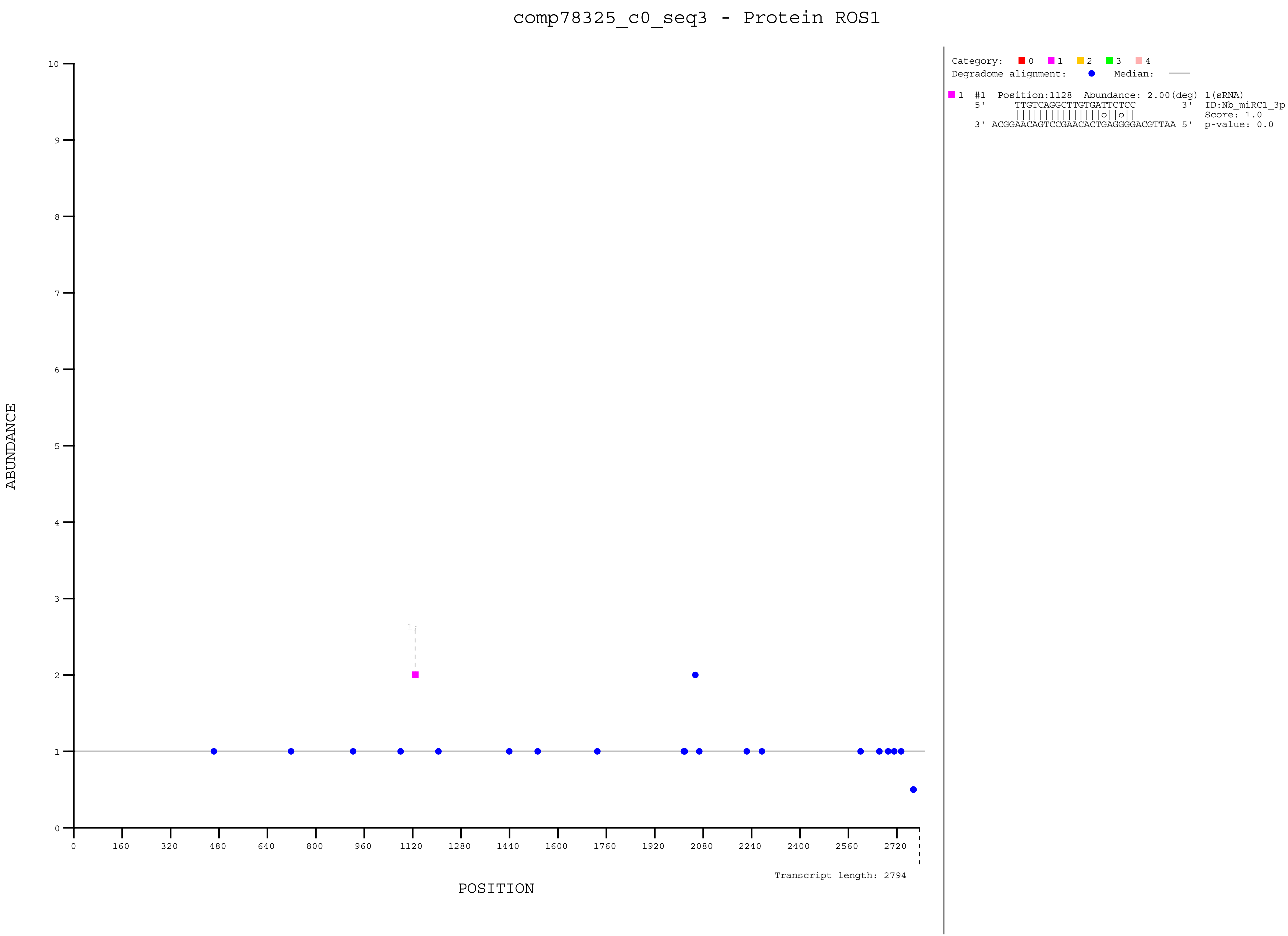
comp78325_c0_seq3 - Protein ROS1
Category: 0 1 2 3 4
Degradome alignment:
Median:
1 #1 Position:1128 Abundance: 2.00(deg) 1(sRNA)
5' TTGTCAGGCTTGTGATTCTCC 3' ID:Nb_miRC1_3p
||||||||||||||o||o||
Score: 1.0
3' ACGGAACAGTCCGAACACTGAGGGGACGTTAA 5' p-value: 0.0
ABUNDANCE
POSITION
Transcript length: 2794

comp75577_c0_seq17 - Down syndrome critical region protein 3 homolog

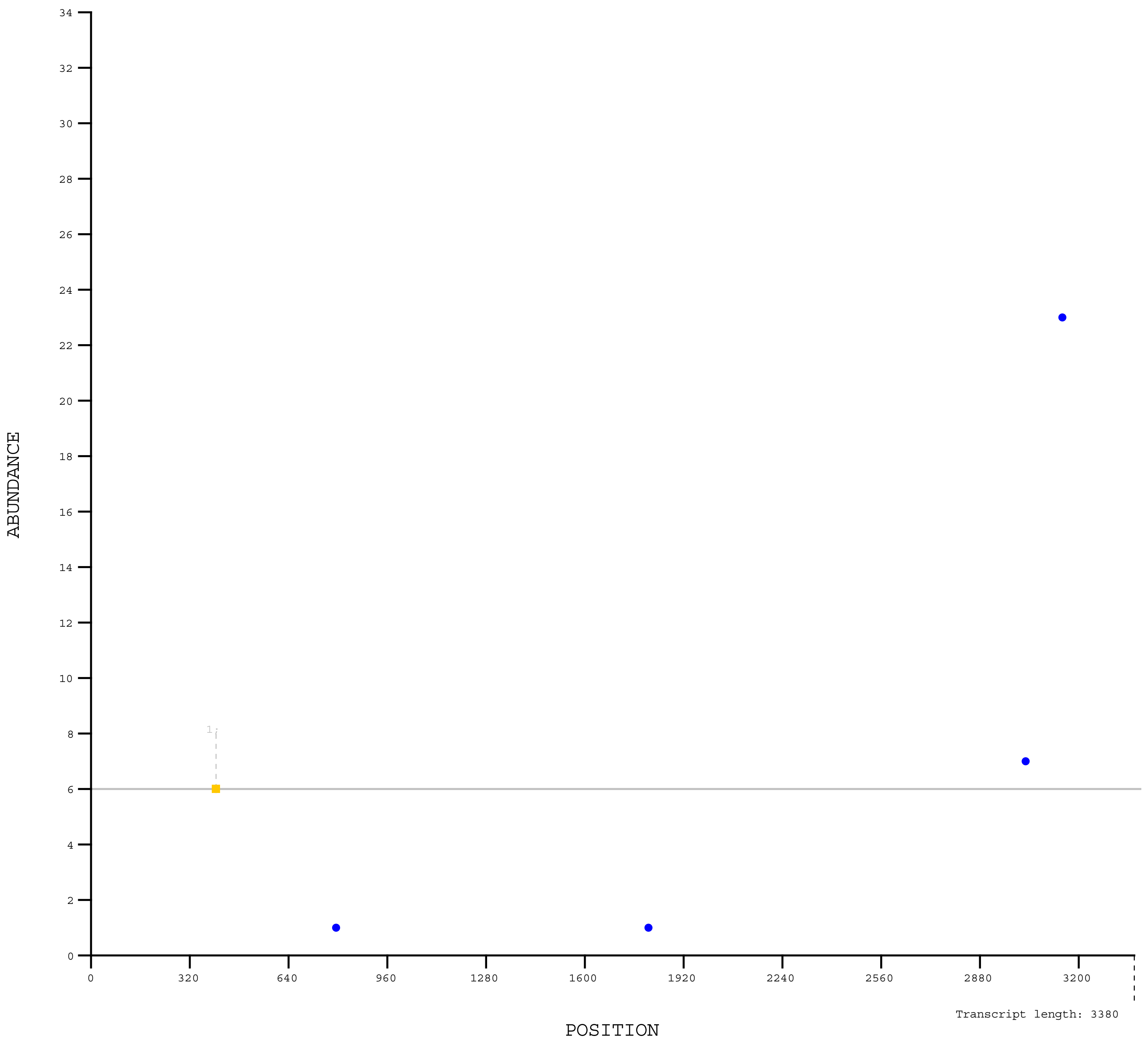

Category: 0 1 2 3 4
Degradome alignment: Median:

```
2  #1  Position:405  Abundance: 6.00(deg) 1(sRNA)
   5'      TGTTATCTCAGTGTTGGCATG          3'  ID:Nb_miRC2_3p
           | |||| |||||o|||| ||o              Score: 4.0
   3' TGTCAGAATA-AGTCATAACCTTATCCGAATT 5'  p-value: 0.04
```

comp79768_c0_seq4 - Elongation factor Ts

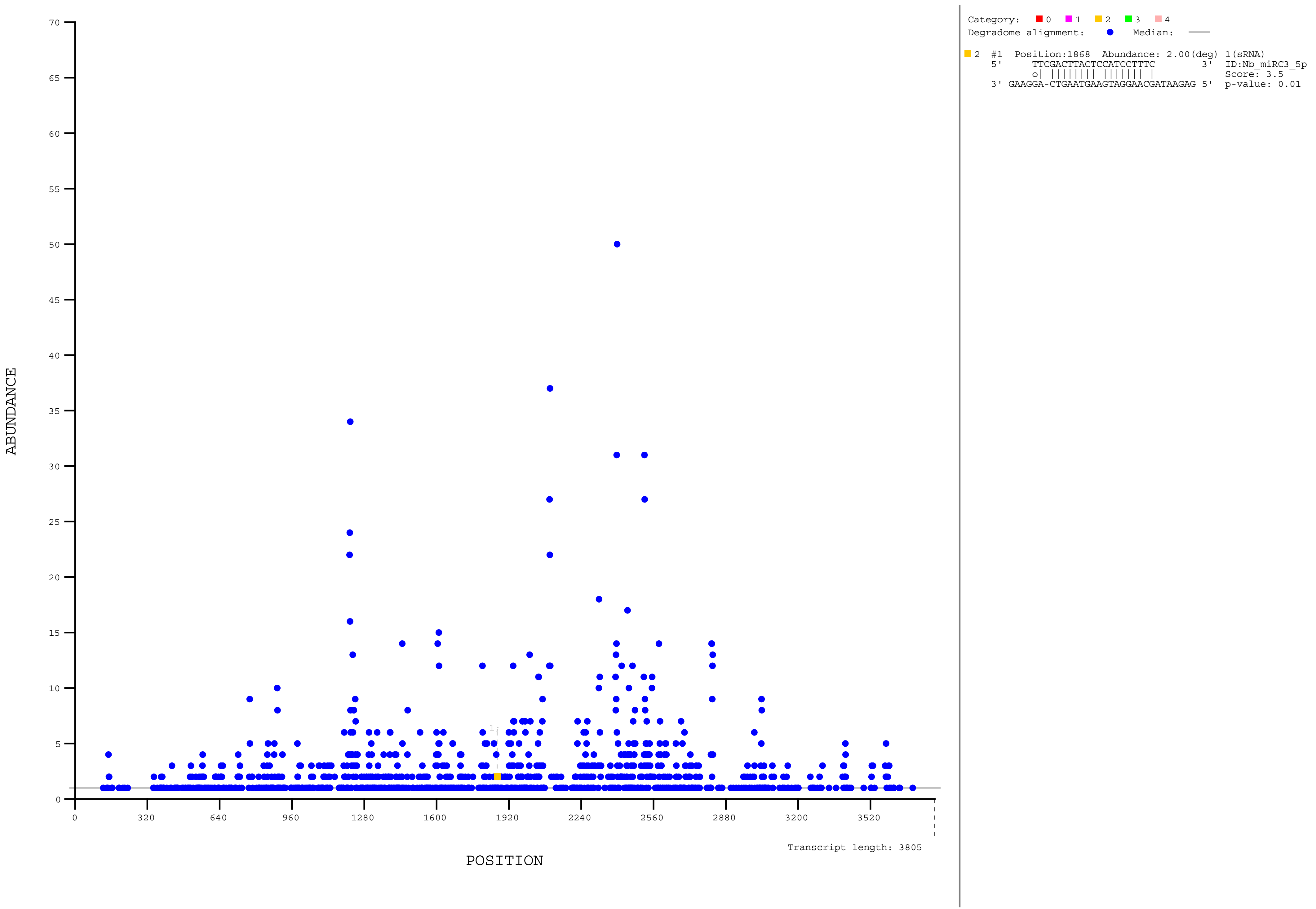

comp76033_c0_seq2 - CBL-interacting serine/threonine-protein kinase 5

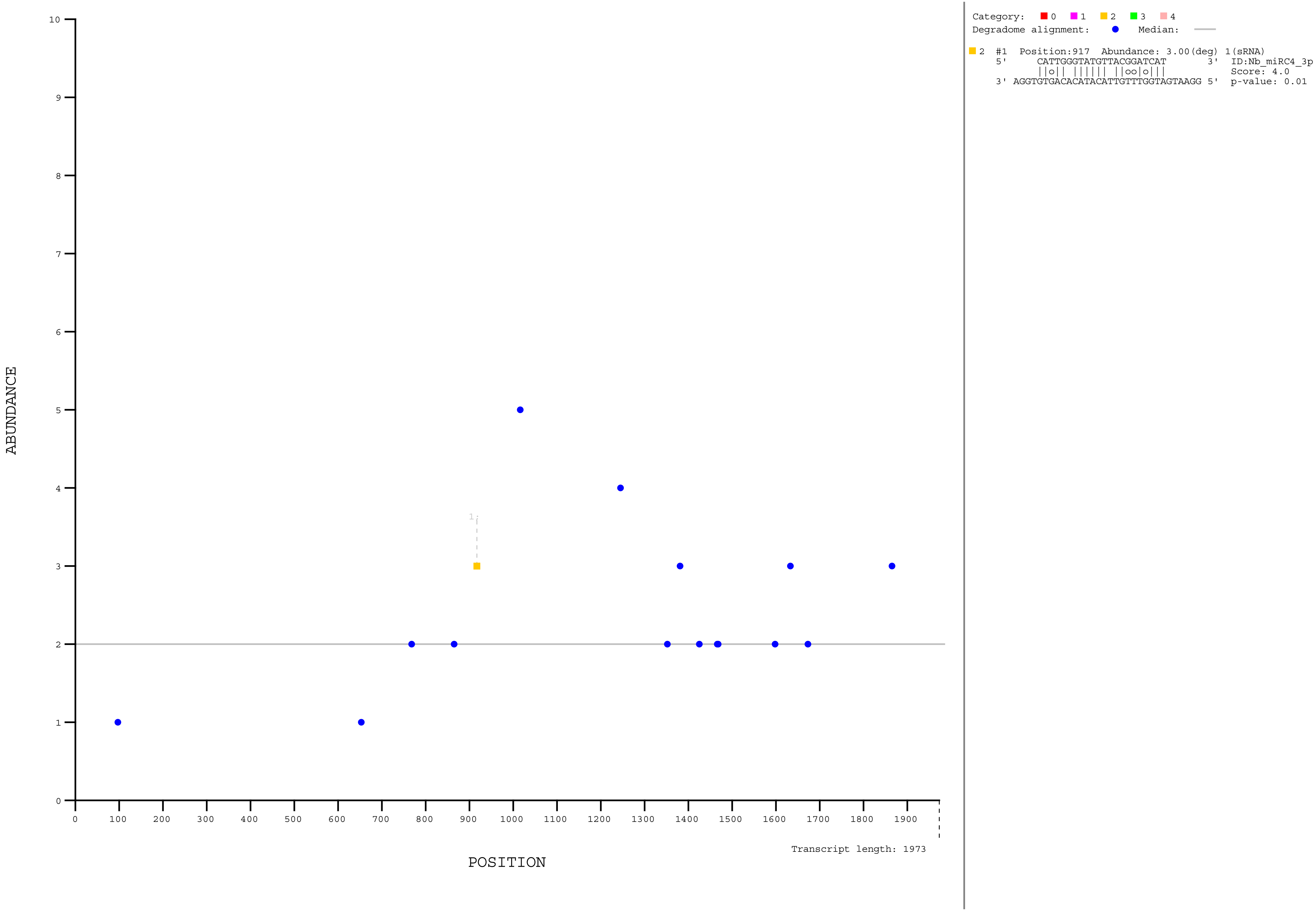

comp73317_c0_seq28 * Mbolufpljb _fldbkbpfp c^`qlo .-

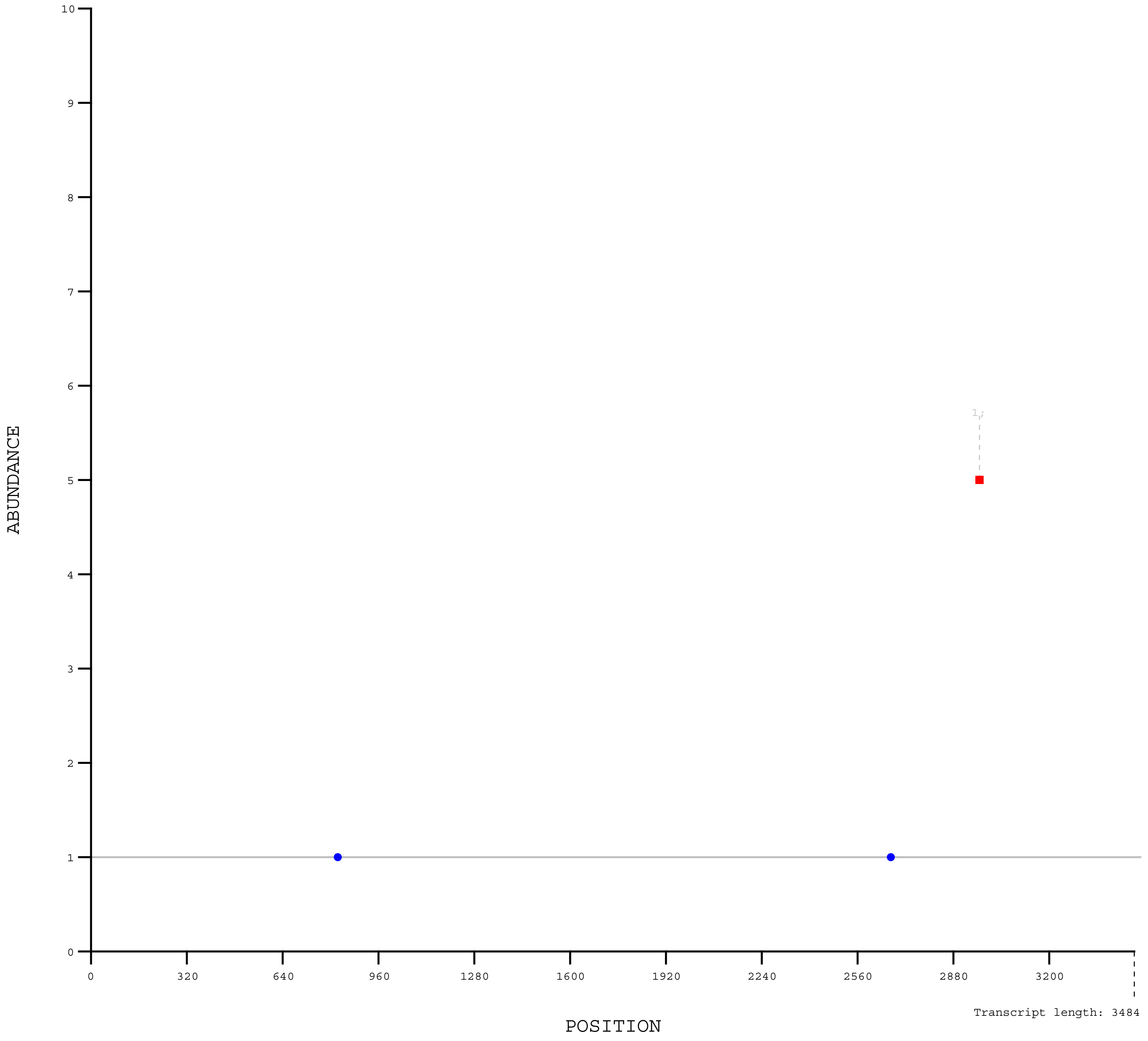


```
Category: 0 1 2 3 4
Degradome alignment:    Median:

0  #1  Position:2967  Abundance: 5.00(deg) 3(sRNA)
   5'     GGAGGGTAGTGTGTACGCAGACCT     3'  ID:K_\jfO@2\0m
          ||||||||o|||||| |||||  |         Score: 3.5
   3' GATTCCTCCCATTACACATACGTCTCCAACAA 5'  p-value: 0.0
```

comp69461_c0_seq1 - Protein CREG1

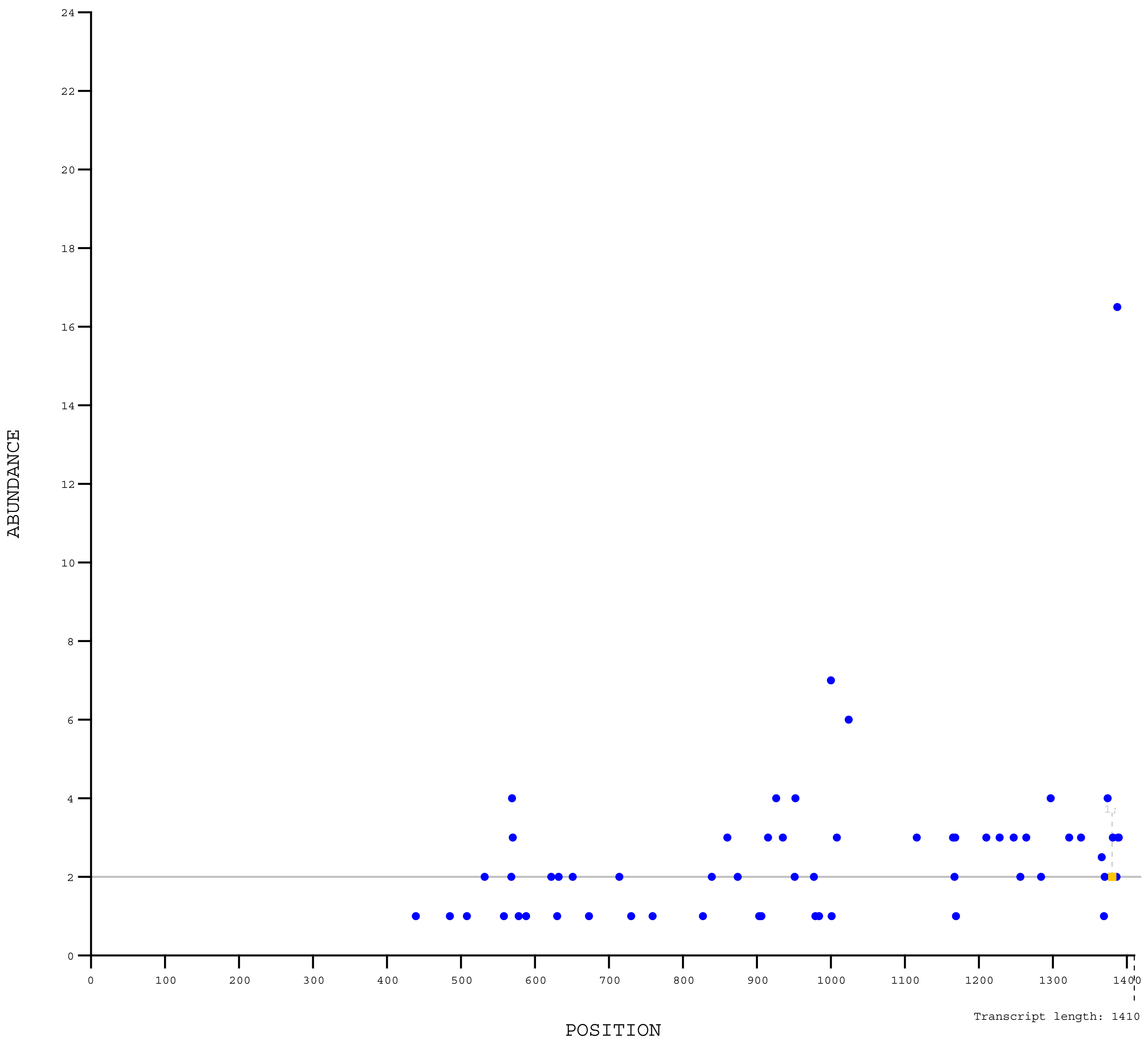

Category: 0 1 2 3 4
Degradome alignment: ● Median: —

```
2  #1  Position:1380  Abundance: 2.00(deg) 3(sRNA)
   5'     GGAGGGTAGTGTGTACGCAGACCT     3'  ID:Nb_miRC5_3p
          |||o||||||||||||||||||||         Score: 0.5
   3' GACCCCTTCCATCACACATGCGTCTGGATTCG 5'  p-value: 0.0
```

comp76292_c1_seq18 - Probable WRKY transcription factor 53

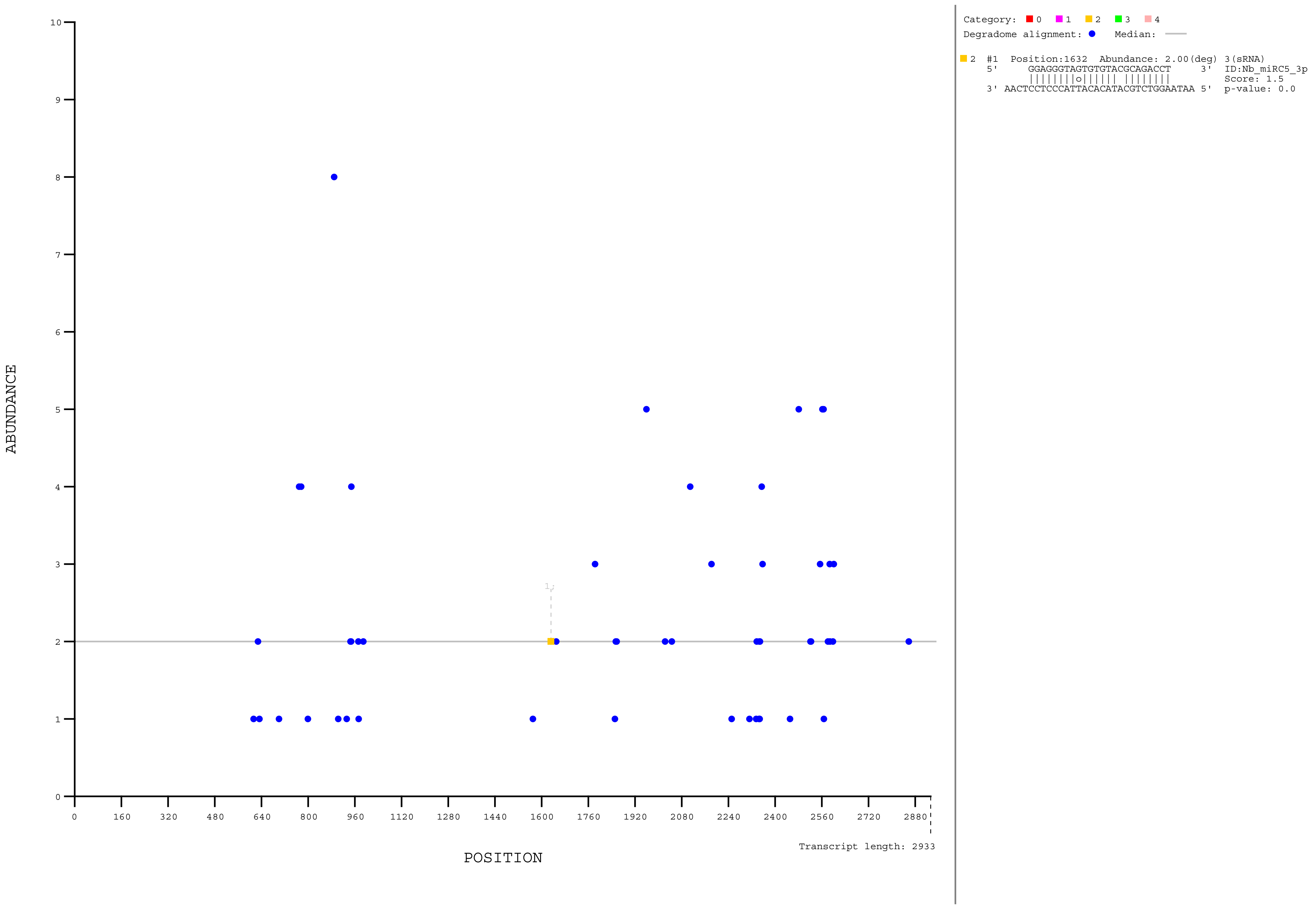

comp72235_c0_seq2 - Cytochrome b561 and DOMON domain-containing protein At5g47530-like

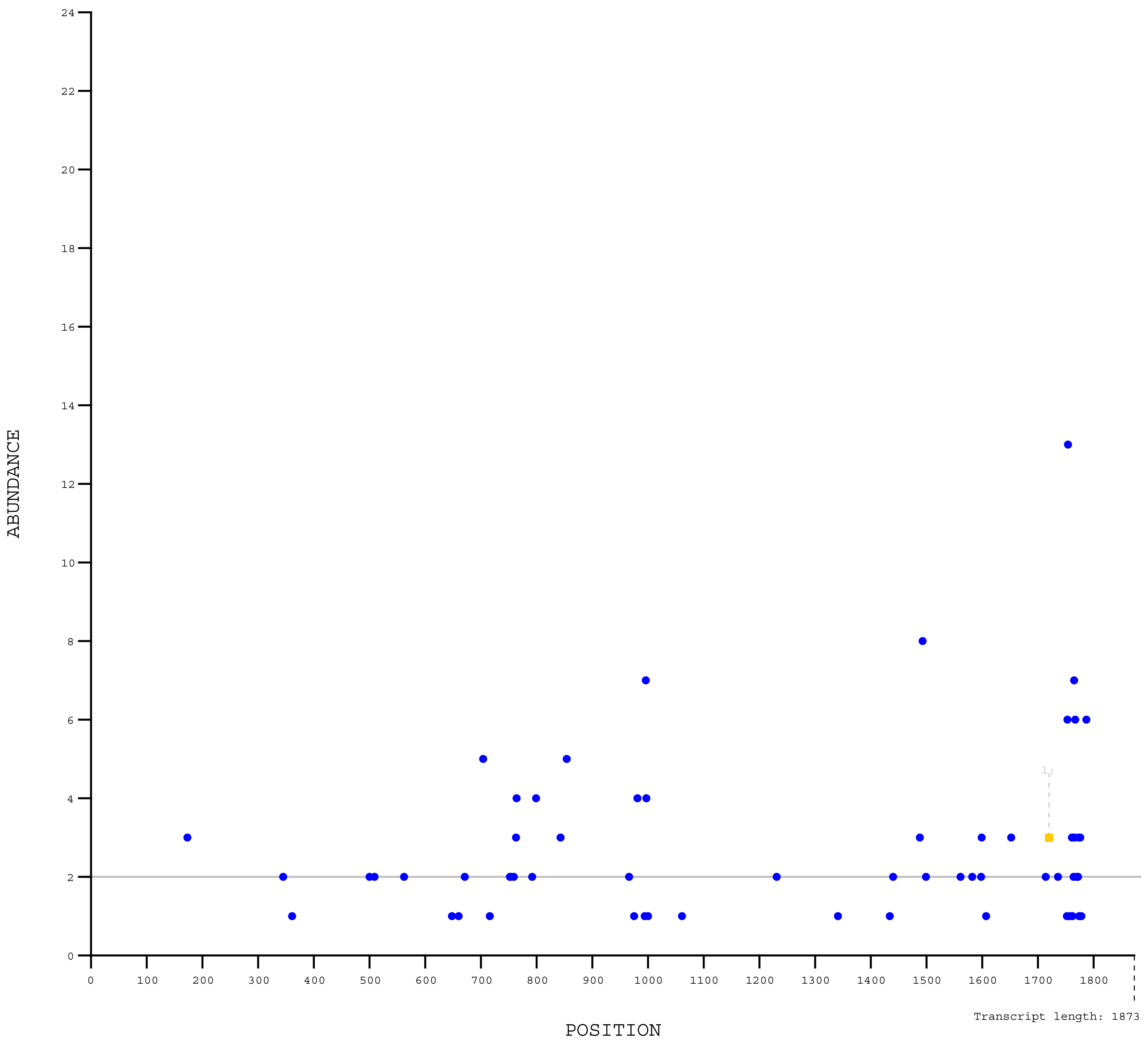

Category: 0 1 2 3 4

Degradome alignment: ● Median: —

```
2  #1  Position:1720  Abundance: 3.00(deg) 3(sRNA)
   5'     GGAGGGTAGTGTGTACGCAGACCT     3'  ID:Nb_miRC5_3p
          |o||||||o||||||||o| |||||        Score: 2.5
   3' GACCCTTCCCATTACACATGTGCCTGGAATGG 5'  p-value: 0.0
```

comp70843_c0_seq1 - Protein TIC 21, chloroplastic

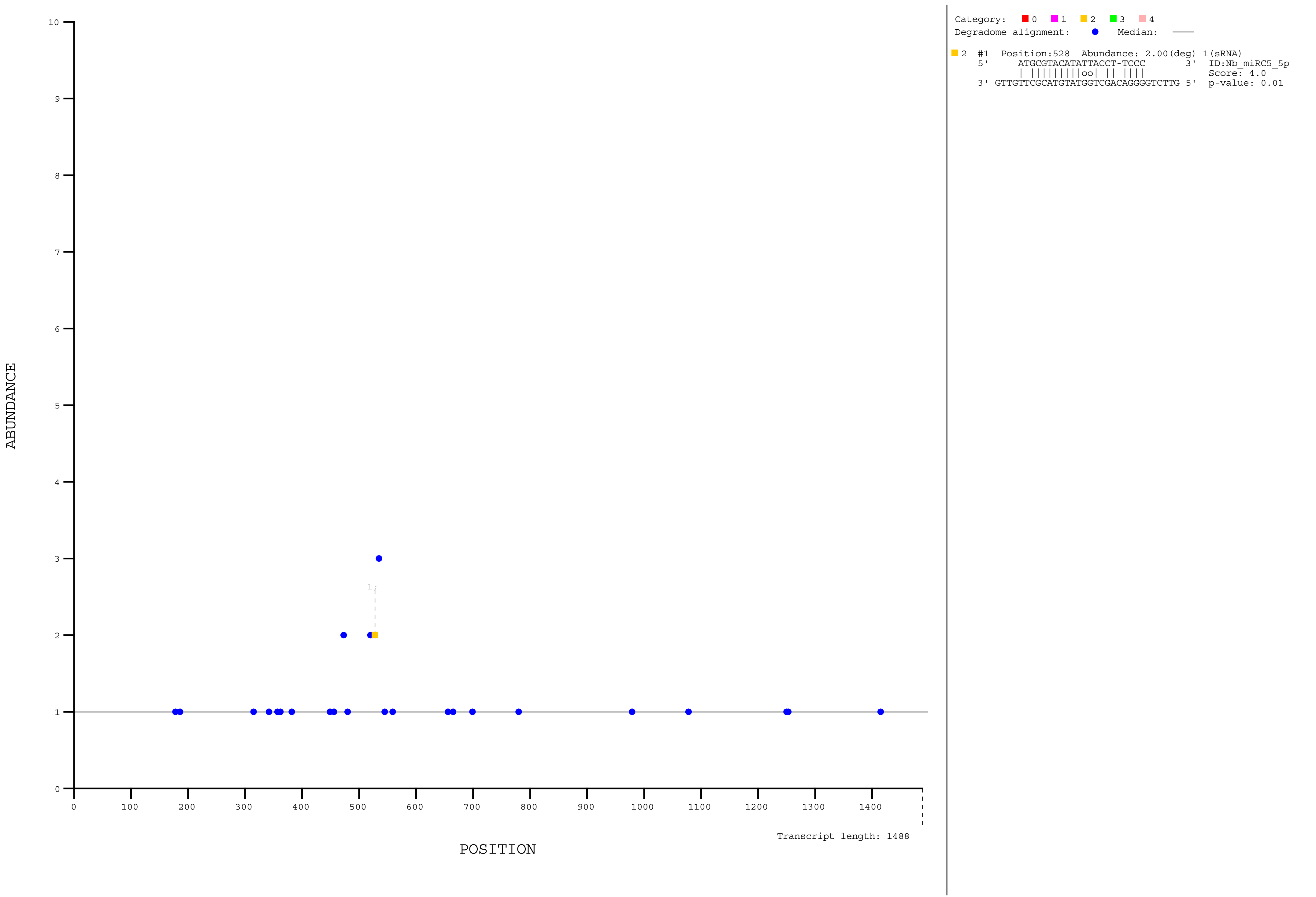

comp75362_c2_seq1 - G-type lectin S-receptor-like serine/threonine-protein kinase At1g11330

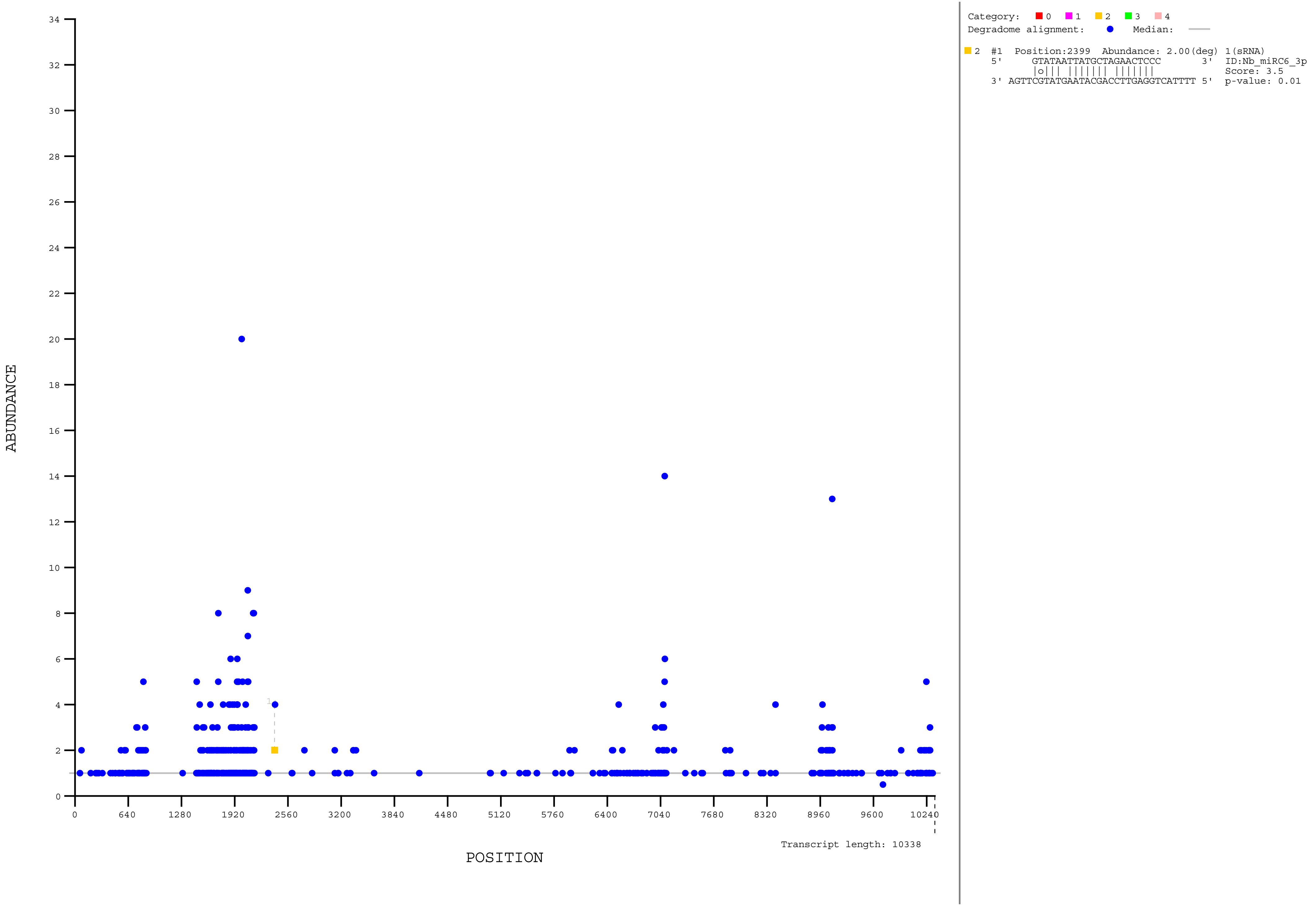

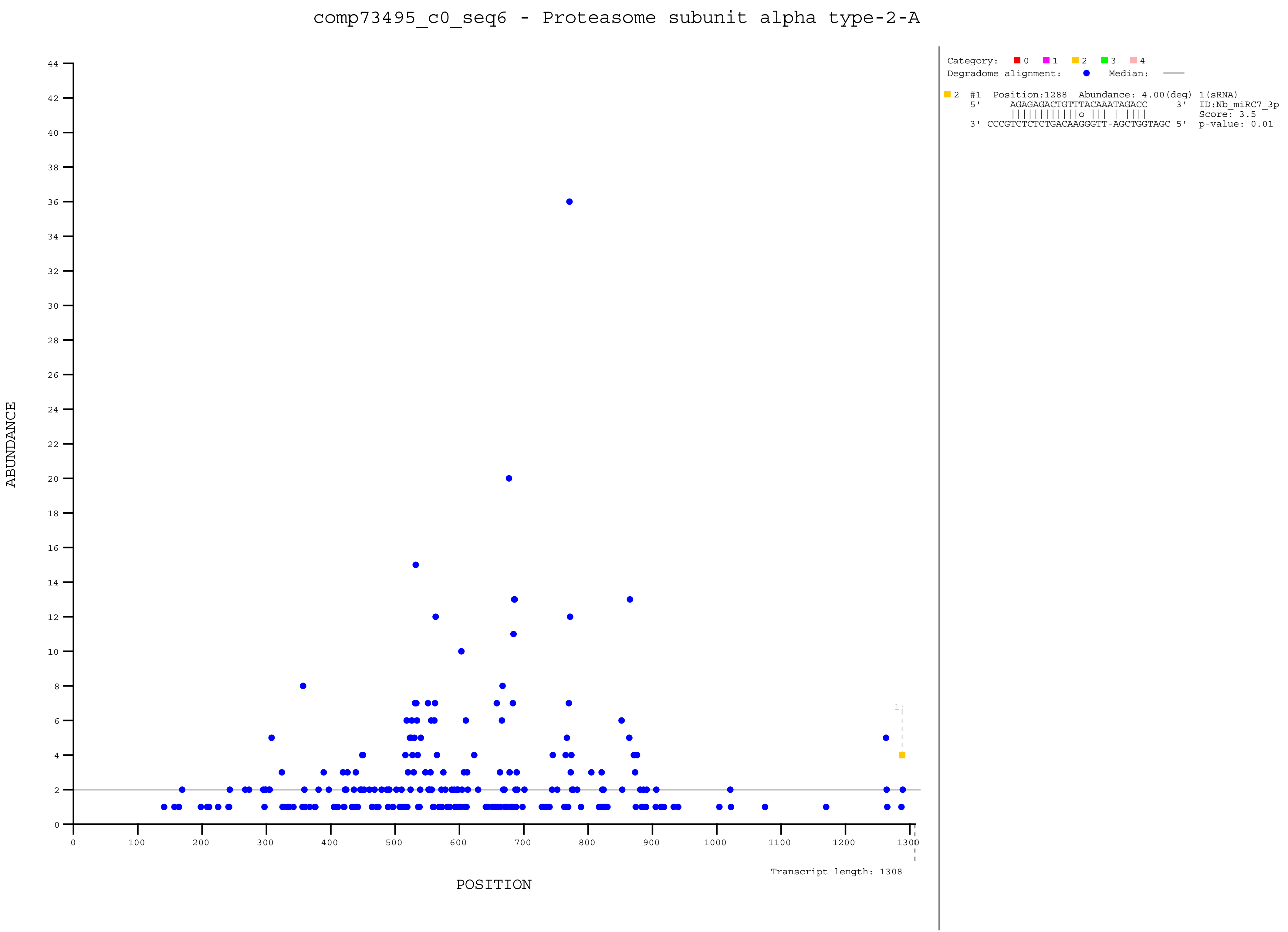
comp73495_c0_seq6 - Proteasome subunit alpha type-2-A
ABUNDANCE
0
2
4
6
8
10
12
14
16
18
20
22
24
26
28
30
32
34
36
38
40
42
44
POSITION
0
100
200
300
400
500
600
700
800
900
1000
1100
1200
1300
Transcript length: 1308
Category: 0 1 2 3 4
Degradome alignment:
Median:
2 #1 Position:1288 Abundance: 4.00(deg) 1(sRNA)
5' AGAGAGACTGTTTACAAATAGACC 3' ID:Nb_miRC7_3p
||||||||||||o ||| | |||| Score: 3.5
3' CCCGTCTCTCTGACAAGGGTT-AGCTGGTAGC 5' p-value: 0.01

comp56753_c0_seq1 - WAT1-related protein At5g40240-like

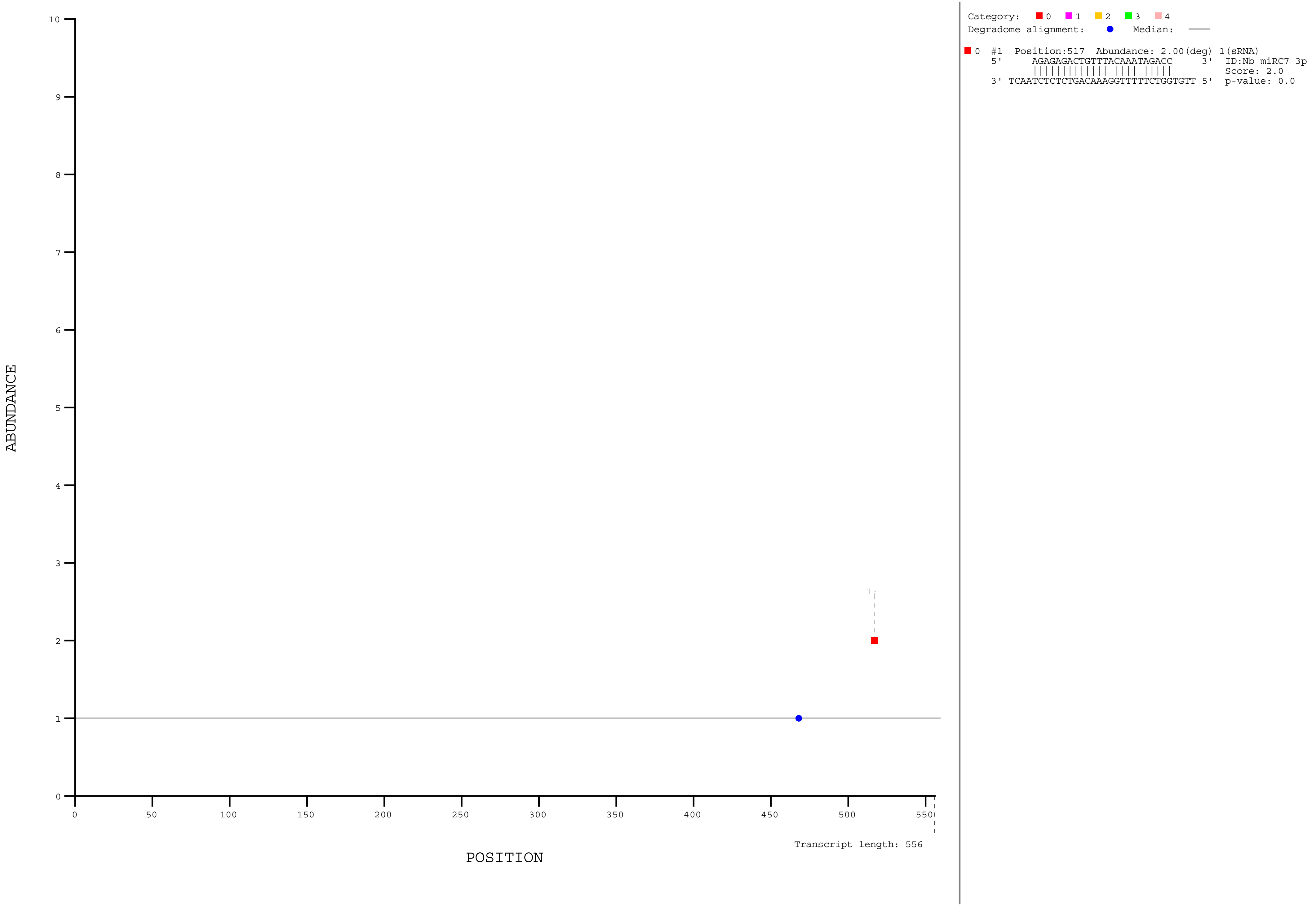

comp71383_c0_seq6 - Aquaporin TIP1-3

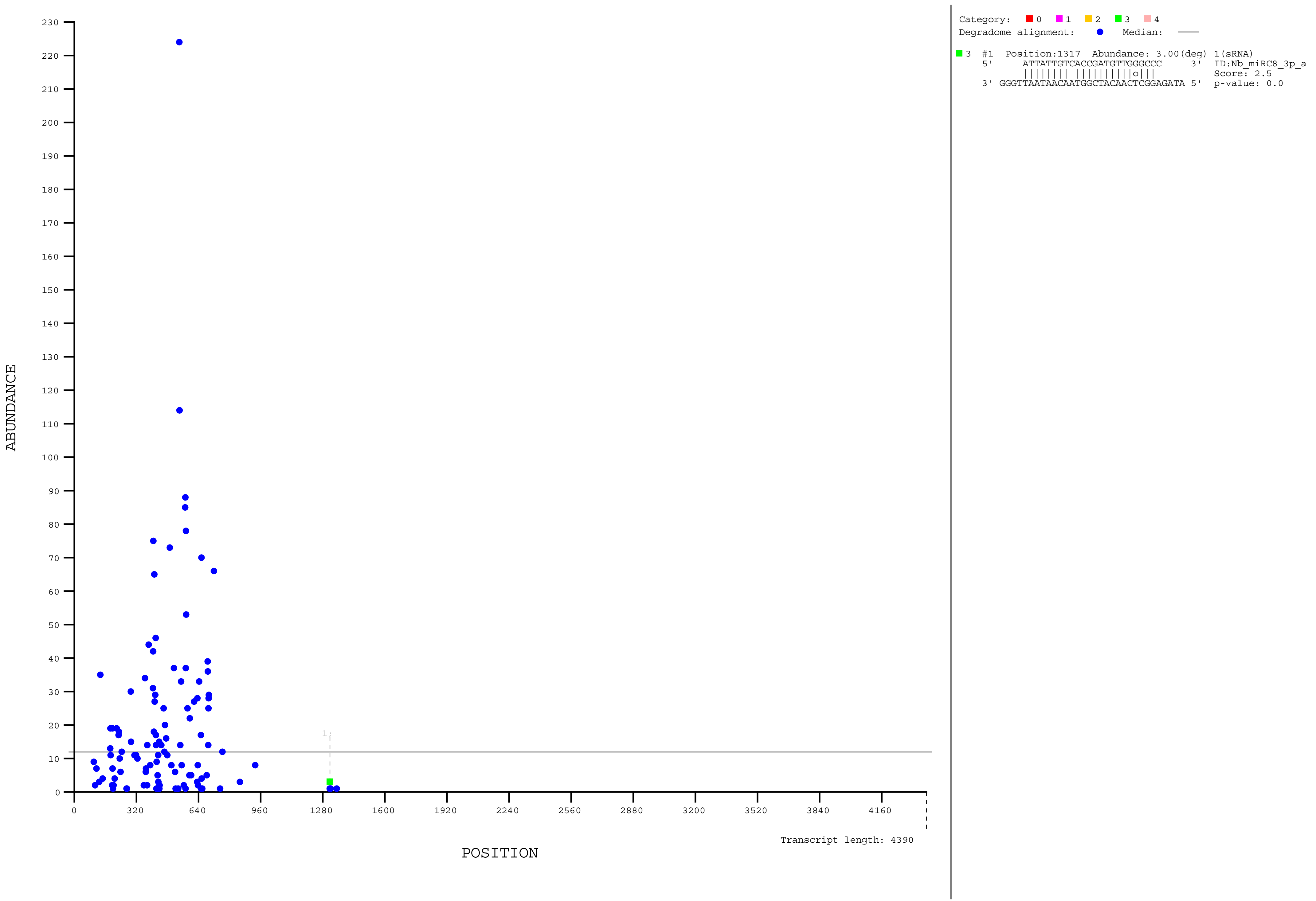

comp75266_c2_seq4 - Probable isoaspartyl peptidase/L-asparaginase 3

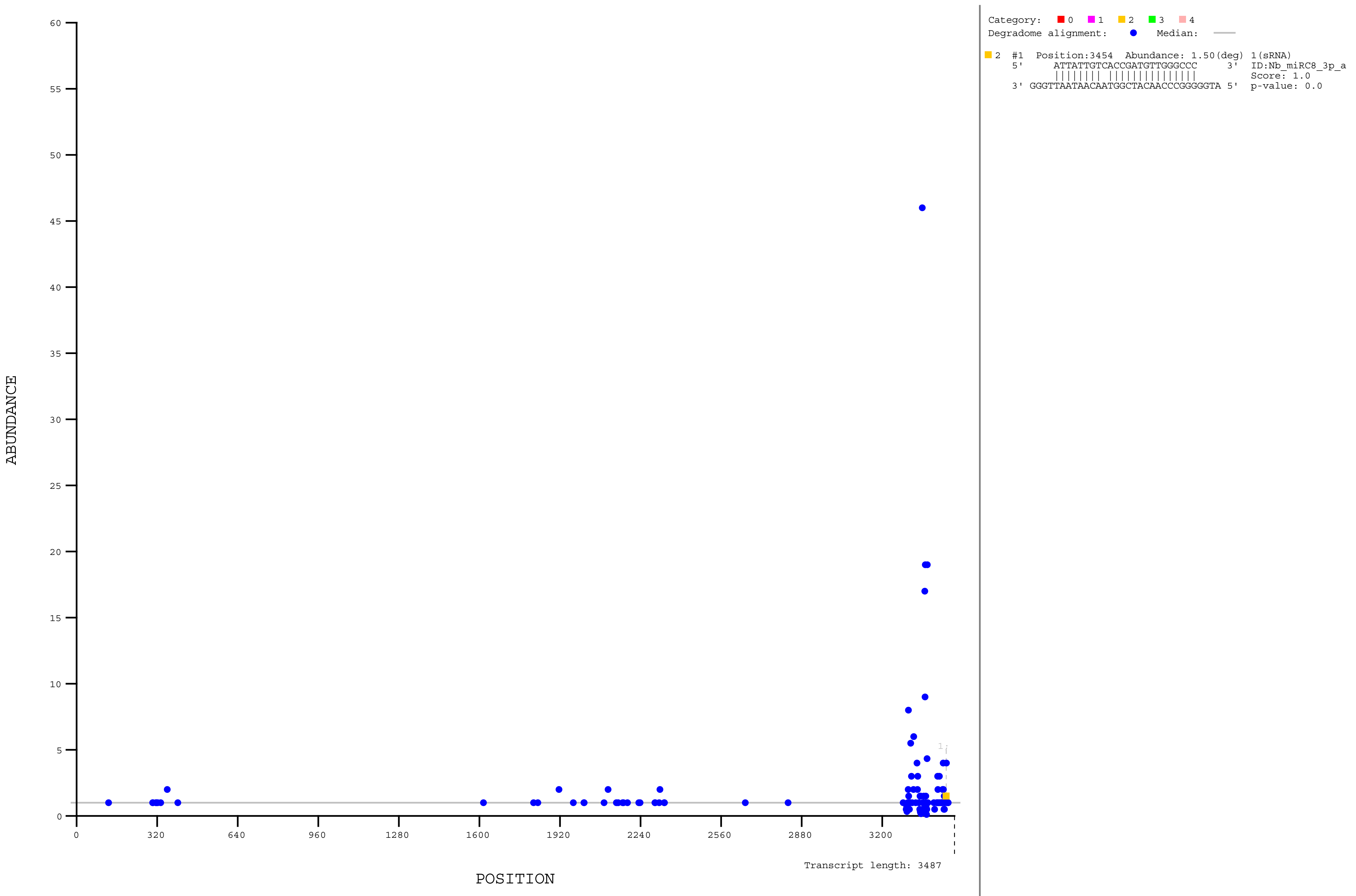

comp71496_c0_seq1 - Serine/threonine-protein phosphatase 2A regulatory subunit B'' subunit alpha

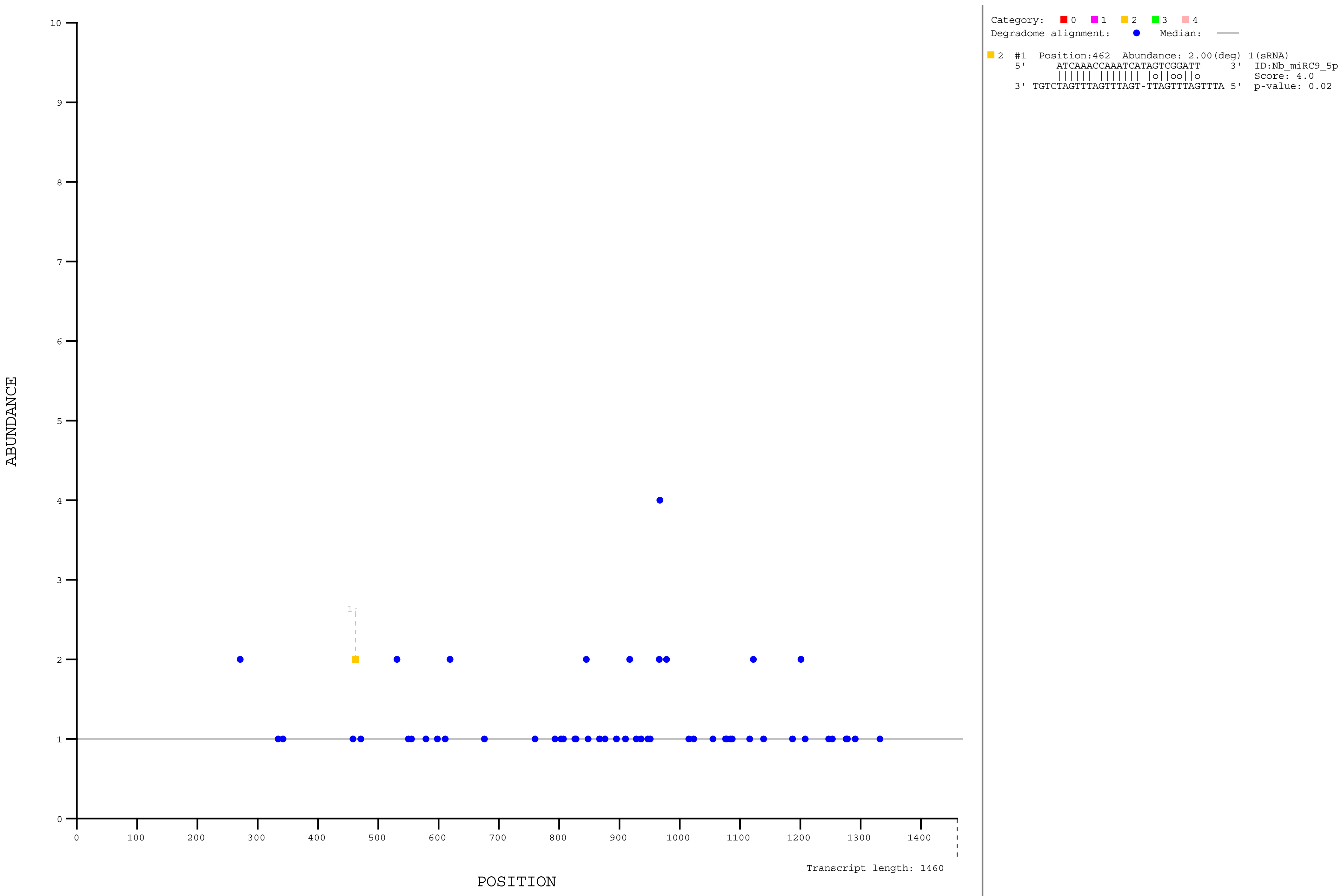

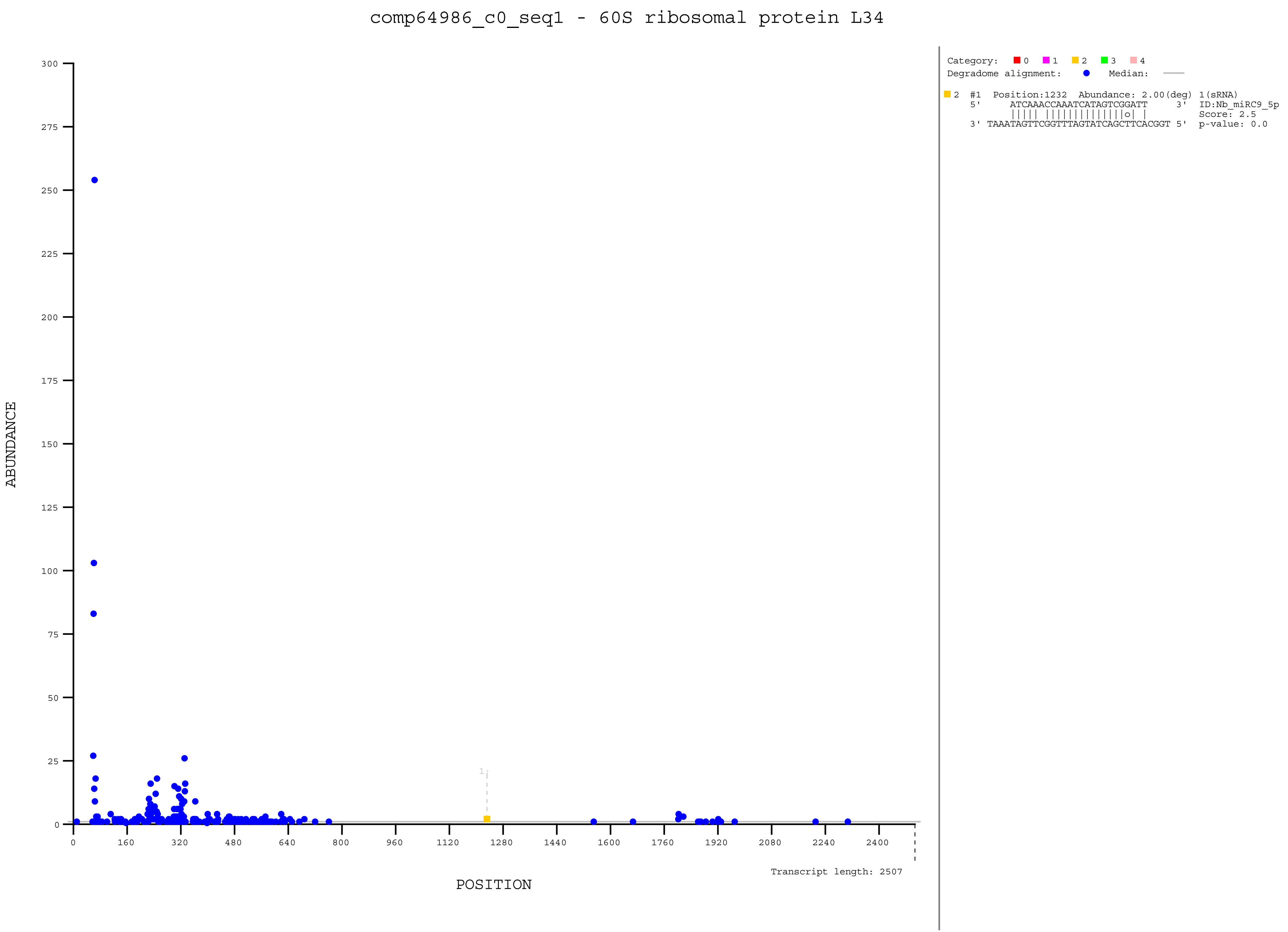
comp64986_c0_seq1 - 60S ribosomal protein L34
ABUNDANCE
300
275
250
225
200
175
150
125
100
75
50
25
0
0
160
320
480
640
800
960
1120
1280
1440
1600
1760
1920
2080
2240
2400
POSITION
Transcript length: 2507
Category: 0 1 2 3 4
Degradome alignment:
Median:
2 #1 Position:1232 Abundance: 2.00(deg) 1(sRNA)
5' ATCAAACCAAATCATAGTCGGATT 3' ID:Nb_miRC9_5p
Score: 2.5
3' TAAATAGTTCGGTTTAGTATCAGCTTCACGGT 5' p-value: 0.0

comp77607_c3_seq4 - Suberization-associated anionic peroxidase

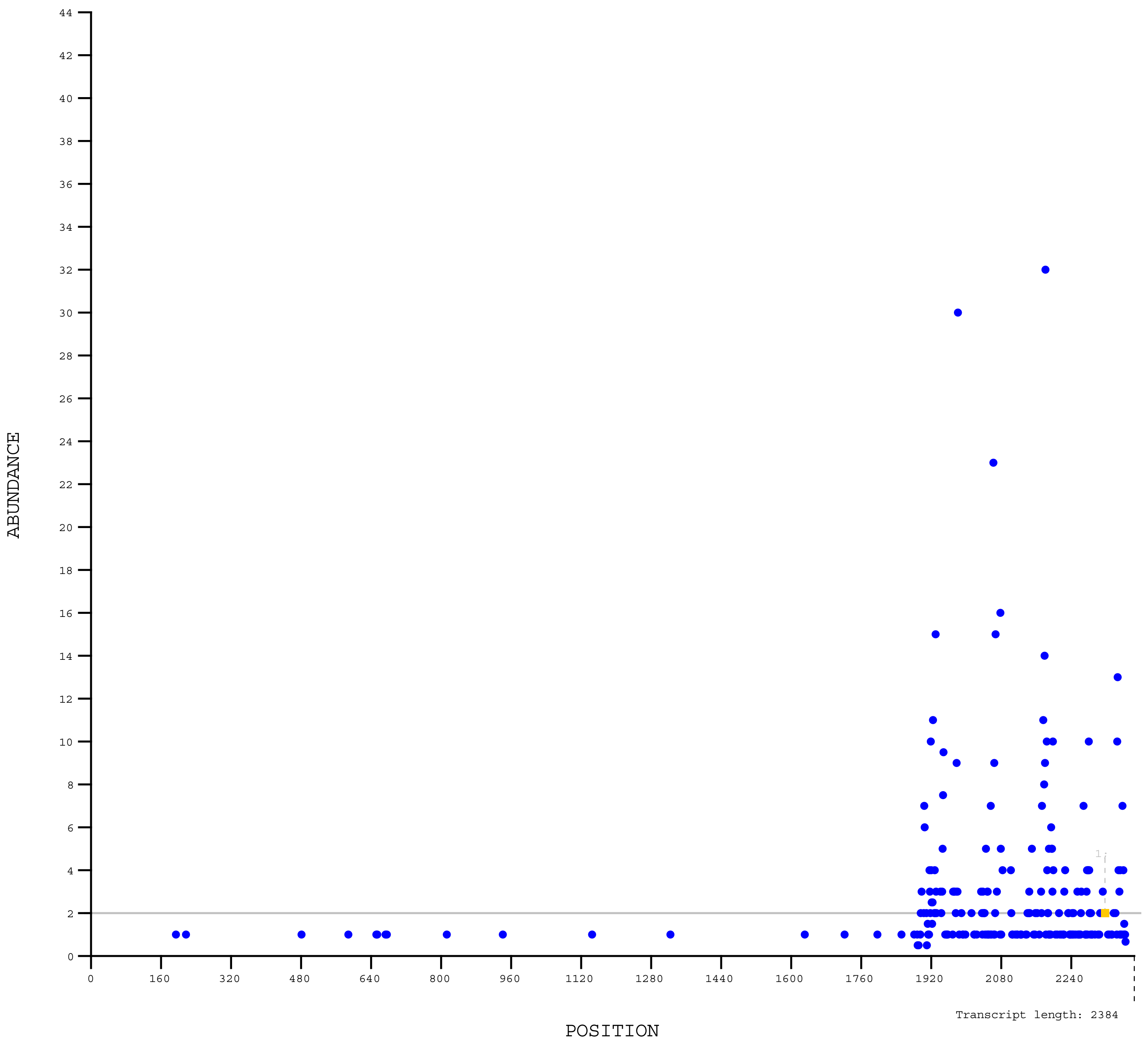


```
Category:  0  1  2  3  4
Degradome alignment:      Median:

2  #1  Position:2317  Abundance: 2.00(deg) 1(sRNA)
   5'     AGGGCTGAGAGGTTGTTTCCAATC     3'  ID:Nb_miRC10_3p
          ||||| ||||||o||o|||||  |         Score: 4.0
   3' GTTTTCCCGTCTCTCCGACGAAGGTACGCTGG 5'  p-value: 0.01
```

comp80078_c3_seq4 - Formate--tetrahydrofolate ligase

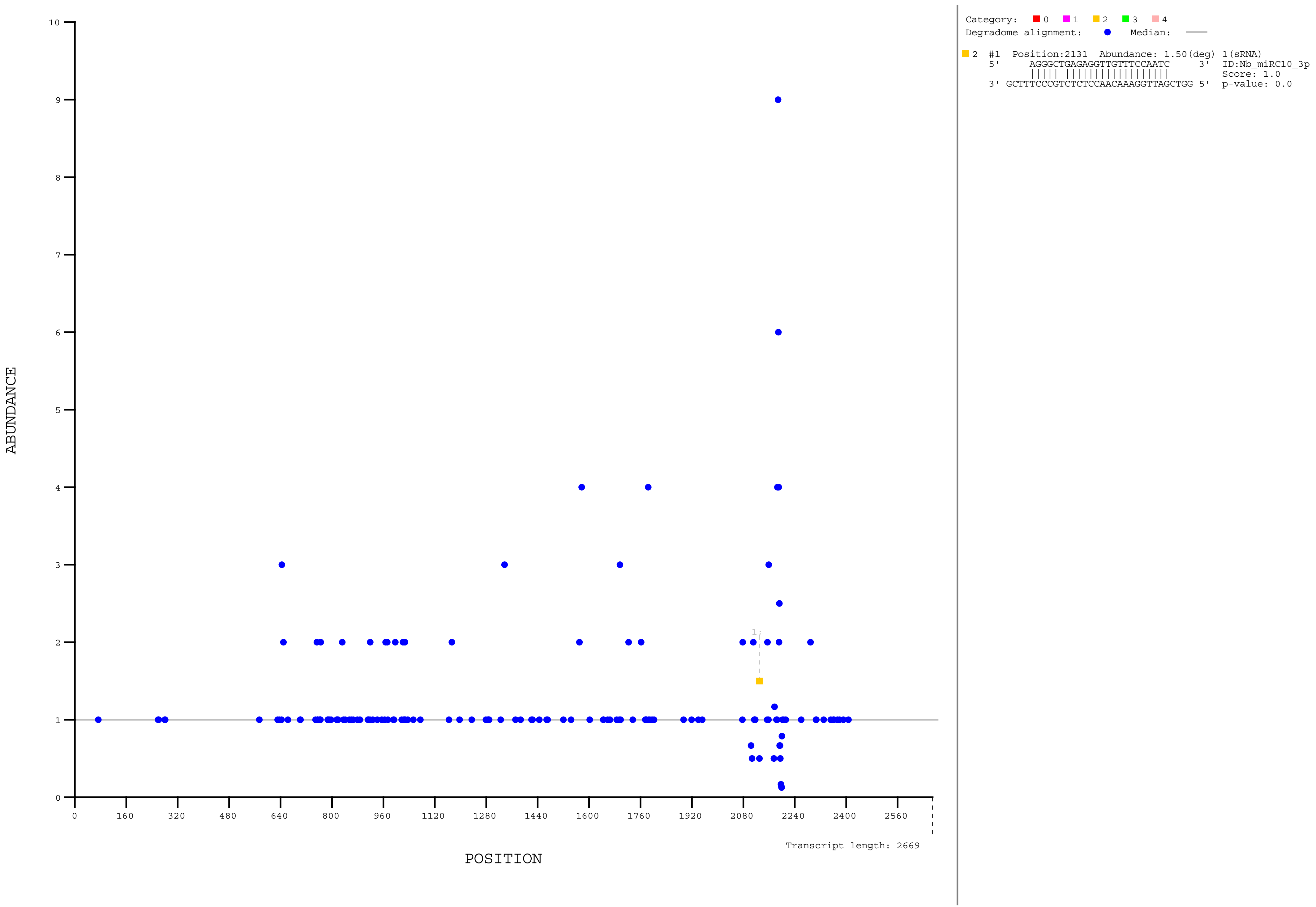

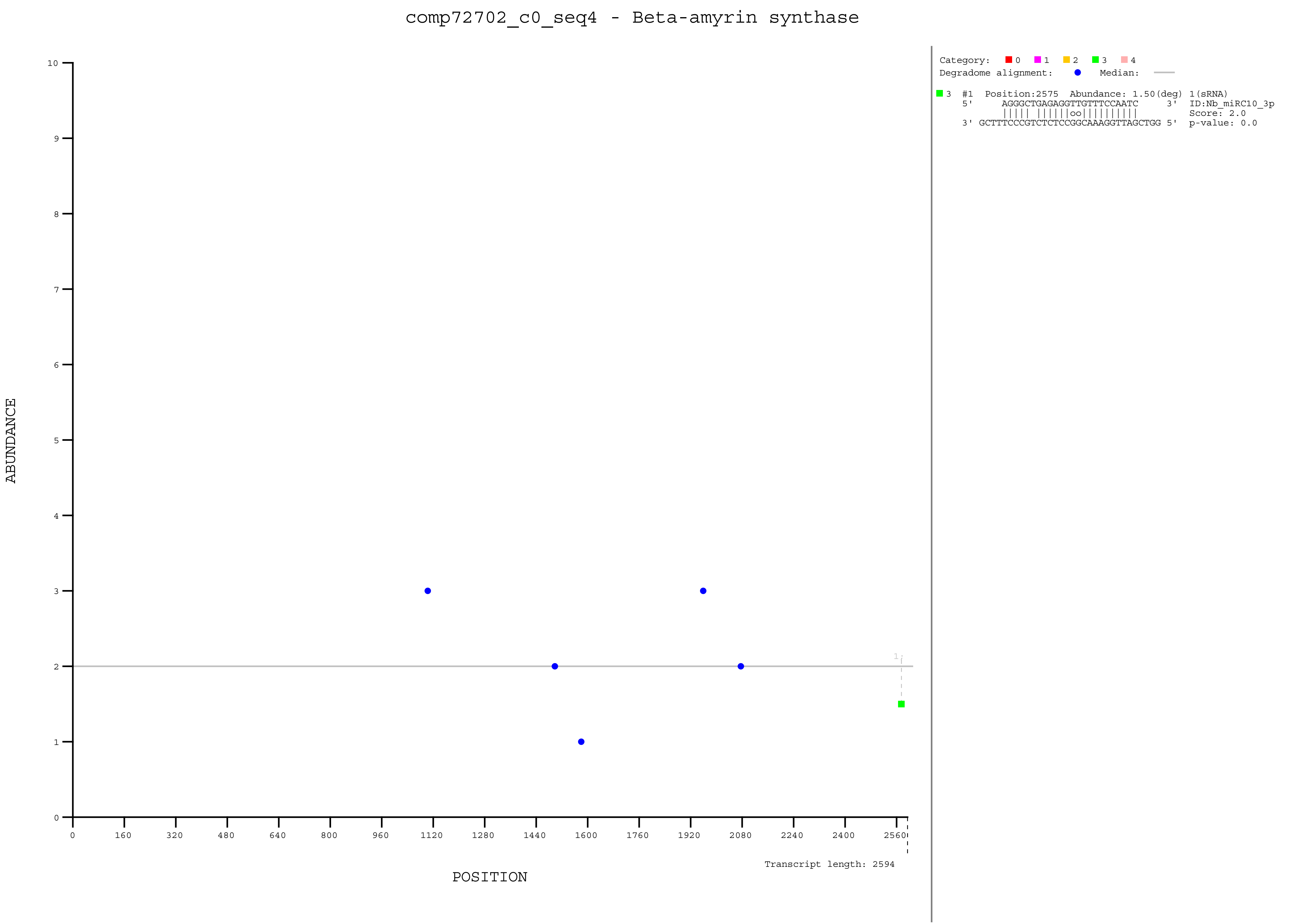
comp72702_c0_seq4 - Beta-amyrin synthase
ABUNDANCE
10
9
8
7
6
5
4
3
2
1
0
0
160
320
480
640
800
960
1120
1280
1440
1600
1760
1920
2080
2240
2400
2560
POSITION
Transcript length: 2594
1
Category: 0 1 2 3 4
Degradome alignment:
Median:
3 #1 Position:2575 Abundance: 1.50(deg) 1(sRNA)
5' AGGGCTGAGAGGTTGTTTCCAATC 3' ID:Nb_miRC10_3p
||||| ||||||oo|||||||||| Score: 2.0
3' GCTTTCCCGTCTCTCCGGCAAAGGTTAGCTGG 5' p-value: 0.0

comp82078_c0_seq1 - Threonine deaminase

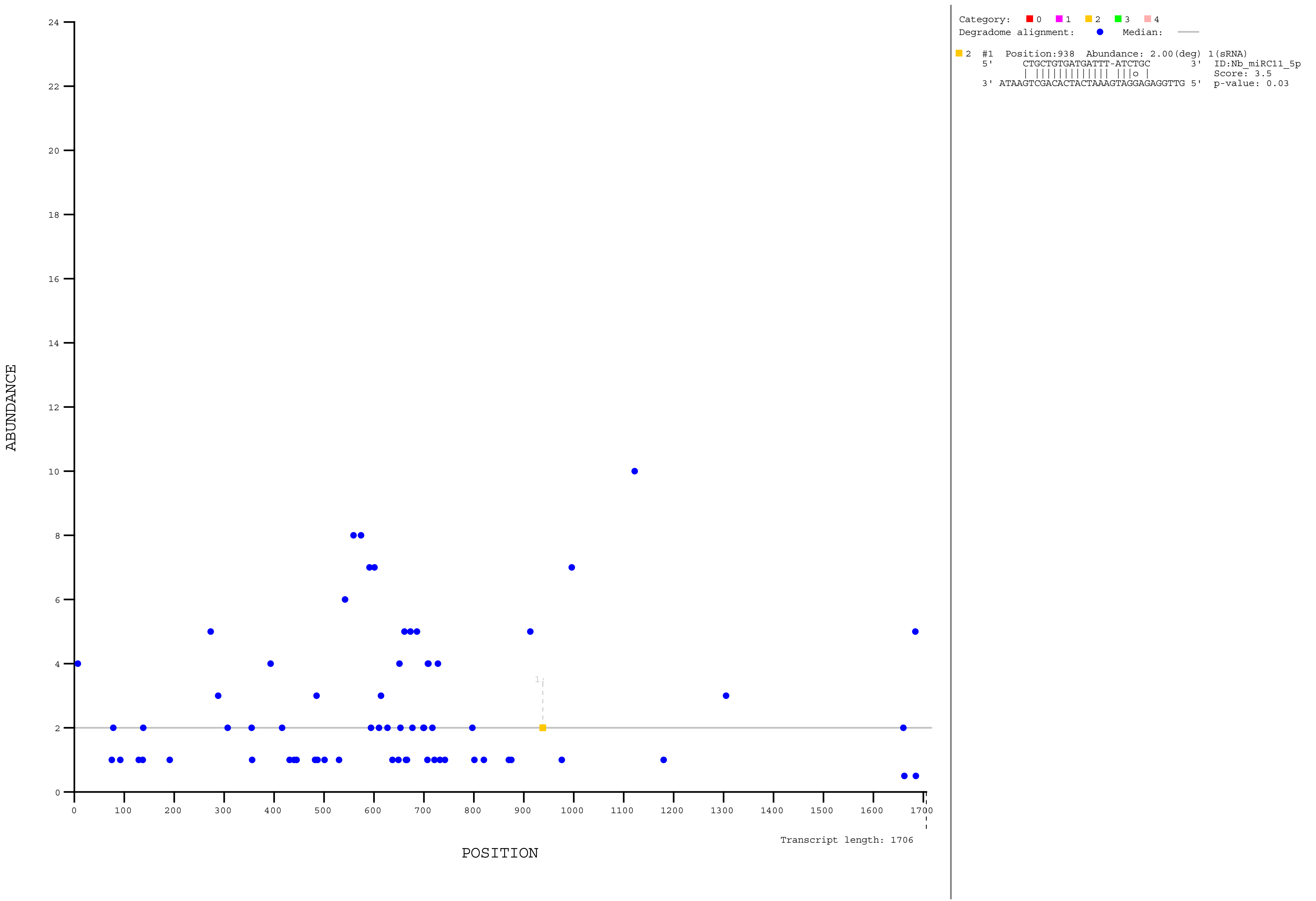

comp78978_c0_seq3 - Transcription factor RF2a

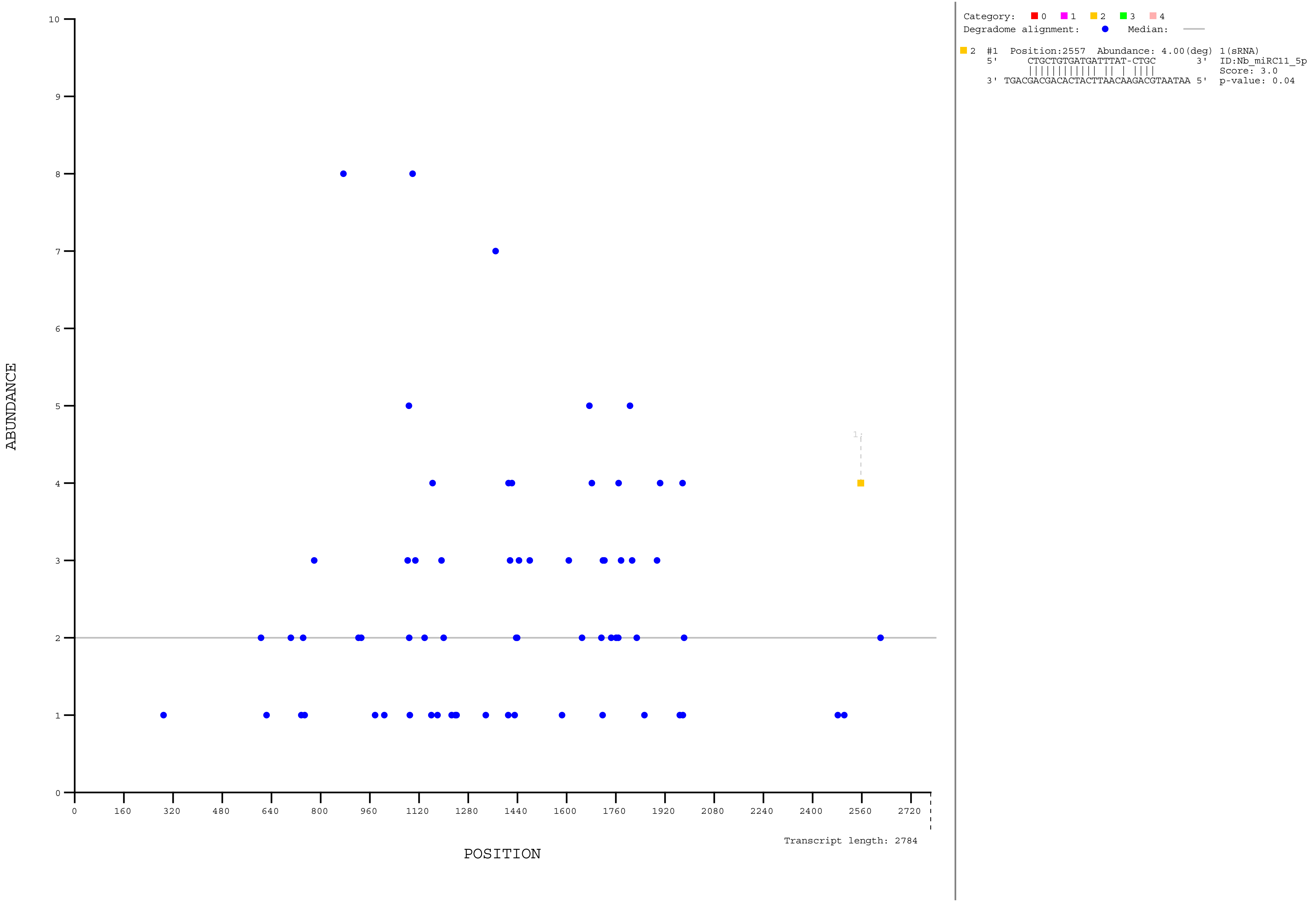

comp89465_c0_seq1 - Ervatamin-B-like

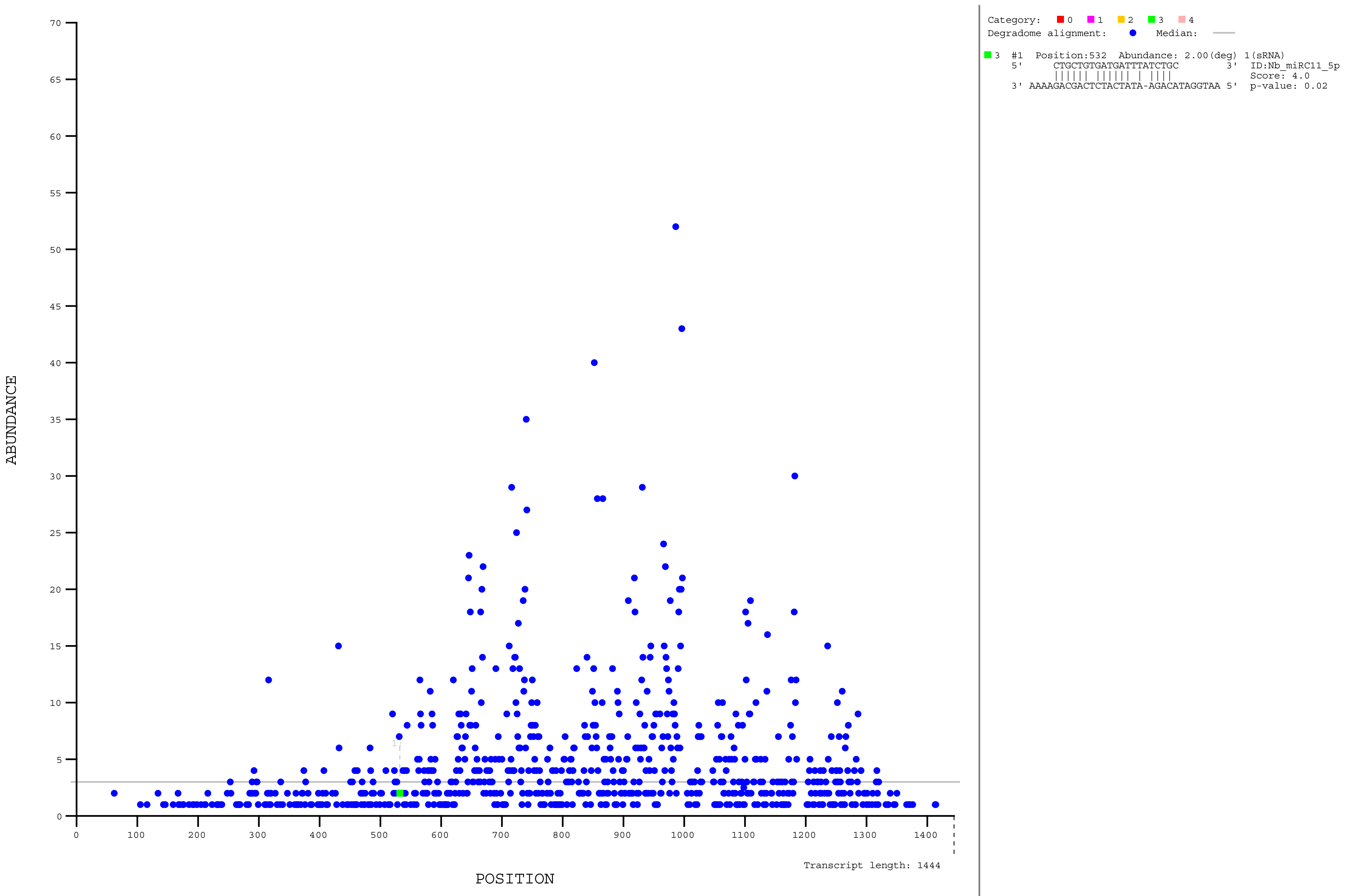

comp79295_c0_seq1 - Probably inactive leucine-rich repeat receptor-like protein kinase At3g28040

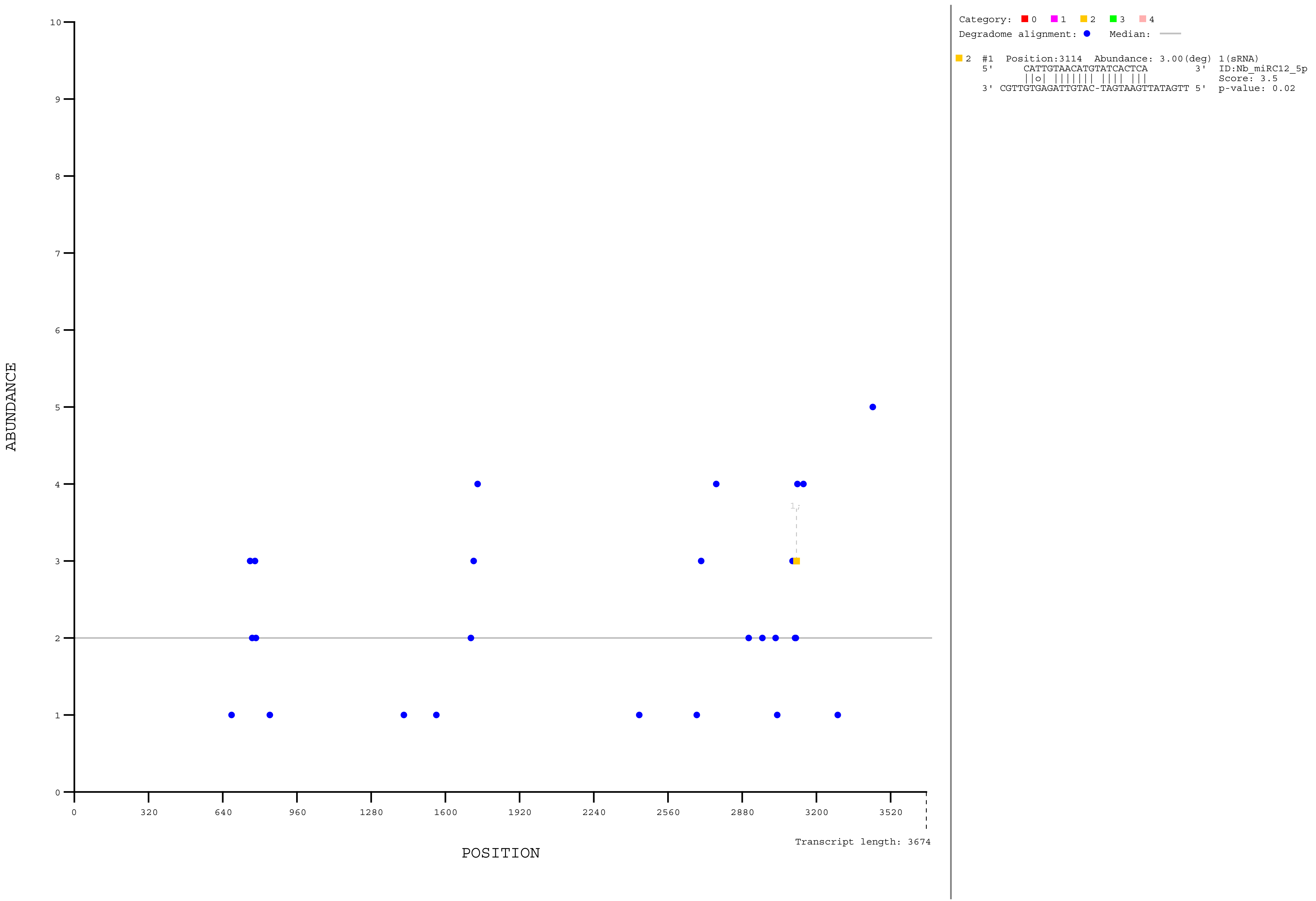

comp56752_c0_seq1 - ZRT/IRT-like protein 1

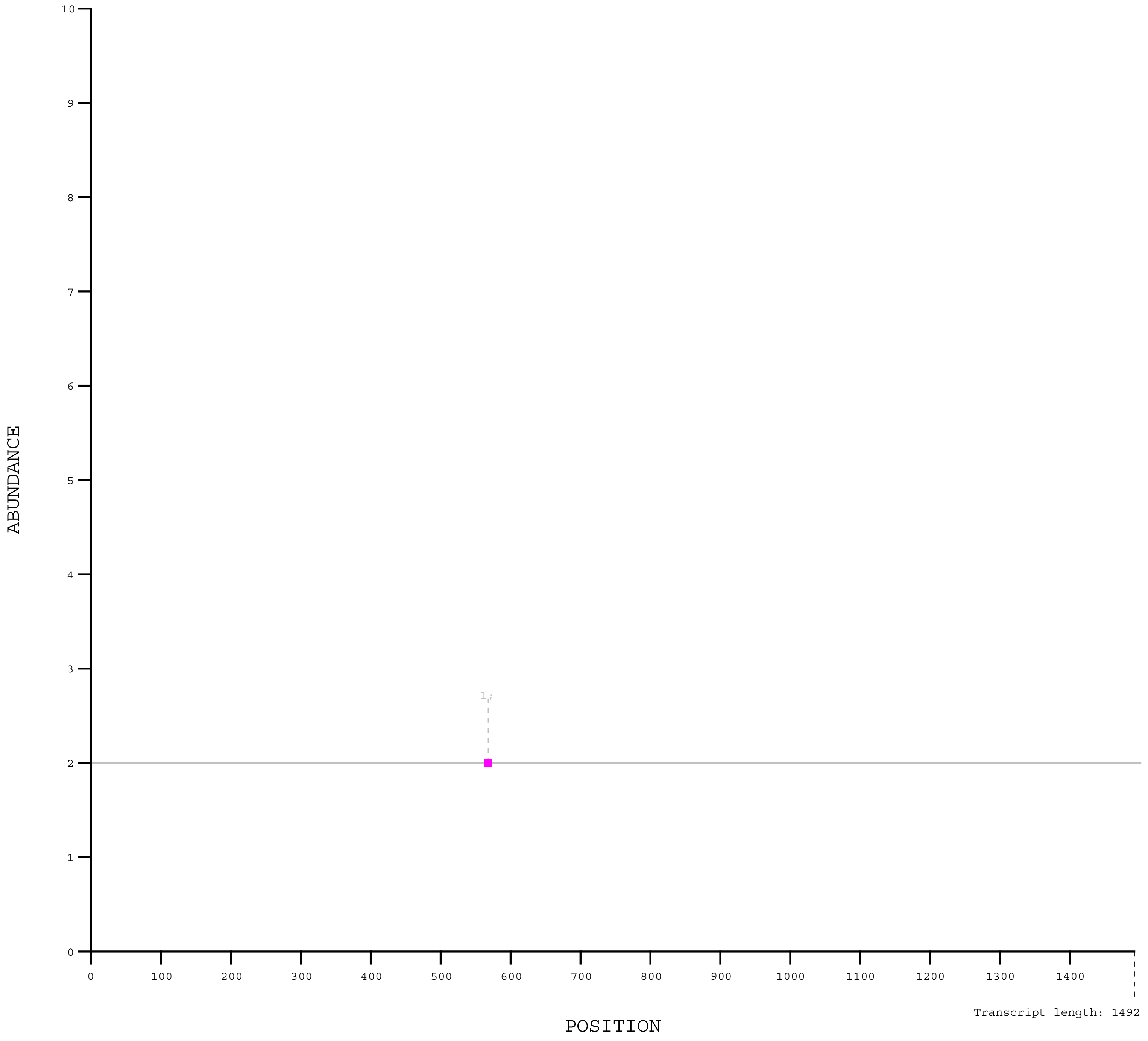


```
Category: 0  1  2  3  4
Degradome alignment:    Median:

1  #1  Position:568  Abundance: 2.00(deg) 1(sRNA)
   5'     CATTGTAACATGTATCACTCA        3'  ID:Nb_miRC12_5p
          | |||o|| |||||||| |||            Score: 3.5
   3' TTGAGCAACGTT-TACATAGTTAGTAGTTTGT 5'  p-value: 0.0
```

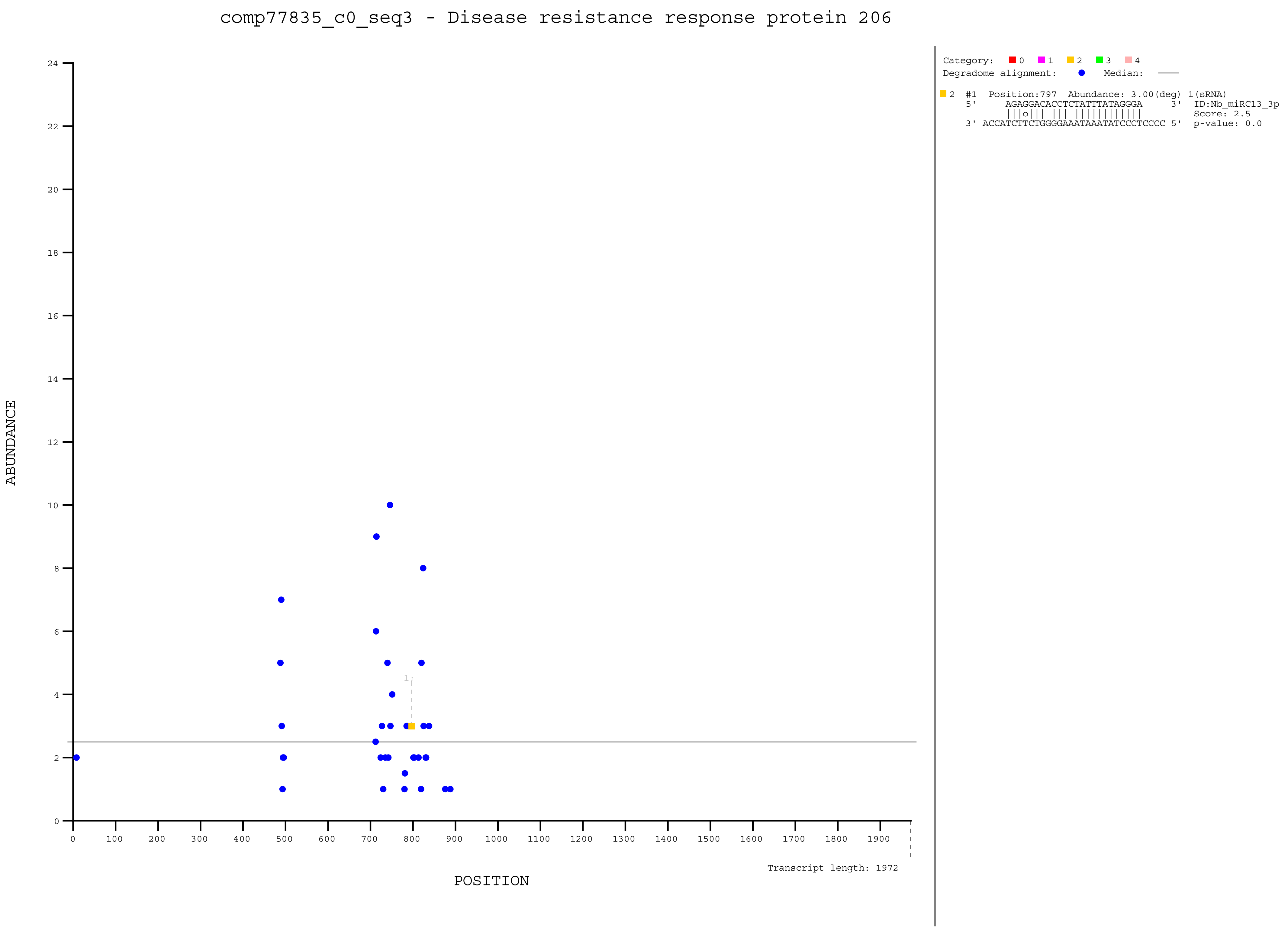

comp77835_c0_seq3 - Disease resistance response protein 206
ABUNDANCE
POSITION
Transcript length: 1972
Category: 0 1 2 3 4
Degradome alignment:
Median:
2 #1 Position:797 Abundance: 3.00(deg) 1(sRNA)
5' AGAGGACACCTCTATTTATAGGGA 3' ID:Nb_miRC13_3p
|||o||| ||| ||||||||||||
Score: 2.5
3' ACCATCTTCTGGGGAAATAAATATCCCTCCCC 5' p-value: 0.0

comp74263_c0_seq1 - Chaperonin 60 subunit beta 2, chloroplastic

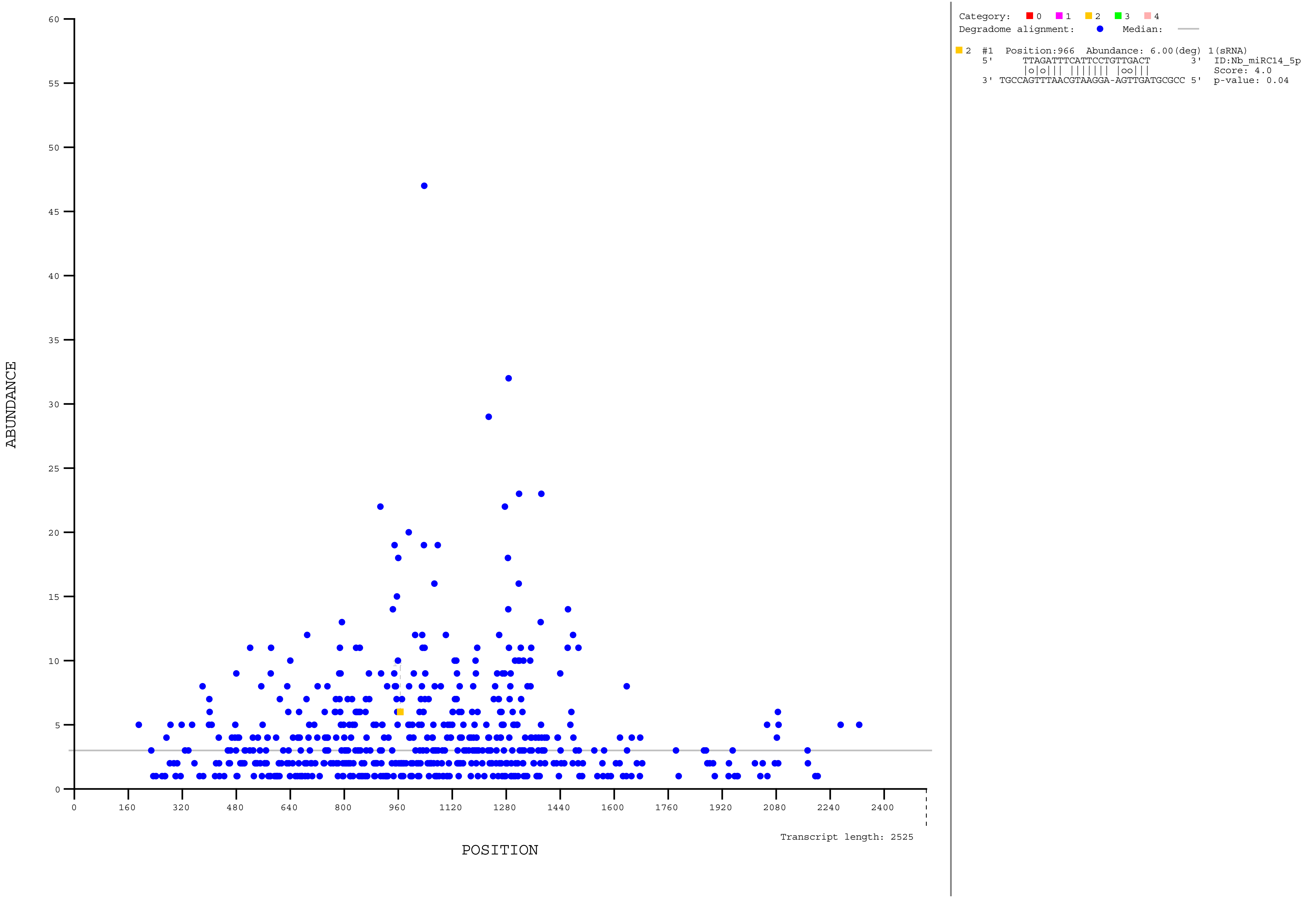

comp78088_c1_seq1 - Peroxisome biogenesis protein 19-1

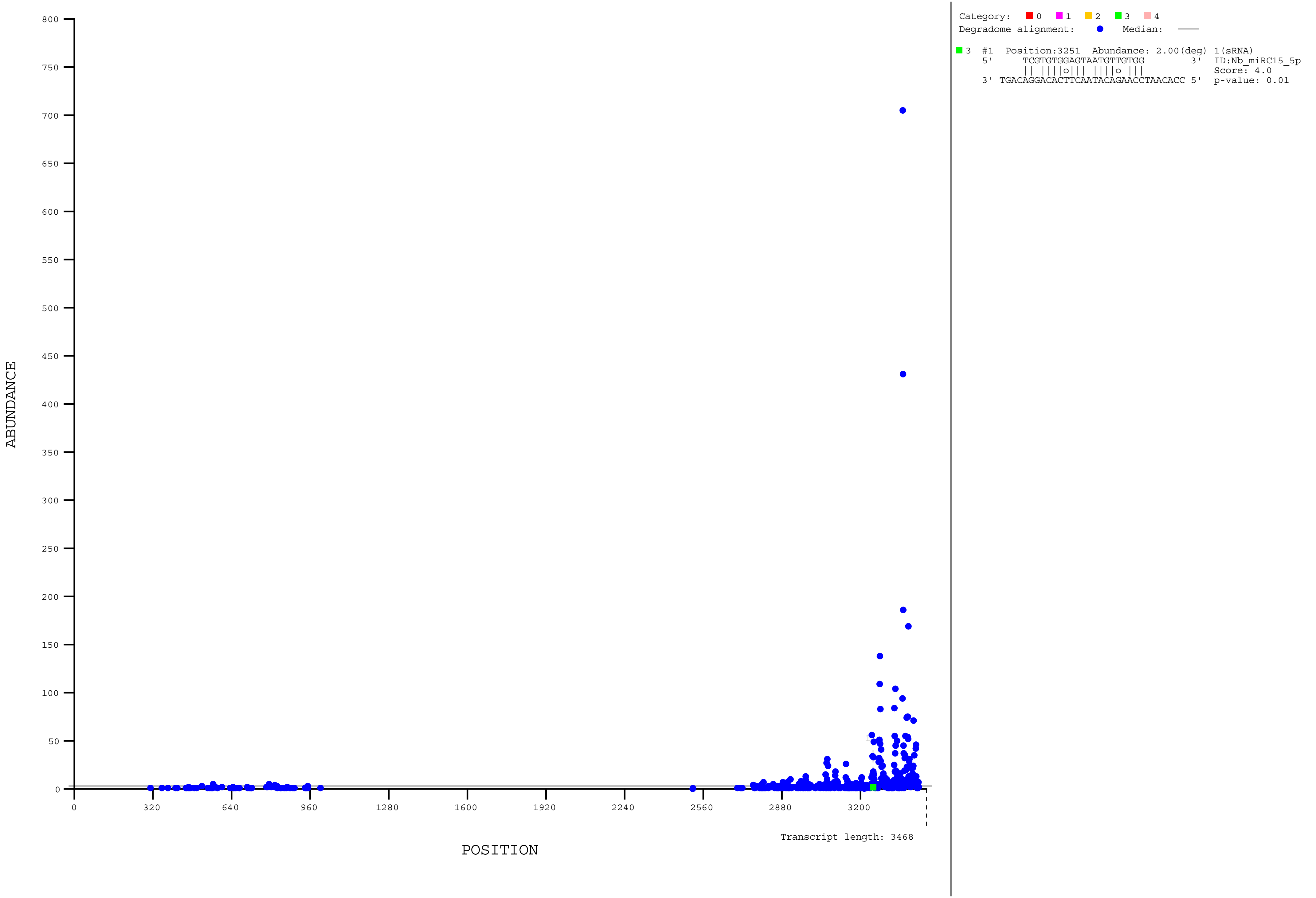

comp85070_c0_seq1 - (E,E)-geranyllinalool synthase

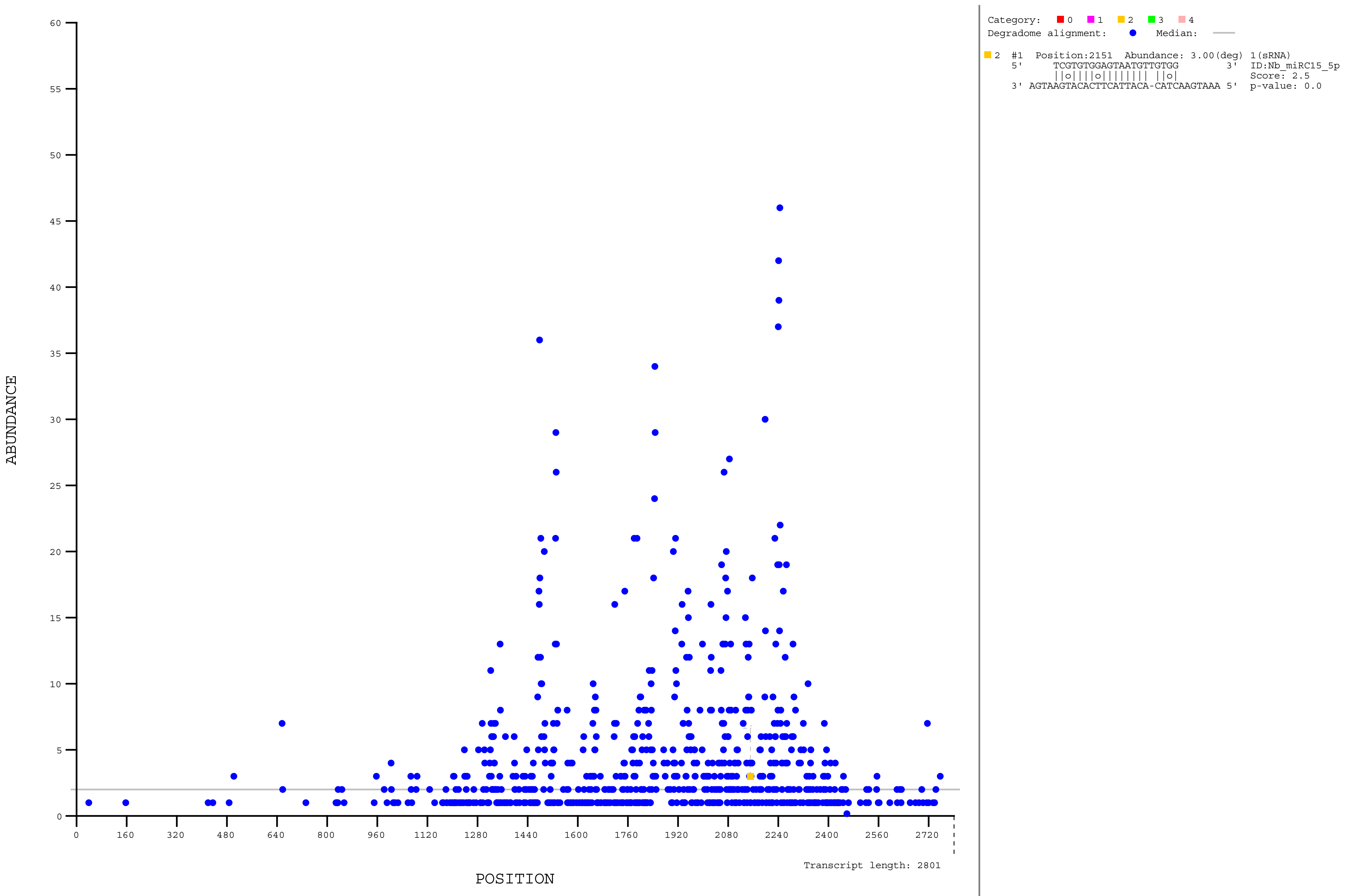

comp88815_c0_seq1 - Polyphenol oxidase, chloroplastic

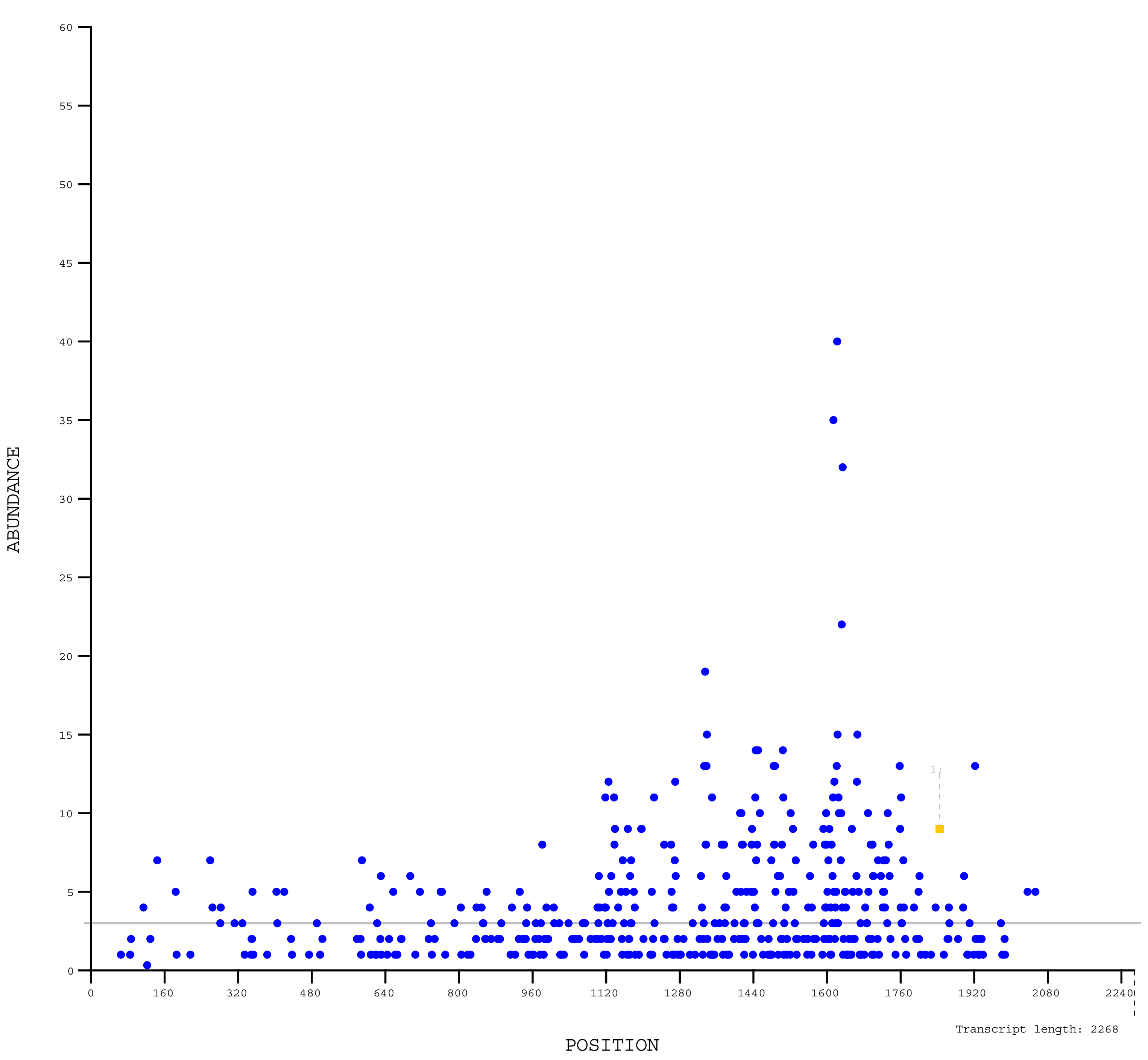

Category: 0 1 2 3 4
Degradome alignment: ● Median:

2 #1 Position:1845 Abundance: 9.00(deg) 1(sRNA)

```
5'     TTTGGGACCAAAGTCACCAAC         3'  ID:Nb_miRC16_3p
       |||||o|| |||||||||| |             Score: 2.5
3' TAAGAAACCTTGTTTTCAGTGGTGGTGTACTA 5'  p-value: 0.01
```

comp63365_c1_seq3 - Heterotrimeric GTP binding protein alpha subunit

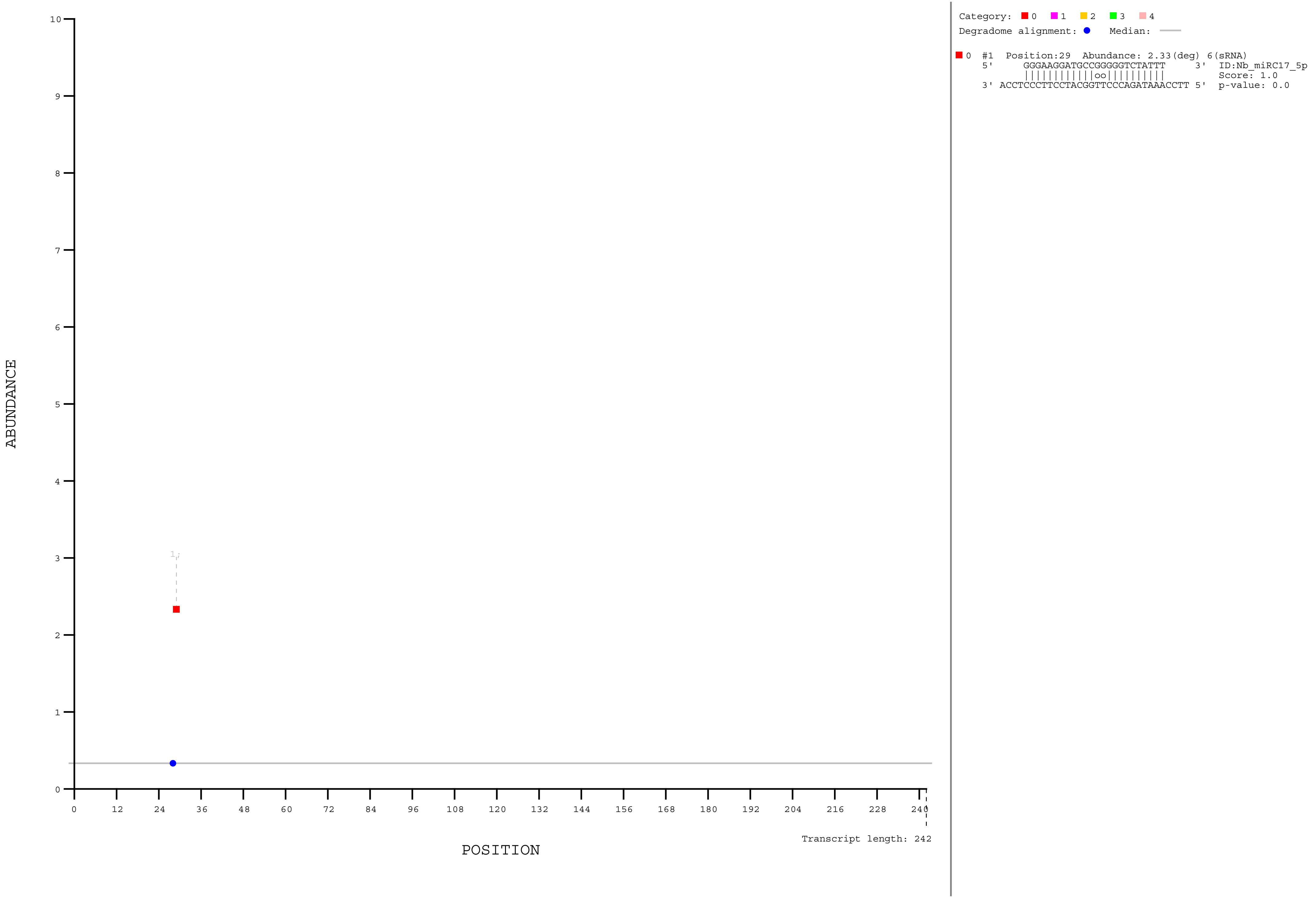

comp67145_c0_seq1 - 50S ribosomal protein L3, chloroplastic

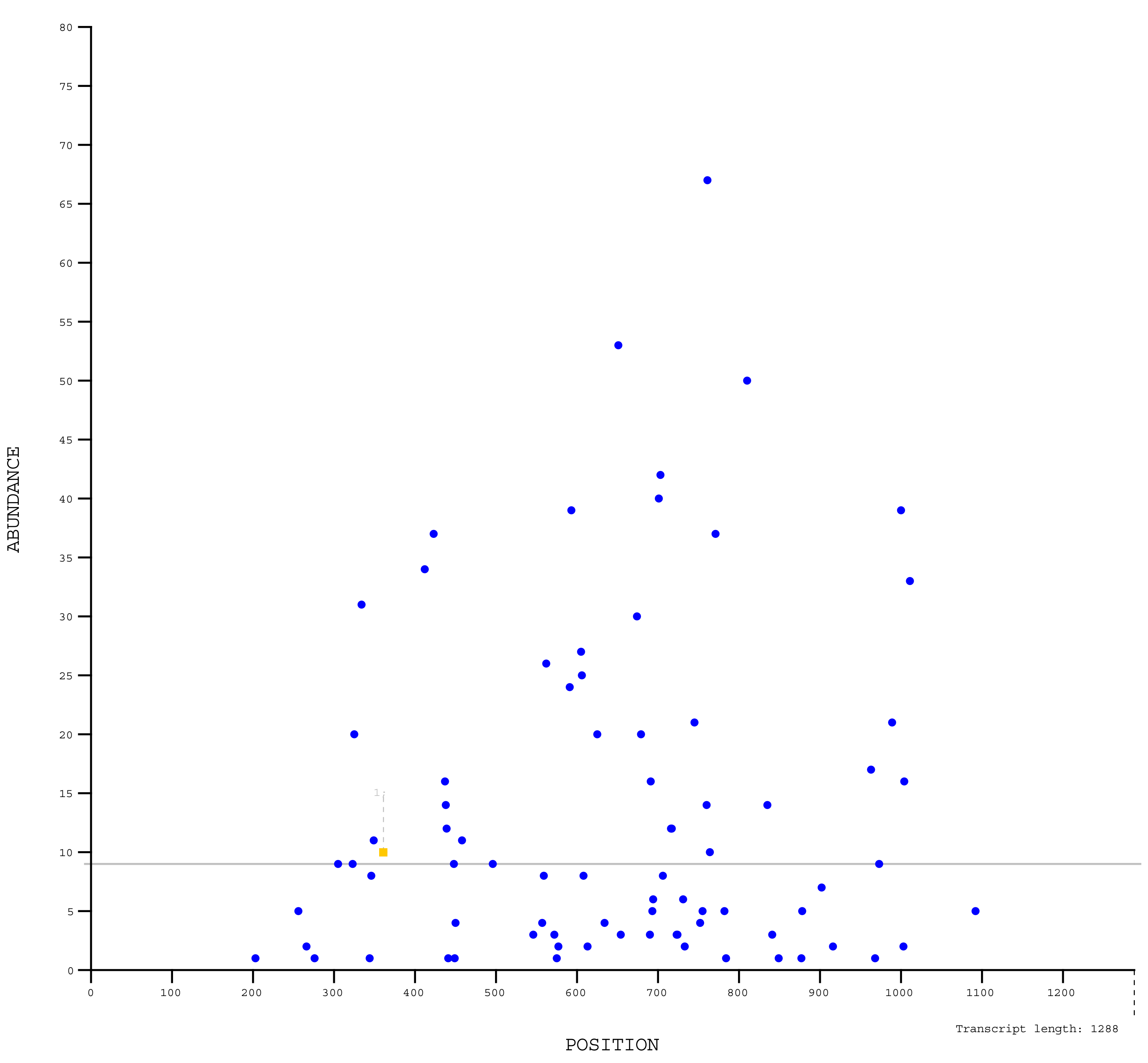


```
Category:  0  1  2  3  4
Degradome alignment:      Median:

2  #1  Position:361  Abundance: 10.00(deg) 1(sRNA)
   5'     TTTTCGGAAACCTAATAACTT        3'  ID:Nb_miRC18_3p
          ||o|| ||||||o||o||||             Score: 3.5
   3' ACGGAAGAG-CTTTGGGTTGTTGACAATGTCC 5'  p-value: 0.01
```
