# Supplementary material for: Identification of Nicotiana benthamiana microRNAs and their targets using high throughput sequencing and degradome analysis
Source: BMC Genomics. 2015 Dec 1;16:1025. doi: 10.1186/s12864-015-2209-6 (PMC4667520; doi:10.1186/s12864-015-2209-6)
Supplement: Additional file 11: — This file lists the complete mRNA sequences of miRNA targets found in our samples in fasta formats. (PDF 416 kb) [file 12864_2015_2209_MOESM11_ESM.pdf]

Additional File 1.

```
>comp77119_c0_seq6 2667_2942
AGAAACAGGAAAGTAGCTACAAGTGGCTTTATCTCATTCTATCTCTTACAAGTTACAATG
GAGAGAGACAGCCATATACTATTCTATGAACTGCTACCCCAAAAATTATAATGCCACATC
TCTTTCACTGAATCATATCTCAAAATTACACACAAAAACTCATCCCCTTGATTTGTTTGT
GGTACACTTAACCCATATCCCCGCATCAGATCACTTCCTTTCTCCTTTCCCAAGTTCTCA
CATTTTTCTGTCATTTTGGCAGCTTTCTATTTTGTATATTTGGACCACTTCTTTTCACTT
ACCAACTGTGTACTACTTTTTCTTGCTATAGCTCCTTTTGCTTCCACTAAGGAAGCAAAT
ATTTAACTTGTCTACTTGCTTCTTCATTGATAACAGTTATTATGGGGTTTCCTCCTCTTT
CTGTTTCTGCTCCTTTCAGTTGTCCTTTTTATAGTGTTTAATAACTTCCCTTTTACTGGG
TTTTCAAGAATATTTTTGCAGCAGTTCTTGAGCTTGTCAAGTGTATTTTTGGTTATGGAG
CTGAAAATTCAAAGTAGTTCCTGTGTGGAAGAGTGAAGCAGCATATTGGGTTTGTATGAA
ATTATTGAAGAAATTATTTAATTCTTGGAAAAATGGAGTCTTGGAGCTATCTTTCAGGGG
GAAAGGGTTTCATATCAGAGGAATCAGTTTCTATAAATGATGGAATGGGGCGAGCTAAAA
ATGGATTAATGGGTTGGGAATTGAAGACCCCTAACAGCTATATGGTGTGCTCAAGTCAAG
AAGGTAGTACTAATAATCAAGGATTTCCTGAATTGGGTTCTCAAAATTTGATGAGAAAAA
CTATGTTCAGTGACCAAAGTTCTTTTGGTTCTGTGGCAGCTAGTCCTAGTGCATATTCTG
GGGAAGATAAGTCCAAGTCAAGTTCCAAGTTATCAAGCTCAGTTGTGGACTTAAACTCTA
GAGATTCATCACTGATTGATTTGAAGCTTGGAAGATTTCCTGATCAAATAGATGCAAATG
TTTACAAATCTCCCAAGATTATGCCTAATGTCTCTTGTGCTGAGTCCATTGTTCCAGCCA
AGAGAATGAGAGCAGGAGGTGTGAATTCCTATCCATTTTGTCAGGTTCAAGGTTGTGGGA
AGGATCTCAGCTCTTGTAAGGATTATCACAAGAGGCATAAAGTTTGTGAGGTTCACTCAA
AGACAGCTAAGGTTATCGTAAACGGCATCGAGCAAAGGTTTTGTCAGCAATGTAGCAGGT
TCCATCTGCTAGCTGAATTTGATGATGGCAAACGAAGCTGCAGGAAACGTCTTGCTGGTC
ATAATGAGCGCCGAAGGAAGCCCCATACTGGTACCAGATTTCGAGGAACTTCTTTTGCGA
CATCATCTTTTGCTTGCCAAGACATACTTGGTGGCCGCTTTCTGCATCAGCCAAAATTTG
AGATGAATGACTGGTATAAGAATGTAAAAGTTGAAGATGGTGTTGAACGTAGCCCCCAAC
TTGCCATACCCTTTTCAAATGAACAGTTGCAACCAAAATCTGTTTTTACGTTATATCATG
CCAAGAAACAATGTCAACCTCTCCATGATGAAGGACAACCTGCTGTAACAACGAGCAGAA
TTAATGAGAATATCATCTCATCTCTGCAAGATATGCGAGGATCAGATTTTGTTTCCCCTT
CATTGTTCCACACCACCTCAACTGGATCTGAAGTCTTGCATGTCTTGGACACATCTTGTA
CTATTCATGGGCTATCGGGGATATCAAACTCCAGCAATGCTCTCTCTCTTCTGTCATCTC
AATCACGGGACTCGTCAAATCATTCCACTGTTTTTCGTGCCGCTCATCCCCTGATATCCC
CAAGCAATAATTCACGTTACAGTCTGACTCAAGCATCTAAAGAAGTCCTGGGACTTAGTC
CTCAGGATTCAGCAGGTAGAATGTCCAACACATTCAATTCATCTGGGGTCAATTCTGCAG
AAGGTGGACTGGACCAGATACTATTTCCTAGCAACACTTGTGGCGTCAATGGGATCATTC
AAGGTCCAAATTTTGTGAACCACAAGGATCAACTTTCTTGTGAAGATGGACCCACTATCG
ATTTGCTCCAGTTGTCATCGCAGCTTCAGCGTGTGGAGCATCAAAGGCTTTCCATGCAAG
TGAAGCAGGGCAGCAACACTTTTCCCGGTCTCAGAATCACTTGAGCAGGTAGAAAAAGGT
TATGTATCACTATCAGAGTTTTGCCCAACTATATCTCATATTTTTTATGGTTATTGAATT
ACCTTTCTCTTTTAGCTTCAATAACTATTGCCTTGCTGTTATTTTTGGCATAAGTCTTTG
CCAACTGGATGAAATATCACTTTCTTGTCAAGCTACATTCTTAGATTTTCACCTACAATT
ATGTTTCATATTTCTTACTCTATGCACCTCTATGTTACCTGCTAGGACTGTGATGGCGTC
GGATTAATTTTTATTGTCAACTTCCAATAATGGCTGACCGTATCTTGGTATTGGACGTCC
CATATAGATGCTTATCTTGTATGCAAAAGCAGGAGGTCTGTAGATTCTGTTTCAATGCAG
CTGGGATCTAATACTTTTTGTGCATTTTTCTTGTTTTACTCGTTCCATTTTTCTTTTCTG
AGGTCCAGTAAGAGCGTATAATTCTTCTAGTGCCTCCGGCAGATGGTTACTAATCTGTCT
AATAAAGTTGCTAGCATCAATAGTCCACGCAAATGACTACTGCATGAGTAATATTGCCCT
AAATTGAACTTGCTGTCTGCAGATTCAAATATCTGGATTGACGTGAAAATGGTTGGCTGC
TGAACAGCTAAAATGTGCAGCAACTCGGGCAAGAAGCAGCCAGCTTTTCCAGCTACCAAA
GATGGGAGCGTTGAGAAGCAAGTCTTTGGAATCTTGATGTTCATTTCTTCATTGTTGTTC
ATCTCTTTCTCATTTTGATATGGACAACTGTTCCTCCCGACACTGTCTTATTCAATAATA
ATCTGTTTTTCTTTCGGATAATTGACAAAAACAACCCTGATTCGTAGTCAAGTTGGTTTT
GTGTACGGAATTATCCACCCTCTTTGTAGTGTTATGTGCTTCATTTCTTTGGTATGAACT
AGTTGCTCGGGGTACTTCATGAACAATTTGATGAATTGCATGATTTGTGTGCAAAAAAAA
AA
```

>comp69399_c0_seq4 393_944
TGTCAGCAATGCAGCAGGTTTCATTCCTTGGTAGAGTTTGATGATGGAAAGAGAAGCTGC
CGGAAACGTCTTGATGGACATAACAGGCGTCGAAGGAAACCTCAGCCTGATTCTATGGCC
AGAACTTCTGGATTACTTTTTACCGGCCAGCAAGGTTAGGGTCATTCCTTAATAATCAGA
ATGCTTTTCTATATGAAAGGAAACATGAGTCTTTTTCCCACCTTATTCTTAGAAATTGGC
TAACATTATTTTTACATACCCTTAGTTTTTTATGTTGATTCAAAGAAAACTCAACTGATT
TATTTGTAGGGACGAAACTTCTGTCATTTAGCAGTCAACAAATTTTTCCAAGTGCAGCTG
CATGGGCTGGCGTAGTCAAAACGGACAGCGACATGGTATTATACAACAACCAATCGCATA
TAAATTGCATGGACAATCAAAACTCGTTCCCTGATTCTTCAGCTTGTAGCTACAAAGGAG
GAAATCAATTCCAGTTCATGCAAGGCAGTGACCATAGTCTCCCTGAAGCTTCAATCTGCC
AGCCACTTGTTGATCATGCCAATTCTGCAGCAGCAGCAGTTTCTAGCAGCGGACAAAAGA
TCTTCTCCAGTGGATTGAACGAAATTGTGGACTCTGATCGTGCTCTCTCTCTTCTGTCAT
CAGCACCCGCTGTAACTAGGGAGATTGGTTTTAGTCACATGGTGCAGCAGCCTGCTTCTA
TCCCCCGGCCCCAGTCACAATCCGTCGCTCATAGCCTACACTATGGTGGTCTAAGCCAGT
ACCCTTTTGCTCAAGATTTCAATAGCCAACCTCAAGATTCTGCTGCAGATTCACATGTTA
GCAACAACAGCTCTCTGCATTTCCATGATATGTTACAAAATGGACCAGATGGATCATCTA
CAAGTGCTGGCTGTGAGCAAACACTAGCCTTTATGTGGGACTAAAAAAACTTTCAGGATA
TCTAAATTTGGTCTCTGTTAACATCAAGAAAATTCCTTCCAAGCAAATGTTATTCCCATT
AAGAATGTTTAGCTTCTAATTAGCGACAATTTCTTCTCTAAAAGGGTCGAATGTCAGTAA
AATCCTATCATGAAGTCATTTGTGACATAGGTTGACTTTCGTGTGAAATATGAGTTGGCC
ATTCTATATTCCCTTCATTATATTTCTCTCTTCTCGTATACTTTTATTCTCTCAATTGAG
ATGCCGAATTTTGCATCCTAATCCATAAACTTTCTGACACGGGATGTGGAGCCAAAATTT
G

>comp71582_c0_seq2 1_1230
GGGTGGAGGTGGTGGTGGTGGTGGGGTGGTTCAAGTGGTAGTGGCGGTGGTGTTAGTAGT
AGAGCCTCAAAAAAGTTGAAGCAAAAAAAAGTCCCACAAAGAGGACCTGGTGTTGCTCAA
CTTGAGAGGATGTTAGAAGAACAACATAAAAAAGATTCAATTTTGACACCAAATTTAGTG
CATTCAACCAATACTATGTCCAAAAACTTAGCAGTTGAATGTCCTAGTTTTAGGCCTACA
ATCCCTTTACCACCACCTTCAAAAGTGGATCTACGACCTTCGAAAAGTTGTGTCCTTAGA
CCAAATCCTTCTGGTCCTGTTATTGATGGTTTTCGTCCGAAGACTTTACAATCATCAAAA
CCATTGAATAGGAGTGGATGTGAGTTAAGTTGGTCATCTGTTTTGAGTCCAGGAAATGAT
AATTCCCCTAAGTTGTGGAGTTGTGAGTACAGTCCTGAAGGAGAAAGCCAACAGGGAAAT
CACAATGGTGTTGTTTTCGGGCCAAATGTAAATTTGCCTAATGAACTGCATCATCCCATT
TTGCCTCTACCAAGTGTGCTGCAAAGGTCACAACAATATCAGCAACCTTCTTGTTCTTCA
TCAATGGTGAATATCTCATCAGGGATTTCATCATCATCATCATCTGTATTAAATTATCAG
ATGGAGCCCCCTTCAAACCAAAATTATTATTGCTGTAGTTATTTGCCTCTGTGGCCTGAA
GAAGTGAAGATGGTTGGCATGAAAAGGCCATGTCCCTTCTCTCCAGAGTATCCACCAGTC
CCTGCATTTCACTGCAAGTTTCCTCCGGGTTCTGTTAGCCTTGCATCCAGATCGCCCGAA
TCAGCTTCCTGCAGCAATGAATGCACAGCAAGTTTAGAATCAGGCAATCGACTTAAAAGA
GAAGTTCCTTCAGGATCACGATCTATATCCGAGTCCAAACCAAGGAATGTCTTCAGACAA
AATAGAGCTCTTAATGGAGATTTTCTCACGTTGGCTCCTCCTTCAACTGCTTCACCATAT
CAGAGTCAAGAGACACATACATTGGAATCCACAAACTGTCAGGGAGTTGCTGAAGAGCCA
ACGACATGTTCAGGTTTAATCAGATCAGTTCAACAACCCATCTTCGGATTATTCCCATCT
GCAAAAGTACAACTTGGCCAAGAAGGAACGAACAAAAGCAACTGCCACGCTGAAGTAGGA
GAAACCGTTGATCTCAATCTGAAGCTATAGAAGTTAGTCAATTTACGCTTGGTGCTTTTG
CCAGGAGAATGTCTTATAACATCTGTTTGAGAGATGGCTCCTTCAAGTCTTAAGGTGCTT
CTGCAGGGTTTTTATTCCTCTGTGCGGTAGAATAACATTCTTTCTGATGAATGTCTTCAC
CATTTGTTGGGTTAATTTGTAGTTAAATTGCATATACAAGCTTGAGCATGAAAAAAATAT
GCAAAGATCTTGGCCGCATGCGCATGTTAATATGCAAAGTCATGTTGGAGAGGTGGGGAT
TAGCCACTCGGTCTATGACGATGAGAAGTGAGATTTTTCAAGTGGAACGACTTAGTCGTT
CTGGTTCCATTGATTTGTATGTCTCATTTTCTGTAAACTGTTAGTATGTTATGCATGGCG
CGTTGATTCATTTGGCTATTAAGGTTTACTTATCTCAAGTCACACAAGGCAAATGAAATG
TGTAGGATTCTGGAATAATACTTGTTCAAAATAGCAGTTCAATAATATGTCTTGCACTTG
CTGGGTCTAGGAACGCTGGTTTTATCCCTAGGATTAGATCAATGGTGTATACAGTATCTT
AAGCGATCATGTCAGCTAAGTGTCACTACTCAGCCGGCTTGTAAGTTTTGGTCTAAGAAC
TAACTAATGCTTTGCTATCTGTCTCAATTTTGATATTTACTTTATATTTTGC

>comp69815_c0_seq4 1_894
CGCACAGATAATGAGATAAAGAACTACTGGAACACCAGAATAAAGAAACGACGGTGTGTA
GGCTTGCAAATATATCCTCCGGATATTTGTTTCCAGGCATTTAGTGAGAACAAACAAAAT
AACGAGCTGGGAATTTTCTCCTCTGCAAATTCACATCCTGATTTCTTGCCTATTAACGGT
TTTGAGATTCCAACTGTGGAATTCAAAAAATTGGAACCTAGTCAGCAGTTGTATCCTCGG
GCACTTGTTAATATTGCTGCTAGTAGCTTTTTTGATATTCCTGCAACTAGCCTGCTGGCT
CAGGCTCTTAGTTCTTCTAATACTTGTTCTGGCCTCTCAACAATGCATCCATCCAAGCTT
ATACGAGGATCAGAATCTTGGTTCTTTGGTATGACTGATGATCTTTTTCAAGCTTGCCAT
CAATATCAAAATAATAGTTCTTTGTTTGCTCAATCCTTGGGGTTTTCCTCGTCATATACA
CATAATCTGACATCTGATCATCACCCATCATTCTCCGGTGAAATTCCTGGCAGCCATTCC
CCTTTAAATGGCAACTCTTCTTCTTCAGAGCTCAAATGGGCTAAGAAGCTGGAGCTCCCT
TCACTCCAAATTCAGATGGCAAATTGGGGTTCACCTTCCCCACTTCCTTCACTTGAGTCC
TTCGATACTTTGATTCAATACCCTCCAAATGAGCATACTGTTTCGGGCAGTCTGTCACCT
CGGAACAGTGGTCTATTGGACGCTGTACTTTATGAATCACAAACATCCAAAGCTTCAAAG
CATAATTCACATCAGGAGACTTCTTGTGATGTAGTGGATGATTCATGTCCTGATCTCCAA
GAGACACAGTGGGGAACTTACAGTAACCCAATCTCTCCTTTAGGTCATTCT

>comp73942_c0_seq1 927_2375
GTTCTTATTGGTGTTAGACTGTTAGTGAAGTCTGTAATTTCATGTTAGGAGCTCCTATTA
CATTAATTAAGAGATCTTTCACATTCATTTTGAGCTTTGCATTTATTAATTATGGTGTAA
ACATCGCGTGCAAGTAAGTCTTTTTAATGATTTTTTGGCTGAGGTCATGCTGTGCAATGC
AATTAACTGGGCATTTTTGTGCATTTTTCATTAGCAACTAAATCATTGAATTTCTTGTAT
GCTGATTTCGAAGCTAAATGATTGGTCGATACTGTTGGCGACTGCTTTGTTTTATTTATT
ATTTTTAGACGTTAGCGGCTTCTTTGTTCTATTCGACAACTATGACGTTTATGTTTATTT
GCATTGCTCAAATTGTTTAAGTTGTTTTATACACCTAGGATTGAGCTCAGTGAATTTCTC
TGTGGACTTGTATATATACCCTTTTCCTTCTTCGATTTACTTCATTTTACCCCCAAGATT
TGGTTGTGTCATGTTATTTTGGTAGTTACTAGTTAGATGAGATATTGTGGTCCGGGAATC
CAGAATAGATTATCCTTTTTCATCATTTATCTTTTTACCTTTTTCGTTATTGTGTGAGAA
TGTATGTCAATCTTGCTTTCTGAAGTTGGCGGTTCATTTTTATCCATCGCATTCTGTAAT
TTCAGCACTTGCTTTATTTTGAATAAAAATCTTACTTAAAGCCAAAAAGGAAAATAAAGA
AATAAAAAAGGTTGGCTGTTTCTTTAGTAACCTCGATTGGCTCGTCATGAATTGTGTCAC
TCCTTTTGGACATCTCGCCTTTATTTCTCCCTTGACTTTTATAACAAACAATTTCACATC
TCCTTTCATTGGCAGTTACATGCTATTTTTTATTTATTATCTTTTCAGGCTAAACTATTG
GGACGAACTTTTTAACATTTTAGGATATGAGCATCACTTTTGAAACCGATGACATGATGA
CATCCAAAGTAGACGTGGATTCGCCCGACGAAGCTACTGGTGGAGTAAATGCCGGTGGAA
GTGAACCACTTAAAAAAGGGCCCTGGACTTCCGCGGAAGATGCAATTTTAATGGATTACG
TCATGAAACATGGCGAGGGGAACTGGAATGCTGTCCAGAAGCAATCAGGACTTGCTCGTT
GTGGTAAAAGTTGTCGTTTGCGGTGGGCAAATCACCTGAGACCAGATTTAAAGAAAGGTG
CATTTACTCCGGAGGAAGAGCGGCGGATAGTTGAGTTGCATGCTAAGATGGGAAACAAAT
GGGCACGAATGGCTGTTGAGTTGCCTGGCCGCACAGATAATGAAATAAAGAACTACTGGA
ACACCAGGATAAAGCGACATCAGCGTGCCGGCTTGCCAGTTTACCCTCCAGATATTTGTT
TCTTGGCAAGTCAGAACAAACAAAATGAGGAGTTGAGTGCATTCTCCTCTGTAGATGCAC
AAAATCCTGATGTCTCGGGAACTAACAATTTTGATATTCCTGCTGTAGAGTTCAAAAATT
TGGAACTCAATCGGCCGTTATATCCGCCACCACTCTTTGAACTTCCTTCTAATAGCCTGC
TCGATATTACTGTAAGTGGCTTTCTTGCCCAGGGTCATAGTCCTCCCTATAATAGTACGT
CTTTCCTTTCGACAATGCATCCGTCAAAGCGTATACGAGGATCAGAATCCATGTTCCTCA
GTTCAAATGGTGGTTCTTTGCTTGGTCAGCCCTTGGGGTTTTCTTCATATAATAATCATA
ATGTAACATATGATGATCACGGACCATTCTCGAGTATTGTAATTCCGGGCAGCCATGCCC
CTTTAAATGGCAATTCCTCTTCTCCAGAGGAGCTCCCTTCACTCCAAAATCAGACGGCGA
ATTGGGGCCCACCATCTTCCCCTTTTCCTTCACTAGAGTCCGTTGATACTCTAATTCAGT
CACCTCCAACTGGACACAGTGAATCGGGTAGTGATCTGTCGCCTAGGAACAGTGGTTTAC
TGGATGCTATGCTTTATGGATCACAAACTATGAAAGCTTCAACCGATAACTCACACCAAG
AGAATGAGACGTTTGGTGATGCAGTCGATAATTATTATGGCGATCCACTCTCTCCTTTGA
GTCATTTCACTGCATCAGCATTTAGTGAACATGCCCCTATTGACGGAAGCTCATTACATG
AGTTCCCGTCATTAGCCACAATGCCAGGTGAGAATGATGAACTTTTTGGCTTCTCCGTAT
TATCTGCATATTTAATTGTAGTGTATTTATACTTCATAATTGGATTATTTGATGATGTTG
AAGGATGCAAAGTTAAGCAAGAGAATGTTGCCACATCATCACGGCAAGCTTGTGGACGCG
ATTCTTGTGCATGGGATGCCATGTCCACTGTGTAGTCGATGGCCTGAACGCGATTATGGT

ATTTTAGAACACCTTTGGGAACATGTTTTTTTGAGACAAAACTTGGCTTTCTCAGTTATT
ATATGACAAGAGAATACTATGTAATATTTTTCATTAACTGGAAATACTTCTGCATGTTCT
GACTTCTGAGTGCATTGAGTTTAGGCAATCTAAAGACAAATACACTGGTACATCTTTTCA
TGTGGCATCAAATTGATTTCAATTTTTATAATATGAGACAGGCTTAGTTAAAAAAAAA

>comp78286_c0_seq3 693_2783
AGGAGCACAAGTATATATATAAGGAGTATAAAGAAGTGTGATAGTGTCAATTTCATAAGC
AATCAGCCTGCCACCCTCTCTCTGGTCGGCAACGAAGCTTCAGGTGACTGTTGAGTGTAT
GTGGTTTGTGTGTCTCTTGTCTCTGTCTGTCCCCATCAACACACTCTGTTCTCTCTATAC
CCCTACACCCACTCCACCCACCCCTCTTTCTCTCTCTCTTTTTACTGTTATTTATGTTGT
CTAAATTCTAAGCAACCTTCCTATTCGTTCATTCTCTTCAAGTAAAAGCACATTTCGTTT
TTCCCTATTGCATTGATCTGATAATATGGAAGCTCTGCTTTGTATTTTCATGTCAAAACC
TTTATTTTGAAGGTAAACTTGTCTTCTACCTTCTCTCTTATCATGCTTTTCCTCTTTTTC
ATTTTCTGAAAAGTAAATGGGGTTTCTCAGGAACAAATAACCAAGACATTTCAAGAGTCT
CTCTATATAAATTATTCCTTCTTTCAATTTCTGGCTTTTCACTAGTCTAAATTGGACAAA
TCCTAAAGAATTTTATCATTTTCCTTCTGTTTCTCTCCCCCTCTTTCCTTGTTTCAAAAC
TCTGATACTTGGAACCTTTTTTGTTGTTACAGAACTGAGATTCGGGAAAAAGTTAGCATT
TTTATGTGTTTGGAGGTTAAGAATAAGGGAGAATGATTAATATTATGAATCCAGTGAACA
AACCTATGAATGAAGTGGAGAAAGGATTGGATCCTCAGTTATGGCATGCCTGTGCTGGTG
GTATGGTCCAAATGCCACCACTGAACTCAAAAGCCTTTTACTTTCCTCAGGGACATGCTG
AACATGCCCACAAAAATGTTGATTTTGGGGGTTTCCCTAAAATTCCACCACTTATTCTTT
GTAGAGTTTCATCCATTAGGTACTTGGCTGATACTGAGACTGATGAGGTCTATGCCAAGA
TAAGATTGGTTCCATTAAAAGGCAACGAATGTGATGTTGAAGATGATGCTGGATTTCTTG
GGCTTGATAAGAATGAGAATCAAGAGAAACCTAATTCATTTGCCAAGACATTGACACAGT
CTGATGCTAACAATGGTGGGGGGTTTTCTGTGCCAAGATATTGCGCTGAAACTATATTTC
CACGGCTGGATTACTCGGCTGAGCCTCCTGTCCAGACCATTTTGGCTAAGGATGTTCATG
GGGAGGTTTGGAAGTTTAGGCATATTTATAGGGGGACACCAAGGCGGCATCTTTTAACGA
CTGGATGGAGCAATTTTGTGAACCAGAAAAAGCTTGTTGCAGGGGATTCAATTGTGTTCT
TGAGGGCGGAAAATGGGGATCTTTGTGTTGGGATCCGGAGGGCGAAGAGGGGGATTGGTA
GCGGACCGGAGGTGCCCTCCGGATGGAATACTGGACCAGGGAATTATGCCTCTTCACTGT
ATCATGGGTTTCCTGGGTTCTTGAGTGAAGATGAGAAGAAGTTCATCCGAAATGGTCAGG
CAGCAAAGTCTGATGATGGTATAAGGGGAAAAGGTAAAGTTCTAGCTGAATCTGTCATTG
AAGCAGCAAAACTTGCTGCCTGTGGCCAGCCATTTGAGGTGGTTTATTATCCCCGAGCAA
GCACTCCGGAGTTTGTCGTCAAGGCCTCTGCGGTAAGAGCTGCAATGAGAGTACAATGGT
CTTCTGGGATGAGGTTCAAAATGCCTTTCGAGACTGAAGATTCCTCAAGGATAAGCTGGT
TCATGGGGACCATATCTTCTGTTCATGTTGAGGACCCCATCCACTGGCCAAATTCTCCAT
GGCGCCTTCTCCAGGTGCATTGGGATGAGCCAGATTTGCTTCAAAATGTGAAGAGAGTGA
GTCCATGGTTGGTTGAATTGGTTTCAAATATGCCTCCCATCCATCTCTCTCCGTTCTCAC
CCCCACGAAAGAAGTTGAGGCTTCCACAACCCCCTGAATTTCCCATGGTTGGGCAACTCT
CGATGTCTTCACTTCTCAACAACCCTTTAAGCCCCAGCAGCCCTTTGTGTTGTATATCGG
ACAACATTCCTGCAGGCATACAGGGAGCCAGGCATGCTCAGTGTGGATTATCGTCATCAG
ATCTCCACTTCAACAAACTGCAGTCAGGGCTATTGCCATTTGGATTCAAAGAACTCGAAC
GTTCGGTCCCGCCTTCTAGACTTCTTAAAGGCGGCAGCTTCATGACCAGTTTTGACAATG
ATGAGAATATCTCTTGTCTATTAACCATGGGTAATACCTCTCCCACTTCAATGAAACGTA
AGGAAACACAAACACCGACGTTCATGTTATTTGGTAAACCAATTCTTACCGAGCAGCAGA
TGTCCCTGAGCTGCTCCAGTGAAACAGTTGGCAACAGTTCATCTAATGGGAATCCGGAGA
AGATGATGAATCTCTCCGAGGGTTCAGGATCAGCTGTTATTCATAATGGTCCACCAGAAA
ATTCTTCAGGTGAGGTACTTCCTTGGTATAAAGATCAGAAACTTGAATTTAGAATGGAGA
CCGGTCACTGCAAGGTTTTCATGGAATCCGAGGATGTAGGTCGAACACTTGACCTTTCAG
TCCTTGGCTCATATGAAGAATTGTATAAAAAGCTAGCTGACATGTTTGGTGTGGGAAGAT
CAGAGATGCTAAGCAATGTGCTTTACCAAGACTTAACTGGTACTGTTAAGCACACTGGAG
ACGAACCGTTCAGTCAGTTTGTGCAGACAGCAAGAAGGCTTACCATCTTAACAGATTCTG
GCAGCGATAACATACGGAGATAGAGACTGATGGATATCGATCCTTGTTAATTGTATAGTT
GATTGCTACAATAGTAGTTTCAACCTACTTCCTTTTCTGCTTTGAGTTGTTTTTAAGAGT
TGAAGGGGAAATCTCTCCAACCTGTGTTTCGCAAATTGTAAACTGAAGACAGATTCAGCT
TTGTTTGATATTTTCAATCTACCTGGATTTCATCTTGCAGCTTTTTTGTTGATCTTACAA
GAATGCAGTTTTTTCATTCAGCTTCTATTGACAATGTAGAAATTAAAATTAGTATATGGA
ACATGATGCTAGATTAATGTGTAAAAAAGAA

>comp79462_c0_seq1 2477_3379
CTCGCTCTTTGTGTGAATATATTTGTTTGCTTTTTGAGACAAAAACATGACAAAGAGTTT
TTTGTAGTGAAAGCCACACTGTAAACCAGTTTTTCACTTTCCATCGCTTTTGATTGATTG
TTAGGGTTTTCTTGTTTTTCTTTTTTTCCCTTGTTGTGTTTCTGTCTGGTGAATTGTGAA
GTGGGGTGCTGTTATTTTGTTCTTGGTGTTGGAGAAACAGTGTAAAGGTGGAATCTTGAG
TTTTTGGAACAAAATTACTGAGTTTCTTGATTTTGGGAAGAAAATTTTGGATTTTTGGGC
TGATTTTGAACTCAAAGTGAGTCAAGAATACTCGTGACCTTAATAATTTTGGCGGAGCAG
GGAATTTTTTTGTGTTTTAATGGATGATTAGGAGGGAATATCCTGTTTTGAAGTGCTCAA
ATTGAAGAAAATGAGGAGTTTAGGGTTCAATTTAGTCAAAGTTGTTTTAAATTATGCGGT
AATTCAACACCCTTTCTAGAGGCCTGAAACTTTCAAGAAAATTTGACTTTTTTAGGACTT
GAATTTTGAGATGCTTTTGCAGAGAGAGTACTATGTACTTTTAATTGGTATCACTGGAGA
TAGATTTCTTTGTTGAACAGATATAGAAAGCATATTTTTCATTGCAAGTGTGAGAACAAG
TCATCCTAATGGGGAAGTAAGTAGTATTTATGAGCTAAAGAGTTGGCTTTGAATTGATTG
TCATATCTGTAAAATTCTGTTGAAATCAAGATGATTACTTTTATGGATCCAAAGGACAAA
GTGAAGGAAATAGATAAGTGCTTAGATTCTCAACTATGGCATGCTTGTGCTGGTAGTATG
GTACAAATGCCCCCAATTAGTTCAAAAGTGTTTTATTTTCCTCAAGGACACTCGGAGCAC
GCCTGTGGAAATGTCGATTTTAGGAGCTCCAACATAAGGATTCCATCTTATGTTCCGTGT
AAAGTCTCGGCTATTAAATATATGGCAGATCCTGAGACTGATGAGGTTTTCGCCAAGATT
AGGTTGATTCCTGTTAGTAAGAATGAAGTTGAATTTGATGATGATGGGGCTGTTGGGATG
AACGGTTCAGATAACCAAGATAAACCTACTTCATTTGCAAAGACATTGACACAATCTGAT
GCAAATAATGGTGGAGGTTTTTCTGTTCCGAGGTATTGTGCAGAGACGATCTTTCCTCGG
TTGGATTACTCTGCGGATCCTCCTGTCCAGACCATCCTTGCTAAAGATGTTCACGGGGAG
ACGTGGAAATTCAGACATATTTACAGGGGTACGCCAAGGCGCCATCTTTTAACAACTGGA
TGGAGTACTTTTGTGAACCATAAAAAGCTTGTTGCTGGCGATTCCATTGTGTTCTTGAGA
GCCGAAAACGGGGACCTTTGCGTAGGCATTAGACGGGCAAAGAGGGGAATTGGAGGTGGA
CCTGAGACTTCTTCTGGCTGGAATCCAGCTGGTGGGAACTGTATGGTACCATACGGAGGG
TTTTCCAGTTTTCTCAGGGAAGACGAGAATAAATTAATGAGAAATGGGGGGAATGGAAAC
AGTAGTGGAAATTTGATGAACAGGGGAAAGGTGAAGGCAGAATCAGTCGTTGAAGCTGCA
AGTTTTGCAGCCAGTGAGCAGCCATTTGAGGTGATCTACTACCCTCGTGCGAGCACTCCA
GAATTCTGCATTAAGGCCTCACTTGTGAAGTCTGCGTTGCAGATCCGTTGGTGCTCAGGG
ATGAGGTTCAAGATGCCTTTTGAAACTGAAGATTCTTCAAGGATAAGCTGGTTCATGGGA
ACTATATCTTCAGTCCAGGTTGTCGATCCTATGCGATGGCCAGATTCACCGTGGAGGCTT
CTTCAGGTAATATGATCTTTGTTTACTGCTTGTAAGCACGCATATTTAGTCTTGTTTCTC
AGTATTTTCTGCCCATTGTTCGATAGGCAGATTCTCATATGATGTGATTCACATGGGTAC
TTTAATATAGTAATAGGACTCTAATCTGTTCAAGAAAAGATACACTTAAATTATGCTGCA
AACATTCTTTCTAGAGTAAATCATATTTGATTTTGTCTTTTATTCAAAATTTCTTTTATA
GGAAAAGAGAAATATGCTTCTGATAAGTTCTATGATCTTGTGAAGTTGGAGATATATCCC
GTATATCGGTGACCGATACTTGGAGTGCACTATCTGTGCAAACTCTGTAATGCACAATTT
TTCTGCTACCTCCTTTTTGGAGTGGTAAAAATGGTTGCTTTTGTACTTAAAATGGTTGCT
TATATTTCCCAAACACGGGTTTGAATTATTACTTGCCAAAGAAGGGGATGGAAGCCGACG
ATTAATCTATTGTTACTTAATTTAATATGTTCCTCTCTTAAGTTGAGATAAATTGCTGCA
GGTGACGTGGGACGAACCTGATCTGCTTCAAAATGTGAAACGTGTCAGCCCATGGCTTGT
GGAGTTGGTCTCGAACATGCCTACCATTCATCTTTCTCCCTTCTCACCCCCACGGAAAAA
ACTAAGATTACCTCAACATCCAGATTTTCCTCTTGATGGCCACCACCTTCCTATGCCGGC
TTTCTCCGGCAACCACCTCCTAGGGCCTAGTAGCCCCTTTGGTTGTCTTCCCGACAACAC
CCCTGCTGGCATGCAGGGAGCCAGGCATGCTCAATATGGTTTATCATTATCAGATATCCA
CTTCAATAAGTTGCATTCTAGTTTGTTTCCTGTTGGTTTTCCGCCACTTGATCAGGCGGC
AGCAAACCCTAGACCCTCGAATAGTCCAATGATTCGCAAGCCATGCAACAGCGAGAATAT
TTCTTGCTTGCTAACCATGGGAAATTCTACTCATGACACTACGAAGAAATCTGATATTGG
GAAAGCACCGCAACTCGTGCTTTTCGGCAAACCTATACTTACTGAGCAGCAGATCTCTCT
TAGCTGCTCGGGAGATACTGTTTCTACGGTGCGTACAGGGAATAGTTCTTCGGACGGAAA
TGCAGATAAAATAGGTAATGTTTCTGATGGCTCAGGTTCTGCACTTAATCAACGTGGTCT
AACGGAAAGCTCACCACGTGACACATTTCAGTCCGAACCAAATACTGAAATTGGACACTG
CAAGGTTTTTATGGATTCAGAAGATGTAGGTCGCACTCTGGATCTTTCATCGATAGGATC
TTATGAGGAGTTATGCAGGAAATTGACAAATATGTTTGGCATTGAAAACTCAGAGATTCT
TAGTCATGTGCTTTACCGAGATATCACTGGCACGGTCAAGCAACTTGGTGACGAACCATA

TAGCGACTTTATCAGAACAGCACGAAGGTTAACAATTCTAACTGATTCAAGCAGCGATAA
TGTTGGACTCAGGGAGTAGAGATAATAAGGAAATCTCCACTTTCCTTTCGAGCTGTGCAG
CTGGATGATCGAGTCCTCTGTTCCTAACATTTTAACTATTTCGGACACGCTAAAGAAGAT
ATTTAACAAGTTCAAGAGTGGAAGTTTGCGGTTCTGCAAACTTTTAAGTTCGAACGCTGT
TTTTCTCAAGGACAAGATGTTTACTTTCTAGGTTTCTTGGAATTGTTTGACTTTTGTTCA
GACAATACCTACAAAGAACAAGACGGACTTGCTTTTTGTAGGCTTTGTCTGGTTATTTTA
GAATTTGTCAATGGAAAGCCTTCTAAGAAGGCTAAGTAATGCTACTTCTAGTTTTTTGTT
TACTTTGTACAAGGAAAAATATGTTCTTATTTTCTATCTATTGCATATAGTAAAATACTT
GAGAGACTATAAACCTAGTTTGTTTAGGCGGCCTATATCATACCCTTTGGGGTGCGGCCC
TTACTCGGATCCTGCGTGAATGCGGGATGCATTCATTTTTCTTTTTATGGGTGATCCTAA
TTCTGGCAGCGAGCTTTTGTGGGTGATTCTAATTCTGGCACCGAGCACAGGCACATCCAC
TGTCTTGTACTTGAACTCTATGTAGTTGAAAATTTTGAAAAAAATTTATATATTTATACT
AAATCAAT

>comp72325_c0_seq2 310_1305
TCTCTCTCTAGGCTTATCGATATGATAATAAGGGCCGTAGTAGAGTAGAGTACAGAGAGA
TGATCAGAGAGAAACAGAAGAGGGTACAGAGCAGTTGCACAGATTTTTCCAGCGACATCA
TCCATCCTTATGATTTCCCCCTAATCTTTCAGCAAATCCCATGAACTGCCAGCCCTCTAT
ATTATTACCCCACTACTCAGTTTCCATTTTCACAACGTCAACTTCTTCTAATAAATCTCA
AGCATCATGCTCTAGCTGTTTTCCTAGTTTCTTGTTGCATTTTTCTTCTGTTAGCTAGCC
TCTTTTTCAATGGAGAACTACCAGCATTTCGACTGCAGTGATGGGAATTTGCCTCCTGGT
TTTAGGTTCCATCCAACGGATGAAGAACTCATCACTTACTACCTACTGAAGAAGGTTCTT
GACGGCAACTTTACTGCTCGAGCCATTGCTGAAGTTGACCTCAACAAATGCGAGCCCTGG
GAGCTACCTGGGAAAGCCAAGATGGGAGAAAAAGAGTGGTACTTTTTCAGCCTACGTGAT
CGGAAGTACCCAACGGGGCTGAGAACTAACAGAGCTACTGAGGCTGGTTACTGGAAAGCT
ACAGGGAAAGACAGGGAGATTTACAGCTCAAAGACTTGTGCACTTGTGGGCATGAAGAAA
ACCCTAGTTTTCTACCGAGGTAGGGCTCCTAAAGGAGAGAAAAGCAACTGGGTTATGCAT
GAATATCGCCTTGATGGAAAATTTGCCTATCACTACATCTCAAGAAGTTCTAAGGACGAG
TGGGTAATTTCAAGGGTCTTTCAGAAGAATGGTTCCACTGTCGGTGGCAACAAGAAAAGG
TTAAGTTCAAGTATAAACATGTATCAGGAAGTAAGTTCACCGTCCTCTGTTTCTCAGCTT
CCACCGCTCCTTGATTCCTCTCCTTACAGCACCACTGCCTCTTCTGCTGTCATTAATGCT
GATCGTGAGAGCTTCAAAAAGGAGCACGTGCCCTGTTTCTCCACAACTGCTACACACAGT
TTCGACCCAACAAGTTCTGTCTTTGACATTTCATCGAACAGCCTGCATGCACTGCCACCT
CCTAGTTTCAGTGCCATTCTTGACTCTTCCACTAATTTTACCCACTACACAAGGAACTCA
ACTTTTCCCAGCTTAAGGTCCCTTCATGAGAACCTGCAGCTTCCGTTGTTTTCCAGTGGA
GCTCCGGCCATGCATGGTGGTTTTTCTGCTTCGATGGCCAACTGGCCGGTGCCGGAGGCT
CAGAAAGTTGAACAGTCTGAACTTGATTGTATGTGGGGTTACTGAATGTATATGCATTTA
AGTGTTTAACTTTATTTAACTGTGATGTTCTATTTTATCAGTACTTGATTAGTAAGTGAA
TTACTGTTAATGTTCTGAAATGTTTCCTTTTGGTTGGGTAAGGAATGTACTGAGTGGCAG
CTTTATTTAGTAGTGTTGTCACGTTGTTGTAAACGTTCGGCGTAATTTAATGCATGTATT
ATTTGCTGTCTTTTCGTACGTTCTACATTTGAGGTCCAAATTGCTCAGAACTTCA

>comp80060_c0_seq5 1298_3817
TTTTTTATTAAAAAAAGAAGTTGTAGTATTATTATACTCTATCAGATTACTGAATAGTAG
TATTTTATTCGTACATTATAATTTCAATTAAATAATTGAAGAGAAAGGACATAAAAGAAA
GCACAAAAACACAAAGGCAAGGGCAACAGCAGCAGCATAAAAACACTGGCATTTTCGATT
TGCGAGCTCAGAAAGCTTTAACTCAAGCAAATTCCCACATCCTTCTCTCTCTCTTTCTCC
ATTTTCTTTTTCCCTTTTCTCACCCACCACTCTCTCACACCTCACCTTACACACTAAAAA
AAACATCACCTCTCTCTCTAAAAAATTCAATCTTTTTGCTGTTCCGACATGTCTTTTAGA
GTTTGTTTCAGTTTCAGATCTTAAGGGTGGGAGTGTTATGCTTCTTCTAATATATTGAAG
CTCAAGAAAGCTAAGAAACAGAGCAAAAATTTTGGTTTTTTTTCTCTTACTTTTTGTGGG
GGTAATTCTTGGTATTTGTAATCTTAAAGCTAGCTATTTTATGTATATACTGAAGGGGTT
GTGGTGATTTGTTTGTCTACTTTAAGAAGGTGCCATCTTTTTCAGTAATATTTGGGTAAA
AGTTCTCCTTTTTTTGGCCTTAAACGCGAAGATTCAGGCCTCTCTCAACGTGTCATTTGT
TCTCTGTAGTAAACACAGCTGGAGAAGTAATTACATAGAGGTAAAAAAGGGGGTTAAAAG
ATTACAAAGAATTGAGTGTTTGAAAAAAAAAAACAGAGGGCTGAGGTAAAAAGTTGATGG
TTTAAAAAAAAAAACTTAAATGATGATAAAGTTTAGAGCTTTATGTGAATGGAAATGGTG
TTGTGTTTGTATCAAACACGAGTAGTTTACAGCTTATGTGAATTTGAAAGAGAGAGAGAA

CTTTTGTCTGTATTTATATCCTTTTCAGCCATATCTTTCGTTAGAGCAGTTTTGGCTGTA
CCTTAATTCGTAAGGTTTTAAGCGTGAAGTGTGTGTTTGAGCCTTGTGTTATAAGGGGCA
TAAAGTATAGAAACTACAAAAGGGGGCACCTAGGAGTCTTCTGGCTCAATCAAGATCGTT
CATTTAATCTTGTCTGAGATCACTAGAAAAAGAAAAAGGAAGATAAAGATAAAGTCTTTG
TTTCTGAGAATCTTAGTTCTCTCTGTTGATATATATAATAAAAGCTGTTTACAGGGAATA
TATCTACTTGGGGTGTTCTATTTTTTAAAAGGCTGTTTGAAAATTTGGAAATCTTGAATT
TTTTTTTTTGGTTTGGGATTTTGGGGTTTGAGGGAAAATGGCTATGGTTGCACAGCAACA
TAGGGAGAGTAGCAGTGGTAGTATTACAAAACATCTTGACAGTAGTGGAAAGTATGTCCG
GTATACAGCTGAGCAAGTTGAGGCATTGGAGAGGGTTTATGCAGAGTGCCCTAAGCCTAG
CTCATTGCGACGCCAGCAATTGATCCGTGAATGCCCTATTCTGTCGAATATCGAGCCTAA
GCAGATCAAAGTTTGGTTTCAAAACAGAAGGTGTCGAGAGAAGCAAAGGAAAGAGTCTTC
TAGGCTACAGACTGTAAATAGAAAGCTTTCTGCAATGAATAAACTATTGATGGAGGAGAA
TGATCGCTTGCAAAAACAGGTTTCGCAGCTTGTGTGTGAAAATGGCTTTATGCGGCAACA
ATTGCATACTGCATCAGCGGCCACTACTGATGTAAGTTGTGAGTCAGTGGTTACCACCCC
TCAGCATTCCCTCAGAGATGCTAACAACCCTGCTGGACTGCTGTCGATTGCAGAAGAAAC
CTTAGCAGAGTTCCTTTCCAAGGCTACAGGAACTGCTGTTGATTGGGTCCCGATGCCTGG
GATGAAGCCTGGTCCGGATTCAGTTGGGATTTTTGCCATCTCACGCAGTTGTAGTGGAGT
GGCAGCCCGAGCATGTGGTCTTGTTAGTTTAGAGCCGACAAAGATTGCTGAGATCCTCAA
AGATCGACCTTCTTGGTTCCGAGACTGCCGGAACGTTGAAGTTTTCACGATGTTTTCTGC
AGGAAATGGAACAATTGAGCTTTTGTACACGCAGATATATGCTCCTACCACCTTGTCTCC
TGCACGTGATTTTTGGACTCTGAGATACACAACCACCCTGGAGAATGGTAGTTTTGTGGT
TTGTGAAAGATCCCTCTCTGGTACTGGAGCTGGGCCGAATGCTGCTTCTGCTTCCCAGTT
TGTAAGAGCTCAAATGCTTCCGTCCGGATATCTAATCCGACCGTGTGACGGTGGAGGATC
CATTATACATATTGTTGACCATCTGAATCTTGAGGCATGGAGTGCCCCCGAGATCCTGCG
TCCACTTTATGAATCGTCAAAAGTTGTGGCACAGAAAATGACTATTGCGGCACTTCGATA
TGCAAGGCAAATAGCTCAGGAGACTAGTGGGGAGGTTGTATATGGTCTGGGAAGGCAACC
TGCAGTTCTTCGAACATTTAGCCAGAGATTAAGCAGAGGCTTCAATGACGCCATCAATGG
ATTCAGTGATGATGGCTGGTCATTGTTAAGTTCTGATGGTGGTGAAGATGTTATAGTTGC
TGTCAATTCAAGGAAGAACATTGCCACCACTTCTGTTCCTCTTTCACCGCTGGGAGGCAT
TCTTTGCGCCAAAGCATCAATGCTACTCCAGAATGTACCTCCTGCTGTGCTGGTTCGATT
TCTCAGGGAGCACCGTTCAGAGTGGGCGGACTTTAATGTTGATGCCTATGTAGCTTCCTC
GATGAAATCTTGTTCATATGCATATCCTGGGATGAGGCCTACCAGATTTACCGGAAGTCA
GATAATAATGCCACTTGGCCATACAATTGAACATGAAGAGATGCTTGAGGTTATTAGATT
GGAAGGGCACTCTATTGGCCAGGAAGATGCTTTTATGCCAAGAGATATTCACCTTCTCCA
GATGTGTAGTGGAACTGATGAGGATGCTGTCGGAGCTTGTTCTGAACTAGTTTTTGCTGC
AATTGATGAGATGTTTCCAGATGATGCACCCCTGTTGCCCTCCGGGTTTCGTATCATTCC
TCTCGAGTCAAAATCAAGCGATCCCCAGGATACATCGAATGCTCATAGAACGCTGGATCT
AGCATCAAGTCTTGAAGTTGGCCCAGCAACAAACCCTGCTACTGGAGATGTGGTCTCTGG
CTACAGTGCACGGTCTGTATTGACAATTGCTTTTCAATTTCCATTCGAGGACAATCTTCA
AGACAATGTAGCTACCATGGCGCGGCAGTATGTTCGCAGTGTGGTTTCATCTGTCCAACG
GGTTGCCATGGCAATATCTCCCACAGGAGTGAATTCAACATTCGGGTCCAAGCTTTCTCC
AGGCTCCCCTGAAGCAGTAACTTTGTCGCACTGGATCTGCCAGAGCTACAGTTATCACAT
GGGGACAGAGTTGCTTCAAACTGATTCGAGGGGCGATGAATCAGTGCTAAAAAATCTTTG
GCAACATCAGGATGCTGTTTTGTGCTGCTCATTGAAGTCTCTGCCGGTTTTTATTTTTGC
TAATAAGGCTGGGCTTGATATGCTGGAGACCACCTTAGTTGCTTTACAAGACATTACTCT
AGATAAGATATTTGATGAATCTGGCCGGAAAGTGTTGTTCGCTGAATTTCCCAAGATCAT
GGAACAGGGTTTCGTGTACTTGCCGGGTGGTATGTGCATTTCAGCAATGGGACGACATAT
TTCATATGAACAAGCTATTGCATGGAAAGTCTTTGCTTCTGAAGAAACTAGTGTCCACTG
CTTAGCCTTCTCATTTATAAACTGGTCATTTGTTTAATGTTGCTGTCAAATCTCTTTTCT
TTTTTGACATCTTCCTTACAGAGGACACTGACAGCCAGGAACACAGTTGAACGGAATGAT
CTGGGGACTGATGAAAATTTTGTAACATGGGGGGGCGCTCCCATTTGTTTTGCCTTTAAT
TTAATTAGACTAAATTTGTATTTTGCTTCCTGAATTCTCCATACTTTTATGTAAATTTTC
TGGTGCAGCTTTTTTGAGTGCAAATATTTGTTTCCGCATATTCCGGCACTGATTTATTTT
AAATTTAGCTTAAGTATTTGCAGTGATATTCAAAATGCTGCAATGTTGCCAAACAAAA

>comp79517_c0_seq6 1043_3556
GGCATATTGGTGGTAGCGACGTCTCTATCACAGATATTCCATATCTGGTGCAGCAAAAGA
GTCACACTGTACTGTACGTCTGTATGAAGATGTAGTAGAGTAGTAGGGGTGATTCAATTG

GCCTCAAAAGAGGAAAAGAAAAGGTTACGAACCACTGATCTGTTACAGCTCAAAACCACT
TTCCCAATCTAAACAAAACCATACTCCCCCAGCCCCACCCCCAACATCACATTACCTCCC
AAATACAGCACTTATCCCAAGTTTCAGTTACTTTTTCCTAACATATACCCCAAATCAGTT
CATTTTCTTGATCCAAGATCTATCCTTTTATATAAATATCATACCTAAAAATCTTCCCTT
AAAGATGCTGAATATTGGAAAGAAGTGAAAGAGGGAAGTTCATAGCTATGAAGATTTGGT
TTCTGGGTTTTGCTCATTATGCTTTCAAAAGCTTAAAGTTTGCATCTTTTTGGTAAATAC
CAAAGTTTAAAGTTTGCTACTTTACTGTTTAGTAAGTGGATGGAAGAGGAAATGAAGCTT
GCAAATCTTTTATCTTTATAGTGAGGAAAAAAAGAACATTTTTTTATGAAATAATGGTGT
ATTTAATTGATATTGGTATTAGTGAAAATGTATGTTTAGTTCTTGGGAGCTCTGAATTCG
ATAGATATATGTTTTCTTGTGCTTGACGCTGAGGATAGTGCCGTTTCGTTTTGGTAGTTG
GTTGTCTGCTTATTTGTTTCCACATCAAGAATCTTGATTTTTGGGACCATAGCTTTTAGT
ATTACCAAGAAAAAGAGAAAACAAGCTGTGGTTTTGATGCAAGTGTTTGCATTTTTGTGA
AGAAGGGGAGAAGCTGTGACTCTTGTTTTATTGGTTACTCAATATTTTTGGGACCATTCC
TTTTACTATTACCAAGAAAAAGTTGTGTTTTGATGCAAGTGTTTGTACTGTTGTGAAGAA
GGGGGAAAGCTGTGAATCTTGATTTGTTGATTACTCATATTTTGGAACTGTTGTGTAAAA
GGGTGGATTATAAGAAAAAATAATGTCAATGTCCTGCAAGGATGGTAAGTCAGTTGATGA
TAATGGGAAGTATGTTCGGTATACACCTGAGCAGGTTGAAGCTCTTGAAAGGCTATATCA
CGAGTGTCCAAAACCCAGTTCAATGCGTCGCCAGCAGCTTATTCGTGAATGCCCTATTCT
CTCTCACATTGAGCCTAGGCAAATCAAAGTTTGGTTTCAGAACAGAAGATGTAGGGAAAA
ACAGAGGAAAGAGTCATCTAGACTTCAGGGTGTAAATAGGAAGCTGTCAGCTATGAATAA
GCTATTAATGGAAGAAAACGATAGATTGCAGAAGCAAGTTTCTCAACTGGTATATGAAAA
TGGCTACTTCCGCAAGCAAACTCAAACTACAAAGATTGCTTCAAAAGACACCAGTTGTGA
ATCAGTGGTAACGAGTGGTCACCCCCACTTGTCACCTCAGCATCCTCCAAGAGATGCTAG
TCCTGCAGGGCTCTTGTCCATTGCAGAAGAGACTTTAACAGAGTTTCTATCAAAGGCTAC
TGGAACTGCTGTTGAGTGGGTCCAAATGCCTGGAATGAAGCCTGGTCCGGATTCCATTGG
AATCACTGCTATTTCTCATAGTTGCTCAGGAGTAGCCTCTCGAGCTTGTGGCCTTGTGGG
TCTAGAGCCTACAAGAGTTGCTGAGATCCTCAAAGATCGGCCATCATGGTTCCGTGATTG
TCGAGCAGTGGATGTTCTCAATGTGATGTCTACTGGGAGCGGTGGAACCATTGAGTTGAT
ATACATGCAGCTCTACGCACCTACTACTCTGGCACCAGCTCGTGACTTCTGGTTAATGCG
GTATACATCTGTTATGGAAGATGGTAGCCTTGTGGTATGTGAGAGATCACTGAACAACAC
TCAGAATGGTCCTAGCATGCCACCGGTGCAGAATTTTGTGAGAGCAGAACTGCTTCCAAG
TGGGTATCTGATACGACCCTGTGAGGGGGGTGGTTCAATCATTCACATAGTTGACCATAT
GGATCTAGAGCCCTGGAGTGTTCCCGAAGTATTGCGTCCACTGTATGAGTCATCAACAGT
GCTGTCGCAGAGGACAACAATGGCGGCATTACGTCACCTGAGACAAATTTCTCAAGAAAT
TTCTCATCCTACTGTCACTGGTTGGGGAAGAAGACCTGCAGCCCTGCGTGCACTTGGTCA
AAGATTGAGCAAGGGTTTTAACGAGGCTGTTAATGGTTTTACTGATGAGGGATGGTCTAT
GCTTGAAAGTGATGGTATAGATGATGTTACACTTGTTGTCAACTCATCACCCAGCAAATT
GATGGGTGCAAATATCTCTTATGCCAACGGATTTCCATCAATGAGTAGCGCTGTATTATG
TGCCAAGGCATCTATGCTTTTACAGAACGTACCTCCTGCGATTCTGCTAAGGTTCTTGCG
GGAACACCGATCTGAATGGGCAGACTCTGGGATTGATGCCTATTCAGCTGCTGCAGTTAA
AGCTGGTCCATGCAGCATACCAGTCACTCGAACTGGAAGTTTTGGTGGACAAATCATTCT
TCCACTGGCTCACACTATTGAACATGAAGAGTTTATGGAGGTGATAAGACTTGAAAGCAT
CGGCCACTATCAAGATGATATGATTATGCCTGGTGACATCTTCCTTTTGCAACTTTGTAG
TGGAGTGGATGAGAACGCTGCCGGAACATGTGCGGAGCTCGTCTTTGCTCCTATTGATGC
CTCTTTTGCTGATGATGCTCCTCTTCTTCCCTCTGGTTTTCGTATCATTCCTCTGGACTC
CAAGACGGATGCCTCTAGTCCAAACCGCACACTAGATCTTGCATCGACTCTTGAGGTTGG
ACCGGCTGGAAGTCGACCAAATGGCGATTATTCCAAAAACTCTGGTAGCACGAAATCGGT
TATGACAATAGCATTTCAATTTGCATTTGAGATCCATCTTCAAGAAAACATAGCTGCTAT
GGCACGGCAATATGTCCGCAGTATCATATCATCTGTTCAGAGGGTTGCTTTGGCACTCTC
TCCATCTCGTCTTGGTTCTCTCCCTGGTCTAAGGTCTCCACCTGGCACACCTGAAGCACA
AACGCTTGCCCGTTGGATATGTCAAAGCTATAGGTTCTTTTTAGGTGTAGAGCTGCTCAA
ATCTGTTAGCGGAGGAAGTGAATCCATCCTGAAAGAAATTTGGCATCACTCAGATGCTCT
GATGTGTTGTTCTTTGAAGGCATTGCCTGTTTTCACATTTGCAAATGAGGCAGGACTTGA
CATGCTAGAAACCACCTTGGTCTCCCTCCAAGATATTACTCTCGAAAAGATTTTTGATGA
CAACGGAAAAAAGGCACTCTTCTCTGAGCTTCCTCAAATAATGCAGCAGGGTTTTGCTTG
TCTTCAAGGCGGTATCTGTTTGTCGAGTATGGGAAGGCCAATATCGTATGAGAGAGCAGT
AGCTTGGAAAGTATTGAATGAAGATGAAGATCCCCATTGTATATGTTTCATGTTCGTCAA
TTGGTCTTTCGTCTGATATATGTACGTAGGATATGCATACAAACTTAGATCAGCAACAA

ACACCCCCTTTTGCTTTATGGATCAACCCTCTTGCCTTTAGGCAAAAAAAAAATCTGCAA
GTGGGCTCGTATTTCTTTTCCCGTTCTCTCGTCATATATACACTTGTGTATTTTGGTTGG
TTTGTAAACTTTGTTACTTTCTTTTATCTTATGTTTTTCACTTCCCCTTGGCACTTGCTA
TATTACATTTAGCTCTTGCTCGAGTTTTCCTGGAAAATTGATTGAAGGTTAAGAGAGA

>comp79347_c3_seq8 336_611
CGGATGTGCAAGCAAGGCCAAGACCTTGGCATGGGCAAGGTTTGCTTGGAAGGGCTTGAC
AAGGCAACGGACGGGCGGCAGCATGGCATGGCATGCGCGCCAATATCAGTGGGTCGACGG
GCTGGAACGGGCAAGCAAGGCCAAGACCTTGTGGCGGACAAGGTTTGCTTGGAAAGGGCT
TGACAAGGCAGCATCGGCAAAATGGGGCGGCATGGCACGGCATGCGCGCCAAAGACAGTC
GGCATGACATTGGTCGGGACAGCTAAATGCGGGCTAAGCTAAGGCAAAGCGGGAATGCGG
GTTGACGGCAAAGGCTTTCAATAGCCAAGGCAAGGCTATAGGAAAGAAAATACAAGTGAT
GGCACGAGGGAAATGAGGCAGAAAGGAACATGGCAACGGACAAGGGCCAAAGGCAGTGTG
CGCACGGTGTATGGGCGCCGGGCAGTGTGCGGGAAATATGATGGCAGCGGATTGCGCCGG
CATTGGTATGGGACAATGTTAAATTGACTAAGGCATAGCTCGAGCATCCTTAGGGCGGAA
TGCAGCTGGTCCGAATTGCACATGAGAAGCACATGGAAGCGGTTGGCAGGAGTTCCTTGT
TTGTAGGCCATGATCTCCCATGTTCCCCCATGATCCCCATTATCTTGTAATGCTTGTATA
ATGCCCCATTTCGTGAGTAGTGGATCCTAAGTGGCTGGGGTTGTCAGCCACACGTTAGGA
ACGTGGGCTAGCTTAGTGGGACAAGGCAGTTGGCCGAAAATGTGCATGTGCTGCCAAGTG
AAGGGTTCCTATGTGCTATAAATAGGACCCTTAGGCTTAGGCAGAAAAGGGGTCAAGTAA
TTCGTGTGTAGTTGCTTGCTGGGAAACACGAGGGAAACGTGAGGAGTTCTAAGTGTTTCA
AGGAAAAAGGGGACTTGGGTTGTGAGACAATCCGAGTGTTCGGCCAAGATTGACAAGGTC
ATCTTAGTCGGAGAGTGGGCTGTGAGCGGCATTGTTCATAGCGAGAGTGAGGGTCCTTTG
TATTCTTTTCCATATTAATATAAAGGTTGGGAAATTGTTCCCGTATATGCTTGTCTCATT
TAAATTCTGTTTTGCTGTCCAAATTCGTCTAAGGCTAAACTTGTGTGCGATTGGCAAGTT
TTGGGGGAAGGCTGAGTCAGGCAAGGCTTGACTGCCGCACGGAGGTGAAACCTCGGCCAG
GATTTAAGTGGCCAAAATCGCCCCCGTAACACTTAGCAACAGATGTAGGAATCATTCTGA
AGTTTTGGAGCTTTTTCCAAATTATGTTAAATTAATAGTAAATAATAAAAGATTCACGTC
TCAGCTATTCACGGAACTATACCTCCTATAAAATTAAACTACTAAAGATGACTAAATTAT
TTTAAGCAAATTAACGAAGAGAAAAAACATGTTATGGTAACAGACATGACAAAATTTTAT
TTTTACAAATAAAACTATATAGTGATCCATCAAACAAACACTGAACCAACTTTCCAGAAT
TTTAAATTGGATTCAATCACAATGGAACATTGTTCAACTAATGTAAACCAATTCAACTCC
ACACGGTCTCACCAAGCTAACAGACTTCAAAACTAACAGACAAGAATAGGAGATGTATTT
AACAGATGAACAATAGCATATCTAGAGTTCATAACCACTACATGCACATAAATAAAGCAA
ACATTTTGATAAGAGTCATCGTATTAATGTGCAGCAAAGTGCTGCAAGTATTTACAGGAG
ATAGCCACATTACAGAATATATAACAAGCTCAAAAGATTGATCATGGCTTCTATCATAAA
CAAACACGCAATTGCTTGACAACCATCATAAGTAAAGCTTTACACATGACCCTCTGGTTT
AAACTACGTCACAAGAGTCACAACACACAATTTTCACCCTTCAATTTGAACTAATTAAGC
TTGAAATAAAGTGAAATCTTACAGAAGAAAGCATATCCAAATTTAGAGCAGATTCGTTGC
TATTATATCTAGAGATACTGCGATTTTAACAAAAAAACATCAATGCACAATAGATAGAGT
TATCAACACTATCACATTTAATTTAATTTCAATCATGCTCGAGCACAAAATCATATATAA
TATACATATTTAGAGATAAAGAGGATCTAGACAGAATTCTTGCAAAGCTAAATGAATCAG
TCAATCAAAGAATTTAGACAAAATAGCCATCATTTCTTGCAAATCAAAGGACAATATAGA
TTTATGATATGCACCTTGAGATCAAATAGCACTTGGATCCGGAATCGTTTGGAACAGAGA
CCTCGATGCAACAATCTCGAACGGAAACCTCAGCGAGACTGGAAACGACATCCTCGCTCA
CCTCAGATAGGGACCCGACTGAAAATCAGAAGCAAACAAGAAATCGACATGAAAATCGAA
GAGATTCGAACTAGAAATGAGAATGAAACAAGTTCGTTCGATCCCTTTGTTCTCACTATT
TTCCAACTCTCACGTTTTCCCGTTCCTGTTGCCCCTTTTCTGTTTCGATTTCAATTCCCT
TCTCCTGTTATTTTTGTTTCGTTTTAGTTCAGAAGAGAGGTGAAAGCACGTTTAGAGTGT
GTGAAGCACGTTTAGAGTGTGTGCTTGTTTCGCGTGATTGATTTTCAAGCGCGGGATCGT
GTGCTTGAAAAGGCAGAACGCTGGGAGGGGTATTTGGGTGTCCTCCGGTCTCAGCTTTTA
CAGGCGTCCCTTCCCTATCTATTCTATAGTGTATCCGTATATATGTGTTTGTATCTGCGT
GGATTTGTTAAGAAGAAATCACAACTCTGCAGCCCTCTCTTACATGCGTCTCCTTCTGTG
TATAGGCGTATATGTGTGTTTTTGTGAAGAAACAGCCCCAGCGTCCTTCAATCTCCAGTC
CTCTCTACTGTCTTCTGTATATGCG

>comp79742_c0_seq4 124_2421
AACCCTTTCTATATAAACCAAATCACCTTGCGCTTCAAACTCGCGTCCTCCCCTTTTCTT

CTTCTTCTTCATTCTACAACCAAGAACCACTCTTCACCGGTTCCTATATTTTCCTAAAGA
AAAATGGCATCTCACATTGTTGGATACCCCCGTATGGGCCCAAAGAGAGAGCTCAAATTT
GCTCTCGAGTCTTTCTGGGATGGAAAGAGCACCGCTGAGGACTTGAAGAAGGTGTCTGCT
GACCTCAGGTCATCTATCTGGAAACAGATGGCTGATGCTGGTATTAAGTACATTCCCAGC
AACACCTTCTCTTACTATGATCAAGTGCTCGACACAACCGCGATGCTTGGTGCCGTCCCT
TCTAGGTACAACTGGACCGGTGGTGAGATCGGGTTCGACACTTACTTTTCCATGGCCAGA
GGAAATGCCTCTGTCCCTGCTATGGAGATGACAAAGTGGTTTGACACCAACTACCACTTC
ATTGTCCCTGAGTTGGGACCTGATGTTAACTTTTCTTATGCTTCTCACAAGGCAGTAGAT
GAGTACAAAGAGGCCAAGGGGCTTGGAGTAGATACTGTTCCAGTGCTTGTTGGTCCAGTT
TCATACTTGTTGCTTTCCAAACCTGCTAAGGGCGTTGAGAAATCTTTCCCCTTGTTGTCA
CTTCTGGACAAAATCCTTCCAATTTACAAGGAAGTTATCACGGAGTTGAAGGCAGCTGGT
GCTTCTTGGATTCAGCTTGACGAACCTACGCTTGTGTTGGATCTAGAGGCTCACAAATTG
GAAGCATTCACTAAGGCCTATGCTGAGTTGGAGTCATCTCTATCTGGCCTTAATGTTATC
GTTGAGACCTACTTTGCTGATGTTCCTGCTGAGGCATTCAAAACCCTCACTGGTTTGAAA
GGAGTTACTGGATTTGGCTTTGACTTGGTCCGTGGAACTCAGACTCTCGATTTAATCAAG
GGTGGTTTCCCTTCTGGCAAGTACTTGTTTGCTGGAGTTGTTGATGGGAGGAACATTTGG
GCTAATGATCTTGCTGCATCTCTTAATCTCCTGCAATCTCTTGAGGGCATTGTAGGAAAA
GACAAGCTTGTTGTCTCTACATCTTGCTCCTTACTCCACACTGCTGTTGATCTGGTCAAC
GAGCCAAAGCTAGACAGTGAAATCAAATCATGGCTCGCATTTGCTGCCCAAAAGGTTGTT
GAAGTAAATGCTTTAGCCAAGGCATTGACTGGTGCCAAGGATGAGGCTTTCTTCTCTGCC
AATGCTGCTGCTCAAGCTTCCAGAAAGTCCTCCCCAAGAGTGACAAATGAAGCTGTTCAA
AAGGCTTCCGCTGCTCTTCAAGGATCTGACCACCGCCGTGCTACAAATGTTAGTGCTAGA
CTTGATGCCCAACAAAAGAAACTTAACCTACCAATTCTGCCTACAACCACCATTGGATCC
TTCCCTCAGACTGTGGAGCTTAGAAGAGTTCGTCGTGAATACAAGGCCAAGAAGATCTCT
GAGGAGGAATATGTTAAAGCCATCAAGGAGGAAATCAAGAAGGTTGTTGACCTCCAGGAA
GAGCTTGACATTGATGTCTTGGTTCATGGAGAGCCCGAGAGGAACGATATGGTTGAATAC
TTTGGGGAACAACTTTCTGGTTTTGCATTCACTGCTAATGGATGGGTTCAATCTTATGGA
TCTCGATGTGTGAAGCCACCAATCATCTATGGTGATGTCAGTCGCCCAAAGCCAATGACT
GTATTCTGGTCCAGTATAGCTCAGAGCATGACCAAACGCCCAATGAAGGGAATGCTTACA
GGACCAGTTACCATTCTCAATTGGTCATTTGTCAGAAATGACCAGCCAAGATTTGAGACC
TGCTACCAGATTGCTTTGGCCATTAAGGATGAGGTGGAGGATTTGGAGAAGGCAGGCATC
AGTGTCATCCAAATCGATGAAGCTGCACTGAGAGAGGGGTTGCCTCTTAGGAAGGCTGAG
CATGCTTTCTACTTGAACTGGGCCGTTCACTCCTTCAGAATCACCAATGTCGATATCCAG
GACACTACCCAGATCCACACTCACATGTGCTACTCCAACTTCAATGACATTATCCACTCC
ATCATCGACATGGATGCTGATGTGATCACAATTGAGAACTCACGTTCCGATGAGAAGCTC
CTCTCAGTTTTCAGGGAGGGAGTGAAGTACGGAGCTGGCATTGGTCCCGGTGTCTATGAC
ATCCACTCTCCAAGAATACCATCAACCGAAGAAATTGCTGATAGAGTGAACAAGATGCTT
GCTGTTCTTGACACCAACATCTTGTGGGTCAACCCTGACTGTGGTCTTAAGACCCGTAAG
TACACCGAGGTGAAGCCAGCACTCCAAGCCATGGTTTCTGCAGCCAAGGCCATCCGCACC
CAGCTTGCCAGCGCCAAGTGAGCTAGTTGGAAGAAACAAACCCCTCGGGGCATTACTCAT
GGTGTTCTTTAACTTGAAAAGGAAATTTTCTATTGATTTTTAATAATTGTGCCTGCTATG
ATATTATTCTGTAGGTTAGTAGTACTGTCCCCTGGCCTCCCATTCTTCTTTTTTATGTTT
TTCTTTAGAGCATTCTCTCTCTGGAGGAAATCATATCGGCTCTATTTTGATGATGTTATC
CTCAAATTTATTTGAAAGGCGTGAAGAAGATTTATGTATTCTAAATGAATGAAAACCAAA
GAATTGCCATCCTCTGTTTTCATTCATGTCTGTTGGTGATTGTTTGCAAGTCAGCATCTT
TTTCTTCTTCGAGTTCTGCTTGGGCATAATTGGTCTTCATATCTAAA

>comp74516_c0_seq2 292_2619
AATAAAAGGCGTTATTTTAGGAGGCAACATCTGGAAAGATTCCGTTTGGCACATATTGTG
TAATAAAAACATAGAATATGATAGTAATAAACAAATAATGCATAATAATAGTAGTAAAGT
CTATATGAAATGTATAGATAATCCCTAAAAATATCTGGCTGGGTATCCAAATTGAATGGA
AAGTGAATCACTCAGTATTTTCAATGTGGACCCAATAAGGTCCACCCAATTGGTTTCTTC
TTCTTTCTTATATAAACCAGAAAATTGGTTTCCTCCGGCAGACTGTCTATAATGGCGTCA
ATTTCCGTTTTTCCATTCTTTTTCATTATTATCTCTTTCTGCCTCACTCCGGTGACCATT
TCCGTCCAATCCGATGGTCATGAAACTTTCATCATTCACGTTTCCAAATCCGATAAGCCC
CGTGTTTTCACCACCCACCACCATTGGTACTCCTCCATCATCCGATCCGTTTCCCAACAC
CCTTCTAAAATCCTCTACACCTATGAACGTGCCGCCGTGGGCTTCTCTGCACGCCTCACA
GCCGCTCAGGCCGATCAGCTCCGCCGTATTCCCGGTGTAATCTCCGTCCTTCCCGACGAA

GTACGCCATCTCCACACCACCCATACCCCTACCTTCTTGGGACTTGCTGACTCTTTCGGC
CTTTGGCCCAACTCCGATTACGCTGATGATGTCATCGTCGGAGTTCTGGACACGGGTATA
TGGCCGGAAAGACCGAGTTTTTCCGACGAGGGTCTCTCTACGGTTCCTTCAAGTTGGAAA
GGGAAGTGCGTTACTGGACCTGATTTTCCTGAAACCTCATGTAATAAAAAAATCATAGGC
GCTCAAATGTTTTACAAAGGGTATGAAGCTAAACATGGCCCAATGGATGAATCAAAAGAA
TCAAAATCGCCAAGAGATACTGAAGGACATGGAACACATACAGCATCAACAGCAGCTGGT
TCTTTAGTGGCAAATGCTAGCTTTTACCAATATGCCAAAGGTGAAGCTAGAGGTATGGCT
ATAAAAGCAAGAATAGCCGCTTACAAGATTTGCTGGAAAAATGGCTGTTTTAATTCTGAT
ATATTGGCTGCCATGGATCAAGCTGTTGATGATGGTGTGCATGTGATCTCACTTTCTGTT
GGGGCTAACGGTTATGCTCCACATTATCTGTATGATTCTATTGCAATTGGAGCTTTTGGT
GCATCTGAACATGGCGTCCTCGTCTCATGTTCAGCTGGAAATTCTGGTCCCGGAGCTTAT
ACGGCAGTGAACATTGCCCCGTGGATGCTCACTGTTGGTGCATCAACTATAGATCGTGAG
TTCCCGGCAGATGTTATTTTAGGAGATAATAGAATATTTGGTGGTGTTTCATTGTACTCC
GGCAATCCTTTGACCGATGCCAAATTGCCGGTGGTTTATTCCGGCGACTGTGGTAGCAAA
TACTGTTATCCAGGAAAGCTAGATCCTAAAAAAGTCGCAGGAAAAATTGTTTTATGCGAT
AGGGGAGGCAACGCTAGGGTTGAAAAAGGGAGTGCGGTGAAGCAGGCAGGCGGAGTAGGG
ATGATACTTGCTAATTTGGCTGACTCCGGCGAAGAACTCGTCGCCGATTCACATCTTCTC
CCGGCGACGATGGTCGGTCAAAAAGCTGGAGACAAAATAAGACACTACGTAACGTCTGAT
CCTTCACCCACGGCGACGATCGTGTTCAGAGGAACAGTGATCGGAAAATCACCGGCAGCA
CCACGTGTAGCGGCGTTCTCGAGCCGAGGACCTAATCATTTGACGCCGGAGATTCTTAAA
CCGGATGTTATTGCACCTGGAGTTAACATTTTGGCCGGTTGGACCGGATCTGTTGGACCG
ACCGATTTGGATATTGACACGAGAAGAGTAGAATTCAATATTATTTCTGGAACTTCCATG
TCGTGCCCTCACGTTGGGGGATTGGCTGCTTTACTTAGAAGGGCCCACCCAAAGTGGACC
CCAGCAGCGGTAAAGTCAGCACTTATGACAACAGCTTACAACTTGGACAATTCTGGTAAA
GTATTTACAGATCTTGCCACTGGCCAAGAATCTACTCCCTTCGTTCATGGATCAGGTCAT
GTAGACCCGAACCGAGCATTGGATCCGGGTTTGATTTACGACATCGAAACTAGCGATTAC
GTAAATTTCCTATGCTCCATTGGCTATGACGGCGACGATGTCGCCGTGTTCGCGAGAGAT
TCTTCTCGAGTGAATTGCAGTGAACGAAGTTTGGCTACTCCGGGAGACCTGAATTACCCG
TCGTTCTCCGTTGTTTTTACCGGTGAGAGCAACGGTGTGGTTAAATACAAGCGGGTGGTG
AAGAATGTAGGAAAAAATACAGATGCTGTGTATGAAGTGAAGGTGAATGCGCCGTCGTCG
GTGGAGGTGAATGTATCACCGGCGAAGCTTGTATTCAGTGAGGAAAAGCAAAGTTTGTCG
TATGAGATTAGCTTAAAGAGTAAAAAGAGTGGTGATTTGCAGATGGTGAAGGGGATTGAA
TCTGCATTTGGGTCGATTGAATGGAGTGATGGAATTCACAATGTGAGAAGCCCAATTGCG
GTGCGTTGGCGTCACTATTCTGATGCAGCATCCATGTGAGTAATGGATGATTGTTCTTTA
TATTGCATTGCATGGACCAATAAACTGGGATGATGACAAATTGAAAGACGAAATGTTGCT
AGAGGATCATCGAATTTGTCCAACTTTAATTTCACTTTCTTTACCTTTTGTTCTCTGATG
TTGTTCAGATTGATGTATATATGAATGAAGCATACCCAGTTGTTTCACAGTACTTACGTA
ACAGTTCAAAAATCGCATCATACGTGCAATTGAATACCAGTTCCAATATAAATCAGTCTC
ATTCATAGCGTCACAAATAAAACAGCAACAACAACTCAGTGTGGTCCCACAAGTGGAGTC
TGGGGAG

>comp79927_c1_seq7 1_243
AGTAAAATCTACCTTAAACGCGTTGACAAAGTTCAATTCTTGTCCTTTTGGGAAGCTTCA
GGCTCCTCCGACTCCTTTTCACCTGATCCTGCTACTTTCTGTGATTTTCTTTCTGTTTTT
AGTAACTCAGTCATAACCCTGTATAAGGTACTTCCTTTTCTTGATCTTTTGCATTTTCCG
CTGCTGATTATGGATATTTCTCTTGATTCAAGAACCCCATTGATAAGTTCTATTTTCACC
TGATCAATTTGATATATTTCCTTTAGAATATCTATCTTTTGCAATTTAACCCCCCCTCCC
CCCCTCTCCCCCCTCTCTCTTTCTCTTGTAGTCCTTTAGTGCCATTTTTTCTCTGCTCCC
ATCTTTTGGAGAGTGCGGTAATCGGCTTTTACGGCGGCGGCGGTTTTTGAGTTGATACAA
TGGTGCGGAAGAGGAGAACTGAGTTACCTGGTTCTGGTGAGAGCTCTGGGTCTCAAGAAA
CTGGAGGACAGGGTCGTGGCCAGCATCCACAGCAGCTGCAACAACAACAAGGAGGCTACC
AAGGTGCTGAAGAAACTGGAGCACAGGGTCGCGGCCAGCGTCCACAGCAGCAGCAACAAG
GCGGTTACCAAGGTGGAGGAAGAGGCTGGAGGCCGCAGCAGGGAGGATATGGTGGTCGTG
GTGCTGGTGGACTTCCACGTGGTGGAATGACCCCCCAACAGTCATATGGTGGAATGGCCC
CTCAACAGTCCTATGGTGGACCTTCTGAATACTACCAACAGGGCAGGGGAACCCAGCAGT
ATCAACGAGGTGGAGGACAACCCCAGCGCCGTGGTGGCATGGGTGGCCATGGGGCACCTT
CTGGTGGCCCTCCTCGGCCACCAGTACCCGAGCTGCACCAAGCAACCCAGACTCAACATC
AGCCTGTACCATATGGAAGACCATCAGAAACATACTCGGAGGCTGGTTCCTCGTCTCAGC

CACCCGAACCAACGACACAGCAAGTGACTCAGCAATTCCAGCAACTTGTTGTGCAGCCAG
AAGCAGCTGCAACCCAAGCAATACAACCAGCATCGAGCAAGTCGATGAGGTTTCCACTCC
GGCCAGGAAAGGGTAGTACTGGTATTAGATGCATAGTTAAGGCCAATCACTTCTTTGCCG
AGTTACCTGACAAAGATCTGCACCAGTATGATGTTTCAATTACTCCTGAGGTCGCCTCTC
GGGGTGTCAACCGGGCTGTCATGGAGCAGCTGGTGAAGCTTTATAGAGAATCCCATCTTG
GGAAGAGGCTTCCAGCCTATGACGGAAGAAAAAGTCTATACACAGCAGGGCCCCTCCCTT
TTGTTCAAAAGGATTTTAAAATCACTCTAATTGATGATGATGATGGACCTGGTGGTGCTA
GGAGGGAAAGAGAGTTTAAAGTTGTGATCAAGCTGGCGGCTCGTGCTGATCTTCATCACT
TGGGGATGTTCTTACAAGGGAGACAGGCTGATGCACCGCAAGAAGCACTTCAGGTGCTGG
ATATTGTGCTACGTGAGTTGCCAACATCTAGGTATTGTCCTGTGGGCCGCTCTTTCTATT
CCCCTCATTTAGGACGAAGACAACCACTGGGTGAAGGTTTAGAGAGCTGGCGTGGCTTCT
ATCAAAGTATTCGTCCTACACAGATGGGATTATCCCTGAATATTGATATGTCTTCCACGG
CTTTCATTGAGCCACTGCCGATTATTGACTTCGTGAGCCAGCTTCTGAATCGGGATATCT
CTTCTAGACCACTGTCTGATGCTGACCGCGTTAAGATAAAGAAGGCACTGAGAGGTGTAA
AGGTGGAGGTCACTCATCGTGGAAATATGCGGAGGAAGTACCGCATTTCGGGTTTAACAT
CTCAAGCAACAAGAGAGTTGACCTTCCCTGTTGATGAAAATGGTACAGTGAAATCTGTAA
TTGAGTATTTTCGAGAAACATATGGGTTTGTAATTCAGCATACTCAGTGGCCTTGTCTAC
AAGTTGGAAATCAGCAGAGACCTAATTACTTGCCAATGGAAGTCTGCAAGATTGTGGAGG
GACAAAGGTACTCAAAGCGCTTGAATGAGAGACAGATTACTGCACTTCTGAAAGTGACCT
GCCAGCGTCCCCAAGAGAGGGAGCGTGATATTCTTGAGACCGTACATCATAATGCCTATG
CTAATGACCCATATGCCAAGGAGTTTGGTATTAAGATTAGTGACAAGTTGGCACAAGTTG
AGGCTCGTATTTTGCCTCCACCTCGGCTTAAATATCATGATAACGGTCGAGAAAAGGACT
GCCTGCCACAAGTTGGCCAATGGAATATGATGAATAAGAAAATGGTTAATGGAGGGACGG
TTAACAATTGGATCTGCATAAACTTCTCACGCAATGTGCAAGACAGTGTTGCTCATGGGT
TTTGCTCTGAGCTTGCACAAATGTGCCAGATATCTGGCATGAATTTCAATCCAAATCCTG
TTCTGCCACCTTCGAGTGCACACCCTGATCAGGTCGAAAGAGTCTTGAAAACTCGATTTC
ATGATGCTATGACTAAGTTGCAGCAGCATGGGAGAGAGCTTGATTTGCTAGTTGTCATCT
TGCCTGACAATAATGGATCTCTTTATGGTGATCTGAAGCGTATTTGTGAGACTGAACTAG
GAGTTGTCTCACAGTGCTGTTTGACAAAACATGTATTTAAGATGAGCAAACAGTATCTAG
CCAATGTAGCGTTGAAAATAAATGTGAAGGTGGGAGGGAGAAACACTGTGCTTGTTGATG
CAATATCGAGGCGAATTCCTCTTGTCAGCGACCGGCCTACCATTATTTTTGGTGCAGATG
TCACCCACCCTCACCCTGGGGAGGACTCTAGCCCATCCATTGCCGCGGTGGTTGCTTCTC
AAGATTGGCCTGAGATTACAAAGTATGCTGGTCTGGTTTCTGCTCAAGCCCATAGGCAAG
AGCTTATTCAGGATCTGTACACGACTAGGCAAGATCCTGTTAAAGGGACGGTTGCTGGTG
GAATGATTAAGGACTTACTTATATCCTTCCGAAGAGCTACTGGACAAAAGCCTCAGAGAA
TCATTTTCTACAGGTATGGTGAAATAATGCGGTTACCAGTGCAATATGCGATTTTTCCAT
TCTTGTTCTTTTGCTTAAATGAATTACTTTCTCATTTTGGGATGGTGTCAGTGAAGGATA
ATTTTATCTAGTGCTTCTTTTCGAACTTGATGCCATCCGCTAAGTATGTTCCTTTCTTTT
TGTTATTTATTCTTGCTTGTTTATTCTTAAGTATAGATTATGTGTAATCTGCTCATTTCC
TTTTCCTACTTTTATTTATTAGAGGTATTGCACTGACTTGAGTTAATTCAGGCATGTGCG
TCTTTGGAGCCAAATTATCAGCCCCCAGTTACATTTGTTGTGGTTCAGAAACGACATCAC
ACAAGGCTTTTTGCCAATAACCACCGTGACAGAAATGCAGTTGACAGGAGCGGGAACATT
ATACCTGGTACTGTTGTAGATTCAAAGATATGCCACCCGACAGAGTTTGATTTCTATCTT
TGTAGCCATGCCGGCATACAGGGTACGAGCCGTCCAGCTCACTACCATGTTCTATGGGAC
GAGAACAAATTCACAGCGGATGCGCTGCAGTCTTTGACCAACAACCTCTGCTATACATAT
GCAAGGTGCACGCGTTCCGTCTCCATCGTTCCCCCTGCATATTATGCACATTTGGCAGCT
TTCCGTGCTCGATTATATATGGAGCCTGAGACATCTGACGGTGGTTCAGTAACAAGTGGG
GCTGCTGCTGGCAGAGGGGGTGGTGCAGGAGCTGCTGGAAGGAACACCCGAGCCCCAGGT
GCTTGTGCTGCTGCTGTTAGGCCTCTTCCTGCGCTCAAGGATAATGTGAAGAGGGTTATG
TTCTACTGCTAGTCTTAACAGCAAGCTATAGTTTGGACTTTATTTGCGTGCATCCTTTGG
AGCCATTGACTGTTAATCATGGCTTATCATGACTGTTTGGTTTTTGCGTTTTCCTATAAA
TCATAGTTGTAACTGCTTGGCTTCCCCTAATATTTGTAACTATTGATGCCGGAAAACTTT
TTAATGTGTGATGTTGTGGTTTTAAGCAGTGCTCTCCTTAGATTCCTCGTGATGATATTT
TTATTTTTGCACTGAATTTTAACGGTGCCATCAACAGGGTATAATAGATCGAAACCATCA
GTGCTTGTTTA

>comp72459_c1_seq2 1230_1673
CTCTCATCTTTTAAAAAAAGAAAGAAGTCAGAAATGGTTAAATTTTTGAAAACTGATGAT

GCCATTTATGTGTTAGCAAGCTACTCTACTATTTACGTTAGCTAGTATTGGAATTTTCTT
TTTAATTTTTAAACAGCAGAAGCAACATGTTTTTTTAATACTATCTCTTTTATAGAGAG
AGAGAGAGAGTTTGAAGGAAGGGAACATGCTTAAAATCATCACAACAGACGACTCTTGTG
CTTATTTTCCTTTGTTTTCCTAATCTAAAGTTGTGCAGCCTGCTACTCCTCTTTGTTGTT
CAACCGGATTATGCCTAGGAAAAATTATGTGTGGATAAGCAAATGGCTGTTCGAGCTCCA
GCTTTACCAAATAGAAGCTTAGAGCAAACATCCACCTGCTTTGTGCCTCATTTTGCTGTC
AATAATTCAATATGGTGGAGTTCCAATGAAGTACAGTCTTCACATTCTCTTTTGAAAAAT
TTAACTTTAGAAGTGGTACCGCACTGTCAGCAGATGAAGCCTCTTGGTCTGGGATTACAA
GGCGACGACTCATACTCCACCCAATCAAATAGTAACTCCCCGCTAGAAGTGACTGCTTTG
GGGAAAACAGGTTCTCAAGATCAATGCGCTTCTCCTGGATCTGTTTATTTGCTTTCCTCA
GTTCATGGCGAGAGCTACAAAAGGAATAAGCAAGCTCAAATCAAGACTAATCCTTTTATT
AGATATCCAGAATATCCGACCGATTGTTCACAAAGTGAAATGGGCCATTCAATTAGCCAC
GCTCCTTTTGGTTACTCCGACCCCTATCTCAGTGGCTTTTATGCTGCTTATGGACCTCAT
GTTTTTGTGAGTGCTGTAATGTCGTATTTGCCTCTCGTATTGCCTGTCCTCAATTAAAAT
GCAATTAATCTTGATTTTCCTTTTATCTTCCTATCCTTATGTAAATGATGTTTTAAAACT
TCAAGTTGTTTGATTTTACTAAAATTGTACTTCATCCTTTATTAACCTGAAGAGTGCTTA
AGCCATTGTATGTAGGAATTATCATTGTTTTTCTACTTATAAGTGCATTATTTGTATTCC
GAGTCAAGCTTTAAACTCTTGATACAATCTGTGCACTAGGTGATTTTAAATGTATTTGTT
CGGAAAGGAGAACGGCAATGGATATTTTATTTTGACTCTTCTAGTCTCAATATAGTTTTA
ATTCTGAAGTTTGATCAATTCAGCCTCAAATGATGGGGATAGCACCTGCCCGCCTCCCAC
TACCCCTCGATCTTGCAGAAGATGGACCAATTTATGTAAATGCAAAACAGTATCATGGGA
TCTTAAGGCGAAGACAGATACGTGCTAAGCTTGAGGCTCAAAATAAACTTGTCAAAGCTA
GAAAGCCATATCTTCATGAATCACGGCATCTTCACGCGGTAAAGAGGGTTAGAGGGTCCG
GAGGGCGTTTTCTCAGCACAAAGAAGCTTCAAGAATCAATTCCAAACGACTTGAATTCTT
CTCAGGATTTGGTTACACGTGAGGCTCACACTTCTGCTTTCTTGAGCGTCCGACAGGATG
CTGCGCATAACAGCGTCATTTTCCAGCAGCAGCAGCATGATCACAGGGCCTCAAGTCTAT
CTTCTCACATGGCTGTTTCCCTGCAAAGTAGTAGAAGTACTGGCATTTCTTGAACTCACA
GTTGTCTGGTGATTTAAGTTTCAACTCACCGGCGGGCAATTCATCCTTGGCTCTTTTACT
CTTACTGTTATGTCTTCCTGGTTACTCTAATTTTTTGGTAAGGTGTTCTCTACTTATTCT
AATTATCAACTGGATTTTCCACTTTTGCAATGGTGTACTTATGGTTGAGCTCTAGGTGCT
TGCACCCAACTTTATGGAAAAGTTAAAAGTTCTCTTGTTATGTTTAATGGCTTGTTGTTG
TTACTGCTTGTAGTTACTGTAAATCTCACTTTATATCTTTAAATTCAAACTATTTACCTT
TTAATATTCAAAAAAA

>comp72338_c0_seq4 1046_1396
ACAAACTTGTCAAAAATAGAAAGGTATTTGGGTACTTTTTGCTGTTCCCCATTTTAGTAC
TCAGTTCATTAGCTTACATCTGTCTCATTTCTTCTTATGAACTGCACTATGACCCGTACA
AATTAAAGAAAAAGAGAATAGATTAAAAAGAGGGTAGCAAGATGATAGCAGTTTTGAACT
GTCTTATAATTTATTCTTTACTCATGCATAACGATATTTCCTTAGTAACGACTTAACAAG
GAATTTCCCTTGAGAAAATAAGCAAATTATATGTATCCATGTATGTATAAAGTAATTCAG
TAACAGTTGTCAACATAAGATTGGAATTTTATAGCAGGACATTCCGATATTAACAAACTT
TCATCAGCCTGTAATATACGTAACAGAAAGCTACTAGTACGTACTGTTCCGTTAATGTGT
GTGGTGCATGAGCTTAACCACTCTAACCAAAGTCTTTTCTTTTGGAGAAGTTAGTGATTC
TTGTTTATGAAGTGCAAGTTGTACAGTACAGGCAGGTATAAAGCAAATAGTAGTTGACCC
ATGTGAAATTCTGAGGCCAAGCAAGCTGCTGCAATTTGTTTTCATGCAGTTATATTTGGG
AACACACAGAGATGTGATCAGGCGTTGTATCATTATATAAATGTTCAATATTACTGAGGA
AGTCATGATTCCCCTTGTCTTCAATAATTGTCTGCTGTCGTATTCAAGTAGGGAAAGATT
CGTCATATCAATCATTTGTGGTAAAAGAAACAAGCAAATATGCTGGTTGGAGACATTTAA
GGAAAGGAAAAAAATGAAATTGGTAACTTCTTCCCTTGGTGGGGGGACGGTAGCTACTAG
TGAATTTTGCATTTCCATATTTTAGGGGATGGAATGGAACAATATAAACTTTTCAAGCTG
GTCTATAACACCAGTAACCAAACAGGACAAGATAAGAAAACTCAAGTGGGGTGACTACCA
TTAGGAAATAGGAAGTACAGGGTAGAGTGTTCCCAAAAAATAGGCTTCTAAAATTTGTTG
CCTTATACTTCTATCATTCTTGAAGATGAATTTTGGGCAGCTTATTCTTCTGATCTTTTA
TTGCATCCAGCCATATCTTCACGAGTCACGGCATCTTCATGCAGTGAATAGAGTTAGAGG
GTCCGGAGGACGTTTTCTTAGCTCAAAGAAAGTCAATCAATCTGATCCAAATAGTCACCC
TACTAACTCAACTTCTTCTCAAGACTCAATTGAGCGTGAAAATTCAACTTCTGCTTTCTC
AAGTGTCCGGAAAGATGTCAACAACGGTGTCAGTTTCCAGAAGCAACCTGATCACAGGGC
CTTTGGTGTATCCTCTCACATGGCTATTACCATGCAAGGCAATGGGACTCAGCAACGTGC

TCCAGTTGTCCGGTGATAATTTCTTAACTGGCAAAGATAGTCACCGGCAGGCAAATCATC
CTTGGCTTTATCCTTTCTATTAACTATCTCTGGTTTTTGTAAGTGTCAAATGGATTCCAC
TGCTGTAATAATGAATTTGGTTTGTTAATCCTTGATTTGAACCTAAGACTTAAGAAAGTT
TTTGGTACTGAATTAGTTTTCTGTTTTGGTTGCACGTCCGCACCAAGATAAACAAAAAAC
GGTTGCTGGTTTTTCCAAGTGGAACAGCAGGTTGATGTATATTGCCAAAATTTAATCAGA
GAAAGTTGTGCAATGTGTTCTGTGGACTTTCTTGAACCTGAGCTAGCTTGAGAAGTGTTT
TCGAACTATTTAGACAGGAGGTAAAATTTTCTGCTTGGATTGTATTAGTATATCTTAGAA
CCAACACCTTACTTGGATTGTAATGTGTCGGTCAGCAGTGAAACCAGAATTTGAACGGTA
TGGACTCTAAGTTCTAAGATAGCGACATCAGATGTTAGTAATTGAG

>comp73705_c1_seq8 1254_1739
TGCCACCAACACTCTTCCTCAACCACATGTGCACCATAATCATTCAATGGCTTGCCTATC
CTACCCTTGGGCTGACACTTACTTTGGAAGGCTTGTGGCTACTTATGGATCAAATGCTAT
TGTAAGTGACATTGACTACATGCTCTTTATCTAAAGCAAATTTTATAAGATTGCGGTCTC
TTTATATTTTCTTCATGATTCAACATCCAAAATATAAGGTTACTAGAGTGAAAGAGTCGA
ACTTAGACTAGTTTATTTCTGTCTGATGCTACCAATGTCATTGCAACTCAAGTAGCTCAC
ATTCTCACCTTTTTATGTTGGGAAAATTACTCATATTCCATGTCAAAATGTGTCTCTTTA
TGCATTCATCTTTCTTTCTTTCTTCTTGTATCTGGGAGGCTGTGCTTGTCATAAATTTCT
TTGTTTGTCAAAGAGTAGCTATTAAACTCGAGGCAACTGATTTTGTTTGTGGCACAGCAT
CTTAAGAGGAATAGTGAAGCTAATACTAGTCATCTTTGAAGCACTTCTCTAGCTTTTGGA
ATAACCTGAACACGACTTGAGGTATAGCAGAAGGAGATAGCAATCTTGTGATCGAGTTCC
AGTTTTTGAAGTTTATCAACTTATCAAGTATCATTGGACGCTCCTCCTTTACTGATGTTA
CCTTTGTTTTCTCCCTAAATCTAGAGTCCTAATACTATGAAATTGGTCTGCGCACTAACA
AGCTGAAAGTAATATAGACTGCATGGAACTATTGAAGTAACAATATATTCTTACTGCATA
ATCTCCATATAGACTTGTCATTTAAAATGCTAATTAGATTGCTGCTACTTGGTAAGTTGG
AAAGTGATCTATTCTCTACTCATATCTTTTTCCAAGCAAACCAAAACGAATCTGATATGT
GGAGATACAAATGATGCACCAAAATAGCTCTCTTTCATGATGTCCACCGTCCTTTTACCA
CATGAATCTTGTGATTTGCAAAACCTACTGAATTCATCATGCAACTAACTTGTGCCATCT
TTTCCGAGCAAGTAGCACTTGTACGTGCCAGTTCTTCAAAAGTAGACAGTAGTAATTCAT
TCTGCCGCCTTTATTGAAATGAAGATCTCTTCATCTCATTGAATGCTGCTCATGTTCAGC
TCCTCAATCTTCAACTTGAATGAAAATAGAATTTATTTCACTTCGCACTGAACTTTTTGC
TGTTTTTATTTAATTCCTTTTCCGATGTTGGATATATCTAGATTTATCCTCAAATGGTTG
GTGTCACCTCCACAAGAGTTGCACTTCCTCTTGAATGCACTGAGAGCTTGCCCATCTATG
TGAATGCGAAACAATACAGTGCTATCCTCAAACGACGTCAAGTCCGTGCCAAGCTGGAGG
TTCAGAATAAGCTTGTCAAAGACAGAAAGCCATATCTTCACGAGTCTCGACATCGTCACG
CAATGAAGAGAGCTAGGGGTACTGGAGGGCGCTTTTTGAACACAAAGAATATGCAGCAAT
CCAAGCCTTCATCTCCAACACACGACAAAAGTATTTTTAAGGGTACGGCAGGTGGCAACT
TAACTAGATCCATTGTTCAGCACTCAGAAAGTGGTAGTTGGGGCACTTCCACCCAATCTG
GTTCCGATGTTACGAGCATCTTCAGTGGTGATGACATGTTCCAGCAGCCAGAGTTCAGAG
TCTCTAGTTTCCCTTTCCACATGCAGGAAGCTGCAGACTTCATGCATGTTGGAACTTGAC
AACACGTTGTATTATCCATGATGTCGGTGCCAACACAGGCTGCATTACAAAATGCATTCT
TATTGTTTGCACTCAGATGGATAATCGGCAGTTCATCCTTGGCTATGTTACTCTTTCCTT
TTCCAGAACTGAAATCGTTAACATCACTTTTTCGTAGGGTTTGTTCTTCGGTATGGTATA
CAGTTCATGGTTTGTGATGAATAGATCGCAGGTTGGTTCTGCACGTCATCAAGAAGGACA
ATGAGAGAAGTCAAACCATTTAGACTGTTCTTTATCAGAAACTGTGGAGATTAAGGCAGT
CTTCTCCTTTTCTTGGTGGAGCGACGTGTAGATCATGCTGCATTTCTAATTCTGTACTTG
CTACTGTTATTTATGTTACTGTTTTTCATTTCCTGTTGCTTTGTATTGCTCTCATTGTTA
GTACTTTTGACTTGGGCTTTGTGTGCAACAAAATAAGCTTATTCAACCATTTTCATGCAC
TTCTGTGCTTGTGCAATTGAGCGGGTGATAACTGCACAAAATAAGGGGTTTAATTTTAAC
CCGGATTTAGTGCTTACAATTCAATTTCTTTTATTCTATTGGAACACTTCGTTTGCTTAG
GAGACAAGGTATCCCATGATTAATTATTTTGGGATTGTTATCCTACCTTCCCATAGGGTT
GAAATAACACTACAATCTCGGGATTAGTTATACCACGATTTTATC

>comp67524_c0_seq9 544_1167
CGGTTGGAAGTGGGGGTTGCTAACAACCCCTCTTGTCTCACTCTTGGACTTTGATGACCA
TAAATACTCAAATAATTTGTGAGGCGTATATACAATGAATCTCCAAATAGCACATTAGGT
GATGCTCATGTGGACCTTAGTTTAAAGGCTGTGGTAATGAGTCATGTGGAACTTAATTCG
AGAAAAGAGATTGTTGGATACACAAACAAAATTACACTTTGGTAGCTGAAATAAAAAGAA

AGCTACATAAGACGTACAAATATTTTTAATAGTGTGACGCCTTTTAAGAAAAATAGTATG
AGTTAGGTGTAAAGCGTACAATATCAACCATATTTAAGAGTATACATTTGGGCTATCTTA
GTCAAACACCTGAAGGTAAGGTCATGAAGAGTTTTGAAGGAATTGATTGGAATTTTATCG
ACCTACTCAAGGCTCACTAAACAAGTGATCCCAAATCTTCACAAAAGGCAGAAAAGCTTT
CAGATGGTGTTTCTATGAAATCATCCAATATAGACTGTCAAGGCCACTTTGAGCCCCTGA
TTTATGCAAAGTATCCTTATGGAGAGAAGTGCTTTTCAACTTATGGTTCTCAAGTTTCGA
AAGTATAACAGAGTTTCTATTCTCAAAAATTTCAAGGGCCGCGTTATGCTACCATTGAAT
TTGACCTCTGATGAAGGCCCGATATTTGTCAATGCAAAGCAGTATCATGGAATACTTAGG
CGCAGAAAGTTTCGTGTAAAGGAAATGGAGAATAAGTTTCTTAAACCGCGCAAGCCGTTC
TTGCACCTCTCGCGCCATCTCCATGCCATGCGTCGACCTAGGGGAGGTGGCGGCCGCTTC
TTGAACACAAGAAAGGTGAATGGAAGTACCAATGATGGTACAACCAAGACTTGTAACAGG
AAATCTGAACCCAATGGATCACAGAATTCTGAAGTTCTGCAGTCTGATCATATAGGCAAT
TTTAACTCGCCAAACGAAACAACAAGCAGAATGTCCTGTAATTCTTCAGAGGTTACTAGC
TTCTACTCCAGGGAAAATCTTGATACTTATACAGTTCATAATCCTAAGCCATCAATTCAG
GCTTTTTTTGATATGATGAATACTGTCATGCCCAGTAAGTGGGGTTCTGCAGCAGCAGAT
AGTTGCTGCAACCTCAACATTTGGTAACGTCGTCCTTACTACAACCAAGTTGACATCTCT
GGACTATGTTCCTACCGCGGTTTGGTTGCTAGAATAGGCAGATCATCCTTGGCTATCGAC
AGGCAGTTCATCCTTGGCTTATCCGCATTAAGGTGTCGCCTCTATCATTCCATTAGCTAT
TAAGCTATGTGGATGTTACGAGGTGCTAAAACCTTAATGCAGTTTACTAACAGCAATAGT
AAGACATACATCTTAAATCTAAATAAAGTTCATGAAGGTCCAAATAATCAGGTTTGCGAT
GTTGTGCATATGTTGGTTTGAAAAAATAGAGTTGATCGTATGTTGATGATTAAACTTGAT
ATTCACTAATGTGTAATTTGGTGTGTTTTAGACTGGAAGTGTATTTGGATGGAAATGAAG
TATTTATGTAATAATATTTTGGATATAAGTTTGTATAATATGTGCAATGTCTTCTACTCA
TAAATATATAAG

>comp76988_c0_seq2 277_1911
TAGGCCCGCACGCACGCAAAGATAAAAAGCCAGCAATCATCATCCTCTCAAAAAGCCTCC
ATTTTCTCCCAAAGTTTTCAGAGCCATTGAAGAAAGTAGAAGGCAAAGCTAAAAAGACCA
CACCCACCATACAAAGTCTTCTTCTATTAAAGCAACTACAAACCATAACACGCACTACAA
ACACTCGCGCAAAAAACAATTCACTCACTTGTTAGTTACATAGTCTTTACAGACTTTGGC
ACGGTTACCAAATAATCCTCTCTTTTTCCCAGAGAAATGAAAGTGCCCTTTTCCTCTAAT
GACAACGTGAGTTCAAAACCATTGATAAGCAATAACAACAACAACAATCAGCTCCGGAAC
GCTACTTTTCCGGCCACCACCAACGGCCCTAATTTGTGCTACGAACCCAAATCAGTCCTC
GAACTCCGCCGCAGTCCCAGCCCTATCGTTGAGAAGCAGGTGATAACAAATCCTGATTTA
TCTGCCCTTTGCGGCGGCGACGATCCTCTCCAGCTAGGTGATCATGTTCTGAGCAACTTC
GAAGATTGGGATTCTTTGATGAGAGAACTTGGTTTGCAAGACGATTCTGCTTCACTTTCA
AAATCTAACAACCCTCAACTCAGCCTTACACAAAGCGAGTCGCTGACTCAGTTTCCTAAC
CTCTCTGAATTCTCAGCTGACTCGAATCAGTTCCCTACATCTGATTTTTCTTTCTCTGAC
AATTTCCCACAGCAATTTCAATCAGTGAATCAGGGAAATTACATTAACGCCCTTGATCTC
TCCGGTGATATCCACCAGAATAATTGGAACGTAGGATTTGATTACGTGGATGAGCTCATT
CGTTTCGCTGAGTGCTTCGAAACCAACGCGTTCCAGCTCGCACATGTGATACTGGCACGG
CTCAATCAACGGCTCAGATCCGCCGCCGGAAAACCTCTTCAACGAGCTGCCTTTTACTTC
AAGGAAGCACTCCAAGCCCAACTCGCTGGGTCAACTCGGCAGACTCGCCCTGCCAGCTCA
TCCGACGTGATTCAGACCATCAAATCCTACAAAATTTTCTCAAACATATCTCCAATCCCT
ATGTTCTCCAGCTTCACAGCAAATCAAGCCGTGCTTGAGGCAGTCGACGGCTCGATGCTA
GTGCACGTCATCGACTTCGACATCGGACTCGGAGGCCACTGGGCTTCCTTCATGAAAGAG
TTAGCCGATAAAGCCGAGTGTCGTAAAGCCAACGCGCCGGTTCTTCGAATTACGGCTCTA
GTTCCTGAAGAGTACGCTGTAGAATCGAGGTTAATCAGAGAAAATTTAACACAATTTGCC
CGTGAACTCAATATCGGTTTCGAAATAGATTTTGTGCTAATTCGAACTTTCGAGTTGTTA
TCCTTCAAAGCAATAAAGTTCATGGACGGAGAGAAGACAGCAGTGCTTTTATCCCCCGCT
ATCTTCCGACGGATCGGATCAGTGTTTGTTAACGACCTCCGTCGTATTTCCCCTAACATG
GTGGTACACGTGGACAGCGAGGGGCTGATGGGTTACGGAACAGCGTCGTTTCGTCAGACA
GTGATTGATGGGTTGGAATTTTACTCTACGTTGCTGGAATCACTAGAAGCAGCAAATATC
GGTGGCGGAAATTGTGGGGATTGGATGAGGAAAATTGAAAATTTCGTGCTGTTCCCGAAG
ATAGTGGAGATGGTGGCGGCAGTAGGGCGTCGAGGAGGTGGTTCGTGGAAGGATGCGATG
GTGGCTGCCGGTTTCCGGCCGGTGGGATTAAGCCAGTTTGCAGATTTTCAAGCAGATTGT
TTGTTAGGGAGAGTACAGGTTAGGGGATTCCACGTGGCAAAGAGACAAGTGGAGATGTTG
CTGTGCTGGCATGATAGGGCCCTAGTAGCTACGTCAGCTTGGAGGTGTTAATAATAAAAA

CTACTAACCCTAAACTAGTTGTAATAATAGCAGTGATTTCGATCTTTAGCTATATGGTTT
TAGTTTTAGGGGTGGGCGTAGCATAACAGCAGTTAAAACTCAAAAGAAACTTTAAGAAGT
CGCATGTTTTTTTAATGCTTTTTTTGTTTTTCTTAATTGTGTTGGCACTATCTTTTTTTG
TAATTTTGTAGCTTAATTTCGAGTCTCAGGTTGGTCTCTCTAATTAGTGGATAATTGCAA
TGTGTGTAACTTTTTCGCTGTTAATTTTAAAGAAATCTCCCAGCGAATCGCCATGTTAAT
ATGTTATTTTCCATGTTTTGGCATTTGCATCTATATTCAG

>comp80119_c0_seq4 672_2573
CTGGAAGAAAAAAATCTTTGAGCTTCTATCAGATTGGTCCAATATAGAAGAAATATTGGGC
TTCCTTGGTTTATGCTTGTAGGCTTACTGATTGAACTCTGATCCGAAAGACCAATACAAT
CCAGCCCCTTTCCTTTTACAAACATACGCAATATCGAAGGAGCTCTTCTTTTCTTTTTTC
TGATGGAACACTTTCCTTTCTTCATCTGAAACAATCAAAAACCTATATTTGTCCTTCTTT
AGCTCAATCTTCTGCTCAATCAAAGTACTCACTACATTCTAATCTTGTTAGAAAAATTAA
GATCCATCTCGAAGTTAAAGATGAAATGGAGACCAAGTTCATATTTTGGAGCCAATGGCG
TCACTGCCTTCAAATCTTGGTAAAAAAAAATAAGATCCACCAAGTAGTTAAAGGCAAGAAA
CCTAATTATTTACTGTTAACTTGTTTCTTTGTTATGGTTATTTTGTGTTAATTTTTGTAG
TGGGTTAGCTGATTTTTTTAATTTTTTTTAATTTTAATGTTCAGATGATTGTAATACCTC
AATGTATTCCTAAATCCTAACACCTAAAGGGGTTGTTGGTGTTTCTGGTTTTGTCCCTTT
AATTTATTCTTCACCAGAAGCACCAATTTGCAATAAAGATTCAAACTTTACAAGAAATTG
AACTTGTTTCTATGTTGGTTACGACAAGTCACAGTCCATCAGCTTCATCTTCCTCATGTT
CTTATGGTGGAAATATGGGAGAAAAATCTGATCTTGGATTTGAGGATTTGGATAATTTGT
TGCCTGAACTTGCCGGTTCCGACCAGACCCTTTTCCGGTGCATTTCCGGCGATATGGAGG
ACCCATCAGTTAGCTTGAAACAATTAATCCAAGGAGGAAATGCAAATGCTGATTTGGGTT
GTGGAGTTTCAGTTGCAGGTTCTTTTGCTTGTACTGATAATATTTCCTTTTCTAGTTCAG
ATATTTCTTTAGATGCCAACATTGAGAAAATTGGTTCTGTTGTCATAGACTCAAATAACA
GACCAAATAATAACTTTGAAAATCCGAATGTCAGTCTTTTGGCCAAGAGTTTACCTCCTT
CTTTGAGCTTTCAAGAACAACAATCAGAACAGAAGCCTCAAATTTCTTGTCCACAAATAA
TGGCAAACCAAACCCAGTTCCAGAATTCCGCTTATGTTAACTTGTTTGGCTCATCATCAT
ACAACATGAATCAAGAACAGCCGCCACCCAAGCGCCACAATTCAGGTATCCTGGGTTCAA
GCTTAGGCTTTCTATTGCCTAAAGTTCCATTCTTTAATCCCAGTGGTGACTTATTGCTGA
GGAAACAACCACTGGGACAAATTCAGCAACAAGTCAATTTGCTGCCTCCTCACCAGTTTC
AGCCAACACCATTATTTGTACCTAAGCTTGAGGCAGCTGCTGGTGGTGGTAATGGTAATG
TGATGGTGCCTCGTCATCAACAGCAGGAACAACAGTTCATTTATGACCAGATTTTTCAGG
CCTCTGAATTATTACTGGCCGGACAATTCTCAAACGCGCAAATGATATTGGCGCGGCTCA
ATCAACAGCTCTCTCCCATTGGCAAACCCTTCAAGAGGGCTGCTTTTTACTTCAAAGAGG
CTCTGCAATTGCCTTTCCTTTTGCCTCGTACATCCACATCTTTTCCGCCAAGAATTCCCA
CCCCATTTGATTGTGTGCTTAAGATGGATGCTTATAAGGCCTTTTCTGAAGTATCTCCGC
TTATTCAGTTCATGAATTTCACCTCCAATCAAGCTATTTTTGAAGCTCTTGGGGATGCCA
AGGAAATTCACATAATAGATTTTGACATTGGCTGTGGTGCTCAATGGTCCTCATTTATGC
AAGAACTCCGGAGCAGCAATAGAAAGGCAACTTCCTTAAAGATTACTGCCTTTGTATCTC
CTTCAACCCACCACTCCGTTGAGATTGGCATCATGCACGAAAGTTTAACGCTGTTTGCTA
ATGATTTGGGAATCAGATTTGAGCTCGAAGTTATTAACTTGGATTCCTTTGACCCTAAGA
CTTATCCCTTATCCTCCTTGAGGTCATCTGAGTGTGAGGCTATTGCTATTAATTTCCCCA
TCTGGTCTATTTCAAGTTGTCTATTTGCATTTCCTTCACTTCTTCACTGTATGAAGCAGC
TTTCACCAAAAGTTGTTGTATCATTGGAACGTGGATGTGAACGTACTGAACTCCCCTTAA
AGCATCACCTCCTCCACGCCCTCCAATATTATGAGATACTTTTAGCCAGTATTGATGCTG
CTAATTTAACACCAGACGTTGGGGAAAAAATTGAGAGGTCTCTCCTCCAGTCTAGCATTG
AGAACATGGTCTTAGGGCGCCTCCAATCCCCTAACCGAATTCCCCCATGGAGAAACCTAT
TTGCTTCTGCAGGATTTTCACCTATTGCGTTTAGTAATCTAACTGAAATCCAGGCAGAAA
TCCTTGTTAAGAGAACTCAGGTAGGAGGATTTCATGTCGAGAAGCGCCAGACGTCACTTG
TGCTATCCTGGAAGCAGCAGGAACTCTTGTCAGCTTTGTCTTGGAGGTGCTGAGGAGCTT
TATCTTATGAAGCCAGGCGACTCTTTCTTTTAACAGCAACATTTACTAGAGGTTTTGACT
CTTCCATTTGATGTCTGTTAAAAAATCTGCTTACATTTGCTATGTCATTTTCTCCTTATG
TGTAGTCTTTGACTGTAGGCAGGCCTTCCGCGTTAAACAAATCTTAACTGTTATCCCTGT
TTAAATATCAGTGTGTTGTATCTTCCTAAGTTTCACTATGTATTAATTTCTCTCTTTTCC
ATGTTCTGATACAATTGCTAAGTAATATTAACAATTTCCGGATGGTAATGATTAATGACC
TGCTTT

>comp75200_c0_seq2 459_2717
AGACAAGCCACAAAAACTAAAATAAAATACCCTTCTGAAAAAAAAAAACGAAACTTAAAA
CTAAAATGAAAACAAAAGTTAGAGAGAGAAGATGAAGTGCACTTTCTTACTTTTATGTGT
TGCACGCATCCATGGTATAAATGAGAAGGAAAACCAGAGTACTATTATAAGGCCATCAAC
AAAAACAGAAGAAAAGAAAATAGCTAGTAGTTAGTAAAATTCTTATTAACATCAAAACCC
CTTTTAACACCCTTCACAATCTTGGCTTAGCTAGTTTGTCCCATTGTCCAAGAAGAAGAA
GAAGAAGTACAAAAGAAGCATCAAAGAAGAAAGAAGTTATTTTGTGGCTGCTTGACCTTA
GATGGACATCTTCAGTTTCAAGGAAATATTTTTTCTTTGAAGATTTTCTAGGTGATATCT
TTTGATTTGAGGCCAAAATTTTTGTGGGTATACTTTAGATGAAGGGGATGCCCTTACCCT
TTGATTTTGAGGGGAAGGGGGTGTTAGACTTGGACGTTGTGTTAAATAAAAATAGCATCT
TGAATTCTTGGAACAACAAAAACAACAATAGCAAAGAAAGTTGTTATCTTGTAAATAGTC
CAAGTGCTGTACTGGATATTATAAGGAGTCCAAGGCCTGTTAATTCGTCTTCAACCCTGT
CTTCATCTTTGGGTGGTGGTGGTGGTGGTGGCACTGCTTCCACAGATACGGCATGCGCAG
TTTCTGCAAACCCATCTTCTAAATGGCAGCAGCAGCAACAAGATAACACCACTGCCACCA
GTTCCAATGTAGGAGGAGCTGCTGCTGAATCTGAGTTTCAACAAGTTGCTGCTGCTACAG
AAGGGAAGAAATGTGGTATGGAGGAGTGGGAAGGTGGGTTATCAGAGTCAGTAATGGCTT
CTCCTTGTCAAGAACAGTCTATACTTGGGTGGATAATGGGTGATGTAGATGACCCCTCCA
TGTCTAACTTGAACAAGGTGTTGCAGGTTAGCAGCCCTATGGACTATGAATTCAATGCAG
GATTTGGGGTTGTGGATCAAGGTTTTGGTGTTGACCAAATTGGTACTAGTAGCTTCATGC
CTGCAATTAATAGCTCTTCTGTTTCAAGTTTTCCTCCTACTACTACCAGAATGAAAAGTG
ATAAGATTGGCTTGCTTTCTAACATACCAACAAATCTTCCTCAAAATCCAATCTTTCCTT
CCTTCTCTAACAATCTTGGCCCAGTTTCCTTCAGCCAGACACAACAGCAGCCATTTGAGA
GCACAGATTTGAAGCCTCAGAGTTTCCACTCACAGTTCTTGATTAACCAGCACCAAACAC
AGATTCCTCAAAACCCATCATTTCTTTTGCCATTGCCATTTGCACAGCAGGAGCAGAATC
TTGTCTTGCCACCAAAGGCGAAAAGGCACAACCCCGGGACCCTTGAACAACAGGGCTCTC
AGATCTCCCAACAACTGTTTATAGATGCAGGACAACAGCAGCCACCACCTTCCCATCAGC
TCCAGCTGCTTCCCCATTTTAGGCCAGGAGGACCAATAGGAACAAAGCCAAAGATGGTGG
GGGAAGAAATGGGGCAGTTTCATCAACTACAACTACAGCAGCAACAGCAACAGCAACAAG
CAATTATTGACCAGCTATTCAAAGCTGCAGAGCTGGTCCAGACAGGGAATCCAGTACTCG
CGCAAGGGATATTGGCGCGGCTCAATCACCAGCTCTCTCCAATTGGTAAGCCTTACTATA
GGGCTGCTTTTTATTGCAAGGAAGCTTTAGAATTGCTACTTCATACCAACACCAACAATT
TGAACAACCCCTCTATACCATCTTCTTCACCTTTTAGTCTCATCTTCAAGATTGGTGCCT
ATAAGTCCTTTTCTGAGATCTCACCAGTTGCACAATTTGCCAATTTTACTTGTAATCAAG
CCATGCTTGAGGTCTTGGATGGGTTTGAAAGAATTCATATTGTTGATTTTGATATCGGCT
ATGGCAGGCAATGGGCTTCTCTTATGCAAGAGCTTGCCTTGAGAAGTGGTGGCGCACCTA
CCCTGAAAATAACTGCATTGGCCTCACCCTCCACACATGACCAACTAGAGCTTGGACTCA
CTAGAGAAAGTTTGATCCATTTTGCTAACGAAATCAACATAGAATTTGAGTTTGAGATTT
TAAGCATTGATTCTTTGAATTCAACGTCGTGGTCACTGCCTCCTCTAGTCTCAGAGAATG
AGGCAATTGCCGTCAATCTTCCTATTAGTTCGTTTTCGAGCTATCAGCTGTCACTCCCGT
TGGTTCTTCGTTTTGTGAAGCAGCTGTCACCTAGGATTGTGGTTTCTGTGGACAAAAGTT
GTGACCGGACTGACCTACCATTTCCAAACCATGTAATTCAAGTCCTTCAGTCATACTCAA
ACATTCTCGAGTCGCTAGATGCTGTAAATGTGAACTTTGATACCCTCCAAAAGATTGAAA
GGTTCTTGCTCCAACCAAGAATTGAGAGAATTCTAATGGGTCGATTTTGTTCCCCTGAAA
AGACGCAGTATTGGAGGGCACTGTTTTTGTCATCTGGACTCTCCCCGTTACCTTTCAGCA
ATTTTACAGAATCACAGGCCGAGTGTTTGGGGAAGAGAACTCCTGTTCGGGGTTTCCACA
TAGAGAAGAGGCAGTCTTCGCTTGTTCTCTGCTGGCAGCGAAAGGAGCTCATCTCAGCTT
CAGCTTGGAGGTGCTGAGGAAAAGTAATTAAATTTGCAGTTGCCAACCAACCAAGGTGCT
GCAAATTCACCATCACTGTTTTAGTATCTGAACCTTTATGTTTTTCTTGCCCGTAATTTT
CATTTGCTAATTTTTCTTGTGGCATAGGTAGATTGTTTCGGTGTTATAAGAACAATCAAA
CATTCTGTTAACTAGTATTTTACCATAGTTCCTTATCTCAGGGTCTTTGTTTAATTGTGG
TATTACTGTGATGGAGCTCTATAATTTTGTGGAGGTAGGGCATATATCCCCTCTTTCTAG
TTATGCTGAGAACCTTGTTGTACTCTTGGCAACATTACAAAGCCAGAAATCGGGAAAAGA
TGGAATGTTAACTCATTTCATTGACGTGCTTGTGTATATTGCATTGATCATCTCACTAGC
ATGACATGTTTTGCTATTCCATGGGTGAATCTTCTGTTTAAGTTGCTAATTTATGTTATT
GTTCTGTCTGTAGCCCTCTGCCAACCTTATATTTGCTCAAACTCTTGCTCTTCTTGCAAA
TTTGAAGATATATCAT

>comp78340_c2_seq4 552_1409

CTGGGGTTGGGTGAAAAGGGGTGGCTGCTTTTAGGGGCAGTGTTCTAAAATTGGCCTCGT
CTATGTCTGTATTGGGCAGCTAAGAGAGGAATGGCACAAGATCTAGTGAAAATACTACTA
GTAGTAGTATAGTTAATACAAGTGGGAAGCATATATCTATCATATCTAACCCTATATCTC
TCTCCACTTAACTTCTTTTTTCTTTTCCCAACTACTTGAACTCTCTATAAGTCGTAGGTT
TCATGTTCTTTCAATACTCAGCACACAACTTTCGATTTTTCATCATTATTTTATATATAT
TCAACTCCACGCATCTATTTCAGACAGACAAAAGAGAGAGAGACAAAGAGGCACAACTAG
AGGGAGAGAGAGGAGAATATTTAAAAGTTTAGGAAACTGAAAACAAGTCCTTTTGTAGCA
ATAGCCATAACCCCCACACCACCATACCACCCCCCACGCAAGGGTCGGAGATGAGAGGAG
CTGACCCATAAAATATCTGCAAAATTTTTTACTACTATTTTTTATTGTATAGTTTTGTGA
TTCTTGGGTTTATGGAGTGTAGAGAAATGTGGGATCTAAACGATTCTCCAGATCGACGAA
GGGATGATAAATCAGAAGAAGGCTGCTCTTCTCCTATAGAGCTAGAGGGCGATGATGAGA
AAGCTAAACGGGTCGGATCCGTTTCGAATTCAAGTTCATCGGCAGTAGCTATTGATGATA
TTTCGGAGGAGGAAGATGGAGAAAAAGGCAAGAAAAAGAGTAGTCCTAGCAACAAAATAT
TCGGGTTCTCCGTGGTGGGTCCCGGTAACGACGATGAGGAACAACCGGTAACCCGTCAGT
TTTTTCCGGTTGATGAGTCTGAAAAGGGTGCGCCTACTAATGGATCCCCGAATTTTCCCA
TGGCTCACTGGGTTGGTGTTAAATTTTACCAAACTGAGCCACTTGGCAACACGGGAGCGG
GCAAGCCAATGGATGTGGCTCAACAACAGCACCAGCAGCAGCCTATGAAGAAGAGCCGCC
GTGGACCAAGGTCTAGGAGCTCACAGTACCGTGGTGTTACCTTTTACCGGAGAACTGGCC
GGTGGGAGTCTCACATATGGGATTGTGGAAAGCAAGTTTATCTAGGTGGATTTGATACGG
CACATGCAGCAGCTCGGGCATATGATAGGGCAGCTATCAAATTCCGGGGAGTTGAAGCAG
ACATAAACTTTAACTTAGAGGATTATGAGTCCGACTTGAAACAGATGACCAATTTAACAA
AGGAAGAATTTGTGCATGTGCTTCGGAGACAAAGTACTGGATTTCCGAGGGGAAGCTCCA
AGTATAGAGGGGTAACTTTGCACAAATGCGGTAGATGGGAAGCTAGAATGGGACAGTTCT
TAGGCAAAAAGTATACAGTAAAAAAATAAATCCTATTCTCATGCTTATTATTCTGCTGAA
TGGGTTCAATCCATTTTCTTAAGAATAATCCTTTTTTTCTCAATTCTATTCTTTGCTACC
TATTAAGTAACTTTTATTCTTGAATTTTTGTTGAATAGGTACGTTTATTTGGGCCTCTTT
GATACTGAGGTTGAAGCTGCCAGGGCTTATGATAAAGCTGCCATCAAATGTAATGGGAAG
GATGCAGTTACTAACTTTGATCCTAGCATTTATGAAAATGAACTCAACTCAACTGAATCT
ACTGATAATGCAGCAGATCACAATCTTGACCTAAGCTTGGGTGGTTCAAGCTCAAAGCAA
GGAAACCGAGAAATTGGGGATAATAATAATAATAGGGGTCAAAATCCTTCATCTATGCAA
TTTGATGTTGATTGGCGGCAGCAAGGATTAAGGCCTGAGAAGCAAACTGCTCCAGTTGAT
ATGAATGCTCGAAGAAGAGATAAGGGGTACAATGAATCAGAGACGTTGCAGCTCTTGAAC
CAGACACATCTACATTCTCCAGTCTCGTTGAAACCTAATAATAATAGTAGTCAAGTGCAA
CGGTTTGGCCAATTTATGAGACCTGGTGAATCCCATATGATTCAAATGTTTCCACATCAG
TTCAGCTCATCAAATTATCAAGTTCAATTTCCAAGCGGCAGCAATGGAAGCAGAATTGGA
GCTACAAATGTAGGAGAAATTTCGCTGTCAAAAAGCAATGCTTCTTCACAATGGCAATCC
AATTATCCTCCACAGATATTTGCAGCCGCTGCAGCATCATCAGGATTCCCCCAGCAGATA
GTGAGACCTCAATATTGGTCCTCAGAAAATGGCTTCCATCACTCTCTCATGAGACCCTCT
AATTAACCATATTCCACTTCTTCATCATCATATTTCCTTGCCATTTTCTACTTGTGTTTT
TAAAGCTTTTATAAAGAGTGAAGGGTTGAGATTCTTAACACATATTACCCAAGGCTCTTA
ATTTGTTAGAACCCCGCGAAAAAGAAATACAGACAAAATTAGTCTTGAGAAATTCTAACT
CTTTGGAAAATATGTATTTGTCCAAAACTAACTGAAGGAAAGATTTTCAACTGGTCTTCT
ATTAGATTCTTCTGACTATAAACTGCCAATTTAATTTGCAGACGTGCTTTTCACCAACCT
CTCATTGTAACTTGTTTACATATGGAGTATTATATATTAATTTTTTATTCGCTTATTTTT
GTCTTCATTCTTTTACTTGTAACAAAAGCCCAACTACTACAAATTCCCATTTGAGTAATG
AGATGGAATAAAGAAATTAACGTCTTTGGGTAATTGATGTGTAATGAAAAGAGTACCAAA
ACTCTCTGACAATGACGGTACACATCTTCTTTTAAAATGGGGAATTTTTTATTAGTCTTG
TTCATACTCTGAACGTCTCTGATGTCAAATGGATAGCGCAGGTCAAAGTTCACCCTTTTT
TTTCTTCTATTTCTTTTTTAGACATGTGAAAATAAGGGCAGAGTGAAAAGAAAGAGGGGA
GGTAGGCAGATGGGATGGTTTTGTTTTCTGGGAAAGATCACTGGATGGGAATGAATTGTC
ATTGTCAATGGATGTCCTTTGAATCAGTTCTCATGACTAATATGTGTCAGACAAGGCTAT
TATGGTGAAAGGGACTTAACAACAATAACAAAAACCCTAATATAATTTCATTAATGGGTC
TACCGAGA

>comp68096_c0_seq1 79_1191
CAAGTGCCTTAAAAAAACTAAAGAACCCAAAAGCTAGAAGAGAGGAAGGAGGAGAATACT
TGGAATCTGGATCCTCCAATGAATCATCAGCTTCAAATATCAGATGAAGAAGATGGATCA
TCAGAAGAAGAGGAGGAGGATGAAGAGGAAGTTTTTCAAGAAAATGGCATTGAGAACCTT

TATAGTGATTGTCAAAACCAGAACTTACAGCAGCTGCCAATGCCACTCTGCCCAAAACCA
GCGAAATGGGCTAATTTCACAACATATAAAGAAGAACTGAACAAAGTAACAAAAGGTATG
AAGCCAAAGAGAACCAAACAAGATGTGATAGAAGTTCAGAGAGGCCGAATTATTCGATCC
ACGGGGAGAAAAGACAGGCACAGCAAAGTTTCAACTGCAAAGGGTCCGAGAGACAGACGA
GTTAGGCTTTCACCAAACACTGCAATTCAGTTCTATGATGTGCAAGATAGGCTTGGCTAC
GACCGTCCAAGTGAGGCCATTGATTGGCTGATTAAAGAAGCTAAGGTTGCAATTGATGCT
CTTGGTAAGTTTCCTAAACTTAATGCCAAAATCCAGTATTCATTTGATCAACTTTTAGAT
GAAGGCTGTATAGAACAGAGTCCAAGATTTAGTCAGCAAAATTATGGCATTCCAAATTCT
GAATATGGAGTTCAGAACCACCAGCAGGAGGTGAATTATGACATCCCAATTCAGAATGTG
AGCTTATTTTCATCAGCAGAAGGTGCCAAGATTTCATTTTTGAGTGAGTTCCAGAGTTAC
CCACATGACCATTTTCTCAATTTCCAGTCTTTTCAAGAAGACACAATCCTTTCCTCTGGT
GATCATCAAGACAGCTTCTTAACCAGTTCAATTCCAATATATTCCAACTCCAGTTTGGAA
ATGACTAGATATAAAAGAAGTCTGAATGGGAATGTTGCCAGTTCAAATTCAGGAAACGGA
GAGGATTATTCTTCATTCAGTTTAGTAGCACATCATTTCCCATCAGTTTTAAGTCAAAAC
CAGGTTATTTCTCATAGGGAACCCCTTCAGTCCAGCCTTTTTCCTCTAATCAGTGGTGAT
CCATGGAGCACACAACTTCAGACCCTTTCTTATGATGGTTTCTCAGGGCAGATTTCAAGT
GCACCAAGAATTCAAGGGGAAGAGGAGCAGGGAACATTTTCTGCTCTTTGACCCACGGCA
ACTGCATTACTCATTAAGACTCACTACTTCCCTTTCCAGAATCTTTTTTGACTTTTTTCA
GGAGCTGAAATTTCTGCTGAGTGGACCGACGTAATGCAGAATTTCCTATATTATTATCTT
TCCTATATTGGGTACCAGATGTTTATCCAAATTATATAACCCTCTTGTCCCTAATTAAGT
TCTTTTTATTGTGTAATTTTACCTCAATTGGATGATCAGCACCTCATCAAAAGTTCCATA
ATCTCAATTTGGAGGGGCACGAGAGGTGAAGAGGAAATAATAAAAATGACAAGGCTTAGA
TTAAAAGCATGAATAGTTTAGACCTAAGGTAAGAGAAAGAAGAATCCTCGAATTAATGAA
AGGAAGACAAAGATGCGAAAAGGGGGTTATGTATTTG

>comp73714_c2_seq12 2093_2317
GGGGGGGGGGGGGGGGGGGGGTTGGAACGATTAGGATTTTTACCGAAGCTTCGATCCAAAA
TTCAAAAGGCTTGAGTCTGATTTGAGAGAGATGGGATGGAGATTGATGATGTGGAGATTA
AAGGGAGTATGTGATGTAAATTTGGAGTCGATTGGATCATCGCCACCGACGGAGGCGATT
TCCAGTATGCGATTCACGGCAGAGACGGTGAAGGGATACAAGGGTGGCTAGGGTTTGGTT
TCAGAGAGGATGAGAGGAGAAATGAGATGAGTTTTTATTTTTATTTTTTCAGATCGAAG
GGGAAAAATAAACTTTTTCATTCCCTATATAATAAAACATGCTAGTACTAGCTTTTCTAG
CTTATGGGTTTCATTTTGATGGTGGCAAAGCTTACTGATAGAATCAAGACTTCAAGTCCA
GTACAATAAAGGGCTGAGGAGCTAAGAGTTCTGAGCAAGTAGACAGGCTTTGCTTTTGAA
AGCACTGCAAAAAGGAGCCTTCTTTTTCCCTCTCCTCTCTTCTGACATTTGCAACCAGCC
CCCCTCCCTCTCTAGTACCAAACCTGATACATCGAACACAACTTATTTTCTCTTTATTTT
TGTGTGTATGGAAATACCACCGAAAAAGCCAAAGTACTCTGTGTGATCATCATCAATGCA
AAGTACATCCAAAACCCACATCTACCATACATCAACCCATACACCTACCTATCTACACTT
TTTTTCTTATCTACCTTTCTTTTTCTCCTCCATCCCTAAACCCTAATTACAATCCCAAAT
CCACATAGAGTTAAAAAAAAAATACCCTCTTTGCTGATTGGTGTTATTCTCTATTTTTCA
AGAAAGGTGGGTTGGTTTAGAGTGGTTGCTTGAGAAAGGTATGAAAGTGGTATGGAGGTG
GATGAGATTCAAAAACAAGGGTGTAAGTTTCCAAGAATCAGCAATGGAAATGGTAGATTT
GAGTCACAAGATGAAAATGGAGCGGTGAAAGGGAGTCATGGGCTTGGCGGCGGTGGTATT
GGTAGGCTTTGTGGGTGGCCTTCAAATCGGATTGTTCGAGTTTCTCGTGCATCAGGAGGG
AAAGATAGGCACAGTAAGGTGTGGACTTCAAAAGGGCTCCGAGATCGGAGAGTTAGGCTG
TCTGTTAACACAGCTATACAGTTCTACGATTTGCAAGATCGACTTGGTTATGATCAACCA
AGCAAAGCAGTTGAATGGTTGTTAAAAGCAGCCGCTCCTTCAATCTCTGAGCTTCCTTCT
CTCAATGCTTTTCCAGACACACAACAGATCAGTGACGATAAAAGGTCTAGTGCTGGAACC
GAGCAAGGTCCTGATTCAGCTGAGGTTGAAATGGACGGTGATCCGAATTACCGACACCAA
CATCATGTGTCTTTGTCGAAATCTGCTTGTAGTAGCACGTCTGAGACAAGCAAAGGTTCT
GGGTTGTCTCTATCCCGGTCAGAAAATCGCGTCAAGGCGAGGGAACGGGCAAGGGAAAGG
GCTGCAGAAAAGGAGAAGGAGAAGGAAAACGAGTCAACTCGAACTGCACATCACCAAAAT
GTGCATCCAATTTCACATAATTCTTCTTTCACCGAACTATTGACGGGAGGTATGAACAGC
AACAACAATATTAATACTTGTAATGGTAGAGGTGGTGAGACAAACTTGTTTAACAAATCT
CCTAGGCAATGGTCCTCCGCATCTCCATTTGAGTACTTTCCCTCAGGACTTTTAGGTCTT
CGTGGAAACGACAATTCTACTTCTGGTTATCAAGCCCAAGTTCATTTGGGAAATCCTATA
CAACAGCAGCCACAGCCACTCCCTATGTCATCACCAATGTTTAGTATTACGGGAGACCAT
CATCCCGAGCTGCAGCATTTTCCATTTTTAAGTGACCATTTGGGAACTAATGGCCATGCC

AATAATAGTGCTAATGAGTATAATTTGAACTTTAGCATATCATCATCGTCTTCTTCCGGC
CTTGCTGGTTATAATAGGGGGACCCTTCAGTCCAATTCTTCTCCGTTATTGCCTCATTTC
CAGAGGTATGAGGGATCACAAAGTTTCTTCAATGGCAGCACAATAGCTGCTGCTATAGCT
CCCAACAGCACTGCTGCTGTATCAGTTGACAACCATCACCACTTTCTGTCTGCATATGAT
GCCGGCCGCTTACAGCTCTTCTATAATGACAGTAGGCATTCAGATCAGAAAGGAAAAGGA
AAAAACTGAGTTTCCATCCCATCTTCACAAATGTTTTCTCATTTCTGGGTGCTTTTTGGA
TTGACGTGTTATAGTACAATGAAAATGGCTTTTTCATTTCTTGGTTTCAGATAAAGGCAA
TTGAAGATGGAAAGCAGCTGCAACTGAATGAAGCATATGCTTTTCTCTGCTTCAGTGCCA
TAAGATTATTGTGCTTCTTAAATCCTCATTCTGCTTTTGAGTATTCTTATTGGTATTCTC
ATGTTTAGGCATATCAGGTTCTGTGTGTTCTTTTGAGACATAGCGTGTTGTTGATTGAAG
TTCTTCTTTTTCCTCATTCAATTGTGATTCTGCACTCGAATTGTCTGGAAAGCCCATCCC
CTTCAACCAAAAAAAAA

>comp80206_c0_seq2 468_4724
GGGGTAAAAGGGGGTAGGGGTGAGGGATGAATAGTGGTGATTAATGGTACAAACCTGCTT
TTTGGTGGAGAGAAGAACCTGATGCCTTCTTTTGATGTGTGAGTGGGTGGTGCTCTTTTC
TCATCAAAACATACTCTCTGGTCTATGCTACTCTATCTCTTCTTTCCATCTTCTTATTAC
TCTCCTCAGCTACTACTTCTCCTCTTAGTAATACTTTTGGCTGATTTTTATGAAGAAAGG
GTAAAATCTTGGTGTTTTAGTGGTGGTGGGGTTTTGTTTTGTTGTCTGTGGAGAGAGGCC
TTTTAATTAGAGGCTACTGTTATGCTGAAGGCTAAGTATAATAAGGAGATAAGACTAAGT
AGTAGTAGTAGTCTTGGAAAGTATAATAATAGGAGCATCAGCAGCAGTTCCTGTGACTAT
TGGGAAAACAGCACAAGAAAAAGAAGAAAGGAGAAGCTTATCAGCAGATGTTATCCATTG
AAAAATTTGCAGCAGCAGATCCTTCCCAAATCTCTCTACTGAAAAGTAGTAGCAGTGATG
AGAGGTCTTCTTCTTCTGATGATGATAAGAAGCAACTAGATCTGAACCATATTGATGACA
ACAAACCACTCCCCAAATTCTCCTTAAGAGATTATGTTTTCAGAAGCCGGAGAAAGGACA
TAAAGACCAATTGGCCATTTTCTCAGAAAAAATTTGCAGCTTTGTTTGAAGCATGGCGTGA
CAGATTTGTTACCCCCTTTTCAATATGTTAAGGGATATGCAGTAGATAATTGTTCGATTG
ACAAGGACAGAAATTTTGATCAGGAGGAGCATGTTAAGGTAGATGATGATCCTAGATGTA
TGTCAAAATTAGCTGCGGATCATAGAAATATCAGTTCAAGTCAATCTGATAAAGAGAAGG
TGATCCAGTCAACGATAACCATTCAGTCTTGTTCTGAAATTAATTCAGTTCCAACAGCTG
AAAGGAATCCTAGCTTGGAAATAGAAGCTGTTGGGAAATCTGAGGGTAAAGGTCTTTTAC
CTCCCATGTCAGACAAGAGCGGCAGCACAGCACAACCACCAGCAGCAAAAAAGTGCAGAT
TAATAGTCAAGTTGGGAAACGTTACAGATCATCGGACCGTAGACGAGGAAACTACCACCA
GCAGCAATTTCATGGCTTCTGAGGCAATGGCTTCGAAAGTATGTCCAGTTTGCAAGACTT
TCACATCCTCATCAAACACCACCTTGAACGCTCACATAGATCAATGTCTCTCTGGAGAGT
CAACAATCAAATGGACAGAAAATTCTAGTAAGGTGATTAAGCACAGGATAAAGCCAAGGA
AAACGAGATTGATGGTGGATATCTACACAACAGCTGCGTGCTGTACGCTGGAGGATCTCG
ATAAGAGGAATGGAACCAACTGGGCCTCAAACCCGAGTTTGTGTGTTCGAGAGACTGAGG
TATCTGCTGTTGAGAAACTGGATAAACCACCTCCTGTCACTCATGAATGCACTGATAATG
AAGGTGCTGTTTATATTGATGCCAATGGCACAAAGGTTCGAATTCTGTCCAAGTTCAGCG
ATGAGCAGCCACAATCATCAAATCTGATTAATGATCCTCTTCAGAAAAATTTGGTAGACG
GAGATAAAAGAAGCAAATTCATTTTGACAAATAAGAGGAAGAAGAAGAATCATACCCCGA
GACAGCACAAGCTTCTGAAATCTTCTCGTACTAAAAAATTTTGCTTGTCCAAGCCCTATC
ACTGTCCCAAGATTAAGAGTGGTCAAGATAGCACCTTCTCCCCCAGAGAAAATGTTGTCA
GAGAGGATTTCTTAAATACGCATCTCAGATCTCCAGAACAGGTGGTATTAAATGGATTAG
GAACCATAAAACAATGGGCATGTTCGAAGAGAACTGGCCTCACAAGGAAGATCAGTGATA
AGGGCAACCATCAGCGTTCTGGAGGTGTTATGTTGACTAGCGTTGAAGATGGTAATGTTG
CGATGCCTATGACTGATTCATCTTTGAAGATAAGAAGCTATCTTTACAAATCTCCAAGAT
CATCTGTTAATACTGTTTGTTTGCCTGAGAGTAGCCAAAGAAAGGGGGATGTGCTACTTA
AGCCTCAAGATGAACATAGCGAGGAGCTTTCTCTGCAAAAGAGAGTGGATTTTTCATTTT
CTCAATCTCAATTCCCATCTAACAAGAAGAGATCCCTTGTGTTACAAAGGAACAAAGAAA
AGCATTTGAAGGTAGATGTTCATTCTGTAAACAATGGCCCCGGTGATCGTCCAAAAGTAA
CAGTAGATCATGCACTTTCTGTGAAAAATATGAGGGTTGGGAGGAACACAGATCTACTAG
AAAAGGCGGATAACTCTGAGATCAATGGTGAACCGTCGACCTCTCACCCAGCATTCTCCT
TGAAAGCTAGGAAGTTGTCATCATTGAGGAAGAACCTTTTGTCTGTCAGTGAAGGGCCTG
CTCGTGGTGTTAAATGTAGCTTGAAGTGGAAAACAGCTTCCCTGAAGAAGTCTAGTATGC
GCTGCACTTCAGAATCAGAAGAAGCCGTAGTCTGTCAAACTAAAGGAGAGAAACGCTCTA
TAAGAGGAGATCCCAGTGAAACTAAAGTTCAAGGGAGCAAGAGTTGCGATAGGGTAATTG

TTAAAAGATCTAGAATTTTAAGCATCAGCGAAAATAGGGAGGGAGTTATGGTTTCTAATG
TGGAGGGTTCTCTGGGCTTGAAGAGCTGCTCACAGTCTTCAGCTGAGATTCATTCTGATA
ATGAAACTGGCAGCACTTTAACAGGTGCTTTTGATGCAATGAGATCTGGAAAAGTGAATG
ATCAAACTCAGAATGACAAGACTATGGATCCAGCTGTTGCCTCGGAATTTGCTGCGAGGG
GAGATTTTATGAGTTTTAGCAAGTCTTTGGATGCTGGATCTGATGAGTTGTCTGGTTCTG
CTAGATCTCAGTTGTTTAGTGAAGAGTATGAAGGATCATTTCTTGGCACCAAAGCTGCAA
CACGTTCACAAGATCCTATTTTAGGTGTTGAAGAAGAGATGTTTAGTGCAGTGGAAATTG
GGAAAAGTATGATTGATCACAATCTCCATGATGATGTTACTGAATTGGGATGTAATGATG
TTCAAGGGAATTACTTTTTGAAGGTTGATCCAATTCCCATACCAGGACCGCCTGGATCTT
TTTTACCTAGTCCTGGTCGTATGAGCTCAGAAGACTTTCATGGGAGTTCATCATTAACCA
GCAGTAAAATTCAGTCTTCTGCAGATTATCCTGAATTCTTTGATCAAGATTCTTCTGGTT
CCCCTACTTCTGCTGCATCAACCGTTTCTAACTCCACTATGGCTAGAACTGGCTCAAGAT
ATTCGGACAAGTTGTCTGGTGATGGAAGGGATTCTTCTGAAAGTCTCAGATGTCACACAT
CTGGCTGGGAAGATAAAAGGTCTAGCATTTCTGGCAGCAGTACTGTTGATCTTCTGGTGG
AAAATTCTGTCACTCGACTTCAGACAGAAAATACAGGAGACGATAGGGATGGACTGGACA
AATTTAAGGCAAATACATTCTTTCCTGGAAAAGGAGCTTTCAGATTTACAAATGATAAGC
CATGTTGTTGTGTCAGGAAAGAAGGAGCATCTCAAGAGTCACAGCTCTTGCAGCGAAGAG
CCACGGCACCTTCCCCTTTTCCTGCCAGTGAGGAGCAGTTGAGGTGTGATTCAATCAGAA
GACCTAATAATATCAGTAACTCATTTTCTCTTAGTGACTCGAGTTCAGGACCTGAAACAA
ATGCCACTAAATCATCCACTGGATATACTCAATTTGGAGTTTCTGCTGATTCTGACTTCA
AGCTCCGAACTCGTGATTCCGAGTCTTTTAGTCCATCTGCTTCCAATCCAGTTCTCAGGC
TGATGGGAAAGGACTTAATGGTGGTCAACAAAGATGAAGATTCCCCATTGAAAAGATCAT
CTCACTCAAATTCCATGAATGACCTGGTAAACACTAGACTTGCTGGTGTTTCTTGTGGCA
GTCTTCATAGTGAGGATCCCTACTCCTCTCGTCAGGTGGATGCACATAATAGTCTTGTAT
CTCACTTGCCTCAAACTGGTGATACAGTTCAGCATTTTGATGTCAGATTGTTAAATGGTT
TCAAAAGTCGCGACAGTTACTCAAGGCCACAACAGCTGTCCCCTACATCTCCAGTCTCGA
TTTTGTGCAAGAGTAGTGATGGCGGATTGATGGATCCCGTTGGCAGACAAGACTATTTAG
GAGGGTGCACTTTACATACTGTGCTCAATGGACCAAATAAGACATGTGATGGAAAAAAGT
TCGTGGCAACTCCCATATCTCATTGGCAAAATTCTACTTCAGTTGGGAATGCCGTCAAAG
AAATTATTATAATTGATGATTCTCCAGTAAACGGGGCTGATTCTTCATGCACCATGGGTA
CAGGGAGAAGCAAGTCGTCAACTGGTATCCAAATGCAGATGATCAGCTCGGGCTATACCT
CAAAGTTTGTGAATTTCTGCGAAAATAGACCGTGTTCTCCTTACAGCGGATCTGGAGTAG
CTCAGAATGCGAATTTGCCTACTCAAAGGAACGAGATTCCTGCTAAGTGGAATGGCAATC
TGGAAGGTTGTAGTTTTGTTCGTCCAAGCTCTTTCTCGGCTTCCTCATCACCTGCAGGTC
CTTTTAGATCATCATCTTTCTATTATTCTGCCGGCTTTTCATAACCGAGAATGTTGCCTT
GCATGGGCATTTACTTTCTTGGCAGACAATAGAAACCTGACGCTTACAAAGCATAATATA
GCAGTCTGACCGAAAACACACACGGCAAGTTTGAGCAGATGAGTTATTCTAGATTTGCAG
GTTTTGCTTTATAGCTACTTGAAAAACTGAAAGTTCCATAGAGATAAAACGCATTTTACT
GAGGCCAGTTCGAAAGTTCCCTGGAGTCCTACTATGTATAAAAGTCTTTGAATAATGAGT
TGTTCCGAGGATATGAACTTCCAAAGTGGTTTCTTCTTAATATCAATATCTTTCTTTTCC
CCATTTAAAGGAAAAAAGGCAACTCATACACGGTACTTTATGTCTCTTTCTATTTTTTCT
GGGATTGTAATGTACAAATACCCTAATATTAGGTAGTATTACTTGTTACTTGTTATTATA
TGATAAGTATTTAGATCCATACAGAGAAAAGGATTCCAAATCCTAATTCACGCATATATT
AGGGTTTCTCTAATATTACTGCATATATCTACGTCAATTTTCTTCTATACTTTTCTCACT
AATTACTTTGTAAACCTAGCTATCTTTTTCATGTAGGATTTATGTTGCAAACTTTTATCG
ATCACGGAGTTGAAAGAAATGATGATGTTTAAGCTTGTGGTCTTTCATGTTTTATTATTT

>comp60101_c0_seq1 5351_5605
AACCAGATAATTATCCCAACAGATATTTGGCTATTTTGTGGAGCAAGTTCCCAAATGGGC
AATTTTCATGCAAGGATGGATAAGGTATTTTGACACTCATACTCAAGCCACAGGACACCC
ATCTAAAACCCAACGACACAAGCCCCTTAATTTCCGGCAGGTGGCAATCTCTCCTTCTAC
GCTACCGCCGGAATCCGCCGTCTTCCTACTCAAATTACTAAAACACCCTTAAAAAACAAA
CTTACTAAAACAGCCCTGCAACTGCAATGCAAAATCCACTATTTAAACCACTTCCACTCT
CGACACCTCAATCATATTCACACTCATTACTTTCTCCTTCTTCCCCCATCATTGTGCCCA
AAAGGCTGAGTGATATTAGGGTTAATAATAGTAGTAGATTAATTAGGGCCATGCCTTGTA
CTTCGCTAGTGAAACCAAGTGACGGTAACGGAGCTGTGCGTAGTTTCAAGCTTAAGGAAT
CCACTTTCCTTGCTGCTCAAATGCCGAAGAAAGAGATTGCCGCTGATCGATTCATCGAAG
CTCACCCTGAGTACGACGGTCGCGGCGTCGTCATTGCCATTTTCGATTCTGGTGTTGATC

CCGCTGCGGCTGGATTACGAGTTACTTCTGATGGAAAGCCGAAGGTCATAGATGTTATAG
ATTGCACGGGGAGCGGAGATGTTGATATGTCTACGGTGGTGAAGGCTGATAATGATGGTT
GTATTCGTGGGGCTTCTGGGGCTTCTCTTTTCATAAATTCTTTGTGGAAGAATCCATCTG
GTGATTGGCATGTTGGCTGCAAATTGGTGTATGAGCTCTTTACTGATACACTGACTTCTC
GAGTAAAGAAAGAACGGAAGAAAAGGTGGGATGAGAAAAATCAGGAAGCAATAGCAGAGG
CTGTAAAGCAACTTGATGAATTTGATAAGAAACACACAAAAGTCGAAGGTGTGCACTTGA
AAAGGATCCGTGAAGATCTCCAGAACAGAGTTGATCTCCTGCGCAAGCAAGCTGATAGCT
ATGATGATAAGGGGCCAGTTGTTGATGCTGTTGTATGGCACGACGGAGAACTTTGGAGAG
CTGCTTTGGACACACAGAGTCTTGAGAATGATCCAGGATGTGGGAAGCTTGCGGATTTTG
TTCCTTTAACTAATTACAGGCTTGAACAAAAACATGGAGTTTTTAGTAAATTGGATGCCT
GTACGTGTGTGCTAAATGTCTACAATGGAGGAAACATTTTAAGTATTGTAACAGATAGCT
CACCACATGCCACGCATGTTGCTGGTATTGCCGCAGCCTTCCACCCAGAGGAACCTTTGC
TAAATGGAGTTGCACCTGGAGCACAAATTGTGTCTTGTAAAATTGGAGATTCACGGTTGG
GATCTATGGAAACAGGAACTGGTTTGACTAGGGCCTTGATTGCAGCTGTAGAGCATAAAT
GTGATGTCATCAATATGAGTTATGGAGAACCTACACTGCTTCCAGATTATGGCCGCTTTG
TTGACCTTGTCAATGAGGTGGTCAACAAACATCGTCTAATATTTGTCAGCAGTGCCGGCA
ATAGCGGCCCGGCTTTGACTACTGTGGGTGCACCTGGTGGCACTTCGTCAAGCATAATTG
GAGTGGGAGCATATGTCTCCCCAGCTATGGCTGCTGGTACTCACTTATTAGTTGAGCCAC
CTACTGAAGGCCTTGAATACACTTGGTCTAGTCGGGGACCTACTGTGGATGGTGATCTTG
GTGTCTGCATAAGTGCCCCTGGTGGTGCTGTGGCTCCTGTTCCCACATGGACCCTTCAAA
GACGCATGTTAATGAATGGAACATCAATGGCATCTCCATGTGCCTGTGGCGGGGTGGCTT
TACTTGTCAGCGCTATGAAGGCAGAAGGTATTCCTGTTAGTCCATACAGTGTACGGAAGG
CTCTTGAGAATACATCTGTACCCGTGAGTGCTTTACTAGAAGAAAAATTATCCGCTGGAC
AGGGACTCATGCAAGTTGACAAGGCCTATGAGTACATCCAAAAAGTGCAAAATGTTCCAT
GTGTATGGTATCAAGTAAAGATCAAGCAGGCTGGCAAAACAAGTAAACCAACTCTGTCTC
TTGCATCACGAGGCATCTACCTTAGGGATCCTAATTATTGTCACCAGTCTACGGAGTGGA
CAGTGGAAGTTGAACCGAAGTTCCATGAAGACGCAAATAATTTAGATCAATTGGTTCCTT
TTGAAGAGTGCATTAAGTTGTTTTCTACTGGAGATGCGGTAGTGAAAGCTCCTGAGTACC
TCCTTCTCACCCATAATGGACGCAGCTTCAGCATAGTGGTGGACCCCACCAATCTAAGTG
ATGGCTTGCATTATTATGAAGTATGTGGCATAGATAGCAAATCTCCATGGCGTGGACCGC
TTTTCAGAATTCCAGTTACTATAACAAAGCCGACTGCTGTAAAGATCCGACCCCCTCTAA
TTTCCTTCCAAGGCATTTCATTCGTACCAGGCCAAATTGAAAGGAGATTTATTGAGGTAC
CATTTGGTGCTACCTGGGTTGAGGCCACCATGAGGACATATGGATTTGATACAGCAAGAA
GATTCTTCATAGACACTGTTCAGCTATCCCCATTGCAAAGGCCCATTAAATGGGAAAGCG
TGGCCACATTTTCATCACCTTCATCCAAAAGCTTTGCCTTTCGAGTTGAGGGTGGTCGGA
CAATGGAACTAGCAGTTGCCCAATTTTGGTCCAGCGGCATAGGCAGTCATGAAACAACAA
TTGTCGATTTTGAGATCGCGTTCCGTGGTATAAACATCAGCAAAGAGGAAGTAATTCTTG
ATGGAAGTGAAGCACCCGTAAGGATTGATGCTGAAGCTCTTCTGGCAGCTGAAAGACTTG
TCCCTTCTGCAGTGCTCGACAAGATAAGAGTTCCATATCGTCCGATTGATGCCAAACTGC
ATGCACTGTCAGCAGATCGGGACAAATTACCTTCAGGAAAACAAATTCTGGCTCTTACAT
TAATTTACAAGCTCAAACTGGAGGATGGTGCAGAACTAAAGCCGCAGATTCCCTTACTTA
ACAACCGGATCTATGATAACAAGTTTGAGTCTCAGTTTTATATGATATCGGATGTGAATA
AGCGAGTGCATGCTATGGGTGATGTTTATCCAGATTTCGCAAAGCTTCCAAAGGGTGAAT
ACACTATACAGCTATATTTGAGGCATGACAATGTACAGTATCTAGAGAAGATGAAGCAAT
TGGTGTTATTCACTGAAAGAAAATTGGAGGAAAAGGAGATTGTTCGGTTGAACTTCTATT
CTCAACCTGATGGCCCACTCACGGGTGATGGTTCCTTCAAGTCTTCAGATCTAGTTCCAG
GTGAGAAAGAAGCATTTTATGTGGGTCCTCCGGTGAAGGACAAGCTCCCAAAGAATTCCC
CTGAAGGATCTGTATTGTTTGGCCGAATTTCATATGGAAAGCTAGTGTATAAAGATAGTG
AAGAGGGAAAAAATCCTGCATCGTATCAAATATCTTATCTTGTGCCACCAATTAAGCTCG
ATGAAAACAAAGGAAAAAGTTCGACTGACCCCAAGACTGTCTCAGAAAGACTAGAAGAAC
AGGTCCGCGATGCTAAAATAAAGGTTCTTGCTAGCTTGAATCAAGACTCTGATGAGGAGC
GTGCAGAATGGAAGAAGTTATCTCTGTTGCTTAAGTCAGAGTACCCAAAATACACTACTT
TGCTTGCTAAAATATTGGAAGGATTGCTTTCTCGGAACAATGTTGAAGACAAAATCCATC
ATTACACAGAGATTATCAGTGCAGCAGACGAGGTTGTGACTAGCATTGATAGAGATGAAT
TGGCCAAGTACTGTGCCCTCAACAGTGATCCAGAAGACGAGGCTGCAGAAAAAATGAAGA
AGAAGATGGAGACAACCCGTGAACAGTTAACTGAAGCAATCTACCAGAAGGGATTGGCTT
TGGCAGAGATTGAAGCATTGAAGGGTGACAACAATGTGGATAAGGCAGATAGCCAGGCTG
CATCAGAATCTGATGTTACTTCCGATATGTTTGAAGAGAACTTCAAAGAGCTAAAGAAT

GGGTGGATGTGAAGTCTTCTAAATATGGTGTCCTTTCTGTTTACCGTGAGAGGCATCATG
GAAGACTTGGAACTGCACTTAAGGTTTTAATCGACATGATACAAGATGATGCTGATCCAC
CTAAGAAGAAGTTTTATGAACTAAAGCTCTCTCTGCTCGACCAGATAGGATGGAGCCACT
TGGTAGTGTATGAGAAACAGTGGATGCAAGTACGATTTCCATCGTCTCTTCCACTTTTTT
AGTTCTTGTTCTGTGTAACACATTCATATTGAATAGAATAGGGAGACGAGAGATGTGAAC
GGAGTTCTGCATGAAAAACAGGAATATGCACGGTCGAATAAAATCTCTTTTTCCTCCCCA
GTTCAATACTACCTTCAACGAAAGTCTAAACCAAGTACTGTAAAATCTAAATTGTGTATC
GCGGCGCTCTGACAACACGCGGCCTCTATCAGGAATAACATCTATTGCATATCTGCATTC
TTGGCAATTCTGCGGATCCTATATATTTGAATAGTACGGCAGGAATGTCGAGAGGAGTGT
ATGGCTGGAAGTGGCTAACAGAAGTGGAAGATGATGTGAGGATTTGAACCGTTAACTTGG
CATATTTTATTGAATTGATTTTGCAAGTTCCTTTCGCTTTATGGTTCTCGTACATTCAAC
AACATTCTCGAATACGGGTATATAATCGGATTTCTAGATACCATTTATTTGGTCTGAAGA
TAGGCCTTAAATGCTAGTGCTACTCATGTTTTGGGAGCCGTATGGTGCTGCTCGTTCAGT
TGAAGTCTCCCTACCTGACCTTGTATCGTTTTTCTATTTGATTGTGAGGGAATACACTAT
TAGGATTGGCAGAACAAAGGGGTCTTAATTTCGGGCAAGAGGGAAAGCGAAACTTGTAAT
TCCTCGTTAACTCAAGGCTGGTAAGTTCGTTGTTTCTGCTGGAGTTCGCCTTGTCCCGTG
TTCATTGCGCAAGTTTCATGTAAATCATTTGATTGCAGCTTAATATTCATCTTTTGTGGC
AATATGATATCACCTGCAATGAGCAGGCAATAACTTTAGATCCGAGTTTCGACATTATAA
GAAGAGAGGTTTACAATTAATAAGACATTCCTCAATCTTCCTTGTGATAAAAAAACGAAG
AGGAAAAGGTAGAGGAAAAAAGAAAAATTCAGACTGAGGTGGCTGCCTATCTGACCGTAA
GGAGGTGTACAAATGTCGATAGGTTGGTTGCCTTTTGCTGTAAATTTGAATATGAAATTA
TTCTGGAAATCTAAAGGGGTTGGAGTGTAAGAGGCAGTCCTTGTTTGGGCCCAAGAGAAA
GCATAATAGAAGGGCAGTATCTATAGTTAGGTGAAATGGAAAAGATCTTTCTCAAGATTA
GAGACGAGACAATTTTGTACTCCATCATTGTCAAATTCCTACCAATGCACATTCTCCCTC
CAAATCCAAAGGGTAAATATCCCATTTTGTGTTTACATCCGCCATTTATCATGTCTATCC
TTAAACCTTTCTGATTTGAATTCATTTACATCTTCTCCCCGAAGTTGCTTGTCGTGATGC
ATGG

>comp75863_c0_seq1 200_1609
AAAACTTCCAAGTCATTTTACACTTTATTTTTTCGTTCTTTCTTTCACTATCACTCCAAC
ATTATAAAACACAAATCAGTGTAAGAAAATTATTTTTAAATTCCCTAAAGCTCCACCAAA
CACTCTCTCCCTCCAAATAAAACCTCTAATTTTTTCAAACCCCCAAAAAGAAAGAGAGAA
GAGCCTAACTAAGGCTTCAATGGCGACTATGGCTTCTCTTTTTCTCAAAACCCCAGGTCC
ATCTAAGTCTTTATCCAAAACCCAGAAAACCCATTTCGTGTTACCCCTCAATCTCCCTGT
TTCGTATTCGTGGCGTTCCAAGTTCCGTGCCGTACCGGGGGCCCGAATCCGATGCGGGCC
GCTGATTGAGCCCGACGGCGGAAAGCTCGTGGACCTCATCGTCGAGAAGCCACTGAGAGA
TTTGAAGAGGAGACAGGCTTTGTCTCTTCCGCAAATCAAGCTGTCAAAGATTGATATCCA
ATGGGTTCATGTGCTCAGCGAGGGCTGGGCCAGCCCGTTGAAAGGATTCATGAGAGAATC
CGAGTTCCTCCAAACTCTTCATTTTAACTCGCTCCGACTCGGTGACGGCGGCTCAGTCGT
CAACATGTCGGTGCCGATTGTGTTAGCCATCGACGATTCTCATAAGAATAGGATCGGTGG
GTCTACCAGTGTTGCCCTTGTTGATGACAAGGACAGGCTTATTGCCATTCTTAACGATGT
TGAGATCTACAAGCATAATAAAGAAGAACGGATAGCCAGAACTTGGGGAACAACTGCCCC
AGGTCTACCTTACGCCGATGAAGCAATAACTCATGCTGGAAACTGGCTAATTGGTGGTGA
TTTGGAAGTTATAGAACCAATCAAGTACGATGATGGTCTTGACAGGTTCCGGCTTTCCCC
TGCTGAACTTCGAGATGAGTTTACGAGGCGCAATGCTGATGCAGTATTTGCTTTTCAACT
CAGAAATCCAGTGCATAATGGCCACGCATTATTGATGACAGACACACGTCGTCGACTTCT
TGAGATGGGATACAAGAATCCTGTCCTTTTGCTTCATCCTCTGGGAGGTTACACAAAGGC
AGATGATGTTCCACTTCAGTGGCGAATGAGGCAACATGAGAAGGTACTTGAAGATGGGGT
GCTTGACCCAGAGACTACTGTGGTCTCTATATTCCCATCTCCCATGCACTATGCTGGCCC
AACTGAGGTGCAATGGCACGCAAAGGCACGCATCAATGCAGGAGCTAACTTTTACATTGT
GGGCCGGGATCCAGCTGGAATGGGCCATCCATTGGAGAAGAGAGATCTGTATGATGCAGA
TCATGGAAAGAAGGTACTCAGTATGGCCCCCGGGTTAGAGCGGCTGAATATCTTGCCTTT
CAAGGTGGCTGCATACGATAAGAATCAGAATAGAATGGCATTCTTTGATCCCTCTAGGCC
TCAAGACTTTCTCTTCATATCAGGCACCAAGATGCGATCACTTGCAAAGAATAAAGAGAG
CCCTCCAGATGGTTTTATGTGCCCTGGTGGTTGGAAGGTCTTGGTGGAATACTATGATAG
TTTGACTCCAGCCGGGAACGGCAGAATTCCTGAACCTGTCCCAGTTTGAAAGTATCATCA
TTATCAAGGTGTACCCCAATGTGTTTGGGAGAAACGTTAAAATGTAATATGGCTCCTGAT
TTGGTACACATTCCTCTCAAAGGACAAAACAATAATAATAGTGAGGCACTGGTATTGTGT

GAGAAAAGGTGTCTCCATCTTGTAAATTATTAGTGCCAAACATGGCACCTTAACTTTGTC
GGCACATCTGTTTGCAGTTGTATCATAGGTTATGCTGTCATGAATATGGATTTCAGATTT
TCTGTTGTTATGTCAGAGTATTGATTCAAGAAAATGAATTATGTAGCAGGCCTGCTTTCA
ATTCTTTCATTAGAAGGATAAGCCAATTTAGCTCGACCTTGCCTATTAAATTCTACTCTA
CGCCAATGTTTACCAAGTTAGTTTAGTTGGCTAATCACGCTGGAGATTAAATTCAAAACT
GAAGTCTAACTGGGTTCCTCTATATTCTATAATGACGTAAGTTTCAAACTGGGTGCCACC
GGCGCCCTGCTTGAATTCTCACTTATTAAAGATGGGTTTGCCCGCGATAAGATTTCCA

>comp29318_c1_seq1 108_620
AAGAAGCCTTTTAAATAGTTGAAGATGTGCTTTTGAACAACTTCAGGTCTGTCAAGGGCT
TTCAATTAACTTTGAGGCCTTAGCTTCTAAGCTTCACCTTCAAGAAAATGGCTATAACAA
GAGAAGGTGAGATAGAGTCCCCACGCTTTGCCCCTGAGGCATCAAACAACAAAGTTCTCC
CCCAAACAGTTGAAGAAACAACTCCTCCAACTTTGGCAGAGAGTCAGAATAATACTTGTC
ATCAGTTCTCCCAAACACTTCAAGTAGAGAAACGATTGTCTCTGTTCCCCCAAATACCTA
TGGCTTCTGAGAATGAGCAAACGCGAACGCGTAATTGGGTACTAAATGTACCAGAACCAC
CAAGCCTATTATCCAAGCTGAAGAGTTCATTCAAGAAAACCATATTCTCTCCTCTTGAAA
ACAAGCTCCACTGCGTTGGAAAGCAGCCTGTTCGTACACTCATTTCGATTCTCAGGACCA
TATTTCCTCCACTTACTTGGTGTAAAGAATACAATGTAACAAAATTCAAAAGTGATATCT
TGGCTGGTTTAACTCTTGCAAGCCTTTGCATCCCTCAGAGCATTGGCTACGCGACTCTTG
CAAAACTTGATCCTCAATA

>comp71133_c0_seq1 1089_1355
TAAGCCCTCAACTCCAAGCCTCACCTTCCATTTTTCCTTTCTCCATTTTCCACAGAAAAA
TACAAATGGAAAACAACTCCATTCTCTAAAGCAACTCACTGTTTGGATTCTCCTCTTTCT
CTTCTTTACTATTTTCTGTAGAAATGGCGGCAGTTACTTCAGTGTCGTTCTCGGCGATTG
TTCAATCCGCCGAAAGGAAATCCTCCGTTTCTTCGTCTCGTTCTGTTGATACATTTAGGT
TCCGTTCCAACGTCTCCTTTGATTGTTTCAATGTTCGATCTTTGAATTCCAGTTTCAGTT
CGAATTCTTCCACCTCTCGCTTTGTCGTTCATTGCATGTCCACAGATTTGCCAACTGTGG
CCGAAACTAAAATGAATTTCTTGAAGGCTTATAAGCGTCCAATTCCAACTGTCTACAACA
CAGTGTTACAAGAGCTAATTGTGCAGCAACATTTGATAAAGTACAAGAAATCCTACCGAT
ATGATCCCGTGTTTGCGCTTGGTTTCGTCACTGTATATGATCAACTTATGGAAGGCTACC
CAAGTGAAGAGGATCGTGATGCCATCTTCAAAGCATATATAGAGGCGCTCAATGAGGATC
CTGTGCAATACAGAGCTGATGCACAAAAAATTTGAAGAATGGGCTCGTACTCAAAATGCCA
ATACGCTAATTGACTTCTCATCCAGAGATGGAGAAGTTGAAAACATTCTGAAAGATATTG
CACAGCGAGCTGGAACCAAGGATAGTTTTTGCTACAGTCGACTATTTGCGGTTGGTCTTT
TTCGCCTACTTGAGTTGGCAAATGTAACTGATCCGACCATCTTAGAAAAGCTTTGTGCTT
CACTGAATATAAACAAGAAAAGTGTGGATAGGGACCTTGATGTTTATCGCAATTTGCTCT
CCAAGCTGGTTCAGGCTAAAGAGCTGTTGAAGGAATATGTGGAAAGGGAGAAGAAAAAAA
GAGGAGAAAGGGAATCTCAGAAGGCCAACGAGGCTGTCACAAAATGCTTGGGAGATTACC
AATATGCTGGGAGGTAAGTCAAAGATCATTTCGTCCTCCTGCTTGGACCTCATTGACTTG
TTTCCCTAATGAAACAGTCCATGTATGATAGAGTTCAGTCTCCAAAGCTCAGGCAAATTA
TATTAGAAAATCGAAGTTATGTTCAATGTTTTGTTATTACAGCACTTGAGGTATATTCCA
ATGTTCATTATTTTCTGGTTAAACAGAATAGCTTTATTGTTGCAACTACCTCTATTATAT
TTGTAGATAAAACCTCAAGTAGTGACAAAAATCAAGATTTGCTCAGCAGTCATGCTTCTC
ATTTTCCATGGTTCTGCCTTTGTTTCAAGTTGTAAACAGTGTCTTTACAACTATCTATAC
GTTTTAGTTGGCAAACTAATGTTCATTGATTATCAGAGTACTGATAAAATCAGAATAACT
CTGCTGTAACAATGTACTACGTAGATATACCTCTTCAAGGGGGTGAGTATTCATACTTGC
AGAAAATTTGTGAAGGTAAAATAGTAATTATATCTGCCACCTTATCCGAGATTAGGGCCT
TTACGTAGAGAACTTAGTCAG

>comp77639_c0_seq4 942_2765
CAATTAAAAAGTGACAGCTAGCCCAACCTAACTACCTAAAAAATTAGTCAAATTTTAAGA
GGTTTTGGGTTTGGCATTGCGCAGTAATCCATTTAGTCAGATCCCAAAACTTACATTCTT
CTTCACCTGAAATTGCAACACAGCAGCATCAATGGAAGCTGCTAGGGTTTAGAATTCTCA
ATTGCTTTTTTCCTCCGCCGCAGGTGCCGCCAATAATGGAGGAGTTGGCTTTTTTACTTT
CTGTTATTGCTAATTTCTAAAAGTCTAAAGCTTTAAAGCTTTAATATCTTTTCCTACTAA
TTGAGAGAAACTTTTCAGTTAAAAAAGAAATATTTTCTTTCATTCTCACAAATTTGGAAA
TATCGACAATCCTCAATATACTTCATACTGTAGTACTTATTTTCCCCCAAAAAACAATAT

TAGAATAAACTCTCAGGCATCAATAGAATCTTTCATCGTATCGTAATCTTCATTGTAGAA
ATCAAGTTTTGGTTTTTTTGGGGCGTTGATGTTTTCCTTCTTCAACATCTTTTGGCGTAG
GCCACACAAAATAATTTAATCTGAAAAGGAAAAAAAATGCTAATGAAGTTCTCACATGCT
AAATTTGTGGTGAGACACAACTGATTTGCTATGTTAGCGTCGGGTGTGTACTTGATATAC
TTAATAACCACTTGAAGAGTCAAAATGGTAAAGGGGTCAATTGAAGTATTTGTTTGATCG
TTGAAGATAACTTAAAGAGGGTCGTTTATGTATTTTGCCTTTAAAAAAACAAAAAACAAA
AAACAAAAAACCCAACTCTTTTAAAATGGTCCTTGCTTCGCCTACAGCAAAAATCAAAAC
TCCTTCTGTCTCTTTCTTGCTCTCAAACTGACCCCCCTCAAATGCTCCATTTTTTAGCTA
TACAACTTCTTGTTAAAAGAGTGAACTTTAGTTTTTGTTAGATGGATTTTGGGGTGGTGG
GGAGTTTAGATAATGGTGGGGTGATTTGTTCAGACAATACAAGTAGTAATATGTTTACTG
CAGCTGCTGCCTCTGTTGAGACCAAGCAAAAGTGGTACGGATCTACTGGATTTCACAAGA
ATCAAAGATTTTCTACTACTAATACTGCAACTGAAGATGACTGGAATAATAAGGACACTA
AATTAGCTAAGACTAGTGATGATTTCTCTTCCTCAAAATCAGCAGCAGCAGCAGCAGCTA
CAATGCTGTTTCAGCAACAACTTAGAAGTAATTGTCCTAACAGCCAGCAAATGCTTAGCT
TTTCTTCATCCAACTCTCAGAATGTGACAATGCCCTTTCATCATTACACTTCAACTCCTT
TTACTAGGAACTCTTCAGGCTGTGGCAGTGGAGGTATGAATGCTGGAAACATGCATGGGG
TAATAGCAGGGGTAAAAGGGCCATTTACTCCATCACAGTGGATGGAGTTGGAACACCAGG
CTTTGATCTACAAGTATATCACTGCAAATGTTCCAATACCTCCTTATCTCCTCAGCCCCA
TTAGAAAAGCACTTGAATCTGCTGGCTTCACAACTTTTGCAGGCCTTAGACCCAATGCTT
TGGGATGGGGTGCATTCCATATAGGATTTTCCAACAGCAACGATCCGGAGCCAGGAAGGT
GTAGGAGAACGGATGGGAAGAAATGGCGGTGCTCAAGAGATGCAGTTGCCGATCAAAAGT
ACTGTGAACGGCACATGAACAGAGGCCGCCATCGTTCAAGAAAGCCTGTGGAAGGACAAA
CTGGTGCTACTTCCGCCAAGCTGATGAATGTATCTTCTTCAGCATCAGCATCAACATCAG
CAGCATTAAATCCCGGAGGCGCTGCATCCAATACTCTACCTCTCTCACATCCCCAACTTA
GCAACTTGCCGCTTGCTGTTACTAACCCCTCCACTACTTCCCCTTATCTTGAGAGGAATT
ATACGCCCAAGGAAAATTTTGGTGAAAGGTATTCAGATACGGAAGGCCTTACAGTTGTTT
CATCAAGTGAGAAAGTTGTTTCATCAAGTGAGAAAGAGAGCCAATATTCAATTCAGAAAG
AGCAGAATCTTTATGGAGTAACTTCAAGGGCTGAATTTGGATTAGTTTGTGCTGACTCTT
TGATCAATCCACTAAATAAAAACTCTTCACTAGTGAATTGCCGTGGCTATGGCAATTCCG
AAGAAAGCAAATCGCAGCATCATCCACTTCGACTATTCATGGACGATTGGCCCAAAAACC
AGTCTGATCAATCCACAGTTTCTTGGCCAGACATTGATTTGCAGTCAGATAGGACTCAGC
TATCCATTTCCATACCCGTGGTCACTTCAGACTTCATGTCTTCCACTTCTTCTCCTTCGA
ACGACAAACTCAGGACCTCACCACAGTACAGACCGTCACAGGAGCACGAGACAACAACTC
AAATGGGATTAGGCATTGGCACCATCGTCAGTGATCATAACCAAAGACAAGGAAATTGGA
TTCCTATAACTTGGGAAACTTCCATGGGTGGTCCCCTCGGTGAGGTCTTGCATAGCACTA
ATAACAGCTCGGGCGATTGCAAGAACAAATCAGCAGCTCTTAATCTCATGACCGAGGGGT
GGGATAGGAGCCCCCGGATGGGATCATCACCAACTGGGGTTTTACAAAAGTCCTCGTTCG
GTTCCCTTTCTAACAGCAGTGCAGGGAGCAGTCCAAGAGCCGAAAACAGTAAGACAAACG
AAGGTTCCAGTCTTTGCAATGGCCTTGGAACAACTCTTATGAATCCAATACTGCCTGCCA
TGTAATATGTAAAACTAGGATATGCTATTTTGTAATTCCAGAAGTGAGCTATTTCCCTTT
TTCCCCCTAATGCTCGCATCCTTTGCCAATCAATCTTGTACTGAAGATATCAAAATGACT
ATGCATTCCTGTTAAGATTTGTTCATTTATTTGCTACTGTAATATTTTTCAGCTTTTTGG
GCAAAAAAAAAAAA

>comp67678_c0_seq1 205_573
ATAAAGTTTTCATACCATGACGCGTAAGGTTAATTCGCAATAAAGCGTAAAATCAGTTAC
ATTTTGGTAATTTAACACCTCCCTGTCCCTATATAAAGTCTCTTCTTGGGAATACACTCT
CATTCAGCGTTAGTTTTACGAGGAGCTAAAGTTAAGGGCTGGTTGGTTGTTTGAAATTTT
TTGTTTCTAAGAAATTTGTGAGAAATGGCTGCAGCTTCTGAGGAGGGGCAAGTGTTCGGC
TGCCACAAGATTGAGGAATGGAATGAGCAGTTCCAGAAAGGTGTGGAGACTCAGAAATTG
GTGGTGGTGGATTTTACTGCTTCCTGGTGTGGCCCTTGCCGTTTTATTGCCCCAGTTCTT
GCTGACATTGCTAAGAAGATGCCCCATGTTATATTCCTCAAGGTTGATGTTGATGAACTG
AAGACTGTTGCTGAGGAATGGAGTGTGGAGGCAATGCCGACATTTGTCTTCCTTAAAGAT
GGGAAGGAAGTGGACAGGGTTGTTGGTGCCAAGAAAGAGGAGTTGCAGCAGACCATAGTG
AAGCATGCTGCTCCTGCTACTGTGACAGCATGAAAATCTTCTTAATCAAGGGGACAATAT
TCCCTATTTAGTATTGTCTTTTGTAATAACCAAATAACTTGTTCGAACTTCACACTGTGG
ATGGTTGTAAGTTTTATTATCCACCATGTTTTTATTGCTTATGTGAACCTTTGTCTTCGT
GCTTGGAATCTGATTTGTGCATATGGTGTTATTGGTGCAAGGTTATATGCTCAACTCTAC

AGAAAGACTATTTTCAGACTTCTACTTTTGTGAGGTTTTCATTTTGCTTGGATAGCACTA
GACTTATTTACAGTTTGCTTTGTTCTTGAGGTTCTCTTGGTACGTTATCCGGTATCTCGG
AGTATCTTTTGAGTATGCGTTTGGACGATATAGCATGGTTGAAATTGGAGAAAAGGGTAT
TTGAAGTTGTAAGTCGAAAAGTATATTTGGATTGTAAGTTGAAGTTGTGTTTGAGCAAGA
ATTTTCATTGGAAAAATAGTTCGAAGATTTGTGCTTGAAATTTTCTATTTCACTTAAAAT
ATTCAAATATTTTTCTACTATTTCAAATGCGTGTGCTTGTGTCTTTTATTGTTATCTTTT
TTTGTTTTGGTGGTGTCTTTTGTTTGTGAATTGTGACAAAGACAAACAAGCAACATTTCT
TGGCTTGGTAGTCTTGTGAAATTTGTTTAGTTCGAGTGAGCTGAATGCACACTAGATCAT
TTTTGATGGCAATAGATCCAGATTCAGTTTAAGTTGGCCAAAGTGACTAATTTCCCTTTC
GATTCCCTCTTGCTCATTTATAAATGATACGAGACATGACTTGAAATCTTATGTCTAGGG
ACTTGCTCTTATTTTCCTTGTGTCGGTCATTGGTTACTCAAAGAAAGAATGTGAGACACC
ATACTGTGGATGGAAACAGAGAGCGTCAAAACTCAAATATATACATAAATCACGCAAACT
AGAAAAACATAAGACTGGGACAAGGAAAGATGGAACAGGATATGCAGGGAAAGTAGCCAA
AATATTGAAGAAAAAGGCAGTAGTTGCAGGCTGGGGGCTGCACGACGCATCATTTAATGC
AAAATTCAATGAGAATATTGCGTCTTTCGAGCAGGAGAGTGTTTTTCGAGTTCGAGCAAA
TGCAATGCGACATCCAGGCAAAAAAGGGCTCGGGTCCGATCTGCGCATACGTCACTAAAG
AAGATAAGAATCCACATATATGGGGAACACGTCAAAGACCCATGTTATGGAAGTAGCAGA
GGCTTATGAGGGAAGGAGAAGCACACACCTCAATAACCCTGTCTTTGCGAATTGAATGAA
ATTAAAAGTACAGCCAGTTTAAGCTGGTTTTCCACCTATTTTTAGTGTTAGGCCATTTCT
AGCAAGGTCTAAAGGACATGAAGATAGGTCGCCTTTAGTCTTTTGATTATAGCAGGGGAC
AGCGTACCGCCCTGCTATAA

>comp50987_c1_seq1 2_379
GTTTAAGAAGTTTAGCTACAGAGGCGTTGATCTCGATGCTCTTCTCGATATGTCCACTGA
TGAGCTCGTGAAGCTCTTCAATGCTCGTCCTCGTAGAAGGTTTCAGAGAGGTTTGAAGAG
GAAGCCGATGGCACTGATCAAGAAGCTGCGGAAGGCTAAACGCGAGGCTCCACCAGGTGA
AAAGCCAGAGCCTGTCAAGACTCACCTGAGGAACATGATTATCGTTCCTGAAATGATTGG
AAGTGTTATTGGAATTTATAATGGAAAGACATTCAATCAGATTGAAGTCAAGCCTGAGAT
GATTGGCCACTATTTGGCTGAGTTCTCCATCTCATATAAGCCCGTCAAGCACGGTAGACC
TGGTATTGGTGCTACT

>comp69468_c0_seq1 204_1724
CAAGTACACAGTCCTCGGCATATTATTTTTGACTTCTCCCCTATTTTATTTCTCACAAAT
CTCGCATCTTCTCCCACGTTTCCTTCTTCCTCCTTCCCTTTCACTCCTTCTACTCACTCT
CTTTCACCTCCATTTCTCTGCAACAAAACAAGAAAAGAAGAAAAATGTTCTTTTTAATCC
CAAAAAACTTTTATTTTAACTAAATGGGCAAAGCATCTAAATGGTTTAGAGGGCTTCTTG
GCCTTAAGAAAAACGACCCATCATCTTCTTCCTCTACCCAAAATAATCCAAATCCAAACC
CAACTAAGTCCACTAAAAAAAAATGGAGCTTTGTTAAATCTTACAGAGAAAAAGATTCTT
CTTTTGTTAAACCTGCTGATAATAAATCGAATTATGGTGTCTCAACCGACGCCATTGGCA
GTGTTGATCCGACTAAGCGTGCGATTGCGGTGGCGGAAGCAACCGCTGTTGTGGCGGAAG
CTGCTATCGCCGCCGCCCAAGCCGCTGCTGCGGTGGTGAAACTTACTAGTAGCGGCAGAG
GTACCGCTGCTACTAATAATACTAGTGGTGGTGGTGCTGCTGTGATGACGTGGAACGGTG
TTGCTGTCAGCTCCTCCTCCGCCTCCTCTGCGGCGGTTGTTAACAGAAGTAGAACAGAAT
CCGGCAGCCGAGAAGATTGGGCCGCCGTCGTCATACAATCACATTTCCGGGCTTATCTGT
CGAGGAGAGCTTTACGAGCGTTGAAGGGGCTTGTGAAGCTTCAGGCATTGGTGAGAGGTC
ATATTGTGAGGCAACAAACTGCTGATTATCTACGGCAAATGCAAGCTATGATTAGAGCTC
AGTCGAGGGCTCGTGCTGGACGATTTCAGGTCTCGGGATCCCCTCATTCTAGCACCAAAT
CTGTTCAGTTTCTTCATGCTGGTCCTACAACTCCGGAGAAGTTCGAGCATGTTATCCGGG
CAAGGAGCATGAAGCACGACGAAACATTTATGCTCAAGCGGAACATCTCGAAATCAAATT
GGAAGGTGATTGATTCAGGAAAGGCACGAATTAGACCCCAAGGATCTTCTGCAAGAACCA
ATTCTTTCGATGATGTAAAAAGTGACAAGATCCTCGAGATAGATAGTGGGAAACCGTATG
CCACGCCTAAACAAAGGAACCTCTTTCACTCTTCCCATTTTAGCTTGAATTCTGACCAAT
ACAGCTATAGCTTAACAACATCAAAGGAATCAACAGCTCATCAAACGGTTCCCAGTCCAT
CGTCCTGTGGAAATCAACCCCTGAGCCCACTGAAGTTCAATCAAGAACTCGAGGAAGCCT
GCTTCTGCACTGCTGATAATAGCCCTCAGTTTTACTCGGCTTCTTCAAAAGGCGGTAGTT
CAAAGAGAGGACCATTTACTCCAACTAAGAGCGACGGTTCAAGAAGCTGTTTGAGTGGAT
ACTCTGATCATCCTAACTACATGTCTTACACCGAATCAGCCAAGGCTAAGGTACGTTCAA
TGAGTGCACCAAAACAAAGGCCACATTACGAGAGATCAAGCTCCATAAAAAGGTACTCGA

TTCATGGTTACAGCGAGTCAAGAACCAACTCACAAAAGGGTTCCTTTTATGCTAGCTTCA
CTGGTAAAGCTTATCCTGGTTCTGGTCGATTGGACCGGCTTGGAATGCCTGTTATTAGGG
CAGATCCATCTGGATTCAGTGGTGGTCTTCGGCACAGATATTAAGCTAATTGTAGTTGAA
GTTTTCAGTTTAATTCTATAACTGTTCATCAACTCTGATTATGCTAACTTTGTATTACCA
TTGGCATTTTCCTTGTTGGAACCACGGTGCTAATGTGAAGCAATTTATGTTTAGTTCTAA
ATCTTGCAAAAAGATGGAATATCTTGGTTATAGCTATATGCTGCTTCCATGAAATTGGGT
GTGATCAATTTACAGTAGTCTTACGGCCAATCCCCGGAATCTTGCGTAAATACGAGACGT
TTTGTGTGCCGAACTTAGCTATTGCTTGGCAGTAGATTAATGTAAACCAAAATGACTTTT
CTTTAAAAATCAATCCCTCTGTCCTTCAATCCCCCTTTACCAAATATATTTGTATTTGTA
TTTTTTTCTTTTTGGGATTTTGAATGATTCAACCATCCAACAATGAACTGAATTCAACAC
TGGCAGTCTGGCACCAGCCCATTTAAGCATATCTTCCATTTTCCCAGGAAGATCTTTGT

>comp77795_c0_seq2 2_1780
CCCACCCCCTCCCCCACCTCCACCGCCGAGGTTAGCACCTAATGAGAGTGATTTGGGATA
TTCAGATGATGAGGAAATGCAAAAAAGTAGTGAATTTAGAGAAAGAGGTAAAATCTTTAT
AGGCAATTTACCATTATGGGTTAAAAAAAAGGAGCTTGAAGAGTTCTTTAGGCAATTTGG
GCCAATAAAGAGTGTGATTTTAATAAAGGGTCATCATGAAACAGAAATGAATAAGGGATT
TGGGTTTGTAATATATGGGGGCTCAAGCTCAACTGCTGAAAAGGCAGCAACGAAGGCTGT
GGAGTTTGATGGGGTTGAATTTCATGGGAAGGTTTTGACTGTTAAATTGGATGATGGAAG
GAGAATGAAGGCAAAAACCGAGGAAAGGAAGAGGTGGGTGGAAGGGGAAGATGATGTTGA
GTATAGGTCCAAGTGGCATGAGGAGAGGGAAGGGTCAAGGAGGGATTTGAGGAAAGTTCT
AGATACAGCGCCAGAGAATTGGCAGGCTGTCGTGCAAGCTTTCGAGAGAATCAAGAAGCC
ATCCAGGAAAGAGTTTGGTTTAATGGTGAACTATTATGGAAGACGTGGTGATATGCATCG
TGCACGTGAAACCTTTGAAAAGATGCGTGCTCGAGGTATAGAGCCAACTGTTCATGTATA
TACCAACCTCATCCATGCTTATGCGGTAGCTAGAGACATGGAAGAAGCATTATCTTGTGT
GAGGAGAATGAAAGATGAAGGAATCGAAATGAGTTTGGTGACTTACAGCATCCTTGTTGA
TGGATACGCGAAACTGGGCAACATAGAAGCTGCAGAGCGATGGTTTAAAGAGGCTAAAGA
GAGGCATCCAACTTTAAATGCCATTATATATGGCAGCATCATCTATGCTAATTGCCAAAC
AAGCAATATGGACCGAGCTGAAGAGCTGGTTAGGGAGATGGAAGGGCAGGGTATTGATGC
TCCAATTGATATATATCATATAATGATGGATGGTTATACTTCGGCAGGGAATGAGGATAA
ATGTCTCATTGTATTTGATAGACTTAAAGAGTGTGGATTTGCTCCTTCAGTTGTCAGTTA
CGGATGTTTAATGAATCTCTATATCAAGATGGGCAAAGTCTCCAAAGCTTTTGAGGTCAG
TGAAATGATGAAGTTGGCTGGCATAAAGCATAACATGAAGACATATTCCATGTTGATCAA
TGGCTTCATTTATTTGAAAGATTGGGCAAATGCTTTTGCAATTTTTGAGGATGTAATAAG
AGATGGTTTGAAGCCAGATGTGGTCCTTTATAATAACATTATCAGAGCATTTTGTGGCAT
GGGTAACATGGACCGTGCTTTACGTATTGTTGAGCAAATGAAAAAGGAGAGGCATAGACC
TACCTCGCGGACTTTTATGCCGATTATACATGCCTTTGCTAAAGCTGGAGCAATAAGAAA
GGCACTTGACGTTTTCGATACTATGAGAAGGAGTGGATGCGTCCCAACTGTTCATACTTA
TAATGCATTAATTCTTGGCCTTGTTGGGAAGCGTCAGATGGACAAAGCTGTGGAAGCTGT
AGATGAGATGTTGCTTGCAGGAATTAGGCCAAATGAGCGTACATATTCAACCATTATGGA
TGGTTATGCATCTCTTGGTGATACAGGAAAAGCTTTTGAATATTTTTCAAGAATTAAAGA
TGAGGGTCTGGAGCTTGACGTATATACATATGAGGCATTGCTCAAGGCATGTTGCAAGTC
AGGCAGGATGCAAAGTGCTTTGGCAGTGACAAAAGAA

>comp77334_c1_seq28 1342_2196
TATATATGTGTGTGTGTGTGTGTTATTTGTTCACCTTTCGCCATGTCACTATAATTAATA
ATAATTAATATATGAGAATTGATAGTAAATTGGCAACTTTTACCCTAGCTACCAACCTAC
CCCTCCTCCTTTATCCAGTCTTGGGACTGGCAATGTAAGCAAAGCTCACACACGCAGATT
TATAATAATTAATATATAAATAGTAATTTATTTATCTTTTGAACTATTCATAAATTAAAT
TATAATCTTCCACGAATTTAATTGTCATGATGTAGCCACACGGTGAGCATAGAAGCAACT
ACTCTTCCGCCTTCTACTAAACAATTTTACTTTTCATGTTTTTCTTCATCGGTTTTCAAC
TTCTTATTTTTGTTAAAAACGGTACATAAATGTTTTTACAATTTATATAAATAACCTAAA
AAAGGGGACAACCCGTTGCAAGAAGCATCCAACATTCACGTAGGGTTTAGGTAAGGCTGC
ATCCTTAGGGTGTGATATAGGCAGCCTACAGGCTACCATAATGCAAGTATCAATGGCTTA
TATAAGTCACACGAGACAACTTTATCGCTGCTCCAAGAATGTCTTTCCAATTTAATGATA
AAAATTAGCTAATTTATATATAGTACATCAAATTCAAAAAACAACACAGTATTCCTCGAA
TACGTCCTCTCTTTTTGAAAAAGCCGGGCAAATTGCATTGTCTCATCTAAAATGAAGGAT
AAATAATGCCCTGGATTTTGTGATTTGAACCTGTGATTTTAGCTGAATGGATAATAGCCT

TCATTATGCTAAATTCAATCATTTATTTAAATAGTTAATTTTTTACGGCCGAAGGGCTTG
CTTAAATATAGATCCAGCCATATTTGCATCTAATGGGCTATATCTTTGTGCTTCCTTTTG
GCAATACAGCTAAGGTTGGCATGTTTTTTGCCTTTTCATAGGCTTATCCAGTGTTTGCTT
TTTGGTTTTACACATATTCCAAGGTGTACAGCTGTATAAAATTTCAACGTCTCTCTCAAG
GACTCCTATTGAGATTGTGCCCAGGTTTCAAATCCCTATTACCATATTCCTGTCTCATGG
AGTTTTGACTGCAGTGTAGCCATTCAAATTTAGTAAAACTTTTATTTTTTTGGCTAGAGA
AAGGTCTTTCTTTTTCATTTGGTGGTTGAGGGGCTTGTTAGGCAAATATGTAGCTCATTC
TATTTCACATGTGCAGTGAGTTATTGTTCTTATTATGGGAAGAAGTTTGACCCTGAGCCA
GGAAGATGTAGAAGAACTGATGGAAAGAAATGGAGGTGCTCCAAAGATGCACATCCTGAC
TCAAAATATTGTGAGCGGCACATGCATCGAGGTCGCAACCGTTCAAGAAAGCCTGTGGAA
ATTATGACAACAACATCAACTAACACATCAACAACAACAAGACCTATTTCTAACACTACT
ATTAATACTACAACTCCAACAGCAATTTCCATCTCATCAATCAACAAAAATACAAACAAT
ATTTCCTCTTCTAGCTCCAATTCTTCACATCACTCTTTTTCTTATCTTACTTCAAATCAT
GAACCCCACCAATATCCTTTCCTTTATCCTCATTCTTCTTCCTCTAGACCATCCCCTGCT
GCCATTGGTTTGGAATCTTCTACTTATTCCAGAAATGGATATGGAGATGGATTGAAGGAG
GATGTAGATGAGCATATTTTCTTCTCAGAAACTTCAGGGACAGTGAGAAGTACATGTGGT
TCAAATTCAGTGGATGATACTTGGCAATTATCACCATTAACAATGGCTTCACCAATGAAA
CAGAGAACGTATTCTCTATCACAAAATACAGGACACTCAGCTTCTTCTTACTTACAACTT
CAAAGCTTAAATGAATCATCAAAAGATCATCATTATCAGTATTATAATGATGCCAAAAGT
ATGAAATTTGAGACAGAAGATCAGCAGCAGCAGCCTAAGAAAGTAATGCACCATTTTTTT
GATGAATGGCCAAAAGACAACAAAGATTCTTGGCTTGATTCTGAGGACAAATTTTCAGGC
CTTTCGAAAACTCAACTCTCAATTTCAATACCAAATCCCTCACATGACTTCTTCATCACC
ACTAATGGTAAAATTATCAAGAATCTCTTTCTTTTTTTGTTTTTTTTTCATTTTATAACT
TTATATGGTCGAAAGGAAGTCTTGAAGCAATTGTAAATTTGTTTTCGTGCCCGTTTGGAT
TGACTTAAAAAAGTGGGGTTTCACAAGAAATAACTTTACCAAAAAACAATAAGTTGAAGT
GTTACCTTGCTTATTGCTTTGGCTTGTTTTAACTTTTAAACTTTGCCAAATATTGAAAAA
AAAGGGGCTAAGATGAGCTTAAAACCGATTTGACCAGTTTAAAAGCCAATCTAAACACCC
TCTAAGTTGGGGTACTGCCCTTTCCTACCCTCTATCTATCTATCAGTGATTTTTTTTTTA
ATCACTTTAACATGGTTATGGCCAAAAGATTTATGTTTTGACCAAAATAGTTGAACTTTG
TTTTTGCAGAGAAATGAGCTTTGTGAATCAA

>comp79406_c0_seq1 393_3506
CTTTGCTTCTGCTGCTTCTACTGTTGAATCAATAATAGCCATATTATCTTTCCTTTTTCA
TATGCAAATTACTTAATATATGCAGGTTTTTATTTTTTATATTTAATGTGATGTAAGCTG
TCTTTAACTCAAGAAAGAGAAAAAAAAGAAGAAGAGAAAAACTCACAAGCATCTTCTTGA
TTTCTTTAATGGTAGAAAACAAAGCCACTCTCTGCTAATTCTTCTTTTGACATCACAAGA
AATATTCTTTTTAAGATCTCCCAAATACTACTAGGAGAAGTGGGTAGCTGTGTTCTTGAA
TTTCTTAAAGGTCCTTTCTTTAGTTGCTTTTTTGCTTCTATGAGTTGGTGCTGTTTGTGA
TCTTTTTGTGATTTATTTTGATGGGACTGTAAATGGGGTCGCTTGAAAATGGGATTTCTT
TAAAGAAAGATCAAAGCTTGCTTCGTTCTGCATCAGCAACTAGTGGGAGGAATAATGCAT
TTGGTCAAAGAGCAGTCAGATCGAGATTTGCAAGATTCTTGTTTGTTAACAAGATCAATT
ATTTACAATGGATTTGTACTGTGGCTGTATTCTTCTTCTTTGTTGTTCTATTCCAGATGC
TCTTGCCTGGTTCTTTAATGGAAAAATCAGGGAATCTGAGTTCTCAAGATAGTGAAGTAG
TGGATTTAGCTCTCTTGAAAGAGTTGGGGGCTTTGGATTTTGGGGAAGATATTAAGTTTG
AGCCTTTGAAACTTTTAGCTAAATTTCGTGATGAAGCTGTGGAGGCCAATGGGACTGTTG
CTTCAAGGACTGTACTGAGATTTGGTTATAGAAAACCTAAGCTGGCTTTGGTATTTGCTA
ATTTGTTGGTTGATCCATATCAAATAATGATGACTAATGTTGCAGCTGCATTACACGAGA
TTGGCTATGAAATTGAGGTGCTCTCACTTGAAGATGGTCCAGTGAGATCCATTTGGAAAG
ATGTAGGAGTTCCAGTCATCATCATGAACACCAATGGGGATACAAAGATCTCTGTAGATT
GGTTAAACTATGATGGCCTACTGGTGAACTCTCTTGAAGCTGTCAACGTCTTGTCTTGTG
TAATGCAGGAGCCTTTCAAGAATGTACCTCTGGTATGGACCATTAATGAAGTTACACTTG
CTTCTCGGTTGGAGCAGTATATTTCAAGTGGGGAGAACAATGTTGTGGACAACTGGAGAA
AAATCTTTACTCGTGCCAATGTTGTTGTCTTCCCAAACTATATCCTACCGATAGCTTATT
CAGTCTGTGATGCTGGAAACTACTTTGTTATTCCGGGTTCTCCTAAAGAAGCATGGGAAG
TTGATATGTCGATGGCTGTATCCAATGATAATTTACGAGCTAAGATGGACTATGCACCTG
AAGACTTTGTTATTGTGGTTGTGGGGAGTCAGCTTCTATATAAAGGCCTCTGGCTAGAAC
AAGCCCTTGTTTTACAGGCTCTATTACCGGTTTTTCCTGAATTAACAAATGATGGCAATT
CAAACTCCCGTTTCAAGATTGTTATACTGGCTGGGGGTTCAAATGCCAATTACACTGTGG

CTGTGGAGGCTATTGCGCGGAACTTAAGATATCCCAAGGGAATGGTGAAGCATGTTGCTC
CTGCCGAAGTTACAGATAAAACTTTGAGTGTGGCTGACCTTGTCATATATGCATCTTTCC
GTGAGGAGCAATCTTTTCCGATCATTTTGCTAAAAGCAATGTGCTTTGGGAAACCTATAG
TGGCTCCAGATCTACCAATGATAAAGAAATACGTCAATGATAGTGTGAATGGTTATCTTT
TTCCAAAAGAAAATGTCAACGTTTTAACACAGATCATGTTGCAACTTGTATCAAATGGTG
AACTATCAGTTCTAGCCCATAATGCTGCTTCAGTTGGACAACATACTGCAAGGAACCTTA
TGGTTTCAGAAAGTGTGGAAGGGTATGCTTTATTATTGGAGAACATTCTCCGATTTCCAT
CCGAAGTTGCATATCCCAAGGCTGTTACTGAAATTCCTGAAAAACCAAAAGCAGAATGGC
AGTGGCATCTTTTTGAAGCAATCAAAACAAAATATTCTCAGAACAAGACTTTGAAGACCT
CAAGTTATTTGAATAAGATTGAAAGGCAATGGAATCCGACCCAAAAAGAGGGTTCTGCAG
CTGTGGTGGAGAAGAACGAGAATTTTTTGTACGACATTTGGGAGGACCACAGAAATACTG
AGATAGCTAATGTGAGAAAGCGAAGAGAAGACGAAGAGTTGAAGGACAGAACTGATCAAT
CTCGGGGAACATGGGAGGAAGTATACAGAAATGCCAAAAGGGCTGATCGGTCTAGGAACG
ATTTGCGTGAAAGGGATGAAGGGGAGCTTGAAAGAACTGGTCAGCCATTGTGCATTTATG
AACCTTATTTCGGCGAAGGGACCTGGCCCTTCTTGCATAGTACAACACTTTATCGTGGTT
TTGGACTTTCTACCAAAGGGAGACGATCCGGTCATGATGATATTGATGGTCCTTCTCGGC
TTTCACTTCTAAATAACCCTTACTATAGAGACGTTCTTGGTGAATATGGAGCCTTTTTCG
CTATTGCTAACAGGATTGATAGGATACACAAAAATGCCTGGATAGGGTTTCAATCTTGGA
GAGCAACAGCAAGACAGCAACTGTTGTCCAACACTGCTGAAAAGTCACTAGTTGATGCTA
TTGAAGCGCGAAGGCATGGTGACACACTCTATTTTTGGGCTCGTATGGATGTGGACCCAA
GGAACACGCTTAGACAAGATTTCTGGTCATTTTGTGATGCTCTTAACGCTGGAAATTGCC
AGTTTGCTTTCTCTGAGGCGCTAAAAAAGATGTATGGCTTAAAACAGAATTTGAGCTCTC
TTCCTCCCATGCCTATGGATGGAGACACATGGTCTGTCATGCACTCTTGGGCTTTGCCTA
CCAAGTCTTTCCTAGAGTTTGTTATGTTTTCAAGGATGTTTGTTGACGCACTCGACTCAC
AATTTTACGAAGATCACCATCGAAGTGGCCGATGTTATCTGAGTTTGACCAAGGACAAAC
ATTGTTATTCTAGAGTCCTTGAGATGCTTGTAAATGTTTGGGCATACCATAGTGCAAGAA
GAATGATGTACGTGGATCCGCGGACGGGCTTAATGCAAGAACAACATAGACTAAAAAACC
GTGAAGGCAAAATGTGGGTGAAATGGTTTCAACTTAACACTCTTAAGAGCATGGACGAGG
AGCTGGCTGAGGAGATGGATACTGACCACCCAAAAAGAAGGTGGTTGTGGCCGTCAACTG
GTGAAGTGTTTTGGCAAGGTATCTATGAGAAAGAGAGAAATTTGAGAAACAAAGAGAAGG
AGAAAAGGAGGCAACAAAGTAAGGATAAAATCTTGAGGATTAAGAAACGAACTCATCAAA
AAGCATTAGGAAAATATGTCAAGCCTCCACCTGAGGAGTTGGAACATCCGAACACAACGA
CGACAACAGCAACAGTTATGAGGTAGCACTAGCCTACAGTAGTCTGTCCAAAGTTAAGTA
GTCTAATTTGGTTAATATTATTTTTTCAAAATCCCCACCATTTTTTCATTTTTGTTCAGA
GGGATGCTATAGGTTTATGTTGGGTATGAGAGGATATTGAGCATAGATGCATCCTTCTGA
TATTGTTGCCAAGCATATGCATGTATGGTAGTGTTTCTTTTCTCCATTTTTTCTTTAGAA
AATTCTCTTTTCCTTATTTTGCATCGCTAATTTTCACTTCAAGATTCTCTTTTTACTTAT
TCATATAGGAGACTGAGAGGGATGGATTTGTAAACTAACAGTTACACGCTGAGCTTCAGC
GATTGTATGTGTTGTAACTTGAGAAAATTCTCTATACCTCTGCTATGATTAGTTACTTTG
GTGTAAACTAAGTTGTCGCTGAGTTAAGCTAAAAAAGGCGCATCTTATTTGTGAAAGGAA
AGGCC

>comp76253_c0_seq1 643_1557
TGAACATCTCTTTATTTATTGTTTGTTGTTTTCTATCCTACAGTGTTCATCCGTTTTGGC
TTTGATGCCTCAGGCAAGTGTCAGCACTCTCAGCTGTATTCTCATAAACTGAGACCTTCT
CCAATTTCTGGCTAAGTAAATTTTTACTAGTTGGTTTAGTTGCAGCACTTTGCTTTTCTC
CCATGTAAAAAAGTCAAGAAATGCAGCCTCATCTCTCTTCTGCCACACCCCTTTCTCACT
CTGGTATCTCTCTCTCTGTTACTACATGTTTATATTTGTTTTATCTGCGAAAGATTGAAT
TTTGCTTTGTGGGGTTTGTGATACTGATGAAATTATGCTTACTTGGATGAAGGAAAAGAT
GAAAGAGAGAAGAAAGAAGGGTCACCACCTTGCATAAAGTTGTCTTTAGGCTATGTGGTT
GAGGAAGAAGAAAAAGGTGTCAATGCGATTGCCAAGTCTGTGTTCACAACAGCTCAGTTT
CATGAACTACAGCTCCAAGCTGTGATTTTTAAGTACATAGTCTCTGGTCTTCCAGTTCCT
TTTCATCTCTTTTTCACCATTTGGAATACTGTTTCCAGCTCATTAGGCGCTGCTGCAATC
AACAGACTTTACCCTACCTTGGGAAGATTTGATTACGGAAGCATGATGGATCCTGAACCA
GGGAGGTGCAGAAGAACTGATGGTAAGAAATGGAGGTGCAAAAGGGATGTGATTCCAGGT
CAGAAGTACTGTGGGCAGCACATGCATAGGGGTCGTCGTTCAAGAAAGCTTGTGGAAGCT
TCCGAACATGTTATGAAATCAGATGATATGCGTATCACTTCCAAGAGAACTAAACTGTAT
GCTAATTCTGACGATCCCGGTAGCAAATGTGCTCCCGCTAGTAGTAAAACTTCTTGTTTG

TCTTCTACTAGCAGAAATAACCAAAATAACAGCATCGAGGATTCATCTGCTAAACCAAGA
CCCACTAATTCCTGCAACAGCAGTGAGAAACAAGACAACCACATGACTGTCAATAGAAAG
GACACCACAACTGGCATAAGAACCCAATCTTTCATCGCTGCTAACGATACTGATAGCAAG
AGTATTGATTTCAATCGTATTAGTCCAAGCAAAACCAACCAAAAGCAGAGCTATGGAGAT
GCTAGGAATAGTGGTGGTTTGGTTCCAGTTTTTGGTATATCTGCAAAGATTGATCTTCAA
CACACTACAGAAACTGAACCGCAGAGGTGCAGAAGATCAGATGGTAAGAAATGGAGATGC
AGCAGGAATGCTATTCCTCGTCAAAAGTACTGTGAGACGCACATGCACAGGGGAGCCAAA
AAGATAATTGCAGCCTCAGACTCGGTCATTGTCGAGCCTTCAAAACCTTCCTCATACAGG
TTTTGCCATCAAGTTATACCTAAAAATGATGTTACTGGAATAAACTTGAACACCAGTCTT
TCCATTTCAACACTCCCGGATCCTCAAAACATTACGGAAGATGATAACTCTAACAGTAAC
AGTACTAGTGATGCAACCACTATCACTGATGAAATACCATCATTTGTGTCGCATTAGTTG
AAAAATGCGGAGGCTTTTTGATATTGGTATTCTTTTTTGGCTAGATGTCTGAACTTGAGA
AGTGATCAAGGTCAGTTTGTGTAGTAGTTGTTAATGGTCATTTACATGTTCCTAGTTTAT
AGATTTTTTAGGATTATAAATTCTGTTTTGTACTAGGTTTGATGGATCAGATGAACATGT
ACTTGTTCAATCTGTGGGCTAATCAATGACTATTTTTGTTCAACGAGCAAGTTCTA

>comp69128_c0_seq2 114_806
ATTATTCTTCTGCTATCGTAAGACAGAATTCTTCTACTCACTCAATTGCATAAACCCCAC
CTATAACCTGCTTACGAAGGCAAAACAAGAAGAGTAGAAAAATCAAACCACAAATGGATC
TTAATGGGTTTGCAGCCATGGACATGGATGCTGAAAATCTGGGTCCCTGTTTAACCAGCT
TTGTAGATGATGGTACTGTTGAGAGTCACAGGTACTTCCTCGCGCGCAGAACAGTGCTCG
AAATGCTTAAAGACCGTGGTTTTGCAATACCCAATTCGGAAATTGAGTCTACACTTGAAG
AATTCAGAGAAAAATTTGGTCAAAAACCTGATATTGAGCGCCTCAGAATCTCTTCCATGC
ATAGGAATGACCTTTCCAATAAGGTTTTGGCAGTCTTCTGTGGGCCAAATGCTGTGAAAG
TAAATGTCATTCGCAGCATTTTAACTCAAATCATGAACAAGGAATCTTTGAGTAGGCTGA
TATTAATCATTCAGAAACCAATGACCGCTCCAGCTATGAAAGCTGTGGAACTCCTCCCAT
TTAAAGTTGAAATTTTCCAGATCACAGACTTGCTCGTCAATATCACAAAACATGTTTTAA
AGCCAAAGCATGAGCTGTTGAACAATGAAGAGAAAGAGAAGCTGTTAAAGAAGTACAACT
TGGAAGAGAAACAGCCCATTAAGAAATCTGGATCAGTATATGAAATTCTCGGACATGCTA
GGAGGGATAGAGATAGAAGGTGGATGATGTTGTTTGTTGCCGAGGATGTCTCAGAAGGAT
GCAATTGCTCGGTACTATGGACTTGAGAAGGGGCAGGTGGTGAAGGTCACGTACAGCAGT
GAAATCATCGAGACGCATGTTACATATCGCTGTGTCTGGTAATGCGACTATGTTGTAAAT
CCTATCTAGCAGCCTTACTGTGTTCGTTAAATTAGCCTGCAGCAGCGTAACTTACCACAA
CATGTTCTGTTAAAACGATGCTGGTTTAACTTTTCCTAACTCCCTCTTCCACCCCCCTGG
GTTCCCTTCTCCACTTTAGCTCAAATGAAGAGCTTGTAGGTAGTATATTGGTACACTTTG
TCGTCGTTTCTCTGCGTTTTATATGCCCAGATTGTCTGTACTTATTGTGCTAATAGGGGT
GGTGGCTACTCTAATTTGCAAAATTTGACCATGTTGGAATGTAATAGGAGCTTTAAAATA
GCTTTTCTTAGCTTTTTCATTTGTGAATCGTAGCTAACTTATATATTGGGAAAGAGTTTA
ATTTCGATACAAAAAAA

>comp73318_c2_seq1 87_1109
TACTTTTGCTACATTCACTACTATTCTTTTCACTAAGCAATTTTCTCTCCTAATTTCTTT
AAACCCCTTTTTTTCTCCCCTAAGCCATGGCATCTGACAAGAAGATCAAGATCGGAATCA
ATGGATTTGGAAGGATTGGTCGTTTGGTGGCAAGAGTTGCTCTGCAGAGAGATGATGTTG
AACTAGTTGCAGTGAACGATCCATTTATCTCTACTGATTACATGACGTACATGTTTAAGT
ATGATTCAGTTCATGGACAATGGAAACACCATGAGCTTAAAGTCAAGGATGAAAAGACCC
TTCTTTTTGGTGAGAAGTCCGTCAGAGTCTTTGGAATTAGGAACCCTGAAGAAATTCCAT
GGGCTGAAGCTGGTGCTGATTTCGTTGTGGAATCCACTGGTGTCTTCACTGACAAGGACA
AGGCTGCTGCTCACTTGAAGGGTGGTGCCAAGAAGGTTGTGATCTCTGCTCCTAGCAAGG
ATGCCCCCATGTTTGTTGTGGGTGTCAACGAGAAGGAATACAAGCCAGAATATGACATTG
TCTCCAATGCTAGTTGCACTACCAACTGCCTTGCACCTTTGGCTAAGGTCATCAATGATA
GGTTTGGCATTGTGGAGGGTCTCATGACTACTGTCCACTCCCTTACTGCCACCCAGAAGA
CTGTTGATGGTCCATCCATGAAGGACTGGAGAGGTGGAAGAGCTGCTTCATTCAACATCA
TTCCTAGCAGCACTGGTGCTGCCAAGGCTGTTGGAAAAGTACTCCCAGCTCTTAATGGAA
AATTGACTGGAATGGCCTTCAGAGTTCCAACTGTTGATGTTTCTGTTGTGGACCTTACTG
TAAGACTAGAGAAAGAGGCCTCTTATGATGAGATCAAAGCTGCAATCAAGGAGGAATCAG
AGGGTAAGTTGAAGGGCATCTTGGGATTCACCGAAGATGATGTGGTTTCCACAGACTTTG
TTGGAGACAGCAGGTCAAGCATTTTCGATGCCAAGGCTGGAATTGCCTTGAGCAAGAGCT

TTGTGAAACTTGTGTCGTGGTATGACAATGAATGGGGTTACAGCTCTCGTGTGATTGATT
TGATCTGCCATATGGCTTCTGTTGCTTGAGGATTTGATGCTGGTGAAATGGCCTCTTTAG
TTTTTGATTGAATCATAGGAGTATTAGTTTTCTATGGCCGGGAGCGGTCTTCTTGCTTAA
TTGTAATGGAATAACCAGAGAGGAACTACTGTGTTATCTTTGAGGAATGTTGGGCTTTTT
TCATTTGAATTGTCATGAATGAAATTTTACTTTTTTCCAATACGAAGTTTGTTTTCGTTT
CTTGGTTTTTGTTATCCTTGGTTTATGTCCTGGTTTGGCTTAAAAGATTGAAGATTACAC
TACCTATGTTTCTGCTACTTCTTCCTGTTGAAGATCACATTTGATAATGCATTAAAGTTT
CTTATTGGCTCTGTGAAAAGTATTAAAGGTGGATTTCTAATTGGCAAGCTCTCTC

>comp75344_c1_seq2 127_1839
GGCTTTTGTAGGTGTACAAACTCCTCCTTATATAAAGGTTTCATTTCTCATTTTTCTCTC
CACCAAATTCTTCAGCTTCTACCTTGCCTTTACAATTGAATTGTTGTATTCTTCTTTTTT
GATCAAATGGGGCATCAGGTTTTCTTGTTTGGTTGCACTATAATAGCTTTTCTAGCTTCT
TACTCCTCTTTGGCTTCAGCTGCAGTTGTTGAGCATTCTTTTCATGTGCAAAACCGTACT
ATAACGAGACTTTGTCGTAGACAAGTGATCACTGCTGTAAATGGAAGTCTTCCTGGTCCA
ACCATACGTGTAAACGAAGGAGACACCCTTGTTGTTCATGTCTTCAACCTTTCACCTTAT
AATCTCACTATTCATTGGCATGGAGTGTTTCAGCTACTAAGTGGGTGGGCTGATGGACCC
GAATTTGCGACCCAGTGCCCGATCCGACCCGGACATAGCTACACCTACAAGTTTAACGTA
ACGGGTCAAGAAGGGACTCTGTGGTGGCATGCACATGTATCATGGCTTAGAGCCACTGTA
CATGGTGCACTCATTATTCGACCCAAAAAAGGAAACTCTTATCCTTTCCCTAAACCTTAC
AGAGAAGTTCCTATCCTTTTAGGAGAATGGTGGAACGCCAATGTTGTAGACGTGGAGAAT
GAAGGACTAGCCAGTGGCGCTGCACCTAATAACTCTGATGCTTACACCATTAATGGATGG
CCAGGAGATCTTTACCCTTGCTCTGTTAATCAAACATACAAGTTGACAGTGAAACATGGG
AAAACATATCTCCTTCGTATCATCAATGCTGCACTCAATAATCAGCTCTTTTTTAAGATC
GCAAAGCATAAAATGAAAGTTATCGCGGTTGATGCTGCTTACACTGATTCTTACGTTACA
GACGTAGTCGTTACGGGACCAGGTCAGACAACCGACGTCCTCTTAACGGCCGATCAGGCG
CCGGCGTCGTACTTCATGGCGGCTAGCCCTTACGCTAGTGCAGCCGGGGTACCATTTGAC
AACACAACCACAAGAGGAATTATAATATATGAAGGTGCACAGACATCAACTCCACTAATG
CCAATTTTACCAGCCTTTAATGATACACCAACAGCCCATAAATTCTTCACAAATATAACT
GGGCTTGTAACTGGGCCATTTTGGATCCCTCCACCTCGTAAAGTGGACGAACACATGTTT
ATTACTATTGGGCTGGGCCTAACTGCTTGTAATAGACAAGGAAATGCAACATGTGGAGGC
CCAAATGGACAAAGATTTGCTGCTAGTATGAATAATGCATCTTTCCAATTACCTGACAAG
ATTTCAATGTTGGAGGCATTTTTCCACAACGTGAATGGAGTTTACACTACTGATTTCCCT
AACCAACCACCACTGAAATTTGACTACACAAATGCTAATAATAGCATGAATCCTGCTATT
ATAATGACTACAAAGTCTACTAAAGTGAAGAAAGTTAAGTTCAATGCCACAGTTGAGATT
GTGTTCCAAAACACTGCTTTGATTGGGATAGAGAACCATCCCATTCACTTGCATGGATTT
AACTTCTATGTCTTGGCTCAAGGATTTGGAAATTATAATCCCGCAGTTGATCGGAAAAAA
TTTAACCTTTTCAATCCTCAAGAACGTAACACAATTGGTGTTCCAGTTGGAGGATGGGCT
GTCGTTAGATTCCGAGCTAATAATCCAGGTGTATGGTTGATGCATTGTCATTTGGATGTT
CACTTGCCTTGGGGATTAGCTACATCTTTTGTTGTTGAGAATGGGCCAACACTATCAGCT
AGGCTACCTCCACCACCACCAGATCTACCTAAATGCTAGATTATTTCCTTTTTGTTTATT
TTTATTAGGATAGCAGAGTGTGATTATATGATTCTTGGTTTGTTTCATTCTTCCAATTGT
ATAAGCGAGTCATTTGTTTTCTGGTCATTTTTTGACTTGTTAAACAAAACATGGTTGTAT
TTAGTTATTATCATTCTTTAAGATTTATGTTAAGTGTAATGTATCATCCTTGCCTGAGAT
TGCTCTTTGACTTATGAATAAAGTTACTGTCTTTTGGATTCCTGCTAAAAAA

>comp77652_c0_seq1 1261_3177
CGATTCCTATATTAACAAACTAATGAAAGTAAGCCATACAGAAAGTTAGGAAGGAGTAGA
TATGCAGAATACTTAGAAATATCATTGAGTGCAGCGTTGATGAAAATGTCGAATTTCTGC
AGATATAATGATGCTGCAATATTAGTCATTTCACAAATCGTTTTCATCTACGCTGCACTC
AATCATGTTTCAGTGCTCTGAAGAAATAGTTAGTTTGCCCTAATACAGTCCCACAATCTT
CTGTTGTCTACCTCCTCACAAGTTAAGACCTCAAGTTTAACATCTTTTTTTCGTAGAGTT
AGCAACTCATGGTTCAAAACTCGATGAATACTAAAACCGCTCATCTATCCTTCTCCAGTT
GAATACAAGACTTTTTGTTTGAGGCAGATTTGAACCTGTAATGTGCACCAGACCCACACA
TCATGTGTTGCACCTCCAGTCTGCTATTTCATACCCAATCCAGTCAACGGTAAAAATGCA
ATCCTCTCCAATGACAAATTAACCATTACCCAAGATCAGATGACAAACAAAGGTTCATGG
CCATGTCAAAACACTACAGAAGGATCTTCCCCATGTTGCACAGTCGAATGTTTCATAAAA
CTACTCAGCAACTAGTCAAGCAATATACCAAATTATATTGAATATAATGTCCTCAAGGGA

AAAAATATTACCCATAAGTGGAGGAATTGCCCATTACACATCAGAAGCATCTTCCCCATG
TTGCATAGCTGAAAAGTTCATAAAACTACTCCTCAACTAGTTGAATATAAGTTCCATACG
AGACAAGATTACCCATAAGAGAAGGTATTAATCATTGCAAATAGAATCAAAAGAAGAGTT
GAGATTTGTCGCAGAATCTTCAGATCGCTATGAAAGTACTTACTACACAATCATTGGGGA
AGTAAAAAGTTGGACTACAAAATTTCCCCCACATTTCCACGGGTTTGATCACGGAATTGG
ATGCATCAGCCTACCATGAGACATACGGCCTCAATCCCAAAACTCTCTCATCAATACATT
CTTGATATTCATTATCTCACATAGTGATCAAGAATAAGAAACAATAATAAGAATCAAGGT
TAGAGCTAAACTAAACTCAATGTTTTTTCTAAAAGGCATTTCATCCTTATTCTCTTATAA
TACCAGATGTTGTGTATATTATGATATTATCTTTCCTTTACGAGATATTACTCCATCAAA
ATTGTGGAATCGTTTCCCCTACATTGGGGTCGGGAGCAGAAGTAAGCAAATGAGACCTTT
TTAAGGGGCCATCAGTCCTCCAATTGTGTGAAAGAGAGACTCCGCCCAGTCTTCTTTGAA
AAGCAACTTTTGCAATGTCTGCAGCACATCATATTCCGGATCCAGTTTTCGCTGCCATCC
CTCCAACACCAAAGTAGTCACAATCACAGTGCAAATGTTGCCATCAATATTCACCCGATG
ACGTCTAACTTGCTCAAGCAATTGTTGCATGCAATCCGCCGGATGAATGCCACCTCCTTC
AGGTGTTCTCCAAAAGTCAAAAGTTCCCTCCACTTCCTTGATGAATTCCCCTGGGTTCGG
ACAGCTCTGTTGTTTAGATAGTCCAAGAGTACATTCTGCAGCAGAGCGGCCATCTCGAAG
TGCCACAGCCTTAAAGAACTCCAGCAGAAGGAGCCGATCCTTATTAGATAGCTCAGCAGT
CATACCCACATCTAGGAAGACCACATGAGGCTTCGACTTGAAAAGTCCTTTACCTGAAGC
TCTGTCTTGCGGTGTTCTAACAAGAATGTTCCCAGGATGCATGTCTGCGTGTATGAAATT
GTCCACCAACATCATCTTTAGTAGTGCATGAGTCCCAATGTGGGCAAGGGCACTTCTGAC
ACTCTCACGTCCGTCGAGCTCTTTAATACGTTCTTCAAGCTCTTCAATGTAGCGCAAGAT
ATTTTCACCATCTTCATAAGTTTCCACTAAAACTGCTGGATGCACCAAAGGATATAGAGG
TCTGGGAAATGATACATCCTTCCATCTGCGGAAGTTGTATATGAACCGACTTAAGTTAGC
TGCTTCCCTAGAAAGATCAACTTGAGACATCATAAAGACCGCAAACTGCTGTATGCTTTC
ATCCAGTCTTAACCACTTCAAATTCGGGAAGACCTTTGAAAGTTTTGCAAACAAGTTAAT
TAATACGAAATCCCTCCTTATAGATTCACCAACGCCTGGGTGCCTAACTTTGACGGCCAC
ACGAATGGGTTTAACCCGTTGTCTAGGATATCGAAATCTCAATGTAGCCCGATGAATTTG
AGCAATACTACCAGATGCGACAGGCTCCTCCTCAAAGTTTTCGAATATTTCAGGAAGCTT
GCGTCCGAACGCCTTTTCGATGCTTCTTTTTGTGTACACATAGCTATGTGCTGGTGCCTT
AGAATGGAGTTCCGCAAGTTCATTACACATATCATCTGGAAATAGATCTGGCCTTGCTGC
CGCCCATTGACCCCACTTAATGAACGCTGGACCCGCCTTCTCTAGAGTTTTTCGCACAAC
ACGAAGCCACTTCTTCCTAAATTCAATACCAAGGGAGTCTGCAAAAGGAGCCATAAGTAT
ACACGGGAAGAACAATAGTGTTAAGTATACAGCTCTAAAGAGCAAAATCAAGCCCTCTAA
AAGTAAGAAGACAACTGAAGTCAGGTAGACATGTCCATCCTTTGCATGCATGTAGAGTGA
TTCCGGGGATGTGAAGCCTTCTGCTTCGGCAAACACACTTCGCTTCCATGCTAGCTCTCC
AATAAGGAAGGCTAAAATGCCAGGAGAAACCAGATGTGAGCGATTCAAAGCAAGGCTCAT
CATACATGTAACCCTACTTATCGGTGGAAGAATTGCTCCGCCGCTTGAATGAAGTTGAAG
TAGCCGTTTCCAGGCAATTCGAGCATGGTGTGACACTGTCTCACTTGTTAGGAACAAACA
GCTCGATTTAGAATTGTATGCCTTCCATAAGGTTCCTTTTGCCTCATATAAAATGCTGGA
GGTAAATCCTCTATGACAAACTCTACATTGTGAATGAAATCTGGAAAAGATGTAAGAAAG
TCCGGCAGGGCAAGACGTCCAAACCACTGAGTAGCTGGCCTTTGGGCTGGCAAGAAAGGA
ATGAGCAACCCTTTTAACGCTGCCCTTCACCAAAATTCTGCACCTTGATGTGGCCATTGA
AAACTGGTGCCGGCATTTTTTTTCACTCCCACGCAAAGAATCTATGGCTCATGGCGTCCGA
GTGAAGACAGAAAAACGAGCTGCGAATGGTTGTTTTGGGCTCAGTGGCGCTGCCGTTTGA
CGGATGTGCGCGCATCAATGGACTGAAAATCTACCGGGTTGTAAGAGTTTTGGGCAAATG
ACCCGATTTCGTTCCCAAATTTCAACCTGCTAAATTACTTTAAATTTTGATTTTGATTTT
GATTTATTATTTTTGTGGTCTTATTTTATGTGACGTAATTTGACTAGTTATCA

>comp100607_c0_seq1 102_557
ATAAGTAAACCCCTAACTATCGTCCATTCTTCACCAACGAAACCAGAAAAAAATAGAAAA
AATCAGCAGGGACGACCTGAGGAACATAGACACAAGTGAACATGGCAAAAATACTAGAAT
TAGTCAGTTTCATTGTGGTTTTTGCATTAGTTATAAATTGTGCTGCTGCAGAAACATATA
ATGTTGGAGGAAGTCTAGGATGGACAGTTCCACCAGGTGGAGAAGCTGACTACAAAAATT
GGGCTGCTAAACAAAGCTTCAAGTCTGGTGATACGTTAGTATTCACATGGAGTGGAATAC
ACACAGTGGCTTATGTGAGCAAAGAAGATTATGAAAAATGTTCAACTACAAATCATAGTC
AAGGCACAAGCCCAGTAACCATTAGTATCAGTGGTACTGGCCCTCACTACTTCATCTGCA
CCATTGGCAAACACTGTAGCCTCGGCCAGAAAATGAACATCACCGCCGCTGGATCGTCGG
CCGCCACCTCGGCTTTAGCCACCACTGCCTTGTCTGCATTGTTGTTGATCTTGCCTATTG

TCATCACTTTCATATGAAAATGAAATCTTATTTCTATGTAATTATCTCAAAATATTTGCT
TTTGAATGTTTTCGTTGCTCTAATTGTGATTAATATGTTAATCTTTGATTTCAGTGTACC
ATATAATAACAATTTGTAATGGATGAAATACTTTATTCAATAGACCATTGCAGGAGTGGA
TGTAATAATTTAATTAAATGTAAATACAAGAGACTTTATTAAAAAAAAA

>comp85708_c0_seq1 121_624
AGTTGGGCTAACATCATTCCAAACTTGTGTATTTCTTCAGTATCTCTCTGGCTCCAATAA
AGTCAGAAACAAAGAACAAAAGGCTTCTAATTTCAATTCTTTGAAGAAATTATATATCTA
ATGGCTGTGGCTGCAACCATTTTCCTATTTCTTCTCCTTGCTTCTCTATTGTCTTTTGCC
AAGGAGCATATTGTTGGAGGCAGCAATGGATGGAGCCAATCCGTAGATTACTCCTCTTGG
GCATCTGGAGAAACTTTTAATGTCGGTGACACCCTTGTGTTCAACTATGATGGAAGCCAT
GGGGTGGACATAGTAACAAAAGATGGCTACGACAATTGCAACACTGCAAATGCCATCAAA
AGCTTTACTGATGGCAGCACTACCATTACCCTTGCAAGTAGTCCTGTTGAAGTCTATTTC
GTTTGTCCTAAACTTAATCACTGTGCTACTGGCATGAAATTGGCCATTAAAGTTCAAGGA
ACTTCTTCCGGCTCCGGCTCCGGCTCCGACTTGACGCCTCCGACCACTAAGCCTAATGGT
GCCTCCGGTGTTTTTGGCGCCATGAGCAAAATGGTTCTTGGATTTTCAGTTGTGTTTGGT
GCCTTGTTTGCATTCATGGTCTAGGGAAGAGGCAGTGCAATATTTGAGTTGTTATTTTCT
TTACGTTTTCCATTTATTTTGTGTTCAGTTGTCGATCGCTTTCTTTCCTTGTTAATTATG
TTAATGTATTCGTATGAGTTATGTATGGAATAAGGAATACTATCTTATTTCTTGTACCTG
TTAAGACATGATACAGCCAAGTTTGTCATTTGCAAGGATTAATTGTGCCTCACCCCATCA
GCGTTGGATTAGTTGTATTGTTTGTACTAATATTATTTGGCTCGTCTATATAGCTAATCA
AAAGTTATTTATAGATGAGATGTATTTAAATTAGGGAAAGACTGCA

>comp79061_c1_seq3 184_1473
TTGAAATTAACATAATTTTGGATCACACGTTTTATTCATTTCAACTAGTACGATTCCAAT
TCCACGTGTCATTATAACCGTTGAATATTCACCCTGCACCACTACTATACATTTCTTTTC
TTTTCTCTTTCCTCTCAATCTGCATTTCTCTACTTCTCCGATTCCGACTCAGCCGGAGAT
AAAATGACGGCGAATCTCCTTCGATTCTCCCTCGCACCTAATAACAAACTCACTTCCTGC
TTCAGTCACTCCAATGTCAACCACCAACGACGTCGTATCAACGGCCATCACTTCAATGCT
CTCATCCACGAACGACGTCGTAGCAGTCAGCTATTGCTGCGGCGCAATGCCGTATTCGCG
AAGGCTGTTGAGTTTAAAGCTCCGGCGACCGGAACTGAACAGCAGCAGCAGTTGAAGAAA
GATGAAACGACAGTACTACTCGACGTGAGCGGGATGATGTGTGGCGCGTGCGTCACACGC
GTCAAGTCAATCCTCTCCGCCGACGACCGAGTTGACTCGGCCGTGGTCAACATGTTGACC
GAGACAGCTGCGGTAAAATTGAAACCGGAGGCCGGTGAGAGTTTTGCGGCCGCTGAGGAG
TTAGCTCAGAGGCTGACCGGATGTGGTTTTCCGACAAATAAGAGGTCATCGGGGTTAGGA
GTAGATGAGAAGGTGAAGAAGTGGAAGGAAATGGTGGAGAAAAAGGAGGCATTGCTTATT
GAGAGTCGAAACAGAGTGTTTTTTGCTTGGTCTCTGGTCGCTTTATGCTGTGGAACTCAC
GCTACTCATATATTGCACTCTCTTGGCATTCATATTGGTCACGGTTCAGTGTTGGATGTA
CTGCATAATTCTTATGTCAAAGCTGGATTGGCAATAGGGGCTTTACTTGGACCTGGACGA
GACCTGCTTTTTGATGGTGTTCGGGCATTCACAAAGGGATCACCAAATATGAACTCTCTT
GTTGGCTTTGGATCGATTGCTGCTTTTGCAATTAGTTCTGTCTCACTACTTAACCCTGCC
CTTCAATGGGAAGCAACATTCTTTGATGAGCCGGTCATGCTTCTTGGGTTTGTACTACTA
GGACGTTCTTTGGAGGAAAGGGCAAGGCTGAAAGCATCTAGTGACATGAATGAGCTATTA
TCGCTTATATCCACTCAGTCTAGACTTGTGGTTACTTCTTCCGGCAGTGGTTCCTCAGCT
GATGTTGTCGGCTCTGATGCAATATGTATTGAAGTTCCAACTGATGATATTCGAGTTGGT
GATTCTTTGCTCGTTTTGCCCGGAGAAACTATACCCGTAGATGGAAGAGTCGTTGCAGGT
CGAAGTGTTGTGGATGAATCTATGCTTACTGGAGAGTCTCTTCCAGTGTTTAAAGAGAAG
GGCTTTTCTGTCTCAGCTGGTACAATAAACTGGTCCTTTAAGGATTCAGGCCTCTTCCAC
TGGTTCCAACTCAACAATTTCCAAGATTGTTAACATGGTTGAGGATGCTCAAGGCCGTGA
AGCACCAATTCAGCGACTAGCCGATACAATTGCTGGGCCATTTGTTTACAGTGTGATGAC
ATTATCAGCTGCAACTTTTGGGTTCTGGTATTATGTTGGATCGCACATCTTCCCAGATGT
TTTACTGAATGATATTGCCGGACCAGAAGGAGATCCTTTGCTTCTGAGCCTGAAACTTGC
TGTAGATGTATTGGTAGTCTCCTGCCCTTGCGCTTTAGGCCTTGCCACACCAACTGCAAT
TCTAGTTGGCACCTCACTAGGAGCAAGACAAGGGCTTCTTATTAGAGGAGGAGATGTACT
GGAGCGCTTGGCAAGCGTGGATCATGTCATGCTGGACAAGACAGGAACTCTGACTGAAGG
AAAACCTGCTGTCTCTGCTGTAGCGTCCTTGGGTCATGAGGAATTAGAGATTCTTCAAAT
AGCTGCTGCAGTGGAGAAAACAGCATCTCACCCCATTGCTCATGCTATTATAACCAAAGC
AGAATCGTTGGATTTGAGTATCCCTGTCACACGTGGACAATTAGCTGAACCTGGCTCTGG

AACCATGGCAGAAGTAAATGGGCTCTTGGTTGCAATTGGAAAGCTGAAATGGGTTCAAGA
ACGTTTCCAGCAAAAAGCAGACCTTTCTGATTTGAGGAGTCTTGAGCAATCTGTGATGCA
TAAATCATTAGAGGACAGGCAATCATCTAACCATTCAACCACAGTTGTATACGTTGGTCG
GGAGGGAGAGGGTGTGATTGGGGCTATTGCGATCTCTGATAAATTACGCGAGGATGCTGA
GTCTACCATAAGAAGGCTTCAGGACAAGGGAATTGAAACAGTACTGTTATCGGGAGACAG
GGAAGAGGCAGTTGCAACTGTAGCAAAGTCGGTTGGCATAAAAGACAAATTTGTCAATCC
ATCTTTGTCACCCCAGCAGAAATCTGCTGCCATCTCGGTTCTTCAAGCTTCAGGCCACCG
TGTTGCCATGGTTGGTGATGGCATTAATGATGCACCTTCTCTTGCTCTTGCTGATGTGGG
GATTGCTTTGCGAGTTGAAGGACAGGAAACTGCTGCTTCAAATGCAGCATCTATTATACT
CCTCGGAAATAGACTTTCACAGGTGGTGGAAGCTCTTGATCTTGCTCGGGCAACTATGGC
AAAAGTTCATCAAAATTTGTCTTGGGCGGTTGCATATAATGTTGTCGCTATACCCATTGC
AGCTGGGGTGCTACTTCCAAATTTTGATTTTGCAATGACACCATCTCTTTCAGGAGGATT
AATGGCTTTGAGCTCAATATTCGTCGTCTCCAACTCACTGCTTCTACAATTCCACGGTTC
TCAAACGAAAAGAAAAGAGAACCTTACCTATAAACGCGCTCAAACTGAGCTATAACTCTT
GCAAATTATGGAGTTGGCATGTAAGTATCTGAATAATCTTTTGTGCTACTCATTCTTCGA
CTTTGCTGTTATGTCAGCTTAATGGGGGAGAAATCTGGGAGGAGCTAGTGTCATAATATA
CTAGTATAGAGCATTGCCAAACTAGCTTCTATTTGAAAACCGGGACAATAGACAATGGTG
TGTTAACACGAAATAAACAAGTAAATAATTATTATTTAATTATGAGTTCAAATC

>comp75828_c0_seq3 249_1742
ATAAATTACCATCATATATAGAAGTTTAGGGACAGAGAAAGCTTTTTTCCACTTTAATCC
AGTGCTGACGTGTCAGCTCATCCTCCAAAATCTAAAATCTAAATCCCAGATGTTTCATGT
TCAGTAGCACTCCAACTGTTTATCCTCCCCTCTGAAATGGAATTACCAAAAATTCTCAGT
TAATTTTCTACCAAATTGATATCAATCTCCATAGCCAAAAACCTACTAGTTTATAGCTTC
ACACAATAATGCAATCTCCATCTCTAGACGCTTCCAAATCCTTTAAGCTTCAACAACAGC
AACTCCAATTCCTAGGGTTTTGCACTTACCCTTCCACTACTTCACCTTCAATCCGCAAGC
TAAATTCTCGCTATCCTTGCGTTTCCTGCAGTAGAAAATTTGATCGTAAGGAGAGAGTGA
AAGTGAAGGGGAAGGAAAATGTGTGGAGCATTGACAACGAACTTGCGAGAGAAGGAGATA
AGACGAGGACGAGGAGTAGGAGGAAAAGAGGTGGAAAAAGGATGGGAAATGTTACTAGGA
AGAGTAAAGGTGAAAAAGTTATGGTCTCTGTTGCTATGTTAATGGAAGTCGAAACCGTTT
TGCAGACTCAGGAACCTGTAATCCGACCTGCATGGAATACATTTGCTAGCAGTCTTAGTG
GGATATGGAAGGGAGTCGGAGCTGTATTTTCTCCTATCACGGCAGAAATGGAACCTATTG
AGATCGGTAACAAGAATGAACACCTCTTTGACTGCTATACTTCTTCTCGTGTTGAGGTAG
TGCCATCTTCTTCTGCAAGTCAAAAATCTCAAATCCGAAGGAAAGTGAATTGGGTAACAC
TGAATTCCCATGGTGAAGTTCCGGAACCTAATGGAGGTGATGACAGAAGCATAGAGAAGC
GCACAGGTGCAGATCTGTCTTTCCATATGCATGAAACACCCGATAGAAAATCAAGACATC
AAAAACTGCCAAAATTTGAATCTTTTGACTTTGAGAAGAGTGATATTATGGAGGAAGATA
TTATGGGAATGGAACCTGGTCTTGTTTTCTTTGAGGACGGCTCTTACTCAAGAGGTCCAG
TTGATATTCCAGTTGGTGAACTTGATGAGTCTAAGTATTACATTTCGCCAACTTTTAAGT
TTGAGCAATGTCTTGTCAAAGGTTGTCACAAGAGACTCCGCATAGTGCATACAATAGACT
TCAGTAATGGGGGTTCGGACATCCAAATATTGAGGGTTGCTGTATATGAAGAACAGTGGA
TTAGTCCTGCTAATCTATCTGACGAGAGCGACACGGAGCTGGACGTGAAGCTTTTCTCTC
AAAGACGAAGACTCCAACCATCAGAGCTGGCTGGATCGTGGAAGGTGTTTGAGATGAGCG
CAACACCTATATACAGTGAGGAGGACGTGACTCAAGAAACAGACGACGTACCCTACGTAT
ACCTATGCACGGAAAACCTGAAGAAGAGAAGCCTTCCAGAAAATCCAGCATACTTTGGAG
AAGAAGAGATGTTAGATATGCAGGATGTAACTGTTCTTTGGCTTCCAGGAGGTGTCACGA
GCTATGTTGATGTGAACAAAGATGGCATCCTCTGTATAGGAGTCGGGTGGTATTCGGACG
AGGGCGTGAATCTTGTTATGGAAAGAGATTATGAAATGGATGGGAAACTCAAAGAGGTTC
GATGGAAGTCTGAAATGAAGAGAAGGTGGACTAATCCACCACCCATGTCTCTGCATCTGT
AAGGGAAGCAGCAGCAAGGTTTCCGAAAAGGTTAACGGTTTCATTTATTCAGTCACATGA
CGGAGCATTACGAGGGTTTGAGGTTTCCTATCTTTTATATATTCATAAACAAATGAGTAT
ATCAATTCCTCTTTTATTATACTAATAAGGCGACATTTACTTTCACAAAGTGCGCCGTGT
ATTTAGGACCTACCCTCTCTATTGGCTATTGCATCCCCAACAACCGACTTTTTCGGTTCT
TGTTTTATTTCAAATGTTTTTTTATCATGATGAGTTAGTTATCCATGGTGAAAGAACCAT
TCCACAAAGTTTTGTTTGAAATTTTATCCCGTTATCAAAAACCATAGGCTATTATTTTAA
CATTTCAGTTTTGTTTGAATTTGATACACTAACATTTTATCCCGTTATCAAAAACCTTAG
ACTATTATTTTAACCTTTCAGTTTAAAAAAGCATAATTAAGAATTGTCCTTCGTTAAAGT
AACCAAGAAAAAAAAGCGACAAAACAAAACTATAGCAAAGATCAAACTCAACATTTTATT

TTATTTTTTGCTAATATGCATTCAACTTAACAAATAGTTATAAAATTTTCATGACTTAAT
AGCATTCATTGGATACTTAGTAAATAATGTCTTTTTCTCTGTCAAACTTTTTGTTGCTCA
TTTGCCTAGCGGTAAAGACAGTACTAAAGTCATTTAGCCGGTAAGTATTAGAAACTTTAA
AAGAATGAAAAGATACACATAAACCAACTGATGCTATTAATACGCCACTTTTGACCATCT
ATTAAGAGAATCTCTTTTAGTTATCTGCATTTGATTTGCTTGATCAATTGAGTATAGTAG
TAAATGTATGGACTAATTAATTGTTAGATCCAGATCTAAAATGGGGAAGCGTGATTCCTA
ACCCCAATCATTTTTTATTTTACAGAAGAACACGACTTCAGCATTTCTATGCGCGATATA
AATTCTAATTAATTTTATAGTATTATTCATTCTCGATCCAATTATATAAGAGGCTTTCGA
CTTTTTCATTTCAACAAAAAGAATTCTCTTGCATATAGATATATAGTAGGAAAAAGAAGC
ACAAGAATGAGCAAAAAGTTAGTGTTGCTCATGTTCTTAGTGGCTGCAACTTTTTGCAGC
CACCAAGCAGCGGTGGCAGAAGAGGCCACTACCGACACGGAGATAGTCTCCTCGGCCAGT
AGGTCGTGGCCATTCCCCCGCTGTTGTGATAGTGACAAACAGAAGGTGAAAAAATGCATG
ACAAATACAACTTCCATAGACGATTGCTGCCCAACATTTAAGAGCATACTTGGTCGTAAA
TGTCCCTGCTATCGTTATGCCAATTACTTAGATAATCAAGCTTTGATTACTCTTGCTAGT
TATTGTGATGTCAAGAATCCATGCATGTGTGAAAAGCAAGAAGACATGATAGAGGGCGTC
GATTCCACCACTTCCGTCACGAGGCCATGCCCTCATCCTCATCCACGACCACGTCCACGT
CCACAATGGAGCTGTTGCGCTGGTGACAAAGCAAAAATAAAGACTTGCATGACAAACACA
ACCTCCATAGATGAGTGTTGCCCAACATTCAAGAATACTATTGGCCGTAGCTGTTCCTGC
CATCGTTATGCCGAGCAGTTAGATAATCAAGGTTTAATTACTCTTGAGGCTTATTGTGAT
GTTACCAATCCATGTAAGAAAGTGCAAGTGATATAGCTTCCCCAGTAGGAGATTGGAGAA
AATAATGTTACATGTTTAATATTAGTGAGACACGAGATGAATTCTAGTCCATCTCTCTTG
AATAAAGTTTTGTAATCGTGTTTTATAAGTCATGTCATTTGGATAAAATGTACTATCTGT
ACTCTTTGGATTAGTGACAACACGATCAATTCCCTTTTCCATTTGGATAGGGTTAGGCCT
CATTTCATTAACAACCTTTACGTCCTCGTTAATTTTATGTTTTTGTTTGAGAGCACTATT
ACATCCTATCTTTTTTGTTTTCTCATTGTTTATTTTTTTCAACTTATTTCCCTTCTATCA
TACCCTTGATCGTTTCTCATCATCTTCACCATATAACTTGGTAAAAAGCTTCATACATAT
GACATGATAGCTTACAACATAATTGAT

>comp75426_c0_seq1 2_226
ACGAACCACAATATAAAGGCTTATTCCTCCTGTTAGGGTTACACAAACATCCTCTTCTTT
TGCCTTTCTCTGTATTTTCTCTTTCCTTTATCCACTGAAAGAAAAAAAAATATTGAATCT
GGGATTTGCCATCTATTCTTATTTTTATTTGATGGCTTCTTTTGGATTTTAAGGTTGAAA
GAATATTTGGGAATTTTGGTCTTGTTAAAGATAGTGCTGTGTCCATTTTCTTGAAATTTC
CTTGTCATTTCCCTCCATTTCTTCATCTTATTTTAGTTGGGCTGGCTCTAATATTCTTAA
AGTTTGAATCTTTTACCTTAGAGTTATTCAGTTTTGAAGGGTTGATCTCTTTCTGATAAT
TTCTTTTCACTTCCAATTCCTATTGAAGGTGCGGATTTTTTGGGGTTTGATCCGAATTTC
GTGTTTGTCATTTCATTTCTCCTATTTCTACTCTCAACAATTTGTGGGTTGAATTTCATA
TTGTCAAAAAAATTATAAGATTTTCTTTATTGGAACAATTTGACTGTTTCATGTGACAAA
ACAAAAACAAAAATCTATAGAAAATGCCAGTTACAGATTATCAAGGGGCATCAGCATCTT
TCTCAAATTTTGGAAGGTCACTTTTGAGTATGCGCCGTGATCAGGTGCATTCAATGGAAT
CTGCTCATGAGGCAACTAGCCAAGAGCTAGAACTTGAATCTTTTCAAAAGCAAGTCGCTG
AACGTTTCAACGACTTAGCTTCTGTTGATTCTGATCAACTGCTTTCGATCCCGTGGATTC
GAAAGCTCTTGGATGTATTCCTCTGTTGCCAGGAACAATTCAGGTCCATTGTGTTTAATA
ACACAGCTTACTTGAATAAAGCTCCAATGGACCGTTATATTACTGATTATTTCGATAGGA
GTGTGAAGGGTTTGGATGTATGTAACGCGATAAGGGATGGAATTGAGCAGATCAGGCAAT
GGCAGAAGCAGATGGAGATTATATTTTGTGCATTGGAGAATCAGAGGAGTGTTGGTGAAG
GTCAATTTCGTCGCGCCAAAAAGGCATTGGTCGATTTGACTATTGGTATGCTAGACGAAA
AGGATTCTAATCCAACTGTTAACCATAGAAACAGATCATTCGGGCGAAACAATACTCAAA
ATGATCATAAGTCTATGGGGCATTTTAGGTCTTTATCGTGGAGTGTATCGAGGAATTGGT
CTGCTGCTAAGCAGCTCCAAGCAATTGGTAATAATTTAGTTGCTCCGAGGAGTAATGAAA
TTGTTGCTAGTAATGGATTATCTTTGGCTGTTTTTACAATGAGTTATGTGTTGTATTTTG
TAATGTGGGCACTTGTGGCTGCAATTCCTTGCCAAGACCGCGGGTTGCAAACACATTTTT
ATGTGACTAGGCAATTTGTTTGGGCTGTTCCGATTTTGTCTCTCCACGAGAGGATTTTGG
AGGAATCGAAGAGGAGGGATCGTAGAAATGCTTGTGGATTATTGAAGGAGATTCAGGAGA
TTGAGAAATGCGCACACCAAATGAACGAATTGATCGATAGTGTTAACTTCCCAATCACAG
AGGAAAAAGAGGGAGAAGTAAAGGAAAGAGTTCATGAACTCGGGCTTGTCTATGATGGTT
TAAAGGGTGGATTGGATCCATTGGAGCGTCAGGTTAGAGAAGTGTTTCATAGGATCGTTC
GGAGCAGGACTGAAGGCCTTGACTCTATTGGAAGATGAAATTGTGTAGTAGAATTGGTTG

ATGATTGTCTTTTGAGAAGGCATATATTATCAGAACATGAGCATAATATGATATAGATTT
TTCCCTTTTTTTCTTTTCTTTGGTTGTGACCCTTTTTTAGATGAGAAGATGAGGGAGAAT
GGTTAATGGTGCAATGTACCAGAAAAATAAGTAGTTTAAAGTAAAAATCATGTATTTTCA
CTTAATATTATGTAAAGAAATTAGGAAAAGAGAAGGTGGATCTTTGGTCCATTTTGGTGA
TGTTCATCTTGTTTGGGATTGTATAATCACATTTCTGAGTAAGTTTGTTCAGTTTTTCTT
CTTCTTGGTACTTGCAACATTGGTATATATTGATACATCTCTTGATTAAACTGAAAGTTG
CTATTCTGTATTAAGATGTTATACTCAAA

>comp79500_c0_seq4 305_3562
CGAAAAGGGAAATTTTTTAAAAAGACTACTACACTTTAGACGTTGTACCGTGTGCAGAAG
CGCTTGTACAAACTAAGTTTCATTTTTATAAAAGGAAAGTTTAAAAATCTTCTTTTTCTT
TGAAAAACACTCCTAAAAAGGGAAGGAAGCTTCCTAACACTTTCTACGAAACACAAAAGC
ATTTTACTTTACATTGCTCTCTCTGCACTTCAGTTGTCACATTGCGTCGGTTACTCCAAC
AATCGTGAGAAAAAAAATTGTTGTGTCAAATCTGGTGTCATCTCTCCTGCGACAGTCACA
GAAGATGAGTGGCGAAGAAAATGTGAAAATGTCGCCGTATAGGCGGCATCAGAATAGTAA
TGAGGATTTGGAAGCTGGGATTAATGGCAGCAGTAGCAGGTCTATGGATTGTGGTGAAAG
TCCTTTTGATATTCCAAGGACTAAGAGTGCACCTATTGACCGCTTGAAACGCTGGAGGCA
AGCTGCGCTTGTCCTTAATGCTTCTCGTCGATTTCGATATACCTTGGACTTAAAAAAGGA
AGAAGAAAGAAAGCAATTGATTGCTAAAATAAGAACCCATGCCCAAGTTATTCGGGCAGC
TGTTCTTTTTCAAGAAGCGGGGAAAACAGTCAACGGGGATGGGGCTCTTAAGACGTTGCC
TCCAACTACTACCTCACTTGGCGAGTTTGATATCAGCCAAGAGGAGCTGGCGTATATGTC
TAGAGAGCATGACGTCCCTGCTTTGCAGCGATGTGGAGGGGTTAAAGGGGTGTCAGAGAA
GTTGAAAACAAATTTAGATAAAGGAATTGATGGAGATGAAGTAGATCTGCTGAAACGGAA
GAATGCATATGGATCCAACACATATCCTCGAAAGAAAGGGAGGAGTTTCTGGAGGTTTGT
CTGGGAAGCTTGCTGCGATACAACCCTGATTATTTTGATGGTAGCCGCAGCTGCATCATT
GGCGCTGGGCATAAAGACTGAGGGTATAAAAGAAGGATGGTACGATGGAGGAAGCATTGC
TTTGGCAGTGATTATTGTCATAGTTGTGACAGCTGTAAGTGACTATAAGCAATCACTTCA
GTTCCAAAATCTTAATGAAGAGAAGCAGAACATACAAATTGAGGTTGTTAGAGGTGGGCG
GAGAATTCCCGTTTCAATATTTGATGTAGTTGTTGGTGATGTTGTGCCTCTGAAAATTGG
TGACCAGGTACCTGCTGATGGAATATTAATCTCGGGTCACTCTCTTGCAATTGATGAATC
AAGCATGACAGGAGAGAGCAAGATTGTTCACAAGGATTCAAAATCACCTTTCCTTATGTC
TGGATGCAAAGTAGCCGATGGCTATGGAACGATGCTGGTGATAGGCGTTGGAATAAACAC
AGAATGGGGATTGCTCATGGCAAGTATTACGGAGGATAATGGAGAGGAAACACCATTGCA
GGTCCGTCTGAATGGGGTGGCAACCTTCATTGGTATAGTTGGTCTCACTGTAGCTTTAGC
CGTTCTGATTGTCCTTATGATCAGATTCTTTACTGGGCATACTTATAACCCAGACGGCAC
TGTTCAATTCAAATCTGGGAAAACAAGGGTTGGCAAGGCTGTAGATGGAGCTATAAAGAT
CTTCACTGTTGCCGTTACTATTGTGGTTGTGGCAGTGCCAGAAGGACTTCCTTTGGCAGT
TACCCTAACGCTTGCCTATTCAATGAGGAAAATGATGGCAGATAAGGCTTTGGTGAGAAG
GCTTTCTGCTTGTGAAACTATGGGCTCCGCAACTACAATTTGCAGTGATAAAACTGGAAC
CTTGACTTTGAATCAGATGACTGTAGTTGAGGCATATGTCTGCGGTAAAAAGATTGATCC
ACCTGATGATAGATCTGCATTGCCTCCAACTGTGTTGTCTCTACTCCATGAAGGAGTGGG
ACTGAATACCACTGGAAGCATTTTTGTGCCACAGGGTGGTGGTGCTGCTGAGATTTCTGG
ATCGCCGACAGAGAAAGCTATTCTCCAATGGGCTGTCAATCTTGGGATGAACTTTAATGC
AGTACGATTAGAAGCATCCATTATCCATGCTTTCCCATTCAACTCAGAGAAAAAAAGAGG
TGGCGTTGCTGTGAAGCTGCATGATTCTGAGGTTCACTTGCACTGGAAAGGGGCGGCTGA
AATTGTTCTCTCTTGTTGTACAAGTTTCATTGATGAGAATGGGTCGGTGGTTCCTCTGGG
TGATGATAAGGTGTCACGTTTCAAGCAATCCATCAATGACATGGCAGCATCAAGTTTGCG
ATGCGTTGCTATTGCCTACAGACAATATGACGTTGAGAAAGTTCCCAACGAAGAAGAAGT
AGAGCAATGGCAAATACCTGAGGGTGATCTGGTATTACTTGCCATTGTTGGCATTAAGGA
TCCATGCCGACCAGGTGTAAGAGATGCAGTTCAGTTATGTATTGATGCTGGAGTGAAGGT
AAGGATGGTCACTGGTGACAACTTACAAACAGCAAGAGCAATAGCTTTGGAATGTGGGAT
TCTTAAGTCTGATGCTGATGCAACTGAACCAAACCTGATTGAAGGGAAGAGGTTTAGAGC
TTTATTGGAGGAAGATAGGAAGGAAGTGGCAGAGAAAATATCGGTTATGGGAAGATCTTC
TCCTAATGATAAGCTCTTACTTGTTCAAGCACTAAGGAGTAAGGGCCATGTTGTAGCTGT
AACTGGTGATGGAACTAATGATGCTCCTGCACTACATGAGGCCGATATTGGTCTTGCTAT
GGGCATTCAAGGAACAGAAGTTGCCAAAGAAAGTTCAGATATCATCATCCTGGATGACAA
TTTTGCTTCTGTTGTGAAGGTTGTCCGATGGGGCAGATCCGTATATGCTAACATCCAGAA
ATTCATCCAGTTTCAGCTAACTGTTAACGTTGCTGCTCTTATAATTAATGTTGTTGCTGC

AATTTCTGCTGGTGATGTCCCATTGAATGCTGTTCAGCTACTTTGGGTAAATCTTATTAT
GGACACCTTGGGTGCGCTTGCACTGGCCACAGAACCACCAACTGATCATCTAATGCGCCG
AGCTCCTGTTGGTCGTAGGGAACCTCTCGTCACCAATATAATGTGGAGAAACCTATTAAT
ACAGGCTCTGTATCAAGTTACAGTTCTCCTAATCCTCAATTTCCGAGGTGAACAAATTCT
GCATTTGGAGCATGAGACGAGGGAGCATGCTGTTAAAGTGAAGAATACCTTGATTTTCAA
TGCTTTTGTCCTGTGTCAGGTTTTCAATGAATTCAATGCTCGGAAGCCAGATGAGATGAA
TGTATTCAAAGGAGTCCACAAAAACCGTCTGTTCATTTCAATCATCGGCTTCACATTGGT
GCTTCAGGTTATCATCATATTCTTTCTTGGAAAATTCGTTTCAACTGTGAGACTGAGTTG
GCAATTGTGGCTTGTTTCAATAGTCATTGGACTTATAAGCTGGCCCCTTGCTGCTCTTGG
GAAGTTGATACCTGTCCCTGAGAAGCCATTTGGGGACTACTTTTCCAAGAAATCACCGAG
AAATCAACAGGAGTGTTCATAGACGTTTATACATACACGAATTTAACTAACAAGCGTCTG
CGTTTTGTAAGGGTGGCGTTTCTTTTGCTACTGTACAGAAATTTGCCGCTAATTTTTTAT
TAGATCTTGGGTCATTTTGTGAATTTGGTAGAACAGTATAAATATATTTCTTGAGTGCCT
CTTTAAATTCGGATGTAGTCCCTCTGCAGCATATATTCAAATTTTGCTGTTATGTACTCC
ACTTATGTTGTCGGAGGTAAAAAAAAGCCTTTTCTGCTGTTCATATAACATGAATCTATC
ATTTATACGAGGAATTGATTACTTGGCAATTTGCTTTAAAAAAAAAAAAAAA

>comp70698_c0_seq1 752_1555
GGCAAGTTCATGACTTTTATGCTTTTCTTTTCGATTTTTCCATAATATATGCTTCTCTGC
CTCTGTTGGTACTATTTGTGTTGTAAAAAACTTACTCCGTAGTCCGTACTCTGTTGCAAT
ATATGTGGGCGACTATAGTTTTGTCCTTTCTTTGCTGTTATATTCAAACAAAAACTATAT
AAACAACAACAACAACATTAACCCATAGACTCTTTATTTTCCAATTCAAAGGAACAAATT
TTATTTTGCCTTTTCATCGCTTTGAGTGGTGGGATGAGTGGGTGTGGGGAAAAGGGGGAG
GGTTTGGAGTTGTGCTGTTGACTAATGCCTATTCTTTTATTTGTTGATATGGTTGTGGCA
TATAGTAGAAATCTGAAGATATAATTCGTGATACCCCCATCATAAGGATGTTGCTTTAGC
TTATTATAACTTTGTCTATTTTTACATTTGTAGGGGCTTTTATGAGCGTACTTCGTGGAT
TTTTTTTTTTTTTGGCTGCTTCAAACTATCATTTTCTTGGCTTTATTTATGGATAGTTGA
GGTGATATTGTGATGTTTCTTTTCTCGTGATAATGCTTATGGGCCAAGCTGATATATTAA
GCTTGTTGTTTGCGGCTGGTGTGTGGTGTGCAACATACATGTTGGTTGCAGTTGTTGGCT
TCTTTCAGATTTTCAATTTTGTCAGAACCTCTATCCTCAGTTAACCTCTCTATTTGAGCA
ACGTGTAAAAGGAACAGAAAAAGCAGTGATCATGGGCTACGCACAGTTGGTTATTGGCCC
TGCAGGCAGTGGAAAGTCCACTTACTGCTCTAGTTTGCATCAACATTGTGAGACCACGCG
GCGCACTATCCATATTGTCAACTTGGATCCTGCTGCCGAGAACTTTGACTATCCAGTGGC
TATGGACATTAGAGAACTCATCTCACTGGATGATGTTATGGAAGAACTAGGTCTCGGACC
AAATGGTGGCCTTATGTATTGCATGGAACATCTTGAAGAAAATTTGGATGAGTGGCTAAC
AGAAGAATTGGACAACTACTTGGATGATGACTACCTAGTGTTTGACTGCCCAGGCCAGAT
TGAACTTTTTTCACATGTTCCTGTGCTGAAGAACTTTGTGGAACACCTAAAGCGTAAAAA
TTTTAATGTTTGTGTTGTATATTTACTTGATTCTCAGTTTATCACAGATGTGACCAAATT
CATAAGTGGTTGCATGTCTTCACTCTCTGCTATGGTTCAACTTGAACTACCCCATGTTAA
CATTCTCTCAAAAATGGACCTTGTGACAAACAAAAAGGATGTTGAGAATTACTTGGATCC
AGAGCCTCAACTGTTACTAGCAGAGTTGAATCGGCGAATGGCTCCACAATTTCAGAAGCT
TAACAAATCTTTGATTGAAGTGGTGGACCAGTACAGCATGGTGAGCTTTCTCCCTCTTGA
CTTGAGAAAAGAGAGCAGCATACAGTACATCTTATCTCAGATTGACAACTGCATTCAGTA
TGGAGAGGATGCTGATGTAAAGGTTAAAGACTTCGACCCTGAGGAGGACGATTAATAATT
AATTCTTCTTGACTCGTTCTAAAAGGAGCACAAGGTCTTACTCAAATCAGCAAGCTCTAG
GGAGGCAATGCTGTGGTGGGCGGACGAAAATCAAGTGCCTTTTTTGCAGGACTTAAACCT
TTTTGAGTTTTACCTCTGAAATTGCCTCTATCTTCTCATTAAGTTGTAATTTTTCCCCAA
ACATATTTGATGTAAAAGACTTTCTATTCTCTGGTGTTCTCCTGGTACAAATCACTGCCG
ATGTTGAGGCATGTAAATGGACCTCAGTGGACTTCTTTCGGATATGATGTTTTATGCGAG
CTTTCGCGAAGATTTCAGATTCTTCTTTACTTTTTTGTTTAAAATTTGGTTGACATTGAA
GCTATTATGCTTTTGCCATTTCTTTTTGTGGCAGAATCGGTGTACTGATTATTTTTCTCT
CTAGTGAGTCTAATGTAATAGGGTTTATTTGCTCTTAAAGAAAAA

>comp74553_c0_seq5 265_702
AGAAACGAGCTCATTATCCTCGAGTTTCGAAGGTTACAATTGTTCCTAAAGGTTGATCCT
ACACGATTTCTCATTCCTTAAAAAATAAGGAGAGAATTTTTCAGGTTCGAGCCCTGTACG
ATGCAAACTAAGTCTGGTATTTAAGTGAAGAAGGATAGAGAGGTGGGCTCATAATCCTCA
AGTTTCAGACGGTTACAACTGGTTAGCTCAATTTTTTGGTCATTCAAAAATAAGGAGATA

ATTTTGGGTATTTTAAGTGATCAAATGGGATCATCTGGATTTTTCCTCTTATGTATTCTT
CATTCTATGGTGGCATTAACTTCTGGAGGTTTAATGATGTTTTATAGCAATGAGGTATTT
ATATTTAGCCATGGCAGAGAACGTGCTAGTAAACTTATGGGGTCAACACCACATGATCAA
TTAATAATCCAAATATCAGATTCATTTTCTGGACTGCTTTTATTTGCAATTGGATTTTTC
TTGTTCATGGTTGCATTTATAAAGGATAGAGAATTCCATGGATTCTTTGCTAAAGGATGT
GTTCTTCTTCATATTGCTATGGCTATTTGGAGAATTTACTTTGAGAGGAAACTTGAAGAA
GATCTTGGTCGTGATTGGTTGAGACTTGTTGTTGCTGACATTGCTTTAGGTCTTTCTTGG
GTTTTCTTTCTTGTTTACTCTTGGAGAGAGAAGTATGATTAGTCTTGCTAGAGGGGCGTG
CCCGCCATCCACGAGTTTCGAACGGTACGCTACTAGGTTCATTATCCATCGAGTTTTGAA
CGATGCACTACTGTGTTCATTATCCGTCGAGTTTTGGACAGTGCGTTACTGACTTTAAGG
GATTTTTCGGTTATTAAGAAAAGTTTAATTAGTCTTTGTAGAGGGTGTGCTCGTTATTCG
TCGAGTTTTGAAAGGTGCACTACTGTGTCCATTATCAACCGAGTTTTGGATGATGTGTTG
CTGACCCTCAGGGGTTTCTCTTTGGTTAGTCTTTGTAGAGGTAAATGGTGCGACATTGTC
TCCATTATTCATCGAGTTTCGAATGGTGCACTACTGTGTCCATTATGCGAAACTCGGTGC
GCTACTATGTTCATGATCCACTGAGTTTTGAAGGGTGTGTTTGTTGACCTTCAGGGATTT
CTCGGTTATTAAAAAAAAATTTGATTAGTCTTTGTTTGCGTTTTTACATTTTTATTCACAA
GACGACAACATCCGTTCTTGTTCAAGTATCAATCAACTATGTCTCAATTCCGAACTAGTT
GGATCGCTTGGGGCATCATATGGATATGATCGTGTGATAAGAAGCTACTTTGCAAGTTAA
AATTTTGAAACACAAAAAAAGATTGTGTTTCTCTGCTTCAATCTTGTACAGTACTTTCTT
CTTTCTTTTTTTGTTCTTTTTCTTTTTGTGTGGATAAAGCTTGTATAGTACTTTCATTTC
TCCTTTAATTCTCTTTGATGTAAGTTTTTGTTGATAGAAATGAGAAGTGTTTGTGATCTC
CTAATGTTGGCTTCTATTGAAATTTTACCTTCTTATTTAAAAA

>comp75715_c0_seq5 271_2055
TTTTTAAGCAATAGTATTTCTTTCTTCTACTGTTTTATAACAAAAACAAAGTTATTGGTA
GATTTCACGACGAGGCCACAAAGGTTGTTGGATCTTTCCTACAGTCTTCCCTTTGTAGCC
GTACATCACCCACTACCCTCACCAATTCTCCTCATCTTCTACCTATATACCTCAACCCCT
CCTTTCTCTTCATCACCTATATCCATTTTCACAAACCCTTCTCCTAACACCCTCCATTTC
ATTATACATTGTTTCTTTCTCAGTTAAAAAATGAAGGTAATTGACAAAATTCACGAATCC
GCCAAAACTGATAGAGTAGTTTTCTCCTTTGAATTCTTTCCCCCAAAAACTGAAGATGGG
GTTGATAATTTGTTCGAAAGAATGGAAAGAATGGTGTCACATAATCCCTCTTTCTGTGAT
ATTACATGGGGTGCAGGTGGATCAACAGCAGATCTGACACTTGAGATTGCAAACAGAATG
CAGAACATGGTTTGTGTTGAAACCATGATGCATTTGACTTGTACAAATATGCCTGTTGAG
AAAATTGATCATGCCCTTGAAACTGTCAAGTCTAATGGGATTCAGAATGTTCTTGCTCTT
CGCGGTGATCCTCCTCATGGACAAGATAAGTTTGTCCAAGTTGAAGGTGGTTTTGCCTGT
GCCTTAGATCTGGTAAAGCACATGCGGGCCAAGTACGGGGACTACTTCGGGATAACTGTC
GCAGGTTACCCAGAGGCACATCCTGATGTTATACCTGCTAATGGAATAGCTACACAAGAG
ACATATGAAAATGACCTTGCCTATCTCAAAAAGAAGGTTGATGCTGGTGCTGATCTCATT
GTAACTCAACTCTTCTATGATACTGATATTTTCCTCAAGTTTGTCAATGATTGTCGTCAA
ATTGGGATAACTTGTCCTATTGTCCCGGGAATCATGCCCATCAACAATTATAAGGGCTTC
TTGCGCATGACTGGTTTTTGCAAAACCAAGATCCCAGCAGAGATTACAGCTGCCTTGGAA
CCAATCAAGGATAACGAAGAAGCTGTTAAAGCTTACGGAATTCACCAAGCGACTGAAATG
TGCAAGAAGATTTTGGCCACTGGCATTAAGACCTTGCATCTTTACACATTAAACATGGAG
AAATCAGCACTAGCTATTTTGATGAATCTTGGATTGATAGAAGAGTCCAAAATTTCAAGG
CCATTACCTTGGAGACGTCCTACAAATGTTTTCCGTGTTAAAGAAGATGTCCGTCCTATA
TTCTGGGCCAACCGTCCGAAGAGCTACATTTCAAGGACCATTGGTTGGGATGAGTATCCA
CATGGCCGATGGGGCAATGCTCAAAATCCATCATATGGAGCACTTAGTGATTATCAGTTC
ATGAGGGCACGTTCACGTGATAAGAAACTTCAAGAGGAATGGGCTGTTGCTCTCAACAGT
GTGGAAGACATCTATGAGAGATTTATGAACTACTGTCTTGGGAAGCTGAGAAGTTGTCCT
TGGTCTGAGTTAGATGGGCTTCAGCCAGAGACGAAGATCATCGATGAACATTTGGGTAAC
ATAAACACGAAAGGTTTCTTGACTATTAACAGCCAACCAGCAGTTAATGGAGCAAAATCT
GATGCTCCTTCTATTGGATGGGGTGGTCCTGGTGGATATGTTTATCAAAAGGCATACTTA
GAGTTCTTTTGCTCTCACGAGAAGTTGAATGCTCTTGTTGAGAAATGCAAGGCTTTCCCC
TTCCTTACCTATATGGCTGTGAACAAGGAAGGAAACTGGATTTCCAACGCCAACCAAACC
AATGTCAACGCGGTGACATGGGGAGTTTTCCCAGGTAAGGAGATTGTACAACCAACTGTG
GTTGATCCTGCCAGCTTCATGGTATGGAAGGATGAGGCGTTTGAAATCTGGTCAAGAGGC
TGGGCTCAATTATACCAAGAGAGTGATCCATCAAGAAAATTGCTTGAACAAGTTCAGAAC
AGTTACTTCTTGGTTAGCCTTGTCGACAACGATTACATCAATGGAGATTTGTTTGCCATC

TTCAAGGATATCTGAATTTAGCAGCCATGCAGAGCTCCTAATTATAAGTTTCTTCTTTCG
ATTTGCGTGCAAAAGTTATGATTGTTGTTGTTTTATGTTCCTGTAACAGGAATAATTCAG
TGGGAATCTGTTCCCTTCATTTTTTAACTGCAAATTTGATATACATTGTTCCTCAGTCTT
TTGCAACAATTATGAGCATCTGTATACAATTTTTTTAATTTAGCTGTCTAAATATATAGA
CATGGCATTATTCTTCCAAAAAAAAAAAA

>comp80883_c0_seq1 2_310
TACTGGATTCAAGTGTGGTATCAATTATCAGCCACCGACTGTTGTTCCTGGAGGTGATCT
GGCCAAGGTGCAAAGGGCTGTATGTATGATTTCCAACTCAACAAGTGTTGCGGAGGTCTT
TTCACGCATTGACCACAAGTTCGATCTAATGTATGCCAAGCGTGCTTTCGTGCACTGGTA
TGTTGGTGAGGGTATGGAAGAAGGTGAGTTCAGTGAAGCACGTGAAGATCTGGCTGCTCT
GGAAAAGGATTACGAGGAAGTTGGTGCTGAATTGGAGGAAGGAGAAGACGATGATCATGA
GGAATACTAAATACCTCATGCATTGCTTTGTTTATTGGATTGTAATTTTCTTTTGGTTGT
TCATCAGTTGTGGCATTTTAGATATTCCAGTACTAGTTTGTATTTTGATGGTGTCTTGTA
TGCTGAAATGACTTCTGCTTTATGAAAGTTCAAACATGCCTGTTTTGTGTGAAAAAAAAA
AAAAAAAAAAAA

>comp75221_c0_seq2 455_1678
GAGTGACTACACTATAGGTCTTCCCTTGATGTGCTAAAGAAAAAAACAAAAGTCGAAGAC
TAAACAAGCTCTCTCCTCTATAGCTAAGACAATTCACAATTCATTTCATTCATTACTCTG
TGTGTGTGTGTGTGTTTTTCTCGTGTTCACTTCTCTTTCTTCGTCCTTTCTGTGCTACTG
AGAATACCCTTTCTCTTTCTCTCTAACTTTATTAGCTCTTTCTCTCTGTACGCCGTCGTT
TTACCGCCGGTGATGTTTATTTAACCGGAGGATTTGGTCGGTTGGAAAGTTCGATCGGCT
GTAGAGTACAATTAGCACACGGCACCAGCTTAATCACAAAGTCAAGCTCTTTTGATCGCG
GGCTGGAGAGGTTCAGTTTGATTGTAGAAACCCACATGCAGTAGAAATAAGTCAGATTTG
AAGCTTTTGTGCTGTAGCTGCTTTGTCAGAAAAAAATGTTGGAGGCTCCAAAATTTGTTGG
ACTTATAGACTTGAATCAAAACCTGTCACAAAATTTCTACCATAAGCTTGGTGAGGGGTC
AAACATGTCAATTGAGAGTTATGGGAGCTTGCAGTTGAGCAATGGTGGAGGTTCTGTTGC
AATGTCGATGGATAACAGCAGTGTCGGGTCAAATGATTCTCACACTCGTATTTTAAACCA
CCAGGGCCTCAATCGCGTCCACAATAACTATTCTGTTGCAGCTAGCGTAAACAGGGGGAG
AGTTTCTAACGGGTTGAGCAATGATGCCCTCGCTCAAGCCTTGATTGATCCTCAATTCCC
CACCATTGGGCTTGAGAATTATGATGAGTGGACAATTGACTTGAGGAAGCTTAACATGGG
ACCAGCTTTTGCTCAAGGAGCTTTTGGAAAACTCTACAAGGGAACTTATAATGGTGAGGA
TGTTGCTATCAAGCTTTTGGAGAGGCCAGAACATGATCTTGAGAGGGCTCACTTGATGGA
GCAACAGTTTCAGCAGGAAGTCATGATGTTGGCAAGGTTGAAACATCCAAACATAGTTCG
GTTTATCGGTGCGTGCCGTAAACCCATGGTGTGGTGTATTGTCACTGAATATGCAAAAGG
AGGATCAGTTCGTCAGTTTCTCACTAAGCGAAAAAATCAATCTGTGCCCTTGAAGTTAGC
AGTAAAGCAGGCCTTGGATGTGGCAAGGGGTATGGAATATGTGCATGGCCTGAATCTGAT
ACATCGTGACCTGAAATCTGACAACCTACTGATTGCTGCTGATAAATCAATCAAGATTGC
GGACTTTGGGGTTGCTCGTATTGAGGTGCAGACTGAAGGAATGACACCGGAGACTGGAAC
ATACCGCTGGATGGCTCCGGAGATGATCCAGCACCGACCATACACCCAAAAAGTTGATGT
TTATAGTTTTGGCATTGTTCTCTGGGAGCTCATAACAGGGATGCTTCCTTTCCAGAACAT
GACTGCTGTACAGGCAGCTTTTGCTGTTGTCAACAAAGGCGTCCGCCCAACGATCCCCAA
TGATTGTTTGCCTGTGTTATCTGAGATCATGACCCGCTGCTGGGATGCTGACCCTGATAA
TAGGCCACCTTTCTCTCAGGTGGTCGGAATGCTTGAAGCAGCAGAGACAGAAATCATGAC
TACTGTCAGAAAGGCCCGTTTCAGGTGCTGCATCAGTCAACCTATGACTACAGATTGATA
TGGAAAGAGGAAATAGTAGGCAGTAAGAGCAAGAGTAAAGAATTTGGGAAGGCAAAACAA
AACTGATACCAAAGTTTTGCCATTTGTATCTACGAGGCATAATTCGTCATTCAGATAGAA
AAAAAGGGAGAAAGTTATGATAAAAACTCTCTTTGTTGTATCTTCTTTTGTTTTTCCTTT
TCTTCTTGGATTGAAGATTCTGATTAGTGATAATAGAAGTATGTGTAATTATACATGTCC
GTTTCTGGCTGTATTTAGATCCTACCTTTTATTTTACTTTGATCAATTTTTCAGGTGCTG
AAGATGTTATATGCTGATCCACCTCACACCCCCTACCCTTTTGTACTTCTGATGATGCTT
GTTTTATTTGTGCAGAAAATGTG

>comp84169_c0_seq1 105_1127
CTTAATTACCCATTCACTTTCCCCTTTATAAATGAAACAGAGAAAAAAAATAAAACAATC
TCACTTCTTATCCTACTACAGCCTTTGAACACTTAAAAACTGCCATGGCAGAGCACAAGA
TTATAGCAGAAAACACCATGGAAGATGCTTACAAGTTTCTTAACATTATCCAAAATCCAG

ATGGCTCTCTAACTAGAAAATACCCATTTCCTAATGTGCCTTCAAATCCAGAAAATGATC
CAAATTCACAACTTATTTCACTCTCTAAAGACATTCCTCTTAACCCCACCAACAACACCT
TCATCCGCCTCTTTCGGCCCGTTAATCCATCGCCTAACACTAAATTACCGTTGATCATCT
ACTTCCACGGTGGTGGATTTATACTCTTTAGTGCAAGTTCTATTTTTTTCCATGAATCTT
GTAATGCTATGGCAGCTCAGTTCCCTGCATTGGTTGCTTCAGTTGAGTATCGGCTTGCCC
CCGAACACCGCCTTCCGGCCGCCTATGACGACGCCGTGGATGCCATTAAATGGGCTATAG
ATCAAGCTCTTGGTACTGGTGATCATGTTGATCCATGGTTGAAAGAGTGTGTTGATTTTT
CAAAAATTTTCTTGATGGGTAGTAGTGCAGGAGGAAATATCGTGTACCATGCAGGTCTAC
GTGCACTCGATCTTGATCTAGATCCGATCAAAATCGTAGGGTTGATAATCAATCAGGCCT
ACTTTGGTGGAGTTGAGAGAACAGAATCTGAACTTAAGTTTGTTAATGATAAAGTTGTGC
CGTTGCATGCAAATGATCTGATGTGGTCGCTTGCATTGCCTAGCGGTGCGGATCGCGATC
ACGAATATTCTAATCCGTTTAGTAGTGAGAATAAGTTGAAGGAGATGATCCAACGGCTGC
CGAGGTGTATGATCCGAGGGTATGCAGGGGATCCACTGGTTGATCGACAGAAGAGGTTTG
CGAAGATGGTGGAGTTACATGGTGTGCACGTGACTTGTCAGTTTCTTGAAACTGGACATC
ATGCTGTTGAGATATTTGATCCTAAGAGTGCTCAAGATTTGTATGATTCTATCAAGGATT
TCATCAAATCTACTTGTGATGGAAATGTGGGCAAATCAGCTATGTGATGCAGTGTGATAA
GTGAAAATTTGTCTAAAGTCAACTGGGATCATTAATGCTTTCGCTTCTTTATTTATTGCT
TTTACAAGTGCAATGTATGTTAGTCGGACTTAATAGCAAGGGGTCGTTTGATTGAAGGTA
TTAGAACAAATCATGCAAGCATTAATTCTAAATATTATTAATATCTTATTTGGTATATTT
TTTTAATTTGTGAATAACTAATATATATATATATTAGTTA

>comp66224_c0_seq2 136_780
GTACTTTTAACATTATGGTGATGATATGATGATGATAATGGTACCTCTCTTATTCTAATT
AATATTATTGTTTTGATTTAGATTTGGATCTTTTGCAATTGATAGGTCAATATTGATACG
TAAAGAGAAGTGGCAATGGCATATAAGGTGGATCATGAATATGATTACTTATTCAAGATT
GTTTTGATTGGAGATTCAGGTGTTGGTAAATCTAATATACTTTCTAGATTTACCCGAAAT
GAATTTTGTTTGGAATCTAAGTCTACCATTGGCGTCGAATTTGCAACAAGGACCCTTCAG
GTAGAGGGCAAGACAGTAAAGGCACAAATATGGGACACTGCTGGTCAAGAAAGGTATAGA
GCAATAACAAGTGCATATTACAGAGGAGCCGTTGGTGCACTTTTGGTCTACGACATAACC
AAAAGACAAACCTTCGAAAACGTGAGCCGTTGGCTTCGTGAACTTAGGGACCATGCCGAT
TCCAATATTGTTATCATGTTGGCCGGAAATAAGTCAGATTTGAACCATCTCCGAGCAGTC
CCTGACCAGGACGCTAGGATCTTGGCCGAGAAAGAAGGGCTCTCGTTCATCGAGACGTCT
GCTCTCGAGGCGTATAACGTTGAGAAAGCGTTTCAGACTATATTGTTGGACATTTATCAG
ATCATAAGTAGGAAGGCTCTGGCTGCTCAAGAAGCAGCTGCTATTCCTGGTCAAGGCACT
GCTATTAATGTTGGAGAGTATTCTGGTAACAATAACAAACGACCATGTTGTTCTAATTAA
CTGAGAGGGATTAAAAAGGAAAGATGTAAATGTTTATGCCAGATTATCATACTTTTGTGT
CTAGCAGTTTTATCAGACAATGCTATATAGAGATTATAGGTTACTTAAAGATGTTGTAAT
CTATTACAAGTGAGGTGAAAATGTTGTACTAATACTTTTAATTTCTTGGCCTTCTTTTGT
TGAAGGAAATATGATTTTGCAAAAAAA

>comp74264_c0_seq1 147_1052
CGCCTCATTAGGATCGGTCTACTAGGGCTTTTGTTTAGGTATTTTAGCTTTGCCTCGCAC
AAGTATATGTTCTCTTCACAAACAGCTTTTCCTCATTTCCCCACGCCCTTAACCCCCCGC
CGCCGGAGGCTAATCCAGGAGGAACAATGGCATTCATCAAAGTCCAGAAGACTAGGGCTT
ACTTTAAGCGTTTCCAGGTTAAATTCAAGAGAAGGAGAGAGGGGAAGACTGACTACAGAG
CAAGGAATCGCCTGATTAATCAGGACAAAAACAAGTACAACACTCCAAAATATCGTTTCG
TTGTCCGATTTACTAACAAGGACATAATTGCACAAATTGTGTCGGCTAGTATTGCTGGTG
ACATGATTCTTGCCTCTGCTTATGCTAACGAGCTGCCTCGTTATGGCCTTGAAGTTGGAC
TGACAAATTATGCTGCTGCCTACTGTACTGGACTTCTTTTGGCACGACGAGTTCTCAAAA
AGCTTGAAATGGACGAGGAGTATCAAGGGAACCTTGAGGCCAATGGGGAGGATTACTCTG
TTGAACCTGCTGAAAGCAGGAGGCCTTTCCGTGCTCTCTTGGATGTTGGCCTTATAAGAA
CTACCACAGGCAATCGTGTTTTTGGTGCTCTCAAGGGTGCATTGGATGGTGGGATTGATA
TCCCTCATAGCGAGAAAAGGTTTGCTGGATTCGGCAAGGATTCCAAGCAACTTGATGCAG
ATGTTCACCGCAAGTATATCTACGGTGGCCACGTTTCTGCATATATGAAAACATTGATGG
AAGATGAACCTGAGAAATATCAGTCACACTTTAGCGAGTACATCAAGACGGGTCTTGAGG
CTGATGATCTTGAGGAGATGTACAAGAAGGTTCATGCTGCCATACGTGCAGATCCAAGCT
CAAAGAAGTCTGACAAGCCACCTCCTAAGCAGCACAAGAGGTACAACCTTAAGAAGCTAA
CTTATGACGAGAGGAAAGCTAGGTTGATCGAAAGACTGAATGCTTTGAATGCTGCTGCTG

GAAATGATGATGATGAGGAAGACGATAATTAGTTAACTACATAACAAGAATTTTGGATTC
TTGTCGCTTATGGGTTTTTCTATTATATTTTTCTTTTCTTGTTTTCTACTTAGTAGAAAT
CTCAGTTTTGCATGCTTAGTAGAACGAGACTTCACCAAAGTGACGATGTGTGAGATTATA
TTTGCTTCATGCATTTATTCCGTTGAGTTTTAACTTCTTTACATTCACACATTAGAAGTT
TTACACTATCGAGTCAACGATAAGATAAACTATGAGTACACATGATAGT

>comp79937_c1_seq1 312_1058
AAATAGTTGAGGTGGTGGAAGTTGGCTCGAACATGACGTGTAAAACAATTGTGAAAAAAG
AAAATGTGGAAACAAAAAAACTAGGACGCCGGCGTTGATGGTTAGGTTTAAGAAAGAAAA
AAGATCTGAGGGGTAATTAGGTAAACCGACTAAAGTGCGGCAGTAAGAGAAGCCCTAGCT
CCACTCATCCTTCGCCTATAAATAGACCCCAATAACCCCCTGAAACCCTAATCCTCTCAT
ATATTTTCGTTTCTCTCTGTGCTGTGCGGCAACAGATCTATATTTGCTTTCAAGTCTTTT
TTTCAATCAACATGGGTAAAGAGAAATTTCACATCAACATTGTGGTCATTGGCCACGTCG
ACTCTGGTAAGTCGACTACCACTGGTCACTTGATCTACAAGCTTGGTGGTATTGACAAGC
GTGTTATTGAGAGGTTCGAGAAGGAAGCTGCTGAGATGAACAAGAGGTCATTCAAGTATG
CCTGGGTGCTTGACAAGCTTAAGGCTGAACGTGAGCGTGGTATCACCATTGATATTGCCT
TGTGGAAGTTTGAGACCACCAAGTACTACTGCACTGTGATTGATGCCCCCGGACACAGGG
ACTTTATCAAGAACATGATCACTGGTACTTCCCAGGCTGATTGTGCTGTTCTTATTATTG
ACTCCACCACTGGTGGTTTTGAAGCTGGTATTTCCAAGGATGGTCAGACCCGTGAGCACG
CATTGCTTGCTTTCACTCTTGGTGTCAAGCAAATGATTTGCTGCTGCAACAAGATGGATG
CTACCACCCCCAAGTACTCCAAGGCTAGGTACGATGAAATCGTAAAGGAGGTTTCTTCCT
ACCTCAAGAAGGTAGGATACAACCCTGACAAAATCCCCTTTGTCCCCATCTCTGGTTTCG
AGGGTGATAACATGATCGAAAGATCAACCAACCTTGACTGGTACAAGGGCCCAACTCTCC
TTGAGGCTCTTGACCAGATCAATGAGCCCAAGAGGCCCTCAGACAAGCCCCTCAGGCTCC
CACTTCAGGATGTGTACAAGATTGGTGGTATTGGTA

>comp78325_c0_seq3 335_2797
TTTCTGTTTTGGCTTCTTTCCTACTTTCTCTCTCCCCTTTATGGAATACTCAGTTTCAGT
TTCTCTTTGCCGTCCACTGCCTCTGTGAAATCTCCGGCGGTATTCCATTTCCTCAAGTTT
TGTTTTGCAGGATGATTTCATAAAACAGTTCCCCAACTGAAGCAGTTATTATTACACCTT
GTATAAGTCCTGCCCTTTTTTTCCTTATTTTTTTCTTTTCTATGAGACTACTTTGAGGTT
ATCCCTGCAAAAAAAAATACTGTCTACCATTTTATCCAAATAAAGTAAAATAAAGGCAGA
AAATTTGGGTTTTTACCAAGTCTGTCGCAGCGGCATGGAGGGTAGGCAAAGAAGATCAAT
TTCACAAATAGAAGATTTTCAGATTGCCAATTCATGGTTTCCAACAACTCCAGCAAAACC
AAGTTTGTCACCAATCTGCGGGAATAGGCAACAAAATCAGCAAGCTCAAGAAAATGGGTT
GGAGTTGAAAAGAATTTCACAAGGGCAAGGTCTAAGCCAAGTGATCCAGCCAGAATTGAG
GAGTTTCTTGCAAGAACCTGAAGGTCAACATGTGGCAGCATGTCGTGGTTCGACAAATTC
AGTAACAGAGTATTTTGACACTTGGGAAGCAGCATCAGGCACAAAATCCAAAATATATGG
AGACAACAATATGTATAATAACTTCTCTACTGATGATGTAGACAAATGGAGCAATGTGTC
CTTCGGACATCTCTTGGCACTAGCACATGCTGCTGGATCCACAGCCGCCGCTGAAAATAC
AGAAACTAATTTTACCATGAGCGGTTCTTTCAATCCCCTTGCCAGTTCGCAGAATGCAGA
TGGAGGCTCTATTTGCTCGAGATTCCCTTTTAACCTGAATTCACCAGCAGATGAAGTCTT
GAGAAGCAACAATGCTGTCCAGTTTGAACCAATAACACCAGACCAGAGCAAGAACAAAGG
GGGCCGAGCATCTGATGAGTTAAATCTAGATATTAACGAGACACCACAACTAAGCCACAT
GCGGTCATGTGAAGACACATTAAAGAGAGCACAAGCAAATGATCCTCAACAGAAGAGAGA
GCAGTCAGGGCTGGTGTTGAACATATCAGAATTGCAGGGGAGTCACAAGCCTGACAAGGC
AGACGAACAGGATGCTGAACAGAATAATACACCACAGCAGAAACAAAGAAGGAAGAAGCA
CCGGCCTAAAGTGGTAATTGAAGGTCAGACTAAAAGGACTCCTAAACCAAAAAAAATCCA
GCAGCATAGCTCAAAGGAAACAACAGGAGAAAAAAGGAAATATATCCGAAGGAACAAGGT
TGAGGAACCTCCAGGCACACCCTCAGACAAGGTAGACGGTATGACTTGTCCCCAGAGCCA
ACTTCCTAGTTCCAGAGAAATCCAAAGATCAAAGAGGAGGTACGTCAGAAGAAATAAAGT
TAACAAGCCCGCACCAAATCCTGCCGAAGATGAAACAATTGATCCACCAAATATTTCTCG
TCCAAGAAGATCCTGCAGGAGATCACTGAATTTTGACTCAGAAAGCAGATTAAGTAATGA
AAGCTCTTCACGCTGGCACTCTTCAACTGGGGAGGACTTCCATGAGAATCAATCCCGTTC
TAGTGTACATCATGGAAAGGATATTGAGGTCACAACAGGAAAGAAAGAAGGCACAGTTTA
TAACATCGCATGTTCCAGAGGAAAGTGTAAAATCATTTTCTCGGATGAAACTCATGATAA
ACAGGCAAGCATTTTGGAAATGACACCAAAGAGTCTGAATGGTTCCAACTGCAGCAGCAG
TGCATGCTTGATCCAGGAAGCACCAGAAAGAGCCTTGAAAAGACGACGTTCTTCTATAAC

TAATGAAGCAGAACTTTACAGCACAAATGTCAAGGGAGCCTACTTCAACTCCATGCAGGC
ATATCAAGCAATCCTCCCAGCAAATGAGCCTTATGCTCACCGTACACAAGGGATGCATTT
TCCTACAATTTACAAGAAAAAGAGAACAGAAAAGGGCCATCCCACAGCAACATCTTACGC
TAAGCCTTTTACATGTGAAACTAACTATCTGTCTTTGTCTCAATGCAACATTGGTCTTTC
CCAAGCCAGTACGTCTGCAAATGACAAAGCTAATAATAGAATGTGGAATCCCGAACTTGT
ACCAGCATTTATAGAAGCAGAGGGGTTAAGAAGAAAAAGATCAAAAGGCATAAGTAAAGT
ACGTGACTTAGCATCACTGCTTGAAATTTGTAAACACTTTCCTACAACTCCTGCCAAAGA
AGCATCGATATCTGAATTTGGGGAAAGATATTCAGATCAGCCTAATACATGCATGGAAGC
CCTAGTTGCTGACACCTGTGCAATAATGAAAACAAAAAAGCGGTCAAAGAGAAGCATCCT
AGTCAGTTCCACAGCATCCTATATGTATGCTCAACAACAATTCACAACAAATGCAAGGGG
TTTCCTACCAGCTATAACATGGAGATCCCCAGTTGACGAAATTGCCGAACGCTTGCAGTA
TCTTGATTTAAATAGAGAAAGCATCCAAGATCAATATCAATATGGGGAAATCACCTACCA
GAATAAGTTTCAAGCAGAAAATGCCCTTGTTATTTATCGAAGAGATGGGAGTATTGTCCC
TTTTGCAGGATCATTCATTAGAAGACGAAAACCGCGGCCCAAGGTTGACCTTGATGATGA
GACCACTAGGGTATGGAAGCTTCTGCTGCAAGATATAAATAGTGAAGGCATTGATGGTAC
AGATGAAGATAAGGCAAAATGGTGGGAAGAAGAA

>comp75577_c0_seq17 1347_1982
CGCACTCCTGTAAGGTAAGTCTAATACGAGTTATAAAACGCATTCATCTAAAGAGTTATA
AATTGCGTTTTATTGAATAAAACGGACTCGAACTTGTGGCCGGTATTTGCAAACCAACAC
CGGCGACAACTCCAGAGACAGGTCGCGGGGTTTTTGCGCTCAGCAGCTTTGTTCCTCGTT
GTCGTTTTTTGTCGTCATGTCAGTAGTAATCAAACTTTCCCGATCCAACCGCATCTATCG
CCCTAATGAAGCAGTGCAAGGGAAAATCGTAACGAAGCTCTCTTCTTCGATTTCTCACCA
AGGCATTCGACTCAATGTCAACGCTTCAGTCAACTTGCAGGTTAGAGGAGGATCAGCGGG
AGTTATTGAATCTATTTATGGCGTTATTAAGCCTATTCCAATACTGAATAAGACTGTTGA
GGTCCTAAAATCTGGTAAGATCAGCTCAGGTACTACAGAGGTATGTTTTTAGAATTGAGT
GCTGGTAAATTTTGCTGTCTTATGTTGTTTCAATTAATGAGTTTTTTCCTCCCTGCAAAA
TATGTACTTCCTATATGGTAGTACATGCCAAGGTTTGGGTTTTCCCCTCGAAGAAAAATG
CTGATATGAGAACTAGAAGAAGAAGGGAGTTATGCAAAAAAATAGAAAAATCAGCATTCG
CACGCGGTTAAGTGAAAATGCTTGTGTGGTTCTTGGTAGTGATCTAACACTCCATAACAC
GTTTTCTATCTCTCAAGTAGTTCTCCTTTTTAAGAATAAACTGCCGATATCTGGGTCAAC
TGGACGATAAAGGTTCCTCTATTCGTCCCTTACCCTAACATTTTGTCCAAAAAAGAAACA
AGAATCCCCTTAATTGTTCCTTATCAGAAAAAGAATCACCTTAAATTGTTCCGGTGGTCA
GTTATGTCAGAAAAGAAGAAGTAGTTATAAACAGCAGTAGAGAAACAAAAATCCCCCTGG
ACTCGGGAGAAATCAAATCAATTCCTCCTCTACCAGCTAAACAAGGCAGTAGTATTACGC
GTCATTGCTCATACTTATCAGCAAATGACAGTCTTAGGGAGATTTGGGCTCTGAAAGTGA
CATAATGATTCAAGGAAGTCTGTGAAAGCGAGGAGGAATCTTCAGAATATTACACTGGAG
GCACAAGTTTAAAGATTAGGTCCAGTGAAAAAATGCTCGATCAGTGAGATGCAACTCTTT
CATTGTCCTATGTTCTTATATGGCTCTATATTTGGTTATAAACTATTCTTCTTGAGCCAT
TCATTGTGGTTGTTTTTTCCTTTTGCTTACTCCTTTCGAATCCCTGATTATTCTTTTTGT
TGCCTCCATAGATTCCGTTTTTTTTCATGTTGAAAGATCCAGGAGAAGAACCTTTGGAAA
AACTTTATGAAACATATCATGGTGGGGACATTAGCATTCAGTATCTGGCATCTGTAGACA
TTTCGAGAGGATACTTACACAAGTCTCTATCTGCCACAGTGGAGTTCATAATTGAAAGTG
AAAAGGAGAATCTCCCAGAGAAACCTATTTCTCCTGAAGTGGTCATGTTTTATATCACCC
AAGACACACAGAGGCATTCACTACTTCCCGAGCTAAAATCAGGGGGATTCAGGGTAAGCG
GTAAAATATGCACATTATGTTCTTTGTCGGATCCTATTGAAGGTGAGCTAACTGTTGAAG
CATCTGCTGTTCCTATCCAATCCATTGACATGCACTTGCTGCGAGTGGAGTCAATTTTGG
TCGGTGAAAAAATAGCAACTGAATCTTCTCTGATACAGACAACTCAGATAGCTGATGGGA
ATGTATGTCGAGGCATGGCTCTTCCCATCTACAGTATTCTTCCCCGCCTTTTGACTTGTC
CGTCAATCTTTGCTGGGTATGTGTTCGATCGAATTTACATCTTTCTTGTTTACAAATTCC
CAAGATTCTTGTCTGTTAGGACCTTTGTGGCCTTTCAAAAGTATCATCTCGTTGGTACAT
AATACATAAATGTAATGGAGAGCATGATGGATTCCAAATCATACTTGCAAATGAGTCAGT
TCCAGATTCTAGACCATACCGAGGACGAGTGCGTTCATTCATTTATGCATGCTTCCCTTG
CTCATATATTACCAACTGCAGGCCTCTATCTCATGTTACCCAAAGCAAATTTACTTGAAA
CTTTCTGATTATTAGATATCAACAAAAAGAGAGTTGGCGTATTTCAATCAATAGAAATTG
CTCCTTGTGTTATTATTTGACGATGGTTCGTGTGAGGAACGCATGGAAAGTGATATTTTC
ATGGGGGTATTTGTGAAACTTTCAACAGAAATCTATGACCGAAGGCTAGGAATGTTTTCC
TTTTGGTTTGCTTGATATGCTTGCGTTATCATTAGATGTGGATTATCTTTCTGCTCTTAC

CATTACGCTAGTTGCCAATTGGCTGAATAATCATCTTTGCAGTCCATTTTCCATAGAGTT
CAAAGTTACCATTGTAATAACCTTTCAGTCAGAGCAATCGAAGTTGCATCCAAAATCCAG
TTTCAAAACTCTTAGATCATGGCACGCAGCAGAAAGTGTTCCCCTTGAACTGATTCGGAC
AAAGTGAGATGCTGGGCAGGTAATTATTTGAAAGTTCTGTTGAATTGCTTATTCTAAATT
AGACATCTTGATACAACTTTTATTAGTCCTGCACATATTCGATATTAGCACCTTGCAATG
GTTTTAATGATATAGAAACATAGCCTTCTTAAATATGTTCCGGGGATGCTGAAGTTTTTG
CGTATGTAAATGGGTGAAAACTCGCTTACTGATATTGGCTTAGGCGCCCAGAGGATAGAA
ATTCTGTGTGCGATCTTTCACCTGGAAGATGACTATCAAGTTCATTTGCTCAATGTTGTG
GCCACGAAACTATGTTCCTGTTCTACAAGTGCAAAATTTCCAGGAGATACTGCAAGCGAG
AATTGCACTTCTCGATTGACAAGTGGGGATGCGTAGATATGGGAGGTGAAGATGAGTTTG
TAGAAAGTAAAAGAACTTCCTACTTTTTGTTTCTTTATGTGCACTAGCTGTTACTACTCT
ACACTGGATAAAAGATTGCTGTACACTGGGGGGCAAAATTGCCAACTGGTTTCTTGCAAG
AGAGAAGCAGCTTCACAGAGTTCTGAACCTCATCTGTATGTCTTCCATATTAGGTTATAT
AAAGTGTAGATACATAATTCTTCTGAGGTATGAATTATGATACGTACCATCTTTCTCATC
GATCTAATTCAGTGTAAACATATCACGTGAGCAGTACTGCAATTGGACAGGTGCAGATAG
GTCTTCTGTTAATCAATGACCAGAAAATCTTGTGACAGACTTATTTACATCTTAATTTCT
CTAAATTTATTTATTTGTAT

>comp79768_c0_seq4 287_3412
GCGAAATCCGACGCAAAACGAATCCTTGTCCGACCCGACCCAGGCGAGCAGTGTAGTCTT
TGAGAAAGAGATGATGGTCGGATAAGGATAGAAAGCCCCACCTTTCGTTTCCCTCTTTTT
TATTAGCCATATACTCATTCATTCACTCTCACACATTGAACTCAGAAACTGGGAGAGAGA
GAGAGATATACCTCTATCACCTCAGCAGAAACAACTTCAAAGCTGGGTGCGAAACAGATG
ATCCAATCCTTGTAGTTCACGAATCTTTGACACATCTCAAATCAACATGGCTCCAATGGT
TCCAATTGCAACTAGCAATGTTTCAGTTACTCCAGGAGCTGTCTTTATCACGAGAAAGAA
TACATGTTTATCAAGATGCAATGTTTTGCGGAAATCGAGCAAGCAAACTTTACCTACCCC
AAAATATAACTTGCCTCTGTCAACATCTGTTAAATTGTTTCCCCATTTTAGAGTTCATTG
CATTTTACGACCTAAATTAAGAGGTTTCATAGTATCAGCAACTGAAACTGATGTAGCAGT
GGAAGAAGTAGAATCAGCTGCTACAGATGATGGATCAGGTGAAGCGTCAGAAGCTTCATC
CGATGCTTCTAACATAATTGAAGAAACATCTGTTCGAGCTAAGCGTACAAGACCTGCTAG
GAAGAGTGAGATGCCCCCGGTGAAGAATGAAGATCTTATCCCCGGTGCAACTTTTACTGG
AAAGGTCAGATCAATCCAGCCATTTGGCGCTTTTGTTGATTTTGGAGCTTTTACAGATGG
ACTTGTGCATGTTTCTAGGTTGAGTGATAGTTTTGTAAAGGATGTCGGAAGCATTGTATC
TGTTGGACAAGAGGTGACAGTGAGATTAGTTGAAGCAAACACTGAGACTGGGCGCATATC
TCTCACCATGCGTGAAAGTGATGATCCTAGTAGGCCTCAGCAACAGAAAGATGCTCCAAC
CAGCAGTGACAGACCCCGAACTCCAAGGAAGAACACACAAAGGAACAACCAAAGGAGAGA
TGAGGTTAAGAAAGTCTCGAAGTTTGTCAAAGGGCAAGATCTTGAGGGCACTGTAAAAAA
TTTAGCCAGATCTGGTGCTTTTATATCTCTTCCTGAAGGAGAGGAAGGATTCCTGCCTGC
ATCGGAGGAAGCTGATGAAGCGTTTGGAATTATCGACAGTGGCTCTTCACTACAAGTAGG
TCAAGAAGTTAGTGTCCGTGTATTACGCATTACCAGAGGACAGGTAACTTTGACTATGAA
AAAAGAAGAAGCTGCTTCAGAGTTGGACTCAAAGCTCAACCAGGGTGTTGTCCATTTGGC
GACAAATCCTTTCGTTTTGGCTTTCCGTAGCAATGAAGAAATCTCTTCATTTCTGGATGA
GAGGGAAAAAGAGGAGGAACTAGCTGAACAATCAAAGGAAGATGCAGAGGAAGCAGATGT
GGCGGCTGATAAGACGGATGTCTTGCCTGAAACTACAGGCAATGAGGAAGAAACAGTCAA
TGCTGCAATTGATAGTTTTCCTGAAACTATAGATGACGAGGATACAAAACAAAACATTGA
TGAAGAAGTGGAGTCAGTTTCTGAAAATGCTGAGGCGTCACCTGTTGGAGATGCTGTGGA
ACCTGAAGCTGAAACTGGCTCATCTGAGCAAATGGCTGATCAGATTTCAGCATCTGAAAC
TGTGGCTGGCGAGAAGGTCGTGGAGAAACTAACTGATGATGCAGTAGCGAAAAATGAGGT
TGAAACTCAAATGGCAAGTGTCACAGAAGCTGCCAAAGAAACAGAGGAAACTAGTGGCGA
TGAGAATGGAAGTATACCAAGCCCAGCTGGACAATCTGAAGCTCCTTTGGAGAATAGCAA
GGATGAAGTAAGTCAGGAAGGTGCTGAAGTTCTGGAGAGTAAAGTTGAAAATACTCCTTC
CATAGAGGACCAATCATCTGATACAGCTGCTCAGCAGGAGGAGGTGACTACCACTGCTGA
GCGAGATAGAAATGTTGCAAACTCAAGTGAGCAAAATGGGACTGCTTCATCAAATGAAGC
CGCAGCAAAAGCTATTTCACCTGCTCTTGTGAAACAATTGCGTGAAGAGACAGGAGCTGG
AATGATGGACTGCAAGAATGCTCTTTCAGAAACTGGAGGTGACATTGTTAAAGCGCAGGA
GTATCTCAGGAAAAAGGGGTTGGCAAGTGCTGATAAGAAATCAACTAGAGCTACAGCTGA
AGGTAGAATTGGCTCATACATCCACGACAGCAGAATCGGTGTCCTGATAGAGGTTAACTG
CGAGACAGATTTTGTATCGCGTGGTGACATTTTCAAGGAATTAGTTGATGACTTAGCCAT

GCAAGTCGCTGCATACCCCCAGGTGCAGTATCTTGTTCCAGAAGATGTTCCAGAGGAGAT
CATCAACAAGGAAAGAGAAATCGAGATGCAGAAGGAAGACCTATTGTCAAAACCTGAGCA
GATCAGATCCAAGATTGTTGATGGGCGGATAAATAAGAGGCTTGAAGAACTGGCCTTACT
GGAGCAACCTTACATCAAGAATGATAAATTGGTTGTAAAGGACTGGGTCAAGCAGACAAT
TGCAACGATTGGTGAAAACATCAAAGTGAAGAGATTTGTGAGATACAACCTGGGAGAAGG
TCTTGAAAAGAAGAGCCAAGACTTTGCAGCAGAGGTGGCTGCTCAAACAGCAGCTAAACC
TGTCGCGTCACCAGGAAAAGAGCAACCTGCTGTTGAAGCCAAGGAGACTACTGTTGAGCC
TCCAAAAGCAGCAGTTTCCGCGGCTTTGGTTAAAGAGCTGCGAGAAGAAACTGGAGCTGG
GATGATGGATTGCAAGAAAGCTCTCTCCGAAACGGGAGGGGATCTTGAAAAGGCACAAGA
ATACCTCCGAAAAAAGGGTCTTTCAACTGCTGATAAAAAGGCCAGCCGACTTGCTGCCGA
GGGGAGGATTGGTTCATACATTCATGACTCCCGAATTGGTGTGCTAATAGAAGTTAATTG
TGAAACCGACTTTGTGGGGAGAAGTGAAACCTTTAAGGAATTGGTCGATGATTTGGCAAT
GCAAGTTGCAGCCTGCCCTCAGGTGCAGTTTGTATCAATTGATGAGATTCCAGAAAACGT
TGCCAACAAGGAAAAGGAGTTGGAAATGCAGAGAGAAGATCTTAAGAACAAGCCTGAAAA
TATAAGGGAGAAGATTGTTGAGGGCAGGGTCTCGAAGAGGCTTGGAGAGCTTGTTCTTTT
GGAGCAGCCTTTCATAAAAGATGACAGTGTTTTGGTGAAGGATTTGGTAAAGCAAACTGT
TGCCGCCCTTGGAGAGAACATAAAAGTTAGGAGGTTTATCCGATTTACTTTAGGCGAGGA
AGCCAAAGAAGCCAAGGAAGAAGGAATAATTGAAGAGACAGCTGCTGTATGAAGTTAACT
CTAACTTGGGATGCTGGATATAAGCAAGCAGAAGATGCATAGGAAGACTTTCCACTTCTG
GAGATTGAGCAAGTTTACATCAGTTGACCTATTTTTGTATCCGAGTATAATGTAGTCATA
CTTGATTAGTTTCATTTTTTTCCCTCCTTTTTCCTTTTCCTTTTATATTTACTGGTTATG
TAACTGTTATATTATCATCAGAAACTAGGACTTTCAAAAGCCATTTTGAAGCAAGATGTC
AAAGAAGAACCATAAGAGGCAACTTCAGTGAGTTCAATTTTTCAGCATTAATCTGTCACG
TTTGCCTGCTTGATGTGTGCAGCTGAAAGCCAGTTTTTCCATTCATATGCCACTCTAATT
TATACAAATAGTATACTTTTGTGCT

>comp76033_c0_seq2 367_1701
TAATAATAGATATTTCTATGGGCTTTTGCCGCTCCTTCCATTCTTGCCTAAACCATGTCC
TCCAATAACTAGCGAACAGCCCACTGCTTCCCTATTGTCCAACCATCAGTCTCTGTTCTT
CCGCCACAACATTATTGCAATCCTAAGCTAAATCCCAAAATTCTCAAATCCCGTCGACAT
CTCCACCATCTCACCCATGTCTCCTTTACACCTTGCGTTTTCTTTACCCTGTTTAGAATT
GACTATACGAATATTCTGTATAGTATTACCCTTTTAAAATAAATATCCCATAATTATAAA
CCTCAGTTCTTGCATCTGCTTTAATTTTCACTACAAAATCATTTCTCTCTAACCTTCTTC
TACTTTATGAAAGACCCAATGAATCAATCAAACCAAGAAACCGAGCATATGATGGACGGT
ACGAGAAACATCATATTCGGAAAATACGAGATGGGTAAGCTTTTAGGTCAAGGAACATTT
GCCAAGGTTTATTATGGCAGAAATATCAAAACCTCAGAGAGTGTTGCTATTAAAGTAATC
AACAAAGACCATATTAAAAGAGACGGTTTAATGGAGCAAATCATTCGAGAAATTTCTATC
ATGAGATTAGTTCGACATCCTAACATAGTTGAACTCAAAGAAGTCATGGCTACAAAGCAA
AAAATCTTTGTTGTTATGGAATATGTTAAAGGTGGTGAGCTTTTCGCTAAGATTGCTATT
GGGAAACTTAAGGAAGATGTAGCCAGAAAGTACTTTCAGCAATTAATAAGTGCTGTTGAT
TTTTGCCATAGCCGTGGTGTATTTCACAGGGATTTAAAGCCTGAAAATTTGCTTCTTGAC
GAAAATGAGAATTTAAAGGTTTCAGATTTTGGGCTTTCAGCTTTATCTGAGCAATTAAGG
AATGATGGTTTGTTACATACACAGTGTGGAACTCCAGCTTATGTTGCACCTGAAGTACTA
AGGAAAAAAGGATATGATGGAGCTAAATCAGATATTTGGTCTTGTGGGGTAATTTTATAT
GTTCTTTTAGCTGGTTTTTTACCATTTCAACATGAAAATATGATGAAAATGTATAGGAAA
GTTTTTAAAGCCGAGTATGAGTTTCCACCTTGGTTTTCTCCTGAAGCTAAGAAGCTCATT
TCAAAGCTTTTAGTAGCTGATCCAGATAAAAGAATTTCAATTTCTGCTATAATGAGAGTT
CCTTGGATATTAAAAGATTTTAGCAGATCAAATTCTTTTTCAAGTGAAGAAAATGGTGAT
CAGAAAAATGGTAGCACAGAGCAGTTAGACTTGGGAAATAGGTCAAAATCAGGTCCACCT
TTTTATAACGCATTTGAATTTATATCAGCAATGTCTTCAGGATTTGATTTGTCAAGTCTT
TTTGAAAGTAAAAGGAAATCTGGCTCAATGTTCACGTCCAAATCCTCAGCTTCAGCAATC
ATGTCAAAGTTAGAATCTTTAGCCAAGAAAATGAATTTTGAGATTGAGTCTAAGGAATTC
AAGGTGAAAATGCAAGGAACATCCGAGGGGCGTAAAGGGAAATTATCAGTAATGGCAGAA
GTGTTTGAAGTTGCACCAGAAGTGGCTGTTGTTGAGTTCTCTAAATCTGCAGGAGACACA
TTTGAGTATAGGAAATTGTGTGAAGAAGATATACGGCCTTCTCTCAAAGACATTGTTTGG
ACATGGCAAGGTGAGAATTGATGGCAGAGACTAAACTGCAATGCTACTTTACTCCATTGT
AAATTATTGGTTCTTGTTGGAGTTATATGTACCAAACACAACCATAACTATAGCATTTTA
CTTTATTCAAGTTCCCAATTTTGTGTAATACTATAATGGTGAGTTGCACTTTCTTTCATC

AGTGATGTGTAAATACTACTAAATGCTTTGTAAGTTCTGTAGCAAGTATTTTAATCTAAC
TTCTGTATGTGAAAACTACTCCATATTCCAGTTGGAGCAGAGACGAATTCAGA

>comp73317_c0_seq28 1673_2236
GGTGCAGGGAAGCAAACATTAGGAGAAGAATACTGTGACATCACTCAGGTTCAGGGAGTT
TATGTTTTGCCCTTTCTGCCTCGGGACTAAGTGTCCTAAGCCATTTGCTTTACTCTTTTT
TACTACTCCCACTGCTCCGATTTTACGTGTGGAATTTGACTCAGCATAGAGTTAAGAAAG
CAAACAAGTTTCTTGAAACTTGTGGTTCTAAACCTGCCATGACATTTTAGTGATTATAAA
AGCATGCCATTAAGGGTAAAATGTGAAATTTAGAGTTAAATTATTACCAAATATAGAAAA
AGGGCCACTCTTTTCGAACAGATTAATAAGGAAATAATGTCACCCAACCCAAAATGGAAC
AGAGAGTAATTTGTTTGATACGGAGGAAAGAATAGTATTCTCTCTTGATTAATGTGTTCG
TGGATATATTTTCTGGCATAATTGTAGTGTTCAAAAGCATGCATTTAGACATTGTATAAA
TGATGTTCTAGCTAACATGTAATTGGTTCTGTTAGCAGTTATATTCATCGAGAAGTGTTG
GTCTAGTACATTCTTTCTTTCTGATAATATCTTGATCCAAAATGTTGTATACATAGAGTT
CAAAACCGGTCCACTGTTCTAAATGACATGATCTAACCATTTGATGTTCAAAACTGCATC
AGCAAATTTTTGCTGTTCTCCTTTGCAATTAAGTGTCTATGTTCCCATTACAATTTGGTA
TAACAGGTTGCAGGACCATATGGGCTTTCCCCAACACCTGCAAGACGTGCTCTTTTTATT
TTCTATCAGTCAGCCGTTCCATACATTGCAGAAAGAGTCAGTTCTAGAATAGCTTCCCGT
GGCATCACCCTTGCTGATTCAATGGCCGACTATACATTTGGGGACGTTCGTAATTCAAGC
ATCGAAACTGAGGCATCAGTGACTGTTGAGATACAGTCATCTTCAACATCCACTCCATCA
ATTTCAGCTCTTTCAAGATTAAAAGCCAAGATAAGGGGTTTCTGGTTGTATGCAGTTCGA
AGGTGGCCTTCGGTATGCCCCCTTCTTCCTAAGTTTTTGTTATGTAAGATTGCCAAAGCC
TGGGTGTGAAATGAAGTGTTTCTCTCTCACTTTTATATCTATTCTCTGTCACATATGTAA
TCCACAAACTCAATGGATAAAGTTGCTTAAGTTAAACTGGATTTCCTTTGAGTTGGATGC
TCTGTTTTCTTAAGTTCTCTTACAATTTAAATTACTGTATTACATTCGCTTGCTCCTAAG
GAAGCATCTATGGCACTTTGTATTTCCTGAAGCATTTCAGACTGTTGGATGGTGATTTGG
ATCTTGTGCACTCGCCAATCGTTAAGCCAGGGGTGCCCTGTGTAGTATTATTGCAAACAA
ATGTTTGTGATATTATATTTATGTAGGACAAAGGTTTAGAAGTACTTCCATCCTGATTAG
AATGCTTTGTAGCACGACAAAACCCTATTTGCTGAGGGTCTACTCGTGGAGCTGCCAGGG
CCATGTTCAACTCCTTTATATAACTTGCCAAAGTGGCTAAGACAGAGTTGACATGTAGAG
ATAATTGGGAAGTACTTCCAATTTTTGTTATAGCAACATACATCCTTGCTGATTCTTGTA
GGGGCTTCCTCTTGCTCGTGAGGTGTTGCAATTGGTTATCCGGACCAATCTCATGTTCTT
CTATTTTGAAGGATTTTATTATCATATATCAAAACGTGCTGCGGGCATTCGCTATGTGTT
CATTGGCAAACCTATGAACCAACGACCTAGATATCAAATACTAGGGGTCTTCCTTCTAAT
TCAATTATGTATTCTTGCTGCTGATGGTCTGAGGCGTAGTAGTTTATCACCCATTTCAGC
TTCTGTTCAACAAGCACCCTTTGGAACCTATCAGACTTCCACAGGCCGAGGTTTACCTGT
TTTAAATGAGGAAGGCAATCTAAGTACTGCTGAAACTGAGAAGTATGGGCTGGTTGCTGA
ATCCACATCAACCTCAGAGGTACTCAAGCACATGGAATTACAAATGATTAATCACAATCC
CTACTCGCAAGGAAGCAATCCAAGCAAATGCACGCTTTGCCTAAGCAGCCGCCAAGATCC
AACAGCCACACCTTGTGGTCATGTGTTTTGCTGGAACTGCATAATGGAGTGGTGCAATGA
GAAGCCTGAATGTCCTCTTTGCCGTTCTCCTATAACACATTCAAGCTTAGTTTGTCTGTA
TCACTCAGATTTTTAGTCATGGAATGGTCAGATATTGCAAAAAGATTGACTGCAAATAAA
TCACCAAGTTCGGGTCTGTTCTCAATGTTTCAGACCCTGAAGCTCCTGGAAGCCATATTG
GGGTAAAAAGGGTGGTTGTGAGCTGTGTAAAGAAGGAGAGTTTTGCTATTGATTTACCAA
AGTTAATCAGGTTAACTTTTGTGCTATGCACAATGGTGTAGCAGATCAATCTGTCGAAAG
CAACCACATAACAGTGTTCCTCGTCTTACACAATACCGCTACTTGTCTTGCACTGAAGAA
AAAGCATTGGATTTCCATCATAAGAAGTTATCTCTGCTGTTCCGTATATTGTTGATTTTA
CATGTGCTTCGGGACCGGATTGACTGGATAAAGTTGCGACAGAATCAATCATTGTTGAGG
TATGACTGGTATTTGTGATATATGACAAGATTACATTTTCACCTGTGATACTCAAAAATT
AGCCTTTTCAACCTCAAAATTATATTATGTGGAGGTGTTACAGATAACTGTCGCTGGTTT
TCTAAACCAAAATATGCACCTTCCTTTATTTACTTCTCATCCTATGCTTCTATTGCAATA
GAAATACATTTCATTGCTTGGTCTACTCGCAATATTTGCTCGGTACTTCATTATACATGG
AAAAAGAATTTACCAGACCGTACTGACTTTACATTTTCCACATTCTCTTGGGCTGAGAGT
CTATCAGAAACAACCTCTGCATACACATTACCCTCCTTAGACCCCACTTGTGGGATTATA
ATGGGTATGTTGTTGGAGGATAGCATCAATAGCCATCAAAGAACGGATGATCCCTGCAGA
AGAATTAAACAAGTCAACAAGCTGCGCTGCATCATTTCTTTAATGCTGTGAGATTTGCTT
CTATTTTGTCATATTTATTACAATTATTTTTAAAACTTTTCACTCACGTTTTAAAACTC
ATTCTTTTGTTTTGTTATTGTTTAGCGAAAGTATCAACCAAGTAATATCGGAAAGAAAAA

AAGCAAAAGCTCAGTTTGGAGCTTTGCAGTTTTGGAAGATTATCTCAATGCAAGCCATTT
CCTTTCTTGTTTTCTTTCTTTTTCTTGCTCATCTTATTTTAGCAAGTGAACCAACTCTTA
GGGGCACGAGTTTTTTCCAGAGAGCTAGAAGTATACACTGGTAAAACTATGGGGGTCAAA
TGGGCCGGGCGGCCCGGTTCTAGACCGGCCCTGACCGGTTTTAAACCGGACCGGTGGTAA
GGGG

>comp69461_c0_seq1 371_982
TTTCAAGCTTTCCTCCCAAATAAAGTACACCAGAATTAGCACTTTTTATACCCCAAAACC
ACGTTATCCTTCACATTTTTGTGCCTGTGCTTGCTTTAATATACTATAGTTTTTCTCGTG
TACGCATCATGCTGCTCCCCCTGGAAACTTCTTCATCTGTTCTTTTCTGTTAAAAACTGC
ACTTTCCATTTCTTTTTTACTTTTCTATTATCAGTCAAAATTGCAAAATCCATAAAATAT
AAGAGTAATTCTTACTTTGTACTAAGCAAGTATATAAATACCAGAACAGAAACTCTCTTT
ATCTATAAAATTGCAATACTCTTACATTCCCTTCTCCTCTCCAGTCTTTATTTAGAATAC
AGTAAACAGCATGAATATCAACAAGGCAGCTTCATTTTTACTTCCTTTGGTGTTTTTCTT
GGGTTTATCCCAAGAATTTGTTAATGGGCGCCCTCTTATACTTTCACTGACCAGACCCAA
GCCAGATGATGCTGCCCTCTTTGCTCGTTGGCTTGTTTCTCAGAGTTCTTGGGGTGTTCT
TAATACTATAGCAAGTGATATGGGAGGAGCACCGTTCGGGAATGTTGTCTCATTTAGTGA
TGGGTTACCAGACAAAGGTCGTGGCATACCGTACTTCTACTTAACGAAACTTGATCCCAC
TGCTAGAAATGCATTAAAGGACCAGAGATCATCACTTACAATCAGTGAGTACGCCATTGG
AACTTGTGGCAAGACAGATCCTGAGAATCCATCTTGCGCCAAAATTACCCTCATAGGGAA
GTTGAAGTTGCTTAATGGAGATCCGAAGGAAACTAGCTTTGCTCAAACTGCTTTATTCAC
AAAACACCCTGAGATGAAAGGTTGGCCCAAGGGTCACAATTTCCAGATCTTCAAATTAGA
GATTGAAGAGATATTTATGATCAACTGGTTTGGCGGTCCCAAACCTCTTACTGTCGATCA
GTACTTGCAGGCTAAAATGTGAGTTTAGAGCAGTAAAAAAGTTGCTAAATAAAAAGTCAG
ATATTGTATTTTCTCAATGAATAAATAAAATAAAATAAAAACTTGCCGTCTGTCTTAGTG
ATGTCATTCCTTATTTAACATGATCCATACACAGGGATAGTCGCGCGGCCATTGCATGAA
TGCCCTTTACATATACAATCTGCAGACATGTTCTTTGCAACTGAAGCTTTCACGCTGGTT
TATATAAGAGGGAAAAAAAACAGCTGTAAATAGAAGATACTTGTTAGGCTCATTATCATT
CATTCATATATGCATTGGGAAAATTGTATGATTTCTTCAACATTCATTGTGATTCTGTAT
TGTAAATTTCGGAAACAACCTCTTTGCTCCCTCAGGGTAGGGCTTAGGTCTGCGTACACA
CTACCTTCCCCAGACCCCACTAAGTGGGAT

>comp76292_c1_seq18 500_808
GTAAAGAATCTGAAAAGAGAGAGGGACAAGAGACAAAAAGGAAACTGAAACCTGAAAGGA
AGCAGCAATTAGTTGACCATCTTTCTGCCATTACCTAATGCTTCTGAAACGCATCTCATT
CTGCCGAAAGTTCCTACAAATAGGACCCATTCTCTATATTTCCCCTTTGACCTCAACAAG
CATAAACCCCACATAAAATCTTAATCAACTTACCAAATAAGAACGTCATTTTCACCTTTT
ATATTACTCGTTTCCATTTCCAAGAGTCCTTTTTGCTTCGCTGAATATTCACAATCTTCT
TCACCACATGTTTTTCATGTCTTTTATCACTTTTATTACCAAAGATCTTACCTTTAAAGC
CCCAAAAATCCCACTAGTTCATTGTTTTATCAAATTTAGAGACATCTGAAGCCAAGCAGC
AGAATCAGAGATAGCTGGTTGACTCTTGGTATTCATGTTACTTGGTTAAATTCTTAAGCT
TCTGCAAGCCAAAAGTCAAATGTACCCCAGGGTTAAGGTGAGACAACAAAAAGAAGAAGA
TGATGAGTATGCATATGAGTCTTTGCCATCATTGAAGGCTTTTGAATCCCTTTCCTTAAG
TGACTTATCTTCTTCAGATGATTCGCCAACATCAGTTGTGAGGATACCCCGAGCATGTAT
TTTAAGCCCAGATAGACATGGGATTCCATCATCAACAGGGAGAACCAAGGACAATAACCA
AAACATTTCTGGTGGAAGCAAAACAAATCCAAGTGCCACTTTAGTGCCACGACCACGTGC
AGTCTTATCAAGCCCGAGTAATCCATGATGAATTTTGTATTGTAAAATTAACACAAACTT
TAAATTCTAGATACTTAATGTTGTAGCACAATCATATTTTTCATCACCAATGTACGATGG
AAGAAACGTAGAAATGCAGTTGACATTTACATTTTACATGCTTATTATTGACATTATCAT
GTAAAGCATACATTCCCCGTTTTTATGGATCTGTACAGATTTTTTCTGAATACTTTAAAG
CTTAAACTTGCTATTTTGAGGCCAGTAAGTGTGGCTGCGTATAAGAGAAGGAAACCCCTA
ATAGGTTTGTAGAATAGCTTCATCTTCATTGTAATTTTTGAGCTACATCACATGAGTTCT
GCTGATAATGATGCAATGTCTTTTTGGCTATCTCATCTCTCTACATTACTTAAATGGATA
AAGGAGTGGGAAATAAGGGGCATGAAGTCTTGGTGATTCTACACTAACATGACAAATGAT
GAAATGATACCTGTAGTTTGTCAATGCATCAGATATATACTTCCGCTATTCAAATAAATA
AACTTCATGCACATAATCAACATATAGGTACTGGTTTTCGGTAAGACATCAAGGGAAATT
TGCTGAATTTGATTGAGCAGGATGGATCTAAATTATTCTTGCATCAGAAATGGACTTACC
AACTCATCTGACAAATTTGCTTAGACGAGAAGTAGGAATGTTGCTAGTGTATTTTCAAAA

GTTGATCTTGACTCTACACTATTTTGTGAAATTTGTTCATGTCTTTCTATATCTCCATTA
TTCCTTGAGCCGAGGATCTATCGGAAATAATCTATATTCCTTCATAAGATAGGAATAAGG
TCTGCATACACATTACCCTCCTCAAACCCCCACTGTGAGAGATTCTTTGTTGTTATTGTA
TCCCCAAGGTAAAACTAACAAACGATATGTATATGATCACTGATAGTTTCACATAGACTA
TTAATGTAATTTACTCAGACGAGACGACATATCAGTTTTAGAAAGTGAAACTGGTGCATT
CTTACATTTGGTGGAGGTCCTTGCTAGGAAATTTTAATTTGTTACACACAATTAATCATT
GTAGGAAGATGGTGTCTCGAAGGACAGAGTACGTAAATGCTCGATCTGGAGAAGAGGTTC
CACCTGAGGATGGATACAGTTGGAGGAAATATAGACACAAATATATTTTAGGGGCAAAAC
ATCCCAGAGAATATTATCGATGTGCTCCTCGTCGTTTATCTAGTTGTACGGCTACTAAGA
TGGTCCAGCGAAGTGAGACAGAGGCATCAGTTTTCGAAGTTATCTATGGAGGAAGCCACA
GTTGTGGTCAAGAAAGCAAACTGCAGCAAAATGGAGAATTAGTTGCTGCACTAACAAAAG
AAACACAATGTGATGAAGCTGGAAGAAGAGGGGAAATATCTGAATCCTATTTCACTGAGA
TGGTTTCAACCCCAAATACTTCATTCAATAATTCCTCAACAGGGATATGTAAACTCCAAT
TCAATCCTCAACATGACCAATTCTGATTTCATGACAACACCAACTTCAGCACATGCAGAG
GGGAATTCCTCAGCTGAGGATCTTTGGACTACATTTTTTTCTACCTTGAAATGAGATTGA
TAGTCAAGTAACTATCTAGTCCACATAAAAGCTACTTTTAGGAGGACTTGCAGGTTCTTT
CATATGACGGTGGTATCCGAACCATCTTGCTCGCACCTTCACTAATTCCACGGTACCTCC
TACCTCTCAACAGCATAGGTATCGGATAACTTTATTCACCAAAACTTAGACAAATGAGAA
GAGATCACCTAATAATCTTTGCTTCTGTTGGACTTGCAGGGCTTTATTCTATCAATATAT
TGATAATTATTGATGAACTTACTGTGTCAATAAAGGCTATTTTGGTATGAAGGATGGCGG
ATAATTAATCTCAGGATTAAATTTGAGATGAATTTAATCCATATTTAATTGAAATAAAAT
CATGGTATAAATTAGTTATCCCGGCAATTAGTAGTAGTTGAGTAACAAACATATCTCTAA
CGTAATGCAAGTTGTTTAGATCAAATACATCATTTTATATTTATAGGGCTTGAGACTTTT
GTTAACCTCATGTTTGAACTGCATTTGCTGATGTAATAATAACAATAACCCAG

>comp72235_c0_seq2 239_1444
ATTGTTGTCGGTTTAAAATCAGGATCCATAAAGTTGAAATTCTAACTCCACTGGTGTATC
CAACCCCACATAAGTACAAATATTAAAGAAATCTTCCAAGCCAAACTATCTAACTAATCT
TAACCCCACTACCATTTATATATTAGCCCCTCCATTTCTTCTCATTCCATCATAAACTTT
TTCTCCAAACACCAAAATAACAACAACATAGTATTCTAGAATCCTTAGTTTCTTGAAAAT
GTTCAGAGATTTCTTGATTTCATGTATTCTGTTTTCTCTGTTTGTGTCATCAACATATGC
TCAATCATGCACTAAGTACAACTTTACAAGCAACCAAGTTTTCACTTCATGCAGTGATTT
ACCTTACTTGAACTCATTCCTTCACTGGACTTATGACCCTTCTTCAAAAACCGCGAAAAT
CGCCTTTCGACACACTAAGATTGCCTCCTCAAGATGGGTAGCATGGGCTATAAATCCAAC
AGCTCAAGGCATGGTTGGTTCACAAGCATTAGTTGCATATCAAAAATCAGATGGAAAAAT
GAGAGTTTATACATCCCCTGTCACAAGTTATCAAACACAGTTACAAGAAGGGAATCTGAG
TTTTCCTGTCTCTGATTTATCAGCTACTTATTCGAATAACGAAATCGCGATTTTTGCTAC
ATTAAAACTTGATAAGTTTAACTCTACAATTGTGAACCAACTATGGCAAGAAGGTCCACT
TTCAGGAGATTCTCCTGCAGTGCATGATACTTCTGGTGTTAATGTTCAATCTGCTGGAGC
TCTTAGTCTTCTTTCTGGACAATCTAAGACTACTACAACTGGAGCTTCAAGCTCACAATT
TAACAAGAAAAATCTGCATGGACTGCTAAATGCAGTGAGTTGGGGGATCATGATGCCTAT
AGGAATCTTATTTGCAAGGTATTTAAAGGATTTTTCAGATCCTGCATGGTTTTACCTACA
CTCCATTTGGCAAATCACAGCTTATGTTATTGGTGTTGCTGGTTGGGCTACTGGTCTTCA
ACTGGGGAGTGAATCTCCTGGTATTAAATTTACTGTCCACAGAACCATTGGGATTGTCCT
GTTTTCCCTTGCTACCCTCCAGGCTTCTGCTATGCTTCTAAGGCCAAAAAGGGATCACAA
ACACAGGATCTACTGGAACATTTACCACAGATCAGTTGGTTACTCAATTGTTGTCCTTGG
AATCTTCAACATATTCAAAGGTTTGAACATATTGAATCCTGAGAACAAATGGAGAATAGC
TTATATTGGAACATTAATTGGTTTAGGAATCATTGCTGCATTCTTGGAAGTTATTACATG
GTGTGTGGTTATAAAGAGAAATAAATCTGTTACTACTGTTGAGAAGAATACACAAGGACA
AGGAGGTTTATATGAACCAAATGGCTATGGAAATGGTACTACTAGGATACATTACAGAGT
TTAGACATTGAAATATTCTTCTGTACTGGAGATAGTTTGATTTGTCTACTTATGTAGTTC
AAACTTGGATATGATGCCAGAGGAATATTTAGACAGAGAGCATCATGGGATGGTTTCTTT
TTCTTTTTTCCCTTTTTTTTGTTCTGTTCGGGTTTTATTTTCTTGAATTTCAGCCTCTCT
ACCTTCTCAGACATGGGTGCATACACACTACCCTCCCCAGAAATTTCTTGAATTTTAGCC
TCTCTACCTTCTCAAGGTAGGGGTAAGGTCCGTGTACACATTACCCTTCCCAGACCGCAC
TCGTTACACCGAAATTGTTGTTGCTGTTGTTTTGTAGTTCGGGTTTTATTTTCTAGAATT
TGTTGTTGATATTTACATATGTACTTTACTCCTTATGCAATATAAATCAGGGTGTTCTTC
TCAAAAAAAAAAA

>comp70843_c0_seq1 178_1023
ATTTTTCTTCTTTTCTTTTTTCCAGTTTCATGAAACTTTAAAAAAATTCCGTATGGATCC
CTTTAATTCACTTTAAAAAGGGGAAAAAGAGAAAAGAATAAAATAAAGGAAGATGAAAAT
CAGTATTGCACCACCTAGACATTACGTTGCGTAAGCTACTGCATCTTCGAACTAGCTATG
CAAACTCTACTCTTGCCGGCTGCTCACGCCGGCGGTGCACCATTACCACTTCCACCGGCC
GGAAAATCATGTCGGTATAGTTTATTCGGACGCCCAAATACCCTAATTTCTTCGCCGTCC
TCATCATTATCTTTTCCCCCCTTAAAGACAAGGCCTCTTTCTTTGCCTCAATTTAGTCTC
ATAAACAAACGGACCAGAATCTTGGCTTCTGCTCCCGTTTCTGCACCCTACACTTCTCCA
AATAACGAGTCTGAAAAAGCTAAGTTAGTTCAGGTTGCGAAAAGACTACAGAATACTGCA
AGGTACTTCAAGAGATTCGGTAGTCTGGGGTTCTGGGGACAGCTGGTATGTACGCTTGTT
GCTGCGGTGATCCTTTCATTTTCTATTGTTATTACGGGGAAGATTACATCGCCTTTCACA
TTCTACTCAACTGCGGGTGGAATTGCAGCTGCTTTTGTTTCAGTTTTTTGGTCATTCGGC
TATATTCGCTTGTCTGAAAAGCTTCGGCGGACAGCTAATGATCCTTCAAAGGCTCCTCCT
CGTGCTGATGTTGTGAAAAGCTTGAAGAATGGAATAGTTGTGAACCTTCTGGGAATGGGT
GCTGCTGTACTTGGCATGCAAGCAACTGTCGGATCATTGGTGGCTAAGGCTCTTACCACC
TCAGCTAACCCGTATAGTATTACTCCTGGAACTAGCCCCGTGCTTGCTTTGGATGTATTT
CTGGTTCAGGCATCAGCGAATACCATCGTTGCACACTTTCTTGGTCTAGTATTCTCATTG
GAGCTGTTGCGCTCAGTCACCTTGCCACCTTCAGAAGGCATTCCGGTGCCAAGGGTTGCA
TGAAGTGTCAACAAGATAATACTGCAGAATAGAGTTAACAAAGGCTTTTGAGATCGCAGC
CATGTATTTACTATAACGAGTGACCTACTTTTTGGTGACTTGCAAATGAAATTACTTTCT
CCAATTTCTGAAAGGCTTCTTGTAGAAATCTCAACGACTGCCCTTCCATATGGTTTTTGG
TGCGTTTAAAAAGAGTTGTTTGCCACCAGTTAAAAATTCCTCTCACGGTTCAATAAGAAA
CTCAACGCCTTCTTTTTGTGTTTGTCTACTAGGCAATAAATTACCGACCATTTCTTTCCT
TCTGTGTGGTTCTTCTCCTTTTTCTTCTGTACCCTGGTTCCTTTTTTACTCTTTGTTCTC
TTACGGGGTAAGACCAATGTATAAAAATATTAGTATTAACATTTGGCAGAGAGTTGCCGA
TATTGCAGAGTTAGAAGTTCATCAGATTTTCAAGATGTTCATTGCGAA
>comp56753_c0_seq1 215_457
ACACTGAGAGTGTAAAGAAGAGACCAACTTGATTAATGACATCGCCCTTTTCCCACATAA
AACAAAACAAACATTGTCAATTTAGTACGGAATCATGTGTAAAGTCATATGAAGAGCTAT
TCTCATGTTTTTCATCTATGAAAAGTTGCTGTCTTTCATCAGATTCTGAACAAGATTTCA
TTATTTATCATAGAATAGAGTGTAAAGAAGAGAAATGGTTATAATGGAGATGCAATCTTG
GTCAAAGGATGTTTTCCCTTTTGCTGCAATGGTGTTGGTTGAATGCTGTGAAATGGGAAT
GATTACACTTGGAAAAGCAGCTATTAATGATGGAATGAGTAATCTTGTTTATGTTGTTTA
CTATAATGCCCTTGGTACCTTTCTTCTTCTTCCTTTCTTAATCTTCCAAAGGTGCAGGTT
TGTCAAAATCAAATACTCTTCTGCCATTTTTTCGTGATTACTTCTTTAGTTTTTGGTAAG
TTCTTGGATGTAAGTATGTTGTGGTCTTTTTGGAAACAGTCTCTCTAACTAACGGTAGGG
GTAAGGTCTGCGTACA

>comp56773_c0_seq1 174_857
AAGTAGTATGTACGACACACCTTACCCTACAACACACCTTACCCTACCGTCGGTAGAGAG
TATAGCGGTAAAAGCCACGCAATTTTAAATACAAGAACAAGAATGACCATCCAATTTACT
GTTTGCAATTTGCAACTAGAAGATTAGAAGTTAACGGCTCTCTCTTTGTTTCCATGGCAG
CAACGAGCGCAGTGGCAATGGCAATGGCTTCACCCTCGCCCCACCTGCTTATCCACTCTC
CACATAGTAGTGTCAGGGCTATCTCCAAGAATATCTTGTCCAGCCATTCTGCAATTTTTC
CCGCAACAGCGGTTACATATCAGGGGTTATTGAATCACTGTATCTTCTCACGAGGATGCC
TAAGATCTTTGGTCAAGCCAGTATTTGCTGCTGGTTCAGGCCTAGAGGCATCTGTTGCTG
GTGATGAGGCTTTGATAAGCGTTAAAAATGCTAAGATAGCCGTGGAATCCCAAGATGCTG
AAAAGATACATGTCAGAGTAGATGTGGACAAGGAGGACACAAGAATAGTTTTTGAGAAAG
TTCTGGCAAATTTAGCACGTTCTGCACCACCTGTTCCAGGCTTCCGCAGGGAAAAAGGAG
GGAAAACATCTAAGGTTCCTAGGGACTTTCTGCTGCAGATACTTGGTGAAGATCGAGTTA
CCAATTTTGTAATCCGGGAAATTGTTACCTCAACCCTCGCTGATTATGTGAAGAAGGAGA
ACTTGTCAGTGAAGGATAACAAGATTAGCACCACCCAAACTGCCCATGAACTCAAATCAT
CGTTTACTATTGGAGCTGAATTCGGATTCAATGCCACCTTAGAGCTTGAAGATGCAAAAA
CTGAAGCCACCACATAAACTGACAGAAGATGTGACACTTCTTTTGCGTAAATGTCAATCT
CATTATCTAATCTGTTAGAATCAGTAATTCTTGAGGTCAGTAGATTGAAGATCTTCAAAG
AGCTACTCCTATTAATTTGTAAAATGTGAGAAGTGATGGCTTAGGTTGCATGCATGATAT
ATACATATGTAATGATGTGGTGTAACATTTCTATTTCTCTTTTATGGCTTTGTTGGGTTG

TCAGTTGTGGTGGCAGAAAAATATCTGTCTCTAATAATGTTTGTGGCATTGGGCAACTAT
CAGCTTTGAATTGGAAAAGAAATCCAATAAAAGTTAGTCGTAATTGCAGTCGTGGATTCT
CTCGAGAGGTCATAATTGTCGCGAGCAAACATTAACGAGGAAAACTTTTTTGTTATGTTA
CTAGTAATT

>comp75362_c2_seq1 7645_8472
TAAAAAACTATCTTATCCAACCCCTACTTTCATATCCAAAATGACATTATTATTTTGTCT
CAAATCACTGTTACAGCCATCATTTCCGCTGTATATTTAACTGACCAAAACTTTGCCAAT
ATCAGTAGTTCAAACACTTGTTTTAATATCTTTTGCTAATTTCAGATCAACCCCTTTTCA
TTTCTTGAACAATTTTCATGGCTCTTCATGCTGCTGCTCTTGTTCCTTCTGCTTTCTCCA
TTTCCAAAGAGGGCAAAGCTAGTGCAAACTTGAAGAATTCTAGTCTTTTTGGAGTCTCTC
TCTCTGACTATACTAAATCTGATTTCCGCTCCTCTTCATTCAAAGTCAAGAGCCAAAGAA
GATTGTCCAATGGAGCAGTAAGGGCAACAATGGTTGCATCTCCAGATGTAACCACTAATT
CTCCAGCAGGAAAGAAAACTTTAAGAAAAGGGTGTGTAATAGTCACTGGAGCCTCTTCAG
GATTAGGCCTAGCCACAGCAAAAGCACTATCCGAGACCGGAAAATGGCATGTAATTATGG
CTTGTAGGGACTTTCTAAAAGCTGAGAAAGCTGCAAAATCAGTAGGCATGCCTAAGGAGA
ATTACACCATCATGCATTTAGACCTCGCGTCGCTTGACAGTGTTCGCCAGTTTGTCGATA
ACTTCAGGAGGTCCGGTCGCCCTCTTGATGTGTTGGTTGCTAATGCAGCTGTGTATCAAC
CTACTGCTAAAGAGCCTTCATTTACAGCTGAAGGATTTGAGCTTAGTGTTGGCACAAATC
ATCTTGGACATTTCCTTCTTTCAAGATTGTTGCTTGATGACTTGAAGCAATCTGATTACC
CTTCTAAAAGACTCATAATTGTTGGTTCAATTACAGGTATAACTGAAACACTTAACACAC
TGAGTATTACAATAATCTCCCATAAGCGCTATAGCGCTCTCACATATCTGATATGCCCTT
GCTTACAGAGGCGGATCCAGAATTTAAATTCTATCCGTTCAACCTTTAGAACTTTTAGCA
TTGATTTTGTTGGTTTAAACCTATCATTTACTGCAAATTTAATGAACTTTTACACAAAAA
TCTATGTTGTGTCTCAAAAGTACTTGGTTCAGACGAACTTGGTAACGCCACTCTACATCG
GCCCCTGCTTGCCTATTGCCTTATCTCTTACTAAGAAACTAATCTAGATAGACACCCTTC
TTTGTCCTTGTTTTAGTAAAATACGATCTCTTTTAGCTGGTTCACCTCATATATTCATCT
ATTTCTCAAACCATTCTCTTTTTTAGCTGAACATCTATCGGAAATAGCCTCTCCGCCCAG
GTAAAGGTAAAGGTAAGGCTGGTCACATCCTACCCTCTGACCCAACTTACTGGAATCCAC
TGGGTGGTTGTTGTTGTTATTGTTCTCTTATATACTCTTTGTAGGGGGAGTAACTTTTCT
TGACTAAAATCACATGACAGGGAACACAAATACTTTGGCTGGAAATGTACCTCCAAAGGC
GAATCTCGGTGACTTGAGAGGTATGGCCGGGGGTTTAAATGGTATTAACAGTTCAGCGAT
GATCGATGGTGGGGAATTTGATGGCGCCAAAGCATACAAAGACAGCAAGGTCTGTAACAT
GCTCACAATGCAGGAATTCCATCGTCGATACCACGAAGAAACTGGCATTACATTTGCCTC
TCTTTACCCTGGCTGCATTGCAACAACAGGGCTATTCAGAGAACATATTCCCTTGTTTAG
GCTCCTTTTCCCTCCATTCCAGAAGTATATTACTAAGGGATTTGTTTCTGAGACTGAAGC
TGGAAAGAGACTTGCTCAGGTTGTAAGTGATCCAAGCCTGACTAAATCAGGTGTGTACTG
GAGCTGGAACAAAGATTCAGCTTCATTTGAGAACCAGTTGTCTGAAGAAGCTAGTGATGT
AGAGAAAGCGCGTAAAGTATGGGAAGTCAGCGAGAAACTCGTCGGTTTGGTTTAACTTTT
CGGGCTAACCTTAACCATTGCCAAGGCAAACAAGAAAAGAAGAGTTGATGAGTGAAAATT
TGTATCGGAATTTGTAGGCTGTTGTAAAGAGAACTGTCACCTTTAGTTAGGATTGAAAAT
AAATGTTATTTTCTGATGGAAAATGTCACTAATTTACTTTGAAGTTAAATGAAGTGATGG
CTCATGAGCATATTGTTAGTTCAATTATCTGTTTTGCTTCCCTCTATGTGGCCAGAATAA
AGTATATTTCAAGTATTAGAACTTTGCAGCCATATACGCGAGAAATTTAAAAATAGCCAG
ATTTACAATTGATCAAAAGTAATTGGAATTTAGCCACTTTTTATATAAAGATAAATCTGA
GCGAAAACAATGTTCAAAATCCGAAAAATACACCCGCAAATTTTACTGGAGTTCCAGCAT
AAGTATGCTTGAACTCCCGCATATTTTACGGGAGTTCCAGGATAACTATGCTAGAACTCC
TGCAAATTTTATGGGAGTTAAAGTATAATTATGCTAGAACTCCCGCATATTTTACGCGAG
TTCTAGCAAGTATAAATGTCTAGTATAATATACTGAAGTTTGGAGCGCTGGTGTTCCAGC
CTCCCGTATATTATACAGAGTTAGCAAAGTATACCGGTCCAGCATAATATGCTGGAGTTC
GTACATAATTGCACAAGAACTTCCGTATATTATACGGGACCGGTCGCTGTTGCAGTAAAA
TAATGGCTATTTCTTATTAACTTGGTAAATGCTGGCTATTTTTGATTGACCGGTGTGAAA
ACTGGCTATACCATGATAATTTAACCTCATTTGCTGCCCGCTTGGATGATAGGCCCAACT
TCCAGTTGATTTTTGGCCCAGCAACTATTTTTCTTGTGAGGCCCAACGTCAAGTATATGT
CACATTCTTTCTTTCTTCTTCATCCAAGTTTGGCACACTCTCTCCATCTTCGGCGAACTT
TGCTTCTCTCCGGCAAAAATAGAGCTGTGATTTCCCTTTTTGTCCTCTGGGTAATCCCTT
TTTCCAATGCTCTTTAGTTGTAAATATTCTTAATAGAAAATGAAAAATAAAAAAAGAGAA
TGCCATAAAAGGTGCATTTTTATCATTACATTTTTAGGTTTCAGCATTCCTTTTAATGGT

AATATAATAATCTATGAACTGGGGGTTTATCCGTTTGGTGTTTATTGTTAGTTACTGCAT
AAAGAGTATAACTTCAACTTCTGATTTTTCCAATTTAATATGCATCTTAGCTTTCCATGT
TAATAGTATCCTGTGATATTCTTAATTGAAAAAAGAAATTAGAAAAAGGTGGATAAGGCC
AAAAAGTTGTAATCTTTATCATTATATTGCTGGTTTTCAGCATGCTTTTTAATAGTAAAA
CACGAATAAATTACCCCACCTGGTGTTTACATCAAACCATACTGATTTACCATGGTTAAT
TCATAGATTACGCATGAAGTGAGGAACTAGGAATGCTGCTTGGAGAAAATGATCGCTCTC
AGGAAGTTGAACCTCAGGATAGCAGCTTGCCCTTTTTCTTTGCTCCGGTTGTTTGCTGTC
AATGGTCATTTTGTAGTTTTCAAACACGCAATTCAGTCTGGTTGGAGTAGTGGGTTTGTC
AATCGGTTAGGTGGCTTCAGTCAGGAAAACATAGAGATTCAGAGTGGAGTCGACTGGACT
GTGTTTGCTCAGACTGCTCCATTTTCTACTGTTGCTGGGACAATCTTAATCCAGGCTCAA
GACCTTGGTAAAATGTCTGAGGAGTTAGAAAATGTGATTGATGAAGATAAACTTGATGAT
GCATGGGTTTTGTATGAGCGACACATACAAATGGCCGGGTTTCCTAGAAAATCAATTGTT
AATAAACTTGTAGCAGCTTTTGCCGAAAGATCAGATTTTGGACAGCTGGAGAGGGCTTAT
GCTTTAGTTGAACAGGTATTTGAGGAGAACAGGCATGACTTGCTTGGGAGGAATACTCTT
ATCCATCTTGCTTTAGCCCTTGCTAAATGTGGATTACCTATTCCCGCATCTACTCTTGTA
AGGAAGCTTGTCGAAACGGAAAAGTATCCACCTGTGAGTGCTTGGTCTGCAATTCTGGCT
TATATGTCACAAACCTCTGATGGAGCATACCTTGCGGTTGAATTGGTTCTGGAAATAGGT
TACTTGTTCCAAGACGGAAGAGTTGATCCCCGTAAAAAGAGCAACGAACCTCTACTTTCC
ATGAAGCCTAATACTACTTGTTTTAATATTGCTTTAGCTGGATGTCTTCTGTTTGGAACT
ACTAGAAAAGCAGAGCAGCTCCTTGACATGATTGCCCGAATTAACTTGAAAGCTGATGCT
ACCTTACTGATCATAATGGCTCATATTTATGAGAAGAATGGACGAAGGGAGGAGCTCAAG
AAGCTTAAGAGAGACATGGAAGAGGCCCCTAATGCGACTGAGATGCAGTTCCGTCAGTTC
TATAATTGTTTGCTTTCGTGCTACTTAAATTTTGGAGATCTTGAATCTGCATCCCATATG
GTTTTAGAAATGCTTCGGAAGGCAGAGAAAGCTAAAAATTCTCTTGGTGTAGCTAATTTG
CTGCTTGAACCTTCCAGAAGTGGCAATGCATCACCTGTAAATGTTCCCCTCGATGATGCA
TTTGATCGAAATCTGGATGGATCAGAAAACTTTGTATCGTACGAAGATCTCTGTAGAGAC
AGAAAGTTTTTAAAGCTGCAGATTATTGCTAAAAGCTTACTTGATGTCTTGGTAGTTAAG
TTGCAGAAGCAAATCGAATTTATTACCAGTGAACGTGGTATTCTCCAGCCTACTGAGAAA
TTATACGTTAAATTGGTAAAGGCTTTCTTAGAAGCTGGGAGAACAAAAGATCTTGCAGAC
TTTCTTATTAAGGCAGAGAAAGAAGATTCTCCGGTTTCAGTTGATGATTCTTCCTTGGTT
CATGTGATAAACTCTTGTATTTCACTTGGATGGTTAGATCAGGCACACGACCTACTGGAT
GAAATGCGCTTGGCTGGCGTTAGAACTGGTTCATCTGTTTATTCATCTCTTTTGAAAGCA
TACTGTAGAGAGAACCGAGCTGGGGAAGTTGCATCTCTGCTTAGAGATGCTTGTAAAGCC
GGGGTTCAGGTAGATGCAAGCTGTTACGAAGTATTGATCCAGTCCAGGGCGCTTCAGAAG
GACACTCAAGGGGCCCTTGATCTGTTCAAAGAGATGAAAGAGTCTAAGATACCAAGAACG
GGTCATCAAGAATTTGAGAAATTGGTCAAAGGGTCTGCAGAAGGAGGTGACCCCAGTTTA
GTGATGATGCTTTTGCATGAAATCAAAGAAGGACAAAAGGTAGATTATGGAGTTCATGAC
TGGAATAACGTAATTCACTTTTTCTGCAAAAAGAGGTTGTTGCAAGATGCTGAAAAGGCT
TTTAAGAAGATGAGGAGTTTGGGACATGCTCCAAATGCACAGACTTTTCATTCTCTGGTT
ACAGGTTATGCCGCCATTGGTGGAAAATATTTGGAGGTGACGGAATTATGGGGCGAAATG
AAGTCTCTTGCTTTTTCTAGTGGAATGAAGTTTGATCAGGAACTGTTAGATGCTGTGCTT
TATACATTTGTTAGAGGTGGTTTCTTTGTTCGAGCGAATGAAGTTGTGGAGATGATGGAG
AAACGTAATATGTTTATTGACAAGTACAAATATCGTACCCTCTTCTTAAAGTACCACAAA
ACACTCTACAAGGGAAAGGCTCCGAAATTCCAGTCAGAAACCCAGATGAAAAAGAGAGAG
GCAGCATTGAACTTTAAGAGATGGGCTGGGTTATGCTGAAGAGTCAATTGTCTGTAGCCT
CAAGGATATGACCCTGCAATATTGGAACTTATTGCTCCGATAATTCCTAAATGGGATGTC
AGCGAATTGGATGGAGCTCCATGTTGATCCATATGTCTTAGTTTTGACGTAATGAGCTTT
CTTTACTTTGAGTTAATTAACATGGCGGTTTCGCCTAGAACGTGAACTATGGAGGAGGAT
GCATTTGATGTAGGAGCAGAGTGGAATTTGAAGCAGGGTATCCCTATCCATCGCTTGATG
CTAGCAGATCAAGGCATCTCCGTGGTTTTCTTTGTCATTATCAATTTTTGATCCTCCAAT
CCTTGTTATCATAAATGCCTTGCAAATTAGTCTTAACAGTGAGATGCATCCTGCTTTCTC
TTTGCATGCTTTTGAGGAAAGCGTCTTAATCATGGTTGATTTAGTCATGATTGAAGATCA
TCTTTTTATTCCGTGCTCTGGAATTATTTCAACTCGATGTTCAGAGTGAAGCATTTATTT
GCTTTACGATAAGAAATCCGTCCATTTAATTATACGGATGCATTATTCTTCTATCGAATG
GCGTATGCCCGAGGTGCCTGCATTATTTAACATCCCAAAGAAGGGCTTAGTTTCTGCTAA
ACTGGTTGGCACTACCATTGCCCTTCTGCTACTCATCATGATTCATTTAAGGGAACCTGA
TTTTGGTTACTTTTGATTGAATATAGATATTTCGAGAATGTATGTAACATTGAAAGATCT
GAAAACTTTGAGGCTTATTCTTTTAAGAAGTAAACTTTCTACAGAATCTGGTGATTTTTT

ATTGGAACACTGAAAGGCCTATACTTTTAATATGTAAACTTCTTGCTGATACTTGTCACT
CCCTCATCCACGTATATACATCTTTATTTATCTTTCAATCACTTGCAGTTTTAATTTCTT
CGAGAGTTTATGGTGCTGAGTCGCTTCTTTGGCATTCCCTTTTGTTAAGACGAGATAATA
TCTGCAACAAATGGAATAAGGAGCTGGCCTTCGCTAGAGACAGCCCGCTGAATCATAAGG
GCCTCCTGCAATATATATATATATAGTAGATTTGTTGAAGTTGAGCATTCTTCGCTTCTTCT
TTGGTAAGACGGTTCAAAACATGTTTTCTTAGAGAAGTAATTGTGAAGTGATATGGTTAA
ACATCGAAGTGTTAGAGGGATTCTGTAGTTTAGTTAAACTTTCCTCTCATACTTGTTATG
TTCTAAGGATTAGTTGGCTCTAATTTATTGTTGTATGTATTTTGAGTTCGGTATGAACAA
TGAAGGTGTTAGATTGTTGTGTTGTATTGCCTTTGAAAACTTTGATCATTTAATATAACT
ATGTTTTGCGCGGGTGAATTCATAATATAGTTGAACGGAGAGTGCGAAAGGAAAATGATG
CTTCAACTTTTAACTATATTAATATTTCTTTAATTTTTCTCTTTTTCCATGATGGTGGTG
TCCAGTTCAGTTTGCATGCACCTCGATTAGCTCACTGGTTGCTTTACTATCTCCCGCCAG
AACAGGATTTTGCACAGAAGTAGATAGCATTACTAGCACTCAATCTCTGAGAGATCCTGG
AATTTTATCGTCGCCAGATAGCGTCTTCAAGTTGGGATTTTTCAGTCCTCTAAACAGCAC
TAATAGATATGTGGCGATGGAAATGTCGTCGCTACAAATGGAGAGGAGGAGATTCTTTGG
TCATCAAATATTTCAACAACCTCTCAGGTAAACTTAGTTTCCCTTCTCCAAGATTCTGGT
AACTTTGTTCTCGTAAATCGTCTGAACAACGCGAGCACAATATGGCAAAGTTTTGAACAT
CCTTCTGATTCACTTGTCTCTGAAATGAGAGTAAGTGAAAACACAAGGACAGGGAAAAGG
ATTGAAATAAAATCTTGGAGAAGCCCATGGGATCCTAATTTTGGAAACTTTTCTTTGGGC
ATGAAATCTGAAATCATTCCTCAGGTATATATTTGGAAAGGTAACCGTCCCTATTGGCGA
AGTGGTCAATGGAATGGCCAGATTTTCATTGGACTGCAGAATATGTATTCTGTGTCCGTT
GATGAATTCAGTGTAGTGAACGATCGTGAAGGTACTGTATATCTTACTGGACCTGTTGGG
TTTAATTTCTTAACGAAGTTCATCTTGGATTGGAAAGGAAACTTAGTTCAATCATTTTGG
GACGAGAATGAGACAACTTGGAAAGTAATGTGGTCAGCTCCCAATAATGATTGTGAAGTT
TATGGAATGTGTGGTCCATTTGGGAGCTGCAATTATTTGGAGCCTCCGATTTGTTCTTGT
CTGAAAGGTTTCGAGCCAAAGCATAGGGAAGAATGGGAAAAGGGGAATTGGACTAGTGGT
TGCATTAGGAGGAGAGCTTTGCAATGTGAAGTGAAGAATAACTCGGGGAATTCAAGTAAA
GAAGATGGATTTCTAAAGATGGAGTTCATGAAATTGCCTGATTTTGCAGAGAGGTCATCT
ACTTCAGAAGACCAATGTATAAGCCAATGCTTGCGTAGTTGTTCCTGCATTGCATATGCA
TATGACTCAGGTATCGGCTGTATGTCGTGGAGTAACAACTTGATTGACATTCAGCGGTTC
CAAAGCTGGGGGGAAGATCTCTATATTCGGGTGGCATATTCAGAGCTTGGTATGTATTCT
GGTGCTCAATAAAAGACTCAATATCCTCTCAGCCCATTTTAGTTTGGGAGAATGACACAA
GGATACTTTTTGTGCCACTGTTGATTTTTTGACATTTAGGAGAATAGCCCAGTGCCTGAT
TTTAAAGCATGCTTGCTAGAATTCAGCAAGTTATGGTGATTGTCAAGATTTGTGACAAAT
CCCGACGATCACCAGAATTTTCAGCCACAATACTGAAAAAATTTGGCATTCATCAGGATT
TTGCCACAATCCTGACAGATTACCAGAATCTTTTCAGCATTGTGGCTTAATATCTGGCAA
TTGCCAGAAGCAGTTTCCAAAATTCTTACCTGCGCGCTTCAAAATTCTGGCTAGAGGTTA
TTTTCCGGCGACATTTCTTAACAGGGTCAAAATTTAAACAGTGCTCCTAAAAGGTCCATC
CAGCGTAATCCTTCTCCAGCCATTGTAGTTTAATCTGTCATTTAAGCTATCATCGACTTA
ATTGAATTCCATGCATATACTTTTTAGTTTTTGAATTCAGTAAAGAACTGATACTGGTTC
ACCTTTTGTGTTTGCTCTACATTGCAGATCATCATAAAGACATAAAGAAAATTGTTATTC
CAGTAATTGTCGGTACTCTTATACTCTGTGTTTGTCTGTTCCTTTTCTGTTTAAGGATGG
TCAGACGTAGAGGAATGAAAAGCAAGGAGGCAGTATTACTTGGTAACAGAATGGAAGACT
TACCAGTCTTCAACTTCGAAACTCTTGCAAATGTAACAGGCCGATATTCTGAGGATAATA
AGCTTGGTCAGGGCGGTTTTGGTCCAGTTTACAGGGGAAAATTGGAAGATGGGAAAGAAA
TAGCAGTCAAGAGGCTTTCAAAAGCCTCTGGACAAGGGCTAGAAGAGTTCATGACTGAAG
TGTTGGTGATCTCTAAAGTCCAACATAGAAACCTTGTTAGACTCTTGGGATGTTGTGTGG
ATAAAGAGGAGAAGATGTTGACTTATGAATATATGCCAAAGAAAAGCTTGGATGTGTTCC
TCTTTGATGAAGTATACCAAGGCATTTTGGATTGGAGGAAACGTTCCATTATCATCGAAG
GGGTTGGGCGAGGACTCCTTTATCTTCACAGAGATTCAAGATTGAAGATAATCCATAGAG
ATTTAAAGCCAAGTAACATTTTGCTCGATAACAAATTCAATCCAAAGATTTCAGATTTTG
GCATGGCTAGGATTTTCGGATAAGACCAAGATCATGCAAACACAAAGAGAGTAGTTGGTA
CTTTTGGATACATGGCCCCGGAATATGCAATGGAAGGAAGATTCTCCGAGAAATCGGATG
TTTTTAGCTTTGGAGTTTTAGTGTTAGAGATCATCAGTGGCCGAAAGAGTACAAGCTCTT
GGAGTGAGACATCCTCTTTGAGCCTTTTGGGATATGCATGGAAGTTATGGAAAGAAGAGG
ATTTATCAACTTTTATCGATCCGTTTATATTGAATCCGAGCATGGAAATGGAGATCAGAA
AATGCATATAAATTGGTTTACTCTGTGTTCAAGAATTTGCTGAAGATAGGCCAAGTATTT
CATCTGTTCTTGCCATGCTTACCAGTGAAAATACAAGTCTGCCAACACCCTTACAGCTTG

CTTTTACTGAAAGACAAGTTGGTTTCTTCACAAAGTGTAACAAGAATAGAGAAAATGAGT
GGAGTTTGAACCATCCAAGTATCTCAACTCTTACTGCTAGATAACATGAATATGTGAAAT
TAGTTTTATAATAAGTACATGATAATCCATCCATCTCAATTTGTGTGACACTCCTTTTAC
AGTCACACTTCTGTATAACCGCATCCTTATATGACAGTCATTTACTATAAAAGCCATATT
TTTCTCGGAAAAAAAAAA

>comp73495_c0_seq6 312_1019
AAACAATTTCTTAATTTCAACGTTATAAAAAGTCATAAAGGCTCAATTCCTCAAAATAAG
CTGGCCTAATGACCTGAAATAATAGTTTAAGGACAACATTATACTTTAGAAAAGAATATT
GAGAGTTGAGAGTCATCTTTCTTTCTCGTCCAAAATATATTTCTTCTTCCTCAAGCAGAA
CAAAAGTAGCAGTGCCGAGGTCTTTACATCTCACTTGCCCCTATTTCTGCAAATCCTGTT
TACTGCTGGAACTTTGCGTTATCATCTTCAAACTATCGATTGGTTATATTAACGACTAGT
CTTTAAACTCAATGGGTGATAGTCAGTACTCATTCTCACTCACCACTTTCAGCCCATCTG
GTAAGCTGGTTCAGATTGAACATGCATTGACTGCTGTTGGATCTGGTCAAACTTCATTAG
GGATTAAAGCTGCTAATGGTGTTGTAATTGCTACTGAGAAGAAGTTGCCATCCATCTTAG
TTGATGAAGCATCTGTGCAGAAAATACAGGTTTTGACACCTAATATTGGAGTTGTCTACA
GTGGGATGGGCCCTGATTCTCGAGTTTTGGTTCGGAAAAGTAGAAAGCAGGCTGAGCAAT
ATCACCGACTCTATAAAGAACCAATCCCTGTCACACAACTGGTGAGGGAAACTGCTGCTG
TCATGCAGGAATTCACCCAATCAGGTGGTGTAAGGCCATTTGGTGTTTCTCTCTTAGTTG
CGGGGTATGATGACAAAGGTCCTCAACTATATCAGGTGGATCCATCAGGCTCATACTTCT
CTTGGAAGGCTTCAGCAATGGGAAAGAATGTGTCAAATGCAAAGACATTTCTCGAGAAGA
GGTATACTGAGGATATTGAACTTGATGATGCTGTACACACTGCTATATTGACCCTGAAGG
AGGGATTTGAGGGGCAGATCTCCGGAAAAAACATTGAAATTGGCATAATCGGCAATGACA
AAGTATTCAAAATTCTCACACCAACTGAAATAGATGATTACCTACAAGAAGTAGAATAGA
TTCCGTTTTCTTCTCTGGTGGTTTGAGGAAAATTAGTTGTCAACTATTTGAGGATATATC
GTTTTGATTTTTCTTTGTTACAGTTGATTATGGCACGCGACAAGGAATTTGTTTATTTTT
CTTTATTACAGTTTGGCCAGCACTCTTGGTTTTTGTTGACCATGAAACTGTTGCTAGTCT
TTGTTGACCATGACCTGAATTTATGTGTCAAAAAAACTATTCCCATTTCCATATAATAAT
GGCACTGAGCCGATGGTCGATTGGGAACAGTCTCTCTGCCCTTTCGGG

>comp71383_c0_seq6 99_857
TGAAGTGGCATATAGCTGCCAGGAAATAAAGAGAGTCACAAATATAGAGCTTTTCTTCTT
CCATCAAAGAAAACATAAAATTTTAAGAAGAAAAGATAATGCCGATTTCAAACATTGCCC
TCGGAAATTTAGCAGAGGCTAGCCAGCCTGATGCTCTCAAGGCTGCACTAGCTGAGTTCA
TTTCAATGCTCATCTTTGTTTTTGCCGGTGAAGGCTCTGGCATGGCCTTCGGTAAGCTAA
CAAATGGCGGAGCAGCCACACCTGCTGGATTGATATCGGCGGCTATAGCCCATGCCTTTG
CCCTTTTTGTGGCAGTTTCAGTAGGAGCAAATATTTCTGGAGGTCACGTAAATCCTGCGG
TCACATTTGGTGCTTTCGTGGGAGGTCACATTACCCTTTTCAGGAGTGTTTTGTATTGGA
TTGCCCAATTGCTTGGATCTGTCGTCGCTTGCGTGCTCCTCAAGTTTGCCACTGGTGGAT
TGGAAACATCGGCATTCGCACTCTCAACAGGAGTTACCCCATGGAACGCAGTTGTTTTTG
AGATAGTGATGACCTTTGGCCTTGTTTACACCGTATACGCAACTGCAGTTGATCCTAAGA
GGGGTAATTTGGGAATTATTGCCCCAATTGCAATTGGTTTCATTGTAGGTGCGAACATTT
TGGCTGGTGGAGCCTTTGATGGTGCATCAATGAACCCTGCTGTGTCATTTGGTCCAGCAG
TGGTTAGTTGGACATGGAATTGCCACTGGGTCTACTGGCTCGGACCATTTGTTGGTGCTG
CCATTGCTGCTTTGGTTTATGAAATTATCTTCATTGGTGACAACACTCACGAGCAGCTCC
CCACCGCTGATTACTGAAGAACAATTTCCTCCTTTCTTTCTCTAAAACCAAATGTTGGAT
ATAATTAATTTAAGCTAAGGTTTCAAGGTTGTGGTTTATTTTGCTGTTCGCTCTTTGTTT
TTCTCTTTGTTGTGGGTTTTCTTTATTGTAAAATCGTGAAAAATCAAGAGATGCAAGTGT
CATGTTTAATGTACTGGAGTTGACTTCTCTTTGATTACCTATATTTATTTGTTGCAGACA
ATCTAACATTAACAACTTGCTACTCCCCCCTAAAAAAGTGGATAAGAAGTTAGAATGTTG
CAGAGCGAAAAGAATGGACATTTGGCCTAACTCAACCCCAAAAACTAGCTTATGAGGTGA
GGATTACCCAAGCCATATAAAGAGACCAAGGACCTCAAATCCCTTCCGATGTGGGACAAA
TCAACACTCCCCTCACGCCCAGGCCTCGGGCAACATAAATAGAGGCTCAACATCGGTAAC
AATAATTGGGACGGGCCTGGCTCTGATACCATGAAAAGAATGAACCTTGGGCCTAACTCA
ACCCCAAGAGGTGAAGATTAACCAAGACCATATAAGGAATCACATCACCATTCCACCCAT
CGATGTTGAACCAACATAAGGTTGGTTCGTGTTTTGTTGAAGCCTGGTATTGGGAAAGTG
ATCTGATTTTTGAATTTACCTTGGCCAAAAAAGTGAGAGATCTTTAAATTGATTAGACTG
GATGAGAACCTTTTTATTTATTTTTTATGGGATATACTTTGTGTTTTTATGACTATGTTT

ACAAGTTGAATCAAGCTGAAAGCTTCTTAGAAGATTTCTGGCTCAAATTTAGCCTGAGTG
TTTCAGATTCCATAAGCTTTAGTTAAGCTAGTTAAGTGAAAAATATTGAAAGACACCAAA
GAGGTGCTGCCCTGTACTGCTGCCAGTATAGAATTAAGAAAACCTGGTCAGTATTCTTGA
TTTATCTGCATACTAGCCTCATTATCCTAAAAACTCCTAAATGCAACAAAGAATAGATTG
CGGAGAAGTCAGGCTGAATCTTTCTAGACTCATTCATGATCCTCCTGCATATCCCCAAAT
TCTTGAAGCCAGAAGCACCAGCAGGAGCTCCTCATATTATGACAAATGACATAGTTGTAC
GCATGGGTTGCTGTTCTGGGTCTACTTTCTGACCTATAAGAGCATTTGGTTAGATCTCTG
CTTCCATTTCCTCAATAGATTGTGTAATAATTTTGTAAAGGAGGAGCGTGGTTGCAGATA
GGGGTAGGGTGTTGTGTACGAATAAGTTCACTGTAAACTCATGCAACAACCGTATGAGCT
TATGGAAGATGTAATATGGAAACATACTGATTGGCTTATGAGCCTACTATCCAGGCAGGT
TGAGCAATCAGCTTAGCTAGAGCTGGATGCTGACTAACTCGCTCTAGTCCAAAACGAGGT
AATATGAGGAGTTCATGAATAATCCAGCTCCATGTGAAAGCGCTACAGCGTACTTCACCA
ATATCTAGTTCTTTCCTTTCCATTGAACTTATAAGAAACACATGCTGTTTGCTCTATACT
TGATTATGTTAATGCAAGCTAGTAATTTGATGTTTTATTGTTTTGGAATTTTCTCAGTTC
CTCAACCCTTGTTCTTTAATAAATTGTTCTAAACTTATAGGGATGATTTTGACTCTTCAT
TTTCCTTTTACATGTGGCGTTTCGATAGCCAAGCAGAGACTTAGATCGAGATATCAAGCT
GTCCTCGCAAGCTCATTGACTAGCATAGCAATATTGTCCTTAGGAGTTAATCTACTGATG
TTTCTTGCATTATCAGTGAGGCCCATTCTTTTGATTCGGCAGAAGAGATGTGGCAGATGA
AAGCATAGGATTGTAGTATACTTTTTCCTCATGTTTTCTTCGGAATCATTAAGCTGGATG
ACTGTCAGTACTGCTCCATTGAATTAGGAAGAAGAATGAACGAAAAGAGTAAAAGATAAA
TGAGAACGTGAAGTTGGTCCTCTAATATCAACCTTATTTAAAAGTATTTTTTTTCTACTA
TCATTCTCATACCCTAGAGAATTTGGAGAAAACCAAAGCACCCAATGCCATCGGAAGATG
TCGATAGTTCTTGGAATTGGTAAGATACAGCATTTTGCAGTCTGTACAATTCATGCCGAG
GAAAGTGTACAAAGTGTAGCCTACTAAAGAGAAAAAAATCCAAGTCTCAACTTGGCATTTG
AGATTGAGGGAAGAATGACATATTCTACTACCTCTGAAAAGAGAGTTCAGTAAATAATAG
TGTAGGCTTCAACTGGCTTGCTTGTGATCTAATCCTCAAAGCATGCTCTTCCCTGTATGG
CTGTATATTCATAGACTTTCTTAACCCAACAACTTTTCTTCCCTGAAATGCCAAAGTTAT
TCCCTGTATGGTTGTTTTGGTTTACTTCTGGATCTTTTTTTTTGGTTTGATACAACATAT
AGCACATGTAATCTCAAAATGGACTTAGGATGAGAGATGATGTAATCACTGGTTTTCAGG
GGACTGTTTGGGATGGGCTGTTGGGGTGCTGTTCAGGTGTGTTGTTCTATTGGGGGAATG
TAGTCTAGTTCTTTTGGTCATGTATTTTTTATTGCAGCCTCTGAACGGCTGTCCCATCTC
ATCTTGATTTGGCTATACTTGTATGTAGCCAGCCATGTTAGGAGACACCTTTGAGTCCTC
TTCTTTTTCTCATAAATTCCCTGTAAAACACTGCACTTCTACATCAATTTTAGCTAAATA
AAAAACAAGATATACCTGAAGGAAAAGGATTTAAAGAGGATACCACAACACTATCTAATT
AAGAATAACTCAAAGGGAAAAACAGAGAGAGCCAGAGATCTGTGTGCAAGAAAAAACTTA
TTCGCCTTCAGGATGGTAAAATCAAGATGCGCCTGTCACCCCACGTCGATCTATCACTAG
GACATGCTTCCACAGTGTTATTATTCCATTGATATGCAAAAGCTACATAAAAATCCGAAG
TACAGAGATCATAGAGTTTGACAACCACAAACGAGAAGGTTGATTTTTTTTAATAAGTTA
CTAATAGAAATTTGTTTACTTAGATCTTACCCTCCAAAAATCACAGCATAGAGCAACAAT
TCGCCTCTAAAAATGCTCAACTAGATTACACACACCTCCTAACTCAGAGCCTTACTACTT
CCAATTCCACCTAAATAATCATCATCTCAAGTGAGCGATACAAAGAACTTTAGTTGCCAG
TTTGTCTGAGCTCGGGACGGACAAAGAGTGATGCAAAGAACTTTAGTTGCCAGTTTGTCT
GAGCTCGGGACGGACAAAGAGTGATGCAAAGAACTTTAGTTGCCAGTTTGTCTGAGCTCG
GGACGGACAAAGTCAGCATCAGCAGGAACTGAAATGTCGACTGTTGGTCGCAATTGGGCA
CAAACTTCAATAGCTTCGTGCACAGGATCACATACGAAATTGGTCATAAGATCCATATTG
ATGGGAGCGTTATTGTCGACTGCCACCAATGCTTGCTGGGTGAGGTTGTAATCAAATCGA
GGGGCCCACT

>comp75266_c2_seq4 3193_3486
CTTCCATCGACAGTAAAATGAAATATCAAGAAAAATATAATGTTGGTTGTTGGAGGTTTC
GTGGGATGAATTACTTTTTTCTGGATTGGGTGAATTATTTATTAACAAAAAAATAAGAAG
TATAATCTAAAGCGTAGAGCGTTGAGTGTTGCAGAGTGAAAAAGAAGGCAGACCGAATTG
AGAGGCGTCTAGCATGAAGAAGCAACTCCAGGTCCCTCATTTTTTCCTCTTCACCACTGC
TCTTCTCCATGGTACTGGGGAATGAAATTGGGAGCAGTTTGCAATATCCTGTGGTAATAA
GCACGTGGCCCTTCTTGGAAGCTGTTAGAGCTGCGTGGAGGGCTGTGGACAGTGGGTTTT
CAGCAGTAGATGCAGTTGTGGAAGGTTGTTCTGCTTGTGAGAAACTAAGATGTGATGGTA
CAGTCACGGCAAAGGAAAGCAACGATGGAACTCTTTTCTGTGAAGATACATGGTTGGTAA
TTATTAAATGAAAGGAAGTGCTTCTTGCAATAAACACGGAAACATCTGCTCTCACTTGTT

GAAAAGAGGTATCGTGATGAAATGTCCAACACATCACTGATGTAGATTGAATAAGCGGAA
CAGCTAACAAAGAAGATGCTAGACATGTCGCTGGAAGAATGACCTGAATAGTAGTTGGAA
GAATGGCTGGTGTGCAGCAGGATGGGGCAGTTCTGACTGCTGAGTGCTCACAAAATGACA
CCGGAAAGAGATGTGGTGCCCGAACAAACCTAAAAGAGGCATACTACCATTAAAAGGATC
ACCAGAGTTAATGCTTGAAGAATGGCCAGCTATTGACTGAAGTAAGAGCAATCACCAGAA
GAGACGCAATGCTGCTGGAGCGGTCACCGAAAGAGGAAAGCTACCACCAAAAGGGTAACC
AAAAAATTGTGCAGTGTCACCAGAATGATCATTAGTAAAAGATTCTGCTGCCAGAACGGT
TACCAGAAGAAGAAAGATTAGCGAAATAGTGTATGGCTGGTAAATAGTGTAAGGCTGCCT
ATTAGTAGAAACTACTTGAAGTCTTCGCCAATATCATTGAAATAATAATACCATTAGAAA
TGAGAAACAAATTCTTCTTGAAAGAGTTGGTTTAGCAAGATACAAGAAGGGTTTCTCATA
TAGGATTTTTAGTTACGCGAAATAAGGAAATCTACCTAATTTCTGGGATTCCAAATGACA
TAATCATTTGAGTATTCTCTTGACTTTCAACAATAAGATAATCAATAAGAATATTGTGTA
CAATATAACTAAAGATTCTCTGGAATTTCATTCATATCACCACATCTACCTCATTTTATA
CGACCTATTTCAATGTATGAGGGTTGATTCAGTTGACAAGATAAGTCCTCAGTCCATTGA
TAGTCTGTTGTTGAAAGTGAAAATTATATATCAAGAAATACATATGAGAAATTAATACAA
ATGATTGTGCAAAAGAAGAATGTTCTTGACAGAAAAGGCAACAAGTCATAAGGTAAGAAA
TGACGCAGTTGAGTGTCTATGTGATGGTTTATGCAATATTATACTTGCATTGCTAGATAT
TCAGTTCATAAGTATTATTTTCATGGCAGTTGGGCCTGGTGGAAGTCCAGATGAGAATGG
AGAAACTACCATAGATGCCATGGTGATGAATGGGGTAATAGCATTTCTGTTTATTCTTGT
GTATTGATGCCATGGTGATGGATGGGGGTAATAAGTGTTGCTATTTATTCTTGTATATTG
CTTCATGATCACTTTGACCAAGGCAAATGCAATTTGCAATTCATTTTGGACTTGCTAAGA
CATGCATAGGTAACAATGGAGGTCGGCGCTGTTGCTGCTATGAGGTATGTGCCAGAGGGC
ATTAAAGCTGCGAAGCTAGTGCTGGAGTATACCAAACATACTATGCTTGTTGGGGATCAA
GCCTCAGCTTTTGCCATTTCAATGGGCCTTCCAGGGCCTATAAACCTAAGCTCAGTCGAG
TCAACAGAGAAGTGGATGAAATGGAAAGAAAATTTTTGCCAACCTAATTTTTGGAAAAAT
GTCTCCCCGGGGGATAATTGTGGTCCATACCATCCAAATGGTCCTTCAATAGGAAGATGT
CTGATGGCGAACGAGCTCCAGCCTAACGAATTTGGATCAGTTAATGTTGGTTTACATAGC
CATGATACAATATCCATGGCTGTCATTGACGAGATTGGACGGATTGCTGTTGGTACGTCA
ACTAACGGAGCCACATTAAAGATTCCCGGAAGGGTTGGTGATGGACCTATAGCAGGGTCT
TCAGCGTATGCAGATTCTGAAGTTGGTGCCTGTGGAGCCACTGGGGATGGTGATATCATG
ATGCGCTTCCTTCCATGTTATCAGGTTGTGGAGAGTATGAGATTAGGAATGGAACCCAAA
CTAGCTGCAAAAGATGCTATATCGCGAATTGCAAGGAAATATCCCGGCTTCATTGGGGCT
CTATTTGCAGTCAACAGAAGTGGTATTCATGCAGGTGCATCCCATGGTTGGACTTTTCAG
TACTCTGTCAGAAATCCTGGGATGAAAGACGTGGAAGTATTTACTGTTCACCCCTGAAAC
TGTGACTGCCACTGCCACTGGTGACTTCTTTAGCATACACAGTAATTTCTTTCTTTTCCA
TTGTGCCACAGTAATTTATTTGGATATGTAAAAGAGATAAGGCAAAATACATCGACAGAC
CATTAAACTTGTCTACGCTTTTCAATGTGACACTCCATCTTAAGTTTCTTCCATTTGAGC
ATTTAACCAGGTAATTACTGTGTCACTTAGACACAAAATGCTGACATGGCAAGGTAAGTG
TTTCTCACATTTTTCAAGCGCGTGAAATCATCCAAAAAAAAATAATCTCCACATACTTCT
TGTCGGAAAATGTATAGCTTATGCCGAAAATTTTCCGATCATCTTGTTAACCAAAATTGT
TTTTAGATTTCTTCATACCATTACTACTGTATCCTCTTTTAGATCTAAGCACCCCCCTAC
CACCTTTAAATCTGGCCACCATCACAAACTTTTCAAATTCCTTCTCATTTTTAGATTTGA
TTGATGCTGCAATTCTGGACGGAAAAGTAGAAGCGCCGCTAGCTGCCTACCTCGTCTTTT
TTATTTATCTTCCACCATGTGAGCAACCACAGGATGGCATGAACAAGAACAGCGGCAAGA
GCCCTCTCATTTTCACTCGTTCGACCAAGCTTCATTTTCCCAACAAAGTTTCGCTTTTTG
CTCAACAATTAAATTTGTGACTTGAATCTTAATCTTGATAAGAATGGACATTGGGCCTAA
CTCAACCCCAAAAGCTAGCTTATGAGGTGAGGATTGCCCAAGCCATATAAGGAGACCAAG
GACCTCAAATCCCTCCGATGTGGGACAACTCAACACCCCCCTCACGCCCAGATCTCTGGA
GCGTGGACAACATAAATGGGGGCCCAACATCGGTAACAATAATTGGGATGGGCCTGGCTC
TGATACC

>comp71496_c0_seq1 489_1463
CAGAGGGGTACGCACAACTGCACAAGTTAGCTCGGACAGAACCTTGGTATAACAATAGAA
GATAGAGAAGTTTATCACGTGCTATTCACGTGATCGATAGACAATTGGATTTTGAGAGTC
GCCTTTAGTTCGGGTTTCTCGTCTTTGGTGCTTTCCTTTTTGTGGAGTGTGAGATTTCCA
GGGAGAAAAAAGTTCCATATTATTATAATATCCCCATTACGTTACACTCTTCATCTTCTC
GTTTTCCTTAGCTTCGAGGGTTTCGTTTCCTCTGTAAATTCTCCCCCCTCCGATCTCTCT
CCGATTCAATATTCAATCCTAGGGTTTCGGGTACTTTCTGTTCCATCCGATTCGTAAATC

TACAAAAAGATATTTTTAACCCTAGTTTCAAATCTCGTGTGATGCGAAGAGCAATTGTGC
TTCTGAAGATCTTTAAGGATTTTGATTTGATTTGATTTGATTTGATTTGATCTGTTTTGG
TTTTCGCGATGGATGTGGATTTCAATGGTGACGTAGCGAGTTTGGATGCTGACCTATTGC
AGCTACCTGAGGTGTCACCTTTGGCTATTAAAACTAACCCTTATGTTGCCGAGAAGCTAT
TCGATCAGTGGTTTTCGCTCCCTGACACTGCCGCTTTGGTGAAATCTTTACTTAAGAATG
CCAAAGGTGGTGGTCCATTAAATGTGTCTGGTACATCTTCAGGCTCAAATGCTGCTGCAA
CTAATTCTTTGCCGTCCATGTTTCCTGCTGGAAGTACACCTCCACTTTCTCCAAGAAGTT
CATCTGGTTCCCCTCGAATCTCAAAGCACAGGGCTGGACCCTCTTCATTGGGGTCTCCTC
TGAAATTAGTGAATGAGCCAGCGAAAGAGCAAATACCACAGTTTTACTTTCAAAATGGTC
GTCCACCTCCAAATGAATTGAAGGAACGGTGCTTGTTTAGGATAAACCAATTTTTTTATG
GTCACACAGATGGACTACAAATGAATGAATTTAAACCAATTACGAAGGAAATATGCAAGC
TTCCATCGTTCTTCTCTGCAGCCCTGTTTAAGAAGATTGATGTTGACAGCACTGGCGTCG
TAACCAGGGATGCTTTTGTTGATTATTGGATCAACGGCAACATGTTAACAAAAGATATAG
CAACTCAAATGTACACAATCTTGAAGCAGCCAGACCTTAGATACCTCGCGCAGGATGACT
TCAAACCCATTCTTCGGGAACTCTTGGCAACACATCCAGGGTTGGAGTTTTTACAGAGCA
CACCCGAATTTCAAGAGCGTTATGCTGAAACTGTAGTCTACAGAATATTTTACTACGTGA
ATCGATCGGGTAATGGTCGTCTTACCCTCAGGGAGCTGAGGCGTTCTGACCTTATTGCCG
CAATGCAGCATGCAGATGAAGAAGATGATATCAATAAGGTCCTAAGGTACTTCTCATACG
AACACTTTTACGTGATATAT

>comp64986_c0_seq1 125_487
GTCAAATTCCTGTACTAGGGTTTTGCCTTTTAACTTGTTTTCTCAAACCTTCTTCACTCC
CTCTTCTGTTCGAGCCTGAGCCTCAACCTAGAGAGACAGAGAAAGCGAGAGAGAGGTTCT
CAAGATGGTGCAGCGACTCACCTACCGGAAGCGCCACAGCTATGCCACCAAATCCAACCA
GCACCGTGTTGTCAAAACCCCTGGTGGGAAGTTGATTTACCAGAGTACCAAGAAGCGGGC
TAGTGGTCCTAAGTGTCCTGTAACTGGAAAGAGGATTCAGGGGATTCCACACTTGCGACC
AACTGAGTATAAGAGATCCAGATTATCTAGGAACAGGAGGACTGTAAACCGTCCCTATGG
TGGAGTATTGTCTGGAAGTGCAGTAAGGGAGAGGATTATTCGAGCTTTCTTGGTGGAAGA
GCAGAAAATTGTGAAGAAGGTTTTGAAGATTCAAAAAGCCAAAGAAAAGCTGGCGGCCAA
GAGCTAAACTGAGAGTTTAGAGGGACTTTGTTTTAAGTTTTGGTGAAATTTTGACATTCA
AGTATTTACTAGTTGTGCACTACAAGTTGACCTGATTGATGCAACTTTTGAGGAGCATTT
TGCTTTAGTTCATTATATCCGACTTCATTCCCCATTCTATATAAATTGAGTTGTCAATCT
TGTCTTAATTTACCTAATATATCACACAATGGTTTTATCTTGCTATTTGAATCATTATTA
TGAGCAACTCAGTTGATGGTGAAAGAAATGTGCCAAAAGCAAGTAGATGTGTAAGCATTG
TCTGAATGTTGGGAGGTCATTGTGCTTGGATCTTGGTTAATGGGGTCTCTTGTTTGATTC
TGTTATTCATGAAGCTGCAACTAGGATAACTATGTATACATTGCTTAGTGATCCTAGTTG
CAAGTGAATCCAAAACTGGGTTTGTAGATATTGTGAGGATGAACATTGCATGCTCTGGTG
TAATACTCTGATTAAGTTAAAGGAAATAAGGTATAATGCTCTGATTCAGAAGGGTAATTT
TTAGAGGCCATGGATTATATACAAAGGTGGCACCATATGGTGTTTTTGTCATGTGCCCTG
ATACACATTTATAATGGTCCATCCAAGTATCTAGGACCTGGACTTCTCTTCTAAAGTATT
CCATCTAGAGGTTTCTTTGGCAACTTAATGAATTGGTTTGCTGTATCTGAAAACTATACC
GTGCTTATTTACTTGGCACTTCGACTATGATTTGGCTTGATAAATGAAACCTTTCACTAC
AGCTTGCTCTAGTAAGGTTGTCACCCAGGTGTATGAGTGGACTCTTTTCTGGGTCCAGAA
TCTTCAGGCCAAGGATTTTTTCTTGAGCCTTGAAATCATATAGTGTTGCAACCATCATGA
TATTCAGTACACCTGGATATTCTTCGTGGATTTTGCGATTCCCTTCAGTGAAGAAAAATC
TCTTTCTTCCAGCACTTCTAATCTCTCCCACAGTTCCTCACACGCTTCCTGCTGGGTAAC
CACGATCTTGGATATGCTGTCAAACAACTGAAAATATGGAAAGCACAAAGTAACAAAATC
TTGAAATTCAAATGTAAAAACACACGATTATATAATCTTACCAGTTGGATGTAACCGGTC
CAGAAACGTACTGTGGCTCTTTCATATGGATCTTCTTGCAAGAGTTTAGGTATGAGATTC
CAAGTTTTATCAATATATTCAATGATGACAGGTGATTCCACAACAGCTCTACCGTTGTGA
ATCAACAGGGGTACCTTTTTGTGGATTGGGTTGTGTTGGAGAAGCAATGGACTCTTGTTG
CTCAAGTCCCTCTCGCACAAGCTGGAAAGGAGATGATGCTGAGTTAAGAAGATGTTCTGG
AGCTAAATCGAATTATTGGAGCTTTGACGTCTGCATAAAAGCCAAGGAATTAAACCGGGA
TTGCTTATGGGGCTCATGGACTGGCGTTGCATACGAAAGTTGGAAGAAAGAGCAAGGGCT
GGAGCAAAAAAAGTACTAGTGCATCGCATAAGGCGTGGCATTAATTGTCGCATTAGTACA
AAATTTCGTCTGAAGATGGAGAAGTTCAGAAACTTGGAATTTTTGGGTCTGACAGAAAAA
AACACTAATGCTAGTCATCGTGCGTAGCGTCAGTACAAAATTTTATCAGACAAACTAAGT
TCGGAGAGTCTACAACAAAGCAAATTGCACTAATGCTACGCACCCTGCTTAGCATAGTGC

GACGCATGAAGTATAAAAAGTTTGTAGGTTTTGCCACTTAAGCTCAAAGATGATAGTTCT
GTCCAGATTTGTTCCTGCATGGTATAACTATGCTAGAAACACGTTTTTTTAGATGACTTT
TCACATTGTTATGTCCCATTTTAACACGGGTCAAACTAGAGTATAATATATTTGTAATTT
TGAAGATTTAATTAATTAAAGAGTCGTCACCTAATTATTTTAACGGTGAATTAGGACACC
AATTTTAGCTAAATAATACTTAAAATTAACTTTATTTTATTGTCTGC

>comp77607_c3_seq4 3_1151
AACTCCCTATAAATAGAAGCAAAAATATACAAGCAAAAAATCATCCACAGCTTTTCTTTC
ATTCAATTAGGCCAATGGCTTTTCGTTTGAGTCATCTGAGCCTTGCACTGAGCCTTGTGG
CTCTTGCACTTGCAGGTGTTGCCATATATAGGAACACTTATGAAGCGATGAGTAAAGGAT
TCCAAACACTTTCTCCAGAGTTAGATCTGCTGGGGTCAGCAGCCAGCATTTTAACCCTAA
ATAATGCTGAGCAAAATTCAGACAGCAAGTTAACTCAACCATTACCTCCATCCGCATGCA
TCTTCTCGGCTGTTCGAGCAGTTGTCAACAGTGCAATTGATAGAGAAAGACGCATGGGAG
CTTCTCTCATTCGTCTCCACTTCCATGACTGCTTTGTTGATGGTTGCGATGGAGGAGTTC
TTCTAGACGATATTCCCGGATCATTCCAAGGGGAAAAAACTTCACCACCCAACAACAACT
CAGCCAGAGGTTTTGAAGTCATAGAACAAGCTAAACAAAGAGTAAAAAATACTTGTCCCA
ACACACCTGTATCTTGCGCAGACATCTTAGCTATTGCTGCTCGGGATTCTGTTGTTAAAC
TAGGAGGACAAGGCTATAACGTTGCACTAGGGAGAAGAGATGCAAGAACGGCCAACTTCA
CTGGTGCTTTAACTCAGCTTCCAGCTCCGTTCGACAATCTAACCGTCCAACTAAGAAAAT
TTAATGACAAAAACTTTAATGCCCGGGAAATGGTGGCGCTAGCTGGTGCCCACACGGTGG
GTTTCACAAGGTGCGCCACCGCGTGCAACAGCAACTTCGTTAACCCAGCGGCACGTCTTC
AATGCAACTGCTCCGTCACCCAAAACGACACCAACTTGCAACAACTGGATACAACTCCAG
CTGTGTTCGACAGAGTTTACTTCCAGGACTTGAACAGGAACCAGGGTATACTTTTCTCGG
ATCAAGTTTTGACGGGGAATACCACCACTGCTGCTATTGTTACAACCTACAGCAATAATG
GTGCTGTTTTCCTTGGAGATTTTGCTGCTGCTATGATCAAGATGGGAAACTTGCCTCCCT
CACAGGGGGTTCAATTGGAAATTCGTGATGTTTGTAGCAGGGTCAATCCCAGCTCTGTGG
CTTCTATGTGAAAGAAAAACTTGAATTTGGAAAAAAAGGAATTCATGAACTCACTATTAG
TTGGTTGATGTTTTCTTAAAATAAAAGAATCACTGGTTCCAGAATGTGGTTCTAGTATTT
GTTGTAATATTTGGTTTCACCATTCTTGTGTAATCATTTGATTCCATGTATAAGCTCCAA
AATTAATGTACTCCTATATTGTTTTTCCAGTGTTAGAAAAGTTCTGAATCAGTTAAGGAG
CTGATCAGAGTAGTTTAATTTACTACTTTTAATTTATATGAATTTATTTGATTGGGTACA
AAGTATAAGAAAAAAATGAAGACATTTGAAATTTATGACGGAGAGATCGATGAGGACGTCA
CTCACCGTATAGGGGCGGGCTGCATGAAATGGAGGCTTGCATCTAGAGTTTTGTGTGATA
AGAAAGTGCCATCGATACTCAAAGTTAAGTTTTATAGAGTTTTGGTTAGACCGGCTATGT
TGTATGGGGCGAGTGTTGGGCGGTTAAGAACTCCCATATCTAGAGGATGAATGTAGCGGA
GATGCGGATGCTGAGGTAGATGTGCGGGCACACTAGGATAGACAAGATCAGAATGAAGAT
ACTCGGACGAAGATGGGCGTCACCCCTGTGGATGATAAGATGCGGGAAGCGAGGCTCAAA
TGGTTCGGGCACGTGCAAAGGAGAAGCATTGATCCACCAGTGAGGAGGTGTGAACGGTTG
GTCGTGGTGGGTACGAGAAGAGGTAGAGGGAGACCTAAGAAGTATTGGGGAGAGGTGATT
AGGCATGACATGGCTAGAATGCGGGTTTCCGAGGACATGACCCTTGACAGGAAAGTCTGG
AGGTCTAGAATCAGGGTTGCAGGATAGGGGCTCTTAGAGTCCTTCGTTCGCCTCACCCGG
GGTTGGGTGTGGGATAGTAGGGTTACCCGTAGATGACTAGTGTTTTAGCGTAGGTCCCCT
CTTGGTTGGTAATATGATAGTTGTCGCTTGTTATTGTCGCATGCGACGGTATTTATGTTT
CTTATGTTTACATAATCATTTACGTTATTTCTGCAGGATTACTTATGCTTTGTTTTGTTA
TTGTGTTTTGTCTTGTCTTGGTTCCTAACTTTATCTTTGTTTTCCAATGCAGCTTTTCAT
TCTGTCCTATGCGCCGAGGGTCGCATGGAAGCAGCCTCTCTGCCCTTTTGGGTAGGGGTA
AGGCTGTCTACATATTACCTTCCCCAGACCCCACCTGGTGGGAG

>comp80078_c3_seq4 193_2097
CTAATACAACGGTGGATATATCTTCTACAAAGACAAGAACTCTGTTTTTGTACTAACACG
CTATTGCCAATAATTGACTTAGACTCGTGTTTCTTTATATCTGAACATTTATCTGATCTA
AAAATACAATCTTGAATATACCCTTTAAAGGAAAAGTCAAGATCTTGAGTTTTTTTTAAG
GCATAAGAAATCATGGGAAAAACAACAAGGAAATTGGAAGTGGTTTCTCCTGTACCAGCA
GATATAGATATAGCTAATTCTGTTGAACCTTTTCATATTTCTGAGATTGCTCAAGAACTC
AATCTTAATCCCAAGCATTATGATTTATATGGCAAGTATAAAGGGAAGGTTCTGTTGTCA
GTGCTTGATGAGGTTGGAGGAAGTCAGGACGGGTATTATGTGGTGGTTGGAGGGATAACT
CCAACCCCTCTTGGAGAAGGGAAGTCCACCACCACGGTCGGTCTTTGCCAGGCTCTGGGA
GCTTTCCTTGATAAAAAGGTTGTCACATGTGTCCGCCAACCATCGCAAGGACCAACATTT

GGAATCAAGGGTGGTGCTGCAGGGGGCGGTTACAGTCAAGTGATACCAATGGATGAGTTC
AATCTTCACTTAACAGGAGATATTCATGCAATTACAGCAGCAAATAACCTCTTGGCTGCT
GCCATTGATACACGAATCTTCCATGAATCTTCTCAGTCTGATAAGGCTCTCTTTAACAGG
TTGTGCCCGCCAAATAAAGAAGGTAAACGAAAGTTCTGCGACATAATGTTTAGGCGTTTG
AAGAAACTTGGTATTGACAAGACGGCCCCTGAAGACCTTACTCCTGAAGAGATCAATAAA
TTTGCTAGGCTAGATATAGATCCCACTTCCATCACATGGAGAAGAGTTATGGATGTCAAT
GATAGGTTTTTGAGAAAAATTACTGTAGGTCAGGGCCCCGAAGAGAAGGGGATGGTGCGG
GAAACAGGATTTGATATTTCAGTTGCCAGTGAAATAATGGCAGTTCTAGCCCTCACGACA
TCACTAGCTGATATGCGAGAGAGGCTGGGGAAAATGGTAGTCGGGAATAGCAAGGCTGGT
GATCCAGTTACGGCTGATGATCTTGGAGTTGGAGGCGCTTTGACCGTCTTGCTGAAAGAT
GCCATTAACCCTACTCTTATGCAGACTCTTGAGGGCACTCCTGTTCTTGTTCATGCTGGT
CCTTTTGCAAATATTGCGCATGGAAATTCATCTATTGTGGCTGACAAGATTGCATTAAAG
CTCGTCGGACCTGGTGGCTTTGTGGTTACAGAGGCTGGCTTTGGTTCTGATATTGGGACA
GAGAAGTTTATGAACATCAAATGTCGATATAGTGGCTTGAAACCTCAATGCGCTGTTATT
GTGGCCACTGTAAGAGCCCTTAAAATGCATGGTGGAGGACCTGACGTCACAGCTGGAAGG
CCTCTTGACCGTGCCTATGTAACTGAGAATGTTGCACTAGTGGAAGCCGGTTGTGTGAAT
CTGGCCAGGCACATTTCAAATACAAAAGCTTATGGTGCAAATGTTGTGGTTGCCGTCAAT
GCATTCACAACTGATACTGAAGCAGAACTAAATGCAGTTAAAAATGCCGCATTTGCTGCA
GGAGCATTTGATGCAGTAATTTGTACTCATCATGCTCATGGTGGCAAAGGAGCGGTGGAT
CTTGGAATTGCAGTTCAGAAGGCCTGTGAGAATGCAACACAACCACTAAGATTCCTGTAC
CCGCTGGATATAGGTATAAAAGATAAAATAGCGGCAATAGCAAAATCATATGGTGCTGAT
GGAGTAGAGTACTCTGAACAGGCTGAGAAACAGATTGAGATGTACGGCAAACAAGGATTC
TCTAATCTGCCAATTTGCATGGCTAAAACACAATATTCATTTTCGCATGAATCTGCGAAG
AAAGGCGCTCCAAGTGGATTCATCCTGCCCATCAGGGATGTTAGAGCAAGCATTGGCGCG
GGATTCATTTATCCTCTTGTTGGCACAATGAGCACAATGCCTGGTCTACCTACGCGTCCT
TGTTTCTACGAGATTGACATTGATACTTCTACTGGAAAGGTAATTGGTCTTTCTTGAGAA
TCTTGAGCCGAGGGTCGATTGGAAACAACCTCTCTGCCCTTTCGGGTAGGGATAAGGCTC
GTCTACATATTACTCTCCCCAGACCCCACTTTGTGGGACTATACTGGGTAGTTGTTGTTG
AGAATCTTGGCAGGTTGCACTGTGTTTTGGCAGAAGTGATGAGTTTGTCGAGTTCAGACT
TCCTCCTTCTACATATTCGACATTGATTGAATAAAATGAGTTTAGTATATCCAGATTTCG
CGTCATGAGGAGTAGTATACTACTCGATTTGTAAAACGGACAAAAATTCTTAGAAAGTGA
TTTAGAACCAGTGTAGATTATGTTGTGAATCTTGCACAAACATTTCAAAATTTTGTCATT
TCTGTTAATGTGCTAATCTTTTAAACTTTGGGCTATTGGATTTCGGCCCAAGTTAGTCCT
ATTCTTGGGCTGGTGTGAGATCTAACTAGCAGCCCATATGGGGCAAGTTGTGCTTTACCA
AGTAAATTGGCTGGGCCGTATGCAGGACTGTGCTTGGGCTTCTACCTGTATACGTAAGGA
AAAATGACACTGTGTAGCCACTCTCAAAA

>comp72702_c0_seq4 152_2431
GCATATATCACTCATTTTCCCCCACAGACCTTAATTTCTTGTCTATCAAACTGCAAACTT
TATAGCCATTTTTGTATTGCTTTCTTGCCAGATCCCCCCACCACCCTCATATAAGACTTG
TAAGCTAGAAGGTTAGAAGAAGAAATTAAGAATGTGGAAATTGAAGATTGCAGAAGGACA
AGATGGGCCATATTTGTTCAGCACAAACAACTACGTTGGACGTCAAATATGGGCTTTTGA
CCCAAATGCTGGAAGCCCAGAAGAACGGGCCGAGATCGAAGAGGCCCGTCAACAATTTTG
GAACAATCGCTTTAAAATCAAGCCTAGTGGTGATCTTCTTTGGCGAATGCAGTTTCTTCG
AGGGAAGAATTTCAAACAAAAGATTCCAGCAGTAAAAGTAGAAGAAGGAGAAGAAATTAC
AAATGAAGTTGCCACAACTGCATTGCGTAGAGCTGTTCATTTCTTTTCAGCATTACAGGC
TAGTGATGGTCATTGGCCTGCTGAAAATGCTGGTCCTTTATTCTTTCTTCCACCTCTTGT
TATGTGTATGTACATCACTGGCCATCTTAATACGGTTTTCCCAGCTGAGCATCGTAAGGA
AATTCTTCGGTATATATACTGTCACCAGAATGAAGATGGTGGATGGGGTTTGCACATAGA
AGGTCACAGTACAATGTTCTGTACAGCATTGAGTTATATTTGCATGAGGATCCTCGGGGA
AGGACCGGATGGTGGCGAAAACAATGCTTGTGCTAGAGCAAGGAAATGGATTCTTGATCA
TGGTAGCGTCACCGCGATTCCTTCCTGGGGTAAAACATGGCTCTCGATTCTTGGAGTTTT
TGAGTGGTTAGGGACCAATCCAGTGCCCCCCGAGTTTTGGATTCTTCCATCTTTTCTTCC
CATACATCCAGCAAAAATGTGGTGTTACTGTCGAACCGTCTACATGCCAATGTCTTATCT
CTATGGGAAGAGATTTGTTGGTCCAATCACACCTCTCATTTTGCAACTGAGGGAAGAGTT
ATATGATCAACCATACAATGAAATTAATTGGAGAAAAGTACGCCATTCATGTGCGAAGGA
GGATCTCTATTACCCTCATCCATTGGTTCAAGATTTGATGTGGGATAGTCTCTACATATG
TACCGAGCCTCTATTGACTCGTTGGCCTTTCAACAAACTGAGAAATAAAGCTCTTGAAGT

TACAATGAAACACATACACTATGAAGACGAGAGTGGTCGATACATCACCATTGGATGTGT
GATAAAAGTATTATGCATGCTTGCTTGTTGGGTTGAGGATCCCAACGGCAATTATTTCAA
AAAACATCTTGCTAGGATCCCAGACTATTTATGGGTAGCTGAAGATGGAATGAAAATGCA
GAGTTTTGGTAGTCAACTATGGGATACTGCCTTTTCTATTCAAGCACTATTGGCCAGTGA
GATGAATGATGAGATGTCAGATACTCTTAGAAAAGGACATGACTTTATAAAGAAATCTCA
GGTGAAGGCCAACCATTCTGGTGATTTTAAAGGGATGTATCGGCATATCTCAAAAGGATC
ATGGACTTTTTCAGATCAAGATCATGGATGGGGAGTATCTGATTGCACTGCCGAAGCATT
AACATGCTGCCTTCTCTTCTCTACAATGCCCTTTGAATTAGTTGGTGAGGCAATCGAACC
TGCACGACTATATGACGCGGTGAATTTTATTCTTTCCTTACAGAGCAAAAATGGGGGTTT
ATCAGCATGGGAGCCTACAGGGGCCTCAGAGTATTTGGAGTTGCTCAATCCTACTGAATT
TTTCGCAGACATTGTTATTGAGCACGAGTATGTTGAGTGCACTGGCTCGTCAATCCAAGC
ACTTGTTCTGTTTAAGAAGCTATACCCTGGACACCGAACCAAGGAGGTTGACAATTTCAT
TGACAACGCAGTTAAATATTTTGAAGATGTACAGAGGCCTGATGGGTCATGGTATGGTAA
CTGGGGTGTGTGCTTCACATATGCTTCCTGGTTTGCTCTTGGAGGGCTTGCTGCAGCAGG
CAAAAGTTACAGCAACTGTGCAGCTGTCCGTAAAGGCGTTGAATTTCTGCTAAGAACACA
AAGGCCTGATGGTGGTTGGGGAGAAAGCTACCGTTCTTGCCCTGACAAGGTATACAGAGA
ACTTGAAACAGATAACTCAAATCTTGTACAAACTGCATGGGCATTAATGGGATTGATTCA
CTCTGGCCAGGTTAATAGAGATCAGAGGCCCCTCCACCGTGCGGCAAGGCTGTTGATTAA
TTGTCAGTTGGAAGATGGTGACTTCCCACAGCAGGAACTAACTGGAGCTTTTTTGAAGAA
TTGCATGATGCACTATGCTTTATACAGAAATATATTTCCATTGTGGGGTTTGGCAGAATA
CCGCAAAAAATGTCTTATTACAAAACTACTAAATATGTTTGAATGCAACCTTCTTCAAAGA
GATATATAGTATGCCATTTGGTCCTTACAAAATTTTTCCAGAAATAATGTAGTTTGTTTT
CTTGTGTTAATGTAAGTGATAAAATATTGAGTCGAGGGTCGATTGGAAACGGCCTCTCTG
CCCTTTCGGGTAGG

>comp82078_c0_seq1 3_1277
ATCATGCACAAGGTGTTGCATTAGGTGCTCAGAGACTTAAATGTACTGCTACGATTGTCA
TGCCTGTCACCACACCAGAGATCAAGATTGAAGCAGTTAAGAACTTGGATGGTAAAGTAG
TTCTACATGGTGACACATTTGATAAAGCTCAGGAACATGCTTTAAAGTTGGCTGAAGATG
AAGGTCTCACATTTATCCCGCCTTTTGATCACCCAGATGTGATCATAGGCCAAGGTACAA
TTGGAACGGAGATTAATCGTCAACTTAAAGATATATATGCAGTATTTGTGCCTGTAGGAG
GAGGAGGTTTAATAGCTGGTGTTGCTGCATATTTCAAAAGGGTTGCTCCTCATACAAAGA
TTATTGGAGTTGAGCCATTTGGTGCAAGTTCAATGACACAATCTTTGTACCATGGAGAAA
GAGTAAAGTTGGAACAAGTTGACAATTTTGCAGATGGCGTAGCTGTTGCACTAGTTGGTG
AAGAAACTTTCCGTCTTTGCAAAGATTTAATAGACGGAATGGTCTTAGTCAGTAACGATG
CTATTAGTGCAGCAGTAAAGGATGTGTACGACGAAGGAAGGAACATATTAGAGACATCAG
GTGCACTTGCCATAGCTGGAGCTGAAGCATACTGCAAATACTATAACATAAAGGGCAAAA
ACGTTGTAGCAATTGCTAGTGGAGCCAATATGGACTTCAGCAAACTAAAATTAGTCGTCG
ATTTAGCAGATATTGGCGGACAGAGGGAAGCTCTGCTGGCTACTTTTATGCCAGAAGAAC
CAGGAAGCTTCAAAAAATTCTGCGAACTTGTGGGACCTATGAATATTACTGAATTTAAGT
ACAGATATAATTCTGGAAGAAAACAAGCTCTGGTACTCTACAGTGTTGGTGTTAATACAA
AATCGGATCTTGAGTCAATGTTGGAGAGGATGAAATCATCACAGCTGAATACTGTCAATC
TCACAAATAATAATTTGGTCAAAGAACATCTAAGGCATTTGATGGGAGGCAGATCAGAAC
CAAGTAATGAGATTTTCTGTCAATTCATATTTCCTGAGAAGCCTGGAGCTTTAAGGAAGT
TCTTAGATGTTTTCAGCCCTCGCTGGAATATAAGTTTGTTCCATTATCGTGAACAGGGAG
AACTTGATGCAAGTGTGTTAGTTGGATTCCAAGTTCCAAAAGGTGAGATAGAAGAGTTCC
GAGTTCAAGCTAACAATCTTGGTTATTCATATGAAATCGAGAGTCTCAACGAGGCTTCCA
AACTTATAATGGAGTGAACAAGATTGATGTATTACTTAGTACTAGAGTATTTGGCTACTA
CTGATAGAGCAATAAAGTTGCAGCTATTGCAATTCGAATAATTTATGAATCAGTAGTAGA
TTTGGTAGGAATAGTGAACCTTTGTGTCTTATCTAATGGGCTTCAGTTCTATGAATAAAA
AGTTTATTATATCAACTACTTTTCTAAATATTAAGTTGGAGTGGCATTTTACTGAATTAC
TATTCTTGGTATGTCCTTGAATTGAAAAATCGAGAACCTTTGGTAGGAAAAGACTAATTT
GAAATAACTTTGATACTAATCAGAGGTTTGGTCCTTCAAGATTTTTACAATTATTTTTCA
CTAGCTAGAAGCTAAGAGCAAGTCATGCTTTTTACACATCAACAACAACAACAACTACCC
AGAATACTCCCACCAGGTGGGGTCTG

>comp78978_c0_seq3 374_2785
TAGAGAAATAGAGGAGCCTCTTCAGCTTTAACTTGGGCTTTGTTCTATCCGTCTAAAATG

GTGGATTCATACTTAGGTACTGTTTAAGACTCAATTTTTTTTTCACACCCAGTTCATTTT
CTTGATTTTTAGATGCACACCCATTTCCTTGTTTCTAATTATTTTTATCTATTTTTTGTG
TTTTTTGTTGTTTAAAGATTGGTCCTTTTTTGGTTCTTGTTTTGTGGTTTTATGCCATTG
ATGAGCTTGTATTGAGAATTTTTTTAGCTTTGAAATTCAGGTGTAACAGAATTGTAAAGC
TAGGATTTTGGTAAAGTTGAAGGGTTTTTAGTAATAGTTGACTATTAGAGTTGAATTTTG
GAGAAAAAATCAGATGGGTGGTGACAATGAGGAAGGAAATAGTGATATGGTGCAAAGACT
TCAATCATCATTTGGTACATCATCTTCTTCACTCCCTAAACAACATCAATCTTTATTATC
AATGAACCAATTGGACATACCTCAATTGACTAGTAGTTCCCAATTTCGTGGTCAAATGAA
GCAGTTTTCCCCCAATTTTAGTGTTGAAAATAGTACTTCTAAAAGAGTAGGCATACCGCC
TTCTCACCCTCAAATGCCCCCTATTTCACCTTACTCTCAGATCCCTGTGACAAGGCCTGT
GAATCAGCAAATGGGAATGCAGAATTTTACTAGTCCAGGGCCATCTCATTCACGACCTTT
ATCACAACCGTCGTTTTTCTCATTGGATTCTTTGCCACCCTTGAGCCCTTCACCGTATAG
GGAATCCTCGTCAACGTCTATGTCTGACCCTATATCAGCTGATGTGTCAATGGGTGATCA
GGATGGCAATTCACATTCTTTATTGCCCCCCTCACCTTTCACTAGGTGTAACTCGTCGAG
GGCAGGAGAGAGTCTTCCTCCTCGTAAGGCTCATAGGCGGTCAAATAGTGATATCCCGTT
TGGCTTTTCTGCAATGATGCAGTCTTCACCGCCTCTTGTTCCGTTAAGGAGCCCAGGTTC
TCTTGAAAGGTCAGTTCCTTCAAGGGAAAAACCAATTCAGTTGGTTAAACGGGAAGCTAT
GTGGGATAAAGGAAATGATAGCAATGCTGAAGGGATGGGTGAGAGGAAATCTGAAGGAGA
AGTTGTGGACGACCTGTTTTCCGCGTATATGAACTTGGACAACATTGATGCATTAAACTC
TTCCGGGACTGATAACAAGCTGGGCAATGAGAATCGTGAAGATTTAGATAGTAGAGCGAG
TGGTACAAAGACAAATGGTGGTGATAGCAGTGATAATGAAGCTACAAGCAGTGTGAATGA
CAGTGGCAGCAGTATGCAGAGGTTAGGGATATCTTCTTCTGTTGAGAAGAAGGAAGGGAT
CAAAAGGAGTGCTGTGGGAGATATTGCTCCGACCACAAGGCACTACAGGAGTGTTTCGAT
GGATAGTTTTATGGGGAAGTTAAACTTCGTTGATGATTCACCAAAGTTGCCTCCGTCTCC
TGGACCACGCCCTGGCCAACTCTCACCGACCAGTTCGCTTGATGCAAATTCAAATAGCTT
CAGTTTGGAAATTGGTAATGGTGAATTTAGTGGAGCTGAATTGAAGAAAATTATGGCAAA
TGAGAAACTTGCAGAGATAGCCTTAGCAGATCCAAAGCGGGCCAAAAGGATTTTAGCCAA
CCGCCAATCTGCTGCTCGTTCAAAAGAGCGAAAGATGAGATACATTGCGGAGTTAGAACA
CAAGGTGCAAACACTGCAGACTGAAGCCACCACATTGTCTGCTCAACTGACACTGTTGCA
GAGAGATTCTGCTGGGCTAACGAGCCAAAACCCACGAGCTGAAGTTTCGTTTGCAAGCCAT
GGAGCAGCAAGCTCAACTCCGTGATGCACTGAATGAAGCATTAACTGCTGAAGTACAACG
GTTGAAGCTTGCATCCGCTGAGATAAGTGCAGACGCTGCCAAGTTTCAGCAGCTTTCTCT
CAATCCTCAGATGTTCCAATTGCAGCAACAGCAGTCAACCCAGCTAAACATGCATCACTT
GCAGCAGCAGCAACAACAACAACAGCAGCAACAGCAACAACAGAATCAACAACAGCAGCA
GCAAATAGGCGGCCAAATGGGTAGCGGAAATTTAAATAGGGCAGCTTTAATGGGACAGAC
TGGCCATCTTCCCATGTTGTCCGGACAAGCTGCTGCAGCTGTCACCCAGTTCAATTTGCA
ATCTCAATTTTTGAATTCGCCGCGGCAGAAGGCAGGTCTAATGCAGGGGAATCAGTTTCA
TACAGGGAATTCCCATGGCCAGTCCTTGCAGGGTATACAGGCAATGGGAATGATGGGATC
TCTCAACATGAGCTCTCAGTTAAGGGCAAATGGTGCCCTTGCATATGCACAACAAAGGGT
CAATCAAAATCAGTTGAGGCAGCAGTTGTCCCAGCAGAATCCTCTCACCACTACGCAGAA
AATATCGGTTCAGAACCTTCCAAGGACGTCATTTATTAACCCTCAGTTACCTGGATTGAC
TCAGAATGGACAATCTACAATAATGCAGAACAATTCATCACAGCAGCAGTGGTTGAAGCA
AATGCCTGCTATTTCTTCTCCTAATTCCCCCTCCTATCGTCTTCAACCGCAGAGGCAGCA
GCAACCAATGCTTATGCAGCAACAATTAACTTCATCTCAACAGTTGCATCAAAATTTAAT
TGGTTTGAACCCACAGCAACTATCCCAAATTGTACAACAGCAACAACAGATTGGCCACCC
TCAGATGCATCAACAGCAACAGCA

>comp89465_c0_seq1 218_1237
TTAAAACATTAAAAAAACATGCGATAATTCAATTATTTTTCTTTACGTGAATCATTCATT
CAAGATTTAATTTGGCAAGGATTAATGTGAGAGAATCACTTCAAAGAACAAATAAACTGA
GGTCTATAAATACCCAAGAAATTCCAATTAGCTTATTCATTCCAAAAATTCAATTACCTT
ATTAAGAGTATTAGATTTAAGAGTAAAACCCTAAATTATGGCTTTTATGTTCAATTGGAG
GCTCAATTTTGCTATTCTACTAGCATTTGGATTGTGTGTTTCTCTAGTCACATCTCGCAC
TTTGTACGAAGCATCAATTACTGAAAAGCACGAGCAATGGATGACTCAGTATGGACGCGT
ATACACAGATGATACAGAAAAGATAAATAGGCTCGAGACGTTCAAACAAAATCTCGAGTA
TATTGAATCAATCAATAAGGATGTAACGCGAACTTATAAATTGGGCATCAATCAATTTGC
TGATATGACAAATGAGGAATTCAAAGCAATTCATAATGGATACAGAATATCATCTCAGCA
GAAAATAAGAACTACATTTTTCCAATACGAAAATGTGACAGTTCCATATAGTATGGATTG

```
GAGAAAAAAAGGTGCAGTTACAAAGATTAAAGACCAAGGACAATGTGGATGTTGTTGGGC
ATTCTCTGCTGTGGCTGCTACAGAAGGAATTAACAGGATTAAAACAGGCAAATTAATTTC
ATTATCTGAGCAAGAACTCGTTGACTGTGACACAAGTTCAAATGAAGGCTGTGAAGGAGG
TCTCATGGATGATGCATTTAAGTACATTATTAAAAATCATGGACTTACTTCAGAATTTAA
TTACCCATATAAAGGAATCGACGGTACTTGTAAAACTGAAAAAGAATCGAATCATGTCGC
GAAAATTCGTGGTTACGAAGATGTTCCAGAAAATAGTGAATCGTCTTTGCTTAAAGCTGT
TGCAAATCAACCTGTATCTGTCGCGATCGATGCTAGTGGATCGGACTTTCAGTTCTACTC
GAATGGTGTTTTTACTGGAAAATGTGGAATTGAGTTAGATCATGGTGTTACAGCAGTTGG
TTATGGTGCAACTAAAGATGGTACAAAATATTGGATTGTGAAGAATTCATGGGGTGTTAG
TTGGGGTGAAAATGGATATATTAGAATGAAAAGAGATATTGTTGCTAAAACAGGACTTTG
TGGAATTGCTATGAAAGCTTCTTATCCAATTGCTTAAACATGTTTACAATTTTGATTAGC
CTCTAGTGCCTGAGCTAGGACAATTATTTAATTCTGATTATTATTATTCATTTGCAAATG
TGATGTTGGATTTTGTTCAATGTAAAGAGGATTCTAACACTGACAAATTAAATGCATTGT
CCTTGCGCGTTTCCAGTAGCTTTAGTGCATTACTCAGCGTTAGCCTTGCGTTTTCTTAAA
AAAA

>comp79295_c0_seq1 283_3321
GGGGACTAGAAACCAAAAAGGAAAGAGAGCTTTTTCACACCCTAAAACACAAGGACCACC
ACAAAACTACCTATCTTAGTCTTTCTTTTTCTCTCTCTCGTGAATATAGTTACTTCATTT
TCCATCCACCTTGTAAAACATGAATATCTAACAATAATCCCAAAAACTCAAGATTCATTC
ATGAAAACATGTACTCTACTACCTAAAATTCACACCCTTTCTCTAACTATTCTCAACTAA
ACATTTGTTTTCTTCTCTTGTTGTAGGAACTAAAAAAAACATATGAAAATGTGGTTTTTT
TCTTGTCCTTCTTGTTTTCTTATCTTCATATTTCATGTTGGTTTATTGTATGGATCTTTA
GCTGATGAAACTTTACAACTTAATGATGATGTTTTGGGTCTCATTGTATTCAAATCAACT
CTTCTTGATCCTAACTCTAAACTCTTATCTTGGAATGAAGATGATAACTCTCCTTGTGCA
TGGGAATTCATCAAATGCAATCCAATGAATGGTAGAGTTTCTGAACTTAACTTAAATGGT
TTAAGTTTATCAGGAAAGATCGGTAGGGGTCTCGAGAAGTTGCGGTCATTACAGGTATTA
TCTTTATCAAACAACAATTTCACTGGTCCTATTAGCCCTGAGTTATCTTTGTTAACAAAT
CTTGAAAATCTTAACCTTAGTCAAAATGGACTTTCAGGAAATATTCCTCCATCTATTTCA
AAAATGACCTCCTTACAGTTTCTTGATCTCTCTGAGAATTCGTTATCTGGACCTGTCTCT
GATACCATATTTGATAACTGTGGTAATTCGCTACGTTATCTTTCTTTATCTGGAAATTTT
CTTGAAGGTGCATTTCCTACCACAGTTTCCAAATGTAACAATTTGAATCATCTCAATGTT
TCAAGAAACCATTTATCTGGCGACCCGGGATTTTCTGAGGGACTCTGGGGATTGACAAGG
CTAAGAACATTAGATCTTTCACATAATGAACTCTCTGGATTAGTACCAAATGGTGTTTCA
GTGTTACATCAGTTGAAAGAGTTGTTGTTACAAGGAAATCAATTTAGTGGAAAGTTACCT
TCTGATATTGGATACTGTCCACACTTGAATAAATTAGATTTGAGTGAAAATCTATTCACA
GGAGCAATCCCAGAGTCAGTACAAAAGCTCAATGCTCTTTCTTTTCTAAGTTTATCCAAC
AATATGATAAATGGAGATTTCCCTCAATGGATAAGTAACATGAGCAGCTTGGTTTACTTA
GATTTTTTAGGCAATAATTTAGAAGGGAAATTGCCTGATTCAATAGGGGACTTGAAAATG
TTGAAATACTTGAGTTTATCTGGTAATAAGTTGAGTGGACATATTCCAAAATCTATGGTT
TATTGTACTAGTTTATCGACGATTCGGCTAAAAGAAAATGCCTTGACTGGTAGCATTCCT
GAGGGACTGTTTGGTATAGGATTAGAAGAAGCAGATTTTTCAAGAAATGAGTTAAGTGGT
TCAATTCCTCCTGGTTCTGGCAAATTGTTTGAATCACTTCAAATTCTTGATTTATCAGGA
AACAATCTTACTGGAAATATTCCAGCTGAAGTTGGACTTTTTTCCAAGTTGAGATATTTG
AATCTTTCTTGGAATAATTTTCAATCAAGATTGCCTCCTGAAGTTGGATATTTTCAGAAT
CTAACGGTGTTAGATCTTCGATATAGTGCTTTAGTTGGATCAATTCCTGGTGATATATGT
GATTCTGGTAGCTTAGGAATTCTTCAGCTTGATGGAAATTCATTTACTGGACCTATTCCT
GATGAGATTGGAAATTGTTCATCCCTCTACTTATTGAGTTTCTCTCATAATAACTTAAGT
GGCTCAATACCAAGGTCTCTTTCAATGTTGAGGAAGCTCAAGATTTTGAAGCTAGAATAT
AACCAATTGAGTGGTGAAATACCACAAGAGCTTGGCAAATTGGAAAATCTTCTGGCTGTT
GATATATCCTATAACAGGCTCGTTGGACGGCTTCCATTAGGTAATATATTCCAGAATTTA
GACCAGAGTTCATTGGAAGGGAATTTGGGCATTTGTTCACCTTTGTTGAAAGGTCCTTGT
AAGATGAATGTGCCAAAGCCTTTGGTTCTTGATCCTTATGCTTATGGAAACCAAATGGGA
GGTCAAAACCGGGGCGATGAGACTTCAAGAAGCAACAGGAAAAGGTTCAAACACCATAGA
TTCCTTAGTATTTCATCCATTGTTGCAATCTCTGCTGCAGCTGTGATCGCGATTGGAGTA
ATGGTGATAGCCTTACTAAATGCTTCTGTTCGAAGGAAGATTGCGTTTGTTGACAACGCC
TTGGAAAGTATGTGCTCAAGTTCTTCTAAATCCGGGAGCTTAGCAACTGGAAAGCTAGTG
TTATTGGACACCAAATCGTCTCCGGATTGGACTCATACCAGTCTTGAATCAGTCCTAAAC
```

AAGGCATGTGAAATCAGTGAAGGTGTTTTCGGGACAGTTTACAAGGCTCCATTGGGAGGG
GAAGGAAGATCAGTAGCTATCAAGAAGCTTGTGACATCAAAGATACTACAATACCCTGAG
GATTTCGATAGAGAAGTCCGAGTTTTAGCAAAAGCAAGGCATCAAAATCTGATATCCTTA
AGAGGGTATTACTGGACTCCTCAACTTCAGCTTTTAGTATCAGATTATGCACCAGAAGGA
AGTTTACAAGCCAAACTACACGAAAGGCCATCGTCTTCACCTCCACTATCTTGGTCCACT
CGATTCAAGATTGTGCTCGGGACAGCCAAGGGACTAGCACATTTGCACCACGCGTTTAGG
CCAGCAATCATTCACTACAACATAAAGCCTAGCAACATCCTCCTTGACGAGAATCTCAAC
CCGAAAATATCAGATTTCGGTCTAGCAAGGCTCGTGACAAAGCTCGATAAACACATGATA
AGCAACAGGTTCCAGAGTGCACTAGGCTACGTAGCACCTGAATTGGCATGCCAGAGCTTA
AGGGTGAACGAAAAGTGTGACGTTTATGGTTTTGGGATGTTGATTCTTGAAATTGTGACA
GGGAGAAGGCCAATTGAGTATTGTGAAGACAATGTCTTGATATTGAATGATCATGTTAGA
GTGTTGCTTGAACAAGGGAATGTGTTAGATTGTGTTGATCCATCATTGGATACATATCCT
GAAGATGAAGTTTTGCCTGTTCTGAAATTGGCTTTGGTATGCACTTCTCAAATACCATCA
AGTAGGCCTTCAATGGCTGAAGTGGTTCAAATCTTGCAGGTCATCAAAACACCTGTTTCT
CAAAGAATGGAAGCATACTAAACATGTCACAAACTTCATTGTCTTTTTATTTGTGTCCTA
TAAATCCTATTGTTATTATCTTAAGCTCCTAATTTGGTTTTTTTATTACTAGATAGTGAT
AGAGAGTTTGGTACTTCTCTATCATTTGTATGAAAAATATGGGAGGGTCCAGTCACTTCA
ATCTTGTTTGTTCTTTTTTCTTTAACTTATCCTGATGAAGTTGATTTGTTTTCACTTATC
TGAAAATCATTTTGGACGTAAATTTACTGGTTTATAACGTTGGATATAACGTTGCAAGTA
ACTTGTTATGTTTGTATTTGCTCTATTGTACCTAAAGCATTGCCAGAAATCACTTTACAG
TATACAGAATGGAA

>comp56752_c0_seq1 118_1230
CTCAATGCCAGTCTCCTTTTATTAGATTTTTGTTTTGTTTAACTGGACTCTCACGCCCAC
AGAAAACAGCAAGGATCTGCACAATCTACCGTGTCTGTTAAAACGTAGAATCTGACCATG
AATAACCACAATGTCCAAGCTTGTTCTTACTGCTATAGAGCTGTAGTATTAACATGCCTT
GTCATTGTAGTCTTCGCGCCTGGAATTTCAGGTGAATGTACTTCCAACATCAAGGTTGAT
CAACCACGAAACACCAAAAACAGTAGTGATTCCCTCAGATATAGACTCATATCAATAGTT
TCAATACTGATTGCTGGTGCAATTGGGGTCAGTCTCCCACTTTTGGCAAGGAAAATTGAA
GCTCTGAGGCCCGAAAATGATATTTTCTTCATGATCAAGGCCTTTGCTGCCGGTGTCATT
CTAGCCACTGGCTTCATCCACATATTGCCTGATGCATTTCAGACACTAACATCACCTTGC
CTGCAAGGCATGAGCCCTTGGGGGAAGTTCCCTTTCACAGGTTTCTTCGCCATGGTCGCT
TCTATTGGATGTTTGATGATTGATACATTTGCAACGAGTTTTTACCAAAAGAGGCACTTT
CACAGAGTTAAGCAGGTCAATATTGTTGACGAAGAAGCAGCTAGAGATGACATACAGCTC
AGTCATAGTCATACCACCCATGTCCATGGCCATGCACATGGGACGACTTATTCCATCGGA
TCTGATCAAGAATTGATTCTGTCTGAAAATATACGAAATCGCATCATATCACAGGTGTTG
GAGCTAGGAATTCTGGTCCACTCCATAATAATTGGAGTTTCCTTAGGTGCTTCACAAAAT
ACCCAAATGATAAAGCCTCTATTGGTTGCTTTGTCATTCCACCAATTCTTTGAAGGCATG
GGGCTTGGAGGCTGTATTTCACAGGCAAAATTCAAGTCTATATCAACAGCAATCATGGCA
ATTCTTTTTTCTCTCACAACACCAGCAGGAATTGGGATTGGGATCGGAATATCGAGGGTG
TATAACGCCCATAGCTCTATTTCTCTAGTAGTCGAAGGGATTCTGAATTCTGCGTCTTCA
GGGATTTTAATCTACATGGCCCTCGTTGATATACTAGCATCAGATTTTATGAATCCAAAG
ATGCAGAACAATGTCAGGCTTCTGTGCGGGGCACACATTTCACTTCTTCTTGGAGCTGGG
TGCATGTCTGTGATGGCTAAATGGGCATGAATAATATTTCAACAAAATGCTTATGTTGAC
ATTCTTTTTTGTTTACAATCTGAAAGCAGGTCAGGTAGGAACTCAGTTGTATTAGTTTCA
TTAATATAGCTCGCTATCGTTTCTTGAATAAACGGCTATCATCAATATATTAAATACTCC
ATCTGTTCCAATTTATGTGAATGGCACGAAATTTAATAAAAAATAAAGAATTTTAAAATT
TATGGTTCTAAACAAATAAAAAAGGGGCCCATAATATTTGTGTGGTTATAAA

>comp77835_c0_seq3 61_621
CTTCCCACTACTCACAAAGCAAAGAGTTATTATTCTCTGAAATTTATTGTCTAGCTACAA
ATGGACTCTGAGTTATTATTTGCAATTCTATGCCTTCTTCTTCTCTCCATCTCTGCTCAG
AGCTACACTCATCGTGCTCGGGCTCCTTGTAAAGAAATGGTGTTTTACTTTCATGACATT
GTTTACAATGGTGAAAATTATAAGAACGCGACTTCAGCCATAGTTGGCGCGCCAGAATGG
GGAAACAGGACCATAATGGCAAGTCCTAACAATTTCGGGGACTTAATTGTGTTTGATGAT
CCAATTACACTTGACAACAATTTACATTCACCACCAGTTGGAAGGGCACAAGGCATGTAT
TTTTACGATCAAATGGACACTTTTAGTTCTTGGCTTGGTTTCTCTTTTGTGTTCAATAAT
ACTGATTATAAAGGAAGCTTGAATTTTGCTGGTCATGACCCTTTGATGAACAAAACTAGG

GACATTTCAGTAATTGGTGGAACTGGTGATTTTTTTATGGCTAGAGGAATAGCTACTTTG
AGCACTGATGCATTTGAAGGAAGTGTTTATTTTCGACTTCGCGTCCATATTAAGCTGTAT
GAGTGTTGGAAATTACTCTAATTCCTCAATTTTTCCTTTATTTGCATTTTAATCCTTGTT
GAGGGATGACTTCAACTTATAATAAACAATTATTGTCTTTTGTTATGAAATCTCCTATTT
AAGGTGAATGTCTATAATTAGAGAGATTCTAGAGTTCTATTCTTGGTAGCTAAATCATCC
CCTCCCTATAAATAAAGGGGTCTTCTACCATTGTAAACCATCCCTAACAATCTCTAAGTA
TCAATAAGAATACTTCTCTCTCTATTCTCTCTTGTTCTTGCATTATTGTTCTTGTCTTTA
TATTATTTCATAACAAGAAATACTTATTAATTTCATAATAATTCCTAATTGCTTCAGGTC
ATTGCCCTACTACATTGGATTGGATTACAGTCTCAAACTGCTCCCCTCGAACAGATTCTG
TGTCTTTGTTGACAATCCTAATGAATGAGCTATGGATTTGACTTCTGCGATCATGGCCAC
CTGATCATTAAGCTTGTTGATGGCCGAGCCAACGCCCACGCCAGCAGCTCCAGCTGTGAT
CGCCATTGGTGCTGTGACTGCGCTCAATCCAGATGAACACATGACAGGGATATTGACTGC
CCTTGAAATTGAGTAAGCTGCTGCTAGTGTTGGTGTTGCCTTTTCGATTAAACCAAGGAC
ACCGGCCTTTGATGGAGTGGAGCATTTTCCTCCTTCTGTTTGGATAATGTCAACACCTTC
TTGTTCTAATAGCTCGGCCAGCTTGACCTGATCGGGAAGGCTTAGTGTGTGAGGCACAGT
GACTGACAATGTCACTAATGGAAGTATCCTCTTTGTCTCCTTGGTTAGTTTCAGAATCTG
CTCAGGAGAGAATACCAATCCCTTCTCATAGAAGGAGTCATAGTTTCCGATCTCGACCAT
TAAGGCTCCTGCTTCAACAGCAGCGGGAAATGCTGCGGGATCCACAGAAGAAACACAAAC
CGGAAGGTTAGTCAAGCTTATGGCAAGCTTGACCAGCTCAGGTTCACAAGCTATGTCTAC
ATGGGTTGCTCCCCCCTTATCTGCGGCAGTAACAACAGAAGCCACATTCTCTTTGTCTAG
ATTCTGCAATCCTGCAATAATCTTAAGGGCTCTTTTGTCATGAAATTCTTTCAAGACTGC
TTCTTTGGTATGTGAAAGCAGGGCTCTCGTGGTACTTCTTCTTCTTCTAGCAATTGAAAT
GCACCTTGGAAGAACAGGAAGGGAATTCTGATTTGGTTTCAAAAGTATTTGGTTTTTGGG
AGAAATTGTTGTGTAGATTGGGATGCTCATTTTCTTCTTTCTTGTGTATGGAGGGAAATT
TTGGCCAGGAGATAACAAACTTAGCAGCTTTAAGCAGGCTGTGGATATTTCC

>comp74263_c0_seq1 230_2029
AACGGGCAGCTGCAAAATGCAAAAATTATCAACGGCTGTGATAGATTAGAAGCTTCAAGA
GCAACAACTACCGTCCATTCACACTCTTCTCTCCTCACCTTTTGGTTTGGTTATATAACC
TAACCGTATCTTCTTTTTCTCAAGAAGGGATAAACTCACCTACTACAGCTACTTCAACAA
CACTAGTAAAACCCTAAGCTTCAACACTCAACCTCTTTTAATTTTCAAGATGGCATCTAC
TTTTGCTGGTATGTCCTCAGTGGGTCCCTTGGCTGCTCCCAGCACTTCTTCAAACAAGCT
TTCCTCTGTTGCTAATATATCTTCAAGCTCTTTCGGGAGCAGAAGAAATGTTATACTAAG
AAAGGCACGCAGTCCAAAAATTTTCGCTGCAGCAAAGGAGTTATATTTCAACAAGGATGG
ATCAGCCATTAAAAGGCTGCAGGTTGGTATGAACAAACTCGCTGATCTAGTTGGAGTTAC
TCTTGGCCCAAAAGGCAGGAATGTTGTTCTTGAGAGCAAGTATGGTGCTCCAAAGATTGT
CAATGACGGAGTTACTGTTGCCAGAGAGGTTGAGTTAGAGGATCCAGTAGAAAACATTGG
TGCTAAACTAGTGAGACAAGCTGCTGCCAAAACCAATGATTTGGCTGGTGATGGGACTAC
AACATCTGTCGTACTAGCCCAGGGCCTAGTTGCTGAAGGAGTTAAGGTGGTTGCAGCTGG
TGCAAACCCTGTCCTGATCACCAGAGGAATTGAGAAGACCACAAAAGCCTTAGTAGCTGA
ATTGAAGAATATGTCAAAAGAGGTAGAAGACAATGAACTAGCAGATGTTGCTGCAGTAAG
TGCTGGGAACAACCCTGAAGTAGGGAGTATGATTGCTGAAGCCATGAGCAAGGTTGGCAG
GAAAGGTGTTGTGACCCTTGAAGAGGGAAAAAGTGCTGAAAACAGTCTCCGCGTAGTTGA
AGGAATGCAATTTGACCGTGGTTACATCTCACCATACTTCGTTACTGACAGTGAGAAAAT
GTCCGTTGAGTATGAGAACTGTAAGTTACTACTGGTTGACAAGAAGATAACAAATGCAAG
AGATCTTGTTGGTGTCCTGGAAGATGCTATCAGAAATGGTTACCCAATTTTAATTATTGC
CGAAGATATTGAGCAAGAAGCACTAGCCACCCTTGTTGTCAATAAGCTTAGAGGTGCTTT
AAAGGTTGCTGCCCTAAAAGCTCCTGGTTTTGGTGAACGGAAAAGCCAGTATCTTGATGA
CATAGCAACCCTTACTGGAGGCACTGTGATTAGGGATGAACTTGGCCTTACCTTAGACAA
GGCTGACAAGGAAGTTCTAGGCCATGCTTCTAAGGTAGTGCTGACTAAGGATGCCACCAC
AATTGTTGGTGACGGTAGCACTCAGGACGCAGTCAATAAGCGTGTTGCGCAGATTAAAAA
CCTTATAGAGGCAGCAGATCAAGATTATGAAAAAGAAAAGCTAAATGAAAGAATTGCTAA
ATTATCAGGCGGTGTGGCTGTTATTCAGGTTGGAGCACAAACTGAAACTGAATTGAAAGA
AAAGAAACTTAGAGTGGAAGATGCTCTCAACGCAACAAAGGCAGCTGTTGAGGAAGGTAT
TGTTGTTGGTGGAGGGTGCACACTCCTGAGACTTGCTTCTAAAGTTGATGCCATCAAGGA
GACCCTTGAGAATGACGAGGAGAAGGTTGGAGCTGATATTGTTAAAAGAGCATTGAGCTA
TCCATTGAAGTTGATTGCTAAAAATGCTGGTGTTAATGGAAGTGTTGTCAGTGAGAAGGT
GCTCTCTAGTGACAATCCGAAATTTGGATACAATGCTGCAACTGGAAATTATGAAGACTT

AATGGCTGCTGGTATTATTGACCCAACAAAGGTGGTGAGATGTTGTCTAGAGCATGCGTC
ATCAGTGGCGAAGACATTCTTGATGTCAGATTGTGTGGTGGTAGAGATCAAGGAGCCTGA
ACCCGCAGTTGCTGGCAACCCAATGGACAACTCAGGATACGGTTACTAGATGATTGAATG
AATGCTACTGATAGATAGAAGAGGAGAACTGCCCTGCGAGATGATAGTGGTAGATGAACA
ATGTCTGGTGGCAATGCTTGAAAGCTTTTATTTTTGTAAAACTTTAGGTATTCTTTCTGG
GACGATATACGTAAATCCTGGTGGGAGTAGCCATCAGGGTTCGGCACCGTATGAACATGC
AATTCAAATATATAATTGCAGTATCAGTGGATGTTTATTGAGTGGCAGAGTGGCAGAACA
GAAATAAATCTTTTATATAGTTTCTCATATTGCAACTTCTACTACCTCTGTTATAGTCTT
ATGGGAATACTGAAGGGAAGAAAAATTGAAGAAAAAATGTGAAGCAACATATAGGTTACA
TCTTGGTGAGGAATTGCAGGATTCACTACCCGTTTATAATCAAACGATTAAGAGAGGGAT
ATAAAAGTGCTCTAGTAATGAAATATTAACTTTCGATTTCCGTAGTTCAACCTTGTCACC
ATTTA

>comp78088_c1_seq1 1536_2366
CTTTATTATCATTTTGATTTCGGACACAGCAAGTTTGGTGACAATTAGATGACCTCGGGA
TGTGCAAAACCCTAGTATATTTTTGTCGGGTTTTTGCGGCAAAAAAACGTCACTGTACCT
TCCCTGAAAAATCTTTACCCTACGTATGCATTACCCTTCATAGAAATCTCACCATCTCAT
TTTTTTTTCTATCATCCATTCACAAAGATTGGATTTGTATAAGACATTTGGCAATACACG
AACAGAAAAAACACAAACAGAATATACTACAGGGGGAATCCATGGCTACTGACCACTCTG
ATGATTTAGACCAACTTCTTGACAGCGCTTTGGATGATTTTCAGAGCCTCAATCTCACTT
CTGCTTCTCAAAGAAATGGGGATGAAGAGGACAAGAAAGAAAGTTGTTGTATGGCTAGTG
AGGTTCAAGGGCTAGGGATGTCGTTGCCCGATTTGAAAGCTAAGCATAAGGGGAAGCAAA
AGGCTTCTTCTAAGGACTCACATGTCTCTGAAGCTCTTGATAAGCTTAGAGAGCAGACAA
GAGAGGCTCTTAAGGGATTAGAATCAGTGGCTGTGCCGAGACCCGGTTTAGAGAGCTTTG
GAAGTGATCCAATGATGGAGGAGTGGGCCAAGCAGTTTGAGGAGCTTGCTGGTTCTCAGG
ATATGGAGTCTATCGTAGAGACCATGATGCAGCAGCTTCTTTCAAAGGAAATTCTTTATG
AACCTATGAGAGAAATTGGAGAAAGATATCCAAAGTGGTTGGAAGACAATAAAGCCAAGT
TAAGCAGTGAAGATTATGAACGTTATAGACACCAGTATGAACTTATAAGAGATCTGAACA
AAGTTTACGAGACTGAACCTAGCAACTTCAACAAAATTGTAGAGCTTATGCAGAAAATGC
AAGAATGTGGCCAACCGCCAAATGATATTGTTCATGAGCTTGCTCCAGACTTTGATATAT
CATCTCTTGGACAGCTATCCCCAGAGATGTTGGAGGGCCAACAGAACTGCTGTGTTATGT
GAAAACTGAAATGTCCCCGTCCGAATGTCCTGCTTGTTTTCTTTTTCCCAGGTTGCATAG
AACATCATTTATTTTGCCCTGGATTCCATTTGTATTCTTATTGCTCTTAATATCTCGGTG
CCTTATTTCCTTTTCTTTTTTGGTTGCAATTCTTAAAATGGTGGAATAAGAACAACACAA
GATACGGCCAGCCAGTGCTTGAAGTTTAAAACAGTAATAGATGATGTAATAAAAGCATCG
ATCTAGTAAACAAATTGATTCACTCCATAGTTGCATCGATGATTTTCACTTGTTAATATA
TAGAGTCCAATAATCATATTTTGTGTTGATTCAAAATTGGGATTCCCACATTCATGCGAG
ATAACATAAAGTTAAAAAAGAGATCACTATCATTTCCTTTATAACTATGATTACAATCAT
TTTATTTTATTTCTATATAAACAAAATGAATGGAAGGTAATTAAAGAATTGATTAAATGG
CAACAGAAACAAACAGTATTCTGTGCAAAATATGTTTAAGCGTCAGAGGGAGCAATCCAG
TGGCGTCCGTTGACATAGCTCTTGGCTAGAAATGGGTGGGCCGTGTCGTAATCCAGCTCC
CGGGCCCATGATACTCCCATCACTGCTGCTGCTCCTGGCCCCCAGCATTTGTACACTCCA
AAAAATGCTGTCTTGTTCTTGTTGCTGACGTGGTCCCAGTCATCCCAGCCACCATGGGCC
ACAACGTCATCGAAGTAGGTGTAGGAATAGACAATTCTGGAATATTGGCCCATAGCACGG
CCCACATAAAGTGGGCCAGACCCTGTAACCCTGCAGCCCATGAATGCAAATCCTGACTTG
TCGTCTGGGGATCTTCTATCTTGGGCTGCTATGGACCCAAATCTTGTGGCTATTGAATGC
AGCTCGCAGTCCTTATACATGGAGCGACCATTGCCAAAAATGAAGTCAATAGAGCCCTCA
ATGTAACATTCTTTGAAATAATGGCGGCCGGCGTCGTCGCAGAGAGTGTCCTGTGCGCCG
TAGAATCCACAGCCGGAGAAGAAAGCCTTGTCGCCTGATATCCGAAATGCCACTGCTTGC
CATCCCTGCATTCCTGGCAATGGGGCTGGTGCTGTGTTCTTGAAGCTAATATTTCTAGCA
GAGAAAAAAGGAGCATAAACAGTGACAGAAGCAGTTTGATAGGTACGAAGCTGTTGTCCA
TCTGCGCCTTTGTCTTTTGCTCTATCATGCCATTCTATTATCGTCACTTCCCTTCCTGCT
CCTTGAAATGTTATGTATGGCTTTGTTTCTGCCACCACCACTTTCTCTATGTAATAACCA
GGGCAAATGTCAATAATGACATTCATTCGGTTGTTGTGTGGAATAGAGTCCACAGCAGCC
TGAACTGACTGGTAATCTCCATAACCATTAACATTAACTGAGATATGGCGAGTGCCTGAA
GGTCCTATCCATTTATGGCGGCGACTGGATGCTGCTGATACACTGCTACAATCTGCAAAG
AAGAAGAAGAAGAAGAATAAATTGTGCAAAGGGGTTTATGAATTTTTTTTCTTTTTTTCT
TCTTCTTTTTTGGACAATGTTTGTTCAGATCTAAAAGTTTGAATGATTCTATGTCGTAGA

AGTTAAGGAATGAGGAATTTGGGAGTTTTGACTGTATTTCCCCCGGAACCTCCCCACGAA
AAGAAAAAAACAAACAATAACGCGATAAAAGACAATACAAAATAAAAAGACCAAAAAAATG
GAATTAAAATTTACATCGTTTGCATTTTTATTGTTATTTTTAGCACGACTTGGAGACGCT
CAGCCAACAGCTTCACCAGGAGTCTTTGATGTTACCAAATATGGCGCAACAACTAATGGC
GATATCAGTAAGGCTGTGTTAGATGCATTTAAAGAGGCATGTGCAGCAACAAGCCCAAGT
ACAATAGTAATACCAAAAGGTACATATCAAATGAATCAAGTAAAACTACAAGGTCCATGC
AAAGCACCAGTTGAGCTACAAGTTCAAGCCATTTTGAAAGCTCCTGCAGATCCTAAACAA
CTCAATGCAGATGTTGAATGGTTTACTGTTAGTTATATTGATCGTTTCACACTTTCTGGC
GGTGGTGTTTTTGATGGCCAAGGTAAACAAGCTTGGGATCAAAATAATTGTAAGGACTCC
AAAAGTTGCAGCAATCTTCCCAATAATTTGAGCTTCAACTTCCTTACAAATTCCACAATC
CAAGACATAACTTCACAGGACAGTAAACTATTCCATATCAACGTGAATGGTTGCAAGAAC
TTGACATTCATCAGAGTAAACATCACAGCTCCAGCAGAAAGTATCAATACAGATGGCATT
CACGTCGCACGATCGAGCAACGTGAACATTACAGATTGTGTAATCGGTACTGGAGATGAC
TGTATTTCTGTAGGAGATGAATTGGAACAGCTTCATATTACTAGAGTT

>comp85070_c0_seq1 3_2648
GTTCCAAGAAAATAAGACATAATCTGTTAGCTTTTCTCTTTGCCATTCTCATGGATTCCT
CATCAATCTCCTCCATCGAATCTCTAGTCAATGAGATCAAGCAAGAGATGTTTTCAAACC
AAGAATTTAACACTTTTCTTACCCCAATATCTGCCTATGACACTGCTTGGTTGGCCATGA
TTTCTTATAATAATAATCAAGAAAAAGCCATTAATGGTCATTCTTTTTCTGGCCCTATGT
TTGAGAGTTGTTTAAATTGGATTCTCAACAACCAAAACGAGCAAGGATTTTGGGGAGAAT
CCAATGGTGAAAATCTTCCCACCATTTCTTCTCTTACTGCTACTCTTGCTTGTATGATAG
CACTCAAGAAATGGAATGTGGGTGAAACCAACATCAAAAAAGGTTTGAAATTTATTCATG
CTAATACGGAAATAATTCTGAAAGAGAATTCTACACAATTTCCACGTTGGTTCACCATTG
TTTTCCCTGCAATGGTTCAACTTGCTAAATCAGTAGGCCTGAAGATCATTTTCATTAATG
GATCAGAAAGATTACTTTCTGACGTGGCCATGAAGAGGAAATTAATTCTTGAAAGTGAAA
ACCTAATGGATATAACAGCAGGGCATGATCAACCACTTTTGGCATATCTAGAGAGCTTGC
CAAATTATTTTGTGGATGAAAAAGACCAAATCCTAAAACATTTAAGAGAAGATGGTTCTT
TGTTTCAATCTCCCTCTGCTACAGCACAAGCATTCATGGCCACAGGAAACCAAAAATGTC
TTGAATATCTCATATCCATTGTTCAAAAGTGTCCAAATGGAGTACCTTCAAGGTTTCCAG
TGGATGAAGAGCTAATAAATCTTTGCATGATTGACCACATTCAGAGAATGGGATTGGCTA
ATCACTTTAACAAAGAGATTGAACAGATTCTTGGCCAAGTTAATAAAAATCAAATGAATA
ACCAGAAGGAGTACTTATCAGAAATAAGTACTTTACCTGAGAGATTATACAAGGACTCAT
TGGCTTTTCGACTTCTACGTTTGCAAGGCTATAAAAAAAATCAAGGAAGTTTTTGTTGGT
TTGTTCATCAACCGCAGATAAGGGCCTACATAGAAAAGAATCAAGAACGTTTTACATGTG
TGATGTACAATGTGTACAGAGCTACAGATCTTATGTTCGAAGGAGAAACTGAAATGGAGA
AAGTACGATCTTTTGCCAGAAATTTTCTTGAACGTTCAATGAAAGTCGTGAACAATGAGG
ACAACTTATTACTACTACCTTCACTTCAAAAAGTGATTAAGCATGAGTTGAATGTTCCAT
GGGTTGCTCGACTAGAACATCTTGATCACAGATTATGTATTGAACTAAGTCAATCTCTCC
CACTTTCAATTGGAAAATCCGCAGATTATTGGTTGTCTTGTCTGCACAATGACAAATTAT
TGAAATTAGCAGTGCAAAATTACGAATTTCGCCAGTCAGTTTACAGGACGGAATTGGAAG
AACTAAAGAGGTGGTCAAAAGAGAAAGGCCTTGTAGACATTGGATTTGGGCGAGAGAAAA
CTACATATTCCTATTTTGCAAGTGCAGCAAGCAGCAGCAGTTTTCTACCTTTTGATTCTT
TACTTCGATTGGTTGTTGCAAAATGTTCCATAGTTATTACAGTTGCTGATGATTTTTATG
ATGAAGAAGCTTCTTTAAATGACTTGCAAATCTTAACCGACGCGGTGCAAAGGTGGGATG
GAAGTAACCTTGATGGACCTAGTAAGATCATATTTGATGCACTTGATGATCTTGTGAGCG
ATGTTGCTAAGTTGTACCACCTTCGACACGCAATTGACATCATGCCCGAACTTCGACATC
AATGGCAGGAGACATTTCTTGCATGGATGATGGAAAGTACGTGGAGTAATAATGTAGCCA
TGCCATCTAGGAATGAATACCTAGAAATTGGCATGATATCAATCGGTGCACATATTTTGG
TTCTTCATGCTGCTTCTCTTGAAAATCCAAGTTTGCCAAGGGAAAAACTTAGGCCAATTA
ATGGCCACTATGAAAATATTACAAAGTTGCTTATGACCACCACTCGTTTGTTGAATGACA
TCCAAAGCCATCAAAAAGAATGTGAAGTAGGGAAAATGAACTACACATTACTTCACATGA
ATGAAAATCCAGGGGCACAACTTGATGATTCAATTGAATCTGTCAAAGAGATTTTGGCCA
ACAAGAAGAAAGAATTTCTTGAAAATGTTCTAATGGATGGATTCAGTGATATGCCAAAGA
CATGCAAACTACTTCATCTTTCTTGCCTAAATGTGTTTCACATGTTTTTCAATACTTGCA
ATTTATTTGATACCAAAGCAGCAATTCTTGAAGATATAATGAGAGCTATTTACATTCCTC
TTCAAGAACAAACCTCAATTACACCATTAAAAACTTCTCCAATTATAACACCTCAAGAAA
AGAAGAAGAAGAAGATTGAGAATATTCCAACTAAAGTTTCTGCTTGTCTCAATGGGA

AAAACTTCAAACATCAAGTTGGCATTGGAATTGGCATAAGTTTTGTTGGAAGGCAAGCTC
CGAAGAATAATCCTTTTGAGAATACGAAAGGATTTGCTTCACAAAAGTTAAGATCATGTT
TTATATAATTTCATTAGTAATAGTTGTTGGAAGAACCTATTAGCTTATTATTTATGTTGT
TGTTATTCTTGTATAATATGTAATATTGTAGTGAAAAATTAAACTCCATTTGCAGTTCCT
CTCTAATAAGTATCACTTCTATATTTTCATTAAAAAAAAAA

>comp88815_c0_seq1 2_1909
AGAGAATAGTCTTTGCTCCATATTCTATATAAATACTAACCCTAGATTGTTCATGAACTA
CACCCTAAACCAATTAAAAATTATAATGGCTTCTCTTCACCTCCCCTCTACCAATACCAT
CTCTTCTTCATCTTCTTCTTCAACTACCACAACTCTTTCCAATTTGCATTCTTCTCCTTT
CTTTACAAAGACCTCAAAAGTTTTCACTTTAAGAAAGTACGGTAACCATCGTTTCCAAGT
CTCATGCAAGGGTACAGAAGATGACCAAACCAATAACACTTCGAAAAATTCTAATTCTTC
AAACAATAAGATCATTGATAGAAGAAACATGCTACTTGGATTAGGAGGTATTTATGGTGC
TGCTACTATTGTTGGTGGTCATCCCTTTGCCTTCGCCGCTCCTGTGCCCGGACCTGACGT
TTCCAAATGTGGCGCTGCAAATTTGCCACCAGGTGCAGCACCAGTCAACTGTTGTCCTCC
AACAACGGCGAATATCATTGACTTCCAACTTCCACCACCGTCAACTACCCTCCGCACTCG
GCCTGCAGCTCATGCCGCCGATAGTGCCTACATAGAGAAATTCAATAGAGCCATTCAGCT
CATGAAACAACTTCCAGATGACGATCCACGTAGCTTCAGGCAACAAGCAAATGTTCATTG
TGCTTACTGTGATGGTGCTTATGACCAACTAGGCTTCCCAAACTCTGAACTCCAAGTTCA
TTTCTCTTGGCTTTTCCTCCCTTTCCATCGTTGTTATCTCTACTTCTTCGAAAAAATCTT
GGGAAGTTTAATAAATGATCCTACTTTCGCTATCCCATTTTGGAACTGGGATCATCCTGA
TGGCATGAGACTTCCTGCCATGTATGCGAACCGTAGTTCTTCTCTCTTCGATCCTCTCCG
TGATCGGAGGCATCAGCCTCCGGTCATGGTTGATCTCGACTTCAATGGAACGGATCCTAA
CATAAGTAACGCTCAACAAACTTCCCAGAATCTGACAATCATGTATAGGCAAATGGTTTC
TCTAGGAAGCACTCCGGCGACTTTCCTCGGAGACCCTTACCGTGCCGGTGGTGAGCCCAG
TGGAGGTGGGTCCCTCGAGAACATTCCTCATGGTCCGATTCATGTTTGGACCGGTGATAG
AACCCAACCTAATTTTGAAAACATGGGAGATTTTTATTCAGCTGCTAGAGACCCTATTTT
CTATGCTCATCATTCTAATATTGATAGATTATGGAGTGTTTGGAAAACCCTAGGTGGAAG
ACGTCAAGATTTTACTGACCCTGATTTTTTAAATGCTTCGTTTTTGTTTTATGACGAAAA
TGCACAAATGGTACGTATTAGGACACGTGACTGTTTGGATACAACAAGACTTGGATATGT
TTATCAAGGTGTAGCTAATCCGTGGATAAATTCTCGTCCAAGGGCTAGGGTTTCAAGTGC
TTTGAGTAATGTACGGAGACTTGTTGAAGCAAGAGCAGCTGAAAATTTTCCAAGTGCAAA
AGATGTTTTCCCAACGAAACTTGACCATTTGATAAGAGTTATGGTGAAAAGGCCAAATAA
GAAGAAGAGAAACAAGAAGGAGAAAGAAGCAAAAGAGGAGATTTTAGTGGTTGAAGGGAT
AGAGCTGGAAACTGATGTTTTTGTCAAGTTTGATGTGTTGATAAATGATGAAGACGAGAC
TGTGATTTCGCCGAATAATGCTGAGTTTGCAGGTAGTTTTGTGAATGTGCCACATCATAG
TCATGGTAAGAGTGACAAGAAACGTAAGACTAAGTTGAAGTTGGCTATAACTGAGCTGTT
GGAAGATTTGGATGCTGAGGATGATGATCATGTGGTGGTGACTTTTGTTCCAAAGAATGG
TTCTGGTGCTGTGAAAATAGGAGGTGTCAAGATTGTGCTAGAGGATTGATTAGTAGCTTG
GCACACTAAATTTCTAGGAGGGATGGGAAGAATGGAACCACAAAAATTTATTATATTTTC
TGTATTCCCATTATGTTTGAGGATTGATTATGAATAGGAAAATAAAAGGATTTTAATTAA
CTTCCTCAATTAATTAAGCATTTTAAGTTTCTTGATATTGTAAGCGTGTTTGAATGGAAT
AAAGGAAGAAAACCACTTACATGGATATGTTGTCATTTCTTCTAAATGTATAACTACAAC
TAGTAATTTTCATACTAAGTAGAATGATTTCTCATTTTACATTATTTCCTAGTGTAAGGG
TCGTCGATTGGTGGCGATGTTAGTCCAACACGCACCTCCAGGAAAAAG

>comp63365_c1_seq3 3_242
GAGAGGCTATTTCCAAATAGACCCTTGGCATCCTTCCCTCCAAGAACTCCCCACCTTGCT
CTTGGGTGACTCGAACTTACAACCTCATGCTTAGAAAGACAAAATTCAACATCAGTAGTT
TTGCTCGAAATACTGAATATGGTATAAGGTACCAAATACAGCTTGTGAATTCGTTCTTGT
CTCTCCAGCTTGGGTAATTTTTTTTCTGTTTGTTGGTGCAGCCACATGGATTCTTTAACT
GC

>comp67145_c0_seq1 106_930
GAAACAATGATAAATCTCAAATCATCCTCTCTTTTCCCATCTTCTACTGGATAAGGAAAC
CCAAAAATAGAGCCCTTTCTCCTTCTCTTTTCCCCTCTCTCAGCCATGTCGCTCTCTTCT
TTCTCCACAGTTCCCACCGCCAAATTCCGCCTCAAAACCACCCCGTTGACCCTCCACTCT
CCATTCCTCCACCGTCTCCCTCTCCGCGCCCTCAAAACCCACAAACCCACTTCACTCCAC

ATCTCAAAATCCTCAATCTCCGCCTCACTCGAAGCCGGAGTTGGCGTAATGGCAACAAAA
CTCGGAATGATGAGCTTCTTCGAGGAATCAGGAACAGTTGTGCCTGTAACAGTTGTTGGG
TTTCGAGAAGGCAATATTGTTACTCAAATTAAAACTGAGGCTACTGATGGGTACAACGCT
GTACAAGTTGGGTATCGACGTGTTAGGGATAGAAAGCTGACTAAACCCGAAATGGGCCAT
CTTGAAAAGTCTGGGATTATTCCTCTCCGCCATCTGCAGGAGTTTAGGCTTCAGTGTATT
GATGGGTTTGAGGTTACGCAAAAGCTTGATTTTGGTGAGCTCTTTAAAGAGGGTGATTTG
GTGGATGTCTCCGGTACAACTATTGGAAAAGGATTTCAAGGTGGTATAAAAAGGCATAAC
TTCAAGAGGGGTCAAATGACTCACGGATCAAAGAGTCATAGACAACTTGGATCAATTGGT
GCTGGGACAACACCCGGGCGTGTATACAAGGGCAAGAAAATGCCTGGAAGAATGGGAGGC
ACAAAGAGAAAGATAAGAAAGCTCAAGATTGTCAAGATCGATGATCAACTCAACATAATA
ATGATCAAAGGAGCTCTTCCTGGTAAGCCAGGAAATCTTTTGCGAATTGCTCCAGCCAAG
ATTGTAGGCAAAAATATCCCCAAGAACTAGTTTCTCTATATTTCAAGCGATCATGTAATT
TTATTCTTTTAAAATATGTAGGTTTGAAATTTTGCTAATTGCATAGGCTGGATAAATATT
CCACCTTCAAGTTCTATTTGTCGGATTTCTGACTAAAACTCATCATTGACATTCTCAATA
TCTTCTGTGTATGAGCAATCTTAGTAGGGCGTAGATAATTCTGATTGAGATGATCAAACC
TGCCGTGATGACTGATTGAAGTGTCAGTTCTGCTGCTTGTGAGCTATTTGGGGATGTTAA
CCGGTGGAAAAGTTAGAAATTTTTCCATGGGTATTCAAATTTGAAAAAATGTGAAAAATA
AATTGAGAGAAAGAGTGTTCAATTGTGT
